# Epitope-Based Vaccine of a *Brucella abortus* Putative Small RNA Target Induces Protection and Less Tissue Damage in Mice

**DOI:** 10.3389/fimmu.2021.778475

**Published:** 2021-12-21

**Authors:** Karen Cristina Oliveira, Gustavo Andrade Brancaglion, Natália C. M. Santos, Leonardo P. Araújo, Evandro Novaes, Renato de Lima Santos, Sergio Costa Oliveira, Patrícia Paiva Corsetti, Leonardo Augusto de Almeida

**Affiliations:** ^1^ Laboratory of Molecular Biology of Microorganisms, Federal University of Alfenas, Alfenas, Brazil; ^2^ Department of Biology, Federal University of Lavras, Lavras, Brazil; ^3^ Department of Clinic and Veterinary Surgery, Veterinary School, Federal University of Minas Gerais, Belo Horizonte, Brazil; ^4^ Department of Biochemistry and Immunology, Federal University of Minas Gerais, Belo Horizonte, Brazil

**Keywords:** *Brucella abortus*, vaccine, immune response, reverse vaccinology, brucellosis, apolipoprotein N-acyltransferase

## Abstract

*Brucella* spp. are Gram-negative, facultative intracellular bacteria that cause brucellosis in humans and animals. Currently available live attenuated vaccines against brucellosis still have drawbacks. Therefore, subunit vaccines, produced using epitope-based antigens, have the advantage of being safe, cost-effective and efficacious. Here, we identified *B. abortus* small RNAs expressed during early infection with bone marrow-derived macrophages (BMDMs) and an apolipoprotein N-acyltransferase (Int) was identified as the putative target of the greatest expressed small RNA. Decreased expression of Int was observed during BMDM infection and the protein sequence was evaluated to rationally select a putative immunogenic epitope by immunoinformatic, which was explored as a vaccinal candidate. C57BL/6 mice were immunized and challenged with *B. abortus*, showing lower recovery in the number of viable bacteria in the liver, spleen, and axillary lymph node and greater production of IgG and fractions when compared to non-vaccinated mice. The vaccinated and infected mice showed the increased expression of *TNF-α*, *IFN-γ*, and IL-6 following expression of the anti-inflammatory genes *IL-10* and *TGF-β* in the liver, justifying the reduction in the number and size of the observed granulomas. BMDMs stimulated with splenocyte supernatants from vaccinated and infected mice increase the CD86+ marker, as well as expressing greater amounts of iNOS and the consequent increase in NO production, suggesting an increase in the phagocytic and microbicidal capacity of these cells to eliminate the bacteria.

## Introduction

Brucellosis is a global zoonotic infectious disease caused by bacteria of the genus *Brucella*. The disease is a serious public health threat worldwide, particularly in developing countries of Central Asia, Africa, South America, and the Mediterranean region (1). Brucellosis affects mammals, causing abortion and infertility in affected animals. This infection can spread from animals to humans, mainly *via* the ingestion of unpasteurized milk or dairy products and, to a lesser extent, *via* direct contact with infected animals (2). In humans, brucellosis can cause a severe febrile disease with various clinical complications ranging from mild to severe symptoms including undulant fever, joint pain arthritis, endocarditis, and meningitis (3–5). The genus *Brucella* includes Gram-negative facultative intracellular bacteria from Alphaproteobacteria, and, currently, the genus consists of 12 species that are classified based on their host preferences (6). Although several *Brucella* species are potentially zoonotic agents, *Brucella melitensis*, *Brucella abortus*, and *Brucella suis* are considered the most pathogenic *Brucella* species that have a serious impact on public health and the livestock industry (7, 8), with *B. abortus* being the most widespread throughout the world, according to the World Health Organization (WHO) (9, 10). Since brucellosis is the most common zoonotic disease worldwide and has become a serious concern in recent years (11), the strategy used to control brucellosis depends mainly on the massive vaccination of domestic animals to prevent the disease from spreading to healthy animals and humans (12, 13). Almost all vaccines against *Brucella* spp. are live attenuated strains with extensive global use but with various drawbacks, such as pathogenicity to humans and residual virulence in animals, which can cause abortion, orchitis, and infertility (14–16). Moreover, it is difficult to differentiate infected animals from vaccinated animals by serological tests. These drawbacks have prompted several research groups to attempt the development of safer vaccines.

Subunit vaccines have promising applications with the advantage of being safe, cost-effective, and efficacious. During the past two decades, various antigens have been extracted from *Brucella*, such as Omp19, Omp25, L7/L12, P39, SodC, InpB, AsnC, and TF (17–24). These available antigens have been shown to provide protection against *Brucella* infection by reducing the organ’s bacterial load in mice. While such findings are highly promising, subunit vaccines using known antigens cannot provide the levels of protection conferred by live attenuated vaccines (25). Further investigation is needed to identify novel antigens, and increase vaccine efficacy. In contrast to the conventional vaccine development that requires cultivation and extensive empirical screening, reverse vaccinology (RV) is an interesting *in silico* approach to identify protective antigens using pathogen genomic data (26–29). RV has been implemented to identify protective antigens of numerous pathogens, including *B. abortus* (30–32).

The main goal of this study was to screen potential antigens in the genome of *B. abortus* using RV as a search strategy and subsequently evaluating the immunogenic capacity of the peptide in an animal model. We used an *in silico* methodology to select epitopes candidates based on their biological characteristics strongly associated with protective antigenicity from putative targets of small RNAs expressed in infected BMDMs. From these predictions, a transmembrane epitope of apolipoprotein N-acyltransferase was selected for efficacy verification in a mouse model showing promising results to be used as an epitope-based vaccine against brucellosis that may induce robust immunity against the bacterium.

## Material and Methods

### Ethics Statement

This study was carried in strict accordance with the Brazilian laws 6638 and 9605 in Animal Experimentation. The protocol was approved by the Committee on the Ethics of Animal Experiments of the Federal University of Alfenas (CEUA 16/2020).

### Mice, Cell Culture and Bacteria

The strain C57BL/6 mice aged 6–8 weeks were purchased from the Federal University of Minas Gerais animal facility (UFMG, Belo Horizonte, Brazil). Bone marrow cells were obtained from femora and tibia of mice and they were grown in bone marrow-derived macrophages (BMDMs) as previously described by our group (33). *B. abortus* virulent strain 2308 was obtained from our own laboratory collection. They were grown in *Brucella* broth medium (BD Pharmingen, San Diego, CA, USA) for 3 days at 37°C.

### BMDM Infection With *B. abortus*

BMDMs were infected with virulent *B. abortus* strain 2308 at a multiplicity of infection of 100:1. Bacteria were centrifuged onto macrophages at 400 × g for 10 min at 4°C and then cells were incubated for 30 min at 37°C under 5% CO_2_. Macrophages were extensively washed with HBSS to remove extracellular bacteria and incubated for an additional 90 min in medium supplemented with 100 μg/mL gentamycin to kill extracellular bacteria. Thereafter, the antibiotic concentration was decreased to 10 μg/mL. Thirty minutes after infection, BMDMs were washed three times with HBSS before processing following homogenization with 100μl of LS TRIzol^®^ reagent Invitrogen (Waltham, Massachusetts, EUA) for total RNA isolation.

### Small RNA Sequencing and Bioinformatics Identification

The construction and sequencing of a strand-specific small RNA (15–50 nt) library was conducted by FASTERIS SA (Plan-les-Ouates, Switzerland), based on the Illumina^®^ TruSeq^®^ Small RNA Library Prep Kit for Illumina HiSeq 2000 sequencing (Illumina Inc., San Diego, CA, USA). To remove adapter sequences from small RNA raw reads of sequencing, the Cutadapt tool was used (34) and sequencing quality was analyzed using the Trimmomatic V0.32 tool (35). Reads were mapped to the *B. melitensis* biovar *Abortus* (strain 2308) genome using the Bowtie program (36) to report the best alignment for each read allowing a maximum of one replacement per alignment. Using BedTool, reads mapped at alignment were analyzed to determine the depth of coverage across the genome determining the hotspots areas of small RNAs (37). Coverage with more than 50 reads and areas with a distance of less than 50bp were joined as the same hotspot area.

### Bioinformatic and Reverse Vaccinology

**Figure 1:**
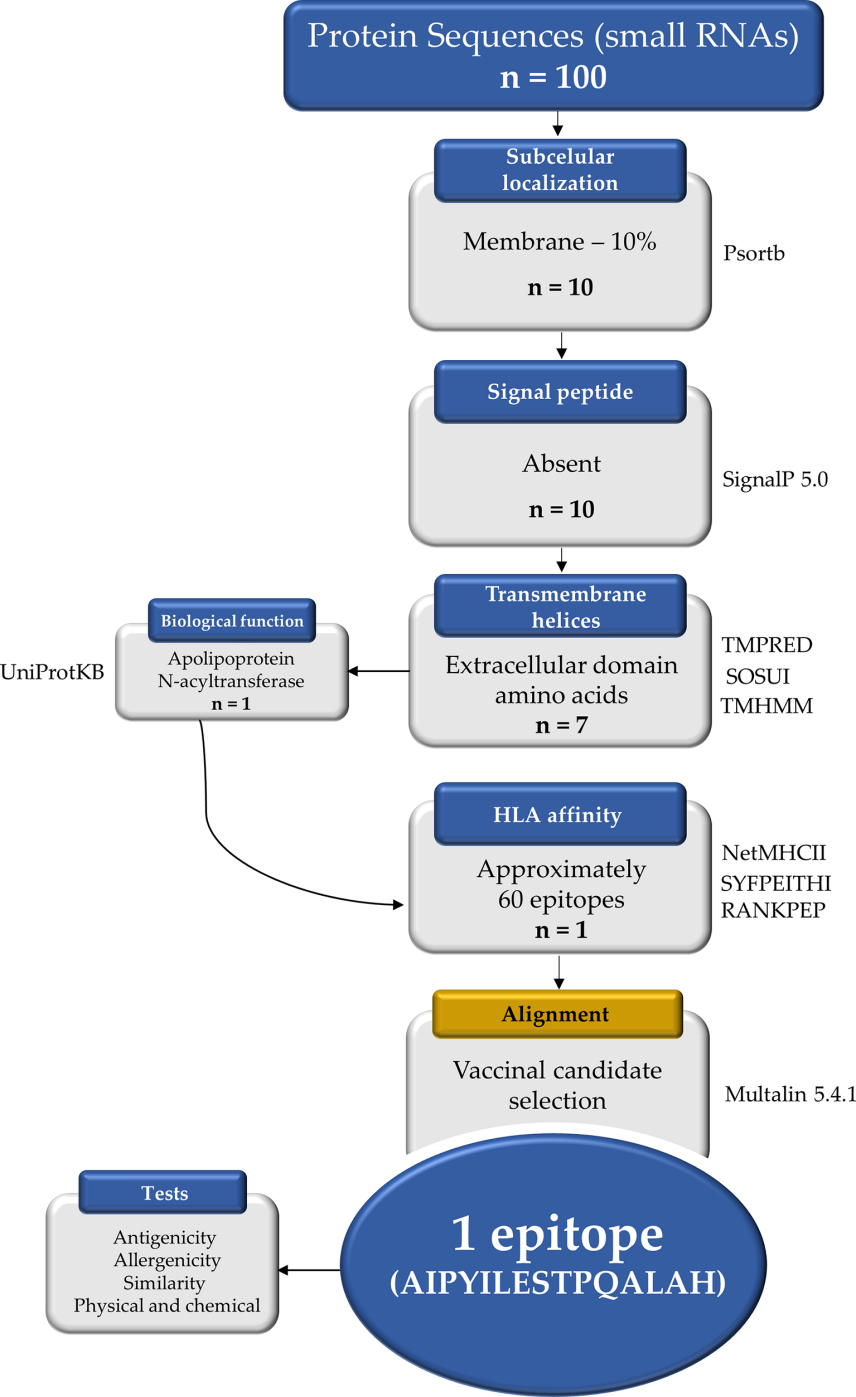
Reverse vaccinology protocol workflow applied in this study to select a vaccinal epitope candidate. The one hundred protein sequences were obtained from the NCBI and ten proteins were localized in the plasma membrane by the Psortb tool. The TMPRED, SOSUI, and TMHMM tools identified 7 proteins with extracellular amino acid domain, but when analyzing the biological functions in the UniProtKB, only one was selected. Approximately 60 epitopes from the extracellular portion showed strong binding by HLAs according to the tools NetMHCII, SYFPEITHI, and RANKPEP. Using the Multalin tool the alignment was performed and the most promiscuous candidate among the epitopes was selected. n, number of proteins.

To assess the putative targets of those small RNAs highly expressed by *B. abortus* during infection, one hundred coding genes were obtained using the intersect BedTool to indicate the overlap between small RNAs and coding genes (37). As the overlaps showed the possibility of affecting more than 50% of the *B. abortus* coding genes, which target genes contain the hottest hotspots (i.e., with the highest average coverage), by Watson-Crick base pairing complementarity was evaluated. The proteins from the putative target coding genes were filtered by assigning selection criteria according to biological characteristics: (1) subcellular location (SCL) using Psortb v3.0.2 program (38), (2) the presence of a signal peptide using SignalP 5.0 Server tool (39), and (3) the presence of transmembrane helices and exposed regions using three tools: TMPRED, SOSUI 1.11, and TMHMM 2.0 (40–42). The obtained proteins were then analyzed for biological function using an online tool UniProt Knowledgebase (UniProtKB) and only one of the 7 was selected (43). The selected protein apolipoprotein N-acyltransferase (WP_002965220.1) was used to identify epitopes composed of fifteen amino acids present in the extracellular portion of Int. The extracellular portion of the surface-associated target protein was subjected to the sequential mapping of epitopes predicted to bind tightly to major histocompatibility complex (MCH) class II, using the tools NetMHCII 2.2, SYFPEITHI, and RANKPEP (44–46). To obtain the best candidates of putative immunogenic epitopes from Int, using Multalin 5.4, the obtained epitopes were aligned, and the linear amino acid sequence including approximately 15 amino acids that presented a repeats sequence was selected (47). For the epitope antigenicity test, the VaxiJen 2.0 server was used with the 0.5 and “probable antigen” cutoff (48). For the allergenicity test, the server “AlgPred: Prediction of Allergenic Proteins and Mapping of IgE Epitopes” was used with the hybrid method that consists of using the following five tools available on the server: SVMc, IgE, epitope, ARPs BLAST, and MAST (49). For the epitope similarity analysis, the Protein BLAST tool available on the NCBI platform was used (50). For the physical and chemical properties test, the ExPASy server ProtParam tool was used with a cutoff of 40 for structure stability values (51). The pipeline of bioinformatic analysis is depicted and summarized in **Figure 1**.

### Real-Time RT-PCR for Apolipoprotein N-Acyltransferase Expression

Thirty-minute *B. abortus*-infected BMDMs or the exponential growth of *B. abortus* in *Brucella* broth medium total RNA were extracted using TRIzol reagent (Invitrogen). Reverse-transcription of 100 ng from total RNA was performed using random primers according to the Illustra™ Ready-To-Go RT-PCR Beads kit (GE Healthcare, Buckinghamshire, UK). Real-time RT-PCR was conducted in a final volume of 10 μL containing the following: SYBR^®^ Green PCR Master Mix (Applied Biosystems, Foster City, CA, USA), with cDNA as the PCR template and primers to amplify specific fragments corresponding to specific gene targets: IntF: 5`-CTGATGTGATTGTCTGGCCG-3`, IntR: 5`-CCTGAGGTGTCGATTCCAGT-3`, *Brucella* 16S F: 5`- TCTCACGACACGAGCTGACG -3`, *Brucella* 16S R: 5`- CCTGAGGTGTCGATTCCAGT -3`. The PCR reaction was performed using the ABI 7500 Real-Time PCR System (Applied Biosystems, Foster City, CA), with the following cycling parameters: 60°C for 10 min, 95°C for 10 min, 40 cycles of 95°C for 15 sec and 60°C for 1 min, and a dissociation stage of 95°C for 15 sec, 60°C for 1 min, 95°C for 15 sec, and 60°C for 15 sec. All data are presented as relative expression units after normalization to the *Brucella* 16S gene. PCR measurements were conducted in triplicate. The differences in the relative expression were analyzed by Student’s *t* test with a two-tailed distribution (p < 0.05 indicates statistical significance).

### Int Structural Modeling and Epitope-MHCII Docking

The Int protein from *Brucella* was retrieved *via* the NCBI database, followed by molecular modeling by homology *via* WebServer Phyre², which uses the Markov model for the best possible global alignments to generate the most accurate protein in its main function (52). After modeling, the epitope under investigation from the protein was obtained, and the preparation was performed using the MGL tool, in which missing atoms were corrected and water molecules were removed (53). The same preparation was performed for MHCII molecules found on the PDB server (Protein Data Bank) (54). For docking execution, both files were converted to the PDBQT format required by AutoDock Vina (55), using the Openbabel tool (56). The gridbox was generated around the active sites of the recovered MHCII proteins. To visualize the interactions between epitope-MHCII in 2D and 3D diagram format, the Ligplot+, and Pymol tools were used, respectively (57, 58).

### Mice Experiments

**Figure 2:**
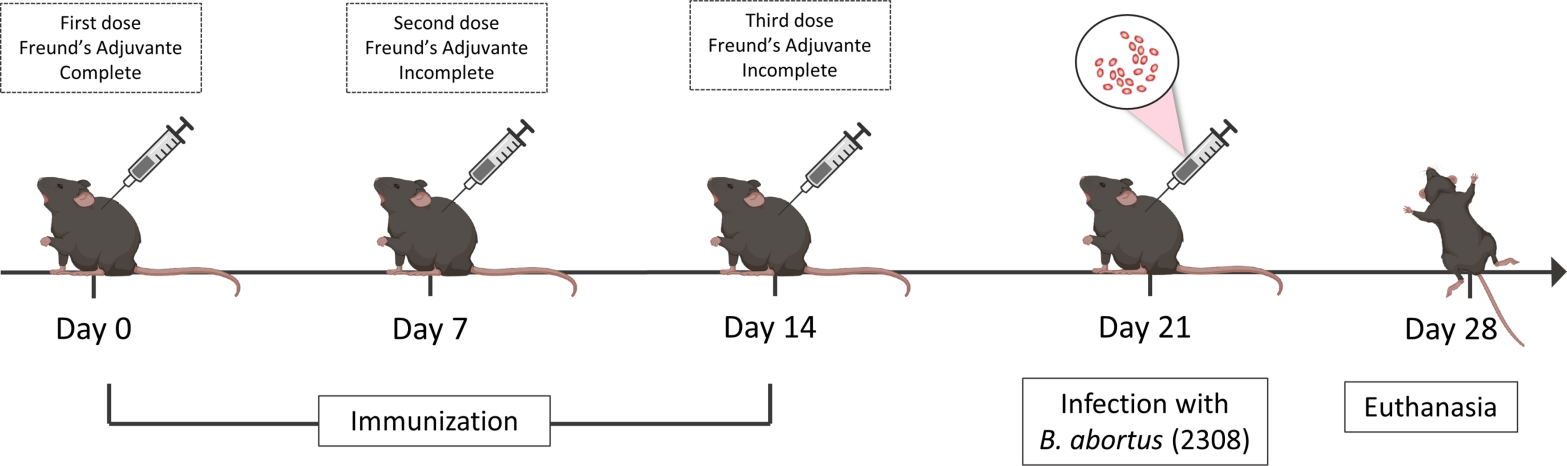
Experimental design of *in vivo* analysis. C57BL/6 mice were vaccinated three times with intervals of seven days between doses, the first dose being in the presence of complete Freund’s adjuvant and the other doses using the same incomplete adjuvant. Seven days after the last immunization the mice were challenged with the virulent strain 2308 of *B. abortus* and on day twenty-eight the animals were euthanized to obtain a spleen, liver and axillary lymph node.

To assess the ability of vaccinal peptide to induce immune responses, 6–8-week-old C57BL/6 mice were contained in cages on a 12:12 light/dark cycle and fed *ad libitum* with standard rodent diet and no water restrictions. Mice were randomly separated into four treatment groups (*n* = 5): (I) vaccinated and infected with *B. abortus*, (II) vaccinated and non-infected with *B. abortus*, (III) non-vaccinated and infected with *B. abortus*, (IV) non-vaccinated and non-infected with *B. abortus* and (V) Adjuvant and infected with *B. abortus*. Mice were immunized intraperitoneally (IP) with a prime and two boosts (0, 7, 14 days) of vaccine formulation containing 10 μg of peptide in phosphate-buffered saline (PBS), combined with complete Freund’s adjuvant (Sigma-Aldrich, St. Louis, MO, USA) at day 0 and incomplete Freund’s adjuvant at days 7 and 14. Group V (adjuvant and infected) followed the same protocol receiving IP phosphate-buffered saline (PBS) combined with Freund’s adjuvant (complete and incomplete). On day 21, mice were infected intraperitoneally with *B. abortus* S2308 virulent strain at a dose of 1x10^6^ CFU/animal in 100 μL PBS. Bacterial loads in the spleen, liver, and axillary lymph node from individual animals were homogenized in PBS, serially diluted 10-fold, and plated on *Brucella* broth agar (Difco, BD-Pharmingen, San Diego, CA). Plates were incubated at 37°C, and the CFU were counted after 3 days as previously described (59). The experimental design is represented in **Figure 2**.

### Antibody Measurement From Serum Samples by ELISA

To detect the immunogenicity of apolipoprotein N-acyltransferase epitope, antigen-specific IgG, IgG1 and IgG2c in the immunized mice were tested by ELISA. Briefly, the immunoassay plates (Maxisorp, Nunc, Denmark) were coated with apolipoprotein N-acyltransferase epitope (1 μg/mL) diluted in 0.1 M bicarbonate buffer (pH 9.0) and incubated at 4°C overnight. The wells were washed three times with phosphate-buffered saline-Tween20 (PBS-T) and then blocked with PBS containing 5% skimmed milk at 37°C for 1h. Vaccinated and non-vaccinated mice serum (1:10) was added to the well and incubated at room temperature for 2h. After adequate washing with PBS-T, plates were incubated with peroxidase-conjugated anti-mouse IgG (1:2500), IgG1 (1:5000), and IgG2c (1:20000) (Sigma Chemical Co., USA) for 1h at 37°C. After washing with PBS-T, substrate solution (0,5 mM TMB, 20 mM H2O2 no buffer PBS, pH 5) was added to each well, and plates were incubated in the dark at room temperature. Color development was stopped by adding 50 μL of 2 M H_2_SO_4_ after 10 min. The absorbance value was measured using a microplate reader (Bio-Tropsch Tek Instruments, Winooski, Vt., USA) at a wavelength of 450 nm. To confirm antigen-specificity, the serum from vaccinated and non-vaccinated mice was also used in immunoassay plates coated with the unrelated antigen lysozyme from hen egg white (1 μg/mL) (Sigma Aldrich) following the same parameters described above.

### Histopathology and Immunohistochemistry Assays

The medial lobes of the mice liver were collected, fixed in 10% buffered formaldehyde solution, dehydrated, diaphanized, and embedded in paraffin. Four-micrometer-thick tissue sections were stained with hematoxylin and eosin (H&E). Digital images were captured and digitized with the AxioVision LE software (Carl Zeiss, Oberkochen, Germany); all of the sample fields were photographed for histopathological evaluation. The histopathological changes were analyzed by Image Pro-PlusR 4.5 software (Media Cybernetics Inc., Silver Spring, MD, USA). The total number and size of granulomas present in histological liver sections was determined using an Axiophot microscope (Carl Zeiss, Oberkochen, Germany) with a 40x objective lens. Immunohistochemistry was performed as previously described (60). Briefly, liver sections were hydrated and incubated with 10% hydrogen peroxide in PBS for 30 min. After being washed with PBS, slides were transferred to a humid chamber at room temperature, incubated with 25 mg/ml of skim milk for 45 min, and then incubated with a primary antibody for 30 min. For immunolabeling, diluted (1:5,000) serum from a rabbit experimentally inoculated with *B. abortus* S19 strain was used as polyclonal anti-*B. abortus* antibody. Then, tissue sections were washed with PBS, incubated with secondary antibody for 20 min, washed again with PBS, and incubated for 20 min with streptavidin-peroxidase from a commercial kit (LSAB + kit; Dako Corporation, Carpinteria, CA). The reaction was revealed using 0.024% diaminobenzidine (DAB; Sigma), and sections were counterstained with Mayer’s hematoxylin.

### Measurement of NO and IFN-γ Into Splenocyte Culture Supernatants

Spleens cells from C57BL/6 mice under treatment obtained after maceration were treated with ACK buffer (0.15 M NH4Cl, 1.0 mM KHCO3, 0.1 mM Na2EDTA, pH 7.2) to lyse red blood cells. After that, the cells were washed with saline (NaCl 0.8%, wt/vol) and suspended in RPMI 1640 (Gibco, Carlsbad, Calif) supplemented with 2 mM L-Glutamine, 25 mM HEPES, 10% (vol/vol) heat-inactivated FBS (Gibco, Carlsbad, Calif), penicillin G sodium (100 U/mL), and streptomycin sulfate (100 μg/mL). Spleen cells (1x10^6^) were cultured in 200µL culture medium and incubated at 37°C with 5% CO_2_. The supernatant of splenocyte cultures was collected after 24h and nitric oxide (NO) measurement was performed according to the Griess method (61). The levels of IFN-γ secreted were quantified from the supernatant of splenocytes cultures by antigen-capture ELISA. For this, 1x10^6^ splenocytes (per well) were cultured in 200 μL of culture medium in the absence and presence of 1ug of the peptide as a stimulus for 72h at 37°C with 5% CO_2_. Aliquots from each well were then taken and IFN-γ levels were measured using the Murine IFN-γ Mini ABTS ELISA Development Kit (PeproTech Inc) following the manufacturer’s instructions. Final cytokine concentrations were calculated using the standard curve for IFN-γ. The final reaction was measured using a microplate reader (Bio-Tropsch Tek Instruments, Winooski, Vt., USA) and read at 405 nm with wavelength correction set at 650 nm.

### Analysis of Surface Markers CD86 and CD11b by Fluorescence Microscopic

BMDMs (1x10^6^ cells per well) were plated on imaging slides (µ-Slide 12-well, glass bottom, Ibidi GmbH, Munich, Germany), followed by stimulation with splenocytes supernatant for 48h. The cells were then washed three times with PBS and incubated with the anti-CD16/32 antibody (BD Biosciences, San Jose, CA) for 2 hours to block nonspecific bonds. The cells were then incubated with anti-CD86 and anti-CD11b, followed by staining with FITC-conjugated and PE-conjugated (BD Biosciences), respectively, overnight at 4°C. The slides were washed with PBS and the nuclei were stained with 150 ng/mL 40,6-diamino-2-phenylindole (DAPI; Thermo Scientific) for 1 hour. All images were captured using a Nikon Eclipse 80i fluorescence microscope (Melville, New York, U.S.A). Image J software was used to analyze the markings obtained for the nucleus (blue fluorescence), CD11b+ cells (green fluorescence), and CD86+ cells (red fluorescence).

### Real-Time RT-PCR for Pro and Anti-Inflammatory Cytokines Expression

Liver and spleen macerate as well as BMDMs stimulated with splenocytes supernatant from the four experimental groups were homogenized with TRIzol reagent (Invitrogen) to isolate total RNA. Reverse-transcription of 1 μg total RNA was performed using Illustra™ Ready-To-Go RT-PCR Beads (GE Healthcare, Buckinghamshire, UK). Real-time RT-PCR was conducted in a final volume of 10 μL containing the following: SYBR^®^ Green PCR Master Mix (Applied Biosystems, Foster City, CA, USA), with cDNA as the PCR template and primers to amplify specific fragments corresponding to specific gene targets: TNF-α F: 5’-CATCTTCTCAAAATTCGAGTGACA-3’, TNF-α R: 5’-TGGGAGTAGACAAGGTACAACCC-3’; IFN-γ F: 5’-TCTGGAGGAACTGGCAAAG-3’, IFN-γ R: 5’-TTCAGACTTCAAAGAGTCTGAGG-3’; IL-6 F: 5’-CCAGGTAGCTATGGTACTCCAGAA-3’, IL-6 R: 5’-GATGGATGCTACCAAACTGGA-3’; IL-10 F: 5’-GGTTGCCAAGCCTTATCGGA-3’, IL-10 R: 5’-ACCTGCTCCACTGCCTTGCT-3’; TGF-β F: 5’-TGACGTCACTGGAGTTGTACGG-3’, TGF-β R: 5’-GGTTCATGTCATGGATGGTGC-3’; iNOS F: 5’-CAGCTGGGCTGTACAAACCTT-3’, iNOS R: 5’-CATTGGAAGTGAAGCGTTTCG-3’. The PCR reaction was performed with ABI 7500 Real-Time PCR System (Applied Biosystems, Foster City, CA), using the following cycling parameters: 60°C for 10 min, 95°C for 10 min, 40 cycles of 95°C for 15 sec and 60°C for 1 min, and a dissociation stage of 95°C for 15 sec, 60°C for 1 min, 95°C for 15 sec, 60°C for 15 sec. All data are presented as relative expression units after normalization to the β-actin gene (F: 5’-AGGTGTGCACTTTTTATTGGTCTCAA-3’, R: 5’-TGTATGAAGGTTTGGTCTCCCT-3’). PCR measurements were conducted in triplicate. The differences in the relative expression were analyzed by analysis of variance (ANOVA) followed by Tukey’s test (p < 0.05 denotes statistical significance).

### Statistical Analysis

Graphs were created and data analysis was performed using GraphPad Prism 8 software (San Diego, CA, USA), using one-way ANOVA or two-way ANOVA (Bonferroni *post hoc* test). Values <0.05 were considered statistically significant.

## Results

### High-Throughput Sequencing Identifies the Expression of Small RNAs of *B. abortus* During Infection in Macrophages

In the small RNA libraries from infected macrophages, we observed that 7.26% of all mapped sequences belonged to the *B. abortus* genome (**Table 1**). Knowing this, using the Bowtie software, it was seen that the small RNAs were found to be distributed in both chromosomes of the bacteria. By analyzing the depth of coverage of the sequences, we identified a total of 3954 regions of broad mapping of small RNAs in the genome of *B. abortus*, 2694 in chromosome I and 1260 in chromosome II. However, in local data of the bacterial genome, the presence of three peaks (hotspots) was observed, with two in chromosome I and one in chromosome II, constituted by small RNAs being mapped in the same position of the genome and large quantities (**Supplementary Figure 1**). With that, these three hotspots were selected, and we next concentrated our analysis on selecting the small RNAs that were mapped multiple times in these hotspots (**Supplementary Figure 2**). In total, we identified one hundred small RNAs with this characteristic which were then submitted to further analysis to detect the target mRNA in NCBI.

**Table 1:** Data obtained after mapping of reads.

	*B. abortus* infected	%	Control noninfectedBMDMs	%
	BMDMs			
Processed Reads	35.713.113		46.516.010	
Reads mapped in the *B. abortus* (S2308) genome	2.593.062	7.26%	17.269	0,04% of the total
Reads mapped in the *Mus musculus* (GRCm38) genome	33.120.051	92.74%	46.498.741	99,96% of mapped reads

### Apolipoprotein N-Acyltransferase (BAB1_2158) Is a Putative Target of *B. abortus* sRNAs and Its Expression Is Diminished During Earlier Time of BMDM Infection

After the sequences were obtained from NCBI, the surface-associated proteins were selected by our SCL prediction tools. In this phase, 10 proteins were included, making up the final list of surface-associated proteins. The exposure of extracellular structures is an attraction for the immune system in the recognition of antigens and, therefore, the topology predictive tools selected seven proteins composed of extracellular domain amino acids. All selected extracellular portions were composed of at least 35 amino acids. To gain more insight into the biological functions of these 7 proteins, an analysis was performed to identify biological patterns associated with antigens. Of the 7 proteins analyzed, all were inferred by homology in UniProtKB, and a few are those reviewed regarding their biological function (**Table 2**). The information obtained by the Gene Ontology (GO) project showed that only two of these proteins would be involved in the biosynthesis process of the bacterial structure, yet one of them had a smaller number of exposed amino acids. The other, in addition to exposing 277 amino acids in the extracellular portion, also participates in the bacterial lipopolysaccharide biosynthesis process, becoming the target protein for further analyses. The other proteins analyzed had a transmembrane transport function, in addition to mediating cellular vesicle fusion processes.

**Table 2:** List of proteins predicted to have transmembrane helices and respective biological functions.

Protein ID (NCBI)	Length (aa)	Single-line annotation (NCBI)	Biological function (UniProtKB)	Gene
WP_002968965.1	386	Lipase	Pathogenesis and negative regulation of endosome organization, and vesicle fusion	BAB2_0654
WP_002964284.1	270	Phosphatidate cytidylyltransferase	CDP-diacylglycerol biosynthetic process	BAB1_1179
WP_002965220.1	532	Apolipoprotein N-acyltransferase	Lipoprotein biosynthetic process	BAB1_2158
WP_002971227.1	430	Xanthine/uracil/vitamin C permease family	Transmembrane transporter activity	BAB2_0578
WP_002971267.1	510	Amino acid permease	Transmembrane transporter activity	BAB2_0864
WP_002966986.1	412	MFS transporter superfamily	Transmembrane transporter activity (carbohydrate)	BAB1_1882
WP_002964796.1	400	OPGC	Transmembrane transporter activity (sugar)	BAB1_1718

**Figure 3:**
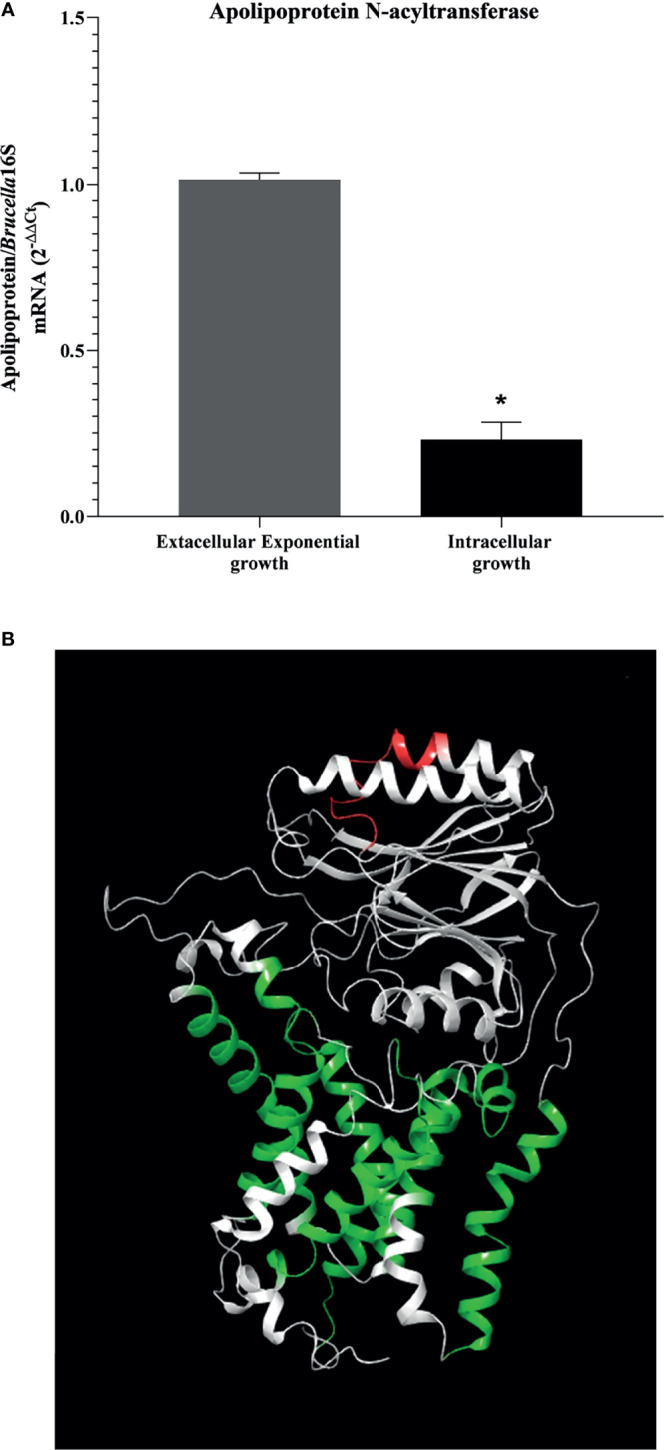
Analysis of apolipoprotein N-acyltransferase target protein. **(A)** Decreased in the gene expression of apolipoprotein N-acyltransferase is shown in an intracellular-growing model. Differential expression analysis of apolipoprotein N-acyltransferase in model of extracellular and intracellular growth was measured by real-time RT-PCR and **(B)** position identification of selected epitope in 3D molecular modeled apolipoprotein N-acyltransferase by homology. Protein is represented in white; the selected epitope is highlighted in red, and the transmembrane helices are indicated in the models in green. Statistically significant differences relative to the extracellular exponential growth are represented by an asterisk (*P <0.05).

Therefore, apolipoprotein N-acyltransferase (BAB1_2158) was selected for the further analysis of differential expression during BMDM infection. Knowing that small RNAs were expressed in large quantities by the bacterium during intracellular infection by *Brucella*, we investigated the levels of mRNA expression of Int in samples of intracellular and extracellular growth in a model of BMDM infection with *B. abortus*. The result of differential expression analysis showed that during BMDM infection, the bacteria decreased the Int gene expression when compared to an exponential extracellular growth model (**Figure 3A**). Taking into account the results of the *in silico* analysis, the negative expression of the Int coding gene may be related to an initial post-transcriptional gene regulation process used by the bacteria to repress the expression of this protein during intracellular infection, thus establishing its replicative niche in the host cell. After reaching the replicative niche, *B. abortus* may increase the Int coding gene since it plays a crucial role in bacterial LPS biosynthesis during replication.

Since the results indicate a possible mechanism of gene expression control by the bacterium in stressful intracellular environments, we were searching for exposed immunogenic epitopes in the extracellular amino acid sequence of Int. The results obtained indicated approximately 60 epitopes that showed a strong binding affinity for HLA molecules (HLA-DRB1 0101, HLA-DRB1 0301, HLA-DRB1 0401, HLA-DRB1 0701, HLA-DRB1 1101, and HLA-DRB1 1501). In this stage, the predictor tools selected epitopes composed of exactly 15 amino acids. When subjected to alignment with the extracellular portion of the protein, the most promiscuous epitopes formed a conserved region; from this region, the epitope “AIPYILESTPQALAH” was selected. To identify the position of the selected putative immunogenic epitope in the Int, we performed molecular modeling, showing the selected epitope highlighted in red with transmembrane helices indicated in the model in green, as indicated in **Figure 3B**. When submitted to in silico screening tests, the epitope did not show the desired antigenicity (0.2800), however showed 100% similarity with sequences present only in the apolipoprotein N-acyltransferase of *B. abortus*. To increase the confidence of using the selected epitope in future *in vitro* and *in vivo* evaluations, allergenicity was tested using the AlgPred software, characterizing it as non-allergenic. Although when analyzing the physicochemical properties, the epitope was shown to be unstable (40.27), since it did not reach the minimum cut-off value for stability, it had a relatively good half-life in mammalian cells (Half-life reticulocytes: 4.4h; Half-life yeast: >20h; Half-life E. coli: >10h), that is, despite its instability in the medium, it is suggestive that when it binds to the MHCII cleft, it can present the desired stability.

### Molecular Docking Between the Selected Putative Immunogenic Epitope From Int and MHC-II Shows High Probabilities of Interaction Between Them

**Figure 4:**
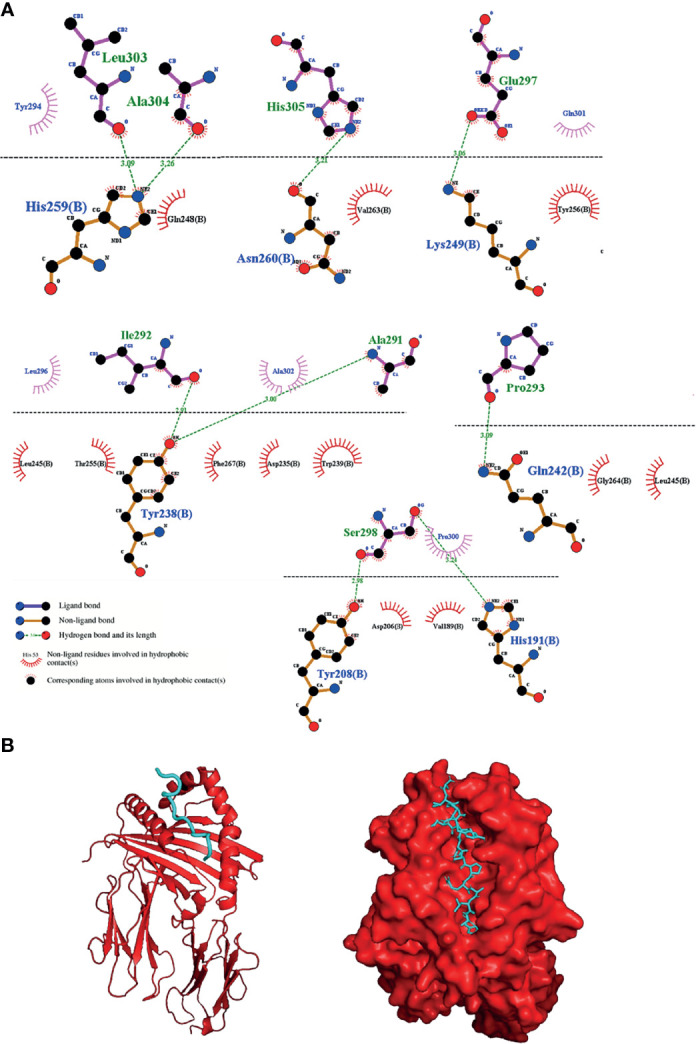
Interaction between the selected epitope from apolipoprotein N-acyltransferase and MHC-II allele. **(A)** 2D diagram of the best interaction between the HLA-DRB1:0101 allele and the epitope, representing above the dotted line epitope amino acids and below line MHCII amino acids and **(B)** 3D diagram of the interactions.

Although not all structures of MHC-II were found in the database (IEDB), we used the structure of 3 available proteins: HLA-DRB1:0101 (PDB: 5NI9), HLA-DRB1:0401 (PDB: 5V4M) and HLA-DRB1:1501 (PDB: 5V4N). When performing the molecular docking of the “AIPYILESTPQALAH” epitope, the results showed great interaction energy of all alleles based on the AutoDock Vina software (**Table 3**). To represent the molecular docking between the epitope and the MHC-II allele, we chose the interaction with the highest interaction power pointed out by the software. The epitope in question interacted very well with the MHCII of the HLA-DRB1:0101 allele, having the best interaction values, with an energy of -8.1 Kcal.mol^-1^ and presenting a total of 9 hydrogen bonds between the epitope and the MHC, with two bonds involving the amino acid HIS259 of the MHC with the amino acids LEU303 at a distance of 3.09Å and with the amino acid ALA304 at a distance of 3.26Å from the epitope. One link of amino acid ASN260 of MHC with amino acid HIS305 was 3.21Å from the epitope, one link of amino acid GLN242 of MHC with amino acid PRO293 showed a distance of 3.09Å from the epitope, two linkages involving amino acid TYR238 of MHC with the amino acid ILE292 was 2.91Å away and the link with the amino acid ALA291 was at a distance of 3.00Å from the epitope; also, a link of the amino acid LYS249 of the MHC with the amino acid GLU297 was at a distance of 3.06Å and finally two bonds involving amino acids TYR208 and HIS191 of the MHC, showed distances of 2.90Å and 3.24Å, respectively, with amino acid SER298 of the epitope (**Figures 4A, B**). In summary, the peptide was shown to have a great ability to interact with MHC-II slits, especially with the HLA-DRB1:0101 allele, showing excellent interaction with 9 of the 15 amino acids of the epitope. Although minor binding strengths have been identified, this does not preclude the possibility of interaction between peptide-alleles.

**Table 3:** Molecular docking results according to AutoDock Vina software.

MHC-II	Connection Power (Kcal.mol-1)	Hbonds	Non-ligand residues involved in hydrophobic contacts MHC	Non-ligand residues involved in hydrophobic contacts epitope
5NI9	-8,1	9	11	5
5V4M	-6,6	5	8	6
5V4N	-7,1	7	10	3

### Immunization of Mice With Epitope-Based Vaccine Induces Protection Against *B. abortus* Infection and Specific Humoral and Cell Immune Responses Against the Epitope From Int

**Figure 5:**
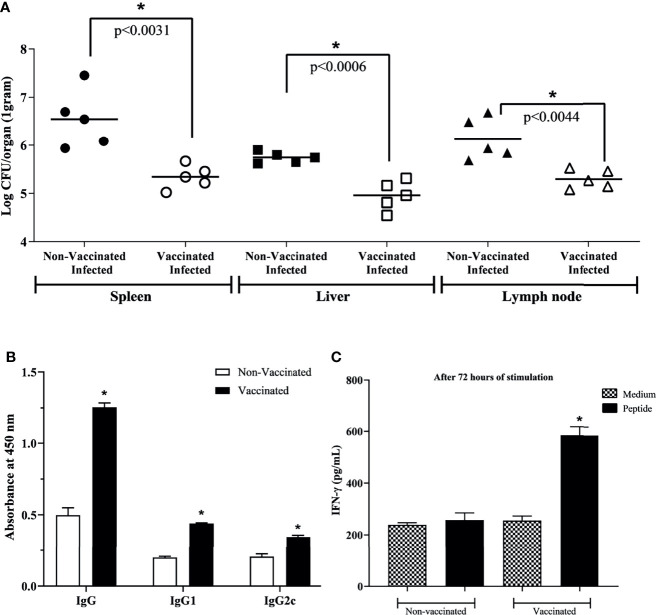
Protective efficacy and specificity after three rounds of immunization. **(A)** For protection evaluation, vaccinated mice were challenged with 1 × 10^6^ CFU/bacteria. One-week post-challenge, organs were collected to determine bacterial loads to assess protection efficacy. A smaller number of viable bacteria was identified in vaccinated mice, indicating great protection efficacy. **(B)** Specific IgG, IgG1, and IgG2c antibodies in the immunized and non-vaccinated mice serum. **(C)** IFN-γ production by vaccinated or non-vaccinated mice splenocytes stimulated or non-stimulated with selected peptide. Data points were individual values of CFU determinations (n = 5) and analyzed using a student’s t test. *P < 0.05 relative to the non-vaccinated and infected group.

After immunization, vaccinated and unvaccinated mice were challenged by intraperitoneal infection with *B. abortus*; after seven days, organs were collected to determine bacterial loads. In the spleen, liver and axillary lymph node of mice vaccinated with the peptide, fewer viable bacteria were recovered when compared to the control group (**Figure 5A**).

To evaluate the potential use of the epitope-based vaccine, the protection level induced in mice against virulent challenge infection was assessed. The degree of vaccine efficacy in C57BL/6 mice was determined by subtracting the mean CFU/organ recovered from mice after vaccination and challenged from the mean CFU/organ recovered from non-vaccinated but challenged control mice. At this time, it was seen that the presence of vaccinal peptide in the animal organism triggered a higher degree of protection against infection, approximately 1.20/0.80/0.84-log in spleen, liver, and axillary lymph node, respectively (**Table 4**). Finally, aiming to exclude the bias of adjuvant composing the vaccine solution, the group that received only adjuvant was evaluated. The result showed 6.12 ± 0.36, 5.73 ± 0.09, and 5.73 ± 0.12 for spleen, liver, and lymph node, respectively, where there was no statistically significant difference compared to the unvaccinated group. Ours results showed that the RV-selected epitope provided significant protection to C57BL/6 mice against *B. abortus*.

**Table 4:** Protective efficacy conferred by vaccinal peptide against *B. abortus* infection.

Group (n = 5)	Log_10_ CFU (Spleen)^a^	Log Protection	Log_10_ CFU (Liver)	Log Protection	Log_10_ CFU (Lymph node)	Log Protection
Vaccinated	5.34 ± 0.28*	1.20	4.96 ± 0.34*	0.80	5.30 ± 0.19*	0.84
Non-vaccinated	6.54 ± 0.63	–	5.74 ± 0.13	–	6.13 ± 0.43	–

Protection units of vaccinated group are compared with that of non-vaccinated with Student’s t-test, *P < 0.05 is statistically significant.

a CFU, colony-forming units.

To verify the peptide potential to induce a specific humoral immune response in immunized mice, the levels of specific IgG, IgG1, and IgG2c antibodies were measured by ELISA in mice serum. The immunoglobulins in immunized mice with peptide vaccine were significantly higher than those of the non-vaccinated group (**Figure 5B**). It was not observed reaction to unrelated antigen lysozyme from hen egg white. In addition, the amount of IFN-γ showed significantly increased in splenocytes from vaccinated mice stimulated with peptide when compared to splenocytes from non-vaccinated mice, or when compared to non-stimulated cells (**Figure 5C**), indicating the specificity of the immune response of immunized mice not only in humoral but also in cellular response.

### Unvaccinated Mice Showed Greater Liver Damage When Infected With *B. abortus*

**Figure 6:**
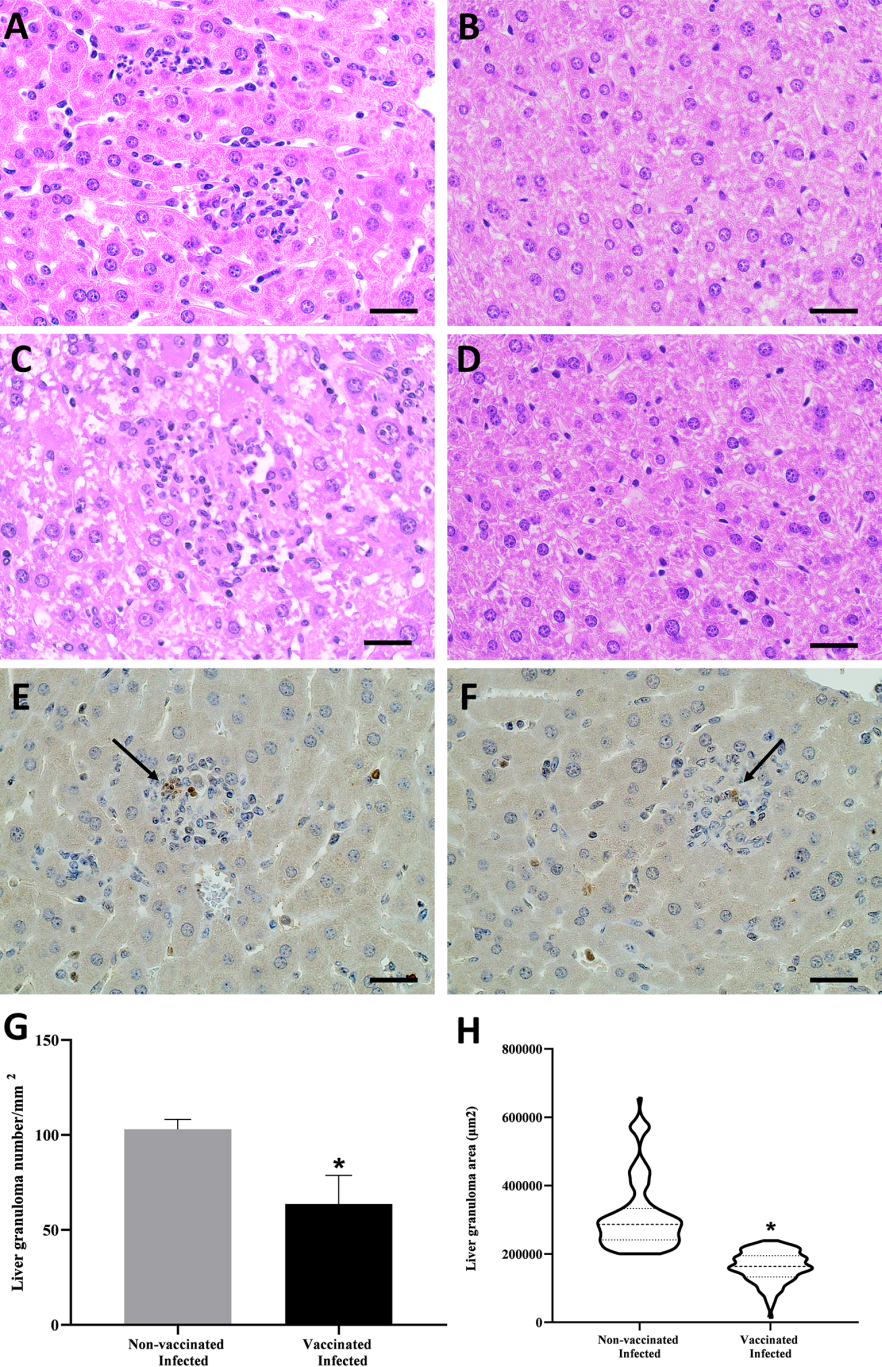
Histopathology analysis, immunohistochemistry, and morphometric of hepatic tissue of *B*. *abortus* infected C57BL/6 mice. **(A)** Representative of hematoxylin- and-eosin-stained sections of hepatic tissue from mice vaccinated and infected, **(B)** vaccinated and non-infected, **(B)** unvaccinated and infected, and **(D)** non-vaccinated and non-infected. Immunohistochemistry sections of hepatic tissue from mice vaccinated and infected **(E)** and non-vaccinated and infected **(F)** mice containing the *B*. *abortus* inside the granuloma. The graphs analyze the granulomas of liver tissue sessions that were sequentially captured in terms of number **(G)** and area **(H)**. Statistically significant differences relative to the non-vaccinated group are represented by an asterisk (*P <0.05). The arrows indicate the *B*. *abortus* within the granuloma. Scale bars: 50 µm.

*B. abortus* infection is associated with the formation of focal granulomatous lesions in the spleen, liver, and lymphoid tissues of both humans and rodents, starting 1–2 weeks post-infection (62). To determine the characteristics of liver pathology upon vaccination with peptide during *B. abortus* infection, we performed the histopathological analysis of liver tissue from vaccinated and unvaccinated C57BL/6 mice and, challenged with *B. abortus*. Infection with *B. abortus* resulted in the formation of hepatic granulomas in both groups (**Figures 6A, C**). In morphometric analysis, a greater number of granulomas was observed in the tissue of animals that were not vaccinated with the peptide and challenged by bacteria compared to the vaccinated group (**Figure 6G**). The same result was seen when analyzing the area, which was greater in granulomas from unvaccinated animals (**Figure 6H**). Histopathological lesions during *Brucella* infection usually are associated with the bacterial load. To determine the relationship between granuloma formation and the bacteria present in the granuloma, we performed immunohistochemistry to immunolabel *B. abortus*. **Figures 6E, F** showed the presence of *B. abortus* in the granulomatous lesions presented in the liver of vaccinated and infected mice and non-vaccinated and infected mice, respectively. The detection of intralesional bacteria confirms that the inflammatory lesions described in this study are due to systemic *B. abortus* infection. No observable lesions were found in tissues from uninfected mice (**Figures 6B, D**).

### Vaccination Induces Positive Expression of Pro- and Anti-Inflammatory Cytokines in Animals Infected With *B. abortus*

**Figure 7:**
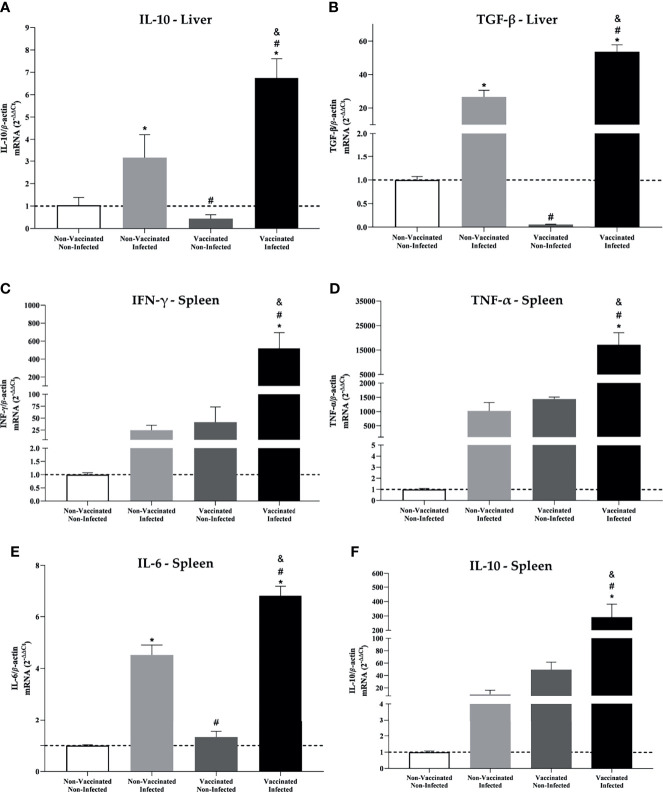
Liver and spleen showed increased expression of pro and anti-inflammatory cytokine in mice vaccinated and infected with *B*. *abortus*. Differential expression analysis of **(A)** IL-10 and **(B)** TGF-β from the liver tissue and **(C)** IFN-γ, **(D)** TNF-α, **(E)** IL-6, and **(F)** IL-10 from the spleen of C57BL/6 mice from the four experimental groups evaluated in this study. Transcript levels were measured by real-time RT-PCR. Error bars represent the mean ± SD of samples assayed in triplicate. *P < 0.05 relative to the non-vaccinated and non-infected group. ^#^ *P* < 0.05 relative to the non-vaccinated and infected group. ^&^ *P <*0.05 relative to the vaccinated and non-infected group.

The liver is the most commonly affected organ in patients with active brucellosis (63), which is why liver macerates from the four experimental groups were collected for evaluation of the expression of the anti-inflammatory genes *IL-10* and *TGF-β*. In the differential expression analysis, an increase in these cytokines was observed in vaccinated and infected C57BL/6 mice when compared to the other groups (**Figures 7A, B**). Therefore, the reduction in liver pathology can be attributed to a decrease in the number of viable bacteria and an increase in anti-inflammatory cytokines, resulting in a consequent reduction in liver damage. Concomitantly, the splenic tissue was evaluated and the results obtained showed that all groups, except for the control, showed upregulation of the proinflammatory cytokine-coding genes *INF-γ*, *TNF-α*, and *IL-6*, which are characteristic of inflammation (**Figures 7C–E**), but the vaccinated and infected group stood out due to the increased expression when compared to other groups. Likewise, it was seen that IL-10 (**Figure 7E**) is up-regulated in this group, suggesting that vaccination induces an attempt to control the inflammatory process generated by systemic infection with *B. abortus*.

### BMDM’s From C57BL/6 Mice Stimulated With Supernatant Splenocytes From the Vaccinated and Infected Group Showed Higher Expression of CD86

**Figure 8:**
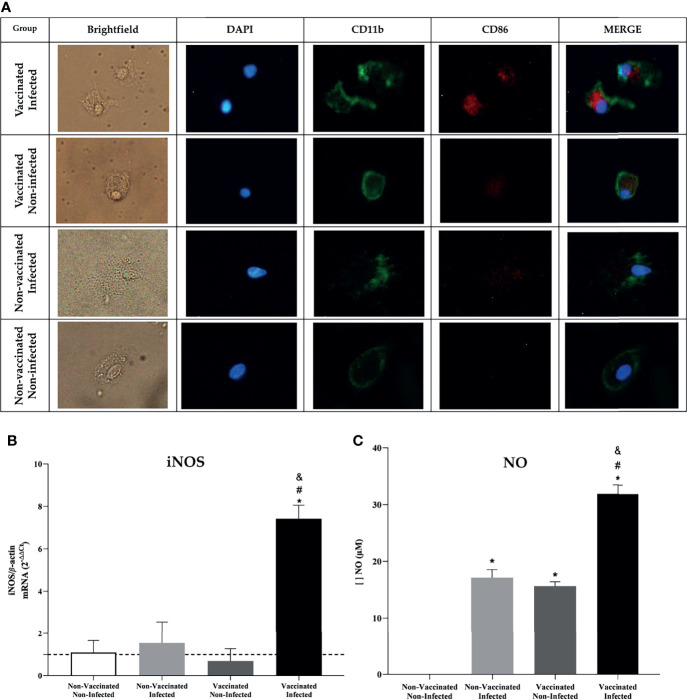
*In-vitro* analysis showed an increased phagocytic and microbicide capacity in BMDM’s. **(A)** Fluorescence microscopy shows activation of BMDM’s by the CD86 molecule after being stimulated with supernatant from the spleen of C57BL/6 animals. **(B)** Differential expression analysis of iNOS in stimulated BMDM’s was measured by real-time RT-PCR and **(C)** NO dosage using Griess method. Error bars represent the mean ± SD of samples assayed in triplicate. *P < 0.05 relative to the non-vaccinated and non-infected group. ^#^ *P* < 0.05 relative to the non-vaccinated and infected group. ^&^ *P <*0.05 relative to the vaccinated and non-infected group. Scale bars: 20 µm.

*In vivo* analysis results indicate the activation of an adaptive immune response. Knowing this, we tried to understand the mechanisms by which a more efficient immune response activation process against the bacteria occurs. BMDMs were evaluated for the expression of co-stimulatory molecules after being stimulated with the splenocyte supernatant. The results obtained by fluorescence microscopy show that BMDMs stimulated with the supernatant from the spleen of vaccinated and infected animals were more activated, due to intense CD86 labeling (**Figure 8A**), and that they even expressed a higher level of *iNOS* expression, the gene that stimulates NO production (**Figure 8B**). To confirm this result, the measurement of NO was performed in the in the supernatant of these macrophages. The results obtained showed the greater production of NO by BMDMs that were more activated (**Figure 8C**), which may reveal an increased phagocytic and microbicide capacity to eliminate the bacteria.

## Discussion

The development of subunit vaccines to protect against brucellosis is crucial to avoid the disadvantages of the used live attenuated vaccines RB51 and S19 against *B. abortus* (64, 65). New vaccines will be designed according to immune responses during a natural infection in animal models and the identification of intracellular and cell surface immunodominant components of *Brucella* spp (66–69). Using RV, it has been shown that the genome of *B. abortus* contains approximately 80 genes encoding putative lipoproteins that have diverse functionalities, including pathogenic processes (70, 71). Knowing this, recent studies have shown that some bacterial cell surface proteins can provide significant protection against *Brucella*, such as L7/L12 (72), Omp19, Omp31 (73), BP26 (74), and Omp25 (75, 76), which have been shown to be immunodominant antigens that stimulate host immunity and trigger a protective response against infection in a mouse model. Bacteria of the genus *Brucella* can live, replicate and persist within phagocytes using their mechanisms to evade the immune system and establish their replicative niche within the host cell (77). In this context, previous studies suggest that small RNAs may be directly related to the timely gene expression of virulence factors in a variety of pathogenic bacteria such as *Listeria* and *Yersinia* (78, 79). Considering that the expression of small bacterial RNAs allows changes in the host cell phenotype, and knowing that these small RNAs act on gene activation and repression, we evaluated the capacity that a rationally predicted epitope of Int, a target protein of small RNAs expressed on large scale by the bacteria during macrophage infection, would have to activate protective immune responses during infection by *B. abortus* in a murine model.

Since *Brucella* species are equipped with a variety of well-organized immune evasion strategies to establish chronic infections, including the use of small non-coding RNAs (80, 81), we performed a detailed analysis from data from the sequencing of small RNAs expressed during the infection of BMDMs with *B. abortus*, showing that 7.26% of the small RNAs were mapped in the bacterial genome. This was consistent with previous reports that identified expression levels of similar small *B. abortus* RNAs during the infection of murine macrophages (82). Casewell et al. reported that *B. abortus* small RNAs, abcR1 and abcR2 play essential roles in pathogenicity and chronic infection, resulting in a significant decrease in intracellular survival in a mouse model and in macrophages (83). Another group identified 129 small RNAs of *Brucella* that play significant roles in diverse biological processes, ranging from physiology to virulence, as well as in host-pathogen interaction (84). These reports shed light on the importance of small non-coding RNAs in *Brucella* immunity, pathogenesis, and intracellular survival, modulating the host’s immune response. Here, we selected a non-allergenic with a good half-life in mammals, yeast, or *E. coli*, from a putative target of a more highly expressed *Brucella* sRNA. Rationally, we also take account of the structural and functional aspects of this target meeting the epitope “AIPYILESTPQALAH” of apolipoprotein N-acyltransferase (Int), even it presented low immunogenicity *in silico*. Although other proteins were predicted as possible targets of *Brucella* sRNA in our analyses, this one, in particular, stood out due to its biological function and strong epitope MHCII-interact capacity. Reportedly, Int functionally constitutes *Brucella*’s outer membrane and plays a crucial role in bacterial LPS biosynthesis (70, 71, 85). Interestingly, one of the main virulence factors of *Brucella* identified so far is its non-canonical LPS (86, 87) which exhibits favorable properties for the bacterium, including low endotoxicity, high resistance to degradation in macrophages, and protection against immune responses (88–90). The differential expression analysis of this protein-coding gene was performed in intracellular growth samples in a BMDM model infected with *B. abortus*, showing a drastic reduction in expression, corroborating our hypothesis that there is a post-transcriptional gene regulation process used by the bacterium to repress the expression of Int during the early time of infection. This regulation can favor the bacteria permanence and replication in the host cell and, after reaching its niche replication, increase the expression of genes related to LPS biosynthesis as apolipoprotein N-acyltransferase. Therefore, in this study, in an unprecedented way, we evaluated the capacity of a specific Int epitope selected by RV to induce an immune response in a murine model infected with *B. abortus*. The candidate epitope-based vaccine was able to trigger protective immune responses, when the amount of viable *B. abortus* in the liver, spleen, and axillary lymph nodes of vaccinated and unvaccinated mice when challenged intraperitoneally by this pathogenic bacterium was evaluated. It was observed that immunization considerably decreased the recovery of *B. abortus* in the tissues evaluated and induced mean systemic protection of 0.94 logs when compared to unvaccinated animals. Other evaluated *Brucella* antigens behaved similarly; for example, the recombinant Omp16 and Omp19 and the encapsulated recombinant liposome Omp25 induced protection comparable to S19 in vaccinated mice after challenge (17, 18, 91). In addition, the Omp28 subunit vaccine increased resistance against the *B. abortus* challenge by inducing a CD4+ Th1 response, that protects against infection, but at a lower level than live attenuated vaccines (92). Other studies, analyzing the level of splenic CFU, showed levels of protection in animals immunized with S19 ranging from 1.4 to 2.9 log (93, 94). Corroborating our results, these findings in the literature reinforce good protection obtained in our study when compared to S19, which is a vaccine strain constituted by the bacterium in its entirety. We also found that the levels of IgG, IgG1and IgG2c in serum of vaccinated mice were higher than those in the non-vaccinated control group. Specific antibodies have been used as important indicators for evaluating vaccine candidates (95). In a particular study, rBLSOmp31 was synthesized and found the protein could induce IgG responses against *Brucella* in the immunized mice (96), as also demonstrated the humoral specificity in immunized mice with the *Brucella* Omp2b protein (97). In this study, we found that an epitope from apolipoprotein N-acyltransferase could also induce the humoral immune response in mice.

The characteristic pathological manifestation of *B. abortus* infection is granulomatous inflammation associated with bacterial load (98). In this study, we detected a significant reduction in the number and size of granulomas in the livers of animals vaccinated with the peptide compared to unvaccinated animals, suggesting that the vaccination induced an effective inflammatory immune response in this tissue. This condition is well defined, whereas, as infection in mice infected with *Brucella* progresses, the granulomas progressively decrease in size and number after 2–3 weeks of infection (62). In parallel, we saw that the reduction of pathology in vaccinated mice was accompanied by an increase in the expression of *IL-10* and *TGF-β* in the liver of infected animals. Although the impact of IL-10 on *Brucella* persistence and the establishment of chronic infection through macrophage modulation has been previously demonstrated using IL-10-deficient mice (99, 100), our findings provide evidence that the increased expression of *IL-10* is related to an immunoregulatory mechanism dampening excessive Th1 responses (101). In this context, it is noted that the absence of IL-10 results in severe pathological changes in different bacterial infections (102, 103). Here, we can speculate that the reduction in liver pathology in immunized and infected animals may have been mediated by an increase in anti-inflammatory cytokines, and attributed to a previous reduction in the number of viable bacteria.

Cell-mediated immunity is considered critical for the protective immune response against facultative intracellular pathogens (104, 105). Our results showed that the Int epitope induced the enhanced production of TNF-α and IFN-γ in spleen cells, suggesting that there is some induction of a Th1-type immune response by the vaccine peptide. In particular, IFN-γ is essential for immune protection against *Brucella* infection that induces more polarization toward Th1 cells (106, 107). In addition, functional TNF-α has been shown to link the proinflammatory response and adaptative immune response in *Brucella*-infected mice (108). High levels of IL-6 were produced by the splenocytes of immunized and infected mice. It was recently shown that IL-6 is required for the induction of IFN-γ and TNF-α by infected splenocytes, in addition to promoting the differentiation of CD8+ T cells, indicating a protective role for IL-6 against *B. abortus* that parallels the type of Th1 immunity response (109, 110). Similarly, significant levels of IL-10 in immunized mice were also detected in spleen cells. These results are consistent with the scenario seen in liver tissue, suggesting that an inflammatory immune response has already occurred to the point of generating an anti-inflammatory response with infection control characteristics. In this context, the balance between the production of pro- and anti-inflammatory cytokines appears to be crucial for the host’s ability to eradicate the infection (100).

**Figure 9:**
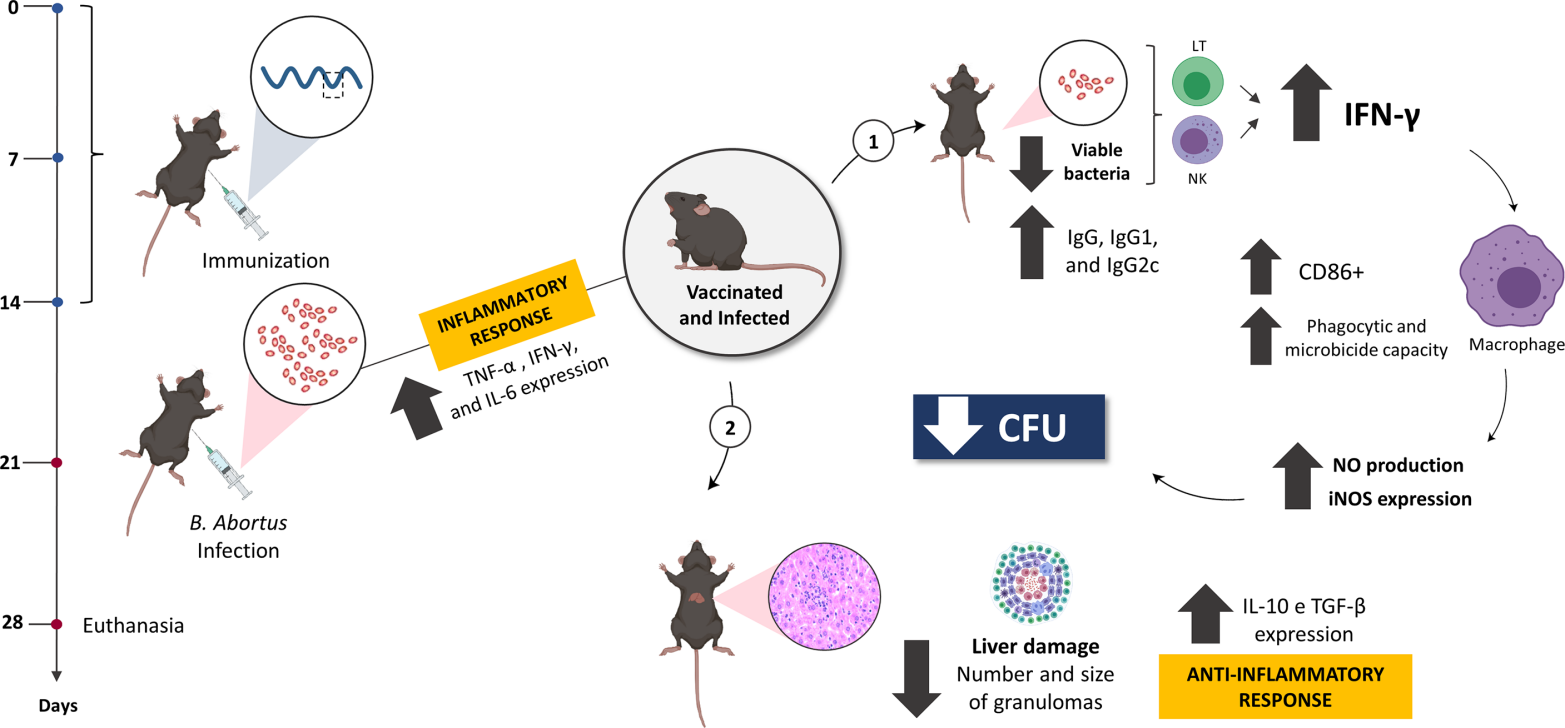
The model proposed in this study suggests the optimal efficacy of the rationally predicted vaccine peptide. After being immunized and challenged, C57BL/6 mice were more resistant to infection by *B*. *abortus*, with the less systemic recovery of viable bacteria and reduced tissue damage, mediated by an anti-inflammatory response. When evaluating the immune response profile, the predominance of characteristic components of the Th1 profile was observed, such as IFN-γ and TNF-α, suggesting that the vaccine peptide stimulates a profile mediated by CD4+ Th1 lymphocytes to secrete specific components such as IFN-γ, that mediates the production of NO and acts directly on infected macrophages, enhancing their microbicidal and phagocytic capacity and controlling the spread of systemic infection by this bacterium. Created with BioRender.

Since immunization triggered an effective adaptive immune response *in vivo*, the activation of BMDMs when stimulated with supernatant from splenocytes from treated animals was evaluated *in vitro*. It was observed that the supernatant from vaccinated and infected animals secreted cellular components capable of stimulating macrophages more intensely in the BMDMs compared to the other groups, even showing that these cells expressed higher levels of iNOS, and the consequent increased production of NO, an important cell signaling molecule involved in infectious diseases and the death of intracellular pathogens (111), justifying here the reduction in the number of viable bacteria recovered from the vaccinated group. *In vivo*, IL-6, TNF-α, and CD80/CD86 are required for activation of the interferon gamma-producing CD4+ Th1 and CD8+ cytotoxic T cells, a protective response induced by the host against brucellosis (81, 106, 111, 112). Although our study did not assess the predominant subset of T cells in the immune response, it is believed that the bactericidal and phagocytic function of macrophages to eliminate the bacteria was mainly enhanced by the secretion of IFN-γ and TNF-α, since these cytokines showed high expression levels in tissues from immunized animals, suggesting that the Int subunit vaccine predominantly induced an effective Th1 profile response and triggered protection against *B. abortus* infection (**Figure 9**).

The information gathered shows that the bioinformatics is a strong approach for vaccine candidate discovery as it offers a faster, cheaper, and safer method to identify potential vaccine targets when compared with traditional laboratory identification methods, particularly when dealing with risk group 3 microorganisms such as *Brucella*. Here, we provide an RV strategy that was able to identify a *B. abortus* antigen that is found to be strongly associated with bacterial virulence. Thus, immunization with the peptide vaccine had a significant effect on protection against murine infection, inducing an immunoprotected response; therefore, it is plausible to assume that this antigen can form a solid basis for designing an efficient and safe vaccine against animal brucellosis.

## Data Availability Statement

The datasets presented in this study can be found in online repositories. The names of the repository/repositories and accession number(s) can be found below: NCBI BioProject, PRJNA765312.

## Ethics Statement

The animal study was reviewed and approved by Committee on the Ethics of Animal Experiments (CEUA-UNIFAL 16/2020).

## Author Contributions

KO and LAA performed study design. LAA, RS, PC, and SO involved in contribution of study materials. KO, GB, LPA, and EN provided guidance for analytical tools and performed bioinformatic analysis. KO and NS prepared figures and reagents. KO, GB, and NS performed acquisition and collection of data *in vitro* and *in vivo*. KO and LAA were involved in manuscript preparation. All authors contributed to the article and approved the submitted version.

## Funding

This study was supported in part by the Coordenação de Aperfeiçoamento de Pessoal de Nível Superior—Brasil (CAPES) (Finance Code 001) and Fundação de Amparo à Pesquisa do Estado de Minas Gerais (Grant 864/14) and Universidade Federal de Alfenas and the Brazilian Ministry of Education (MEC) (EDITAL Nº 002/2020 - PRPPG/REITORIA).

## Conflict of Interest

The authors declare that the research was conducted in the absence of any commercial or financial relationships that could be construed as a potential conflict of interest.

## Publisher’s Note

All claims expressed in this article are solely those of the authors and do not necessarily represent those of their affiliated organizations, or those of the publisher, the editors and the reviewers. Any product that may be evaluated in this article, or claim that may be made by its manufacturer, is not guaranteed or endorsed by the publisher.

## Supplementary Material

Supplementary Figure 1 Arrangement of hotspots along the genome of *B. abortus* bacteria expressed during macrophage infection. **(A)** On chromosome I of *B. abortus* there is the formation of two hotspots characterizing regions of intense mapping of small RNAs and **(B)** on chromosome II there is the formation of only one hotspot.

Supplementary Figure 2 Reads and number of each read from the small RNA sequencing.
