# Supplementary figures and images for: Epitope-Based Vaccine of a Brucella abortus Putative Small RNA Target Induces Protection and Less Tissue Damage in Mice
Source: Front Immunol. 2021 Dec 21;12:778475. doi: 10.3389/fimmu.2021.778475 (PMC8724193; doi:10.3389/fimmu.2021.778475)

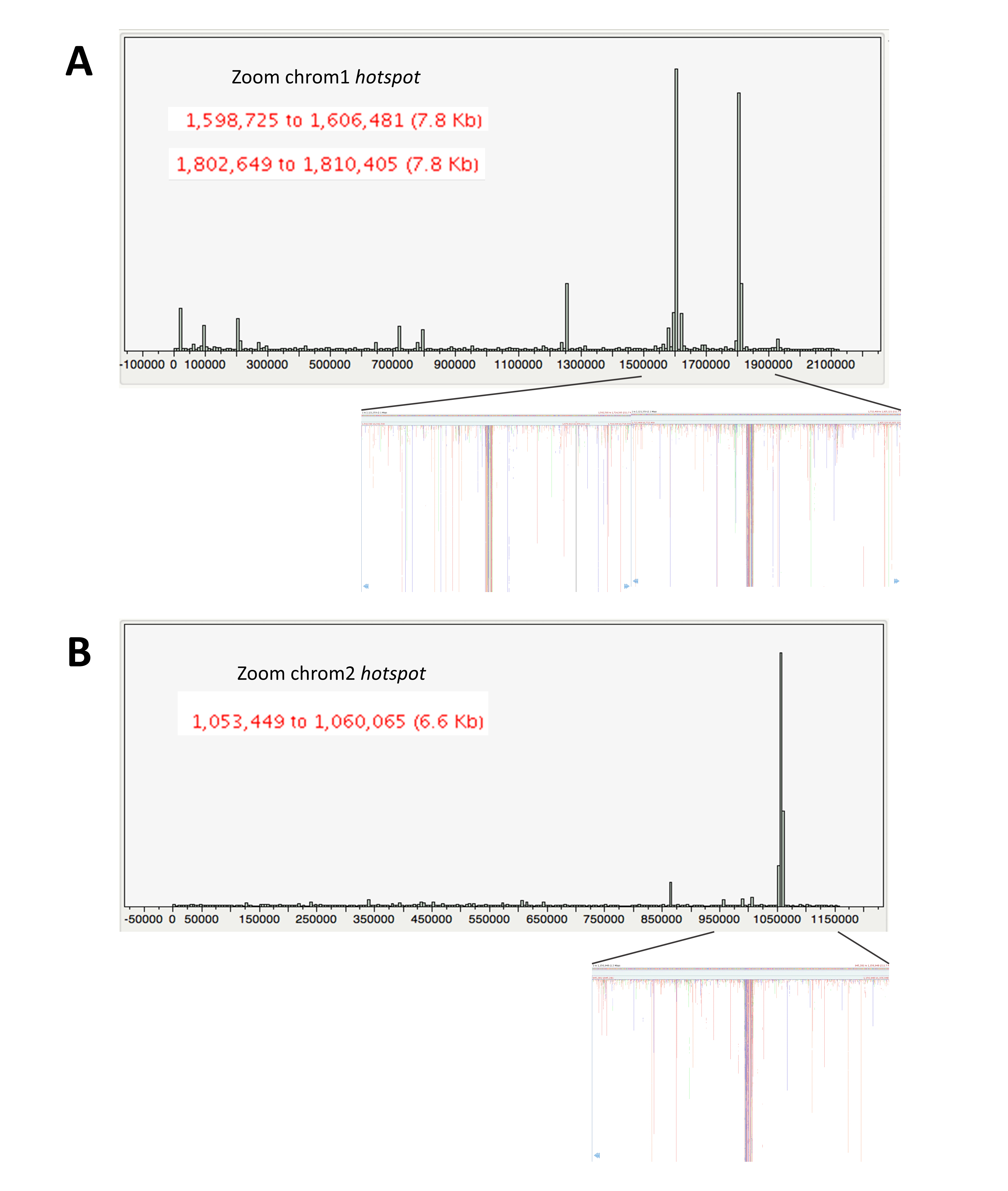

Supplement: Supplementary Figure 1 — Arrangement of hotspots along the genome of B. abortus bacteria expressed during macrophage infection. (A) On chromosome I of B. abortus there is the formation of two hotspots characterizing regions of intense mapping of small RNAs and (B) on chromosome II there is the formation of only one hotspot. [file Image_1.tif]
