# Supplementary material for: Epitope-Based Vaccine of a Brucella abortus Putative Small RNA Target Induces Protection and Less Tissue Damage in Mice
Source: Front Immunol. 2021 Dec 21;12:778475. doi: 10.3389/fimmu.2021.778475 (PMC8724193; doi:10.3389/fimmu.2021.778475)
Supplement: Supplementary Figure 2 — Reads and number of each read from the small RNA sequencing. [file DataSheet_1.pdf]

Sequence/Number of reads

TGCGAAGTCGCAAGACGACGT 199
GGGCTGGGCTACACACGGGCT 14
ACAGTACCTGAAACCGGATGC 7003
TGTATAATGGGTCAGCGACTT 63
TACCTGACTTTGGTTTTCGGA 19
AGGACCGAACCCATATCTGTT 29
TGACAAGTCGAGCAGAGACGA 17
CGGGATGGAGCAGCCCGGTAG 15
GAGTATTCCCTATCAGAGCCG 16
TGCTCACGGGCCGTACCGCAG 17
TCTCAGTTCGGATTGCACTCT 187
TGGCATAAGCCTGCCTGACTG 278
CATAGTGATCCGGTGGTCCCG 581
AAAACAACCAGGATGTTGGCT 70
TCGTCAGCTCGTGTCGTGAGA 241
ACCAACTTCGATCCGAAAACC 17
GTGGGCCTGCAGGTGGTGACG 352
ACCGAATACCCCCGGGGGTAG 271
GCGTAAAGCGCACGTAGGCGG 342
TACTCCGGGGATAACAGGCTG 77
GGCTCAAGCCATACACCGAAG 429
GAAGTTGGAATCGCTAGTAAT 123
GGGCGGCGGCGCCGGCAGCGG 39
ACTCCGCTAGTAGTGGCGAGC 65
TGGCGGCAGGCTTAACACATG 183
TGGCGGAGCGGCTGCACCCGA 4251
GGTCTGACGCCTGCCCGGTGC 1389
ACGCGTAGAACCTTACCAGCC 12
AGTTTGACTGGGGCGGTCGCC 286
GATGACTTGTGGCTAGGGGCG 33
GGAGTACTAGTCGGCAGACAC 16
ACGACGTATAGGGTCTGACGC 701
TACGGTCGCAAGATTAAAACT 188
GTGATACGCTGCGATAAGCGT 29
TTACTGATGAAAATGGATTGA 10
TTATGGCGGAGCGGCTGCACC 9521
CCCCGGCTAACTTCGTGCCAG 69
GGCAACCATAGGGGGGTGGCA 33
TTGGGGGTCATCAGCCTGTTA 15
CACGCTTGATAAGCGTGGGGT 16
CGCTCAACGGATAAAAGGTAC 201
ACGTCAAGTCCTCATGGCCCT 118
AACGCGCAGAACCTTACCAGC 73
AGATAGCTGGTTCTCCGCGAA 42
TTCAGAACGTCGTGAGACAGT 122
TCAAACGAGACTCCGCTAGTA 28
CTGGAGGTATCGGAAGTGAGA 23
TCGGAGATATTCGGAGGAACA 21
CCTAAGGCGAGGCCGAAAGGC 15
GGGCTGTAGCTCAGCTGGGAG 274
CCCGCTGTCTCCAACGCAGAC 819
GACCATTACTAACGCTGAGGT 10

TGAGTGATGAAGGCCCTAGGG 14
CGGCTAACTTCGTGCCAGCAG 14
TTATGGCGGAGCGGCTGCACT 14
CGCTGAGGTGCGAAAGCGTGG 74
TGACGGGGGCCCGCACAAGCG 156
TACACACGTGCTACAATGGTC 12
ACAGGGCTCTGCGAAGTCGCA 1833
CTGCAACTCGAGTGCATGAAG 13
GAAGCAACGCGCAGAACCTTA 24
ACCAGGCCAGTGGCTTTTGTG 177
CGCGTAGTAGCGTTTGCGTCG 10
GGCTCAAGCCATACACCGAAA 23
CGGGACACGTGAAATCCTGTC 20
ATCCGACGATTTCCGAATGGG 131
TGACGCTGAGGTGCGAAAGCG 63
ATGGTAGAGGTGAGTGGAATT 34
TGGCGGAGCGGCTGCACCCGG 12
AAAAACACAGGGCTCTGCGAA 70
TTGGAATCGCTAGTAATCGCG 1315
AGAGTTTGATCCTGGCTCAGG 11
GCTTTGCAAGCAGGGGGTCGT 89
GAGTGAAACAGTACCTGAAAC 22
CAACCATAGGGGGGTGGCACA 29
AACCTGGGGAACTGAAACACC 14
CGGCATTGTCTGCGGATGGTG 51
CCGAAAGTCCAAGGGTTCCTG 10
ACTTGATCACTCCCATTTACC 13
GTGTAGGCGCAGCGAAAGCGA 11
GTTAGGCGGAAGAGATTTTGG 11
ACATCCTGGAGGTATCGGAAG 45
AATGGTACTTCGTCTCAAGAC 102
AAGATGCGGGGTTCCTGCGGT 38
CACGGTAGGGTCAGCGACTGG 107
TGAGGTGCGAAAGCGTGGGGA 80
GCGAGGCCGAAAGGCGTAGTC 11
AAAGCCATCTCAGTTCGGATT 113
TGGTTGACAGGTTGGTTTGAC 10
ACTTCGGAAGAAGCGTGACCT 90
AGTTTGACTGGGGCGGTCTCC 45
TCGGGATGACTTGTGGCTAGG 16004
AGATCGGGATGACTTGTGGCT 435
AGTGCGGCAACGCATGCAGCT 11
GGGGAACTGAAACATCTAAGT 210
GGATAGGTGGTAGACTTTGAA 35
ACGGGATAACCGCTGAAGGCT 30
TCAAGTCCTCATGGCCCTTAC 108
TAAGCGTGGGGTCGGAGGTTA 41
CCTTTGATCCGACGATTTCCG 1001
TTGACTGATCTAGAAGCCCGG 37
CCCATTCCGAACTCGGCCGTG 112
GGGTGGAGCAGCCCGGTAGCT 210
AATTTTGGACAATGGGCGCAA 52
TTATGGCTAAGTGGGAAAGGA 100
CCCGGTCGCGGTTAGTGGAGA 251

GTTAGCCGGCCCCTAAGGCGA 56
GTTTGACTGGGGCGGTCTCCT 40
TTTTACATGGGGGTGCGACTG 12
TCGTCGGTTCGATCCCGTCCG 248
GCGAAATTCCTTGTCGGGTAC 24
ATTCGTGCAGGTCGGAACTTA 11
AAATTCCTTGTCGGGTAAGTA 15
ATATTGGACAATGGGCGAAAG 41
GGGGGTCATCAGCCTGTTATC 15
AACGGATAAAAGGTACGCCGG 68
TACTTGATGAGGGGCCGTAGC 105
TCGGGATGACTTGTGGCTAGA 12
TTGTCGTCAGCTCGTGTCGTG 17
AGAGTTTGATCCTGGCTCAGA 444
TTAATCTGAGCAGGGTTAGCC 74
ACTTCGTGCCAGCAGCCGCGG 23
CGGCATTGTCTGCGGATGGTA 45
AACTGAAACATCTAAGTACCC 32
ACGTATGTGCGTGGTAGCGGA 67
TGGGGAATTTTGGACAATGGG 51
CTGGTTCTCCGCGAAATCTAT 11
ATTCGTGGGCCTGCAGGTGGT 432
CCGCTAGTAGTGGCGAGCGAA 52
GGTAGAGAATACCAAGGCGCT 24
ACAAGTCGAGCAGAGACGAAA 28
CCCGCACAAGCGGTGGAGCAT 12
ATAAAGGGAGTGAGAGACTCC 23
AGAACATAGATCGCAGGCCAG 27
GGCGGCATTGTCTGCGGATGC 16
CTTGAGTATGGTAGAGGTGAG 31
GCGTCGGTATCTGGGCTTGTA 54
ACGGGATAACCGCTGAAGGCC 54
TGCGTCGGTATCTGGGCTTGT 50
TAAGCGTGGGGTCGGAGGTTG 20
GGTGTAGGCGCAGCGAAAGCG 23
CAGTTCGGATTGCACTCTGCC 12
AGCTATGTACGGACGGGATAA 11
ATTGGAAAGAGGCCGGATTTA 10
CTCGACCGAAGTGGGTGATAG 251
TTGAGAGTTTGATCCTGGCTC 1944
TTGATAAGCGTGGGGTCGGAG 32
ATGGCGGAGCGGCTGCACCCT 10
GAAGCTTACCGGTACTAATAG 90
CCTTTTGTATAATGGGTCAGC 25
CGGGGTTCCTGCGGTTAGACG 622
CACTCTGCAACTCGAGTGCAT 161
ATGACTTGTGGCTAGGGGGGA 39
AGGGCAACAACCCTGACCACC 182
TTGGGTTCAGAACGTCGTGAG 145
TTTGATCCTGGCTCAGAACGA 2715
CGTCGGCCCATGTGGGCCGCC 20
GTAACGATAAAGGGAGTGAGA 49
ACGATTTCCGAATGGGGAAAC 19
CCGACGAGGGGAGTGAAACAG 14

TCTAAGTACCCAGAGGAAAGG 416
CGGGGGAAAGATTTATCGGCA 20
CCGTATGTGCCCTTCGGGGGA 61
TTCTCATGTTTGTGTTCTTCG 21
GCGGACCTGGGGAACTGAAAC 12
ACGAGAGGACCGGGATGGACA 38
GTCGTGAGACAGTTCGGTCCC 620
ACTCACCGTCTTACTGATCCT 586
CTAGCGTTGTTCGGATTTACT 124
TTAAGAGGAGAGGTGCAAGCC 107
GCTCACGGGCCGTACCGCAGC 14
GAGCACCTGCTTTGCAAGCAG 34
GCAGACCTGGCAGCGACCTAC 14
ATGCAAGTCGAGCGCCCCGCA 80
CTAGTACGAGAGGACCGGGAT 103
TACCTGAAACCGGATGCATAC 11
ATGTTGGCTTAGAAGCAGCCA 99
CGTTAGTAGTGGCGAGCGAAC 13
GGTTTGTCCTGGGTGACAGCG 32
AGTGGGGAATATTGGACAATG 29
GCTGCATGGCTGTCGTCAGCT 1461
TGAAGTCGGAACAAGGTAGCC 20
AGGGGTGAAAGGCCAATCAAA 12
CGATTTCCGAATGGGGAAACC 19
GACGTATCTCTGGTGGACCTG 60
GCGTAGTCGATGGGAACCACG 64
AGAGTAACGGAGGCGCGCGAT 20
GGATAAAAGGTACGCTGGGGA 13
ATCTGCCGTGGGTGTAGGAAT 16
CACTATCCTTCAGTTAGGCTG 21
CTCAACGGATAAAAGGTACCC 31
CAAATCCTGCCCCCGCAACCA 32
TGGGTCGACCACGATCCAAGC 973
ACTCGGCCGTGAAACGCTCGA 14
TCTAAGCGGGAAACCCACCTG 310
CAGCAGTGGGGAATATTGGAC 15
ACGAGAGGACCGGGATGGACG 5119
GCGGCATTGTCTGCGGATGGA 18
TTTGCGTCTCCGTGTTTTACA 27
CTCGAGTGCATGAAGTTGGAA 56
GTACTTCGTCTCAAGACGCGG 74
TCCGTAAGCCTGTGAAGGGAC 35
GGCTGGGCTACACACGTGCTC 10
ATCGTTCGTCGAGTGCAATGG 33
GGTAGGGTCAGCGACTGGGGT 112
GTGATCAAGTGTCTTAAGGGC 99
GGTGTAGGAATATTGACAGGA 52
GCGAACCTGGAGAACTGAAAC 11
GTGGGGAGTTTGACTGGGGCG 230
TCGCAAGATTAAAACTCAAAG 185
CGGTAGGGTCAGCGACTGGGG 122
GGTGATAGTCCCGTACACGTA 29
TGGGTGTAGGAATATTGACAG 44
TGAACTTTGGCGGACACGTTT 19

TCCGGTGGTCCCGCGTGGAAG 45
ACCATTACTGACGCTGAGGTC 16
CGGGTGAGTAACGCGTGGGAA 18
CCCTATCTGCCGTGGGTGTAG 175
TCCGCCTGGGGAGTACGGTCG 3545
TTCCTGCGGTTAGACGGAAAG 17
CTCACTGGTCTAAATAAGGGT 23
AACCACCAGGTCGGCGAAGAA 54
TGACTGGGGCGGTCTCCTCCT 22
CTGGGGAACTGAAACATCTAA 580
TAGGGTTGTAAAGCTCTTTCA 1618
ACGACATGTGTAGGATAGGTC 14
GTAGTTTGACTGGGGTGGTCG 15
AGTAATCGCGGATCAGCATGC 158
CTGACTTTGGTTTTCGGATCG 81
CGCAACGAGCGCAACCCTCGC 114
AACATAGATCGCAGGCCAGTC 34
CGCGGGGTGGAGCAGCCCGGT 286
TCACGGGCCGTACCGCAGCTG 95
GACCTGGTGGTTATGGCGGAC 53
GATTTGTGAGTAGTTGGGGGT 15
CGTGAAACGCTCCAGCGCCAA 3392
GAAGACCACCACGTTGATAGG 22
GCAACGCATGAAGCTTACCGG 13
GTCGACTCATCGCATCCTGGG 256
CTCCGCTAGTAGTGGCGAGCG 66
CTCCTACGGGAGGCAGCAGTG 71
AGCTGTGGATGCACGTATGTG 31
TATTCCCTATCAGAGCCGTGG 18
GGCGTAAAGCGCACGTAGGCG 267
GAGTAACGGAGGCGCGCGATG 65
CGGAGGCGCTCGATGGTAGGC 10
TTCGGAGGAACACCAGTGGCG 3954
ATGACTTGTGGCTAGGGGTGA 2701
TGGCCGTGAAACGCTCCAGCG 16
TCAGAACGTCGTGAGACAGTT 315
AGCCTGATCCAGCCATGCCGC 132
CGCGGGGTGGAGCAGCTCGGT 10
GACCTGCACGAATGGCGTAAC 103
TGCAACTCGAGTGCATGAAGT 13
TTTCTAGTCATCATAAATAAG 426
GACTCACCGTCTTACTGATCC 470
TAGCTCAGCTGGGAGAGCACC 5328
ACGGAGGCGCGCGATGGGAGG 11
AAGTACCCAGAGGAAAGGACA 573
TGATCGGCCCGCGTTGGATTA 207
AGTAATCGCGGATCAGCATGT 20
AGTCTGAACAGGGCGTTCAGT 26
GCGTAGAACCTTACCAGCCCT 12
GTACGGTCGCAAGACTAAAAC 10
TGTGCCCTTCGGGGGAAAGAT 10
CTCTGCGAAGTCGCAAGACGA 304
ACGGATAAAAGGTACGCTGGG 15
AAGATTAAAACTCAAAGGAAT 81

GATGATCAGCCACACTGGGAC 6092
TGGAGGTATCGGAAGTGAGAA 16
TGGCTGTTCGCCATTTAAAGC 12
GGGCAACCATAGGGGGGTGGC 37
TGGGTTCAGAACGTCGTGAGA 172
AATTCGTAGATATTCGGAAGA 32
ACCTTAGATGACTAGAAAATC 169
TAATATTCGTGGGCCTGCAGG 39
CGATGTCGACTCATCGCATCC 45
GGGTCGTCGGTTCGATCCCGT 334
GGGGAATTTTGGACAATGGGC 53
CGAGTGCATGAAGTTGGAATC 236
TGACTTTGGTTTTCGGATCGA 81
TCTATTTAGGTAGAGCGTCGA 25
ATGACTTGTGGCTAGGGGTGG 107
CTCGTGCATGACCGATAGCGA 315
AGACGAAAGTCGGCCATAGTG 10
TTACTTGATGAGGGGCCGTAG 102
TGCATGAAGTCGGAATCGCTA 10
AAGTCGAGCGCCCCGCAAGGG 69
CTGAGCAGGGTTAGCCGGCCA 31
GAGAAGCTGGTCTTTCTGCTG 304
GGCCCCTAAGGCGAGGCCGAA 13
GAGTGCAATGGCATAAGCCTG 24
TCAGTTCGGATTGCACTCTGC 211
AACGCTCCAGCGCCAATGGTA 54
CGGGGTGGAGCAGCTCGGTAG 10
AAACATTCCGCCTGGGGAGTA 152
CCTTAGGGTTGTAAAGCTCTT 15
ATCGGATCAACTGAAGAGTTG 14
GATACGCTGCGATAAGCGTCG 32
TGGGCAACCATAGGGGGGTGG 30
TATCTCTGGTGGACCTGTTGT 19
CGAGCAGAGACGAAAGTCGGT 129
GTGGTAGACTTTGAAGCAGGG 255
CGGCCCCTAAGGCGAGGCCGA 16
CCGGTAGGTGTAGGCGCAGCG 29
GCGCAGCGAAAGCGAGTCTGA 21
ACCCTGGTAGTCCACGCTGTA 32
ATGGGAACCACGTTAATATTC 23
TCAGCCACACTGGGACTGAGA 297
GAGGCGCGCGATGGTAGGCCC 31
ACGATAAAGGGAGTGAGAGAC 795
GCAGCCCGGTAGCTCGTCAGG 464
TCGGGGGAAAGATTTATCGGC 34
CCTAGTACGAGAGGACCGGGA 28
GGGGAGTACGGTCGCAAGATT 69
GGCGGCAGGCTTAACACATGC 165
TGATACTGGAAGTCTTGAGTA 18
CCATTACTGACGCTGAGGCGC 25
TAGTAATCGCGGATCAGCATG 155
CGGTTCGATCCCGTCCGGCTC 684
GTGAGTGATGAAGGCCCTAGG 17
TTCGCCATTTAAAGCGGTACG 12

TTGACTGGGGCGGTCGCCTCC 141
GTACTCCGGGGATAACAGGCT 85
GTACGGTAACACGTACTGGAG 37
GTTAGGCTGGACCGGAGACAG 184
AAGTCCTCATGGCCCTTACGG 112
AAGTGGGTGATAGTCCCGTAC 71
GTCCCGCAACGAGCGCAACCC 74
GGACTGCCGGTGATAAGCCGA 2047
GCTTGAGAGAACTGCGTTGAA 1814
TCAGTGAAATTGAATTCCCCG 38
AAGAAGCGTGACCTCACTATG 89
CCGGCCCCTAAGGCGAGGCCG 16
TAATGGGTCAGCGACTTAGTG 68
AATGGTGGTGACAGTGGGCAG 94
AACTCAGGGAAACTTGTGCTA 22
TGATGAAGGCCCTAGGGTTGT 108
GCCGGGAAACGCTCCAGCGCC 18
AATATTGGACAATGGGCGAAA 30
CGCCTGCCCGGTGCTGGAAGG 168
TGAGCAGGTTGAAGGTACGGT 503
ACGGTAGGGTCAGCGACTGGG 129
AACCCTGACCACCATCTAAGG 29
TACAATGGTGGTGACAGTGGG 16
TTGATCCGACGATTTCCGAAT 308
ACAGTCGTGAGACATCCTGGA 37
TCGTCTCAAGACGCGGGAGAG 157
AGACCCCGTGCACCTTTACTA 10
GGGCCATCGCTCAACGGATAA 95
GGTGTGACGCCTGCCCGGTGC 11
GGGTGAAGTCGGAACAAGGTA 15
GGAGGCGCGCGATGGTAGGAT 47
GAGAAGAAGCCCCGGCTAACT 29
TCGAGAATTGGAAAGAGGCCG 107
CTTTGGCGGACACGTTTCTTG 175
TTCGGTCCCTATCTGCCGTGG 1406
TCGACTTGATCACTCCCATTT 387
GGGTCGGAGGTTCAAGTCCTC 283
TTTTGGACAATGGGCGCAAGC 64
GATCGAGAATTGGAAAGAGGC 453
TAATTCGAAGCAACGCGCAGA 28
TCAGGGGTGAAATCCCGGGGC 73
GACCATTACTGACGCTGGGGT 16
GACACTGGTGGACTGGTAGAG 376
CAGTGGCGAAGGCGGCTCACT 373
CTCCGAATACCGGGGAGTACT 20
ACGGTAACACGTACTGGAGGA 288
TAGTAGTGGCGAGCGAACGCG 34
TAGTGATCCGGTGGTCCCGCG 109
GGGGGCTAGCGTTGTTCGGAT 877
CACGGCCCAAACTCCTACGGG 12
CCATTACTGACGCTGAGGAGC 10
TAACCACCAGGTCGGCGAAGA 52
TCGTGAGACATCCTGGAGGTA 117
CTAAGTGGGAAAGGATGTGAG 140

AACGGTCGGAAATCGTTCGTC 118
TTTGATCCGACGATTTCCGAA 523
ACTTGATCACTCCCATTTACA 34
AGCACTGGATGGGCTATGGGG 77
CGGCATTGTCTGCGGATGGTT 156
TTGGCACCTCGATGTCGGCTC 11
CTCAGAACGAACGCTGGCGGC 2696
CGGCCGGGAAACGCTCCAGCG 19
TTCAAATCCTGCCCCCGCAAC 29
ATGGAGCAGCCCGGTAGCTCG 15
ATGACCGATAGCGAACCAGTA 129
AAATTCCTTGTCGGGTAAGTT 315
CTTAACACATGCAAGTCGAGC 30
GCGAAATTCCTTGTCGGGTAG 17
TGAAGGCCGCAGGTTCAAATC 42
TTGGTGGGCCTGGGAGGACTT 12
GGCCTAACACATGCAAGTCGA 10
TAAGCGTGGGGTCGGAGGTTC 2466
GGAACCACGTTAATATTCGTG 23
ACGGGATAACCGCTGAAGGCG 16
TTATGAGCCTGACGAGCTACC 896
GGCGGCATTGTCTGCGGATGG 768
ACGTATAGGGTCTGACGCCTG 944
GGGAACCTGCGGCTGGATCAC 34
AATAGCAGAAGTCCTTGAGTA 91
GAGGTTCAAGTCCTCCCAGGC 41
GGCAACGCATGCAGCTTACCG 29
CCCGGTCGCGGTTAGTGGAGC 10
GCGAACCTGGGAAACTGAAAC 12
CAGAGACGAAAGTCGGTCATA 90
TAAGCGTCGGGGAGGTGCGAA 392
CATAGCTCAGCTGGGAGAGCA 12
ACCGGTACTAATAGCTCGATC 5375
TACGTGAGTTGGGTTCAGAAC 97
ACGTCGTGAGACAGTTTGGTC 26
GTAATCGCGGATCAGCATGCC 156
GCGAAGTCGCAAGACGACGTA 60
GTGCTGGGCTACACACGTGCT 10
GAGCAAGCTTAAGCCGGTAGG 104
GTTCCCGGGTCTTGTACACAC 16
CCCAGAGGAAAGGACATCAAA 18
TGGTGGTGACAGTGGGCAGCG 71
CGTACACGTAGAATAGCAGAA 175
ATCGTCGGTTCTTTGAAAACT 12
GGATGATCCGCCACACTGGGA 17
TTAGAAGCGAACCTGGGGACC 10
GCGAAATTCCTTGTCGGGTAA 4574
ACGTCGTGAGACAGTTCGGTC 799
CCTGCATATAGACCGTACCCT 774
CGGCATTGTCTGCGGATGGTC 42
GTAGGGCGGGACACGTGAAAT 15
GTGGTGACAGTGGGCAGCGAG 180
GGCGTAGTCGATGGGAACCAC 67
GACTTCCCCGCTGTCTCCAAC 191

TGCACGTATGTGCGTGGTAGC 35
ACAACCCTGACCACCATCTAA 45
AGGGCTCTGCGAAGTCGCAAG 1119
ACACTGGTGGACTGGTAGAGA 2095
GGCGGCATTGTCTGCGGATGA 18
ATTAAACATTCCGCCTGGGGA 707
AGCCACATTGGGACTGAGACA 11
ATGGCGTAACGACTTCCCCGC 17
AATATTGGACAATGGGCGCAA 379
GAGTCTGAACAGGGCGTTCAG 41
CTTGGCATGCACAGGCGATGA 232
CCGTCGTGGAGAGGGCAACAA 22
GACCGAACCCATATCTGTTGC 17
CAGTTCGGATTGCACTCTGCA 68
GATGACGTCAAGTCCTCATGG 10
CGCAACCCTCGCCCTTAGTTG 11
CCTTTGATCCGACGATTTCCC 11
TAAGCGTGGGGTCGGAGGTTT 11
CATTCGTGACGACATGTGTAG 33
TGGTAGAGAATACCAAGGCGC 33
ACGGGATAACCGCTGAAGGCA 888
GGGGAACTGAAACATCTAAGA 11
CAGGTGCTGCATGGCTGTCGT 753
ACGTGAGTTGGGTTCAGAACG 22
TTACCAGCCCTTGACATCCCG 65
CAGGGGTGAAATCCCGGGGCT 45
CGACCGAAGTGGGTGATAGTC 255
ATGGCGGAGCGGCTGCACCCG 6875
TCGCTAGTAATCGCGGATCAG 83
CAATGAGAGGGATCAAGTGTC 21
CGGATTTACTGGGCGTAAAGC 2580
ACGGGCTGGGCTATACACGTG 16
TAGAGGTGAGTGGAATTCCGA 98
GCCGCGGTAATACGAAGGGGG 289
CAAGGCGCTTGAGAGAACTGC 62
GATGGTAGGCTCAGAACGGTC 193
CATCGCATCCTGGGGCTGGAG 35
GACACTATCCTTCAGTTAGGC 16
GGAACTGAAACATCTAAGTAC 155
TGAAGGGACAGTCGTGAGACA 25
TTTTACCCGAAGGCGCTGTGC 387
GGAGGACCGAACCCATATCTG 31
GAGTTTGATCCTGGCTCAGAA 357
GTATGTGCCCTTCGGGGGAAA 45
GAGGCGCGCGATGGTAGCCTC 12
TAATCGCGGATCAGCATGTCG 19
ACCGGAGACAGGTGCTGCATG 371
CTGGAGAGTTTGATCCTGGCT 12
ACCATTACTGACGCTGAGGTG 16058
CCGTGAAACGCTCCAGCGGCA 11
CACGTATGTGCGTGGTAGCGG 55
AATGGGTCAGCGACTTAGTGT 84
GAAGCGAACCTGGGGAACTGG 38
GCTGAGGTGCGAAAGCGTGGG 87

ACCATTACTGACGCTGGGGTG 16
GCCTGACGAGCTACCGGGCTG 17
TGGGCTACACACGTGCTACAA 321
TTGCAGACCTGGCAGCGACCT 21
GGGCTCAACCCCGGAACTGCC 36
GCGGCATTGTCTGCGGATGGT 509
AGCCATACACCGAAGCTGTGG 27
GGGGATAACAGGCTGATGACC 10
TGGTAGGCTCAGAACGGTCGG 29
TTGTATCTCGAGAAGCTGGTC 30
GCAACGAGCGCAACCCTCGCC 117
ACTTCCCCGCTGTCTCCAACG 273
GGGTGTAGGAATATTGACAGG 42
TCCCAAAACAACCAGGATGTT 87
AAACCAAGTGATCTAGCCATG 11
CATGAAGTTGGAATCGCTAGT 80
AGTGATCCGGTGGTCCCGCGT 68
TGAACCAGTACCGTGAGGGAA 29
TTGAAGGAACTCGGCAAAATG 163
TGAATACGTTCCCGGGCCTTG 180
CGTGATACGCTGCGATAAGCG 29
AGTTCGGATTGCACTCTGCAA 51
ATGGCGGAGCGGCTGCACCCA 12
GCAGCGAAAGCGAGTCTGAAC 25
GAGAGGATGATCCGCCACACT 11
AGAGCATACCAAGGCGCTTGA 10
GCGTAGTAGCGTTTGCGTCGG 10
CCAAAACAACCAGGATGTTGG 87
GCGCTGACACGGATTTGACCT 12
GAGGTATCGGAAGTGAGAATG 20
GGCTGGGCTACACACGTGCTA 13752
GAAGCGAACCTGGGGAACTGA 9180
GACTTGATCACTCCCATTTAC 235
GTGAAGGGACAGTCGTGAGAC 33
ATTTGGTGGATGCCTTGGCAT 37
GGTAGCCGTAGGGGAACCTGC 13
AGGGTTAGCCGGCCCCTAAGG 340
GGTGGAGCAGCCCGGTAGCTC 191
AGCAGAAGTCCTTGAGTAGGG 188
GCGGCATTGTCTGCGGATGGC 49
AGTTGGAGCCCAAGGTTTGTC 80
ACGAGAGGACCGGGATGGACT 15
ACCATTACTGACGCTGAGGTA 48
TAGCTCGTCAGGCTCATAACC 336
TTGGGCTGCGCCTGTTCTTTG 32
GACGTCAAGTCCTCATGGCCC 41
GGAGTTTGGTTAGGATCAGTA 17
TGTAGGAATATTGACAGGATC 119
CGGCTCACTGGACCATTACTG 8214
CTACACACGTGCTACAATGGT 26
TCGAGCAAGCTTAAGCCGGTA 87
TGCCTTTGATACTGGAAGTCT 10
CGTATAGGGTCTGACGCCTGC 916
CTGGGGAACTGAAACATCTAC 13

GAAATTCCTTGTCGGGTAAGT 2169
CTGCATGGCTGTCGTCAGCTC 1435
ATAGGCCGGGTGTGGAAGTGC 297
TTTGTGAGTAGTTGGGGGTGG 20
CGTTCAGTTCGATGCATTAGA 322
AAAAGGTACTCCGGGGATAAC 72
ACGCTCCAGCGCCAATGGTAC 64
CGGGTAAGTTCCGACCTGCAC 17
GAAGCTGTGGATGCACGTATG 40
AGCTCAGTTGGTTAGAGCACA 83
GGAAGGGCCATCGCTCAACGG 84
TGCATGGCTGTCGTCAGCTCG 1085
AATATGGAAGTAGGGCAATAA 11
ACGACATGTGTAGGATAGGTA 12
GCGGTTAGTGGAGACACTATC 41
CCCTTTGATCCGACGATTTCC 1200
TTCGTGCAGGTCGGAACTTAC 11
CGTGCCAGCAGCCGCGGTAAT 16
ACGGGGGCCCGCACAAGCGGT 295
CCTGACGAGCTACCGGGCTGC 20
ATGCGGGGTTCCTGCGGTTAG 129
ACACGCACTGGAGGACCGAAC 30
GACCTGGTGGTTATGGCGGAA 25
CTGAGCAGGGTTAGCCGGCCT 10
TAGCTCAGCTGGGAGAGCACA 12
TAGTCCACGCCGTAAACGATG 21
GAAACCGGATGCCTACAAACA 50
GTTTGATCCTGGCTCAGGACG 13
GACATGTGTAGGATAGGTGGT 984
CGGCCCAAACTCCTACGGGAG 14
CCATCTCAGTTCGGATTGCAC 111
ATAGCTCGATCGACTTGATCA 43
CTGGTCTGAGAGGATGATCAG 10
TCCGACGATTTCCGAATGGGG 182
CCGCAAGGAGGCAGGCGACCA 21
GTTTGGTTAGGCGGAAGAGAT 11
CGAGTGCAATGGCATAAGCCT 49
TCCTGCATATAGACCGTACCC 706
GGCTGGGCTACACACGTGATA 24
CCGACACTGGTGGACTGGTAG 413
AAGTACCCAGAGGAAAGGACC 13
ACGACATGTGTAGGATAGGTG 1113
AATAACGGTCCTAAGGTAGCG 10
GATTTCCGAATGGGGCAACCC 14
GAGGACCGGGATGGACGTACC 10
CCGGTAGCTCGTCAGGCTCAT 17
GCAGAACCTTACCAGCCCTTG 1422
TAACACGTACTGGAGGACCGA 213
CTGAGCAGGGTTAGCCGGCCC 10966
TAGCTCAGCTGGGAGAGCACG 11
GGAAGTAGGGCAATAAGGCAA 16
TCTTGGGGGTCATCAGCCTGT 20
CGACGTATAGGGCCTGACGCC 27
GTCGAGCAGAGACGAAAGTCG 70

GGGGGTGCGACTGATTATAGC 35
TGATAGTCCCGTACACGTAGA 38
AAGGAACTCGGCAAATTGCAC 21
GAGCGGCTGCACCCGATCCCA 82
GGCCGGGTGTGGAAGTGCGGC 541
GTTGGGTTCAGAACGTCGTGA 45
ACGGGCCGTACCGCAGCTGAC 132
GTACACGTAGAATAGCAGAAG 78
GAAGGAACTCGGCAAATTGCA 20
GACCTGGTGGTTATGGCGGAG 3771
CTGATCTAGAAGCCCGGCACC 53
GACGAGGGGAGTGAAACAGTA 17
AGAGTAGGTCGCTGCCAGGTC 5710
AAATCCTGTCTGAACATGGGG 12
TCGGCCGTGAAACGCTCCAAC 11
ATACCGGGGAGTACTAGTCGG 481
GAAAGCGTAACAGCTCACTGG 206
TAGAGCGTCGACCGAATACCC 2024
CGCGGTTAGTGGAGACACTAT 44
TCTTGAGTATGGTAGAGGTGA 35
GAGGTGAGTGGAATTCCGAGT 111
TGAAGAGAAGATGTAATCGGA 23
ACCGCAGACGAGGCGCTGACA 12
GGTCGCAAGACTAAAACTCAA 12
CTTACCAGCCCTTGACATCCC 66
AGCCACACTGGGACTGAGACA 332
GACGGATCGCGTGTGTTGTGA 119
GAAGAAGCCCCGGCTAACTTC 23
CCGCCACACTGGGACTGAGAC 18
CGGAGGTTCAAGTCCTCCCAG 43
CGTTGATAGGCCGGGTGTGGA 48
CCCCTAAGGCGAGGCCGAAAG 15
CGGGCTGGGCTGCACACGTGC 11
CGGAGGCGCGCGATGGTAGGC 37976
TGAGGGAAAGGTGAAAAGAAC 13
TAGGATAGGTGGTAGACTTTG 45
AGCAACGCGCAGAACCTTACC 34
AGCGAACAGGATTAGATACCC 13
GGTGGTCCCGCGTGGAAGGGC 40
TTAAACATTCCGCCTGGGGAG 703
ATGGAAGTAGGGCAATAAGGC 35
GCCGTAAAACGCTCCAGCGCC 12
GGATAACCGCTGAAGGCATCT 93
AGGCGCGCGATGGTAGGGTCA 10
AGTCGGTCATAGTGATCCGGT 131
CTGGGCTACACACGTGCTACA 9786
GGATAAAAGGTACCCCGGGGA 75
TGATCCGACGATTTCCGAATG 254
AGTAGGTCGCTGCCAGGTCTG 876
TAGCTCCTGCATATAGACCGT 260
AATCGTTCGTCGAGTGCAATG 24
AAGGTTTGTCCTGGGTGACAG 32
CCACCACGTTGATAGGCCGGG 158
TGCCTTGGCATGCACAGGCGA 63

GGATGCCTACAAACAGTTGGA 45
CAGAACGTCGTGAGACAGTTC 263
CCATAGCTCAGCTGGGAGAGC 147
GATCGACTTGATCACTCCCAT 328
TAGTCGATGGGAACCACGTTA 21
CAAGGTAGCCGTAGGGGAACC 21
TGCCTACAAACAGTTGGAGCC 22
GTGATAGTCCCGTACACGTAG 28
GACCGTACCCTAAACCGACAC 839
CGAAAGGCGTAGTCGATGGGA 246
CGAGGGGAGTGAAACAGTACC 19
ACCTGGTGGTTATGGCGGACC 31
TGGGGAATATTGGACAATGGG 96
CGACCTGGTGGTTATGGCGGA 3949
GGATTTACTGGGCGTAAAGCG 2558
TGGGTTAAGTCCCGCAACGAG 18
GGCCGTAGCTCAGCTGGGAGC 19
AGTGGGTGATAGTCCCGTACA 26
ACGGGGTTGTTTGGCACCTCG 218
ATTTAGGTAGAGCGTCGACCG 86
CCCGGGTCTTGTACACACCGC 15
TGAGAGAACTGCGTTGAAGGA 5128
CTGGGCTACACACGTGCTACG 91
CTAACGTCCGTCGTGGAGAGG 10
TCCGACCTGCACGAATGGCGT 159
CTCGGCCGTGAAACGTTCCAG 14
TGAAGCTTACCGGTACTAATA 90
TTTCACCGGTGAAGATAATGA 24
GTTGGTTAGAGCACACGCTTG 12
GGTGAAGTCGAAACAAGGTAG 18
AGCTTTACACTGGCATTCGTG 111
ATGACTTGTGGCTAGGGGCGA 13
TAGATCGGGATGACTTGTGGC 457
GGGCTGGGCTACACATGTGCT 13
AAATGATCGGCCCGCGTTGGA 1935
TGACCCCCAAGAGTCCATATC 12
CTCACTGGACCATTACTGACA 63
AGTTCGATGCATTAGACCCGA 82
AAACTCAAAGGAATTGACGGG 172
TAGCTCAGCTGGGAGAGCGCC 26
AACTCGGCAAAATGCACGCGT 414
GAGGATGATCAGCCACACTGG 1207
GTGTAGGAATATTGACAGGAT 111
AACACAGGGCTCTGCGAAGTC 242
CCACATTGGGACTGAGACACG 33
AGCCATCTCAGTTCGGATTGC 130
GCACCTGCTTTGCAAGCAGGG 86
GTTTAGAACGTCGTGAGACAG 11
TGAGACAGTTCGGTCCCTATC 179
CAGAACGGTCGGAAATCGTTC 93
GCATGCAGCTTACCGGTACTA 125
CACGGACCAGACTCCTACGGG 28
CGGATCGCGTGTGTTGTGAGG 655
CTGGCGGCAGGCTTAACACAT 190

CGTCAAGTCCTCATGGCCCTT 59
CGCACGTAGGCGGACTTTTAA 52
AGTAATCGCGGATCAGCACGC 11
GACTTTGGTTTTCGGATCGAA 41
GGACCAGACTCCTACGGGAGG 20
GAGAGGAAGGTGGGGATGACG 30
GCAAGGGGAGCGGCAGACGGG 23
CGCATTAAACATTCCGCCTGG 578
GGTGGGGTAAAGGCTCACCAA 26
ACCTGGGGAACTGAAACATCT 4074
AGCCGGCCCCTAAGGCGAGGC 20
GCGATGGTAGGCTCAGAACGG 318
AAGGAAGATCGAGAATTGGAA 46
GTCGGCAGACACACGGCGGGT 240
CCGTGAAACGCTCCAGCGCCC 57
CTATATACGGACGGGATAACC 159
ACGAGCATTTGCAGTCGAATG 35
GTAAAGGCTCACCAAGGCGAC 36
TAGAAGCGAACCTGGGGAACT 9792
TGCAGACCTGGCAGCGACCTA 15
GCTGGTCTGAGAGGATGATCA 24
ACGTATCTCTGGTGGACCTGT 77
AGTTGGGCACTCTAAGGTGAC 26
TCGGCCGGGAAACGCTCCAGC 19
CTACGGAATAACTCAGGGAAA 173
CGTTCCCGGGCCTTGTACACA 73
ATAGGGTCTGACGCCTGCCCG 970
GTGTGGAAGTGCGGCAACGCA 194
ATAAAAGGTACTCCGGGGATA 60
GCGCAACCCTCGCCCTTAGTT 13
TAGTTTGACTGGGGCGGTCTC 48
CGCTCCAGCGCCAATGGTACT 14
GGAGGCGCTCGATGGTAGGCT 10
GCTTACCGGTACTAATAGCTC 218
GTAGAGCACTGGATGGGCTAT 19
ACCGGGGCTCAAGCCATACAC 2690
CTCACTGGACCATTACTGACG 4285
GGGAATATTGGACAATGGGCG 529
GCCCGGTAGCTCGTCAGGCTC 316
GAAACAGTACCTGAAACCGGA 55
CACCTTAGATGACTAGAAAAT 69
CCGTGAAACGCTCCAGCGCCT 16
TTACTGGGCGTAAAGCGCACC 12
AATTCGTAGATATTCGGAGGA 245
TAGAAGCGAACCTGGGGAACC 29
TAACAGGCTGATGACCCCCAA 341
CGCAAGACGACGTATAGGGTC 29
GAGGACCGAACCCATATCTGT 29
AATGTACCGGGGCTCAAGCCA 253
GGGGGTAGAGCACTGGATGGG 110
ATGAAGGACGTGATACGCTGC 51
GAAGGGACAGTCGTGAGACAT 22
TTCAAGTCCTCCCAGGCCCAC 168
AGTCAGGGGTGAAATCCCGGG 36

ACCTGGGGAACTGAAACATCC 57
GAGCTGAGTTTTGATGGATAT 279
ACAATGGTGGTGACAGTGGGC 107
CAGAGGAAAGGACATCAAACG 21
GCGAACAGGATTAGATACCCT 11
GGTCGGAAATCGTTCGTCGAG 192
CTACACTCGGAATTCCACTCA 11
GCCAATCAAACTTGGAGATAG 15
AAAGGGAGTGAGAGACTCCCT 30
TCCGAATGGGGAAACCCACCT 32
TAAAGGGAGTGAGAGACTCCC 25
AAGCGAGTCTGAACAGGGCGT 29
GTATAGGGCCTGACGCCTGCC 11
ACTCGACCGAAGTGGGTGATA 251
AGCTCCTGCATATAGACCGTA 318
GTCTCAAGACGCGGGAGAGTA 155
GCGAACCTGGGGAACTGAACC 57
ACTGATGAAAATGGATTGACT 10
CCATTACTGACGCTGAGGTGA 11
TGTTGGCTTAGAAGCAGCCAT 71
TCGATGCATTAGACCCGAAAC 25
ATGGTAGGCTCAGAACGGTCG 74
TTACCTGACTTTGGTTTTCGG 18
ATCCCATTCCGAACTCGGCCG 85
AAACAGTACCTGAAACCGGAT 188
AAGCGCACGTAGGCGGACTTT 57
CGGCCCAGACTCCTACGGGAA 43
CAAACTTGGAGATAGCTGGTT 166
CGCGGTAATACGAAGGGGGCT 407
ACGCTGGCGGCAGGCTTAACG 25
TAACACGCACTGGAGGACCGA 14
ATCCTGTCTGAACATGGGTCG 1657
CTCAGCTGGGAGAGCACCTGC 563
ATTTCCGAATGGGGAAACCCA 19
TGAGCAGGGTTAGCCGGCACC 15
CATCAAACGAGACTCCGCTAG 86
TGGGTGATAGTCCCGTACACG 17
GCCGTAGCTCAGCTGGGAGAG 8047
TCGGCCGTGAAACGCTCGAGC 10
GAGGCAGGCGACCACGGTAGG 148
TGCGGACTTTTACGAAAGTCT 10
CCATTACTGACGCTGAGGTGG 20
TAAGAGCTGAGTTTTGATGGA 284
GAACCACGTTAATATTCGTGG 18
CGGCCCAGACTCCTACGGGAG 2735
CAACTGGAGAGTTTGATCCTG 10
ACTTGGAGATAGCTGGTTCTC 849
CAGCTGGGAGAGCACCTGCTT 493
CCACGCCGTAAACGATGAATG 134
GTTGTTCGGATTTACTGGGCG 291
ATACGTTCCCGGGTCTTGTAC 11
GACCAGGGGGTAGCGACTGTT 76
GGAAGACCACCACGTTGATAG 13
CGTAGCTCAGCTGGGAGAGAA 10

CCCCGCAAGGGGAGCGGCAGA 55
GACACGTGAAATCCTGTCTGA 26
ATAGCTCCTGCATATAGACCG 96
ATGCAGCTTACCGGTACTAAT 43
TAGTCCCGTACACGTAGAATA 360
TCCTAACCAAACTCCGAATAC 81
AACACATGCAAGTCGAGCGCC 14
CGAACCTGGGGAACTGAAAAA 70
GACCACGGTAGGGTCAGCGAC 2166
TTCGTGACGACATGTGTAGGA 310
GCCGTAGCTCAGCTGGGAGAA 38
AGCTTACCGGTACTAATAGCT 161
TGCATGGTTGTCGTCAGCTCG 13
CGGGTCCAGGACCGTGTATGG 37
AAGGGCCATCGTTCAACGGAT 99
CTAACCAAACTCCGAATACCG 53
CCGCGTTGGATTAGCTAGTTG 21
GAACTGCCTTTGATACTGGAA 118
CCTGCCCGGTGCTGGAAGGTT 17
ACGGATTTGACCTTCGGGTTT 30
GGGTGCTAACGTCCGTCGTGG 13
ACGCTGGCGGCAGGCTTAACA 785
TAGGCGGACTTTTAAGTCAGG 18
ATGAAGGCCTTAGGGTTGTAA 15
GAAGGCGCGCGATGGTAGGCT 12
CGGTCGCGGTTAGTGGAGACA 94
ACTGGACCATTACTGACGCTA 12
AGGATTAGATACCCTGGTAGT 68
GCGCACGTAGGCGGACTTTTA 87
TTAAGCCGGTAGGTGTAGGCG 24
GATAAAAGGTACGCCGGGGAT 863
AAAGTCGGTCATAGTGATCCG 134
TTTAATTCGAAGCAACGCGCA 24
CGGTCCCTATCTGCCGTGGGG 24
ATACCCTTTGATCCGACGATA 16
GAGTAGGTCGCTGCCAGGTCT 6251
ATCAGCCACACTGGGACTGAG 55
TCGAGCAGAGACGAAAGTCGG 131
CTTAGATGACTAGAAAATCTG 17
AACAGTACCTGAAACCGGATG 797
ATGAGGGGCCGTAGCTCAGCT 222
CATGGGGGTGCGACTGATTAT 45
TGAGCAGGGTTAGCCGGCCCC 10105
TAGCGTTTGCGTCGGTATCTG 10
CAAAAGCCGTCTCAGTTCGGA 24
ACAATGAACTTTGGCGGACAC 16
CAAGGAGGCAGGCGACCACGG 83
AGACGACGTATAGGGTCTGAC 135
AGCCTGCCTGACTGCAAGACT 210
GGTACTAATAGCTCGATCGAC 5436
ACACCAACTTCGATCCGAAAA 41
GTGGCGAAGGCGGCTCACTGG 231
GGGCTAGCGTTGTTCGGATTT 145
CAATGGCATAAGCCTGCCTGA 48

AGTGGAATTCCGAGTGTAGAG 561
CGGCCGTGAAACGCTCGAGCG 10
CAGGATTAGATACCCTGGTAG 39
TATAATGGGTCAGCGACTTAG 64
AAAGCGCACGTAGGCGGACTT 77
TAGATCGCAGGCCAGTCAGCC 90
ACACGGACCAGACTCCTACGG 19
CGTGAGACAGTTCGGTCCCTA 162
AAAGCGGTACGTGAGTTGGGT 10
TAGTTTGACTGGGGTGGTCGC 22
TATAACGGTCCTAAGGTAGCG 10
GATGGGCTATGGGGACTCACC 2688
ACATAGGGGTTAGAAGCGAAC 127
CTGCAAGACTGACAAGTCGAG 47
TTCCTTGTCGGGTAAGTTCCG 244
CTGCCGCCAGCGTTCGTTCTG 39
ACGTTAATATTCGTGGGCCTG 103
TCCTGGCTCAGGACGAACGCT 16
GCGTTGGATTAGCTAGTTGGT 10
GCTAGTAGTGGCGAGCGAACG 66
GGGGCAACCCACCTTAGATGA 26
GGAAAGACCCCGTGCACCTTT 51
ATGTGCGTGGTAGCGGAGCGT 23
TCCCGCGTGGAAGGGCCATCG 10
ACTGGACCATTACTGACGCTG 20454
CTAGCCATGAGCAGGTTGAAG 28
GGTCCTAAGGTAGCGAAATTC 73
CCGACCTGCACGAATGGCGTA 143
GCCATCTCAGTTCGGATTGCA 93
GAGTGATGAAGGCCCTAGGGT 26
GTTCGATGCATTAGACCCGAA 56
GTAGAGGTGAGTGGAATTCCG 103
TGTGGCTAGGGGTGAAAGGCC 587
TTGTGAGTAGTTGGGGGTGGT 12
TTCCCTATCAGAGCCGTGGAA 38
GCCCGGTGCTGGAAGGTTAAG 19
GTGCATGGCTGTCGTCAGCTC 16
GGTTAGCCGGCCCCTAAGGCG 100
AGGTACTCCGGGGATAACAGG 87
TTGCGGACTTTTACGAAAGTC 14
TTAATATTCGTGGGCCTGCAG 32
AGCCTGCCTGACTGCAAGACC 19
GCCTGCCTGACTGCAAGACTG 101
GTATCTCTGGTGGACCTGTTG 19
CGGGCTGGGCTACACACATGC 11
CAGACCAGGGGGTAGCGACTG 108
GGCTGGACCGGAGACAGGTGC 695
TCAGAACGGTCGGAAATCGTT 293
GGAGGCGCGCAATGGTAGGCT 10
CTGGCTCAGAACGAACGCTGA 18
GGGGTGGAGCAGCTCGGTAGC 10
AAATCCTGTCTGAACATGGGT 205
TTAGGCGGAAGAGATTTTGGA 44
CCAGACTCCTACGGGAGGCAG 1000

CATACACCGAAGCTGTGGATG 25
AAAAGGTACGCCGGGGATAAC 211
AAGACCCCGTGCACCTTTACT 55
TAATCGCGGATCAGCATGCCG 110
CAGGTGGTGACGGATCGCGTG 13
CAGAACGTCGTGAGACAGTTG 10
GCGAACGCGGACCAGGCCAGT 2648
AGCTCAGCTGGGAGAGCACCC 13
ATTCCCCGTGAAGATGCGGGG 13
AGACAGGTGCTGCATGGCTGT 69
AGACGGAAAGACCCCGTGCAC 337
GTACTGGAGGACCGAACCCAT 2666
AACGCAGACTCAGTGAAATTG 99
AATTCCGAGTGTAGAGGTGAA 445
GATCCCAAAACAACCAGGATG 68
GTTGTCGTCAGCTCGTGTCGT 13
GACGCGGGAGAGTAGGTCGCT 919
CGGAGGCGCGCGATGGTAGGA 58
TTTACCAAAAACACAGGGCTC 18
AAGGAACTCGGCAAAATGCAC 531
CTCAAGCCATACACCGAAGCT 307
AGCGTTGTTCGGATTTACTGG 26
TCCAACGCAGACTCAGTGAAA 97
CTGGGCTACACACGTGCTACC 264
CCGCGGTGAATACGTTCCCGG 205
CGCGTTGGATTAGCTAGTTGG 12
ATGGTACTTCGTCTCAAGACG 137
TAAGCCGAGAGGAAGGTGGGG 14
TGGCGAAGGCGGCTCACTGGA 354
CGACTTAGTGTATCGAGCAAG 109
GACGGAAAGACCCCGTGAACC 15
TCCGAGTGTAGAGGTGAAATT 467
TGGCCCTTACGGGCTGGGCTA 29
GTCGACCGAATACCCCCGGGG 5187
ACGATCCATAGCTGGTCTGAG 30
TTGGACAATGGGCGCAAGCCT 1709
ATCCTGGCTCAGGACGAACGC 11
TCATGTTGGTGTTGAGACGGA 22
CAGAACGTCGTGAGACAGTTA 12
GGGATGGACGTATCTCTGGTG 736
AGCTCAGCTGGGAGAGCACCT 2622
GGAACTGCCTTTGATACTGGA 161
GACATCAAACGAGACTCCGCT 335
TCCAAAAGCCATCTCAGTTCG 62
TTTGAGAGGATGATCAGCCAC 17
AAGGCGCTGTGCTAACCGCAA 18
CTGCCCGGTGCTGGAAGGTTA 14
AAAGCGAGTCTGAACAGGGCG 17
GAATTCCGAGTGTAGAGGTGA 268
CGACCTGGTGGTTATGGCGGC 10
CTGGCTCAGAACGAACGCTGG 3373
GGCTAGCGTTGTTCGGATTTA 123
ACAGGGCGTTCAGTTCGATGC 364
TCGGCCCATGTGGGCCGCCCC 20

CTGGGCTACACACGTGCTACT 42
GAACTCGGCAAATTGCACGCG 14
TATCCTTCAGTTAGGCTGGAC 12
TGGGCACTCTAAGGGGACTGC 977
TGGGCCGTGAAACGCTCCAGC 14
GGCCGTAGCTCAGCTGGGAGA 10311
GGTGACAGTGGGCAGCGAGCA 308
TTAAACATTCCGCCTGGGGAC 33
TTAGCCGTCGGGGTGTTTACA 183
GTGAAACGCTCCAGCGCCAAT 1072
ACCGTCTTACTGATCCTAACC 163
GGGTGAGTAACGCGTGGGAAC 18
TGACATCCCGGTCGCGGTTAG 156
ACCACGTTAATATTCGTGGGC 10
TATGTGCGTGGTAGCGGAGCG 27
GAACCTTACCAGCCCTTGACA 387
CGGAGGCGCGCGATGGTAGGG 27
GGTGCGAAAGCGTGGGGAGCA 10
AGCAGTGGGGAATTTTGGACA 49
TGGTTTTACCCGAAGGCGCTG 201
TTAGAACATAGATCGCAGGCC 29
CAAGGCGACGATCCATAGCTG 194
TAGATACCCTGGTAGTCCACG 277
CTCACTGGACCATTACTGACC 149
GCAGAAGTCCTTGAGTAGGGC 744
GAGGGGCCGTAGCTCAGCTGG 143
GGAAGTGAGAATGCTGACATG 103
AGAAGAAGCCCCGGCTAACTT 21
AAGGGAGTGAGAGACTCCCTC 41
AAGGTCCCTAAGTTATGGCTA 178
ATGGTTGTCGTCAGCTCGTGT 24
CGACTGTTTAGCAAAAACACA 13
ATGGCTAAGTGGGAAAGGATG 127
GTATCTCGAGAAGCTGGTCTT 11
CAATGAGAGTGATCAAGTGTC 12218
ACGGCCCAGACTCCTACGGGA 6099
CCTGTCTGAACATGGGTCGAC 2168
GTGAGAATGCTGACATGAGTA 335
AAGCTGTGGATGCACGTATGT 41
GTGAGTTGGGTTCAGAACGTC 18
TCGACTCATCGCATCCTGGGG 482
CAACCCACCTTAGATGACTAG 33
ACCTGGGGAACTGAAACATCG 17
CGAAAGTCGGTCATAGTGATC 142
TAGGCTCAGAACGGTCGGAAA 248
CGGCTCATCGCATCCTGGGGC 16
GGCACAGACCAGGGGGTAGCG 12
GGGAGTACGGTCGCAAGATTA 102
TAGAAGCGAACCTGGGGAACG 21
TAGAGCACTGGATGGGCTATG 23
TTACTGGGCGTAAAGCGCACG 1941
GTGGGTGTAGGAATATTGACA 65
AGGACGTGATACGCTGCGATA 31
CGGAAAGACCCCGTGCACCTT 101

CCGTGAAACGCTCCAGCGCCA 11395
TTAGATACCCTGGTAGTCCAC 238
ACAAAGGCAAAGAACAGGCGC 46
GGCTAAGTGGGAAAGGATGTG 126
GCGAAGGCGGCTCACTGGACC 650
GCAGGTTGAAGGTACGGTAAC 932
GCCATGAGCAGGTTGAAGGTA 436
TAAACATTCCGCCTGGGGAGT 377
GGCTGCACCCGATCCCATTCC 14
ACGGCCCAGACTCCTACGGGG 15
GTTCGGATTTACTGGGCGTAA 44
CGTACCCTAAACCGACACTGG 639
ACTGGAGGACCGAACCCATAT 429
AGTTGGTTTTACCCGAAGGCG 34
CTCACTGGACCATTACTGACT 30
CAGAAGTCCTTGAGTAGGGCG 739
GGCATAAGCCTGCCTGACTGC 293
CTCAAGACGCGGGAGAGTAGG 239
ACGGAATAACTCAGGGAAACT 246
GGACTCACCGTCTTACTGATC 469
TTACTGGGCGTAAAGCGCACA 14
CCTTGAGTAGGGCGGGACACG 20
TGACGCCTGCCCGGTGCTGGA 526
CCGTGAAACGCTCCAGCGCCG 23
TTAAGAGCTGAGTTTTGATGG 265
CGTGAGTTGGGTTCAGAACGT 21
ATCCAGCCATGCCGCGTGAGT 12
ACCTGGGGAACTGAAACATCA 130
AGAATAGCAGAAGTCCTTGAG 472
AACAACCCTGACCACCATCTA 15
TGAAACTCAAAGGAATTGACG 11
AAGCCCCGGCTAACTTCGTGC 147
TAGCTTTACACTGGCATTCGT 88
AGAGATTTTGGACGGTTTAGA 15
CCCGCGTTGGATTAGCTAGTT 47
ATTTGGTTGCGGGGGCAGGAT 31
AGGATGTTGGCTTAGAAGCAG 891
GTGCATGACCGATAGCGAACC 288
ATGATCCGCCACACTGGGACT 25
GATACCCTGGTAGTCCACGCT 23
CGGAGCGGCTGCACCCGATCC 1950
CGGCCCAGACTCCTACGGGAC 49
ACGAAAGTCGGCCATAGTGAT 10
GCGGTAATACGAAGGGGGCTA 205
AGGAATTGACGGGGGCCCGCA 16
CTGAAGGCCGCAGGTTCAAAT 17
TTAAGTCCCGCAACGAGCGCA 32
GACAAGTCGAGCAGAGACGAA 16
CCATTACTGACGCTGAGGTGC 15363
AGGCGCTGTGCTAACCGCAAG 26
CGGAGAAGAAGCCCCGGCTAA 96
TTAACACATGCAAGTCGAGCG 29
GCTAGTAATCGCGGATCAGCA 146
CTCCAAAAGCCATCTCAGTTC 55

GCAAGCCTGATCCAGCCATGC 221
GGAGTGAGAGACTCCCTCGCC 41
GGTGAGTAACGCGTGGGAACG 18
TAGAGCATACCAAGGCGCTTG 10
GCGACTGGGGTGAAGTCGTAA 12
ACCGGGGAGTACTAGTCGGCA 808
TAGCTCGATCGACTTGATCAC 41
TAGGTGTAGGCGCAGCGAAAG 26
GACCAGACTCCTACGGGAGGC 15
AACGCATGAAGCTTACCGGTA 61
CGGTTAGTGGAGACACTATCC 40
AAGCCATCTCAGTTCGGATTG 98
CCTGGCTCAGGACGAACGCTG 13
GGTGCTGGAAGGTTAAGAGGA 15
CGTGCATGACCGATAGCGAAC 316
GGGCTGGGCTACACACGTGGT 28
GTTGGGTTAAGTCCCGCAACG 24
ACAGGATTAGATACCCTGGTA 34
CTGAAACCGGATGCCTACAAA 147
AACGCATTAAACATTCCGCCT 459
GATACCCTGGTAGTCCACGCC 363
GCCGCGGTGAATACGTTCCCG 197
AAAGTCCAAGGGTTCCTGCTT 15
CTTCGTGCCAGCAGCCGCGGT 23
AAGTCGGAATCGCTAGTAATC 290
AGAAGCCCCGGCTAACTTCGT 62
TAAGTGGGAAAGGATGTGAGG 101
GTTTGAGAGGATGATCAGCCA 12
CGTGAGACATCCTGGAGGTAT 111
GCCGTAGCTCAGCTGGGAGAC 15
TGGTTGCGGGGGCAGGATTTG 31
GAGAGGATGATCAGTCACACT 14
GGAAAACTCGACCGAAGTGGG 17
GAAGCGTGACCTCACTATGGG 192
ATCTGTTGCAATAGATCGGGA 27
AACCTGCGGCTGGATCACCTC 31
ACGCTGGCGGCAGGCTTAACC 27
ACGTAGAATAGCAGAAGTCCT 1633
ATGACCCCCAAGAGTCCATAT 12
GGGTTCAGAACGTCGTGAGAC 141
CAATGGTACTTCGTCTCAAGA 21
TGCAGGTGGTGACGGATCGCG 75
TCGGATCAACTGAAGAGTTGA 14
TGAAGGCCCTAGGGTTGTAAA 1509
TCTAAGGTCCCTAAGTTATGG 381
GCAAGACGACGTATAGGGTCT 37
TGAAGGAACTCGGCAAATTGC 10
GGGTTAGAAGCGAACCTGGGG 812
CGGTCCCTATCTGCCGTGGGT 1339
AACTGGAGAGTTTGATCCTGG 12
ATCTCCAAAAGCCATCTCAGT 10
GGCGGACTTTTAAGTCAGGGG 68
TCGAGAAGCTGGTCTTTCTGC 306
CCGGTACTAATAGCTCGATCG 5477

GCCGACCTGGTGGTTATGGCG 11
ACTGGACCATTACTGACGCTC 10
ACCTGCACGAATGGCGTAACG 100
GGTACTTCGTCTCAAGACGCG 109
CGGTCGCGGTTAGTGGAGACC 11
AGAAGCGAACCTGGGGAACGG 14
TCCTTGTCGGGTAAGTTCCGA 225
ACTTGATGAGGGGCCGTAGCT 170
AAGGGGGCTAGCGTTGTTCGG 676
CAACGGATAAAAGGTACTCCG 39
GAGTAGGTCGCTGCCAGGTCG 18
TAAAGCGCACGTAGGCGGACC 12
TTAAGTCAGGGGTGAAATCCC 23
ATACCCTTTGATCCGACGATC 14
CCATGAGCAGGTTGAAGGTAC 423
CATGTGTAGGATAGGTGGTAG 971
AGCAGAGACGAAAGTCGGTCA 35
GAGATAGCTGGTTCTCCGCGA 23
GAGGGGAGTGAAACAGTACCT 18
GAAATCCTGTCTGAACATGGG 90
GATAGTCCCGTACACGTAGAA 63
CGGCCGTGAAACGTTCCAGCG 14
CCTGGTAGTCCACGCTGTAAA 25
TGAGTAGTTGGGGGTGGTTTT 11
CCATTACTGACGCTGAGGGGC 88
GACTGGGGTGAAGTCGTAACA 20
GACGGTAACCGGAGAAGAAGC 170
GGTTAGCCGGCCCCTAAGGCC 11
GGGCTTGTAGCTCAGTTGGTT 117
GTGAATACGTTCCCGGGCCTT 166
CGTACGGCGCGTGAGCGAGAA 13
AGGGTTGTAAAGCTCTTTCAC 1626
TTGAAGCAGGGGCGCCAGCCT 262
TACCAAGGCGCTTGAGAGAAC 98
CCGGGGATAACAGGCTGATGA 10
AACGACTTCCCCGCTGTCTCC 74
ATCGCTCAACGGATAAAAGGT 135
GCGTACCTTTTGTATAATGGG 11
TATAATGGGTCAGCGACTTAT 16
AACTGCGTTGAAGGAACTCGG 1123
GGGGGTCGTCGGTTCGATCCC 512
GCGGGGTTCCTGCGGTTAGAC 665
TAAAGCGCACGTAGGCGGACT 247
GTAGAGGTGAAATTCGTAGAT 182
ATACCCTTTGATCCGACGATT 1859
GGGCAACCCACCTTAGATGAC 26
CGGTCCCTATCTGCCGTGGGC 13
CACTGGTCTAAATAAGGGTCT 12
ATTGGACAATGGGCGAAAGCC 36
CCTGCCTGACTGCAAGACTGA 84
TCTCATGTTTGTGTTCTTCGC 21
ACTGGATGGGCTATGGGGACT 3002
GGCGAAGGCGGCTCACTGGAC 430
GATAAAAGGTACGCCGGGGAA 10

TCTGAGAGGATGATCAGTCAC 12
ACTGGACCATTACTGACGCTT 11
ATCGTGAAGAGAAGATGTAAT 25
CGGGCTGGGCCACACACGTGC 13
TTTGATACTGGAAGTCTTGAG 33
CGAAAGCGTGGGGAGCAAACA 12
TTCCCTATCAGAGCCGTGGAC 11
CTAGGTCGTCGGCCCATGTGG 21
CTAAGGGGACTGCCGGTGATA 163
GGCTGGACCGGAGACAGGTGA 11
AGGATCCCAAAACAACCAGGA 70
GGGCCGTACCGCAGCTGACGC 93
AATAGATCGGGATGACTTGTG 173
GCACACGCTTGATAAGCGTGG 11
GAACTGCGTTGAAGGAACTCA 10
CGGGGAGTACTAGTCGGCAGA 335
GGAATTCCGAGTGTAGAGGTG 404
CGAGAGGACCGGGATGGACGG 19
GTCCTCATGGCCCTTACGGGC 118
GCAGCTTACCGGTACTAATAG 45
GTCGTGAGACATCCTGGAGGT 27
GTCGGGGTGTTTACACTTCGG 66
AGGCGGAAGAGATTTTGGACG 17
TTCGAAGCAACGCGCAGAACC 70
GAAACCAAGTGATCTAGCCAT 11
ATAAAGTGGAACGAGTTGGAA 10
GGGCGGCATTGTCTGCGGATC 16
CGTCCGTCGTGGAGAGGGCAA 38
GGCCGGGAAACGCTCCAGCGC 19
GTGGTTGACAGGTTGGTTTGA 10
CGGTTAGACGGAAAGACCCCG 49
AGTGATGAAGGCCCTAGGGTT 34
TCCCGCAACGAGCGCAACCCT 95
CTTACCGGTACTAATAGCTCG 265
GGGGAACCTGCGGCTGGATCA 43
GGACCGGGATGGACGTATCTC 9421
GGTACCCCGGGGATAACAGGC 59
CAGGATGTTGGCTTAGAAGCA 1811
GTCGGGGAGGTGCGAATACCC 313
TAGCAGAAGTCCTTGAGTAGG 86
AAGCGAACCTGGGGAACTGAC 74
GGTCGTCGGCCCATGTGGGCC 17
TTAATTCGAAGCAACGCGCAG 25
CCCACCTGAAAACGAGTATTC 10
ACCTTACCAGCCCTTGACATC 133
GGAGGCAGGCGACCACGGTAG 139
GCCGTAGGGGAACCTGCGGCT 29
CGTAGGGGAACCTGCGGCTGG 29
TTGGTGGATGCCTTGGCATGC 47
GTACCGGGGCTCAAGCCATAC 808
ACGGAGGCGCGCGATGGTAGC 18
CATTGGGACTGAGACACGGCC 42
ACTATAGCTTTACACTGGCAT 15
TGGTGGATGCCTTGGCATGCA 37

GCGGGAGAGGAGGTCGCTGCC 13
GTAAAGCTCTTTCACCGGTGG 12
AGAGAACTGCGTTGAAGGAAG 10
AAGGAATTGACGGGGGCCCGC 24
GGATTGCACTCTGCAACTCGA 334
GGTGAAAAGCACCCCGACGAG 35
AGGGCGGCGGCGCCGGCAGCG 41
CCATTACTGACGCTGGGGTGC 16
TCTCTTTCTTCATTGTTGATT 22
GGTAGTTTGACTGGGGCGGTC 156
GTAATCGCGGATCAGAATGCC 11
CCGCAAGGGGAGCGGCAGACG 51
GAACTGCGTTGAAGGAACTCG 1101
CTGTCCCTAGTACGAGAGGAC 99
CCGGAACTGCCTTTGATACTG 159
CGAGAGGACCGGGATGGACGA 32
GCGTTCCGTAAGCCTGTGAAG 17
ACTCGGCCGGGAAACGCTCCA 18
GATAGCTGGTTCTCCGCGAAA 93
GGTAGTTTGACTGGGGTGGTC 13
GGGGCTGTAGCTCAGCTGGGA 412
GACGTATAGGGCCTGACGCCT 12
GACTTGTGGCTAGGGGTGAAA 921
CAAGACGCGGGAGAGTAGGTC 334
GTAATCGGATCAACTGAAGAG 18
GTAGGTGTAGGCGCAGCGAAA 35
CGCCTGGGGAGTACGGTCGCC 12
GTAAAGCTCTTTCACCGGTGA 994
GTCAGCGACTGGGGTGAAGTC 411
AGCCCAAGGTTTGTCCTGGGT 14
AGAGAACTGCGTTGAAGGAAA 30
AAGCAGGGGGTCGTCGGTTCC 24
CGGCCGTGAAACGCTCCAGCG 35314
AAGCGAACCTGGGGAACTGAT 17
TAAGTCCCGCAACGAGCGCAA 79
ATGTGAGGATCCCAAAACAAC 24
GGCTGTTCGCCATTTAAAGCG 17
AGAGTGATCAAGTGTCTTAAG 213
GCGGGTAGTTTGACTGGGGCG 12
GGCCCGCGTTGGATTAGCTAG 104
GGGGAGTTTGACTGGGGCGGT 192
CCACCTTAGATGACTAGAAAA 69
ATCGGCCCGCGTTGGATTAGC 270
TACGCTGCGATAAGCGTCGGG 56
TGGCGTAACGACTTCCCCGCT 13
AATCGCGGATCAGCATGTCGC 20
ATTTCCGAATGGGGCAACCCA 10
CGACCACGGTAGGGTCAGCGA 2121
TGCCGCGGTGAATACGTTCCC 212
CGTACCTTTTGTATAATGGGT 14
ATAGCTGGTTCTCCGCGAAAT 254
TGCATATAGACCGTACCCTAA 182
GTGACGACATGTGTAGGATAG 154
CAACCAGGATGTTGGCTTAGA 19

AGTGGCGAAGGCGGCTCACTG 301
ATGATCGGCCCGCGTTGGATT 365
AGCTGAGTTTTGATGGATATT 262
TCAGTTAGGCTGGACCGGAGA 41
ACAATGGGCGCAAGCCTGATC 194
TGGTGACAGTGGGCAGCGAGC 202
GGCCTTAGGGTTGTAAAGCTC 29
GGCGGAAGAGATTTTGGACGG 15
GGGTAGAGCACTGGATGGGCT 39
CGGAGGCGCGCGATGGTAGCC 12
AACTGCCTTTGATACTGGAAG 52
TAGCGAACCAGTACCGTGAGG 99
GGAATAACTCAGGGAAACTTG 88
ATGGGGGTGCGACTGATTATA 40
AAGTCCTTGAGTAGGGCGGGA 746
GGAGGCGCGCGACGGTAGGCT 10
GATAGCGAACCAGTACCGTGA 118
CGACGTTCTGAACCCAACTCA 10
TTGACTGGGGCGGTCTCCTCC 26
AGGGGGTAGCGACTGTTTACA 27
ATGCCGCGTGAGTGATGAAGG 24
TGCCCGGTGCTGGAAGGTTAA 16
GGACCGAACCCATATCTGTTG 20
CGTAGATATTCGGAGGAACCC 22
TCCTGGCTCAGAGCGAACGCT 10
GTCGACCACGATCCAAGCCTA 18
TCGGAAGAACACCAGTGGCGA 43
ACCTGGTGGTTATGGCGGAGC 4741
TACTGGAGGACCGAACCCATA 2092
TTACCGGTACTAATAGCTCGA 141
GAGCATACCAAGGCGCTTGAG 10
GATGAAGGCCTTAGGGTTGTA 12
GGACACGTTTCTTGGTAAGAA 57
AAACTTGGAGATAGCTGGTTC 158
AGCGTAACAGCTCACTGGTCT 301
GGAGGAACACCAGTGGCGAAG 1536
TGAGAGACTCCCTCGCCGAAA 24
GAGGCGCGCGATGGTAGACTC 15
CGACCACGATCCAAGCCTAAG 34
TCGTGGAGAGGGCAACAACCC 11
GATACGGCCCAGACTCCTACG 15
ATCTGAGCAGGGTTAGCCGGC 496
GAAGGGCCATCGTTCAACGGA 55
CTGGGTTCAGAACGTCGTGAG 29
ATGAAGTTGGAATCGCTAGTA 100
TACCTGAAACCGGATGCCTAA 12
GGGGCTCAAGCCATACACCGA 1292
GGTTGCGGGGGCAGGATTTGA 28
GGGGTTAGAAGCGAACCTGGG 805
CGAGGCGCTGACACGGATTTG 84
ACTTTGAAGCAGGGGCGCCAG 596
AAACCGACACTGGTGGACTGG 22
CCTGCAGGTGGTGACGGATCG 113
CCGGGTGTGGAAGTGCGGCAA 503

CTGGTAGTCCACGCCGTAAAC 196
TTGAGTATGGTAGAGGTGAGT 29
TCAAGACGCGGGAGAGTAGGT 309
TGCAAGCAGGGGGTCGTCGGT 90
ATAGTGAACCAGTACCGTGAG 33
AGGCGCTTGAGAGAACTGCGT 60
GTAGTGGCGAGCGAACGCGGA 81
CAAAGGCAAAGAACAGGCGCA 41
CTGACGCTGCTGGCCCTGCGC 25
CCGCAGACGAGGCGCTGACAC 14
ATTCCTTGTCGGGTAAGTTCC 294
CCTGGTAGTCCACGCCGTAAA 201
TCGTGCCAGCAGCCGCGGTAA 20
CTTCCCCGCTGTCTCCAACGC 424
GTAGAGCGTCGACCGAATACC 1563
GCTTGTAGCTCAGTTGGTTAG 123
CCGGGGGTAGAGCACTGGATG 190
ACACACGTGCTACAATGGTCG 10
CTGTCGTCAGCTCGTGTCGTG 77
CTGATGACCCCCAAGAGTCCT 13
AGCGGGAAACCCACCTGAAAA 13
CTCGATGTCGACTCATCGCAT 27
GATTGCACTCTGCAACTCGAG 336
GTCCACGCCGTAAACGATGAA 32
AGAAGCTGGTCTTTCTGCTGA 13
TCGCAGGCCAGTCAGCCTGAC 10
ACGCATGAAGCTTACCGGTAC 62
ACAGGATCTGTCCCTAGTACG 214
TATAGCTTTACACTGGCATTC 13
ATGAATGTTAGCCGTCGGGGG 86
GCGGGGGCAGGATTTGAACCT 14
CTTGACATCCCGGTCGCGGTT 221
GACTCATCGCATCCTGGGGCT 440
ATTCCGCCTGGGGAGTACGGT 2328
ATACCCTGGTAGTCCACGCCG 277
AGCTCGTGTCGTGAGATGTTG 218
GAATACCAAGGCGCTTGAGAG 58
GAGATACGGCCCAGACTCCTA 12
GCGGGATGGAGCAGCCCGGTA 15
AGCGAACCTGGGGAACTGAAG 33
GTAGTCCACGCCGTAAACGAT 55
CCTGTGAAGGGACAGTCGTGA 45
GTAGCGACTGTTTACCAAAAA 12
GCCCCGGCTAACTTCGTGCCA 80
ACGGACGGGATAACCGCTGAC 27
ATCAGAGCCGTGGAAGACCAC 16
TGCCTGACTGCAAGACTGACA 78
ATTAAAACTCAAAGGAATTGA 56
GCCGTGAAACGCTCCAGCGAC 17
CATCTAAGGTCCCTAAGTTAT 12
CAGACTCAGTGAAATTGAATT 80
AGATTAAAACTCAAAGGAATT 72
GACCACCACGTTGATAGGCCG 127
TCTCGAGAAGCTGGTCTTTCT 241

CTGACATGAGTAACGATAAAG 16
CGGATAAAAGGTACCCCGGGG 79
TATCGGCAAATGATCGGCCCG 218
AACCAAACTCCGAATACCGGG 38
GTAGCTCAGCTGGGAGAGCGC 19
ACAGGATCTGTCCCTAGTACA 16
CTGCACGAATGGCGTAACGAC 41
ATACATAGGGGTTAGAAGCGA 11
CAATGGTGGTGACAGTGGGCA 96
TTGGCGGACACGTTTCTTGGT 121
GCAACAACCCTGACCACCATC 49
AACGTCCGTCGTGGAGAGGGC 195
TGCTACGGAATAACTCAGGGA 273
AACTCGGCCGTGAAACGCTCC 6123
GCAAAATGCACGCGTAACTTC 30
CTTGAGAGTTTGATCCTGGCG 20
ACATCTAAGTACCCAGAGGAA 180
CGGGCTGGGCTATACACGTGC 19
CCTTAGATGACTAGAAAATCT 19
ACGTACTGGAGGACCGAACAC 11
ACGCATTAAACATTCCGCCTG 1950
GGTTAGGCGGAAGAGATTTTG 11
CTGATGACCCCCAAGAGTCCC 39
AGCGAAATTCCTTGTCGGGTA 1171
GATTTCCGAATGGGGAAACCC 19
TTCTAAGGAAGATCGAGAATT 40
TAGAATAGCAGAAGTCCTTGA 1147
AGAGGGCAACAACCCTGACCA 19
ATCGGCAAATGATCGGCCCGC 1555
CGTGACCTCACTATGGGCAAC 15
CGGTCGCAAGATTAAAACTCA 171
GATGTCGGCTCATCGCATCCT 13
GCACAGACCAGGGGGTAGCGA 40
TGGCATTCGTGACGACATGTG 11
TCTCCAAAAGCCATCTCAGTT 54
AGCGAACGCGGACCAGGCCAG 1666
CGGGGCTCAAGCCATACACCG 1554
AGCGAACCTGGGGAACTGAAA 17565
CGTCGTGGAGAGGGCAACAAC 22
GGCACTCTAAGGGGACTGCCG 252
AGTCGAGCGCCCCGCAAGGGG 62
CTCGGCCGTGAAACGCTCCAC 17
GGAGCCCAAGGTTTGTCCTGG 56
TTCAGTTGGGCACTCTAAGGG 133
CAAACTCCGAATACCGGGGAG 15
AGGATGATCAGCCACACTGGG 1863
TAACCGCTGAAGGCATCTAAG 15
CTTCGGGGGAAAGATTTATCG 23
GGGGTTCCTGCGGTTAGACGG 468
ACAAGGTAGCCGTAGGGGAAC 28
TAAGTACCCAGAGGAAAGGAC 757
GTACCCTAAACCGACACTGGT 479
ACCAAGGCGACGATCCATAGC 340
GGGCTGGGCTACACACGTGCA 29

CGTAAACGATGAATGTTAGCC 235
GCGAACCTGGGGAACTGAAGC 23
AATTCCTTGTCGGGTAAGTTC 171
AGAGCACCTGCTTTGCAAGCA 10
TGTTCGGATTTACTGGGCGTA 150
TAGATATTCGGAGGAACACCT 35
AGTACCTGAAACCGGATGCCT 6527
ACCTCGATGTCGACTCATCGC 28
ACGGTCGGAAATCGTTCGTCG 298
GGGACTCACCGTCTTACTGAT 458
TTACACACCAACTTCGATCCG 14
CAAGAGTCCATATCGACGGGG 14
GTTCAAATCCTGCCCCCGCAA 20
CTAACTTCGTGCCAGCAGCCG 16
GTCAAGTCCTCATGGCCCTTA 59
ACCCTAAACCGACACTGGTGG 546
GGAGTACGGTCGCAAGATTAA 126
TGTCGGGTAAGTTCCGACCTG 60
TTCGATCCCGTCCGGCTCCAC 1686
AAGAGAAACGTGGGCGGCATT 12
GAATGTTAGCCGTCGGGGTGT 445
GCGAAAGCGAGTCTGAACAGG 21
TAGAGGTGAAATTCGTAGATA 97
CTTTCACCGGTGAAGATAATG 12
TACCAAAAACACAGGGCTCTG 16
AGAGGTGAAATTCGTAGATAT 72
ATCGAGAATTGGAAAGAGGCA 14
GTCAGCGACTTAGTGTATCGA 163
AGTCCTCATGGCCCTTACGGG 116
ATGTTAGCCGTCGGGGTGTTT 66
CGGAAATCGTTCGTCGAGTGC 90
CGTAGATATTCGGAAGAACAC 43
TAGATATTCGGAGGAACACCC 35
TTTAGGTAGAGCGTCGACCGA 136
AAACGGCGGCCGTAACAATAA 11
GGACCATTACTAACGCTGAGG 10
TACTAGTCGGCAGACACACGG 15
TGAATGTTAGCCGTCGGGGGG 25
CTGGATGGGCTATGGGGACTC 2959
GGGGTGGAGCAGCCCGGTAGC 208
GTCCCTATCTGCCGTGGGTGT 256
GACTTAGTGTATCGAGCAAGC 108
GGGCTGGGCTACACACGTGCG 155
CCACCTGAAAACGAGTATTCC 10
TACGGAATAACTCAGGGAAAC 159
GGAGACACTATCCTTCAGTTA 13
ACCTGCGGCTGGATCACCTCC 126
TGAAGCAGGGGCGCCAGCCTT 92
TAGCGACTGTTTACCAAAAAC 17
TCGGCCGTGAAACGTTCCAGC 14
CGAAGTGGGTGATAGTCCCGT 297
CGGAGGCGCGCGATGGGAGGC 11
GAGCAGAGACGAAAGTCGGTC 126
CGATCCATAGCTGGTCTGAGA 14

CTATTTAGGTAGAGCGTCGAC 25
GCGGACCAGGCCAGTGGCTTT 884
AGATCGCAGGCCAGTCAGCCT 69
TCGGCAAAATGCACGCGTAAC 59
CCACGTTGATAGGCCGGGTGT 18
GTGGCTAGGGGTGAAAGGCCA 273
CTGGGCTACACACGTGATACA 12
AATGCGTACGGCGCGTGAGCG 11
ACGCTGCGATAAGCGTCGGGG 90
CGAAGGGGGCTAGCGTTGTTC 561
AGCTGGTTCTCCGCGAAATCT 235
TGAAATTGAATTCCCCGTGAA 10
AAGGAGGCAGGCGACCACGGT 89
GAACTCGGCAAAATGCACGCG 452
TAGTAATCGCGGATCAGAATG 11
TCGTGCAGGTCGGAACTTACC 11
TTAGCCGGCCCCTAAGGCGAG 41
GGGGCCATAGCTCAGCTGGGA 1434
CTCGGCAAATTGCACGCGTAA 10
CATTACTGACGCTGAGGCGCG 18
AACCCACCTTAGATGACTAGA 40
GCATTGTCTGCGGATGGTTCG 38
TTTGTTGGAGCAACGCTGGAT 47
CGAGAGGACCGGGATGGACGT 4691
TATATACGGACGGGATAACCG 85
AGTGAACCAGTACCGTGAGGG 36
AGAACCTTACCAGCCCTTGAC 916
GGGCTATGGGGACTCACCGTC 1406
CGCGTAGAACCTTACCAGCCC 12
CGGGGAGTACTAGTCGGCAGC 18
TTATGGATGTCTAACTGCGGC 52
CCAGGGGGTAGCGACTGTTTA 1922
ACGTAGGCGGACTTTTAAGTC 50
GGTGTGGAAGTGCGGCAACGC 255
AAGCCTGCCTGACTGCAAGAC 256
AGGTCGTCGGCCCATGTGGGC 19
TCGGCCCGCGTTGGATTAGCT 465
ACATTGGGACTGAGACACGGC 43
AGTAGTGGCGAGCGAACGCGG 68
CGTCGGGGAGGTGCGAATACC 515
GGGCGGCATTGTCTGCGGATA 17
GATAAAAGGTACCCCGGGGAT 71
TCTGCAACTCGAGTGCATGAA 15
TACTGATGAAAATGGATTGAC 10
CCAGTCAGCCTGACGATCGCT 37
ACGGGCTAGGCTACACACGTG 15
AATGGGCGCAAGCCTGATCCA 103
CTCGTGTCGTGAGATGTTGGG 255
GCTGTGGATGCACGTATGTGC 358
CGGCCGTGAAACGCTCCAGCC 14
ACCAGCCCTTGACATCCCGGT 91
GTCGGTTCGATCCCGTCCGGC 533
AAGCGAACCTGGGGAACTGAA 7005
AAAGACCCCGTGCACCTTTAC 44

AAGCAGGGGGTCGTCGGTTCG 359
TTTCTAAGGAAGATCGAGAAT 12
CCGGTCGCGGTTAGTGGAGAC 221
CGTAGTCGATGGGAACCACGT 60
ACGGAGGCGCGCGATGGTAGA 43
TGGGGTAAAGGCTCACCAAGG 26
AAAGGCGTAGTCGATGGGAAC 270
CGTATGTGCCCTTCGGGGGAA 49
GCAGACGAGGCGCTGACACGG 15
GTGTAGAGGTGAAATTCGTAG 108
GTCCCTAGTACGAGAGGACCG 42
GGGCGGCATTGTCTGCGGATG 3407
TCAAATGAATTGACGGGGGCC 17
TGAGTGGAATTCCGAGTGTAG 283
ACTCTTGGGGGTCATCAGCCT 20
TTGTAAAGCTCTTTCACCGGT 786
GAAGTGAGAATGCTGACATGA 111
TTTCTCTTTCTTCATTGTTGA 20
CGCAGAACCTTACCAGCCCTT 3866
GCCTTGGCATGCACAGGCGAT 159
CTTAGGGTTGTAAAGCTCTTT 14
CACGCACTGGAGGACCGAACC 30
GTGCGAATACCCTTTGATCCG 20
ATCTGTCCCTAGTACGAGAGG 80
AGACATCCTGGAGGTATCGGA 117
TCCGCCACACTGGGACTGAGA 20
AAAGGTACTCCGGGGATAACA 80
CATAGATCGCAGGCCAGTCAG 224
AGAGAACTGCGTTGAAGGAAC 4416
CAATAACGGTCCTAAGGTAGC 21
GGACGGGATAACCGCTGAAGG 2330
GGGAACCACGTTAATATTCGT 23
CGCCTGGGGAGTACGGTCGCA 2767
ACGGAGGCGCGCGATGGTAGG 37882
TAATCTGAGCAGGGTTAGCCG 119
ATCGAAGTTGGTGTGTAAATA 16
GACTTGTGGCTAGGGGTGAAC 25
GGGCGCCAGCCTTTGTGGAGT 10
GTATAGGGTGTGACGCCTGCC 12
GGCATCTAAGCGGGAAACCCA 50
ATCGACTTGATCACTCCCATT 609
GAACTTTGGCGGACACGTTTC 19
TGTGTAGGATAGGTGGTAGAC 996
CTATCTGCCGTGGGTGTAGGA 114
AAGCGAACCTGGGGAACTGAG 45
CGCCATCACCGATTGTATCTC 16
TTGTTCGGATTTACTGGGCGT 191
GACTCCGCTAGTAGTGGCGAG 12
GAAAGACCCCGTGCACCTTTA 43
TCTGACGCCTGCCCGGTGCTG 1211
CCAAAAGCCATCTCAGTTCGG 70
ACTTTGGTTTTCGGATCGAAG 21
AACTCGGCAAATTGCACGCGT 14
GACGGGGGCCCGCACAAGCGG 184

AGTTGGAATCGCTAGTAATCG 366
TGAGGATCCCAAAACAACCAG 29
GGGACTGCCGGTGATAAGCCA 53
TGGAGCAGCCCGGTAGCTCGT 186
CGACGATTTCCGAATGGGGCA 31
ATACGTTCCCGGGCCTTGTAC 180
GGTCGGAGGTTCAAGTCCTCC 30
CTGGAGGACCGAACCCATATC 406
CGATAAAGGGAGTGAGAGACT 659
TAAAAGGTACTCCGGGGATAA 64
CCCGAAGGCGCTGTGCTAACC 225
TTGATTTGTGAGTAGTTGGGG 11
GGCAAATGATCGGCCCGCGTT 2423
GGAGGAACACCAGTGGCGAAC 28
CGAACCAGTACCGTGAGGGAA 144
GCGTTGTTCGGATTTACTGGG 348
GTGCTAACGTCCGTCGTGGAG 32
AATGACGGTAACCGGAGAAGA 34
AGAGGATGATCCGCCACACTG 16
GGCCGTAAAACGCTCCAGCGC 12
GTTTGGCACCTCGATGTCGAC 145
ACACGCTTGATAAGCGTGGGG 16
TGCTGACATGAGTAACGATAA 12
CATGAGCAGGTTGAAGGTACG 393
AGGCTCACCAAGGCGACGATC 76
AGATATTCGGAGGAACACCCG 11
GTACGGCGCGTGAGCGAGAAC 10
GAACTCGGCCGTGAAACGCCC 11
GACTGTTTACCAAAAACACAG 15
GAAGGCCTTAGGGTTGTAAAG 16
TCGTGGGCCTGCAGGTGGTGA 513
GAATATTGACAGGATCTGTCC 150
TCGAAGCAACGCGCAGAACCT 27
GGGGTAAAGGCTCACCAAGGC 31
GAAGGACGTGATACGCTGCGA 53
CTTTGCAGACCTGGCAGCGAC 33
AGGATGTGAGGATCCCAAAAC 24
CCGATAGCGAACCAGTACCGT 157
TCGGCCGTAAAACGCTCCAGC 14
AGGGGGTAGCGACTGTTTACC 1741
ACTCTAAGGGGACTGCCGGTG 195
AAATGCACGCGTAACTTCGGA 89
CTGCCGGTGATAAGCCGAGAG 63
ACTGATCTAGAAGCCCGGCAC 54
CAGCCCTTGACATCCCGGTCG 84
ACGGTAACCGGAGAAGAAGCC 1143
GCCCCTAAGGCGAGGCCGAAA 13
TTAGGCTGGACCGGAGACAGG 545
ATCCCGGGGCTGGAGCAGGTC 10
TGAGAGACTCCCTCGCCGAAC 11
CGGGGTTGTTTGGCACCTCGA 191
TGTCGGCTCATCGCATCCTGG 15
AGAACTGAAACATCTAAGTAC 19
TGTTTAGCAAAAACACAGGGC 11

GGTAAGTTCCGACCTGCACGA 12
AGAAGTCCTTGAGTAGGGCGG 746
ACCGAAGTGGGTGATAGTCCC 331
TGTAGAGGTGAAATTCGTAGA 116
GGGACTGCCGGTGATAAGCCG 3083
GGGCAGTTTGACTGGGGCGGT 14
CGCGTGAGTGATGAAGGCCCT 17
CTAACTGCGGCCCGTTATCCG 11
TCGAGCGCCCCGCAAGGGGAG 73
GCGGCGGCGCCGGCAGCGGCA 31
TCAAGCCATACACCGAAGCTG 276
ATCGGGATGACTTGTGGCTAG 893
TACCTGAAACCGGATGCCTAC 5192
CTGTTTAGCAAAAACACAGGG 13
AGACGACGTATAGGGCCTGAC 22
GCAGAGACGAAAGTCGGTCAT 103
ACGATTTCCGAATGGGGCAAC 19
AAGGCAATATGGAAGTAGGGC 25
GAAAGCGTGGGGAGCAAACAG 12
AGTGAAATAGTACCTGAAACC 25
GCACTCTAAGGGGACTGCCGG 277
GGTACGCCGGGGATAACAGGC 92
TACCCTGGTAGTCCACGCCGT 293
CTTGAGAGTTTGATCCTGGCC 12
ACGTCCGTCGTGGAGAGGGCA 51
GGAAGTCTTGAGTATGGTAGA 45
AACTCGGCCGTGAAACGCTCG 13
ATATTGGACAATGGGCGCAAG 572
GTGAACCAGTACCGTGAGGGA 30
ATAGGTGGTAGACTTTGAAGC 66
ATGAATGTTAGCCGTCGGGGT 6609
CCAACGCAGACTCAGTGAAAT 110
GTGAGACAGTTCGGTCCCTAT 142
ACTGTTTGTTGATTTGTGAGT 28
ACGGACGGGATAACCGCTGAA 2428
GCATGCCGCGGTGAATACGTT 209
CTCGGCCGTGAAACGCTCCAG 32888
GCTAAGTGGGAAAGGATGTGA 156
GTGAAGTCGGAACAAGGTAGC 20
GAAAGTCGGTCATAGTGATCC 119
GGTTTAGAACGTCGTGAGACA 14
AGCGAACCTGGGGAACTGAAT 31
GATTAGATACCCTGGTAGTCC 75
TAGGGTCTGACGCCTGCCCGG 1175
TGGTGACGGATCGCGTGTGTT 19
GATGCCTTGGCATGCACAGGC 33
GAGCGCCCCGCAAGGGGAGCG 70
AAGGCGGCTCACTGGACCATT 375
GGGAGAGTAGGTCGCTGCCAG 4335
TTTGGACAATGGGCGCAAGCC 59
CTGCAGGTGGTGACGGATCGC 103
AGTCCATATCGACGGGGTTGT 63
GAAGGCAATATGGAAGTAGGG 21
TTGCGTCGGTATCTGGGCTTG 47

TAGTCATCATAAATAAGGTAT 37
ACAGGATCTGTCCCTAGTACC 21
GGCGAGCGAACGCGGACCAGG 49
AGCGCACGTAGGCGGACTTTT 62
ATGAATGTTAGCCGTCGGGGC 38
GAACGCTGGCGGCAGGCTTAA 1800
GCTCACTGGTCTAAATAAGGG 20
TCAGTTCGATGCATTAGACCC 154
CGGGCTGGGTTACACACGTGC 10
AGCATTCAGTTGGGCACTCTA 13
CTGCATATAGACCGTACCCTA 241
GTGGAGCAGCCCGGTAGCTCG 172
GCACGTATGTGCGTGGTAGCG 57
CTGATGACCCCCAAGAGTCCA 265
TGACGTCAAGTCCTCATGGCC 48
CAGCGACTGGGGTGAAGTCGT 12
TTGAGTAGGGCGGGACACGTG 36
CCAGCGCCAATGGTACTTCGT 52
TCGGTCATAGTGATCCGGTGG 621
CTGTAGCTCAGCTGGGAGAGC 85
CTTGAGAGTTTGATCCTGGCT 2201
ACATCTAAGTACCCAGAGGAC 73
GGACAATGGGCGAAAGCCTGA 11
CCAATCAAACTTGGAGATAGC 15
GTTTGACTGGGGCGGTCGCCT 212
TAAGAGGAGAGGTGCAAGCCT 130
GGCTACACACGTGCTACAATG 122
CGCATCCTGGGGCTGGAGCAG 456
TTCAGTTGGGCACTCTAAGGT 26
CGAGCTGGGCTACACACGTGC 12
CTCGGCCGTGAAACGCTCCAA 13
AGGGCCATCGTTCAACGGATA 47
CTAGTAATCGCGGATCAGCAT 142
GGCCCGCACAAGCGGTGGAGC 151
AGCGAACCTGGGGAACTGAAC 99
GGGTGTGGAAGTGCGGCAACG 303
AGCGAGTCTGAACAGGGCGTT 49
GGGCTGGGCTACACACGTGCC 139
TGCTAACGTCCGTCGTGGAGA 26
TCAGCCTGACGATCGCTTGCA 10
TCGGTATCTGGGCTTGTAGCT 101
ACCCACCTTAGATGACTAGAA 51
GCGAGCGAACGCGGACCAGGC 1761
AGTAACGGAGGCGCGCGATGG 90
CTTTGCAAGCAGGGGGTCGTC 98
GAAACATCTAAGTACCCAGAG 50
ACTCTCCCGCGTCTTGAGACG 34
CCAAGGCGACGATCCATAGCT 263
ATGCCGCGGTGAATACGTTCC 238
CAACCCTCGCCCTTAGTTGCC 13
AGTACCTGAAACCGGATGCCG 23
TAGATATTCGGAGGAACACCG 110
AAGAGGAGAGGTGCAAGCCTT 126
ACCCCGGAACTGCCTTTGATA 147

GACGAAAGTCGGCCATAGTGA 10
GTAGCTATATACGGACGGGAT 50
GGCCGTGAAACGCTCCACCGC 11
CACGTAGAATAGCAGAAGTCC 603
GCGCGATGGTAGGCTCAGAAC 1322
ACGCTGCGATAAGCGTCGGGC 18
GAGGAAAGGACATCAAACGAG 21
CGAGCAAGCTTAAGCCGGTAG 106
CGCCACACTGGGACTGAGACA 19
TTTGACTGATCTAGAAGCCCG 22
CCCGGTGCTGGAAGGTTAAGA 21
ATCGAGAATTGGAAAGAGGCC 186
CATGGGTCGACCACGATCCAA 987
GCCTAAGTACTCGTGCATGAC 269
GGTTATGGCGGAGCGGCTGCA 182
GGGCTGGGCTACACACATGCT 11
TATTGACAGGATCTGTCCCTA 248
CATGAAGCTTACCGGTACTAA 88
CAAGTGATCTAGCCATGAGCA 92
CGAGAAGCTGGTCTTTCTGCT 305
TAGATATTCGGAGGAACACCA 3737
TAGGAATATTGACAGGATCTG 793
TCCTGGAGGTATCGGAAGTGA 21
GGGGACTCACCGTCTTACTGA 421
GGGCTGGGCTACACACGTGCT 49173
GCCGCAGGTTCAAATCCTGCC 13
AATACGTTCCCGGGCCTTGTA 44
GAGAATTGGAAAGAGGCCGGA 71
TTGGACAATGGGCGAAAGCCT 24
CCGGGCCTTGTACACACCGCC 262
GCGGCAGGCTTAACACATGCA 116
ATTGGGACTGAGACACGGCCC 37
CGTAAAGCGCACGTAGGCGGA 329
GGACGTGATACGCTGCGATAA 28
TATTCGGAAGAACACCAGTGG 38
AACATTCCGCCTGGGGAGTAC 268
CCAGCCACACTGGGACTGAGA 13
TCGTCGAGTGCAATGGCATAA 35
GGATAAAAGGTACTCCGGGGA 46
GCGGACCAGGCCAGTGGCTTG 10
GCCAATGGTACTTCGTCTCAA 32
AAGGTACGCCGGGGATAACAG 120
GTGGCTAGGGGTGAAAGGCCC 20
ACAATAACGGTCCTAAGGTAG 19
CTCCAACGCAGACTCAGTGAA 83
AGTGGGGAATTTTGGACAATG 47
CAAGTCGAGCGCCCCGCAAGG 85
TCAACCCCGGAACTGCCTTTG 27
CTGGGCGTAAAGCGCACGTAG 210
TTGAGAGAACTGCGTTGAAGG 4827
AGGGGGTCGTCGGTTCGATCC 771
CGGAGGCGCGCGACGGTAGGC 10
GAATTGACGGGGGCCCGCACA 18
TGACAGTGGGCAGCGAGCACG 369

TAGGTCGTCGGCCCATGTGGG 21
AAAGGAATTGACGGGGGCCCG 31
TAAGTACTCGTGCATGACCGA 85
TGGGCACTCTAAGGTGACTGC 25
GGCGACCACGGTAGGGTCAGC 2032
ACAACCAGGATGTTGGCTTAG 21
ACCGCCATCACCGATTGTATC 16
AGGCAAAGAACAGGCGCAGCC 10
GAGGAACACCAGTGGCGAAGG 1127
CGAAATTCCTTGTCGGGTAAC 60
AATACCCTTTGATCCGACGAT 850
GCACCTCGATGTCGGCTCATC 26
TGCAGCTTACCGGTACTAATA 24
CGGTAGCTCGTCAGGCTCATA 72
CGGAGGAACACCAGTGGCGAC 58
TGCTTTGCAAGCAGGGGGTCG 90
AGTGAAACAGTACCTGAAACC 27
CCGAAGCTGTGGATGCACGTA 68
CAGTGAAATTGAATTCCCCGT 35
ATATTCGGAAGAACACCAGTG 38
ATGTCGACTCATCGCATCCTG 178
AGTACTCGTGCATGACCGATA 24
CGGGGTAGAGCAGCCCGGTAG 10
TACGAGAGGACCGGGATGGAC 382
AATGATCGGCCCGCGTTGGAC 16
TCGATGGGAACCACGTTAATA 25
TGGCTCAGAACGAACGCTGGC 3307
CTGGAAGTCTTGAGTATGGTA 32
TGACTGGGGCGGTCGCCTCCT 128
CATATAGACCGTACCCTAAAC 276
CGAACTCGGCCGTGAAACGCA 22
TGACGACATGTGTAGGATAGA 26
GTGGGGTCGGAGGTTCAAGTC 3002
TGTCGTCAGCTCGTGTCGTGA 82
AGGCCTTAGGGTTGTAAAGCT 19
TCAAATCCTGCCCCCGCAACC 35
TGATTTGTGAGTAGTTGGGGG 13
GGTCCCTATCTGCCGTGGGTG 396
AAGCCTGATCCAGCCATGCCG 173
CAGCCTGACGATCGCTTGCAG 10
AGCCCGGTAGCTCGTCAGGCT 458
ACACGGATTTGACCTTCGGGT 29
CAGCTCACTGGTCTAAATAAG 17
GATGTGAGGATCCCAAAACAA 21
ACTCAAATGAATTGACGGGGG 16
GTGCAATGGCATAAGCCTGCC 34
CAACGGATAAAAGGTACGCCG 41
GGAAGGGCCATCGTTCAACGG 20
GACTGAGACACGGCCCAGACT 176
CCGATAGTGAACCAGTACCGT 27
AGCAGTGGGGAATATTGGACA 10
GGTAACCGGAGAAGAAGCCCC 886
CGCAAGGGGAGCGGCAGACGG 42
TCGGGGAGGTGCGAATACCCT 30

AATGATCGGCCCGCGTTGGAT 522
AAACGATGAATGTTAGCCGTC 1889
CGACTGTTTACCAAAAACACA 15
ACAGTTCGGTCCCTATCTGCC 1321
GGTAGACTTTGAAGCAGGGGC 296
AACGGTCCTAAGGTAGCGAAC 12
GAAGGCCCTAGGGTTGTAAAG 3462
GAATGCTGACATGAGTAACGA 62
CGATAGTGAACCAGTACCGTG 35
AGTAATCGCGGATCAGAATGC 11
CAGTGGGCAGCGAGCACGCGA 393
GTCCTAAGGTAGCGAAATTCC 76
TTTGCAGACCTGGCAGCGACC 61
GTTCGATCCCGTCCGGCTCCA 1046
AGGCGATGAAGGACGTGATAC 91
GCGTCTCCGTGTTTTACATGG 20
TCAAGTCCTCCCAGGCCCACC 185
CGGTCGGAAATCGTTCGTCGA 271
TTCGTAGATATTCGGAAGAAC 46
TGACGACATGTGTAGGATAGG 173
GGTTGTAAAGCTCTTTCACCG 204
ACTCTGCAACTCGAGTGCATG 142
TGGGAAAGGATGTGAGGATCC 98
GGTCGACCACGATCCAAGCCT 189
GTGACAGTGGGCAGCGAGCAC 483
ATTCAGTTGGGCACTCTAAGG 83
CCTGGGGAACTGAAACATCCA 12
GACACGGACCAGACTCCTACG 12
GCCTTTGATACTGGAAGTCTT 15
ATCGCATCCTGGGGCTGGAGA 13
TTCGTAGATATTCGGAGGAAC 1935
CGGTGGTCCCGCGTGGAAGGG 39
ACGGGCTGGGCTACACACGTA 19
CGCGGACCAGGCCAGTGGCTA 14
TCTAACTGCGGCCCGTTATCC 16
AAACACGGAGACGCAAACTTC 15
CGATTTCCGAATGGGGCAACC 19
GCTAGGGGTGAAAGGCCAATC 27
ACCCACCTGAAAACGAGTATT 10
TAGACTTTGAAGCAGGGGCGC 1056
AGCCGCGGTAATACGAAGGGG 69
CCGACGATTTCCGAATGGGGA 22
CACTGGACCATTACTGACGCC 14
ATATGGAAGTAGGGCAATAAG 43
AGGAAAGGACATCAAACGAGA 23
AAGCGTGGGGTCGGAGGTTCA 1234
AAGGCGACGATCCATAGCTGG 40
GAGTGCATGAAGTTGGAATCG 84
CTGTCTGAACATGGGTCGACC 2001
GCCCTAGGGTTGTAAAGCTCA 16
ACTTCGTCTCAAGACGCGGGA 26
GACACAGGTGCTGCATGGCTG 21
ACTGCAAGACTGACAAGTCGA 47
CACGAATGGCGTAACGACTTC 14

AATAACTCAGGGAAACTTGTG 78
GGGACACGTGAAATCCTGTCT 22
TTGCAATAGATCGGGATGACT 86
TCCATATCGACGGGGTTGTTT 85
AACAGGATTAGATACCCTGGT 12
GATGACTTGTGGCTAGGGGGG 184
CAGTTAGGCTGGACCGGAGAC 36
CTGAGCAGGGTTAGCTGGCCC 13
TGAGACACGGCCCAGACTCCT 266
ACGTTGATAGGCCGGGTGTGG 51
GACGACATGTGTAGGATAGGT 515
AAGGTAGCGAAATTCCTTGTC 216
GTTCGCCATTTAAAGCGGTAC 25
TGATCTAGCCATGAGCAGGTT 74
CGCGGGAGAGTAGGTCGCTGA 10
ATACGCTGCGATAAGCGTCGG 39
GTACTAATAGCTCGATCGACG 13
CAGGGGGTCGTCGGTTCGATC 832
GCCCTAGGGTTGTAAAGCTCG 14
GTAGTTTGACTGGGGCGGTCT 53
CCGAATACCGGGGAGTACTAG 31
ACGGGCTGGGCTACACACGTG 40025
TGCAAGACTGACAAGTCGAGC 20
CGCGGACCAGGCCAGTGGCTG 10
GGAGGCGCGCTATGGTAGGCT 24
GGCCGTGAAACGTTCCAGCGC 14
GTAGGTCGCTGCCAGGTCTGC 1479
GGCCCAGACTCCTACGGGAGG 2072
GCTATATACGGACGGGATAAC 197
CGACGAGGGGAGTGAAACAGT 17
AGGACATCAAACGAGACTCCG 337
CTGGGGCGGTCGCCTCCTAAA 31
CACTGGACCATTACTGACGCT 5441
GGTGGATGCCTTGGCATGCAC 28
AGTGGGCAGCGAGCACGCGAG 298
TTGATACTGGAAGTCTTGAGT 34
AGGATGATCCGCCACACTGGG 13
CGGCCCATGTGGGCCGCCCCC 19
ATCGCGTGTGTTGTGAGGTCT 267
GTTGAAGGCAATATGGAAGTA 13
AACAATAACGGTCCTAAGGTA 19
GGTCAGCGACTTAGTGTATCG 178
GAAGAAGCGTGACCTCACTAT 53
ACAGGCTGATGACCCCCAAGA 5244
TCCCCGTGAAGATGCGGGGTT 26
ACTGAGACACGGACCAGACTC 20
GGAATTGACGGGGGCCCGCAC 13
ACTCGTGCATGACCGATAGCG 294
TGATCAGCCACACTGGGACTA 10
TAAGTTCCGACCTGCACGAAT 16
GTGAAACAGTACCTGAAACCG 29
AACGGATAAAAGGTACTCCGG 34
GTGGTGACGGATCGCGTGTGT 13
CATTACTGACGCTGGGGTGCG 14

GGGCCCGCACAAGCGGTGGAC 13
TGACGGTAACCGGAGAAGAAG 94
GGTAGGTGTAGGCGCAGCGAA 28
CGTGGGTGTAGGAATATTGAC 57
AGATATTCGGAAGAACACCAG 24
AGAGGAGAGGTGCAAGCCTTG 162
CATGAGTAACGATAAAGGGAG 25
AAGCGGTACGTGAGTTGGGTT 12
AACGGATAAAAGGTACCCCGG 69
TGACCACCATCTAAGGTCCCT 29
TGGGGAGTTTGACTGGGGCGG 248
TCGATCGACTTGATCACTCCC 412
AGGGCGGGACACGTGAAATCC 20
TTCGTGCCAGCAGCCGCGGTA 22
AGTTGGGCACTCTAAGGGGAA 27
AAACAACCAGGATGTTGGCTT 50
GCAGCCGCGGTAATACGAAGG 53
TTTGCTACGGAATAACTCAGG 166
GCCACATTGGGACTGAGACAC 19
CCTGGGGAGTACGGTCGCAAG 844
ATGCACAGGCGATGAAGGACG 299
AAGCCGGTAGGTGTAGGCGCA 19
GAGAGAACTGCGTTGAAGGAC 10
AACTCAAAGGAATTGACGGGG 193
CACACCAACTTCGATCCGAAA 14
CACGTAGGCGGACTTTTAAGT 64
CCGTACACGTAGAATAGCAGA 393
GAAGATCGAGAATTGGAAAGA 1041
AGCCTAAGTACTCGTGCATGA 141
ATAGCTGGTCTGAGAGGATGA 76
GTGAAGATGCGGGGTTCCTGC 49
AAATTCGTAGATATTCGGAAG 25
CAGCCACACTGGGACTGAGAC 316
CGGATTTGACCTTCGGGTTTG 24
AAGACGACGTATAGGGTCTGA 56
CGACCTGCACGAATGGCGTAA 107
TGTTTTGTTGGAGCAACGCTG 19
GTAGGATAGGTGGTAGACTTT 51
GGACTGGTAGAGAATACCAAG 133
GAACGCGGACCAGGCCAGTGG 2527
TCGGGGTGTTTACACTTCGGT 55
AGTATTCCCTATCAGAGCCGT 18
TGATCAGCCACACTGGGACTG 3211
CGCGCGATGGTAGGCTCAGAA 1314
ACACTGGCATTCGTGACGACA 222
GCCAGTCAGCCTGACGATCGC 46
GGGCGTTCAGTTCGATGCATG 11
GGAACCTGCGGCTGGATCACC 28
TGGCTGTCGTCAGCTCGTGTC 263
ATTGGACAATGGGCGCAAGCC 1578
TATTCGTGGGCCTGCAGGTGG 159
ATATACGGACGGGATAACCGC 185
AAGACGACGTATAGGGCCTGA 17
AGAACTGCGTTGAAGGAACTC 1806

CCATCGCTCAACGGATAAAAG 88
GATATTCGGAAGAACACCAGT 28
AAGGCCCTAGGGTTGTAAAGC 5745
TTGGGTTAAGTCCCGCAACGA 20
GCTAACGTCCGTCGTGGAGAG 17
CTATAGCTTTACACTGGCATT 13
GAAGTCGTAACAAGGTAGCCG 12
GGGTCAGCGACTGGGGTGAAG 350
TCGGCAAATTGCACGCGTAAC 10
AGCACCTGCTTTGCAAGCAGG 52
GGCGGGTAGTTTGACTGGGGC 18
TTCGGAAGAAGCGTGACCTCA 110
ACGAGCGCAACCCTCGCCCTT 41
TACCTTTTGTATAATGGGTCA 14
GGTTCCTGCGGTTAGACGGAA 258
GCCATACACCGAAGCTGTGGA 32
CCGACCTGGTGGTTATGGCGG 14
TGGGGCAACCCACCTTAGATG 31
ACTGGAGAGTTTGATCCTGGC 12
CAGTACCTGAAACCGGATGCC 6999
CGAATACCCTTTGATCCGACG 216
AGACAGTTCGGTCCCTATCTG 188
TGTGAGGATCCCAAAACAACC 27
GGGCTCAAGCCATACACCGAA 728
GAACGTCGTGAGACAGTTCGG 289
GTGAGACATCCTGGAGGTATC 145
CGTGTCGTGAGATGTTGGGTT 150
CTGGACCATTACTGACGCTAA 10
GGCGCTTGAGAGAACTGCGTT 41
GCTCAGAACGAACGCTGGCGG 2695
ACTGGGGCGGTCGCCTCCTAA 37
CGGGCCTTGTACACACCGCCC 16
GGAAGAGATTTTGGACGGTTT 10
ACTCGGCCGTGAAACGCTCCG 14
AGACCAGGGGGTAGCGACTGT 72
CAGCTCGTGTCGTGAGATGTC 10
GAGACGAAAGTCGGCCATAGT 10
GGCTGATGACCCCCAAGAGTC 4266
GTGAAATTCGTAGATATTCGG 285
GCGGGAGAGTAGGTCGCTGCC 10048
GAGATGTTGGGTTAAGTCCCG 43
GCTGAGTTTTGATGGATATTG 60
CTGGTGGACTGGTAGAGAATA 2462
AGCGAAAGCGAGTCTGAACAG 12
TTTTACGAAAGTCTGCCTGTT 17
GCTAGTTGGTGGGGTAAAGGC 41
AAGAAAGCGTAACAGCTCACT 33
ACATGGGTCGACCACGATCCA 1291
GCTGGTCTTTCTGCTGATACT 15
CGGATTGCACTCTGCAACTCG 342
GTGGGGAATTTTGGACAATGG 47
TCAGCTGGGAGAGCACCTGCT 545
CCAAACTCCGAATACCGAGGA 18
AGGAACTCGGCAAATTGCACG 21

AGACACGGACCAGACTCCTAC 10
ACATAGATCGCAGGCCAGTCA 333
ACGCTGAGGTGCGAAAGCGTG 71
ACTCGGCCGTGAAACGCTCCA 30375
GGGTTCCTGCGGTTAGACGGA 447
GCGGAAGAGATTTTGGACGGT 19
CAGCTCGTGTCGTGAGATGTT 234
GGGTAAAGGCTCACCAAGGCG 31
CTCAACGGATAAAAGGTACGC 83
CCCGTGAAGATGCGGGGTTCC 37
ACTTGTGGCTAGGGGTGAAAC 13
GGCCACACTGGGACTGAGACA 24
AGTCATCATAAATAAGGTATG 10
TGGACCATTACTGACGCTGAC 16
ACTCGGCCGTGAAACGTTCCA 13
TTGCTCACGGGCCGTACCGCA 16
GAGAACTGAAACATCTAAGTA 15
CACATTGGGACTGAGACACGG 41
AACACGTACTGGAGGACCGAA 330
GCAGGTGGTGACGGATCGCGT 66
GACCGATAGTGAACCAGTACC 12
CAACGCGCAGAACCTTACCAG 56
GCAAACCTGGGGAACTGAAAC 16
GGGCCATAGCTCAGCTGGGAG 1085
TACGTTCCCGGGCCTTGTACA 97
CCTCATGGCCCTTACGGGCTG 112
GTATGGTGGGTAGTTTGACTG 21
AGGAACACCAGTGGCGAAGGC 893
GACGGAAAGACCCCGTGCACC 364
CGGCCGTGAAACGCTCCACCG 11
ATGATCAGCCACACTGGGACT 5023
ACATCAAACGAGACTCCGCTA 116
GTCGCAAGATTAAAACTCAAA 144
AGCTGGGTTCAGAACGTCGTG 13
TCCCTATCAGAGCCGTGGAAG 54
CCTTGGCATGCACAGGCGATG 166
CAAGTCCTCATGGCCCTTACG 110
TCTGAGCAGGGGTAGCCGGCC 10
AAAGAAAGCGTAACAGCTCAC 10
GGATAAAAGGTACGCCGGGGA 1484
CCCTAAGGCGAGGCCGAAAGG 15
GTAGCCGTAGGGGAACCTGCG 29
CGGAGGAACACCAGTGGCGAA 2607
CTATCCTTCAGTTAGGCTGGA 12
CGAAATTCCTTGTCGGGTAAA 46
ATGTGTAGGATAGGTGGTAGG 12
TGGTAGAGGTGAGTGGAATTC 34
TCAAACTTGGAGATAGCTGGT 168
AGATCGAGAATTGGAAAGAGA 14
GGGCCGTGAAACGCTCCAGCG 13
GATCCTAACCAAACTCCGAAT 82
TGGCTCAGAACGAACGCTGGA 10
TCGACCGAAGTGGGTGATAGT 255
AAAGCTCTTTCACCGGTGAAG 678

TTCAGTTCGATGCATTAGACC 193
AGCAGCCGCGGTAATACGAAG 10
TCCGAATACCGGGGAGTACTA 26
CCGAGTGTAGAGGTGAAATTC 400
GCGTGACCTCACTATGGGCAA 16
ACTGGGCGTAAAGCGCACGTA 230
GACAGGATCTGTCCCTAGTAC 83
AGGCAGCAGTGGGGAATTTTG 23
ACCGATAGTGAACCAGTACCG 15
GAAGGAACTCGGCAAAATGCA 263
GACCGAATACCCCCGGGGGTA 1040
CCTGAAACCGGATGCCTACAG 11
CGAACTCGGCCGTGAAACGCC 48
ATGAAGTCGGAATCGCTAGTA 202
TCCTAAGGTAGCGAAATTCCT 81
CCATAGCTGGTCTGAGAGGAT 77
TACCCTGGTAGTCCACGCTGT 31
CAGTTCGGTCCCTATCTGCCG 993
AGACGAAAGTCGGTCATAGTG 137
GTTGTTTGGCACCTCGATGTC 175
AGATCGAGAATTGGAAAGAGG 586
GGCGCCAGCCTTTGTGGAGTC 14
ACCTGCTTTGCAAGCAGGGGG 88
GAGTAGGGCGGGACACGTGAA 27
CCGGATGCCTACAAACAGTTG 36
GCATGAAGTCGGAATCGCTAG 32
GTAGGCGGACTTTTAAGTCAG 19
ATGGCATAAGCCTGCCTGACT 177
GAGTGAGAGACTCCCTCGCCG 21
GGTGAAGTCGTAACAAGGTAG 50
TGCGGGGGCAGGATTTGAACC 18
CGGAGGAACACCAGTGGCGAG 85
TTCCGAATGGGGCAACCCACC 12
GGGGTGAAAGGCCAATCAAAC 13
GACAATGGGCGCAAGCCTGAT 254
CGAAATTCCTTGTCGGGTAAG 3305
GGTCAGCGACTGGGGTGAAGT 344
ACATCCCGGTCGCGGTTAGTG 57
GCCCAGACTCCTACGGGAGGC 1325
ATGTGTAGGATAGGTGGTAGA 1124
TCTAGCCATGAGCAGGTTGAA 23
AACGGTCCTAAGGTAGCGAAA 43
GACAGTGGGCAGCGAGCACGC 365
CCGTAGGGGAACCTGCGGCTG 29
CTTGAGTAGGGCGGGACACGT 32
GAATATTGGACAATGGGCGAA 21
CAAGATTAAAACTCAAAGGAA 81
GCTCGTGTCGTGAGATGTTGG 252
CTCTTGGGGGTCATCAGCCTG 20
TATTTGGTTGCGGGGGCAGGA 10
ACCGAAGCTGTGGATGCACGT 129
ATCGCATCCTGGGGCTGGAGC 283
TTGGTTGCGGGGGCAGGATTT 30
GTGACCTCACTATGGGCAACC 15

ATAGGGGTTAGAAGCGAACCT 83
CCTGAAACCGGATGCCTACAA 532
TGACGACATGTGTAGGATAGT 20
CGATGAAGGACGTGATACGCT 53
CCATTCCGAACTCGGCCGTGA 48
CGAACTCGGCCGTGAAACGCT 7282
GGACGTATCTCTGGTGGACCT 287
CACTGGACCATTACTGACGCA 11
CTGCGGTTAGACGGAAAGACC 55
GGGTTGTAAAGCTCTTTCACC 1582
GCATGGTTGTCGTCAGCTCGT 28
CCGACGATTTCCGAATGGGGC 168
CGGCCGTAAAACGCTCCAGCG 12
AATGGCATAAGCCTGCCTGAC 57
GAAGCCCCAGTAAACGGCGGC 10
CAAGCTTAAGCCGGTAGGTGT 15
ACGGGCTGGGCTACACACGTC 30
TTTGGTGGATGCCTTGGCATG 42
GAGAGTGATCAAGTGTCTTAA 216
TGATAGGCCGGGTGTGGAAGT 26
GCCCTAGGGTTGTAAAGCTCC 32
TGCATGACCGATAGCGAACCA 47
AAACACAGGGCTCTGCGAAGT 208
GTAGAATAGCAGAAGTCCTTG 1756
TTCCGAACTCGGCCGTGAAAC 1238
GCCGTGAAACGTTCCAGCGCC 12
GTACTAATAGCTCGATCGACC 26
GGCCCTTACGGGCTGGGCTAC 17
ATGTAATCGGATCAACTGAAG 10
CGCAGACGAGGCGCTGACACG 11
GTATTCCCTATCAGAGCCGTG 21
GGAGAGTAGGTCGCTGCCAGG 4230
AGAGTCCATATCGACGGGGTT 54
GGTGAGTGGAATTCCGAGTGT 53
TTTGTATAATGGGTCAGCGAC 128
GGGCTGCGCCTGTTCTTTGCC 56
CGTGAAGAGAAGATGTAATCG 22
GAAAGTCCAAGGGTTCCTGCT 13
ACACAGGGCTCTGCGAAGTCG 1109
GTGAAGTCGTAACAAGGTAGC 60
ACCAGGGGGTAGCGACTGTTT 1523
CTCGGCCGTGAAACGCTCGAG 12
CGTTGAAGGAACTCGGCAAAG 12
AACTATAACGGTCCTAAGGTA 15
AGCCATGAGCAGGTTGAAGGT 49
GAACGAACGCTGGCGGCAGGC 2894
GCCCTAGGGTTGTAAAGCTCT 6112
GTAGTTTGACTGGGGCGGTCG 254
TGGCGGACACGTTTCTTGGTA 119
GTTAATATTCGTGGGCCTGCA 101
CGCGGGAGAGTAGGTCGCTGC 5701
GTACTAATAGCTCGATCGACT 4899
CGTGAGATGTTGGGTTAAGTC 184
TCGGATTTACTGGGCGTAAAG 2558

CTCAAAGGAATTGACGGGGAC 22
TAAGGGGACTGCCGGTGATAA 182
CACTGGACCATTACTGACGCG 10
TTCCCGGGCCTTGTACACACC 223
TCCGCTAGTAGTGGCGAGCGA 56
CGGAACTGCCTTTGATACTGG 171
GGTAGCTCGTCAGGCTCATAA 72
CGCGGACCAGGCCAGTGGCTT 4371
CTTGAGAGAACTGCGTTGAAG 4295
TCAGTTGGGCACTCTAAGGTG 25
GGGCATTTGGTGGATGCCTTG 43
ACTCAGTGAAATTGAATTCCC 60
GAGGGCAACAACCCTGACCAC 146
GACGTGATACGCTGCGATAAG 21
GGGCTGGGCTACACACGTACT 15
TAACCGGAGAAGAAGCCCCGG 611
GTATGGTAGAGGTGAGTGGAA 23
AGCGCCCCGCAAGGGGAGCGG 74
CGTTGAAGGAACTCGGCAAAA 62
CTAACACATGCAAGTCGAGCG 15
TAAGCCTGCCTGACTGCAAGA 231
GGCTGTCGTCAGCTCGTGTCG 75
GATAAAGGGAGTGAGAGACTC 133
CCTGCGGCTGGATCACCTCCT 102
TCGTCCGTGAAACGCTCCAGC 10
TGGAGCCCAAGGTTTGTCCTG 108
TGGGCAGCGAGCACGCGAGTG 67
AAGTGCGGCAACGCATGCAGC 10
ACAGGCTGATGACCCCCAAGC 10
AAGGCCGCAGGTTCAAATCCT 15
TGGCGAGCGAACGCGGACCAG 43
TTCACCGGTGAAGATAATGAC 23
ACGGTCGCAAGATTAAAACTC 252
ATACAGGTGCTGCATGGCTGT 16
CAGCGACTTAGTGTATCGAGC 161
TGGGCTGCGCCTGTTCTTTGC 56
AGACGCGGGAGAGTAGGTCGC 728
GCAAGCAGGGGGTCGTCGGTT 778
CGGGCTAGGCTACACACGTGC 16
AAGTTCCGACCTGCACGAATG 14
AGGTACGCCGGGGATAACAGG 96
AGTTGGGCACTCTAAGGGGAC 1479
GGCCGTACCGCAGCTGACGCT 50
TGGGTCAGCGACTTAGTGTAT 180
CGGGCTGGGCTACACACGTAC 18
CCCAAGAGTCCATATCGACGG 11
GGATGTTTGGTTAGGCGGAAG 49
GTAGATATTCGGAGGAACAAC 16
CCCTATCAGAGCCGTGGAAGA 31
TAGACGGAAAGACCCCGTGCA 331
GAGAGAACTGCGTTGAAGGAA 3127
CAGTACCTGAAACCGGATGCG 10
CAGTTGGGCACTCTAAGGGGA 1545
CATGGCTGTCGTCAGCTCGTG 1193

GCATAAGCCTGCCTGACTGCA 274
TCGAGTGCATGAAGTTGGAAT 251
TTGACATCCCGGTCGCGGTTA 162
ACCAGTGGCGAAGGCGGCTCG 13
GTCGGAATCGCTAGTAATCGC 215
TAACCAAACTCCGAATACCGG 39
CTGTGGATGCACGTATGTGCG 348
GGGTTAGCCGGCCCCTAAGGC 260
TATGGTAGAGGTGAGTGGAAT 24
GTGAGGATCCCAAAACAACCA 24
CGTTAATATTCGTGGGCCTGC 96
TCGCGGTTAGTGGAGACACTA 41
CGATCCCGTCCGGCTCCACCA 62
CTGAGATACGGCCCAGACTCC 10
CCAGCAGCCGCGGTAATACGA 23
AGAATGCTGACATGAGTAACG 111
ACACGGCCCAGACTCCTACGG 4888
TGAAACCGGATGCCTACAAAC 121
GGCAGGCGACCACGGTAGGGT 245
GTCGGTATCTGGGCTTGTAGC 83
CTGTTGCAATAGATCGGGATG 24
AGATACGGCCCAGACTCCTAC 12
GGACCAGGCCAGTGGCTTTTG 561
CAGTTCGATGCATTAGACCCG 105
GGGCCCGCACAAGCGGTGGAG 151
GCACTCTGCAACTCGAGTGCA 166
AAGTGGGAAAGGATGTGAGGA 102
ACGGAGGCGCGCGATGGTGGG 14
GTCTGAACAGGGCGTTCAGTT 10
GGGCGTTCAGTTCGATGCATT 378
CATAGGGGTTAGAAGCGAACC 115
CGCGCGATGGTAGGCTCAGAC 17
ATTCGTAGATATTCGGAAGAA 45
AGTGAGAGACTCCCTCGCCGA 29
CCGGTGCTGGAAGGTTAAGAG 20
GAGTGGAATTCCGAGTGTAGA 407
AGTCCCGCAACGAGCGCAACC 81
CCGCGGTAATACGAAGGGGGC 426
ACCAGTGGCGAAGGCGGCTCA 406
AAAGCGTAACAGCTCACTGGT 216
TGCCAGCAGCCGCGGTAATAC 15
GATCCGCCACACTGGGACTGA 20
ACTGCGTTGAAGGAACTCGGC 1498
TAATGACGGTAACCGGAGAAG 23
CTCCTGCATATAGACCGTACC 597
AGGGTAGCTATATACGGACGG 19
GGCTCAGAACGGTCGGAAATC 355
GGTAACACGTACTGGAGGACC 344
TTGCAAGCAGGGGGTCGTCGG 87
TGGTGGTTATGGCGGAGCGGC 2974
GGGCGCAAGCCTGATCCAGCC 436
CCGTACCGCAGCTGACGCTGC 17
TTGGCCGTGAAACGCTCCAGC 16
CAGTACCTGAAACCGGATGCA 20

TTGATGAGGGGCCGTAGCTCA 150
GGGCAACAACCCTGACCACCA 27
CCTGGGGAGTACGGTCGCAAC 13
AGGGTCTGACGCCTGCCCGGT 1293
TGTCCTGGGTGACAGCGTACC 56
AGCACCCCGACGAGGGGAGTG 32
ATGGGTCGACCACGATCCAAG 975
TTTATGGATGTCTAACTGCGG 17
CAGTTGGGCACTCTAAGGTGA 26
CGGTAGGTGTAGGCGCAGCGA 34
TGAAGTTGGAATCGCTAGTAA 94
GGGCTACACACGTGCTACAAT 219
TGGACCATTACTGACGCTGAG 18425
AGTCATCATAAATAAGGTATC 20
ACTTGTGGCTAGGGGTGAAAG 1009
GTGGATGCCTTGGCATGCACA 24
ACTGGGCCGTGAAACGCTCCA 13
ACTGGTTGGATGTTTGGTTAG 33
AGAACGAACGCTGGCGGCAGG 2308
GATGTCGACTCATCGCATCCT 102
CAGCGAGCACGCGAGTGTGAG 21
TGGGACTGAGACACGGCCCAG 122
AGCGACTGGGGTGAAGTCGTA 12
CACCACGTTGATAGGCCGGGT 156
CGTGAGTGATGAAGGCCCTAG 19
AAGTCCAAGGGTTCCTGCTTA 18
CGATGGTAGGCTCAGAACGGT 251
GAAATCGTTCGTCGAGTGCAA 15
TAGGGGTGAAAGGCCAATCAA 12
CTGTGAAGGGACAGTCGTGAG 41
CTTGATAAGCGTGGGGTCGGA 102
GAGAGACTCCCTCGCCGAAAG 24
TCGATGTCGACTCATCGCATC 43
ACCATTACTGACGCTGAGGAG 10
GCCTGACTGCAAGACTGACAA 67
CTAAACCGACACTGGTGGACT 124
GGATTAGATACCCTGGTAGTC 63
CAACGAGCGCAACCCTCGCCC 100
GACGACGTATAGGGTCTGACG 205
ATCCCGGTCGCGGTTAGTGGA 116
AGAACGAACGCTGGCGGCAGA 16
GTTTTGTTGGAGCAACGCTGG 26
TGTTTGTTGATTTGTGAGTAG 27
CTGTTTGTTGATTTGTGAGTA 27
GGTAGCGACTGTTTACCAAAA 84
CTCACCAAGGCGACGATCCAT 350
GTTGGAGCAACGCTGGATGGG 33
GGGCTACACACGTGCTACAAC 25
CGGAGACAGGTGCTGCATGGC 270
CGGGGTGTTTACACTTCGGTG 29
TGGACCATTACTGACGCTGAA 63
GTAGGCTCAGAACGGTCGGAA 149
CGGGCTGTGCTACACACGTGC 10
CTTTGAAGCAGGGGCGCCAGC 552

GACGCCTGCCCGGTGCTGGAA 228
ACGAGTATTCCCTATCAGAGC 11
TGGACTGGTAGAGAATACCAA 202
ATGGGCAACCATAGGGGGGTG 34
TGGTGCATGGCTGTCGTCAGC 15
CACAGGGCTCTGCGAAGTCGC 1095
AGTTTGACTGGGGTGGTCGCC 22
ACGACGTATAGGGCCTGACGC 40
GTTAGAAGCGAACCTGGGGAA 2650
GTTAAGAGGAGAGGTGCAAGC 28
CAAAGGAATTGACGGGGGCCC 69
CAAATGATCGGCCCGCGTTGG 2032
CATAGCTGGTCTGAGAGGATG 70
GTAGGGGAACCTGCGGCTGGA 59
GTTCGTCGAGTGCAATGGCAT 37
GACTGGTAGAGAATACCAAGG 65
GCGATGAAGGACGTGATACGC 90
ATTAGATACCCTGGTAGTCCA 133
GACTGATCTAGAAGCCCGGCA 51
CTAAGTACTCGTGCATGACCG 246
CCGTAAACGATGAATGTTAGC 240
GCGGCTGCACCCGATCCCATT 15
CGCGGACCAGGCCAGTGGCCT 16
GCCCGCACAAGCGGTGGAGCA 33
TTCCGCCTGGGGAGTACGGTC 2593
TTACGGGCTGGGCTACACACG 1454
CGGGATGACTTGTGGCTAGGA 11
AAAGGCAAAGAACAGGCGCAG 42
AGGCGCGCGATGGTAGGATCA 16
CCAAAAGCCGTCTCAGTTCGG 22
CTCAAAGGAATTGACGGGGGC 206
TCTCCAACGCAGACTCAGTGA 71
GGCTGTAGCTCAGCTGGGAGA 249
TGCGTTGAAGGAACTCGGCAC 16
GAAGAGATTTTGGACGGTTTA 10
TACTGACGCTGAGGTGCGAAA 3228
ACGCGGACCAGGCCAGTGGCA 39
CAACCCTGACCACCATCTAAG 33
TACACACCAACTTCGATCCGA 14
CGGAGGCGCGCGATGGTGGGC 18
GGAGGCGCGCGATGGTAGGCT 36784
ACGGATAAAAGGTACCCCGGG 78
TTTGAACCTGCGGCCTTCAGG 111
GCGTGGGGTCGGAGGTTCAAG 5676
ATTTATCGGCAAATGATCGGC 36
TCCTGGCTCAGAACGAACGCT 3299
AAGGACGTGATACGCTGCGAT 45
GACTCAGTGAAATTGAATTCC 59
TTGTATAATGGGTCAGCGACT 194
GACTGTTTATCAAAAACACAG 10
ACGCGGGAGAGTAGGTCGCTG 5628
TGCGGCTGGATCACCTCCTTT 151
CAAGCCATACACCGAAGCTGT 54
GCTCAACGGATAAAAGGTACT 113

GTCCTGGGTGACAGCGTACCT 24
GGAGGCGCGCGATGGTGGGCT 20
GAAGATGCGGGGTTCCTGCGG 47
ACCAGCCACACTGGGACTGAG 10
TCGCATCCTGGGGCTGGAGCA 473
AAAATGCACGCGTAACTTCGG 59
GCGGTTAGACGGAAAGACCCC 63
AAGGTACGGTAACACGTACTG 24
GAGGTGCGAAAGCGTGGGGAG 70
CGTGGGGTCGGAGGTTCAAGT 3281
CTGACGCTGAGGTGCGAAAGC 314
GGAAATCGTTCGTCGAGTGCA 36
CGCTGCGATAAGCGTCGGGGC 19
AAATCGTTCGTCGAGTGCAAT 10
GTCCCGCGTGGAAGGGCCATC 10
TAACTATAACGGTCCTAAGGT 10
GGCGGACACGTTTCTTGGTAA 108
GATGACCCCCAAGAGTCCATA 28
TGATACGCTGCGATAAGCGTC 32
TGAGACACGGACCAGACTCCT 10
ACGCGGACCAGGCCAGTGGCG 22
TGCAAGTCGAGCGCCCCGCAA 66
GAAGGGCCATCGCTCAACGGA 241
GATCCGACGATTTCCGAATGG 187
GCATGGCTGTCGTCAGCTCGT 1215
GGACTTTTAAGTCAGGGGTGA 31
GAACTGAAACATCTAAGTACC 124
GTCGGTCATAGTGATCCGGTG 216
GGCTAGGGGTGAAAGGCCAAT 24
GTCGTGAGATGTTGGGTTAAG 134
CGGGATGACTTGTGGCTAGGG 16070
CCCGCAAGGGGAGCGGCAGAC 57
ACGATCCAAGCCTAAGTACTC 39
ATGAGTAACGATAAAGGGAGT 29
TCGCAAGACGACGTATAGGGT 24
GCTGTTCGCCATTTAAAGCGG 26
TGATCCAGCCATGCCGCGTGA 19
GCTCAACGGATAAAAGGTACC 16
GTTAGTAGTGGCGAGCGAACG 25
AATACCCCCGGGGGTAGAGCA 17
TAGTACGAGAGGACCGGGATG 153
TCCAAAAGCCGTCTCAGTTCG 21
CGTGGGGTCGGAGGTTCAAGC 11
TTTAAGTCAGGGGTGAAATCC 22
GTATCGGAAGTGAGAATGCTG 23
GGGTAGCGACTGTTTACCAAA 243
GGAGGCGCGCGATGGTAGGCC 70
GCGCCCCGCAAGGGGAGCGGC 87
ATGTACCGGGGCTCAAGCCAT 522
TCCTGGCTCAGAACGAACGCC 24
GCGTGGGGTCGGAGGTTCAAA 16
TTTACGAAAGTCTGCCTGTTC 12
CATGGTTGTCGTCAGCTCGTG 31
AGCAGGTTGAAGGTACGGTAA 313

CTAAGGAAGATCGAGAATTGG 45
GGCTCAGAACGAACGCTGGCG 2629
CGAAAGTCGGCCATAGTGATC 10
AATAGCTCGATCGACTTGATC 13
AAGGGCCATCGCTCAACGGAT 861
GTAGTCGATGGGAACCACGTT 59
TTAGAAGCGAACCTGGGGAAC 8677
CCCCGGAACTGCCTTTGATAC 213
TTTAAGAGCTGAGTTTTGATG 11
TATAGGGTGTGACGCCTGCCC 15
GAGAACTGCGTTGAAGGAACT 2395
GGCTCACTGGACCATTACTGA 7627
CGATCCAAGCCTAAGTACTCG 15
ATCTAGAAGCCCGGCACCGCA 30
CCTGGAGGTATCGGAAGTGAG 22
AGAACACCAGTGGCGAAGGCG 13
AACGATAAAGGGAGTGAGAGA 191
GCTCACCAAGGCGACGATCCA 359
GCGGGGTAGAGCAGCCCGGTA 10
TAGTGGCGAGCGAACGCGGAC 101
CAGAACGAACGCTGGCGGCAC 11
GCCGTGAAACGCTCCAGAGCC 10
GCAGTGGGGAATTTTGGACAA 54
AAGAGCTGAGTTTTGATGGAT 286
AACGAGGGCGGCGGCGCCGGC 24
CCAGTGGCGAAGGCGGCTCAC 384
ACCTGAAACCGGATGCCTACA 3765
CCTGGGGAACTGAAACATCTC 14
TAGTTGGTGGGGTAAAGGCTC 40
TGGACAATGGGCGAAAGCCTG 17
GAGACATCCTGGAGGTATCGG 116
TGTTGGAGCAACGCTGGATGG 35
ACTATCCTTCAGTTAGGCTGG 12
GGCTATGGGGACTCACCGTCC 13
CCCGGCTAACTTCGTGCCAGC 50
TCATCGCATCCTGGGGCTGGA 283
GCGGAGCGGCTGCACCCGATG 25
GACATCCTGGAGGTATCGGAA 105
AGCGAACCAGTACCGTGAGGG 192
GAGAACTGCGTTGAAGGAACC 15
GGCTCACTGGACCATTACTGG 13
ACGACTTCCCCGCTGTCTCCA 229
TCACCAAGGCGACGATCCATA 348
GACGGGATAACCGCTGAAGGC 2273
GAAGTAGGGCAATAAGGCAAT 12
CAGGGCGTTCAGTTCGATGCA 294
CACGGAGGCGCGCGATGGTAG 16
AACTTTGGCGGACACGTTTCT 24
GCCCTTGACATCCCGGTCGCG 161
TGAACATGGGTCGACCACGAT 1449
CGAGGGCGGCGGCGCCGGCAG 44
CATTGTCTGCGGATGGTTCGA 33
CATTAAACATTCCGCCTGGGC 10
GCGTCGGGGAGGTGCGAATAC 521

TCGGCTCATCGCATCCTGGGG 17
CACCTCGATGTCGGCTCATCG 10
GGCTATGGGGACTCACCGTCT 940
GTAGCGTTTGCGTCGGTATCT 20
GAGTGAAATAGTACCTGAAAC 15
AACCAGGATGTTGGCTTAGAA 49
GAACCTGCGGCTGGATCACCT 27
GCGGAGCGGCTGCACCCGATA 50
GCCTAACACATGCAAGTCGAG 12
GGGAATTTTGGACAATGGGCG 45
TTTGGTTGCGGGGGCAGGATT 36
GATCTAGCCATGAGCAGGTTG 65
CGGGAGAGTAGGTCGCTGCCA 6024
GGGCCGTAGCTCAGCTGGGAC 17
GGAATTTTGGACAATGGGCGC 43
GCAAGTCGAGCGCCCCGCAAG 101
ACCTGAAACCGGATGCCTACG 40
TCGACCGAATACCCCCGGGGG 4503
GTACCCCGGGGATAACAGGCT 54
ATTGTCTGCGGATGGTTCGAG 19
GGGCAGCGAGCACGCGAGTGT 43
CCTGGGGAACTGAAACATCTT 32
ATCTAAGCGGGAAACCCACCT 159
TCGGCAGACACACGGCGGGTG 334
TCATAGTGATCCGGTGGTCCC 665
ATATTCGTGGGCCTGCAGGTG 154
CTGGACCATTACTGACGCTGT 11
ACCATCTAAGGTCCCTAAGTT 12
CCGTGAAGATGCGGGGTTCCT 52
GCCCTTACGGGCTGGGCTACA 13
CCCCGACGAGGGGAGTGAAAC 12
TGATCCGCCACACTGGGACTG 29
GACATGAGTAACGATAAAGGG 19
GACACGTTTCTTGGTAAGAAC 51
ATCCGGTGGTCCCGCGTGGAA 37
TAAACCGACACTGGTGGACTG 50
GCTGGGCTACACACGTGATAC 19
TGGAAGTGCGGCAACGCATGC 28
CATGCACAGGCGATGAAGGAC 439
GGCGCTGACACGGATTTGACC 20
GGTATCTGGGCTTGTAGCTCA 25
ACACCAGTGGCGAAGGCGGCC 12
ACTCCGGGGATAACAGGCTGA 18
GAGTATGGTAGAGGTGAGTGG 15
TGCACAGGCGATGAAGGACGT 182
TCCCGGGTCTTGTACACACCG 12
ATAAAAGGTACCCCGGGGATA 69
TACGGCCCAGACTCCTACGGG 18
TGTGGATGCACGTATGTGCGT 344
CCTGGCTCAGAACGAACGCTA 15
GCTCAGCTGGGAGAGCACCTG 744
TCCCCGCTGTCTCCAACGCAG 670
CACAGGCGATGAAGGACGTGA 121
GGGTAGCTATATACGGACGGG 22

GATGCACGTATGTGCGTGGTA 11
CTTCGGAAGAAGCGTGACCTC 106
TTGATAGGCCGGGTGTGGAAG 25
TGACAGGATCTGTCCCTAGTA 89
ATGGCGGAGCGGCTGCACCAG 12
TGTAGGATAGGTGGTAGACTT 61
AAGAGTCCATATCGACGGGGT 20
CCAAGGTTTGTCCTGGGTGAC 43
GCATATAGACCGTACCCTAAA 255
ATGAAGCTTACCGGTACTAAT 89
GGGCGTAAAGCGCACGTAGGC 243
GATCTAGAAGCCCGGCACCGC 31
TTTGACTGGGGCGGTCGCCTC 172
AGGCTCAGAACGGTCGGAAAT 281
TGGGCGCAAGCCTGATCCAGC 163
TCAACGGATAAAAGGTACCCC 33
TGGACCATTACTGACGCTGGG 17
TGAAGTCGGAATCGCTAGTAA 238
CTGGACCATTACTGACGCTGC 15
TGAAGGACGTGATACGCTGCG 35
CTGTTCGCCATTTAAAGCGGT 26
GGCATTGTCTGCGGATGGTTC 55
CCTATCAGAGCCGTGGAAGAC 30
GCTCTTTCACCGGTGAAGATA 23
AGTAGGGCAATAAGGCAATAT 10
CGTTCCGTAAGCCTGTGAAGG 18
GGGCTGGGCCACACACGTGCT 12
GGACACAGGTGCTGCATGGCT 12
ACTGGTAGAGAATACCAAGGC 74
CGGACCAGACTCCTACGGGAG 20
CGTGAAATCCTGTCTGAACAT 84
AGAGGATGATCAGCCACACTG 233
ACCATTACTGACGCTGAGGGG 107
TCACCGTCTTACTGATCCTAA 155
CCTGGCTCAGAACGAACGCTG 3446
CGGGCTGGGCTACACACGGGC 18
TGATCTAGAAGCCCGGCACCG 41
TGACTGCGTACCTTTTGTATA 12
GTTCAAGTCCTCCCAGGCCCA 130
ACACCAGTGGCGAAGGCGGCT 2721
AGACCGTACCCTAAACCGACA 974
GGAGCGGCTGCACCCGATCCC 1115
GTACTCGTGCATGACCGATAG 30
TACGGGCTGGGCTACACACGA 14
CACAGGTGCTGCATGGCTGTC 40
ACGAGGCGCTGACACGGATTT 57
AACGGCGGCCGTAACAATAAC 12
ATAGACCGTACCCTAAACCGA 1495
ACTGACGCTGAGGTGCGAAAG 1106
GAAGCCCCGGCTAACTTCGTG 98
CGAATGGCGTAACGACTTCCC 11
AGGTATCGGAAGTGAGAATGC 20
TATGGATGTCTAACTGCGGCC 105
CTTGGCCGTGAAACGCTCCAG 15

CGGGCTGGGCTACACACGTGT 12
GGATGACTTGTGGCTAGGGGT 13719
GGCCGTGAAACGCTCGAGCGC 10
AGCGTCGACCGAATACCCCCG 5374
ATGAGCCTGACGAGCTACCGG 53
AAAACTCGACCGAAGTGGGTG 10
CTGAGACACGGCCCAGACTCC 374
ACTGAAACATCTAAGTACCCA 52
AGGCGCAGCGAAAGCGAGTCT 25
GAGACTCCGCTAGTAGTGGCG 14
GTGAAGTCGAAACAAGGTAGC 27
GAAAGTCGGCCATAGTGATCC 10
GGTCCCTAAGTTATGGCTAAG 111
CTGAGCAGGGTTAGCCGGCAC 15
AACTTCGTGCCAGCAGCCGCG 21
CCGAAGGCGCTGTGCTAACCC 21
GAGAATGCTGACATGAGTAAC 132
CTCGGCCGTAAAACGCTCCAG 12
AAACCCACCTGAAAACGAGTA 10
AGAGCTGAGTTTTGATGGATA 283
TTGGATGTTTGGTTAGGCGGA 65
ACATGTGTAGGATAGGTGGTC 11
CGTGACGACATGTGTAGGATA 355
ATGAGCAGGTTGAAGGTACGG 477
CGGCAAAATGCACGCGTAACT 30
CGATGTCGGCTCATCGCATCC 12
GCCGGGGATAACAGGCTGATG 16
ATCCGCCACACTGGGACTGAG 20
ACCGTATGTGCCCTTCGGGGG 56
GAGGAGGTCGCTGCCAGGTCT 14
ATGCCTACAAACAGTTGGAGC 45
TCGTGAGATGTTGGGTTAAGT 156
TCTGAACATGGGTCGACCACG 1419
AAGTCAGGGGTGAAATCCCGG 32
GGATGACTTGTGGCTAGGGGC 60
CGGGCTGGGCTACACACGTGC 52043
GTCGGCCCATGTGGGCCGCCC 20
CGGAGGCGCGCAATGGTAGGC 10
GCGTAACAGCTCACTGGTCTA 46
CATAACCACCAGGTCGGCGAA 60
CACCAGTGGCGAAGGCGGCTC 2580
TCAGAACGAACGCTGGCGGCC 17
TACCCAGAGGAAAGGACATCA 33
AGCAGCCCGGTAGCTCGTCAG 239
ATCATGTTGGTGTTGAGACGG 22
CGCGGGGTAGAGCAGCCCGGT 10
ACGCCGTAAACGATGAATGTT 182
AGTGATCTAGCCATGAGCAGG 99
CGGGCTGGGCTACACATGTGC 15
CAACGCATGCAGCTTACCGGT 33
ACTGACGCTGAGGTGCGAAAA 13
GTAACCGGAGAAGAAGCCCCG 841
GTAGAGCAGCCCGGTAGCTCG 10
GAGCCCAAGGTTTGTCCTGGG 38

GGAATATTGGACAATGGGCGA 14
CTCGATCGACTTGATCACTCC 410
TGAGACATCCTGGAGGTATCG 114
ATATAGACCGTACCCTAAACC 1550
CGCAGACTCAGTGAAATTGAA 78
CGAGCGAACGCGGACCAGGCG 12
GCGGTACGTGAGTTGGGTTCA 100
CGGATGCCTACAAACAGTTGG 47
GTCCGTCGTGGAGAGGGCAAC 36
GCAAGGAGGCAGGCGACCACG 106
GTATAATGGGTCAGCGACTTA 80
AATTCGAAGCAACGCGCAGAA 31
AGAACGGTCGGAAATCGTTCG 63
AAAAGCACCCCGACGAGGGGA 31
CAGGTTGAAGGTACGGTAACA 190
AACCGCTGAAGGCATCTAAGC 10
CGAGACTCCGCTAGTAGTGGC 18
CTACGGGAGGCAGCAGTGGGG 44
GGTTAGAAGCGAACCTGGGGA 785
CTGTTTACCAAAAACACAGGG 19
TTACGGGCTGGGCTACACACT 14
ACGGGCTGGGCTACACACGCG 13
GCTGTGCTAACCGCAAGGAGG 28
CCGTACCCTAAACCGACACTG 723
CCCAGACTCCTACGGGAGGCG 18
ACGCGGACCAGGCCAGTGGCC 36
TGCGTTGAAGGAACTCGGCAA 349
GCGGCTGGATCACCTCCTTTC 166
GTCGGGTAAGTTCCGACCTGC 24
CTAGAAGCCCGGCACCGCAGA 12
ACCACGATCCAAGCCTAAGTA 37
GGCAGCGAGCACGCGAGTGTG 35
CCTGACTGCAAGACTGACAAG 70
GAATACGTTCCCGGGCCTTGT 40
AGTGCATGAAGTTGGAATCGC 50
TGTTGCAATAGATCGGGATGA 24
TGTGGAAGTGCGGCAACGCAT 81
ACTCAAAGGAATTGACGGGGG 206
GCAGCAGTGGGGAATTTTGGA 43
GGAGGCGCGCGATGGTAGGCG 24
GCCGTAAACGATGAATGTTAG 210
ATTCGGAAGAACACCAGTGGC 42
GTTCAGTTCGATGCATTAGAC 339
CGCTGCGATAAGCGTCGGGGA 57
GGTCGCAAGATTAAAACTCAA 148
AGGTCCCTAAGTTATGGCTAA 112
AGTACTAGTCGGCAGACACAC 18
GCTCAACGGATAAAAGGTACG 90
GAGTGTAGAGGTGAAATTCGT 90
GTCGTGGAGAGGGCAACAACC 24
GGCTCAACCCCGGAACTGCCT 35
GCGTAACGACTTCCCCGCTGT 94
CCCAGACTCCTACGGGAGGCA 1289
TACCGGGGAGTACTAGTCGGC 704

ACGCGGACCAGGCCAGTGGCT 6418
GCGTTCAGTTCGATGCATTAG 331
GCGGGAAACCCACCTGAAAAC 16
AGCAAACAGGATTAGATACCC 17
GAGACACTATCCTTCAGTTAG 16
TAACTTCGGAAGAAGCGTGAC 27
CTGCTTTGCAAGCAGGGGGTC 91
CCGAGAGGAAGGTGGGGATGA 102
GCGTTGAAGGAACTCGGCAAA 205
TTCTCTTTCTTCATTGTTGAT 18
TTACGGGCTGGGCTACACACC 45
GGGGGCCCGCACAAGCGGTGG 207
GAACTGAAACATCTAAGTACA 13
AGCGACTTAGTGTATCGAGCA 97
GGGTCCAGGACCGTGTATGGT 37
GACACGGCCCAGACTCCTACG 290
AGGTCCCTAAGTTATGGCTAG 11
AGGTGAAAAGCACCCCGACGA 28
GAAGTCCTTGAGTAGGGCGGG 744
CCTGCCGCCAGCGTTCGTTCT 10
GTAACGGAGGCGCGCGATGGT 674
GGTAGCGAAATTCCTTGTCGG 400
CGAAGCAACGCGCAGAACCTT 24
TCCTCATGGCCCTTACGGGCT 107
AGTACGGTCGCAAGATTAAAA 95
CAGGGTTAGCCGGCCCCTAAG 1014
GAAGTCTTGAGTATGGTAGAG 33
GATGACTTGTGGCTAGGGGAG 15
CAATGAGATTGATCAAGTGTC 11
ACTCAAAGGAATTGACGGGGA 22
GCGTGGGGTCGGAGGTTCAAC 12
AATACCAAGGCGCTTGAGAGA 62
TTAGGTAGAGCGTCGACCGAA 231
ATCCCGGTCCTCTCGTACTAG 10
TGAAATTCGTAGATATTCGGA 281
GGGTAGAGCAGCCCGGTAGCT 10
GGAGGCGCGCGATGGTAGGCA 41
AGCTCAGCTGGGAGAGCGCCT 23
ATTTACTGGGCGTAAAGCGCA 2534
GTAGATATTCGGAAGAACACC 39
ACTCCGAATACCGGGGAGTAC 20
ATGGGGCAACCCACCTTAGAT 31
CATTAAACATTCCGCCTGGGG 596
CACTGGGACTGAGACACGGCC 940
GATGTTGGGTTAAGTCCCGCA 28
CACGTGAAATCCTGTCTGAAC 44
CGGAGATATTCGGAGGAACAC 19
TACTGGGCGTAAAGCGCACGT 1235
GAGTTTGGTTAGGATCAGTAA 14
TTCGGATTGCACTCTGCAACT 109
GATAAAAGGTACTCCGGGGAT 59
CGATAGCGAACCAGTACCGTG 137
GACAGTTCGGTCCCTATCTGC 214
TCAGCGACTTAGTGTATCGAG 169

CCGGGAAACGCTCCAGCGCCA 10
AAGCGTCGGGGAGGTGCGAAT 482
GTGACTGGGGTGAAGTCGTAA 11
TTGGGCACTCTAAGGGGACTG 811
AAGAGATTTTGGACGGTTTAG 13
CAACAACCCTGACCACCATCT 36
GTGGGGAATATTGGACAATGG 65
GGTGAAGTCGGAACAAGGTAG 19
GTTGATAGGCCGGGTGTGGAA 45
CATCTCAGTTCGGATTGCACT 104
CCTGGGGAACTGAAACATCTA 1696
CATTACTGACGCTGAGGTGGG 11
ACCTGAAACCGGATGCCTACC 26
GTGAAATTGAATTCCCCGTGA 28
CAGGCTGATGACCCCCAAGAA 10
GGGCCGTAGCTCAGCTGGGAG 10826
ATTGTATCTCGAGAAGCTGGT 24
CGGGAGAGTAGGTCGCTGCCT 29
ACTGATCCTAACCAAACTCCG 103
GCGGAGCGGCTGCACCCGATT 11
ATGACGTCAAGTCCTCATGGC 27
GTTGGAGCCCAAGGTTTGTCC 86
GCATCTAAGCGGGAAACCCAC 157
GGGAGTACTAGTCGGCAGACA 19
TCGTGAGACAGTTCGGTCTCT 12
TACATAGGGGTTAGAAGCGAA 11
CTGAGTTTTGATGGATATTGG 33
TTGAGAGGATGATCAGCCACA 17
CTCCAAAAGCCGTCTCAGTTC 20
CGGGGTGGAGCAGCCCGGTAG 202
CTTTTGTATAATGGGTCAGCG 34
ATGTCGGCTCATCGCATCCTG 17
CGCCGGGGATAACAGGCTGAT 19
CGGTCCTAAGGTAGCGAAATT 78
GAGAGTAGGTCGCTGCCAGGT 4876
TATGAGCCTGACGAGCTACCG 54
ACCAGTACCGTGAGGGAAAGG 24
CTCAACCCCGGAACTGCCTTT 18
GAGAACTGCGTTGAAGGAACA 20
GATCGGGATGACTTGTGGCTA 427
TGGGGCGGTCGCCTCCTAAAG 15
TGGGCTATGGGGACTCACCGT 1441
TGATCCTAACCAAACTCCGAA 91
AGTGGGAAAGGATGTGAGGAT 136
CAGCCGCGGTAATACGAAGGG 53
AGTGAGAATGCTGACATGAGT 126
GATCGGCCCGCGTTGGATTAG 137
CTTTGATACTGGAAGTCTTGA 20
CCAAAAACACAGGGCTCTGCG 63
GGTGAATACGTTCCCGGGCCT 157
GTTCAGAACGTCGTGAGACAG 101
GCGGAGCGGCTGCACCCGATC 2939
CGTTCCCGGGTCTTGTACACA 10
AGGAATATTGACAGGATCTGT 787

GACCAGGCCAGTGGCTTTTGT 397
TGGTACTTCGTCTCAAGACGC 136
CCCTTACGGGCTGGGCTACAC 194
CATTTGGTGGATGCCTTGGCA 41
CATGCAGCTTACCGGTACTAA 38
TAGGCGGAAGAGATTTTGGAC 37
AATCAAACTTGGAGATAGCTG 15
ATCTAAGTACCCAGAGGAAAG 162
GGCAGTTTGACTGGGGCGGTC 25
ACTGACAAGTCGAGCAGAGAC 51
GGGCCGTAGCTCAGCTGGGAA 72
CAGGCTGATGACCCCCAAGAG 4891
ACGAAAGTCGGTCATAGTGAT 153
CGGGAGAGTAGGTCGCTGCCC 57
GTACGGTCGCAAGATTAAAAC 245
ACGGAGGCGCGCAATGGTAGG 10
CAGAACGAACGCTGGCGGCAG 2407
TATTGGACAATGGGCGAAAGC 43
ATAGATCGGGATGACTTGTGG 496
ACTCGGCAAAATGCACGCGTA 68
GCGTACGGCGCGTGAGCGAGA 16
GAGCGAACGCGGACCAGGCCA 1579
CGAATGGGGCAACCCACCTTA 12
CTGGACCATTACTGACGCTGG 48
AAGGTACCCCGGGGATAACAG 72
GGAGACAGGTGCTGCATGGCT 240
AACACGCACTGGAGGACCGAA 17
TCGTGAAGAGAAGATGTAATC 22
AGAGCAGCCCGGTAGCTCGTC 10
GAGCAGCCCGGTAGCTCGTCA 219
ATTCCGAGTGTAGAGGTGAAA 332
CACGATGAATGTTAGCCGTCG 10
AGGCGACGATCCATAGCTGGT 53
CGGACGGGATAACCGCTGAAG 2412
AACCGCAAGGAGGCAGGCGAC 13
TCCTACGGGAGGCAGCAGTGG 64
CGAGTCTGAACAGGGCGTTCA 80
TACGGGCTGGGCTACACACGT 3149
GTGGTTATGGCGGAGCGGCTG 1032
CGTAGGCGGACTTTTAAGTCA 32
CCTAAGGTAGCGAAATTCCTT 82
GGAGGTATCGGAAGTGAGAAT 13
CGAGGCCGAAAGGCGTAGTCG 13
TCAAAGGAATTGACGGGGACC 22
GACGAAAGTCGGTCATAGTGA 124
ATTCCCTATCAGAGCCGTGGA 50
CAGTCGTGAGACATCCTGGAG 28
AGATACCCTGGTAGTCCACGC 358
CGGTCCAGACTCCTACGGGAG 15
GGGCACTCTAAGGTGACTGCC 18
GCAACCATAGGGGGGTGGCAC 33
GATAACCGCTGAAGGCATCTA 51
GTTGATTTGTGAGTAGTTGGG 10
TTTAAGCAGGAACCCTTGGAC 10

CCGCTGTCTCCAACGCAGACT 810
GTGGAAGTGCGGCAACGCATG 41
GGTGACGGATCGCGTGTGTTG 32
CAAAATGCACGCGTAACTTCG 38
TATGTGCCCTTCGGGGGAAAG 45
CATGAAGTCGGAATCGCTAGT 82
AGTCAGCCTGACGATCGCTTG 19
AAGCGGGAAACCCACCTGAAA 145
ACATGAGTAACGATAAAGGGA 32
ACCAAGTGATCTAGCCATGAG 87
GCTCAGAACGGTCGGAAATCG 556
GCCATAGCTCAGCTGGGAGAG 644
CGGCAGACACACGGCGGGTGC 305
CGGCAGGCTTAACACATGCAA 11
GAGGTGAAATTCGTAGATATT 77
AATGTTAGCCGTCGGGGTGTT 267
GAATGGCGTAACGACTTCCCC 10
CAATCAAACTTGGAGATAGCT 15
AGTCGAGCAGAGACGAAAGTC 44
TAGCCGTAGGGGAACCTGCGG 30
TAGATATTCGGAAGAACACCA 33
CTGGACCATTACTGACGCTGA 22993
GCTCAGGACGAACGCTGGCGG 18
TAAAACTCAAAGGAATTGACG 97
AACCATAGGGGGGTGGCACAG 16
GACCACGATCCAAGCCTAAGT 38
GTCGGCTCATCGCATCCTGGG 15
GCGCTGTGCTAACCGCAAGGA 32
AGGCCCTAGGGTTGTAAAGCT 6990
AGGAACTCGGCAAAATGCACG 595
TCCCTATCTGCCGTGGGTGTA 219
ATCTCAGTTCGGATTGCACTC 114
GTTGGATGTTTGGTTAGGCGG 53
TTTTAAGTCAGGGGTGAAATC 20
AGTTCGGTCCCTATCTGCCGT 958
AGGCATCTAAGCGGGAAACCC 52
ACACCAGTGGCGAAGGCGGCG 10
TGGATGTCTAACTGCGGCCCG 70
GCGAGTCTGAACAGGGCGTTC 125
GGCGTTCAGTTCGATGCATTA 332
CCTAAACCGACACTGGTGGAC 532
AGGTGTAGGCGCAGCGAAAGC 35
GCTGTCGTCAGCTCGTGTCGT 90
GGCTGCGCCTGTTCTTTGCCT 13
GGTCGCGGTTAGTGGAGACAC 64
TGAAGGCAATATGGAAGTAGG 19
ATTCGAAGCAACGCGCAGAAC 49
TCGTTTACGGCGTGGACTACC 12
AGACACTATCCTTCAGTTAGG 16
ACACTATCCTTCAGTTAGGCT 21
AACCCCGGAACTGCCTTTGAT 82
GCTATGTACGGACGGATAAC 11
GACTTTGAAGCAGGGGCGCCA 687
TTCCGAGTGTAGAGGTGAAAT 357

CGGGCTGGGCTACACACGTGG 52
GGATGACTTGTGGCTAGGGGG 186
GCCGTCTCAGTTCGGATTGCA 22
CAACGGATAAAAGGTACCCCG 49
TACTGATCCTAACCAAACTCC 20
AGCTCACTGGTCTAAATAAGG 18
TGGAGGACCGAACCCATATCT 155
TAGACCGTACCCTAAACCGAC 1537
TTAAGGGCATTTGGTGGATGC 10
GAGGCGCGCGATGGTGGGCTC 21
AACGCATGCAGCTTACCGGTA 40
ACGCCTGCCCGGTGCTGGAAG 224
GCGACTGTTTACCAAAAACAC 21
GGAATCGCTAGTAATCGCGGA 1504
GGTAGCTATATACGGACGGGA 28
AAGGCCTTAGGGTTGTAAAGC 16
CTCACGGGCCGTACCGCAGCT 66
ATCGAACTGAACGCCCTGTTC 34
GTTGGCTTAGAAGCAGCCATC 61
GTACTAGTCGGCAGACACACG 15
AGGAGGCAGGCGACCACGGTA 95
CGAGCGAACGCGGACCAGGCC 1731
CTTGATCACTCCCATTTACAA 29
ACATGTGTAGGATAGGTGGTA 1170
ACTTTGGCGGACACGTTTCTT 134
GGTCATAGTGATCCGGTGGTC 620
AGATCGTCGGTTCTTTGAAAA 16
ACTGAAACATCTAAGTACCCT 10
TCAGAACGAACGCTGGCGGCA 2813
CCGGAGACAGGTGCTGCATGG 289
GGATGACTTGTGGCTAGGGGA 33
CATATCGACGGGGTTGTTTGG 80
CGGGCTGGGCTACACACGTGA 89
TACTCTCCCGCGTCTTGAGAC 32
AGGCCAGTGGCTTTTGTGAAT 18
TTTGCGTCGGTATCTGGGCTT 51
TCCGTCGTGGAGAGGGCAACA 25
AGGGGTGAAATCCCGGGGCTC 30
GTGTCGTGAGATGTTGGGTTA 127
ATAGCTTTACACTGGCATTCG 47
TTACTGACGCTGAGGGGCGAA 27
TTTACTGGGCGTAAAGCGCAC 2240
CGAATACCGGGGAGTACTAGT 31
AAATTCGTAGATATTCGGAGG 170
GATGTTGGCTTAGAAGCAGCC 231
ACCAAACTCCGAATACCGAGG 16
TGACTGATCTAGAAGCCCGGC 43
TACCCTAAACCGACACTGGTG 477
GGACACGTGAAATCCTGTCTG 22
TCTGAGAGGATGATCAGCCAC 130
GGAATATTGGACAATGGGCGC 546
GGTAATACGAAGGGGGCTAGC 130
GCCACACTGGGACTGAGACAC 605
CCGAAGGCGCTGTGCTAACCG 47

TTAGTAGTGGCGAGCGAACGC 25
GTTTGACTGGGGTGGTCGCCT 20
TTGCTACGGAATAACTCAGGG 221
GTGAGAGACTCCCTCGCCGAA 36
TAGATCGTCGGTTCTTTGAAA 16
TCCTTGAGTAGGGCGGGACAC 327
CTCATGGCCCTTACGGGCTGG 37
CAGCGCCAATGGTACTTCGTC 51
TGAGATACGGCCCAGACTCCT 12
CATGACCGATAGCGAACCAGT 139
AGCTCGATCGACTTGATCACT 43
TCGACGGGGTTGTTTGGCACC 1552
TATAGGGTCTGACGCCTGCCC 752
TACGCCGGGGATAACAGGCTG 76
TCTGAGCAGGGTTAGCCGGCA 22
GCTACGGAATAACTCAGGGAA 499
GACCATTACTGACGCTGAGGA 40
GCTCAAGCCATACACCGAAGC 374
TCGTAGATATTCGGAGGAACG 36
TTCGGGGGAAAGATTTATCGG 23
TCGACCACGATCCAAGCCTAA 34
CGTCGGGGTGTTTACACTTCG 93
TAAGGAAGATCGAGAATTGGA 49
ATAACGGTCCTAAGGTAGCGA 39
TGACTGGGGTGAAGTCGTAAC 13
CAAGCCTGATCCAGCCATGCC 214
TGGGTAGTTTGACTGGGGCGG 112
TGATAAGCGTGGGGTCGGAGG 34
AGGTGGTAGACTTTGAAGCAG 114
CGACCGAATACCCCCGGGGGA 10
CCGCCATCACCGATTGTATCT 16
GCATCCTGGGGCTGGAGCAGG 448
AGAGCACTGGATGGGCTATGG 26
GGATGTCTAACTGCGGCCCGT 62
GATCCAAGCCTAAGTACTCGT 15
GTAGAGAATACCAAGGCGCTT 53
TCCCGTACACGTAGAATAGCA 442
GTTTGGCACCTCGATGTCGGC 17
AAGCCGAGAGGAAGGTGGGGA 17
GGAGATATTCGGAGGAACACC 13
GGTTGTCGTCAGCTCGTGTCG 10
TTCCGACCTGCACGAATGGCG 17
AGCAGGGGGTCGTCGGTTCGA 386
CCCGGTAGCTCGTCAGGCTCA 36
GTAAACGATGAATGTTAGCCG 74
GTAAGCCTGTGAAGGGACAGT 15
GACCATTACTGACGCTGAGGG 107
ATCTCGAGAAGCTGGTCTTTC 180
CAACGCATGAAGCTTACCGGT 39
CTGCGATAAGCGTCGGGGAGG 33
GGCCGTGAAACGCTCCAGCGC 35088
TCGTAGATATTCGGAGGAACA 7088
ACGGTCCAGACTCCTACGGGA 15
GTCGCGGTTAGTGGAGACACT 41

TATGGCGGAGCGGCTGCACCA 26
AAGTACTCGTGCATGACCGAT 25
AAGGCTCACCAAGGCGACGAT 12
AGTCTTGAGTATGGTAGAGGT 32
ATGGCTGTCGTCAGCTCGTGT 265
GAGAGGATGATCAGCCACACT 159
AGGCCGGGTGTGGAAGTGCGG 561
GGCCGTGAAACGCTCCGGCGC 10
GTATGTGCGTGGTAGCGGAGC 52
ATTGACAGGATCTGTCCCTAG 291
CGACCGAATACCCCCGGGGGG 20
TCAACGGATAAAAGGTACTCC 79
GTCCTTGAGTAGGGCGGGACC 10
CCGCAGGTTCAAATCCTGCCC 12
ACTGGAAGTCTTGAGTATGGT 39
TGGGAACCACGTTAATATTCG 23
GCTGATGACCCCCAAGAGTCA 21
GAGCAGGGTTAGCCGGCCCCG 34
AAACGAGACTCCGCTAGTAGT 23
CCATCTAAGGTCCCTAAGTTA 12
AGAGGACCGGGATGGACGTAT 4118
ACCAAGGCGCTTGAGAGAACT 124
AGCAGGGTTAGCCGGCCCCTA 1910
GCTCAGTTGGTTAGAGCACAC 18
GCACTGGATGGGCTATGGGGA 1242
GAACTCGGCCGTGAAACGCTC 6828
ACAGGCGATGAAGGACGTGAT 139
GCGCGCGATGGTAGGCTCAGC 12
CCCGTACACGTAGAATAGCAG 393
TGGTTAGGCGGAAGAGATTTT 11
TACCCTTTGATCCGACGATTT 869
TTGTCCTGGGTGACAGCGTAC 61
CCACGATCCAAGCCTAAGTAC 45
GAGTACTAGTCGGCAGACACA 15
ATTAGAACATAGATCGCAGGC 22
CGCCGTAAACGATGAATGTTA 176
ACGTATAGGGCCTGACGCCTG 13
CCGGGTCTTGTACACACCGCC 21
TGTGAAGGGACAGTCGTGAGA 35
GGGCTAGGCTACACACGTGCT 16
TGCGAATACCCTTTGATCCGA 20
ATGGCCCTTACGGGCTGGGCT 119
CACTGGTGGACTGGTAGAGAA 1865
ACCACGGTAGGGTCAGCGACT 2108
GGCCATAGCTCAGCTGGGAGA 820
GTGGGAAAGGATGTGAGGATC 183
CCGAAAGGCGTAGTCGATGGG 240
GGCCTGCAGGTGGTGACGGAT 69
TGCAATAGATCGGGATGACTT 47
AACCGACACTGGTGGACTGGT 79
TCGGAAGAAGCGTGACCTCAC 518
CACGTTAATATTCGTGGGCCT 15
CGTTGTTCGGAATTACTGGGC 10
TTCGATGCATTAGACCCGAAA 34

AAGCGTAACAGCTCACTGGTC 399
CGCGGTGAATACGTTCCCGGG 206
ATGAGAGTGATCAAGTGTCTT 927
ATATTCGGAGGAACACCAGTC 12
GCATGAAGTTGGAATCGCTAG 74
GGATGTTGGCTTAGAAGCAGC 629
AGCGACTGTTTACCAAAAACA 15
GCCGAGAGGAAGGTGGGGATG 172
AACGCGGACCAGGCCAGTGGC 2615
TCAACGGATAAAAGGTACGCC 98
GAATACCCTTTGATCCGACGA 254
CTTTACACTGGCATTCGTGAC 446
ACGATGAATGTTAGCCGTCGG 7096
GATGCGGGGTTCCTGCGGTTA 70
CGAAAGCGAGTCTGAACAGGG 33
ACGTGATACGCTGCGATAAGC 39
CTCTACACTCGGAATTCCACT 10
CCGGGTCCAGGACCGTGTATG 36
TCCCGGTCGCGGTTAGTGGAG 224
ATTCGGAGGAACACCAGTGGC 2822
ACACACCAACTTCGATCCGAA 14
TCGGAAATCGTTCGTCGAGTG 92
ATAAGCGTGGGGTCGGAGGTT 158
GGCCGTGAAACGCTCCAGAGC 10
AATACCGGGGAGTACTAGTCG 67
TGTAAAGCTCTTTCACCGGTG 821
GTTGGTGGGGTAAAGGCTCAC 21
GTAGGCGCAGCGAAAGCGAGT 15
GTTAGCCGTCGGGGTGTTTAC 90
AGCCGTCGGGGTGTTTACACT 218
CGGGCTGGGCTACACACGCGC 15
AGAGAAACGTGGGCGGCATTG 11
TGCACTCTGCAACTCGAGTGC 178
ACAGACCAGGGGGTAGCGACT 133
CCGTGAAACGCTCCAGCGCAA 12
ATCTATTTAGGTAGAGCGTCG 25
GGGCTGGGCTACACACGTGTT 10
GCGAACCTGGGGAACTGAAAG 47
CTCGGCAAAATGCACGCGTAA 49
GGAGGCGCGCGATGGTAGACT 15
GGGCATGAAGTTGGAATCGCT 14
GCCGAAAGGCGTAGTCGATGG 192
ATGCACGTATGTGCGTGGTAG 27
GGTAGAGCAGCCCGGTAGCTC 10
CGCTGGCGGCAGGCTTAACAC 729
TGTTCGCCATTTAAAGCGGTA 25
AAGGACATCAAACGAGACTCC 300
TCGGAATCGCTAGTAATCGCG 90
CGCTGCCAGGTCTGCAAAGCA 10
GAACACCAGTGGCGAAGGCGG 784
CGAACCTGGGGAACTGAAACC 182
GGGGCCGTAGCTCAGCTGGGC 17
GAGACTCCCTCGCCGAAAGTC 22
CCAGTAAACGGCGGCCGTAAC 15

GTCTGACGCCTGCCCGGTGCT 1318
TATGGTGGGTAGTTTGACTGG 35
AGACCACCACGTTGATAGGCC 87
CACCTGCTTTGCAAGCAGGGG 86
ATTACTGACGCTGAGGTGCGG 23
CATCTAAGCGGGAAACCCACC 155
AAGGCGCGCGATGGTAGGCTC 12
GGCGCTGTGCTAACCGCAAGG 33
GTGGGGTAAAGGCTCACCAAG 27
TCGATGTCGGCTCATCGCATC 12
CGAACGCGGACCAGGCCAGTG 2514
CTGGGGTGAAGTCGTAACAAG 34
GGCGGCGGCGCCGGCAGCGGC 48
TGGACAATGGGCGCAAGCCTG 1392
TTCCCCGCTGTCTCCAACGCC 11
GTGGGTGATAGTCCCGTACAC 28
AAACATCTAAGTACCCAGAGG 47
AACGAGCATTTGCAGTCGAAT 11
TGCACGAATGGCGTAACGACT 16
GGAAGAAGCGTGACCTCACTA 29
GGTGCTAACGTCCGTCGTGGA 31
CTGACAAGTCGAGCAGAGACG 18
GCCGTGGGTGTAGGAATATTG 49
ATCCTGGAGGTATCGGAAGTG 20
GCGAACCTGGGGAACTGAAAA 263
AGGTTGAAGGTACGGTAACAC 137
GAACCAGTACCGTGAGGGAAA 78
GTATAGGGTCTGACGCCTGCC 905
ATTACTGACGCTGAGGTGCGA 8576
GTAGCTCGTCAGGCTCATAAC 83
CGGGGGCCCGCACAAGCGGTG 233
ACTGGTGGACTGGTAGAGAAT 2592
TTTTGTATAATGGGTCAGCGA 36
CCCCCGGGGGTAGAGCACTGG 365
CGAACCTGGGGAACTGAAACT 91
TTGGAGATAGCTGGTTCTCCG 20
GTGAGTGGAATTCCGAGTGTA 214
GTCTGACGCCTGCCCGGTGCC 12
CTTTGATCCGACGATTTCCGA 671
GCAGGGGGTCGTCGGTTCGAT 821
GTTGGAATCGCTAGTAATCGC 1286
AGAAGCGAACCTGGGGAACTA 10
AGGTACGGTAACACGTACTGG 30
TGACGCTGCTGGCCCTGCGCA 23
ATAGATCGCAGGCCAGTCAGC 192
TAAAGCTCTTTCACCGGTGAA 942
GTAGCTCAGCTGGGAGAGCAA 23
CCGAAGTGGGTGATAGTCCCG 325
AGGCTGATGACCCCCAAGAGA 19
GGCCAGTCAGCCTGACGATCG 45
GGAAAGGACATCAAACGAGAC 22
TGTCGACTCATCGCATCCTGG 166
GCAAGATTAAAACTCAAAGGA 118
TGCCACCCCCCTATGGTTGCC 15

CTGGGACTGAGACACGGCCCA 225
CAAGTCGAGCAGAGACGAAAG 32
TAGCGAAATTCCTTGTCGGGT 923
TATTTAGGTAGAGCGTCGACC 21
GTCCATATCGACGGGGTTGTT 47
AAGTTGGAATCGCTAGTAATC 156
TGAAGTCGTAACAAGGTAGCC 52
GCTGACATGAGTAACGATAAA 14
TCGTGAGACAGTTCGGTCCCA 10
TGGTAGTCCACGCCGTAAACG 88
GAAAGTCTGCCTGTTCTGTAT 10
CCGGGATGGACGTATCTCTGG 862
CAATGGGCGCAAGCCTGATCC 119
ACTTAGTGTATCGAGCAAGCT 49
GGGTGTGACGCCTGCCCGGTG 11
GCGCAGAACCTTACCAGCCCG 11
GACTGTTTAGCAAAAACACAG 13
GATGCCTACAAACAGTTGGAG 48
GGAGGCGCGCGATGGTAGCCT 12
CCAAGAGTCCATATCGACGGG 14
GCGGACTTTTAAGTCAGGGGT 116
GATGAATGTTAGCCGTCGGGC 11
TCGTGACGACATGTGTAGGAT 475
AGGGGACTGCCGGTGATAAGC 2202
CGCATGCAGCTTACCGGTACT 166
TATCAGAGCCGTGGAAGACCA 11
TACGGACGGGATAACCGCTGA 583
CAATAGATCGGGATGACTTGT 220
GGAACTCGGCAAATTGCACGC 17
TTGTTGGAGCAACGCTGGATG 39
CGTCGGTTCGATCCCGTCCGG 205
GGCAGCAGTGGGGAATTTTGG 29
AGAAGCGAACCTGGGGAACTG 9556
TAACGACTTCCCCGCTGTCTC 86
ATGCCTTGGCATGCACAGGCG 48
CGATGAATGTTAGCCGTCGGG 7159
ATATTGACAGGATCTGTCCCT 204
TTGGTGGGGTAAAGGCTCACC 26
CAAAACAACCAGGATGTTGGC 86
GCTCTGCGAAGTCGCAAGACG 380
GCGTGAGTGATGAAGGCCCTA 19
AGGCGTAGTCGATGGGAACCA 68
ACGGAAAGACCCCGTGCACCT 215
TCACTGGACCATTACTGACGC 6873
CATTACTGACGCTGAGGGGCG 68
GGCGCGCGATGGTAGGCTCAG 2077
CTAGGGTTGTAAAGCTCTTTC 1785
TGGAATCGCTAGTAATCGCGG 1312
CGGAGTTTGGTTAGGATCAGT 17
GACTGGGGCGGTCGCCTCCTA 62
TGAGAGGATGATCAGCCACAC 153
CAAAAGCCATCTCAGTTCGGA 71
GAGAGGACCGGGATGGACGAA 12
CGGACACGTTTCTTGGTAAGA 65

GAAGTATAGGGTCTGACGCCT 12
TCTAGTCATCATAAATAAGGT 357
TCGGAGGAACACCAGTGGCGC 20
AAGGTACTCCGGGGATAACAG 90
ACGGATAAAAGGTACGCCGGG 1757
GCTGCGATAAGCGTCGGGGAG 73
TCTGAGCAGGGTTAGCCGGCC 11697
AGTCCTTGAGTAGGGCGGGAC 747
GATCAGCCACACTGGGACTGG 10
TCGACGGGGTTGTTTGGCACA 22
ACTTGAGAGTTTGATTCTGGC 11
AGTCCAAGGGTTCCTGCTTAA 10
CGATGGGAACCACGTTAATAT 20
TATGGCGGAGCGGCTGCACCT 22
TGGGCGTAAAGCGCACGTAGG 211
TCGTAGATATTCGGAGGAACT 17
GGCCGTGAAACGCTCCAGCGG 35
GGGGGTAGCGACTGTTTACCA 660
ACCCTGGTAGTCCACGCCGTA 286
GACCATTACTGACGCTGAGGC 35
GGAGGAACACCAGTGGCGAGG 31
TCTGAGCAGGGTTAGCTGGCC 14
GCTACGGAATAACTCAGGGAC 28
TCAAAGGAATTGACGGGGGCC 128
ACCTGGTGGTTATGGCGGAAC 27
TCGCGTAGTAGCGTTTGCGTC 10
GTTCCTGCGGTTAGACGGAAA 121
GGATGATCAGCCACACTGGGA 4504
GACGCTGAGGTGCGAAAGCGT 42
GGCGCAGCGAAAGCGAGTCTG 30
ACCAGGATGTTGGCTTAGAAG 654
AGGGGAACCTGCGGCTGGATC 89
GTAGGAATATTGACAGGATCT 712
ACGTTCCCGGGCCTTGTACAC 115
TTGTTTGGCACCTCGATGTCG 168
GTCGTCAGCTCGTGTCGTGAG 171
ATTTGCTACGGAATAACTCAG 24
CCGCACAAGCGGTGGAGCATG 11
TCGAGTGCAATGGCATAAGCC 56
AACCGGAGAAGAAGCCCCGGC 546
CCCCAGTAAACGGCGGCCGTA 25
GTTCCGTAAGCCTGTGAAGGG 17
GGCCGTGAAACGCTCCAGCGA 25
TCGTAGATATTCGGAGGAACC 148
GCACGAATGGCGTAACGACTT 16
TTGGTTAGGCGGAAGAGATTT 11
ACACGTTTCTTGGTAAGAACT 32
TCGGCCGTGAAACGCTCCACC 13
GTCTGAACATGGGTCGACCAC 291
AGTCCCGTACACGTAGAATAG 411
AAGCTTACCGGTACTAATAGC 91
AGAGTAGGTCGCTGCCAGGGC 10
GACCATTACTGACGCTGAGGT 16772
TGGTGGGTAGTTTGACTGGGG 71

TCGGTCCCTATCTGCCGTGGG 1517
GTTAATCTGAGCAGGGTTAGC 16
GATCAGCCACACTGGGACTGA 1757
AGCCGTCTCAGTTCGGATTGC 29
TACGCCGGGGATAACAGGCTC 22
TTTGGCGGACACGTTTCTTGG 133
TGGGGGTCATCAGCCTGTTAT 15
TCGACGGGGTTGTTTGGCACG 20
GAACGGTCGGAAATCGTTCGT 67
AAAACTCAAAGGAATTGACGG 136
GCTAACGCATTAAACATTCCG 130
AGATGTTGGGTTAAGTCCCGC 28
AATGCTGACATGAGTAACGAT 35
GCTGGGTTCAGAACGTCGTGA 20
TATGGCGGAGCGGCTGCACCC 9189
TCGTTCGTCGAGTGCAATGGC 59
TACCCCGGGGATAACAGGCTG 69
GAGCAGGGTTAGCCGGCCCCT 8588
GCTGATGACCCCCAAGAGTCC 3508
CCGAATGGGGCAACCCACCTT 163
CAGTGGGGAATTTTGGACAAT 47
TCTAAGGAAGATCGAGAATTG 40
GCGAATACCCTTTGATCCGAC 211
TAAAAGGTACGCCGGGGATAA 197
GCGGGGTGGAGCAGCCCGGTA 315
GTCCTTGAGTAGGGCGGGACA 272
CGACCGAATACCCCCGGGGGT 2119
GGTCCAGACTCCTACGGGAGG 15
GAAGGCGGCTCACTGGACCAT 308
GAACTCGGCCGTGAAACGCTA 20
GGCTCAGGACGAACGCTGGCG 18
TTTACACACCAACTTCGATCC 14
AAGTCCCGCAACGAGCGCAAC 95
CTATGTACGGACGGGATAACC 11
AAGTGAGAATGCTGACATGAG 115
ACGCTTGATAAGCGTGGGGTC 17
ATGCTGACATGAGTAACGATA 22
AGGCCGAAAGGCGTAGTCGAT 27
CGCAGCGAAAGCGAGTCTGAA 22
TAGGGGAACCTGCGGCTGGAT 119
ACCAAACTCCGAATACCGGGA 11
GCGGACACGTTTCTTGGTAAG 80
TAGTTTGACTGGGGCGGTCGC 272
GGATGGACGTATCTCTGGTGG 736
GCGCGCGATGGTAGGCTCAGA 1884
TGGCTCAGAGCGAACGCTGGC 10
TGGCACAGACCAGGGGGTAGC 11
GACATCCCGGTCGCGGTTAGT 65
GTCTTGAGTATGGTAGAGGTG 22
ATCTAAGGTCCCTAAGTTATG 41
TGTACCGGGGCTCAAGCCATA 670
CCGTGGGTGTAGGAATATTGA 56
ATATTCGGAGGAACACCAGTG 926
GGGGTAGAGCAGCCCGGTAGC 10

ACGGAGGCGCTCGATGGTAGG 10
ACTAATAGCTCGATCGACTTG 10
TGAGCAGGGTTAGCTGGCCCC 12
AGCATACCAAGGCGCTTGAGA 10
CCTCGATGTCGACTCATCGCA 27
GCCGTGAAACGCTCCACCGCC 11
GTACCTTTTGTATAATGGGTC 14
CGCCCCGCAAGGGGAGCGGCA 65
GAAGAACACCAGTGGCGAAGG 20
GCAGGTTCAAATCCTGCCCCC 12
GTAACGACTTCCCCGCTGTCT 87
CGCCAATGGTACTTCGTCTCA 39
TTGGCACCTCGATGTCGACTC 49
TCTGTTGCAATAGATCGGGAT 43
TGTGACGCCTGCCCGGTGCTG 10
ACGCAGACTCAGTGAAATTGA 123
CCCGGGGGTAGAGCACTGGAT 233
GCGGAGCGTTCCGTAAGCCTG 22
AGTCGCAAGACGACGTATAGG 17
ACAGCTCACTGGTCTAAATAA 14
GATGGAGCAGCCCGGTAGCTC 15
CCCGGAACTGCCTTTGATACT 245
TAGCCGGCCCCTAAGGCGAGG 24
GAGCAGGGTTAGCCGGCCACT 21
AAAACACAGGGCTCTGCGAAG 119
TGATCCTGGCTCAGAACGAAC 1523
GGGCACTCTAAGGGGACTGCC 704
GCTAACTTCGTGCCAGCAGCC 12
AGAGACTCCCTCGCCGAAAGT 21
AGGATAGGTGGTAGACTTTGA 48
TGAGTATGGTAGAGGTGAGTG 33
GATCGCAGGCCAGTCAGCCTG 34
TGTCGTGAGATGTTGGGTTAA 136
ACCAAACTCCGAATACCGGGG 26
CAAATGAATTGACGGGGGCCC 15
GACGATCCATAGCTGGTCTGA 108
CGAGCGCCCCGCAAGGGGAGC 71
GCGTGGGATCGGAGGTTCAAG 13
ATTGCTCACGGGCCGTACCGC 21
TGACATGAGTAACGATAAAGG 16
CTATAACGGTCCTAAGGTAGC 14
AGGTGCGAAAGCGTGGGGAGC 49
GTTATGGCGGAGCGGCTGCAC 1045
AGTACCCAGAGGAAAGGACAT 501
GGTAGAGGTGAGTGGAATTCC 36
GCATTTGGTGGATGCCTTGGC 47
GCTCCTGCATATAGACCGTAC 555
ACGGCCCAAACTCCTACGGGA 12
CCGAACTCGGCCGTGAAACCC 10
CGTCTCCGTGTTTTACATGGG 18
ACCGATTGTATCTCGAGAAGC 11
AACCACGTTAATATTCGTGGG 10
GTAAAGCGCACGTAGGCGGAC 335
GACGAGGCGCTGACACGGATT 49

GCGAACCTGGGGAACTGAAAT 32
TTGATCACTCCCATTTACAAT 17
CCTGACCACCATCTAAGGTCC 44
TTGATCCTGGCTCAGAACGAA 1334
CATCGTTTACGGCGTGGACTA 12
TGGGCCTGCAGGTGGTGACGG 138
ACGCATGCAGCTTACCGGTAC 219
CCCTAAACCGACACTGGTGGA 534
TGCACGCGTAACTTCGGAAGA 18
TGAAGGCCTTAGGGTTGTAAA 15
GTCGCAAGACGACGTATAGGG 21
GGGACTGAGACACGGCCCAGA 178
AGACTCCGCTAGTAGTGGCGA 14
CGTAGCTCAGCTGGGAGAGCA 6438
CCCACCTTAGATGACTAGAAA 54
GACAGGTGCTGCATGGCTGTC 19
ACAGTGGGCAGCGAGCACGCG 413
CTAGTCATCATAAATAAGGTA 81
CAGGGTAGCTATATACGGACG 23
TACTCGTGCATGACCGATAGC 57
TGGAAGTCTTGAGTATGGTAG 29
GTCTGACGCCTGCCCGGTGCG 11
CTGGACCGGAGACAGGTGCTG 625
CCGGTGGTCCCGCGTGGAAGG 43
GGGGCCGTAGCTCAGCTGGGA 12177
TGGATGGGCTATGGGGACTCA 2143
CGAACCTGGGGAACTGAAACA 16442
CATGCCGCGGTGAATACGTTC 226
TAGGGCGGGACACGTGAAATC 16
ATATCGACGGGGTTGTTTGGC 122
CCATATCGACGGGGTTGTTTG 78
GGTTAGTGGAGACACTATCCT 33
CAGGCCAGTGGCTTTTGTGAA 21
ACCGGAGAAGAAGCCCCGGCT 496
AGCGTGGGGTCGGAGGTTCAA 1253
AACTTCGGAAGAAGCGTGACC 42
TTGTCTGCGGATGGTTCGAGA 13
TATGGGGACTCACCGTCTTAC 93
ACATTCCGCCTGGGGAGTACG 1956
AGACTCCCTCGCCGAAAGTCC 17
TCGTCGGCCCATGTGGGCCGC 20
AATCGGATCAACTGAAGAGTT 15
CCTAACACATGCAAGTCGAGC 19
AGTTGGGTTCAGAACGTCGTG 12
ATACGGACGGGATAACCGCTG 276
GCGAACCTGGGGAACTAAAAC 20
TTCCCCGCTGTCTCCAACGCA 458
CAGACGAGGCGCTGACACGGA 11
ATACGAAGGGGGCTAGCGTTG 29
GCGGCAACGCATGCAGCTTAC 31
GTACCCAGAGGAAAGGACATC 496
CGTAGCTCAGCTGGGAGAGCG 27
ATCCTAACCAAACTCCGAATA 62
GGTACTCCGGGGATAACAGGC 84

GGGGTTGTTTGGCACCTCGAT 193
GCGAACCTGGGGAACTGAAAC 34783
CAGGCGATGAAGGACGTGATA 100
ACGAGGGCGGCGGCGCCGGCA 41
CAACCCCGGAACTGCCTTTGA 48
GATGGGAACCACGTTAATATT 19
GATCTGTCCCTAGTACGAGAG 89
GTACCTGAAACCGGATGCATA 11
AGGCGGACTTTTAAGTCAGGG 24
CGAGTGTAGAGGTGAAATTCG 137
TGGGGACTCACCGTCTTACTG 121
GACCGATAGCGAACCAGTACC 139
AATGCACGCGTAACTTCGGAA 101
GAAGTGGGTGATAGTCCCGTA 90
TGAAACGCTCCAGCGCCAATG 169
AGGTGAAATTCGTAGATATTC 116
CCATTACTAACGCTGAGGTGC 10
GGTAGAGCACTGGATGGGCTA 17
CGTATCTCTGGTGGACCTGTT 47
CCCGCAACGAGCGCAACCCTC 116
GTCGCAAGACTAAAACTCAAA 15
GGGGCCGTAGCTCAGCTGGGG 14
GCGCCAATGGTACTTCGTCTC 53
CGAACCTGGGGAACTGAAACG 62
GTCGTCGGTTCGATCCCGTCC 311
GCCGTAGCTCAGCTGGGAGCG 12
TCCACGCCGTAAACGATGAAT 57
GATAGGCCGGGTGTGGAAGTG 60
AACGTCGTGAGACAGTTCGGT 320
TTGACGGGGGCCCGCACAAGC 190
GCCGCGTGAGTGATGAAGGCC 30
GTGATCCGGTGGTCCCGCGTG 10
CGGGAGGCAGCAGTGGGGAAT 15
GTTTGATCCTGGCTCAGAACG 587
TCACTGGTCTAAATAAGGGTC 38
GGAGGTTCAAGTCCTCCCAGG 41
TGTTTGGTTAGGCGGAAGAGA 33
ACGGTCGCAAGACTAAAACTC 10
GGACATCAAACGAGACTCCGC 344
CTTGGAGATAGCTGGTTCTCC 22
AGGCTGATGACCCCCAAGAGC 17
TAAGGGCATTTGGTGGATGCC 10
GCGGTGAATACGTTCCCGGGC 228
GTAGCTCAGCTGGGAGAGCAC 5940
AACCAAACTCCGAATACCGAG 14
AGTGGTTGACAGGTTGGTTTG 10
TCGTGACGACATGTGTAGGAA 18
GATGAATGTTAGCCGTCGGGG 7214
GGTAGGCTCAGAACGGTCGGA 35
TTGACAGGATCTGTCCCTAGT 306
TCGGAGGAACACCAGTGGCGG 31
AAGCAACGCGCAGAACCTTAC 28
GAAATTCGTAGATATTCGGAA 28
AAAGGACATCAAACGAGACTC 143

TAGCGAAATTCCTTGTCGGGG 11
TCGATCCCGTCCGGCTCCACC 1789
CCAAGTGATCTAGCCATGAGC 95
AGCGAACCTGGGGAACTGAGA 26
ATCGACGGGGTTGTTTGGCAC 282
CAACGCAGACTCAGTGAAATT 102
GGGTTGTTTGGCACCTCGATG 192
GCGCAGAACCTTACCAGCCCT 5954
TACGAAGGGGGCTAGCGTTGT 44
AGGCGACCACGGTAGGGTCAG 2019
TTGAAGGCAATATGGAAGTAG 19
GCTTTACACTGGCATTCGTGA 413
GGATGCCTTGGCATGCACAGG 27
ATGGTGGTGACAGTGGGCAGC 139
CCCTTGACATCCCGGTCGCGG 129
ATACCCTGGTAGTCCACGCTG 30
TAGCCATGAGCAGGTTGAAGG 39
AGGCTGATGACCCCCAAGAGT 4642
GTCGAGTGCAATGGCATAAGC 38
TGCATGAAGTTGGAATCGCTA 39
ACCCTGACCACCATCTAAGGT 30
GTACCTGAAACCGGATGCCTA 5119
GGGTTAAGTCCCGCAACGAGC 15
ACGGTCCTAAGGTAGCGAAAT 73
CCAGGATGTTGGCTTAGAAGC 1549
ATGGGGACTCACCGTCTTACT 89
GAATTTTGGACAATGGGCGCA 38
TGAGCAGGGTTAGCCGGCCAC 24
GGCGATGAAGGACGTGATACG 67
GCATGCACAGGCGATGAAGGA 432
CTGAGGTGCGAAAGCGTGGGG 87
TCCCGGGCCTTGTACACACCG 223
CATCTAAGTACCCAGAGGAAA 165
GGTGGGTAGTTTGACTGGGGC 74
CCAAGAGTCCATATCGACGGC 14
GGGTCAGCGACTTAGTGTATC 198
CGATAAGCGTCGGGGAGGTGC 45
TGAGATGTTGGGTTAAGTCCC 77
CTAAGCGGGAAACCCACCTGA 175
TCACTGGACCATTACTGACGA 26
GGATCTGTCCCTAGTACGAGA 100
GGAGAAGAAGCCCCGGCTAAC 94
GCCGTGAAACGCTCCAGCGGC 34
TGAGTAGGGCGGGACACGTGA 42
CTCAGTTCGGATTGCACTCTG 153
CGGATAAAAGGTACGCCGGGG 1721
GAAGGCCGCAGGTTCAAATCC 17
GCAGCAGTGGGGAATATTGGA 15
CGACGATCCATAGCTGGTCTG 397
TCGTGAGACAGTTCGGTCCCT 295
TCGGAGGAACACCAGTGGCGA 8594
AGTGATCAAGTGTCTTAAGGG 94
TAGGCGCAGCGAAAGCGAGTC 20
GAAATTCGTAGATATTCGGAG 174

CGGATAAAAGGTACGCTGGGG 13
GCGAACCTGGGGAACTGACAC 11
GTTGGGCACTCTAAGGGGACT 1347
GATAGTGAACCAGTACCGTGA 40
AGGTGAGTGGAATTCCGAGTG 62
CGTCGTGAGACAGTTCGGTCC 678
TGGGCTGGGCTACACACGTGC 13
GGTCGTCGGTTCGATCCCGTC 342
AAGCCGTCTCAGTTCGGATTG 26
ATCCTGGGGCTGGAGCAGGTC 511
TGGATGTTTGGTTAGGCGGAA 53
CAGCAGCCGCGGTAATACGAA 11
ACCGGGATGGACGTATCTCTC 40
ACGAAGGGGGCTAGCGTTGTT 528
TTGGGCACTCTAAGGTGACTG 25
ATCAAACTTGGAGATAGCTGG 56
GACCGAAGTGGGTGATAGTCC 255
AACGAACGCTGGCGGCAGGCA 11
TCCAGCCATGCCGCGTGAGTG 15
GGCGCAAGCCTGATCCAGCCA 306
CTTAGTGTATCGAGCAAGCTT 18
GGGTGGAGCAGCTCGGTAGCT 10
TTTGCGGACTTTTACGAAAGT 42
ACGGAAAGACCCCGTGAACCT 12
ACGCGAGTGTGAGCTAATCTC 10
GGCATTTGGTGGATGCCTTGG 43
GGTTAGACGGAAAGACCCCGT 36
CTCTAAGGGGACTGCCGGTGA 129
ATAATGGGTCAGCGACTTAGT 142
GTTGAAGGAACTCGGCAAAAT 95
ATTGCACTCTGCAACTCGAGT 349
TTGCGTCTCCGTGTTTTACAT 27
GGTTGTTTGGCACCTCGATGT 166
GTTCCCGGGCCTTGTACACAC 92
CCGGGGAGTACTAGTCGGCAG 797
AGCTGGTCTGAGAGGATGATC 56
AGTCGTGAGACATCCTGGAGG 27
TGGGGGTGCGACTGATTATAG 38
CTTCTCATGTTTGTGTTCTTC 23
AAGAACACCAGTGGCGAAGGC 14
ATAACTCAGGGAAACTTGTGC 65
CCTTTGATACTGGAAGTCTTG 18
TGACTTGTGGCTAGGGGTGAA 1151
AAGTAGGGCAATAAGGCAATA 10
AGTTTGATCCTGGCTCAGAAC 503
CGTCGGTATCTGGGCTTGTAG 60
CCGGGGCTCAAGCCATACACC 3187
TGAGGGCTTTGGGCAAACATA 10
GTAACTATAACGGTCCTAAGG 10
TCATGGCCCTTACGGGCTGGG 116
CACTGGCATTCGTGACGACAT 211
TTATCGGCAAATGATCGGCCC 133
CGTCGTGAGACAGTTCGGTCT 17
TAGCTATATACGGACGGGATA 77

CGGTCATAGTGATCCGGTGGT 613
GCCCGCGTTGGATTAGCTAGT 93
GAGTGATCAAGTGTCTTAAGG 105
AGAGGTGAGTGGAATTCCGAG 112
CCCAAGGTTTGTCCTGGGTGA 43
ATGGACGTATCTCTGGTGGAC 733
GGCATGAAGTTGGAATCGCTA 14
CCGAACTCGGCCGTGAAACGA 13
GTCATCATAAATAAGGTATCT 10
CTTGTCGGGTAAGTTCCGACC 67
TGAAAAGCACCCCGACGAGGG 35
GATCCTGGCTCAGAACGAACC 13
ATAGTCCCGTACACGTAGAAT 206
GCCTGGGGAGTACGGTCGCAA 1190
CTATGGGGACTCACCGTCTTA 92
GCCAGCAGCCGCGGTAATACG 23
CAGTGGGGAATATTGGACAAT 20
AGAATTGGAAAGAGGCCGGAT 65
TGCGGGGTTCCTGCGGTTAGA 220
ACGTGAAATCCTGTCTGAACA 80
GGGAACTGAAACATCTAAGTA 181
GGTATCGGAAGTGAGAATGCT 17
CGAACCTGGGGAACTAAAACA 14
AGTAGCGTTTGCGTCGGTATC 14
CGCGATGGTAGGCTCAGAACC 23
TCAGCTCGTGTCGTGAGATGT 295
GAGTACGGTCGCAAGATTAAA 111
CCCTGACCACCATCTAAGGTC 40
CGCGCAGAACCTTACCAGCCT 17
ATTCCGAACTCGGCCGTGAAA 999
CGACATGTGTAGGATAGGTGG 1086
CTGATCCAGCCATGCCGCGTG 41
CAGCGAAAGCGAGTCTGAACA 17
TAGGGGTTAGAAGCGAACCTG 326
AATCCTGTCTGAACATGGGTC 1104
AAAAGCCGTCTCAGTTCGGAT 25
CCTATCTGCCGTGGGTGTAGG 159
CAGTTGGAGCCCAAGGTTTGT 77
TGAAGGAACTCGGCAAAATGC 196
GTGCCACCCCCCTATGGTTGC 15
CACGTACTGGAGGACCGAACC 6978
TGGGGTCGGAGGTTCAAGTCC 656
TAGGTCGCTGCCAGGTCTGCA 13
ATTACTGACGCTGAGGGGCGA 35
GAGGGCGGCGGCGCCGGCAGC 40
TATACGGACGGGATAACCGCT 157
GGGGTCGGAGGTTCAAGTCCT 438
ATGAACTTTGGCGGACACGTT 12
ATGGGCTATGGGGACTCACCG 1236
CCGTCGGGGTGTTTACACTTC 126
AGGTAGAGCGTCGACCGAATA 340
CGGCAAATGATCGGCCCGCGT 2515
GGCCGTAGCTCAGCTGGGAAA 12
GAGGGCATGAAGTTGGAATCG 13

GTTCGGATTGCACTCTGCAAC 55
GCCTTAGGGTTGTAAAGCTCT 30
TACAGGTGCTGCATGGCTGTC 12
ACCAAAAACACAGGGCTCTGC 56
TGCGTCTCCGTGTTTTACATG 27
CTTAAGCCGGTAGGTGTAGGC 17
CGCGCAGAACCTTACCAGCCC 6852
CATTACTAACGCTGAGGTGCG 10
TGGAAGTAGGGCAATAAGGCA 28
TTGGAGCAACGCTGGATGGGT 25
TCGCGTGTGTTGTGAGGTCTT 75
AAGGGCATTTGGTGGATGCCT 18
GCTGTCTCCAACGCAGACTCA 40
CTGACCACCATCTAAGGTCCC 36
ACCCGAAGGCGCTGTGCTAAC 194
GCTTGATAAGCGTGGGGTCGG 41
CGACGATTTCCGAATGGGGAA 19
CACGTACTGGAGGACCGAACT 11
CAAGGGGAGCGGCAGACGGGT 20
AAGGGACAGTCGTGAGACATC 38
AAGGTAGCCGTAGGGGAACCT 14
GATGTCTAACTGCGGCCCGTT 57
TCGGCCGTGAAACGCTCCAGA 21
GGGGCCCGCACAAGCGGTGGA 208
GAATACCCCCGGGGGTAGAGC 51
AGTCTGCCTGTTCTGTATGAA 13
CGAGGGCATGAAGTTGGAATC 13
CTTAAGGGCATTTGGTGGATG 10
CGCTTGAGAGAACTGCGTTGA 760
GCCATCGCTCAACGGATAAAA 80
GTTGCAATAGATCGGGATGAC 54
TGGTAGACTTTGAAGCAGGGG 262
TCCGGGTCCAGGACCGTGTAT 31
GAAAGGCGTAGTCGATGGGAA 207
AGATATTCGGAGGAACACCAG 1595
AACCTTACCAGCCCTTGACAT 119
GGGCCATCGTTCAACGGATAA 16
AGGTGGTGCATGGCTGTCGTC 11
ATAGTGATCCGGTGGTCCCGC 540
CTCATCGCATCCTGGGGCTGG 138
CAGGCGACCACGGTAGGGTCA 2026
ATGTCTAACTGCGGCCCGTTA 22
GTGCGGCTGGATCACCTCCTT 37
CGTAGATATTCGGAGGAACAA 11
GGGGTAGAGCACTGGATGGGC 77
TTGCACTCTGCAACTCGAGTG 191
CACGGCCCAGACTCCTACGGG 5126
GCATGAAGCTTACCGGTACTA 76
GTAGCTCAGTTGGTTAGAGCA 114
ATCCCAAAACAACCAGGATGT 53
TATAGACCGTACCCTAAACCG 1581
TTTACCCGAAGGCGCTGTGCT 320
GGATGTGAGGATCCCAAAACA 21
ACGTGCTGGGCTACACACGTG 11

CTCAGAACGGTCGGAAATCGT 514
GCCGGGTGTGGAAGTGCGGCA 438
CTTGGTGGGCCTGGGAGGACT 14
CCGTCTCAGTTCGGATTGCAC 10
GTGACGGATCGCGTGTGTTGT 40
TGGCATGCACAGGCGATGAAG 303
TGGGGAGTACGGTCGCAAGAT 43
CGCAAGGAGGCAGGCGACCAC 21
CGTATAGGGCCTGACGCCTGC 11
TCAACTGGAGAGTTTGATCCT 10
GGACCATTACTGACGCTGGGG 17
GTCCCGTACACGTAGAATAGC 431
TAACGGTCCTAAGGTAGCGAA 52
GCACGTAGGCGGACTTTTAAG 72
CCTTGACATCCCGGTCGCGGT 116
CAACTTGAGAGTTTGATCCTG 25
TGGTTGGATGTTTGGTTAGGC 33
AAAAGGTACCCCGGGGATAAC 75
TCTGTCCCTAGTACGAGAGGA 105
GATATTCGGAGGAACACCAGA 38
ATCGCTAGTAATCGCGGATCA 277
TGAGAGTTTGATCCTGGCTCA 769
TATCTGCCGTGGGTGTAGGAA 20
AATGAGAGTGATCAAGTGTCT 1593
AGCCCTTGACATCCCGGTCGC 86
CGACACTGGTGGACTGGTAGA 406
GCAATGGCATAAGCCTGCCTG 64
GTGGATGCACGTATGTGCGTG 344
TGCGGTTAGACGGAAAGACCC 50
ACTATAACGGTCCTAAGGTAG 12
AGACACGGCCCAGACTCCTAC 201
TTTGGCACCTCGATGTCGACT 93
GGGGAATATTGGACAATGGGC 391
GGGGAGTACTAGTCGGCAGAC 22
TACGGTAACACGTACTGGAGG 62
GGTAAAGGCTCACCAAGGCGA 33
ACGGGCTGGGCCACACACGTG 10
TATCGACGGGGTTGTTTGGCA 197
ACGGGAGGCAGCAGTGGGGAA 30
GCGGGGTGGAGCAGCTCGGTA 10
CGTAACGACTTCCCCGCTGTC 95
ACACGTAGAATAGCAGAAGTC 589
GACTGCAAGACTGACAAGTCG 47
GGCAACGCATGAAGCTTACCG 11
GGGTCGACCACGATCCAAGCC 941
CAGACTCCTACGGGAGGCAGC 591
CATTCCGCCTGGGGAGTACGG 2019
AGTGCAATGGCATAAGCCTGC 21
GGTCTGAGAGGATGATCAGCC 10
GATATTCGGAGGAACACCAGG 22
ACGTACTGGAGGACCGAACCG 12
GAATACCGGGGAGTACTAGTC 38
CGGCTGCACCCGATCCCATTC 14
ACGGAGGCGCGCGACGGTAGG 10

GGAAGATCGAGAATTGGAAAG 1097
GGAACACCAGTGGCGAAGGCG 563
TTACTGACGCTGAGGTGCGAA 7203
CGGTGAATACGTTCCCGGGCC 163
ATGTGCCCTTCGGGGGAAAGA 10
ACGCCGGGGATAACAGGCTGA 24
CACGATCCAAGCCTAAGTACT 41
TTCCGAATGGGGAAACCCACC 18
TTTGCAAGCAGGGGGTCGTCG 53
ACTTGAGAGTTTGATCCTGGC 2618
GTGAGTAACGCGTGGGAACGT 18
TTACACTGGCATTCGTGACGA 416
GAGGCGCGCTATGGTAGGCTC 24
GAGGCGCGCGATGGTAGGGTC 25
GAGAGGACCGGGATGGACGTA 1829
GGCGACGATCCATAGCTGGTC 68
GTTAGACGGAAAGACCCCGTG 76
ATGGGCTGGGCTACACACGTG 12
CGACTTGATCACTCCCATTTA 273
CAGAACCTTACCAGCCCTTGA 1031
CACGCCGTAAACGATGAATGT 135
GCCGTGAAACGCTCCAGCGCA 28
TAACACATGCAAGTCGAGCGG 13
TCACTGGACCATTACTGACCC 16
CTGCCTGACTGCAAGACTGAC 79
CTGGCATTCGTGACGACATGT 151
TTTAGATCGTCGGTTCTTTGA 17
GTCGGAAATCGTTCGTCGAGT 210
CCTAAGTACTCGTGCATGACC 345
CGGCTGGATCACCTCCTTTCT 167
TTGGTTTTACCCGAAGGCGCT 197
CACGGGCCGTACCGCAGCTGA 98
GATGGACGTATCTCTGGTGGA 723
ACGAGACTCCGCTAGTAGTGG 17
GTGATGAAGGCCCTAGGGTTG 64
CCACACTGGGACTGAGACACC 12
CTGCGTTGAAGGAACTCGGCC 23
ATCGGAAGTGAGAATGCTGAC 57
GAGCAGGTTGAAGGTACGGTA 531
GGGCTGGGCTACACACGTGAT 68
GTGTAGGATAGGTGGTAGACC 28
TATCGGAAGTGAGAATGCTGA 21
TGGTGGACTGGTAGAGAATAC 2436
GGGTGAAGTCGTAACAAGGTA 50
CGATCGACTTGATCACTCCCA 366
AAGGCGCTTGAGAGAACTGCG 47
GAACCTGGGGAACTGAAACAC 121
TTGGAAAGAGGCCGGATTTAT 10
ACCGCAAGGAGGCAGGCGACC 20
TGGAGATAGCTGGTTCTCCGC 20
ACCATTACTAACGCTGAGGTG 10
TTCGTCGAGTGCAATGGCATA 40
GATGTTTGGTTAGGCGGAAGA 46
TCCGAACTCGGCCGTGAAACC 46

TTTACACTGGCATTCGTGACG 409
CGGGATGGACGTATCTCTGGT 848
GTCCAGGACCGTGTATGGTGG 10
TGACTGCAAGACTGACAAGTC 72
GCAGGGTTAGCCGGCCCCTAA 1094
CGAAGGCGGCTCACTGGACCA 342
CCTGAAGGCCGCAGGTTCAAA 17
GAGCAGGGTTAGCTGGCCCCT 11
TGATGAGGGGCCGTAGCTCAG 139
ACACCGAAGCTGTGGATGCAC 276
CGTGCTGGGCTACACACGTGC 10
TATGGCTAAGTGGGAAAGGAT 131
GAATCGCTAGTAATCGCGGAT 1455
GTGTAGGATAGGTGGTAGACT 272
AACTCAAATGAATTGACGGGG 12
GTGACTGCGTACCTTTTGTAT 10
TGTTAGCCGTCGGGGTGTTTA 68
CAAGCAGGGGGTCGTCGGTTC 402
GTCAGGGGTGAAATCCCGGGG 36
TAACGTCCGTCGTGGAGAGGG 26
AGGCGCGCGATGGTAGGCTCC 58
ACTCGGCAAATTGCACGCGTA 10
TCTCAAGACGCGGGAGAGTAG 217
TGAATGTTAGCCGTCGGGGTC 10
TGGACGTATCTCTGGTGGACC 705
ATTTTGGACAATGGGCGCAAG 60
ACCCTTTGATCCGACGATTTC 1244
TAGCCGTCGGGGTGTTTACAC 199
TGTAATCGGATCAACTGAAGA 10
CTGATCCTAACCAAACTCCGA 103
GGTTCAGAACGTCGTGAGACA 120
GGTCCAGGACCGTGTATGGTG 35
GGAGTTGGTTTTACCCGAAGG 25
AACGCTGGCGGCAGGCTTAAC 1746
CAGCTTACCGGTACTAATAGC 47
GATAGGTGGTAGACTTTGAAG 42
CTGGGCCGTGAAACGCTCCAG 13
GGTTTTACCCGAAGGCGCTGT 130
TAGGTAGAGCGTCGACCGAAA 13
GAACCTGGGGAACTGAAACAT 7477
CAGTCAGCCTGACGATCGCTT 20
GCCTGTGAAGGGACAGTCGTG 21
CCAAACTCCGAATACCGGGGA 26
TACACTGGCATTCGTGACGAC 420
CGCTGTCTCCAACGCAGACTC 782
TTGCGGGGGCAGGATTTGAAC 39
GCAGGCGACCACGGTAGGGTC 2079
ACCGGGATGGACGTATCTCTA 13
GATGAGGGGCCGTAGCTCAGC 139
GGATGGAGCAGCCCGGTAGCT 15
ACATGCAAGTCGAGCGCCCCG 20
GCCCCAGTAAACGGCGGCCGT 27
AACGAACGCTGGCGGCAGGCC 15
GGAGAACTGAAACATCTAAGT 10

GGCAGACACACGGCGGGTGCT 201
CGAGTATTCCCTATCAGAGCC 13
AGCGCCAATGGTACTTCGTCT 43
AGCGTTTGCGTCGGTATCTGG 10
TGGTGGGGTAAAGGCTCACCA 26
TTGGAGCCCAAGGTTTGTCCT 117
GCTCGATCGACTTGATCACTC 75
CTGACTGCAAGACTGACAAGT 69
GTGAGGGAAAGGTGAAAAGAA 11
CTTCGTCTCAAGACGCGGGAG 33
AAAGGTACCCCGGGGATAACA 71
AGGACCGGGATGGACGTACCT 10
TACCCGAAGGCGCTGTGCTAA 182
CGGCCGTGAAACGCTCCAGAG 10
CTAGTAGTGGCGAGCGAACGC 58
GATCCTGGCTCAGAACGAACG 1365
GTAGCGAAATTCCTTGTCGGG 786
GGTGGTTATGGCGGAGCGGCT 2798
AAAGCACCCCGACGAGGGGAG 31
GTTGGGCACTCTAAGGTGACT 26
AATTGACGGGGGCCCGCACAA 22
ATAGCGAACCAGTACCGTGAG 100
ACGGATCGCGTGTGTTGTGAG 655
TGGGGTGAAGTCGTAACAAGG 34
GCCGTACCGCAGCTGACGCTG 24
CTGGGGAGTACGGTCGCAAGA 127
AACGAACGCTGGCGGCAGGCT 3235
ACCGGGATGGACGTATCTCTG 1235
AATCGCTAGTAATCGCGGATC 1398
GATAAGCGTCGGGGAGGTGCG 31
TAACTTCGTGCCAGCAGCCGC 20
CGCTGTGCTAACCGCAAGGAG 26
AGGGCATGAAGTTGGAATCGC 13
AAGGCATCTAAGCGGGAAACC 27
CCCGCGTGGAAGGGCCATCGC 10
GTGAGATGTTGGGTTAAGTCC 92
GCCTGGGGAGTACGGTCGCAC 19
CTTGGGGGTCATCAGCCTGTT 13
AGAGAATACCAAGGCGCTTGA 66
GGCCCTAGGGTTGTAAAGCTC 7146
TTTATCGGCAAATGATCGGCC 79
GATCCTGGCTCAGAACGAACA 19
GCACAGGCGATGAAGGACGTG 239
AGTTAGGCTGGACCGGAGACA 27
AATCTGAGCAGGGTTAGCCGG 215
CCGAACTCGGCCGTGAAACGC 8123
GCGACCACGGTAGGGTCAGCG 2125
CGACTGTTTATCAAAAACACA 12
TACCGTATGTGCCCTTCGGGG 13
TAGGTGGTAGACTTTGAAGCA 77
CGGATAAAAGGTACTCCGGGG 43
GGTAGAGCGTCGACCGAATAC 466
GCGCTTGAGAGAACTGCGTTG 761
GGCTAACTTCGTGCCAGCAGC 12

GAGGCCGAAAGGCGTAGTCGA 13
AAGCGTGACCTCACTATGGGC 224
TCGGAGGTTCAAGTCCTCCCA 42
GGGGTCGTCGGTTCGATCCCG 333
CACGTACTGGAGGACCGAACA 34
GTTGTAAAGCTCTTTCACCGG 392
ACGAGGGGAGTGAAACAGTAC 18
CTGACGAGCTACCGGGCTGCT 23
CGGCCCGCGTTGGATTAGCTA 256
ACTCCTACGGGAGGCAGCAGT 91
GGGCTCTGCGAAGTCGCAAGA 803
AAGGATGTGAGGATCCCAAAA 12
CATTCCGAACTCGGCCGTGAA 42
TTCTAGTCATCATAAATAAGG 422
CACTGGATGGGCTATGGGGAC 1247
ACACAGGTGCTGCATGGCTGT 38
TCCAAGCCTAAGTACTCGTGC 47
GGAGGCGCGCGATGGGAGGCT 10
TAGGTCGCTGCCAGGTCTGCC 32
GGCAAAGAACAGGCGCAGCCC 10
ACGAGCTGGGCTACACACGTG 10
TGGGGTCGGAGGTTCAAGTCA 23
CGTTGTTCGGATTTACTGGGC 344
AGTGAAATTGAATTCCCCGTG 34
GACGGGGTTGTTTGGCACCTC 496
GGGGCTAGCGTTGTTCGGATT 790
TTGGCATGCACAGGCGATGAA 297
AGTGGCGAGCGAACGCGGACC 114
TGGATGCCTTGGCATGCACAG 18
CCCGGGCCTTGTACACACCGC 251
TGAGAGGATGATCAGTCACAC 13
CACGTACTGGAGGACCGAACG 25
TCCTGCGGTTAGACGGAAAGA 32
CACCGTCTTACTGATCCTAAC 147
CTGTCTCCAACGCAGACTCAG 34
CCATACACCGAAGCTGTGGAT 31
GCTGGGCTACACACGTGCTAC 12948
AACCAGTACCGTGAGGGAAAG 78
CGCGCAGAACCTTACCAGCCA 10
TAAGCGGGAAACCCACCTGAA 163
TCGGCAAATGATCGGCCCGCG 2529
GTTTTACCCGAAGGCGCTGTG 184
CGCGATGGTAGGCTCAGAACG 776
GGAGCAGCCCGGTAGCTCGTC 252
GTACGTGAGTTGGGTTCAGAA 91
TCCTGTCTGAACATGGGTCGA 2104
ATAAGCGTCGGGGAGGTGCGA 316
TCGGCCGTGAAACGCTCCAGC 36830
ACCACCACGTTGATAGGCCGG 157
TGAGAGTGATCAAGTGTCTTA 558
TTTAGAACGTCGTGAGACAGT 10
GTGGGCAGCGAGCACGCGAGT 243
CTCAAATGAATTGACGGGGGC 16
ATACGGCCCAGACTCCTACGG 18

CAAACGAGACTCCGCTAGTAG 25
CAGGGGGTAGCGACTGTTTAC 1931
TTCGGGGTGGATCTGTGGATC 40
GGGGTCGGAGGTTCAAGTCCA 12
GCAACGCGCAGAACCTTACCA 36
CGGAGGCGCGCGATGGTAGAC 20
GTAACTTCGGAAGAAGCGTGA 11
AGCCGAGAGGAAGGTGGGGAT 39
CGTAGATATTCGGAGGAACAC 7033
CCGCGTGAGTGATGAAGGCCC 22
GATCGTCGGTTCTTTGAAAAC 16
CACCAAGGCGACGATCCATAG 344
AGCAAGCTTAAGCCGGTAGGT 98
CTTACGGGCTGGGCTACACAC 866
CTCCAGCGCCAATGGTACTTC 19
CGTCAGCTCGTGTCGTGAGAT 263
CTGAGAGGATGATCAGCCACA 131
ATGGGCGCAAGCCTGATCCAG 129
CTCAGTGAAATTGAATTCCCC 44
GTACGCCGGGGATAACAGGCT 100
GATATTCGGAGGAACACCAGC 23
ACGTACTGGAGGACCGAACCC 6255
ACAGGTGCTGCATGGCTGTCG 703
GGAGATAGCTGGTTCTCCGCG 25
CTGAGAGGATGATCAGTCACA 12
GGTGCTGCATGGCTGTCGTCA 858
GAGATCGAACTGTCACAATGA 10
CAGACTCCTACGGGAGGCAGG 15
GACCGGGATGGACGTATCTCT 4826
CCCTAGGGTTGTAAAGCTCTC 20
TCGTAGATATTCGGAAGAACA 44
GCAGACTCAGTGAAATTGAAT 94
CTGCCTTTGATACTGGAAGTC 28
CCGGCTAACTTCGTGCCAGCA 20
TAACACATGCAAGTCGAGCGC 17
CCACGGTAGGGTCAGCGACTG 2102
GCCGTGAAACGCTCCAGCGCT 11
TGGTTGTCGTCAGCTCGTGTC 22
TAGCGTTGTTCGGATTTACTG 19
GAGTTGGTTTTACCCGAAGGC 32
AAGAAGCCCCGGCTAACTTCG 27
CGTAGAATAGCAGAAGTCCTT 1449
AGCGAACCTGGGGAACTGACA 20
TCGGGTAAGTTCCGACCTGCA 21
TAATCGGATCAACTGAAGAGT 18
CAAGGTTTGTCCTGGGTGACA 33
AGGTACCCCGGGGATAACAGG 65
GATATTCGGAGGAACACCAGT 1532
CCAAGCCTAAGTACTCGTGCA 115
ACTGTTTAGCAAAAACACAGG 13
CCCTAGGGTTGTAAAGCTCTT 2989
ACTAGTCGGCAGACACACGGC 193
TTACCCGAAGGCGCTGTGCTA 225
AGGGTCAGCGACTGGGGTGAA 350

CGTGGGCCTGCAGGTGGTGAC 498
AGCTCTTTCACCGGTGAAGAT 31
AACAGTTGGAGCCCAAGGTTT 15
TCGCTGCCAGGTCTGCAAAGC 10
GTAACAATAACGGTCCTAAGG 13
ATACCCCCGGGGGTAGAGCAC 358
AGCGACTGTTTAGCAAAAACA 12
GATGAAGGACGTGATACGCTG 41
GTCAGCCTGACGATCGCTTGC 18
GAGAGGACCGGGATGGACGTC 12
TAGAGAATACCAAGGCGCTTG 61
GCAGTTTGACTGGGGCGGTCG 21
GAGGATCCCAAAACAACCAGG 54
ATTCGTAGATATTCGGAGGAA 1483
GATTAAAACTCAAAGGAATTG 67
AGACTTTGAAGCAGGGGCGCC 931
CCTACGGGAGGCAGCAGTGGG 55
GAAGAGAAGATGTAATCGGAT 18
GGTTCAAGTCCTCCCAGGCCC 35
AGGCTGGACCGGAGACAGGTG 636
TCAGAGCCGTGGAAGACCACC 65
GCCGTGAAACGCTCCAGCGCC 33401
GGTGAAATTCGTAGATATTCG 192
GCATGACCGATAGCGAACCAG 136
AAGTGATCTAGCCATGAGCAG 103
CGACGTATAGGGTCTGACGCC 740
TCCTGGGGCTGGAGCAGGTCC 480
CTGCGTTGAAGGAACTCGGCA 1173
TCCAGACTCCTACGGGAGGCA 14
TTAGATCGTCGGTTCTTTGAA 16
AGCTCGTCAGGCTCATAACCT 36
GGCTTGTAGCTCAGTTGGTTA 117
TGAATGTTAGCCGTCGGGGTG 2977
TCGGGGTGGATCTGTGGATCG 13
CCCAAAACAACCAGGATGTTG 99
CGCTAGTAGTGGCGAGCGAAC 55
AGGCGCGCGATGGTAGGCTCG 92
TATTCGGAGGAACACCAGTGG 1242
TTTGAAGCAGGGGCGCCAGCC 547
GGCGGCTCACTGGACCATTAC 1136
CAAGCAGGGGGTCGTCGGTTG 12
GACTGCCGGTGATAAGCCGAG 1100
GGGGCTCAACCCCGGAACTGC 14
AGTGTAGAGGTGAAATTCGTA 84
GTGTAGGATAGGTGGTAGACA 72
GAGACAGTTCGGTCCCTATCT 153
TACACCGAAGCTGTGGATGCA 174
ATTGACGGGGGCCCGCACAAG 143
GCCGGCCCCTAAGGCGAGGCC 16
GCATTAAACATTCCGCCTGGG 626
TGAGTAACGCGTGGGAACGTA 16
GGTGGTAGACTTTGAAGCAGG 135
TTTCCGAATGGGGAAACCCAC 19
TGGTTATGGCGGAGCGGCTGC 937

CGGAAGAAGCGTGACCTCACT 450
AAGATCGAGAATTGGAAAGAG 902
CGTCGAGTGCAATGGCATAAG 35
CCGCAACGAGCGCAACCCTCG 114
AGGGGAGTGAAACAGTACCTG 18
CCACGTTAATATTCGTGGGCC 10
AGGCGGCTCACTGGACCATTA 629
AAGCCTAAGTACTCGTGCATG 133
GAACCTGGGGAACTGAAACAA 111
GACTGACAAGTCGAGCAGAGA 23
AGGATCTGTCCCTAGTACGAG 97
TAGGTAGAGCGTCGACCGAAT 284
TAACAATAACGGTCCTAAGGT 20
AACAACCAGGATGTTGGCTTA 15
CGAACCTGGGGAACTGAAAGA 14
CCCCGTGCACCTTTACTATAG 13
ATAGCAGAAGTCCTTGAGTAG 79
TAAGCCTGTGAAGGGACAGTC 13
GGGCTGGGTTACACACGTGCT 10
AGTCGATGGGAACCACGTTAA 21
GGGGTAGCGACTGTTTACCAA 368
ACTTGGCCGTGAAACGCTCCA 13
CAAGCAGGGGGTCGTCGGTTA 13
TGGCTAAGTGGGAAAGGATGT 118
GACTGCCGGTGATAAGCCGAA 18
CTGGTAGTCCACGCTGTAAAC 22
AGGGAGTGAGAGACTCCCTCG 41
AGGGGGCTAGCGTTGTTCGGA 1009
GCCTACAAACAGTTGGAGCCC 17
GTGTAGGATAGGTGGTAGACG 46
GAGACACGGCCCAGACTCCTA 182
CTGCGTTGAAGGAACTCGGCG 23
GGTAGTCCACGCCGTAAACGA 77
TAGCTGGTCTGAGAGGATGAT 80
CCACACTGGGACTGAGACACG 630
CATCCCGGTCGCGGTTAGTGG 55
AGTACCTGAAACCGGATGCAT 16
CAGGATCTGTCCCTAGTACGA 114
TGTCTGAACATGGGTCGACCA 322
AATAGCTCCTGCATATAGACC 29
GAAAGCGAGTCTGAACAGGGC 37
TGAATGTTAGCCGTCGGGGTA 31
CAAAGGAATTGACGGGGACCC 14
CTAGTTGGTGGGGTAAAGGCT 43
AGGCGCGCGATGGTAGGCTCA 10960
CCACCAGGTCGGCGAAGAACA 25
GGGAGTGAGAGACTCCCTCGC 41
TCGGAAGTGAGAATGCTGACA 127
ACTCGGCCGTAAAACGCTCCA 11
GCAACCCTCGCCCTTAGTTGC 10
GTTCGGTCCCTATCTGCCGTG 1036
TCCGAACTCGGCCGTGAAACG 7625
CGTCGACCGAATACCCCCGGG 5360
AGGTGCTGCATGGCTGTCGTC 780

ACCTGACTTTGGTTTTCGGAT 78
ACCCCCGGGGGTAGAGCACTG 447
CCGCCTGGGGAGTACGGTCGC 3609
TAGGCTGGACCGGAGACAGGT 700
ACTGAGACACGGCCCAGACTC 666
AGTTGGTGGGGTAAAGGCTCA 39
GTCCAGACTCCTACGGGAGGC 12
GAACCTGGGGAACTGAAACAG 14
TTTGGCACCTCGATGTCGGCT 13
GCATTCGTGACGACATGTGTA 33
TCGCTCAACGGATAAAAGGTA 168
TGTATGGTGGGTAGTTTGACT 14
CGACTCATCGCATCCTGGGGC 508
AACTTGGAGATAGCTGGTTCT 173
GCTCACTGGACCATTACTGAG 27
GTGCATGAAGTTGGAATCGCT 39
ATCCATAGCTGGTCTGAGAGG 21
CGGTACTAATAGCTCGATCGC 11
AACAGGCTGATGACCCCCAAG 392
ACGAAGGCGCGCGATGGTAGG 13
GTTGCGGGGGCAGGATTTGAA 35
GTAATCGCGGATCAGCATGTC 20
AAATGTACCGGGGCTCAAGCC 131
GGACCATTACTGACGCTGAGG 17878
GGGCCTGCAGGTGGTGACGGA 137
TAGTCGGCAGACACACGGCGG 188
GCTACACACGTGCTACAATGG 41
CTCGAGAAGCTGGTCTTTCTG 311
ACGAACGCTGGCGGCAGGCTC 16
CAAGCCTAAGTACTCGTGCAT 116
GCCGTCGGGGTGTTTACACTT 165
AGACTCCTACGGGAGGCAGCA 208
TTAGGGTTGTAAAGCTCTTTC 13
GAATATTGGACAATGGGCGCA 532
ATAGGGTGTGACGCCTGCCCG 11
CGGAATCGCTAGTAATCGCGG 81
AGGCAGGCGACCACGGTAGGG 150
CGAAGGCGCTGTGCTAACCGC 55
AAAATGTACCGGGGCTCAAGC 13
ACTCATCGCATCCTGGGGCTG 204
CCTGCGGTTAGACGGAAAGAC 55
GGTGGACTGGTAGAGAATACC 2304
GGATGGGCTATGGGGACTCAC 2504
CGGCCGTGAAACGCTCCGGCG 10
GATAAGCGTGGGGTCGGAGGT 54
CATCGCTCAACGGATAAAAGG 90
AGGTAGCGAAATTCCTTGTCG 372
GTCTCCAACGCAGACTCAGTG 22
CATAAGCCTGCCTGACTGCAA 187
CGACTTCCCCGCTGTCTCCAA 205
CCTTGTCGGGTAAGTTCCGAA 20
TCCCTAGTACGAGAGGACCGG 36
GAGGCGCGCGATGGTAGGATC 47
AACCTGGGGAACTGAAACATA 61

GCTCACTGGACCATTACTGAA 199
AAGTCTTGAGTATGGTAGAGG 29
CACCAGGTCGGCGAAGAACAC 25
CGGGCCGTACCGCAGCTGACG 116
AGAAGCGTGACCTCACTATGG 142
TGAAACATCTAAGTACCCAGA 54
CTTTCTAGTCATCATAAATAA 50
ATGACGGTAACCGGAGAAGAA 67
TAACGATAAAGGGAGTGAGAG 112
TTGGGACTGAGACACGGCCCA 27
CTGTGCTAACCGCAAGGAGGC 27
GGACAATGGGCGCAAGCCTGA 525
ATAAGCCTGCCTGACTGCAAG 219
CTAGTCGGCAGACACACGGCG 194
ACACTGGGACTGAGACACGGC 1073
AGGAAGATCGAGAATTGGAAA 34
TAGGGTCAGCGACTGGGGTGA 272
AGGTTTGTCCTGGGTGACAGC 32
CATTACTGACGCTGAGGTGCC 13
CACCAACTTCGATCCGAAAAC 42
AGACGAGGCGCTGACACGGAT 56
GGACCATTACTGACGCTGAGA 13
TAAGGTAGCGAAATTCCTTGT 151
TGGCTCAGGACGAACGCTGGC 23
ACGAACGCTGGCGGCAGGCTT 5598
GAGTAGTTGGGGGTGGTTTTC 11
CGCTAGTAATCGCGGATCAGC 86
TCCCATTCCGAACTCGGCCGT 131
AAGTCGAGCAGAGACGAAAGT 34
GTGAAAAGCACCCCGACGAGG 35
AAGCCATACACCGAAGCTGTG 29
GGGGACTGCCGGTGATAAGCC 3320
GCGTCGACCGAATACCCCCGT 11
TTTGACTGGGGCGGTCTCCTC 28
GAAGGCGCTGTGCTAACCGCA 19
AATATTCGTGGGCCTGCAGGT 72
GCTGGTTCTCCGCGAAATCTA 12
AAGGGGAGCGGCAGACGGGTG 12
TGGGCGGCATTGTCTGCGGAT 5660
GGACCATTACTGACGCTGCGG 12
CGGTCGCAAGACTAAAACTCA 10
ACTGCCTTTGATACTGGAAGT 38
AGCGAACCTGGAGAACTGAAA 13
CACGGATTTGACCTTCGGGTT 29
CGACGGGGTTGTTTGGCACCT 563
TCAGTTGGGCACTCTAAGGGG 1596
TGGAATTCCGAGTGTAGAGGT 526
GTAACACGTACTGGAGGACCG 146
TTGTGGCTAGGGGTGAAAGGC 821
ATGCACGCGTAACTTCGGAAG 181
GGTTCGATCCCGTCCGGCTCC 657
GGGTAGTTTGACTGGGGCGGT 127
CGAGAGGAAGGTGGGGATGAC 103
GCAAGCTTAAGCCGGTAGGTG 82

CCGTAGCTCAGCTGGGAGAGG 13
TAAGCCGGTAGGTGTAGGCGC 58
AATCGCGGATCAGCATGCCGC 28
GAGAGTTTGATCCTGGCTCAG 469
CTTGTGGCTAGGGGTGAAAGG 938
AACGATGAATGTTAGCCGTCG 2993
TAAAAGGTACCCCGGGGATAA 72
CGTGAAGATGCGGGGTTCCTG 56
CAAAAACACAGGGCTCTGCGA 64
GATGACTTGTGGCTAGGGGTC 11
CTACAATGGTGGTGACAGTGG 11
AGCGTCGGGGAGGTGCGAATA 520
GCCCCGCAAGGGGAGCGGCAG 65
GGAGGCGCGCGATGGTAGGGT 26
GTAGACTTTGAAGCAGGGGCG 787
GGCTCATCGCATCCTGGGGCT 13
CCTGGTGGTTATGGCGGAGCG 3147
GCAATAGATCGGGATGACTTG 241
CGGAAGAACACCAGTGGCGAA 42
ATACCAAGGCGCTTGAGAGAA 90
AGGGGTTAGAAGCGAACCTGG 693
CGCTTGATAAGCGTGGGGTCG 28
ACAGTTGGAGCCCAAGGTTTG 80
GTGGAATTCCGAGTGTAGAGG 540
GGAATATTGACAGGATCTGTC 185
TGGGCGGCATTGTCTGCGGAC 11
TGTTTGGCACCTCGATGTCGA 150
AAGTCTGCCTGTTCTGTATGA 11
AGCCCCGGCTAACTTCGTGCC 160
CTGACGCCTGCCCGGTGCTGG 786
GCAACCCACCTTAGATGACTA 23
CTGGCTCAGGACGAACGCTGG 20
GTACGAGAGGACCGGGATGGA 285
CGATGCATTAGACCCGAAACC 24
ACACGTACTGGAGGACCGAAC 7952
TGTATCTCGAGAAGCTGGTCT 19
ACCACGTTGATAGGCCGGGTG 131
TACACGTAGAATAGCAGAAGT 84
GAGAATACCAAGGCGCTTGAG 70
ACCACCAGGTCGGCGAAGAAC 41
TAGTAATCGCGGATCAGCACG 11
GCCTGATCCAGCCATGCCGCG 124
CCTTACCAGCCCTTGACATCC 111
AGTCGGAATCGCTAGTAATCG 245
CCGTAGCTCAGCTGGGAGAGA 34
CCAGGCCAGTGGCTTTTGTGA 56
CCGTAAGCCTGTGAAGGGACA 19
GAATAGCAGAAGTCCTTGAGT 253
GACGACGTATAGGGCCTGACG 33
GTCGAGCGCCCCGCAAGGGGA 59
GAGGCGCGCGATGGTAGGCTC 35083
GAATAACTCAGGGAAACTTGT 85
GGTTGGATGTTTGGTTAGGCG 27
GGGATGACTTGTGGCTAGGGC 18

ACGAATGGCGTAACGACTTCC 11
TACCCCCGGGGGTAGAGCACT 395
GAGGACCGGGATGGACGTATC 9471
GTAGATATTCGGAGGAACACT 26
ACTGAGATACGGCCCAGACTC 12
GTGCGGCAACGCATGCAGCTT 21
GTTCCGACCTGCACGAATGGC 17
AGTCGGCAGACACACGGCGGG 186
GTGCTGCATGGCTGTCGTCAG 778
AAGACGCGGGAGAGTAGGTCG 393
TAAGGCGAGGCCGAAAGGCGT 10
TGCGTACGGCGCGTGAGCGAG 16
TCGTGTCGTGAGATGTTGGGT 192
TGTTGGGTTAAGTCCCGCAAC 23
GCTGGACCGGAGACAGGTGCT 1376
GTGGGTAGTTTGACTGGGGCG 106
GGTGGGGAGTTTGACTGGGGC 148
CTCACCGTCTTACTGATCCTA 170
TACGGGAGGCAGCAGTGGGGA 32
GGGATAACCGCTGAAGGCATC 217
CGAACGCTGGCGGCAGGCTTG 52
TGCTGCATGGCTGTCGTCAGC 806
CACTCTAAGGGGACTGCCGGT 240
GGTACGTGAGTTGGGTTCAGA 89
CCAGCCCTTGACATCCCGGTC 93
CGTCTTACTGATCCTAACCAA 19
GACGATTTCCGAATGGGGAAA 19
CCCTAGTACGAGAGGACCGGG 27
CCAAGGCGCTTGAGAGAACTC 58
TGAGGGGCCGTAGCTCAGCTG 138
AGCGAACCTGGGGAACTAAAA 13
CTGGTTGGATGTTTGGTTAGG 33
CATGCAAGTCGAGCGCCCCGC 20
CGGAAGTGAGAATGCTGACAT 105
GTGGACTGGTAGAGAATACCA 2023
GTAAACGGCGGCCGTAACAAT 10
GGCCGAAAGGCGTAGTCGATG 56
CTATCAGAGCCGTGGAAGACC 25
ATGGGTCAGCGACTTAGTGTA 194
CTTGTAGCTCAGTTGGTTAGA 123
CTTGATGAGGGGCCGTAGCTC 152
TCCCTAAGTTATGGCTAAGTG 21
CCGTCTTACTGATCCTAACCA 26
CAATGAACTTTGGCGGACACG 16
GAGGACCGGGATGGACGTATT 11
AGGGCCATCGCTCAACGGATA 360
AGGGCGTTCAGTTCGATGCAT 316
GTAGATATTCGGAGGAACACC 6894
CCGGAGAAGAAGCCCCGGCTA 427
TACCAGCCCTTGACATCCCGG 57
GCAACGCATGCAGCTTACCGG 33
CTAAGGTAGCGAAATTCCTTG 116
AGGACCGGGATGGACGTATCA 12
ATTCGTGACGACATGTGTAGG 279

ACGGAGGCGCGCTATGGTAGG 24
GATTTACTGGGCGTAAAGCGC 2551
GAATTGGAAAGAGGCCGGATT 31
CCAGAGGAAAGGACATCAAAC 22
CCCTGGTAGTCCACGCTGTAA 26
CGTTCGTCGAGTGCAATGGCA 41
TAAACGATGAATGTTAGCCGT 185
GGCAACAACCCTGACCACCAT 24
GCTGGACCGGAGACAGGTGCC 12
ATGCGTACGGCGCGTGAGCGA 16
CCCTGGTAGTCCACGCCGTAA 240
TTCGTGGGCCTGCAGGTGGTG 432
CCGAATACCCCCGGGGGTAGA 223
AGTTTGATCCTGGCTCAGGAC 17
CGAACGCTGGCGGCAGGCTTA 2437
CATCCTGGGGCTGGAGCAGGA 20
GGTGCATGGCTGTCGTCAGCT 16
CGAAGCTGTGGATGCACGTAT 54
CGGTAATACGAAGGGGGCTAG 129
TGAAATCCTGTCTGAACATGG 20
GACGTATAGGGTCTGACGCCT 667
CATCGAACTGAACGCCCTGTT 26
GCGCAAGCCTGATCCAGCCAT 252
GGCACCTCGATGTCGACTCAT 10
GCTATGGGGACTCACCGTCTT 265
TAGCTCAGTTGGTTAGAGCAC 114
GACCATTACTGACGCTGCGGT 12
ATTTATGATGACTAGAAAGCT 13
TAAAGGCTCACCAAGGCGACG 18
CATCCTGGAGGTATCGGAAGT 43
AGGGCATTTGGTGGATGCCTT 29
GACGATTTCCGAATGGGGCAA 17
AGCCGTAGGGGAACCTGCGGC 30
CGGACCAGGCCAGTGGCTTTT 725
CAGCCCGGTAGCTCGTCAGGC 486
CGGTATCTGGGCTTGTAGCTC 66
ACCGGATGCCTACAAACAGTT 18
CGGTGCTGGAAGGTTAAGAGG 13
ATTTGTGAGTAGTTGGGGGTG 15
GCTGGCGGCAGGCTTAACACT 26
GCAATGAGAGTGATCAAGTGT 192
CTAAGGTCCCTAAGTTATGGC 526
TTGTAGCTCAGTTGGTTAGAG 125
GACTCCTACGGGAGGCAGCAG 125
GCGACGATCCATAGCTGGTCT 399
GCGACTGTTTAGCAAAAACAC 13
CTGCGAAGTCGCAAGACGACG 217
GGTACGGTAACACGTACTGGA 32
CACAGACCAGGGGGTAGCGAC 43
TAACTCAGGGAAACTTGTGCT 30
TTCGGATTTACTGGGCGTAAA 577
GCCTGCCCGGTGCTGGAAGGT 40
ACGGGTGAGTAACGCGTGGGA 19
AGAACGTCGTGAGACAGTTCG 268

CTCAGGACGAACGCTGGCGGC 23
GAGTTTGATCCTGGCTCAGGA 14
CGGCGGCGCCGGCAGCGGCAG 22
CGGGGGTAGAGCACTGGATGG 159
TGGGGAACTGAAACATCTAAC 18
TGGACCATTACTGACGCTGCG 12
GTCTGAGAGGATGATCAGCCA 57
AACACCAGTGGCGAAGGCGGC 921
GGCTCTGCGAAGTCGCAAGAC 753
GAGCACTGGATGGGCTATGGG 51
CTGGTGGTTATGGCGGAGCGG 3036
TGAGAATGCTGACATGAGTAA 144
ACCAGACTCCTACGGGAGGCA 14
CCTGCACGAATGGCGTAACGA 83
GAAACGCTCCAGCGCCAATGG 63
AGCGGCTGCACCCGATCCCAT 29
CTCTGCAACTCGAGTGCATGA 17
CGGGTAGTTTGACTGGGGCGG 14
CGGGGAGGTGCGAATACCCTT 11
CGACTGGGGTGAAGTCGTAAC 12
GCTGGCGGCAGGCTTAACACC 20
ATACACCGAAGCTGTGGATGC 168
ACGGCGGCCGTAACAATAACG 18
GGGCTGGGCTGCACACGTGCT 10
CACCTGAAAACGAGTATTCCC 10
TGAAGATGCGGGGTTCCTGCG 48
TACCGGGGCTCAAGCCATACA 1056
ACCATTACTGACGCTGAGGCG 30
TACGAAAGTCTGCCTGTTCTG 12
TTCCGTAAGCCTGTGAAGGGA 26
CGGTACTAATAGCTCGATCGA 5449
CTAAGGCGAGGCCGAAAGGCG 11
TGCGATAAGCGTCGGGGAGGT 37
GCTCACTGGACCATTACTGAT 23
CCCCGCTGTCTCCAACGCAGA 775
AACCTGGGGAACTGAAACATT 19
CCTTACGGGCTGGGCTACACA 477
ACCTTTTGTATAATGGGTCAG 24
GTCCCTAAGTTATGGCTAAGT 27
ACCCAGAGGAAAGGACATCAA 14
AGTTCCGACCTGCACGAATGG 17
TAAGGTCCCTAAGTTATGGCT 442
CGCAAGATTAAAACTCAAAGG 177
AGGGGCCGTAGCTCAGCTGGG 156
GGATCCCAAAACAACCAGGAT 65
AGACTCAGTGAAATTGAATTC 69
TTACCAAAAACACAGGGCTCT 16
CTGAACATGGGTCGACCACGA 1433
GTGATCTAGCCATGAGCAGGT 102
AGCTATATACGGACGGGATAA 70
CCTGATCCAGCCATGCCGCGT 64
CATTACTGACGCTGAGGTGCG 12668
GGTGGACTGGTAGAGAATACA 22
GCTAGCGTTGTTCGGATTTAC 161

CGGTACGTGAGTTGGGTTCAG 88
CCTGGGGAACTGAAACATCAA 10
GGGCTGGGCTATACACGTGCT 19
CGTCTCAAGACGCGGGAGAGT 157
TGGAAGGGCCATCGCTCAACG 47
GGGTCTGACGCCTGCCCGGTG 1409
TCGTGCATGACCGATAGCGAA 309
CCTAACCAAACTCCGAATACC 88
GGCCATCGCTCAACGGATAAA 93
CCTTGTCGGGTAAGTTCCGAC 66
TTTCCGAATGGGGCAACCCAC 12
ATAACAGGCTGATGACCCCCA 304
TTCCCCGTGAAGATGCGGGGT 23
TGATGACCCCCAAGAGTCCAT 97
CTAACGCATTAAACATTCCGC 137
AAAGCCGTCTCAGTTCGGATT 24
GTCATAGTGATCCGGTGGTCC 600
ACCGATAGCGAACCAGTACCG 156
GAGCGTCGACCGAATACCCCC 4468
GCCTGCAGGTGGTGACGGATC 235
CCCCGGGGGTAGAGCACTGGA 365
GAGTCCATATCGACGGGGTTG 83
GCTCACTGGACCATTACTGAC 5024
AACCTGGGGAACTGAAACATC 5529
GTCGTCGGCCCATGTGGGCCG 20
ATAACCGCTGAAGGCATCTAA 15
TGACCGATAGCGAACCAGTAC 134
CACACGCTTGATAAGCGTGGG 11
ATAGCTCAGCTGGGAGAGCAC 12
CATTACTGACGCTGAGGTGCA 28
TTTGTCCTGGGTGACAGCGTA 48
CGGGTGTGGAAGTGCGGCAAC 503
GGTGGACTGGTAGAGAATACG 10
GAGACAGGTGCTGCATGGCTG 217
AAAAGCCATCTCAGTTCGGAT 108
ACTGCCGGTGATAAGCCGAGA 598
TTAAAACTCAAAGGAATTGAC 79
GATCCCATTCCGAACTCGGCC 30
AGATGCGGGGTTCCTGCGGTT 51
GAGCTGGGCTACACACGTGCT 12
TACTAATAGCTCGATCGACTT 1024
AACATGGGTCGACCACGATCC 1432
ACGAACGCTGGCGGCAGGCTG 16
GTCGATGGGAACCACGTTAAT 38
ACTGGGACTGAGACACGGCCC 656
GGACCATTACTGACGCTGAGC 12
GGCAACCCACCTTAGATGACT 24
TCAGCGACTGGGGTGAAGTCG 407
AGAGCGTCGACCGAATACCCC 3618
CGTACTGGAGGACCGAACCCA 3130
GGGGTGCGACTGATTATAGCC 16
AGGCGCTGACACGGATTTGAC 46
GCGTCGACCGAATACCCCCGG 5482
CGTATGTGCGTGGTAGCGGAG 56

ATGTTTGGTTAGGCGGAAGAG 31
GACCGGAGACAGGTGCTGCAT 221
CTGCGGCTGGATCACCTCCTT 82
ACGGGCTGGGCTACACACGGG 16
AAGACCACCACGTTGATAGGC 39
CCTGGTGGTTATGGCGGAGCC 28
CAAGTCCTCCCAGGCCCACCA 24
CGAAGGCGCGCGATGGTAGGC 13
GAGGCGCGCGATGGTAGGCTG 14
CGCAAGCCTGATCCAGCCATG 244
AACTTGAGAGTTTGATTCTGG 11
GAGACGAAAGTCGGTCATAGT 108
GCGGCTCACTGGACCATTACT 10694
CCTGCTTTGCAAGCAGGGGGT 91
AAAGGTACGCCGGGGATAACA 114
GCGAACCAGTACCGTGAGGGA 277
CTCTTTCTTCATTGTTGATTG 16
AACCCACCTGAAAACGAGTAT 10
GGAAGAACACCAGTGGCGAAG 33
AATATTGACAGGATCTGTCCC 152
CGAGCGCAACCCTCGCCCTTA 14
GATGACTTGTGGCTAGGGGTA 15
ACACGTACTGGAGGACCGAAG 13
AACTTGAGAGTTTGATCCTGG 62
AGAGGATGATCAGTCACACTG 11
CGGAATAACTCAGGGAAACTT 153
AGGTTCAAGTCCTCCCAGGCC 36
GGGTAAGTTCCGACCTGCACG 17
CGCAGGTTCAAATCCTGCCCC 12
TCGGATTGCACTCTGCAACTC 408
GTAGAGCATACCAAGGCGCTT 10
AGTACGAGAGGACCGGGATGG 186
GGACTGAGACACGGCCCAGAC 144
TAGCTGGTTCTCCGCGAAATC 261
TAAACGGCGGCCGTAACAATA 12
GCGATAAGCGTCGGGGAGGTG 58
TATTGGACAATGGGCGCAAGC 650
TAGTGAACCAGTACCGTGAGG 33
AAGCGTGGGGTCGGAGGTTAA 17
ATCCTGGCTCAGAACGAACGC 1500
AGAATACCAAGGCGCTTGAGA 64
GGGGTGAAGTCGTAACAAGGT 38
CGTAAGCCTGTGAAGGGACAG 18
TGTAGCTCAGCTGGGAGAGCA 26
TGCGGCAACGCATGCAGCTTA 21
CGAATACCCCCGGGGGTAGAG 53
GTAGGGTCAGCGACTGGGGTG 196
GATGAAGGCCCTAGGGTTGTA 188
GATCGCGTGTGTTGTGAGGTC 590
CGGTAACCGGAGAAGAAGCCC 961
GGCATGCACAGGCGATGAAGG 328
ACACGTACTGGAGGACCGAAA 50
GATGACTTGTGGCTAGGGGTG 11800
GGCGGAGCGGCTGCACCCGAT 3564

ATGGATGTCTAACTGCGGCCC 166
ACCGACACTGGTGGACTGGTA 419
AGTCCACGCCGTAAACGATGA 24
TTACGAAAGTCTGCCTGTTCT 10
CTTGTGGCTAGGGGTGAAAGC 10
TGTTTACCAAAAACACAGGGC 19
GAGGCGCGCGATGGTAGGCTA 11
GAGTAACGATAAAGGGAGTGA 15
TCCGAATGGGGCAACCCACCT 938
CCGTAGCTCAGCTGGGAGAGC 7271
TGGACCGGAGACAGGTGCTGC 385
AAGGCGTAGTCGATGGGAACC 334
GCGGCTCACTGGACCATTACC 51
ACTCGAGTGCATGAAGTTGGA 165
CGGTAACACGTACTGGAGGAC 414
GCAGCGAGCACGCGAGTGTGA 29
AAACGCTCCAGCGCCAATGGT 49
AGGACCGGGATGGACGTATCT 9150
GTAGATATTCGGAGGAACACG 63
CTGAAACATCTAAGTACCCAG 54
GAGGACCGGGATGGACGTATA 16
GGGATGACTTGTGGCTAGGGA 32
CTGAGCAGGGGTAGCCGGCCC 10
CACACTGGGACTGAGACACGG 716
AAGGCAAAGAACAGGCGCAGC 29
ACCGTACCCTAAACCGACACT 962
CCAAGGCGCTTGAGAGAACTG 83
TTGTCGGGTAAGTTCCGACCT 65
TAACGGAGGCGCGCGATGGTA 3412
TCTAGAAGCCCGGCACCGCAG 24
TGTCCCTAGTACGAGAGGACC 58
TGCAATGGCATAAGCCTGCCT 34
CATCCTGGGGCTGGAGCAGGT 412
GTGAAATCCTGTCTGAACATG 69
TACCGGTACTAATAGCTCGAT 506
AAGTCGGTCATAGTGATCCGG 132
GCTGGACCGGAGACAGGTGCG 13
AACATCTAAGTACCCAGAGGA 53
TAGAGCAGCCCGGTAGCTCGT 10
TGTAGCTCAGTTGGTTAGAGC 120
GTCAGCTCGTGTCGTGAGATG 273
TAGGCCGGGTGTGGAAGTGCG 483
CGAGAATTGGAAAGAGGCCGG 71
GCAGTGGGGAATATTGGACAA 17
ATAAAAGGTACGCCGGGGATA 216
CACCTCGATGTCGACTCATCG 30
GCACCTCGATGTCGACTCATC 53
CGCATGAAGCTTACCGGTACT 72
TGACTGCGAGACTGACAAGTC 10
CTAAGTACCCAGAGGAAAGGA 490
CCCCGTGAAGATGCGGGGTTC 27
ATCGTTTACGGCGTGGACTAC 12
GCAAATGATCGGCCCGCGTTG 2131
CACCGAAGCTGTGGATGCACG 201

TTACTGATCCTAACCAAACTC 13
ATCGCAGGCCAGTCAGCCTGA 26
GCGACTTAGTGTATCGAGCAA 111
ACCGTACCCTAAACCGACACC 12
CAGTTTGACTGGGGCGGTCGC 25
GGGAGTTGGTTTTACCCGAAG 16
GTGGTGCATGGCTGTCGTCAG 15
AAGGGGACTGCCGGTGATAAG 560
ATAACCACCAGGTCGGCGAAG 58
GAAAGGACATCAAACGAGACT 19
GTAGATATTCGGAGGAACACA 41
ATGGTGGGTAGTTTGACTGGG 79
TTTGGGCTGCGCCTGTTCTTT 23
TGAAACAGTACCTGAAACCGG 42
GAGGACCGGGATGGACGTATG 22
GAAAACTCGACCGAAGTGGGT 16
GGGATGACTTGTGGCTAGGGG 15069
GTGGAAGGGCCATCGCTCAAC 45
AGAGACGAAAGTCGGTCATAG 84
GAGGCGCTGACACGGATTTGA 51
TTCGGAAGAACACCAGTGGCG 43
GAAGTCGGAATCGCTAGTAAT 274
TAGGCCGGGTGTGGAAGTGCA 13
CAGGGCTCTGCGAAGTCGCAA 1149
TGGCTAGGGGTGAAAGGCCAA 47
TCGGTTCGATCCCGTCCGGCT 769
TCTGCGAAGTCGCAAGACGAC 309
AACGGAGGCGCGCGATGGTAG 12608
CGAACGCTGGCGGCAGGCTTC 22
CGCGGGATGGAGCAGCCCGGT 14
TTAGACGGAAAGACCCCGTGC 305
ACGGATAAAAGGTACTCCGGG 39
ACCCCGACGAGGGGAGTGAAA 12
CCCGACGAGGGGAGTGAAACA 14
GTGAAGAGAAGATGTAATCGG 22
GCTGGACCGGAGACAGGTGCA 10
ACGCGCAGAACCTTACCAGCC 6965
TAGAACATAGATCGCAGGCCA 28
TCTAAGGGGACTGCCGGTGAT 139
TATGGAAGTAGGGCAATAAGG 36
TGACGGATCGCGTGTGTTGTG 47
TTCGTCTCAAGACGCGGGAGA 38
GGTTAAGAGGAGAGGTGCAAG 10
CTCAACGGATAAAAGGTACTC 88
CAAGACGACGTATAGGGTCTG 48
GCTGTAGCTCAGCTGGGAGAG 109
AGTAACGATAAAGGGAGTGAG 22
CGGCAACGCATGCAGCTTACC 32
GAAACTCAAAGGAATTGACGG 11
TGGGGAACTGAAACATCTAAG 190
CCTGACTTTGGTTTTCGGATC 91
CGGGGTGGATCTGTGGATCGC 13
GGGTGAAAGGCCAATCAAACT 10
GGACCGGAGACAGGTGCTGCA 303

GGATCGCGTGTGTTGTGAGGT 653
AGAAAGCGTAACAGCTCACTG 193
ACTGGGGTGAAGTCGTAACAA 35
TAACGCATTAAACATTCCGCC 149
GCGGGACACGTGAAATCCTGT 19
ACGGACCAGACTCCTACGGGA 28
ATCAAACGAGACTCCGCTAGT 77
GGGATGGAGCAGCCCGGTAGC 15
GAACATGGGTCGACCACGATC 1444
GGTGGTGACAGTGGGCAGCGA 111
GCAAACAGGATTAGATACCCT 18
ATGTTGGGTTAAGTCCCGCAA 22
GGAGTGAAACAGTACCTGAAA 11
ACTGGCATTCGTGACGACATG 484
GTGGCGAGCGAACGCGGACCA 70
TCTGAACAGGGCGTTCAGTTC 16
CGGGATAACCGCTGAAGGCAC 12
TAAGTCAGGGGTGAAATCCCG 28
AGGTAGCCGTAGGGGAACCTG 15
GGCTCACCAAGGCGACGATCC 227
GTCGGAGGTTCAAGTCCTCCC 44
TGGGGAACTGAAACATCTAAA 19
GTTTGTCCTGGGTGACAGCGT 32
CTCGGCCGGGAAACGCTCCAG 17
CCAATGGTACTTCGTCTCAAG 28
GTGCCAGCAGCCGCGGTAATA 15
CGGACTTTTAAGTCAGGGGTG 107
ACTGTTTACCAAAAACACAGG 20
TGAGCCTGACGAGCTACCGGG 48
ACACGTGAAATCCTGTCTGAA 42
GAAAAGCACCCCGACGAGGGG 31
CGGAGGCGCGCTATGGTAGGC 24
GATGATCCGCCACACTGGGAC 16
GAAGGGGGCTAGCGTTGTTCG 601
GTTGGTTTTACCCGAAGGCGC 93
GCAGGGTAGCTATATACGGAC 31
AAGCTCTTTCACCGGTGAAGA 207
GGGCTGGGCTACACACGCGCT 13
TGTGCCACCCCCCTATGGTTG 15
TGTCTCCAACGCAGACTCAGT 29
TGCCGCGTGAGTGATGAAGGC 26
GCGGGAGAGTAGGTCGCTGAC 10
AACGAGCGCAACCCTCGCCCT 68
AAGCACCCCGACGAGGGGAGT 32
TCGGCCGTGAAACGCTCCGGC 10
CTGGTAGAGAATACCAAGGCG 34
GTCGTGAGACAGTTCGGTCTC 15
TCCAGGACCGTGTATGGTGGG 10
GCATTCAGTTGGGCACTCTAA 26
CGGGATAACCGCTGAAGGCAT 240
GCCCAAGGTTTGTCCTGGGTG 38
TCCAGCGCCAATGGTACTTCG 40
GGAACTCGGCAAAATGCACGC 544
CATGGCCCTTACGGCTGGGC 119

GAACATAGATCGCAGGCCAGT 25
CCTAGGGTTGTAAAGCTCTTT 2159
CAGCAGTGGGGAATTTTGGAC 52
TACTTCGTCTCAAGACGCGGG 38
TCCATAGCTGGTCTGAGAGGA 54
ATGAAGGCCCTAGGGTTGTAA 805
TTTTGTTGGAGCAACGCTGGA 34
GCTGGCGGCAGGCTTAACACA 304
TGCGAAGTCGCAAGACGACGT 199
ATCGAGCAAGCTTAAGCCGGT 14
GGGCTGGGCTACACACGGGCT 14
ACAGTACCTGAAACCGGATGC 7003
CTGTTGTGGCGCCAGCCGCAT 2222
TGTATAATGGGTCAGCGACTT 63
TACCTGACTTTGGTTTTCGGA 19
AGGACCGAACCCATATCTGTT 29
TGACAAGTCGAGCAGAGACGA 17
CGGGATGGAGCAGCCCGGTAG 15
GAGTATTCCCTATCAGAGCCG 16
TGCTCACGGGCCGTACCGCAG 17
TCTCAGTTCGGATTGCACTCT 187
TGGCATAAGCCTGCCTGACTG 278
CATAGTGATCCGGTGGTCCCG 581
AAAACAACCAGGATGTTGGCT 71
TCGTCAGCTCGTGTCGTGAGA 241
ACCAACTTCGATCCGAAAACC 17
GTGGGCCTGCAGGTGGTGACG 358
ACCGAATACCCCCGGGGGTAG 271
GCGTAAAGCGCACGTAGGCGG 342
TACTCCGGGGATAACAGGCTG 77
GGCTCAAGCCATACACCGAAG 429
GAAGTTGGAATCGCTAGTAAT 123
GGGCGGCGGCGCCGGCAGCGG 39
ACTCCGCTAGTAGTGGCGAGC 65
TGGCGGCAGGCTTAACACATG 183
TGGCGGAGCGGCTGCACCCGA 4251
GGTCTGACGCCTGCCCGGTGC 1389
ACGCGTAGAACCTTACCAGCC 12
AGTTTGACTGGGGCGGTCGCC 286
GATGACTTGTGGCTAGGGGCG 33
GGAGTACTAGTCGGCAGACAC 16
ACGACGTATAGGGTCTGACGC 701
TACGGTCGCAAGATTAAAACT 188
GTGATACGCTGCGATAAGCGT 29
TTACTGATGAAAATGGATTGA 10
TTATGGCGGAGCGGCTGCACC 9521
CCCCGGCTAACTTCGTGCCAG 69
CCGGTAGGTGTAGGCGCAGCC 12
GGCAACCATAGGGGGGTGGCA 33
TTGGGGGTCATCAGCCTGTTA 15
CACGCTTGATAAGCGTGGGGT 16
CGCTCAACGGATAAAAGGTAC 201
ACGTCAAGTCCTCATGGCCCT 118
AACGCGCAGAACCTTACCAGC 73

AGATAGCTGGTTCTCCGCGAA 42
TTCAGAACGTCGTGAGACAGT 122
TCAAACGAGACTCCGCTAGTA 28
CTGGAGGTATCGGAAGTGAGA 23
TCGGAGATATTCGGAGGAACA 21
TTATTTGGTTGCGGGGGCAGG 12
CCTAAGGCGAGGCCGAAAGGC 15
GGGCTGTAGCTCAGCTGGGAG 274
CCCGCTGTCTCCAACGCAGAC 819
GACCATTACTAACGCTGAGGT 10
TGAGTGATGAAGGCCCTAGGG 14
CGGCTAACTTCGTGCCAGCAG 14
TTATGGCGGAGCGGCTGCACT 14
CGCTGAGGTGCGAAAGCGTGG 74
TGACGGGGGCCCGCACAAGCG 156
TACACACGTGCTACAATGGTC 12
ACAGGGCTCTGCGAAGTCGCA 1833
CTGCAACTCGAGTGCATGAAG 13
GCGCGGCCCATCAGGGCCGAC 33
GAAGCAACGCGCAGAACCTTA 24
ACCAGGCCAGTGGCTTTTGTG 177
CGCGTAGTAGCGTTTGCGTCG 10
GGCTCAAGCCATACACCGAAA 23
CGGGACACGTGAAATCCTGTC 20
ATCCGACGATTTCCGAATGGG 131
TGACGCTGAGGTGCGAAAGCG 63
ATGGTAGAGGTGAGTGGAATT 34
TGGCGGAGCGGCTGCACCCGG 12
AAAAACACAGGGCTCTGCGAA 70
TTGGAATCGCTAGTAATCGCG 1315
AGAGTTTGATCCTGGCTCAGG 11
GCTTTGCAAGCAGGGGGTCGT 89
GAGTGAAACAGTACCTGAAAC 22
CAACCATAGGGGGGTGGCACA 29
AACCTGGGGAACTGAAACACC 14
CGGCATTGTCTGCGGATGGTG 51
CCGAAAGTCCAAGGGTTCCTG 10
ACTTGATCACTCCCATTTACC 13
GTGTAGGCGCAGCGAAAGCGA 14
GTTAGGCGGAAGAGATTTTGG 11
ACATCCTGGAGGTATCGGAAG 45
AATGGTACTTCGTCTCAAGAC 102
AAGATGCGGGGTTCCTGCGGT 38
CACGGTAGGGTCAGCGACTGG 107
TGAGGTGCGAAAGCGTGGGGA 80
GCGAGGCCGAAAGGCGTAGTC 11
AAAGCCATCTCAGTTCGGATT 113
TGGTTGACAGGTTGGTTTGAC 10
ACTTCGGAAGAAGCGTGACCT 90
AGTTTGACTGGGGCGGTCTCC 45
TCGGGATGACTTGTGGCTAGG 16004
AGATCGGGATGACTTGTGGCT 435
AGTGCGGCAACGCATGCAGCT 11
GGGGAACTGAAACATCTAAGT 210

GGATAGGTGGTAGACTTTGAA 35
ACGGGATAACCGCTGAAGGCT 30
TCAAGTCCTCATGGCCCTTAC 108
TAAGCGTGGGGTCGGAGGTTA 41
CCTTTGATCCGACGATTTCCG 1001
TTGACTGATCTAGAAGCCCGG 37
CCCATTCCGAACTCGGCCGTG 112
GGGTGGAGCAGCCCGGTAGCT 210
AATTTTGGACAATGGGCGCAA 52
TTATGGCTAAGTGGGAAAGGA 100
CCCGGTCGCGGTTAGTGGAGA 251
GTTAGCCGGCCCCTAAGGCGA 56
GTTTGACTGGGGCGGTCTCCT 40
TTTTACATGGGGGTGCGACTG 12
TCGTCGGTTCGATCCCGTCCG 248
GCGAAATTCCTTGTCGGGTAC 24
ATTCGTGCAGGTCGGAACTTA 11
AAATTCCTTGTCGGGTAAGTA 15
ATATTGGACAATGGGCGAAAG 41
GGGGGTCATCAGCCTGTTATC 15
AACGGATAAAAGGTACGCCGG 68
TACTTGATGAGGGGCCGTAGC 105
CTGGGGCTGGAGCAGGTCCCA 14
TCGGGATGACTTGTGGCTAGA 12
TTGTCGTCAGCTCGTGTCGTG 17
AGAGTTTGATCCTGGCTCAGA 444
TTAATCTGAGCAGGGTTAGCC 74
ACTTCGTGCCAGCAGCCGCGG 23
CGGCATTGTCTGCGGATGGTA 45
AACTGAAACATCTAAGTACCC 32
ACGTATGTGCGTGGTAGCGGA 67
TGGGGAATTTTGGACAATGGG 51
CTGGTTCTCCGCGAAATCTAT 11
ATTCGTGGGCCTGCAGGTGGT 448
CCGCTAGTAGTGGCGAGCGAA 52
CTGGTGGACCTGTTGTGGCGC 649
GGTAGAGAATACCAAGGCGCT 24
ACAAGTCGAGCAGAGACGAAA 28
CCCGCACAAGCGGTGGAGCAT 12
GGAGGTTGGCTTAGAAGCAGC 75
ATAAAGGGAGTGAGAGACTCC 23
AGAACATAGATCGCAGGCCAG 27
GGCGGCATTGTCTGCGGATGC 16
CTTGAGTATGGTAGAGGTGAG 31
GGTCCCAAGGGTATGGCTGTA 43
GCGTCGGTATCTGGGCTTGTA 54
ACGGGATAACCGCTGAAGGCC 54
TGCGTCGGTATCTGGGCTTGT 50
TAAGCGTGGGGTCGGAGGTTG 20
GGTGTAGGCGCAGCGAAAGCG 27
CAGTTCGGATTGCACTCTGCC 12
AGCTATGTACGGACGGGATAA 11
ATTGGAAAGAGGCCGGATTTA 10
CTCGACCGAAGTGGGTGATAG 251

TTGAGAGTTTGATCCTGGCTC 1944
TTGATAAGCGTGGGGTCGGAG 32
ATGGCGGAGCGGCTGCACCCT 10
GAAGCTTACCGGTACTAATAG 90
CCTTTTGTATAATGGGTCAGC 25
CGGGGTTCCTGCGGTTAGACG 622
CACTCTGCAACTCGAGTGCAT 161
ATGACTTGTGGCTAGGGGGGA 39
AGGGCAACAACCCTGACCACC 182
TTGGGTTCAGAACGTCGTGAG 145
TTTGATCCTGGCTCAGAACGA 2715
CGTCGGCCCATGTGGGCCGCC 21
GTAACGATAAAGGGAGTGAGA 49
ACGATTTCCGAATGGGGAAAC 19
CCGACGAGGGGAGTGAAACAG 14
TCTAAGTACCCAGAGGAAAGG 416
CGGGGGAAAGATTTATCGGCA 20
CCGTATGTGCCCTTCGGGGGA 61
TTCTCATGTTTGTGTTCTTCG 21
GCGGACCTGGGGAACTGAAAC 12
ACGAGAGGACCGGGATGGACA 38
GTCGTGAGACAGTTCGGTCCC 620
ACTCACCGTCTTACTGATCCT 586
CTAGCGTTGTTCGGATTTACT 124
TTAAGAGGAGAGGTGCAAGCC 107
GCTCACGGGCCGTACCGCAGC 14
GAGCACCTGCTTTGCAAGCAG 34
GCAGACCTGGCAGCGACCTAC 14
ATGCAAGTCGAGCGCCCCGCA 85
CTAGTACGAGAGGACCGGGAT 103
TACCTGAAACCGGATGCATAC 11
ATGTTGGCTTAGAAGCAGCCA 120
CGTTAGTAGTGGCGAGCGAAC 13
GGTTTGTCCTGGGTGACAGCG 32
AGTGGGGAATATTGGACAATG 29
GCTGCATGGCTGTCGTCAGCT 1461
TGAAGTCGGAACAAGGTAGCC 20
AGGGGTGAAAGGCCAATCAAA 12
CGATTTCCGAATGGGGAAACC 19
GACGTATCTCTGGTGGACCTG 61
GCGTAGTCGATGGGAACCACG 64
AGAGTAACGGAGGCGCGCGAT 20
GGATAAAAGGTACGCTGGGGA 13
ATCTGCCGTGGGTGTAGGAAT 16
CACTATCCTTCAGTTAGGCTG 21
CTCAACGGATAAAAGGTACCC 31
CAAATCCTGCCCCCGCAACCA 32
TGGGTCGACCACGATCCAAGC 973
ACTCGGCCGTGAAACGCTCGA 14
ATGGCTGTTCGCCATTTAAAG 11
TCTAAGCGGGAAACCCACCTG 310
CAGCAGTGGGGAATATTGGAC 15
ACGAGAGGACCGGGATGGACG 5119
GCGGCATTGTCTGCGGATGGA 18

TTTGCGTCTCCGTGTTTTACA 27
CTCGAGTGCATGAAGTTGGAA 56
GTACTTCGTCTCAAGACGCGG 74
TCCGTAAGCCTGTGAAGGGAC 35
GGCTGGGCTACACACGTGCTC 10
ATCGTTCGTCGAGTGCAATGG 33
AGCAGGTCCCAAGGGTTTGGC 11
GGTAGGGTCAGCGACTGGGGT 112
GTGATCAAGTGTCTTAAGGGC 99
GGTGTAGGAATATTGACAGGA 52
GCGAACCTGGAGAACTGAAAC 11
GTGGGGAGTTTGACTGGGGCG 230
TCGCAAGATTAAAACTCAAAG 185
CGGTAGGGTCAGCGACTGGGG 122
GGTGATAGTCCCGTACACGTA 29
TGGGGCTGGAGCAGGTCCCAA 14
TGGGTGTAGGAATATTGACAG 44
TGAACTTTGGCGGACACGTTT 19
TCCGGTGGTCCCGCGTGGAAG 45
ACCATTACTGACGCTGAGGTC 16
CGGGTGAGTAACGCGTGGGAA 18
CCCTATCTGCCGTGGGTGTAG 175
TCCGCCTGGGGAGTACGGTCG 3545
TTCCTGCGGTTAGACGGAAAG 17
CTCACTGGTCTAAATAAGGGT 23
AACCACCAGGTCGGCGAAGAA 54
TGACTGGGGCGGTCTCCTCCT 22
CTGGGGAACTGAAACATCTAA 580
TAGGGTTGTAAAGCTCTTTCA 1618
ACGACATGTGTAGGATAGGTC 14
GTAGTTTGACTGGGGTGGTCG 15
AGTAATCGCGGATCAGCATGC 158
CTGACTTTGGTTTTCGGATCG 81
CGCAACGAGCGCAACCCTCGC 114
AACATAGATCGCAGGCCAGTC 34
CGCGGGGTGGAGCAGCCCGGT 286
TCACGGGCCGTACCGCAGCTG 95
GACCTGGTGGTTATGGCGGAC 53
GATTTGTGAGTAGTTGGGGGT 15
CGTGAAACGCTCCAGCGCCAA 3392
GAAGACCACCACGTTGATAGG 22
GCAACGCATGAAGCTTACCGG 13
GTCGACTCATCGCATCCTGGG 256
CTCCGCTAGTAGTGGCGAGCG 66
CTCCTACGGGAGGCAGCAGTG 71
AGCTGTGGATGCACGTATGTG 31
TATTCCCTATCAGAGCCGTGG 18
GGCGTAAAGCGCACGTAGGCG 267
GAGTAACGGAGGCGCGCGATG 65
CGGAGGCGCTCGATGGTAGGC 10
TTCGGAGGAACACCAGTGGCG 3954
ATGACTTGTGGCTAGGGGTGA 2701
TGGCCGTGAAACGCTCCAGCG 16
TCAGAACGTCGTGAGACAGTT 315

AGCCTGATCCAGCCATGCCGC 132
CGCGGGGTGGAGCAGCTCGGT 10
GACCTGCACGAATGGCGTAAC 103
TGCAACTCGAGTGCATGAAGT 13
TTTCTAGTCATCATAAATAAG 426
GACTCACCGTCTTACTGATCC 470
TAGCTCAGCTGGGAGAGCACC 5328
ACGGAGGCGCGCGATGGGAGG 11
AAGTACCCAGAGGAAAGGACA 573
TGATCGGCCCGCGTTGGATTA 207
AGTAATCGCGGATCAGCATGT 20
AGTCTGAACAGGGCGTTCAGT 26
GCGTAGAACCTTACCAGCCCT 12
GTACGGTCGCAAGACTAAAAC 10
TGTGCCCTTCGGGGGAAAGAT 10
CTCTGCGAAGTCGCAAGACGA 304
ACGGATAAAAGGTACGCTGGG 15
AAGATTAAAACTCAAAGGAAT 81
GATGATCAGCCACACTGGGAC 6092
TGGAGGTATCGGAAGTGAGAA 16
TGGCTGTTCGCCATTTAAAGC 21
GGGCAACCATAGGGGGGTGGC 37
TGGGTTCAGAACGTCGTGAGA 172
AATTCGTAGATATTCGGAAGA 32
ACCTTAGATGACTAGAAAATC 169
TAATATTCGTGGGCCTGCAGG 41
TAGGATGTTGGCTTAGAAGCA 23
CGATGTCGACTCATCGCATCC 45
GGGTCGTCGGTTCGATCCCGT 334
GGGGAATTTTGGACAATGGGC 53
CGAGTGCATGAAGTTGGAATC 236
TGACTTTGGTTTTCGGATCGA 81
TCTATTTAGGTAGAGCGTCGA 25
GGTGGACCTGTTGTGGCGCCA 236
ATGACTTGTGGCTAGGGGTGG 107
CTCGTGCATGACCGATAGCGA 315
AGACGAAAGTCGGCCATAGTG 10
TTACTTGATGAGGGGCCGTAG 102
TTAGAAGCAGCCATCATTTAA 10
TGCATGAAGTCGGAATCGCTA 10
AAGTCGAGCGCCCCGCAAGGG 76
CTGAGCAGGGTTAGCCGGCCA 31
GAGAAGCTGGTCTTTCTGCTG 304
GGCCCCTAAGGCGAGGCCGAA 13
GAGTGCAATGGCATAAGCCTG 24
TCAGTTCGGATTGCACTCTGC 211
AACGCTCCAGCGCCAATGGTA 54
CGGGGTGGAGCAGCTCGGTAG 10
AAACATTCCGCCTGGGGAGTA 152
CCTTAGGGTTGTAAAGCTCTT 15
ATCGGATCAACTGAAGAGTTG 14
GATACGCTGCGATAAGCGTCG 32
TGGGCAACCATAGGGGGGTGG 30
TATCTCTGGTGGACCTGTTGT 58

CGAGCAGAGACGAAAGTCGGT 129
GTGGTAGACTTTGAAGCAGGG 255
CGGCCCCTAAGGCGAGGCCGA 16
CCGGTAGGTGTAGGCGCAGCG 149
GCGCAGCGAAAGCGAGTCTGA 23
ACCCTGGTAGTCCACGCTGTA 32
ATGGGAACCACGTTAATATTC 23
TCAGCCACACTGGGACTGAGA 297
GAGGCGCGCGATGGTAGGCCC 31
ACGATAAAGGGAGTGAGAGAC 795
GCAGCCCGGTAGCTCGTCAGG 464
TCGGGGGAAAGATTTATCGGC 34
CCTAGTACGAGAGGACCGGGA 28
GGGGAGTACGGTCGCAAGATT 69
GGCGGCAGGCTTAACACATGC 165
TGATACTGGAAGTCTTGAGTA 18
CCATTACTGACGCTGAGGCGC 25
TAGTAATCGCGGATCAGCATG 155
CGGTTCGATCCCGTCCGGCTC 684
GTGAGTGATGAAGGCCCTAGG 17
TTCGCCATTTAAAGCGGTACG 13
TTGACTGGGGCGGTCGCCTCC 141
GTACTCCGGGGATAACAGGCT 85
GTACGGTAACACGTACTGGAG 37
GTTAGGCTGGACCGGAGACAG 184
AAGTCCTCATGGCCCTTACGG 112
AAGTGGGTGATAGTCCCGTAC 71
GTCCCGCAACGAGCGCAACCC 74
GGACTGCCGGTGATAAGCCGA 2047
GCTTGAGAGAACTGCGTTGAA 1814
TCAGTGAAATTGAATTCCCCG 38
AAGAAGCGTGACCTCACTATG 89
CCGGCCCCTAAGGCGAGGCCG 16
TAATGGGTCAGCGACTTAGTG 73
AATGGTGGTGACAGTGGGCAG 94
AACTCAGGGAAACTTGTGCTA 22
TGATGAAGGCCCTAGGGTTGT 108
GCCGGGAAACGCTCCAGCGCC 18
AATATTGGACAATGGGCGAAA 30
CGCCTGCCCGGTGCTGGAAGG 168
TGAGCAGGTTGAAGGTACGGT 503
ACGGTAGGGTCAGCGACTGGG 129
AACCCTGACCACCATCTAAGG 29
TACAATGGTGGTGACAGTGGG 16
TTGATCCGACGATTTCCGAAT 308
CTGTTGTGGCGCCAGCCGCAA 10
ACAGTCGTGAGACATCCTGGA 37
TCGTCTCAAGACGCGGGAGAG 157
AGACCCCGTGCACCTTTACTA 10
GGGCCATCGCTCAACGGATAA 95
GGTGTGACGCCTGCCCGGTGC 11
GGGTGAAGTCGGAACAAGGTA 15
GGAGGCGCGCGATGGTAGGAT 47
GAGAAGAAGCCCCGGCTAACT 29

TCGAGAATTGGAAAGAGGCCG 107
CTTTGGCGGACACGTTTCTTG 175
TTCGGTCCCTATCTGCCGTGG 1406
TCGACTTGATCACTCCCATTT 387
GGGTCGGAGGTTCAAGTCCTC 283
TTTTGGACAATGGGCGCAAGC 64
GATCGAGAATTGGAAAGAGGC 453
TAATTCGAAGCAACGCGCAGA 28
TCAGGGGTGAAATCCCGGGGC 73
GACCATTACTGACGCTGGGGT 16
GACACTGGTGGACTGGTAGAG 376
CAGTGGCGAAGGCGGCTCACT 373
CTCCGAATACCGGGGAGTACT 20
ACGGTAACACGTACTGGAGGA 288
TAGTAGTGGCGAGCGAACGCG 34
TAGTGATCCGGTGGTCCCGCG 109
GGGGGCTAGCGTTGTTCGGAT 877
CTTGTACACACCGCCCGTCAC 23
CACGGCCCAAACTCCTACGGG 12
CCATTACTGACGCTGAGGAGC 10
TAACCACCAGGTCGGCGAAGA 52
TCGTGAGACATCCTGGAGGTA 117
CTAAGTGGGAAAGGATGTGAG 140
AACGGTCGGAAATCGTTCGTC 118
TTTGATCCGACGATTTCCGAA 523
ACTTGATCACTCCCATTTACA 34
AGCACTGGATGGGCTATGGGG 77
CGGCATTGTCTGCGGATGGTT 156
TTGGCACCTCGATGTCGGCTC 11
CTCAGAACGAACGCTGGCGGC 2696
CGGCCGGGAAACGCTCCAGCG 19
TTCAAATCCTGCCCCCGCAAC 29
ATGGAGCAGCCCGGTAGCTCG 15
ATGACCGATAGCGAACCAGTA 129
AAATTCCTTGTCGGGTAAGTT 315
CTTAACACATGCAAGTCGAGC 30
GCGAAATTCCTTGTCGGGTAG 17
TGAAGGCCGCAGGTTCAAATC 42
TTGGTGGGCCTGGGAGGACTT 12
GGCCTAACACATGCAAGTCGA 10
TAAGCGTGGGGTCGGAGGTTC 2466
GGAACCACGTTAATATTCGTG 23
ACGGGATAACCGCTGAAGGCG 16
GGTCCCAAGGGTATGGCTGTT 2447
TTATGAGCCTGACGAGCTACC 896
GGCGGCATTGTCTGCGGATGG 768
ACGTATAGGGTCTGACGCCTG 944
GGGAACCTGCGGCTGGATCAC 34
AATAGCAGAAGTCCTTGAGTA 91
GAGGTTCAAGTCCTCCCAGGC 41
GGCAACGCATGCAGCTTACCG 29
CCCGGTCGCGGTTAGTGGAGC 10
GCGAACCTGGGAAACTGAAAC 12
CAGAGACGAAAGTCGGTCATA 90

TAAGCGTCGGGGAGGTGCGAA 392
CATAGCTCAGCTGGGAGAGCA 12
ACCGGTACTAATAGCTCGATC 5375
TACGTGAGTTGGGTTCAGAAC 97
ACGTCGTGAGACAGTTTGGTC 26
GTAATCGCGGATCAGCATGCC 156
GCGAAGTCGCAAGACGACGTA 60
GTGCTGGGCTACACACGTGCT 10
GAGCAAGCTTAAGCCGGTAGG 114
GTTCCCGGGTCTTGTACACAC 19
CCCAGAGGAAAGGACATCAAA 18
TGGTGGTGACAGTGGGCAGCG 71
CGTACACGTAGAATAGCAGAA 175
ATCGTCGGTTCTTTGAAAACT 12
GGATGATCCGCCACACTGGGA 17
TTAGAAGCGAACCTGGGGACC 10
GCGAAATTCCTTGTCGGGTAA 4574
ACGTCGTGAGACAGTTCGGTC 799
CCTGCATATAGACCGTACCCT 777
CGGCATTGTCTGCGGATGGTC 42
GTAGGGCGGGACACGTGAAAT 15
GTGGTGACAGTGGGCAGCGAG 180
GGCGTAGTCGATGGGAACCAC 67
GACTTCCCCGCTGTCTCCAAC 191
TGCACGTATGTGCGTGGTAGC 35
ACAACCCTGACCACCATCTAA 45
AGGGCTCTGCGAAGTCGCAAG 1119
ACACTGGTGGACTGGTAGAGA 2095
GGCGGCATTGTCTGCGGATGA 18
ATTAAACATTCCGCCTGGGGA 707
AGCCACATTGGGACTGAGACA 11
ATGGCGTAACGACTTCCCCGC 17
AATATTGGACAATGGGCGCAA 379
GAGTCTGAACAGGGCGTTCAG 41
CTTGGCATGCACAGGCGATGA 232
CCGTCGTGGAGAGGGCAACAA 22
GACCGAACCCATATCTGTTGC 17
CAGTTCGGATTGCACTCTGCA 68
GATGACGTCAAGTCCTCATGG 10
CGCAACCCTCGCCCTTAGTTG 11
CCTTTGATCCGACGATTTCCC 11
TAAGCGTGGGGTCGGAGGTTT 11
CATTCGTGACGACATGTGTAG 33
TGGTAGAGAATACCAAGGCGC 33
ACGGGATAACCGCTGAAGGCA 888
GGGGAACTGAAACATCTAAGA 11
CAGGTGCTGCATGGCTGTCGT 753
ACGTGAGTTGGGTTCAGAACG 22
TTACCAGCCCTTGACATCCCG 65
CAGGGGTGAAATCCCGGGGCT 45
CGACCGAAGTGGGTGATAGTC 255
ATGGCGGAGCGGCTGCACCCG 6875
TCGCTAGTAATCGCGGATCAG 83
CAATGAGAGGGATCAAGTGTC 21

CGGATTTACTGGGCGTAAAGC 2580
ACGGGCTGGGCTATACACGTG 16
TAGAGGTGAGTGGAATTCCGA 98
GCCGCGGTAATACGAAGGGGG 289
CAAGGCGCTTGAGAGAACTGC 62
GATGGTAGGCTCAGAACGGTC 193
CATCGCATCCTGGGGCTGGAG 35
GACACTATCCTTCAGTTAGGC 16
GGAACTGAAACATCTAAGTAC 155
TGAAGGGACAGTCGTGAGACA 25
TTTTACCCGAAGGCGCTGTGC 387
GGAGGACCGAACCCATATCTG 31
GAGTTTGATCCTGGCTCAGAA 357
GTATGTGCCCTTCGGGGGAAA 45
CTTGCAGCGAAGCGGTTCCAG 112
GAGGCGCGCGATGGTAGCCTC 12
TAATCGCGGATCAGCATGTCG 19
ACCGGAGACAGGTGCTGCATG 371
CTGGAGAGTTTGATCCTGGCT 12
ACCATTACTGACGCTGAGGTG 16058
CCGTGAAACGCTCCAGCGGCA 11
CACGTATGTGCGTGGTAGCGG 55
TCTCTGGTGGACCTGTTGTGG 401
AATGGGTCAGCGACTTAGTGT 88
GAAGCGAACCTGGGGAACTGG 38
GCTGAGGTGCGAAAGCGTGGG 87
ACCATTACTGACGCTGGGGTG 16
GCCTGACGAGCTACCGGGCTG 17
TGGGCTACACACGTGCTACAA 321
TTGCAGACCTGGCAGCGACCT 21
GGGCTCAACCCCGGAACTGCC 36
GCGGCATTGTCTGCGGATGGT 509
AGCCATACACCGAAGCTGTGG 27
GGGGATAACAGGCTGATGACC 10
TGGTAGGCTCAGAACGGTCGG 29
TTGTATCTCGAGAAGCTGGTC 30
GCAACGAGCGCAACCCTCGCC 117
ACTTCCCCGCTGTCTCCAACG 273
GGGTGTAGGAATATTGACAGG 42
TCCCAAAACAACCAGGATGTT 87
AAACCAAGTGATCTAGCCATG 11
CATGAAGTTGGAATCGCTAGT 80
AGTGATCCGGTGGTCCCGCGT 68
TGAACCAGTACCGTGAGGGAA 29
TTGAAGGAACTCGGCAAAATG 163
TGAATACGTTCCCGGGCCTTG 181
CGTGATACGCTGCGATAAGCG 29
AGTTCGGATTGCACTCTGCAA 51
ATGGCGGAGCGGCTGCACCCA 12
GCAGCGAAAGCGAGTCTGAAC 30
GAGAGGATGATCCGCCACACT 11
AGAGCATACCAAGGCGCTTGA 10
GCGTAGTAGCGTTTGCGTCGG 10
CCAAAACAACCAGGATGTTGG 87

GCGCTGACACGGATTTGACCT 12
GAGGTATCGGAAGTGAGAATG 20
GGCTGGGCTACACACGTGCTA 13752
GAAGCGAACCTGGGGAACTGA 9180
GACTTGATCACTCCCATTTAC 235
GTGAAGGGACAGTCGTGAGAC 33
ATTTGGTGGATGCCTTGGCAT 37
GGTAGCCGTAGGGGAACCTGC 13
AGGGTTAGCCGGCCCCTAAGG 340
GGTGGAGCAGCCCGGTAGCTC 191
AGCAGAAGTCCTTGAGTAGGG 188
GCGGCATTGTCTGCGGATGGC 49
AGTTGGAGCCCAAGGTTTGTC 80
ACGAGAGGACCGGGATGGACT 15
ACCATTACTGACGCTGAGGTA 48
TAGCTCGTCAGGCTCATAACC 336
TTGGGCTGCGCCTGTTCTTTG 32
GACGTCAAGTCCTCATGGCCC 41
GGAGTTTGGTTAGGATCAGTA 17
TGTAGGAATATTGACAGGATC 119
CGGCTCACTGGACCATTACTG 8214
CTACACACGTGCTACAATGGT 26
TCGAGCAAGCTTAAGCCGGTA 101
TGCCTTTGATACTGGAAGTCT 10
CAAGGGTATGGCTGTTCGCCA 117
CGTATAGGGTCTGACGCCTGC 916
CTGGGGAACTGAAACATCTAC 13
GAAATTCCTTGTCGGGTAAGT 2169
CTGCATGGCTGTCGTCAGCTC 1435
ATAGGCCGGGTGTGGAAGTGC 297
TTTGTGAGTAGTTGGGGGTGG 20
CGTTCAGTTCGATGCATTAGA 322
AAAAGGTACTCCGGGGATAAC 72
ACGCTCCAGCGCCAATGGTAC 64
CGGGTAAGTTCCGACCTGCAC 17
GAAGCTGTGGATGCACGTATG 40
AGCTCAGTTGGTTAGAGCACA 83
GGAAGGGCCATCGCTCAACGG 84
TGCATGGCTGTCGTCAGCTCG 1085
AATATGGAAGTAGGGCAATAA 11
ACGACATGTGTAGGATAGGTA 12
GCGGTTAGTGGAGACACTATC 41
CCCTTTGATCCGACGATTTCC 1200
TTCGTGCAGGTCGGAACTTAC 11
CGTGCCAGCAGCCGCGGTAAT 16
ACGGGGGCCCGCACAAGCGGT 295
CCTGACGAGCTACCGGGCTGC 20
ATGCGGGGTTCCTGCGGTTAG 129
ACACGCACTGGAGGACCGAAC 30
GACCTGGTGGTTATGGCGGAA 25
CTGAGCAGGGTTAGCCGGCCT 10
TAGCTCAGCTGGGAGAGCACA 12
TAGTCCACGCCGTAAACGATG 21
GAAACCGGATGCCTACAAACA 50

GTTTGATCCTGGCTCAGGACG 13
GACATGTGTAGGATAGGTGGT 984
CGGCCCAAACTCCTACGGGAG 14
CCATCTCAGTTCGGATTGCAC 111
ATAGCTCGATCGACTTGATCA 43
CTGGTCTGAGAGGATGATCAG 10
TCCGACGATTTCCGAATGGGG 182
CCGCAAGGAGGCAGGCGACCA 21
GTTTGGTTAGGCGGAAGAGAT 11
CGAGTGCAATGGCATAAGCCT 49
TCCTGCATATAGACCGTACCC 710
GGCTGGGCTACACACGTGATA 24
CCGACACTGGTGGACTGGTAG 413
AAGTACCCAGAGGAAAGGACC 13
ACGACATGTGTAGGATAGGTG 1113
AATAACGGTCCTAAGGTAGCG 10
GATTTCCGAATGGGGCAACCC 14
GAGGACCGGGATGGACGTACC 10
CCGGTAGCTCGTCAGGCTCAT 17
GCAGAACCTTACCAGCCCTTG 1422
TAACACGTACTGGAGGACCGA 213
CTGAGCAGGGTTAGCCGGCCC 10966
TAGCTCAGCTGGGAGAGCACG 11
GGAAGTAGGGCAATAAGGCAA 16
TCTTGGGGGTCATCAGCCTGT 20
CGACGTATAGGGCCTGACGCC 27
GTCGAGCAGAGACGAAAGTCG 70
GGGGGTGCGACTGATTATAGC 35
TGATAGTCCCGTACACGTAGA 38
AAGGAACTCGGCAAATTGCAC 21
CCTGTTGTGGCGCCAGCCGCA 3618
GAGCGGCTGCACCCGATCCCA 82
GGCCGGGTGTGGAAGTGCGGC 541
GTTGGGTTCAGAACGTCGTGA 45
ACGGGCCGTACCGCAGCTGAC 132
GTACACGTAGAATAGCAGAAG 78
GAAGGAACTCGGCAAATTGCA 20
GACCTGGTGGTTATGGCGGAG 3771
CTGATCTAGAAGCCCGGCACC 53
GACGAGGGGAGTGAAACAGTA 17
AGAGTAGGTCGCTGCCAGGTC 5710
AAATCCTGTCTGAACATGGGG 12
TCGGCCGTGAAACGCTCCAAC 11
ATACCGGGGAGTACTAGTCGG 481
GAAAGCGTAACAGCTCACTGG 206
TAGAGCGTCGACCGAATACCC 2024
CGCGGTTAGTGGAGACACTAT 44
TCTTGAGTATGGTAGAGGTGA 35
GAGGTGAGTGGAATTCCGAGT 111
TGAAGAGAAGATGTAATCGGA 23
ACCGCAGACGAGGCGCTGACA 12
GGTCGCAAGACTAAAACTCAA 12
CTTACCAGCCCTTGACATCCC 66
AGCCACACTGGGACTGAGACA 332

GACGGATCGCGTGTGTTGTGA 119
GAAGAAGCCCCGGCTAACTTC 23
CCGCCACACTGGGACTGAGAC 18
CGGAGGTTCAAGTCCTCCCAG 43
CGTTGATAGGCCGGGTGTGGA 48
CCCCTAAGGCGAGGCCGAAAG 15
CGGGCTGGGCTGCACACGTGC 11
CGGAGGCGCGCGATGGTAGGC 37976
TGAGGGAAAGGTGAAAAGAAC 13
TAGGATAGGTGGTAGACTTTG 45
AGCAACGCGCAGAACCTTACC 34
AGCGAACAGGATTAGATACCC 13
GGTGGTCCCGCGTGGAAGGGC 40
TTAAACATTCCGCCTGGGGAG 703
ATGGAAGTAGGGCAATAAGGC 35
GCCGTAAAACGCTCCAGCGCC 12
GGATAACCGCTGAAGGCATCT 93
AGGCGCGCGATGGTAGGGTCA 10
AGTCGGTCATAGTGATCCGGT 131
CTGGGCTACACACGTGCTACA 9786
GGATAAAAGGTACCCCGGGGA 75
TGATCCGACGATTTCCGAATG 254
AGTAGGTCGCTGCCAGGTCTG 876
TAGCTCCTGCATATAGACCGT 268
AATCGTTCGTCGAGTGCAATG 24
AAGGTTTGTCCTGGGTGACAG 32
CCACCACGTTGATAGGCCGGG 158
TGCCTTGGCATGCACAGGCGA 63
GGATGCCTACAAACAGTTGGA 45
CAGAACGTCGTGAGACAGTTC 263
CCATAGCTCAGCTGGGAGAGC 147
GATCGACTTGATCACTCCCAT 328
TAGTCGATGGGAACCACGTTA 21
CAAGGTAGCCGTAGGGGAACC 21
TGCCTACAAACAGTTGGAGCC 22
GTGATAGTCCCGTACACGTAG 28
GACCGTACCCTAAACCGACAC 839
CGAAAGGCGTAGTCGATGGGA 246
CGAGGGGAGTGAAACAGTACC 19
ACCTGGTGGTTATGGCGGACC 31
TGGGGAATATTGGACAATGGG 96
CGACCTGGTGGTTATGGCGGA 3949
GGATTTACTGGGCGTAAAGCG 2558
TGGGTTAAGTCCCGCAACGAG 18
GGCCGTAGCTCAGCTGGGAGC 19
AGTGGGTGATAGTCCCGTACA 26
ACGGGGTTGTTTGGCACCTCG 218
ATTTAGGTAGAGCGTCGACCG 86
CCCGGGTCTTGTACACACCGC 22
TGAGAGAACTGCGTTGAAGGA 5128
CTGGGCTACACACGTGCTACG 91
CTAACGTCCGTCGTGGAGAGG 10
ATCTCTGGTGGACCTGTTGTG 117
TCCGACCTGCACGAATGGCGT 159

TGGTGGACCTGTTGTGGCGCC 344
CTCGGCCGTGAAACGTTCCAG 14
TGAAGCTTACCGGTACTAATA 90
TTTCACCGGTGAAGATAATGA 24
GTTGGTTAGAGCACACGCTTG 12
GGTGAAGTCGAAACAAGGTAG 18
AGCTTTACACTGGCATTCGTG 111
ATGACTTGTGGCTAGGGGCGA 13
TAGATCGGGATGACTTGTGGC 457
GGGCTGGGCTACACATGTGCT 13
AAATGATCGGCCCGCGTTGGA 1935
TGACCCCCAAGAGTCCATATC 12
CTCACTGGACCATTACTGACA 63
AGTTCGATGCATTAGACCCGA 82
AAACTCAAAGGAATTGACGGG 172
TAGCTCAGCTGGGAGAGCGCC 26
AACTCGGCAAAATGCACGCGT 414
GAGGATGATCAGCCACACTGG 1207
GTGTAGGAATATTGACAGGAT 111
AACACAGGGCTCTGCGAAGTC 242
CCACATTGGGACTGAGACACG 33
AGCCATCTCAGTTCGGATTGC 130
GCACCTGCTTTGCAAGCAGGG 86
GTTTAGAACGTCGTGAGACAG 11
TGAGACAGTTCGGTCCCTATC 179
CAGAACGGTCGGAAATCGTTC 93
GCATGCAGCTTACCGGTACTA 125
CACGGACCAGACTCCTACGGG 28
CGGATCGCGTGTGTTGTGAGG 657
CTGGCGGCAGGCTTAACACAT 190
CGTCAAGTCCTCATGGCCCTT 59
CGCACGTAGGCGGACTTTTAA 52
AGTAATCGCGGATCAGCACGC 11
GACTTTGGTTTTCGGATCGAA 41
GGACCAGACTCCTACGGGAGG 20
GAGAGGAAGGTGGGGATGACG 30
GCAAGGGGAGCGGCAGACGGG 25
CGCATTAAACATTCCGCCTGG 578
GGTGGGGTAAAGGCTCACCAA 26
ACCTGGGGAACTGAAACATCT 4074
AGCCGGCCCCTAAGGCGAGGC 20
GCGATGGTAGGCTCAGAACGG 318
AAGGAAGATCGAGAATTGGAA 46
GTCGGCAGACACACGGCGGGT 240
CCGTGAAACGCTCCAGCGCCC 57
CTATATACGGACGGGATAACC 159
ACGAGCATTTGCAGTCGAATG 35
GTAAAGGCTCACCAAGGCGAC 36
TAGAAGCGAACCTGGGGAACT 9792
TGCAGACCTGGCAGCGACCTA 15
GCTGGTCTGAGAGGATGATCA 24
TTAGTGTATCGAGCAAGCTTA 17
ACGTATCTCTGGTGGACCTGT 101
AGTTGGGCACTCTAAGGTGAC 26

TCGGCCGGGAAACGCTCCAGC 19
CTACGGAATAACTCAGGGAAA 173
CGTTCCCGGGCCTTGTACACA 79
ATAGGGTCTGACGCCTGCCCG 970
GTGTGGAAGTGCGGCAACGCA 194
ATAAAAGGTACTCCGGGGATA 60
GCGCAACCCTCGCCCTTAGTT 13
TAGTTTGACTGGGGCGGTCTC 48
CGCTCCAGCGCCAATGGTACT 14
GGAGGCGCTCGATGGTAGGCT 10
GCTTACCGGTACTAATAGCTC 218
GTAGAGCACTGGATGGGCTAT 19
ACCGGGGCTCAAGCCATACAC 2690
CTCACTGGACCATTACTGACG 4285
GGGAATATTGGACAATGGGCG 529
GCCCGGTAGCTCGTCAGGCTC 316
GAAACAGTACCTGAAACCGGA 55
CACCTTAGATGACTAGAAAAT 69
CCGTGAAACGCTCCAGCGCCT 16
TTACTGGGCGTAAAGCGCACC 12
AATTCGTAGATATTCGGAGGA 245
TAGAAGCGAACCTGGGGAACC 29
TAACAGGCTGATGACCCCCAA 341
CGCAAGACGACGTATAGGGTC 29
GAGGACCGAACCCATATCTGT 29
AATGTACCGGGGCTCAAGCCA 253
GGGGGTAGAGCACTGGATGGG 110
ATGAAGGACGTGATACGCTGC 51
GAAGGGACAGTCGTGAGACAT 22
TTCAAGTCCTCCCAGGCCCAC 168
AGTCAGGGGTGAAATCCCGGG 36
ACCTGGGGAACTGAAACATCC 57
GAGCTGAGTTTTGATGGATAT 279
ACAATGGTGGTGACAGTGGGC 107
CAGAGGAAAGGACATCAAACG 21
GCGAACAGGATTAGATACCCT 11
GGTCGGAAATCGTTCGTCGAG 192
CTACACTCGGAATTCCACTCA 11
GCCAATCAAACTTGGAGATAG 15
AAAGGGAGTGAGAGACTCCCT 30
TCCGAATGGGGAAACCCACCT 32
TGTACACACCGCCCGTCACAC 18
TAAAGGGAGTGAGAGACTCCC 25
AAGCGAGTCTGAACAGGGCGT 29
GTATAGGGCCTGACGCCTGCC 11
ACTCGACCGAAGTGGGTGATA 251
AGCTCCTGCATATAGACCGTA 324
GTCTCAAGACGCGGGAGAGTA 155
GCGAACCTGGGGAACTGAACC 57
ACTGATGAAAATGGATTGACT 10
CCATTACTGACGCTGAGGTGA 11
TGTTGGCTTAGAAGCAGCCAT 86
TCGATGCATTAGACCCGAAAC 25
ATGGTAGGCTCAGAACGGTCG 74

TTACCTGACTTTGGTTTTCGG 18
ATCCCATTCCGAACTCGGCCG 85
AAACAGTACCTGAAACCGGAT 188
AAGCGCACGTAGGCGGACTTT 57
CGGCCCAGACTCCTACGGGAA 43
CAAACTTGGAGATAGCTGGTT 166
CGCGGTAATACGAAGGGGGCT 407
ACGCTGGCGGCAGGCTTAACG 25
TAACACGCACTGGAGGACCGA 14
AGGGTATGGCTGTTCGCCATT 24
ATCCTGTCTGAACATGGGTCG 1657
CTCAGCTGGGAGAGCACCTGC 563
ATTTCCGAATGGGGAAACCCA 19
TGAGCAGGGTTAGCCGGCACC 15
CATCAAACGAGACTCCGCTAG 86
TGGGTGATAGTCCCGTACACG 17
GCCGTAGCTCAGCTGGGAGAG 8047
TCGGCCGTGAAACGCTCGAGC 10
GAGGCAGGCGACCACGGTAGG 148
TGCGGACTTTTACGAAAGTCT 10
CCATTACTGACGCTGAGGTGG 20
TAAGAGCTGAGTTTTGATGGA 284
GAACCACGTTAATATTCGTGG 18
CGGCCCAGACTCCTACGGGAG 2735
CAACTGGAGAGTTTGATCCTG 10
ACTTGGAGATAGCTGGTTCTC 849
CAGCTGGGAGAGCACCTGCTT 493
CCACGCCGTAAACGATGAATG 134
GTTGTTCGGATTTACTGGGCG 291
ATACGTTCCCGGGTCTTGTAC 13
GACCAGGGGGTAGCGACTGTT 76
GGAAGACCACCACGTTGATAG 13
CGTAGCTCAGCTGGGAGAGAA 10
CCCCGCAAGGGGAGCGGCAGA 57
GACACGTGAAATCCTGTCTGA 26
ATAGCTCCTGCATATAGACCG 105
ATGCAGCTTACCGGTACTAAT 43
TAGTCCCGTACACGTAGAATA 360
TCCTAACCAAACTCCGAATAC 81
AACACATGCAAGTCGAGCGCC 14
CGAACCTGGGGAACTGAAAAA 70
GACCACGGTAGGGTCAGCGAC 2166
TTCGTGACGACATGTGTAGGA 310
GCCGTAGCTCAGCTGGGAGAA 38
AGCTTACCGGTACTAATAGCT 161
TGCATGGTTGTCGTCAGCTCG 13
CGGGTCCAGGACCGTGTATGG 37
AAGGGCCATCGTTCAACGGAT 99
CTAACCAAACTCCGAATACCG 53
CCGCGTTGGATTAGCTAGTTG 21
GAACTGCCTTTGATACTGGAA 118
CCTGCCCGGTGCTGGAAGGTT 17
ACGGATTTGACCTTCGGGTTT 30
GGGTGCTAACGTCCGTCGTGG 13

ACGCTGGCGGCAGGCTTAACA 785
TAGGCGGACTTTTAAGTCAGG 18
ATGAAGGCCTTAGGGTTGTAA 15
GAAGGCGCGCGATGGTAGGCT 12
CGGTCGCGGTTAGTGGAGACA 94
ACTGGACCATTACTGACGCTA 12
AGGATTAGATACCCTGGTAGT 68
GCGCACGTAGGCGGACTTTTA 87
TTAAGCCGGTAGGTGTAGGCG 77
GATAAAAGGTACGCCGGGGAT 863
AAAGTCGGTCATAGTGATCCG 134
TTTAATTCGAAGCAACGCGCA 24
CGGTCCCTATCTGCCGTGGGG 24
ATACCCTTTGATCCGACGATA 16
TGATCTTGCAGCGAAGCGGTT 226
GAGTAGGTCGCTGCCAGGTCT 6251
ATCAGCCACACTGGGACTGAG 55
TCGAGCAGAGACGAAAGTCGG 131
CTTAGATGACTAGAAAATCTG 17
AACAGTACCTGAAACCGGATG 797
ATGAGGGGCCGTAGCTCAGCT 222
CATGGGGGTGCGACTGATTAT 45
TGAGCAGGGTTAGCCGGCCCC 10105
TAGCGTTTGCGTCGGTATCTG 10
CAAAAGCCGTCTCAGTTCGGA 24
ACAATGAACTTTGGCGGACAC 16
CAAGGAGGCAGGCGACCACGG 83
AGACGACGTATAGGGTCTGAC 135
AGCCTGCCTGACTGCAAGACT 210
GGTACTAATAGCTCGATCGAC 5436
ACACCAACTTCGATCCGAAAA 41
GTGGCGAAGGCGGCTCACTGG 231
GGGCTAGCGTTGTTCGGATTT 145
CAATGGCATAAGCCTGCCTGA 48
AGTGGAATTCCGAGTGTAGAG 561
CGGCCGTGAAACGCTCGAGCG 10
CAGGATTAGATACCCTGGTAG 39
TATAATGGGTCAGCGACTTAG 68
AAAGCGCACGTAGGCGGACTT 77
TAGATCGCAGGCCAGTCAGCC 90
ACACGGACCAGACTCCTACGG 19
CGTGAGACAGTTCGGTCCCTA 162
AAAGCGGTACGTGAGTTGGGT 10
TAGTTTGACTGGGGTGGTCGC 22
TATAACGGTCCTAAGGTAGCG 10
GATGGGCTATGGGGACTCACC 2688
ACATAGGGGTTAGAAGCGAAC 127
CTGCAAGACTGACAAGTCGAG 47
TTCCTTGTCGGGTAAGTTCCG 244
CTGCCGCCAGCGTTCGTTCTG 39
ACGTTAATATTCGTGGGCCTG 103
TCCTGGCTCAGGACGAACGCT 16
GCGTTGGATTAGCTAGTTGGT 10
GCTAGTAGTGGCGAGCGAACG 66

GGGGCAACCCACCTTAGATGA 26
GGAAAGACCCCGTGCACCTTT 51
ATGTGCGTGGTAGCGGAGCGT 23
TCCCGCGTGGAAGGGCCATCG 10
ACTGGACCATTACTGACGCTG 20454
CTAGCCATGAGCAGGTTGAAG 28
GGTCCTAAGGTAGCGAAATTC 73
CCGACCTGCACGAATGGCGTA 143
GCCATCTCAGTTCGGATTGCA 93
GAGTGATGAAGGCCCTAGGGT 26
GTTCGATGCATTAGACCCGAA 56
GTAGAGGTGAGTGGAATTCCG 103
TGTGGCTAGGGGTGAAAGGCC 587
TTGTGAGTAGTTGGGGGTGGT 12
TTCCCTATCAGAGCCGTGGAA 38
GCCCGGTGCTGGAAGGTTAAG 19
GTGCATGGCTGTCGTCAGCTC 16
GGTTAGCCGGCCCCTAAGGCG 100
AGGTACTCCGGGGATAACAGG 87
TTGCGGACTTTTACGAAAGTC 14
TTAATATTCGTGGGCCTGCAG 32
AGCCTGCCTGACTGCAAGACC 19
GCCTGCCTGACTGCAAGACTG 101
GTATCTCTGGTGGACCTGTTG 49
CGGGCTGGGCTACACACATGC 11
CAGACCAGGGGGTAGCGACTG 108
GGCTGGACCGGAGACAGGTGC 695
TCAGAACGGTCGGAAATCGTT 293
GGAGGCGCGCAATGGTAGGCT 10
CTGGCTCAGAACGAACGCTGA 18
GGGGTGGAGCAGCTCGGTAGC 10
AAATCCTGTCTGAACATGGGT 205
TTAGGCGGAAGAGATTTTGGA 44
CCAGACTCCTACGGGAGGCAG 1000
CATACACCGAAGCTGTGGATG 25
AAAAGGTACGCCGGGGATAAC 211
AAGACCCCGTGCACCTTTACT 55
TAATCGCGGATCAGCATGCCG 110
CAGGTGGTGACGGATCGCGTG 14
CAGAACGTCGTGAGACAGTTG 10
GCGAACGCGGACCAGGCCAGT 2648
AGCTCAGCTGGGAGAGCACCC 13
ATTCCCCGTGAAGATGCGGGG 13
AGACAGGTGCTGCATGGCTGT 69
AGACGGAAAGACCCCGTGCAC 337
GTACTGGAGGACCGAACCCAT 2666
AACGCAGACTCAGTGAAATTG 99
AATTCCGAGTGTAGAGGTGAA 445
GATCCCAAAACAACCAGGATG 68
GTTGTCGTCAGCTCGTGTCGT 13
GACGCGGGAGAGTAGGTCGCT 919
CGGAGGCGCGCGATGGTAGGA 58
TTTACCAAAAACACAGGGCTC 18
AAGGAACTCGGCAAAATGCAC 531

CTCAAGCCATACACCGAAGCT 307
AGCGTTGTTCGGATTTACTGG 26
GCTGGAGCAGGTCCCAAGGGT 16
TCCAACGCAGACTCAGTGAAA 97
CTGGGCTACACACGTGCTACC 264
CCGCGGTGAATACGTTCCCGG 205
CGCGTTGGATTAGCTAGTTGG 12
ATGGTACTTCGTCTCAAGACG 137
TAAGCCGAGAGGAAGGTGGGG 14
TGGCGAAGGCGGCTCACTGGA 354
CGACTTAGTGTATCGAGCAAG 114
GACGGAAAGACCCCGTGAACC 15
TCCGAGTGTAGAGGTGAAATT 467
TGGCCCTTACGGGCTGGGCTA 29
GTCGACCGAATACCCCCGGGG 5187
ACGATCCATAGCTGGTCTGAG 30
TTGGACAATGGGCGCAAGCCT 1709
ATCCTGGCTCAGGACGAACGC 11
TCATGTTGGTGTTGAGACGGA 22
CAGAACGTCGTGAGACAGTTA 12
GGGATGGACGTATCTCTGGTG 736
AGCTCAGCTGGGAGAGCACCT 2622
GGAACTGCCTTTGATACTGGA 161
GACATCAAACGAGACTCCGCT 335
TCCAAAAGCCATCTCAGTTCG 62
TTTGAGAGGATGATCAGCCAC 17
AAGGCGCTGTGCTAACCGCAA 18
CTGCCCGGTGCTGGAAGGTTA 14
AAAGCGAGTCTGAACAGGGCG 18
GAATTCCGAGTGTAGAGGTGA 268
CGACCTGGTGGTTATGGCGGC 10
CTGGCTCAGAACGAACGCTGG 3373
GGCTAGCGTTGTTCGGATTTA 123
ACAGGGCGTTCAGTTCGATGC 364
TCGGCCCATGTGGGCCGCCCC 21
CTGGGCTACACACGTGCTACT 42
GAACTCGGCAAATTGCACGCG 14
TATCCTTCAGTTAGGCTGGAC 12
TGGGCACTCTAAGGGGACTGC 977
TGGGCCGTGAAACGCTCCAGC 14
CAATAAGGTTAGCGGGCTTTT 64
GGCCGTAGCTCAGCTGGGAGA 10311
GGTGACAGTGGGCAGCGAGCA 308
TTAAACATTCCGCCTGGGGAC 33
TTAGCCGTCGGGGTGTTTACA 183
GTGAAACGCTCCAGCGCCAAT 1072
ACCGTCTTACTGATCCTAACC 163
GGGTGAGTAACGCGTGGGAAC 18
TGACATCCCGGTCGCGGTTAG 156
ACCACGTTAATATTCGTGGGC 10
TATGTGCGTGGTAGCGGAGCG 27
GAACCTTACCAGCCCTTGACA 387
CGGAGGCGCGCGATGGTAGGG 27
GGTGCGAAAGCGTGGGGAGCA 10

AGCAGTGGGGAATTTTGGACA 49
TGGTTTTACCCGAAGGCGCTG 201
TTAGAACATAGATCGCAGGCC 29
CAAGGCGACGATCCATAGCTG 194
TAGATACCCTGGTAGTCCACG 277
CTCACTGGACCATTACTGACC 149
GCAGAAGTCCTTGAGTAGGGC 744
GAGGGGCCGTAGCTCAGCTGG 143
GGAAGTGAGAATGCTGACATG 103
AGAAGAAGCCCCGGCTAACTT 21
AAGGGAGTGAGAGACTCCCTC 41
AAGGTCCCTAAGTTATGGCTA 178
GCCCATCAGGGCCGACGGCCG 40
ATGGTTGTCGTCAGCTCGTGT 24
CGACTGTTTAGCAAAAACACA 13
ATGGCTAAGTGGGAAAGGATG 127
GTATCTCGAGAAGCTGGTCTT 11
CAATGAGAGTGATCAAGTGTC 12218
ACGGCCCAGACTCCTACGGGA 6099
CCTGTCTGAACATGGGTCGAC 2168
GTGAGAATGCTGACATGAGTA 335
AAGCTGTGGATGCACGTATGT 41
GTGAGTTGGGTTCAGAACGTC 18
TCGACTCATCGCATCCTGGGG 482
CAACCCACCTTAGATGACTAG 33
ACCTGGGGAACTGAAACATCG 17
CGAAAGTCGGTCATAGTGATC 142
TAGGCTCAGAACGGTCGGAAA 248
CGGCTCATCGCATCCTGGGGC 16
GGCACAGACCAGGGGGTAGCG 12
GGGAGTACGGTCGCAAGATTA 102
TAGAAGCGAACCTGGGGAACG 21
TAGAGCACTGGATGGGCTATG 23
TTACTGGGCGTAAAGCGCACG 1941
GTGGGTGTAGGAATATTGACA 65
AGTGTATCGAGCAAGCTTAAG 21
AGGACGTGATACGCTGCGATA 31
CGGAAAGACCCCGTGCACCTT 101
CCGTGAAACGCTCCAGCGCCA 11395
TTAGATACCCTGGTAGTCCAC 238
ACAAAGGCAAAGAACAGGCGC 46
GGCTAAGTGGGAAAGGATGTG 126
GCGAAGGCGGCTCACTGGACC 650
GCAGGTTGAAGGTACGGTAAC 932
GCCATGAGCAGGTTGAAGGTA 436
TAAACATTCCGCCTGGGGAGT 377
GGCTGCACCCGATCCCATTCC 14
ACGGCCCAGACTCCTACGGGG 15
GTTCGGATTTACTGGGCGTAA 44
CGTACCCTAAACCGACACTGG 639
ACTGGAGGACCGAACCCATAT 429
AGTTGGTTTTACCCGAAGGCG 34
CTCACTGGACCATTACTGACT 30
CAGAAGTCCTTGAGTAGGGCG 739

GGCATAAGCCTGCCTGACTGC 293
CTCAAGACGCGGGAGAGTAGG 239
ACGGAATAACTCAGGGAAACT 246
GGACTCACCGTCTTACTGATC 469
TTACTGGGCGTAAAGCGCACA 14
CCTTGAGTAGGGCGGGACACG 20
TGACGCCTGCCCGGTGCTGGA 526
CCGTGAAACGCTCCAGCGCCG 23
TTAAGAGCTGAGTTTTGATGG 265
CGTGAGTTGGGTTCAGAACGT 21
ATCCAGCCATGCCGCGTGAGT 12
ACCTGGGGAACTGAAACATCA 130
AGAATAGCAGAAGTCCTTGAG 472
AACAACCCTGACCACCATCTA 15
TGAAACTCAAAGGAATTGACG 11
AAGCCCCGGCTAACTTCGTGC 147
TAGCTTTACACTGGCATTCGT 88
AGAGATTTTGGACGGTTTAGA 15
CCCGCGTTGGATTAGCTAGTT 47
ATTTGGTTGCGGGGGCAGGAT 32
AGGATGTTGGCTTAGAAGCAG 1130
GTGCATGACCGATAGCGAACC 288
ATGATCCGCCACACTGGGACT 25
GATACCCTGGTAGTCCACGCT 23
CGGAGCGGCTGCACCCGATCC 1950
CGGCCCAGACTCCTACGGGAC 49
ACGAAAGTCGGCCATAGTGAT 10
GCGGTAATACGAAGGGGGCTA 205
AGGAATTGACGGGGGCCCGCA 16
CTGAAGGCCGCAGGTTCAAAT 17
TTAAGTCCCGCAACGAGCGCA 32
GACAAGTCGAGCAGAGACGAA 16
CCATTACTGACGCTGAGGTGC 15363
AGGCGCTGTGCTAACCGCAAG 26
CGGAGAAGAAGCCCCGGCTAA 96
TTAACACATGCAAGTCGAGCG 29
GGCCCATCAGGGCCGACGGCC 42
GCTAGTAATCGCGGATCAGCA 146
CTCCAAAAGCCATCTCAGTTC 55
GCAAGCCTGATCCAGCCATGC 221
GGAGTGAGAGACTCCCTCGCC 41
GGTGAGTAACGCGTGGGAACG 18
TAGAGCATACCAAGGCGCTTG 10
GCGACTGGGGTGAAGTCGTAA 12
ACCGGGGAGTACTAGTCGGCA 808
TAGCTCGATCGACTTGATCAC 41
TAGGTGTAGGCGCAGCGAAAG 38
GACCAGACTCCTACGGGAGGC 15
AACGCATGAAGCTTACCGGTA 61
CGGTTAGTGGAGACACTATCC 40
AAGCCATCTCAGTTCGGATTG 98
CCTGGCTCAGGACGAACGCTG 13
GGTGCTGGAAGGTTAAGAGGA 15
CGTGCATGACCGATAGCGAAC 316

GGGCTGGGCTACACACGTGGT 28
GTTGGGTTAAGTCCCGCAACG 24
ACAGGATTAGATACCCTGGTA 34
CTGAAACCGGATGCCTACAAA 147
AGGATGTTGGCTTAGAAGCAA 36
AACGCATTAAACATTCCGCCT 459
GATACCCTGGTAGTCCACGCC 363
GCCGCGGTGAATACGTTCCCG 197
AAAGTCCAAGGGTTCCTGCTT 15
CTTCGTGCCAGCAGCCGCGGT 23
AAGTCGGAATCGCTAGTAATC 290
AGAAGCCCCGGCTAACTTCGT 62
TAAGTGGGAAAGGATGTGAGG 101
GTTTGAGAGGATGATCAGCCA 12
CGTGAGACATCCTGGAGGTAT 111
GCCGTAGCTCAGCTGGGAGAC 15
TGGTTGCGGGGGCAGGATTTG 31
GAGAGGATGATCAGTCACACT 14
GGAAAACTCGACCGAAGTGGG 17
GAAGCGTGACCTCACTATGGG 192
ATCTGTTGCAATAGATCGGGA 27
TCAGGGCCGACGGCCGGTCGG 23
AACCTGCGGCTGGATCACCTC 31
ACGCTGGCGGCAGGCTTAACC 27
ACGTAGAATAGCAGAAGTCCT 1633
ATGACCCCCAAGAGTCCATAT 12
GGGTTCAGAACGTCGTGAGAC 141
CAATGGTACTTCGTCTCAAGA 21
TGCAGGTGGTGACGGATCGCG 79
TCGGATCAACTGAAGAGTTGA 14
TGAAGGCCCTAGGGTTGTAAA 1509
TCTAAGGTCCCTAAGTTATGG 381
GCAAGACGACGTATAGGGTCT 37
TGAAGGAACTCGGCAAATTGC 10
GGGTTAGAAGCGAACCTGGGG 812
CGGTCCCTATCTGCCGTGGGT 1339
AACTGGAGAGTTTGATCCTGG 12
ATCTCCAAAAGCCATCTCAGT 10
GGCGGACTTTTAAGTCAGGGG 68
TCGAGAAGCTGGTCTTTCTGC 306
CCGGTACTAATAGCTCGATCG 5477
GCCGACCTGGTGGTTATGGCG 11
ACTGGACCATTACTGACGCTC 10
ACCTGCACGAATGGCGTAACG 100
GGTACTTCGTCTCAAGACGCG 109
CGGTCGCGGTTAGTGGAGACC 11
AGAAGCGAACCTGGGGAACGG 14
TCCTTGTCGGGTAAGTTCCGA 225
ACTTGATGAGGGGCCGTAGCT 170
AAGGGGGCTAGCGTTGTTCGG 676
CAACGGATAAAAGGTACTCCG 39
GAGTAGGTCGCTGCCAGGTCG 18
TAAAGCGCACGTAGGCGGACC 12
TTAAGTCAGGGGTGAAATCCC 23

ATACCCTTTGATCCGACGATC 14
CCATGAGCAGGTTGAAGGTAC 423
CATGTGTAGGATAGGTGGTAG 971
AGCAGAGACGAAAGTCGGTCA 35
GAGATAGCTGGTTCTCCGCGA 23
GAGGGGAGTGAAACAGTACCT 18
GAAATCCTGTCTGAACATGGG 90
GATAGTCCCGTACACGTAGAA 63
CGGCCGTGAAACGTTCCAGCG 14
CCTGGTAGTCCACGCTGTAAA 25
TGAGTAGTTGGGGGTGGTTTT 11
CCATTACTGACGCTGAGGGGC 88
GACTGGGGTGAAGTCGTAACA 20
GACGGTAACCGGAGAAGAAGC 170
GGTTAGCCGGCCCCTAAGGCC 11
GGGCTTGTAGCTCAGTTGGTT 117
GTGAATACGTTCCCGGGCCTT 167
CGTACGGCGCGTGAGCGAGAA 13
AGGGTTGTAAAGCTCTTTCAC 1626
TTGAAGCAGGGGCGCCAGCCT 262
TACCAAGGCGCTTGAGAGAAC 98
CCGGGGATAACAGGCTGATGA 10
AACGACTTCCCCGCTGTCTCC 74
ATCGCTCAACGGATAAAAGGT 135
GCGTACCTTTTGTATAATGGG 11
TATAATGGGTCAGCGACTTAT 14
AACTGCGTTGAAGGAACTCGG 1123
GGGGGTCGTCGGTTCGATCCC 512
GCGGGGTTCCTGCGGTTAGAC 665
TAAAGCGCACGTAGGCGGACT 247
GTAGAGGTGAAATTCGTAGAT 182
ATACCCTTTGATCCGACGATT 1859
GGGCAACCCACCTTAGATGAC 26
CGGTCCCTATCTGCCGTGGGC 13
CACTGGTCTAAATAAGGGTCT 12
ATTGGACAATGGGCGAAAGCC 36
CCTGCCTGACTGCAAGACTGA 84
TCTCATGTTTGTGTTCTTCGC 21
ACTGGATGGGCTATGGGGACT 3002
GGCGAAGGCGGCTCACTGGAC 430
GATAAAAGGTACGCCGGGGAA 10
TCTGAGAGGATGATCAGTCAC 12
ACTGGACCATTACTGACGCTT 11
ATCGTGAAGAGAAGATGTAAT 25
CGGGCTGGGCCACACACGTGC 13
TTTGATACTGGAAGTCTTGAG 33
CGAAAGCGTGGGGAGCAAACA 12
TTCCCTATCAGAGCCGTGGAC 11
CTAGGTCGTCGGCCCATGTGG 21
CTAAGGGGACTGCCGGTGATA 163
GGCTGGACCGGAGACAGGTGA 11
AGGATCCCAAAACAACCAGGA 70
GGGCCGTACCGCAGCTGACGC 93
AATAGATCGGGATGACTTGTG 173

GCACACGCTTGATAAGCGTGG 11
GAACTGCGTTGAAGGAACTCA 10
CGGGGAGTACTAGTCGGCAGA 335
GGAATTCCGAGTGTAGAGGTG 404
CGAGAGGACCGGGATGGACGG 19
GTCCTCATGGCCCTTACGGGC 118
GCAGCTTACCGGTACTAATAG 45
GTCGTGAGACATCCTGGAGGT 27
GTCGGGGTGTTTACACTTCGG 66
ATAAGGTTAGCGGGCTTTTTT 17
AGGCGGAAGAGATTTTGGACG 17
TTCGAAGCAACGCGCAGAACC 70
GAAACCAAGTGATCTAGCCAT 11
ATAAAGTGGAACGAGTTGGAA 10
GGGCGGCATTGTCTGCGGATC 16
CGTCCGTCGTGGAGAGGGCAA 38
GGCCGGGAAACGCTCCAGCGC 19
GTGGTTGACAGGTTGGTTTGA 10
CGGTTAGACGGAAAGACCCCG 49
AGTGATGAAGGCCCTAGGGTT 34
TCCCGCAACGAGCGCAACCCT 95
CTTACCGGTACTAATAGCTCG 265
GGGGAACCTGCGGCTGGATCA 43
GGACCGGGATGGACGTATCTC 9421
GGTACCCCGGGGATAACAGGC 59
CAGGATGTTGGCTTAGAAGCA 2044
GTCGGGGAGGTGCGAATACCC 313
TAGCAGAAGTCCTTGAGTAGG 86
AAGCGAACCTGGGGAACTGAC 74
GGTCGTCGGCCCATGTGGGCC 17
TTAATTCGAAGCAACGCGCAG 25
CCCACCTGAAAACGAGTATTC 10
ACCTTACCAGCCCTTGACATC 133
GGAGGCAGGCGACCACGGTAG 139
GCCGTAGGGGAACCTGCGGCT 29
CGTAGGGGAACCTGCGGCTGG 29
TTGGTGGATGCCTTGGCATGC 47
GTACCGGGGCTCAAGCCATAC 808
ACGGAGGCGCGCGATGGTAGC 18
CATTGGGACTGAGACACGGCC 42
ACTATAGCTTTACACTGGCAT 15
TGGTGGATGCCTTGGCATGCA 37
GCGGGAGAGGAGGTCGCTGCC 13
GTAAAGCTCTTTCACCGGTGG 12
AGAGAACTGCGTTGAAGGAAG 10
AAGGAATTGACGGGGGCCCGC 24
GGATTGCACTCTGCAACTCGA 334
GGTGAAAAGCACCCCGACGAG 35
AGGGCGGCGGCGCCGGCAGCG 41
CCATTACTGACGCTGGGGTGC 16
TCTCTTTCTTCATTGTTGATT 22
GGTAGTTTGACTGGGGCGGTC 156
GTAATCGCGGATCAGAATGCC 11
CAGGATGTTGGCTTAGAAGCG 28

CCGCAAGGGGAGCGGCAGACG 55
GAACTGCGTTGAAGGAACTCG 1101
CTGTCCCTAGTACGAGAGGAC 99
CCGGAACTGCCTTTGATACTG 159
CGAGAGGACCGGGATGGACGA 32
GCGTTCCGTAAGCCTGTGAAG 17
ACTCGGCCGGGAAACGCTCCA 18
GATAGCTGGTTCTCCGCGAAA 93
GGTAGTTTGACTGGGGTGGTC 13
GGGGCTGTAGCTCAGCTGGGA 412
GACGTATAGGGCCTGACGCCT 12
GACTTGTGGCTAGGGGTGAAA 921
CAAGACGCGGGAGAGTAGGTC 334
GTAATCGGATCAACTGAAGAG 18
GTAGGTGTAGGCGCAGCGAAA 62
CGCCTGGGGAGTACGGTCGCC 12
GTAAAGCTCTTTCACCGGTGA 994
GTCAGCGACTGGGGTGAAGTC 411
AGCCCAAGGTTTGTCCTGGGT 14
AGAGAACTGCGTTGAAGGAAA 30
AAGCAGGGGGTCGTCGGTTCC 24
CGGCCGTGAAACGCTCCAGCG 35314
AAGCGAACCTGGGGAACTGAT 17
TAAGTCCCGCAACGAGCGCAA 79
ATGTGAGGATCCCAAAACAAC 24
GGCTGTTCGCCATTTAAAGCG 26
AGAGTGATCAAGTGTCTTAAG 213
GCGGGTAGTTTGACTGGGGCG 12
GGCCCGCGTTGGATTAGCTAG 104
GGGGAGTTTGACTGGGGCGGT 192
TTGTACACACCGCCCGTCACA 19
CCACCTTAGATGACTAGAAAA 69
ATCGGCCCGCGTTGGATTAGC 270
TACGCTGCGATAAGCGTCGGG 56
TGGCGTAACGACTTCCCCGCT 13
AATCGCGGATCAGCATGTCGC 20
ATTTCCGAATGGGGCAACCCA 10
CGACCACGGTAGGGTCAGCGA 2121
TGCCGCGGTGAATACGTTCCC 212
CGTACCTTTTGTATAATGGGT 14
ATAGCTGGTTCTCCGCGAAAT 254
TGCATATAGACCGTACCCTAA 182
GTGACGACATGTGTAGGATAG 154
CAACCAGGATGTTGGCTTAGA 21
AGTGGCGAAGGCGGCTCACTG 301
ATGATCGGCCCGCGTTGGATT 365
AGCTGAGTTTTGATGGATATT 262
TCAGTTAGGCTGGACCGGAGA 41
ACAATGGGCGCAAGCCTGATC 194
TGGTGACAGTGGGCAGCGAGC 202
GGCCTTAGGGTTGTAAAGCTC 29
GGCGGAAGAGATTTTGGACGG 15
GGGTAGAGCACTGGATGGGCT 39
CGGAGGCGCGCGATGGTAGCC 12

AACTGCCTTTGATACTGGAAG 52
TAGCGAACCAGTACCGTGAGG 99
GGAATAACTCAGGGAAACTTG 88
ATGGGGGTGCGACTGATTATA 40
AAGTCCTTGAGTAGGGCGGGA 746
GGAGGCGCGCGACGGTAGGCT 10
GATAGCGAACCAGTACCGTGA 118
CGACGTTCTGAACCCAACTCA 10
TTGACTGGGGCGGTCTCCTCC 26
AGGGGGTAGCGACTGTTTACA 27
ATGCCGCGTGAGTGATGAAGG 24
TGCCCGGTGCTGGAAGGTTAA 16
GGACCGAACCCATATCTGTTG 20
CGTAGATATTCGGAGGAACCC 22
TCCTGGCTCAGAGCGAACGCT 10
GTCGACCACGATCCAAGCCTA 18
TCGGAAGAACACCAGTGGCGA 43
ACCTGGTGGTTATGGCGGAGC 4741
TACTGGAGGACCGAACCCATA 2092
TTACCGGTACTAATAGCTCGA 141
GAGCATACCAAGGCGCTTGAG 10
GATGAAGGCCTTAGGGTTGTA 12
GGACACGTTTCTTGGTAAGAA 57
AAACTTGGAGATAGCTGGTTC 158
AGCGTAACAGCTCACTGGTCT 301
GGAGGAACACCAGTGGCGAAG 1536
TGAGAGACTCCCTCGCCGAAA 24
GAGGCGCGCGATGGTAGACTC 15
CGACCACGATCCAAGCCTAAG 34
TGCAGCGAAGCGGTTCCAGGA 85
TCGTGGAGAGGGCAACAACCC 11
GATACGGCCCAGACTCCTACG 15
ATCTGAGCAGGGTTAGCCGGC 496
GAAGGGCCATCGTTCAACGGA 55
CTGGGTTCAGAACGTCGTGAG 29
ATGAAGTTGGAATCGCTAGTA 100
TACCTGAAACCGGATGCCTAA 12
GGGGCTCAAGCCATACACCGA 1292
GGTTGCGGGGGCAGGATTTGA 28
GGGGTTAGAAGCGAACCTGGG 805
CGAGGCGCTGACACGGATTTG 84
ACTTTGAAGCAGGGGCGCCAG 596
AAACCGACACTGGTGGACTGG 22
CCTGCAGGTGGTGACGGATCG 117
CCGGGTGTGGAAGTGCGGCAA 503
CTGGTAGTCCACGCCGTAAAC 196
TTGAGTATGGTAGAGGTGAGT 29
TCAAGACGCGGGAGAGTAGGT 309
TGCAAGCAGGGGGTCGTCGGT 90
ATAGTGAACCAGTACCGTGAG 33
AGGCGCTTGAGAGAACTGCGT 60
GTAGTGGCGAGCGAACGCGGA 81
CAAAGGCAAAGAACAGGCGCA 41
CTGACGCTGCTGGCCCTGCGC 25

CCGCAGACGAGGCGCTGACAC 14
ATTCCTTGTCGGGTAAGTTCC 294
CCTGGTAGTCCACGCCGTAAA 201
TCGTGCCAGCAGCCGCGGTAA 20
CTTCCCCGCTGTCTCCAACGC 424
GTAGAGCGTCGACCGAATACC 1563
GCTTGTAGCTCAGTTGGTTAG 123
CCGGGGGTAGAGCACTGGATG 190
ACACACGTGCTACAATGGTCG 10
TTGGATTGATCTTGCAGCGAA 22
CTGTCGTCAGCTCGTGTCGTG 77
CTGATGACCCCCAAGAGTCCT 13
CGAAGCGGTTCCAGGAAATAG 10
AGCGGGAAACCCACCTGAAAA 13
CTCGATGTCGACTCATCGCAT 27
GATTGCACTCTGCAACTCGAG 336
GTCCACGCCGTAAACGATGAA 32
AGAAGCTGGTCTTTCTGCTGA 13
TCGCAGGCCAGTCAGCCTGAC 10
GGACCTGTTGTGGCGCCAGCC 3933
ACGCATGAAGCTTACCGGTAC 62
ACAGGATCTGTCCCTAGTACG 214
TATAGCTTTACACTGGCATTC 13
ATGAATGTTAGCCGTCGGGGG 86
GCGGGGGCAGGATTTGAACCT 14
CTTGACATCCCGGTCGCGGTT 221
GACTCATCGCATCCTGGGGCT 440
ATTCCGCCTGGGGAGTACGGT 2328
ATACCCTGGTAGTCCACGCCG 277
AGCTCGTGTCGTGAGATGTTG 218
GAATACCAAGGCGCTTGAGAG 58
GAGATACGGCCCAGACTCCTA 12
GCGGGATGGAGCAGCCCGGTA 15
AGCGAACCTGGGGAACTGAAG 33
GTAGTCCACGCCGTAAACGAT 55
CCTGTGAAGGGACAGTCGTGA 45
GTAGCGACTGTTTACCAAAAA 12
GCCCCGGCTAACTTCGTGCCA 80
ACGGACGGGATAACCGCTGAC 27
ATCAGAGCCGTGGAAGACCAC 16
TGCCTGACTGCAAGACTGACA 78
ATTAAAACTCAAAGGAATTGA 56
GCCGTGAAACGCTCCAGCGAC 17
CATCTAAGGTCCCTAAGTTAT 12
CAGACTCAGTGAAATTGAATT 80
AGATTAAAACTCAAAGGAATT 72
GACCACCACGTTGATAGGCCG 127
TCTCGAGAAGCTGGTCTTTCT 241
CTGACATGAGTAACGATAAAG 16
CGGATAAAAGGTACCCCGGGG 79
TATCGGCAAATGATCGGCCCG 218
AACCAAACTCCGAATACCGGG 38
GTAGCTCAGCTGGGAGAGCGC 19
ACAGGATCTGTCCCTAGTACA 16

CTGCACGAATGGCGTAACGAC 41
ATACATAGGGGTTAGAAGCGA 11
CAATGGTGGTGACAGTGGGCA 96
TTGGCGGACACGTTTCTTGGT 121
GCAACAACCCTGACCACCATC 49
AACGTCCGTCGTGGAGAGGGC 195
TGCTACGGAATAACTCAGGGA 273
AACTCGGCCGTGAAACGCTCC 6123
GCAAAATGCACGCGTAACTTC 30
CTTGAGAGTTTGATCCTGGCG 20
ACATCTAAGTACCCAGAGGAA 180
CGGGCTGGGCTATACACGTGC 19
CCTTAGATGACTAGAAAATCT 19
ACGTACTGGAGGACCGAACAC 11
ACGCATTAAACATTCCGCCTG 1950
GGTTAGGCGGAAGAGATTTTG 11
CTGATGACCCCCAAGAGTCCC 39
AGCGAAATTCCTTGTCGGGTA 1171
GATTTCCGAATGGGGAAACCC 19
ACACCGCCCGTCACACCATGG 47
TTCTAAGGAAGATCGAGAATT 40
TAGAATAGCAGAAGTCCTTGA 1147
AGAGGGCAACAACCCTGACCA 19
ATCGGCAAATGATCGGCCCGC 1555
CGTGACCTCACTATGGGCAAC 15
CGGTCGCAAGATTAAAACTCA 171
GATGTCGGCTCATCGCATCCT 13
GCACAGACCAGGGGGTAGCGA 40
TGGCATTCGTGACGACATGTG 11
TCTCCAAAAGCCATCTCAGTT 54
AGCGAACGCGGACCAGGCCAG 1666
CGGGGCTCAAGCCATACACCG 1554
AGCGAACCTGGGGAACTGAAA 17565
CGTCGTGGAGAGGGCAACAAC 22
GGCACTCTAAGGGGACTGCCG 252
AGTCGAGCGCCCCGCAAGGGG 65
CTCGGCCGTGAAACGCTCCAC 17
GGAGCCCAAGGTTTGTCCTGG 56
CCAAGGGTATGGCTGTTCGCC 2294
TTCAGTTGGGCACTCTAAGGG 133
CAAACTCCGAATACCGGGGAG 15
AGGATGATCAGCCACACTGGG 1863
TAACCGCTGAAGGCATCTAAG 15
CTTCGGGGGAAAGATTTATCG 23
GGGGTTCCTGCGGTTAGACGG 468
ACAAGGTAGCCGTAGGGGAAC 28
TAAGTACCCAGAGGAAAGGAC 757
GTACCCTAAACCGACACTGGT 479
ACCAAGGCGACGATCCATAGC 340
GGGCTGGGCTACACACGTGCA 29
CGTAAACGATGAATGTTAGCC 235
GCGAACCTGGGGAACTGAAGC 23
AATTCCTTGTCGGGTAAGTTC 171
AGAGCACCTGCTTTGCAAGCA 10

TGTTCGGATTTACTGGGCGTA 150
TAGATATTCGGAGGAACACCT 35
AGTACCTGAAACCGGATGCCT 6527
ACCTCGATGTCGACTCATCGC 28
ACGGTCGGAAATCGTTCGTCG 298
GGGACTCACCGTCTTACTGAT 458
TTACACACCAACTTCGATCCG 14
CAAGAGTCCATATCGACGGGG 14
GTTCAAATCCTGCCCCCGCAA 20
CTAACTTCGTGCCAGCAGCCG 16
GTCAAGTCCTCATGGCCCTTA 59
ACCCTAAACCGACACTGGTGG 546
GGAGTACGGTCGCAAGATTAA 126
TGTCGGGTAAGTTCCGACCTG 60
TTCGATCCCGTCCGGCTCCAC 1686
AAGAGAAACGTGGGCGGCATT 12
GAATGTTAGCCGTCGGGGTGT 445
GCGAAAGCGAGTCTGAACAGG 24
TAGAGGTGAAATTCGTAGATA 97
CTTTCACCGGTGAAGATAATG 12
TACCAAAAACACAGGGCTCTG 16
AGAGGTGAAATTCGTAGATAT 72
ATCGAGAATTGGAAAGAGGCA 14
GTCAGCGACTTAGTGTATCGA 172
AGTCCTCATGGCCCTTACGGG 116
ATGTTAGCCGTCGGGGTGTTT 66
CGGAAATCGTTCGTCGAGTGC 90
CGTAGATATTCGGAAGAACAC 43
TAGATATTCGGAGGAACACCC 35
TTTAGGTAGAGCGTCGACCGA 136
AAACGGCGGCCGTAACAATAA 11
GGACCATTACTAACGCTGAGG 10
TACTAGTCGGCAGACACACGG 15
TGAATGTTAGCCGTCGGGGGG 25
CTGGATGGGCTATGGGGACTC 2959
GGGGTGGAGCAGCCCGGTAGC 208
GTCCCTATCTGCCGTGGGTGT 256
GACTTAGTGTATCGAGCAAGC 113
GGGCTGGGCTACACACGTGCG 155
CCACCTGAAAACGAGTATTCC 10
TACGGAATAACTCAGGGAAAC 159
GGAGACACTATCCTTCAGTTA 13
ACCTGCGGCTGGATCACCTCC 126
TGAAGCAGGGGCGCCAGCCTT 92
TAGCGACTGTTTACCAAAAAC 17
TCGGCCGTGAAACGTTCCAGC 14
CGAAGTGGGTGATAGTCCCGT 297
CGGAGGCGCGCGATGGGAGGC 11
GAGCAGAGACGAAAGTCGGTC 126
CGATCCATAGCTGGTCTGAGA 14
CTATTTAGGTAGAGCGTCGAC 25
GCGGACCAGGCCAGTGGCTTT 884
AGATCGCAGGCCAGTCAGCCT 69
TCGGCAAAATGCACGCGTAAC 59

CCACGTTGATAGGCCGGGTGT 18
GTGGCTAGGGGTGAAAGGCCA 273
CTGGGCTACACACGTGATACA 12
AATGCGTACGGCGCGTGAGCG 11
ACGCTGCGATAAGCGTCGGGG 90
CGAAGGGGGCTAGCGTTGTTC 561
AGCTGGTTCTCCGCGAAATCT 235
TGAAATTGAATTCCCCGTGAA 10
AAGGAGGCAGGCGACCACGGT 89
GAACTCGGCAAAATGCACGCG 452
TAGTAATCGCGGATCAGAATG 11
TCGTGCAGGTCGGAACTTACC 11
TTAGCCGGCCCCTAAGGCGAG 41
GGGGCCATAGCTCAGCTGGGA 1434
CTCGGCAAATTGCACGCGTAA 10
CATTACTGACGCTGAGGCGCG 18
AACCCACCTTAGATGACTAGA 40
GCATTGTCTGCGGATGGTTCG 38
TTTGTTGGAGCAACGCTGGAT 47
CGAGAGGACCGGGATGGACGT 4691
TATATACGGACGGGATAACCG 85
AGTGAACCAGTACCGTGAGGG 36
AGAACCTTACCAGCCCTTGAC 916
GGGCTATGGGGACTCACCGTC 1406
CGCGTAGAACCTTACCAGCCC 12
CGGGGAGTACTAGTCGGCAGC 18
TTATGGATGTCTAACTGCGGC 52
CCAGGGGGTAGCGACTGTTTA 1922
ACGTAGGCGGACTTTTAAGTC 50
GGTGTGGAAGTGCGGCAACGC 255
AAGCCTGCCTGACTGCAAGAC 256
AGGTCGTCGGCCCATGTGGGC 19
TCGGCCCGCGTTGGATTAGCT 465
ACATTGGGACTGAGACACGGC 43
AGTAGTGGCGAGCGAACGCGG 68
CGTCGGGGAGGTGCGAATACC 515
GGGCGGCATTGTCTGCGGATA 17
GATAAAAGGTACCCCGGGGAT 71
TCTGCAACTCGAGTGCATGAA 15
TACTGATGAAAATGGATTGAC 10
CCAGTCAGCCTGACGATCGCT 37
ACGGGCTAGGCTACACACGTG 15
AATGGGCGCAAGCCTGATCCA 103
CTCGTGTCGTGAGATGTTGGG 255
GCTGTGGATGCACGTATGTGC 358
CGGCCGTGAAACGCTCCAGCC 14
ACCAGCCCTTGACATCCCGGT 91
GTCGGTTCGATCCCGTCCGGC 533
AAGCGAACCTGGGGAACTGAA 7005
AAAGACCCCGTGCACCTTTAC 44
AAGCAGGGGGTCGTCGGTTCG 359
TTTCTAAGGAAGATCGAGAAT 12
CCGGTCGCGGTTAGTGGAGAC 221
CGTAGTCGATGGGAACCACGT 60

ACGGAGGCGCGCGATGGTAGA 43
TGGGGTAAAGGCTCACCAAGG 26
AAAGGCGTAGTCGATGGGAAC 270
CGTATGTGCCCTTCGGGGGAA 49
GCAGACGAGGCGCTGACACGG 15
GTGTAGAGGTGAAATTCGTAG 108
CAGGATGTTGGCTTAGAAGCT 27
GTCCCTAGTACGAGAGGACCG 42
GGGCGGCATTGTCTGCGGATG 3407
TCAAATGAATTGACGGGGGCC 17
TGAGTGGAATTCCGAGTGTAG 283
ACTCTTGGGGGTCATCAGCCT 20
TTGTAAAGCTCTTTCACCGGT 786
GAAGTGAGAATGCTGACATGA 111
TTTCTCTTTCTTCATTGTTGA 20
CGCAGAACCTTACCAGCCCTT 3866
GCCTTGGCATGCACAGGCGAT 159
CTTAGGGTTGTAAAGCTCTTT 14
CACGCACTGGAGGACCGAACC 30
GTGCGAATACCCTTTGATCCG 20
ATCTGTCCCTAGTACGAGAGG 80
AGACATCCTGGAGGTATCGGA 117
TCCGCCACACTGGGACTGAGA 20
AAAGGTACTCCGGGGATAACA 80
CATAGATCGCAGGCCAGTCAG 224
AGAGAACTGCGTTGAAGGAAC 4416
CAATAACGGTCCTAAGGTAGC 21
GGACGGGATAACCGCTGAAGG 2330
GGGAACCACGTTAATATTCGT 23
CGCCTGGGGAGTACGGTCGCA 2767
ACGGAGGCGCGCGATGGTAGG 37882
TAATCTGAGCAGGGTTAGCCG 119
ATCGAAGTTGGTGTGTAAATA 16
GACTTGTGGCTAGGGGTGAAC 25
GGGCGCCAGCCTTTGTGGAGT 10
GTATAGGGTGTGACGCCTGCC 12
GGCATCTAAGCGGGAAACCCA 50
ATCGACTTGATCACTCCCATT 609
GAACTTTGGCGGACACGTTTC 19
TGTGTAGGATAGGTGGTAGAC 996
CTATCTGCCGTGGGTGTAGGA 114
AAGCGAACCTGGGGAACTGAG 45
CGCCATCACCGATTGTATCTC 16
TTGTTCGGATTTACTGGGCGT 191
GACTCCGCTAGTAGTGGCGAG 12
GAAAGACCCCGTGCACCTTTA 43
TCTGACGCCTGCCCGGTGCTG 1211
CCAAAAGCCATCTCAGTTCGG 70
ACTTTGGTTTTCGGATCGAAG 21
AACTCGGCAAATTGCACGCGT 14
GACGGGGGCCCGCACAAGCGG 184
AGTTGGAATCGCTAGTAATCG 366
TGAGGATCCCAAAACAACCAG 29
GGGACTGCCGGTGATAAGCCA 53

TGGAGCAGCCCGGTAGCTCGT 186
CGACGATTTCCGAATGGGGCA 31
ATACGTTCCCGGGCCTTGTAC 181
GGTCGGAGGTTCAAGTCCTCC 30
CTGGAGGACCGAACCCATATC 406
CGATAAAGGGAGTGAGAGACT 659
TAAAAGGTACTCCGGGGATAA 64
CCCGAAGGCGCTGTGCTAACC 225
TTGATTTGTGAGTAGTTGGGG 11
GGCAAATGATCGGCCCGCGTT 2423
GGAGGAACACCAGTGGCGAAC 28
CGAACCAGTACCGTGAGGGAA 144
GCGTTGTTCGGATTTACTGGG 348
GTGCTAACGTCCGTCGTGGAG 32
AATGACGGTAACCGGAGAAGA 34
AGAGGATGATCCGCCACACTG 16
GGCCGTAAAACGCTCCAGCGC 12
GTTTGGCACCTCGATGTCGAC 145
ACACGCTTGATAAGCGTGGGG 16
TGCTGACATGAGTAACGATAA 12
CATGAGCAGGTTGAAGGTACG 393
AGGCTCACCAAGGCGACGATC 76
AGATATTCGGAGGAACACCCG 11
GTACGGCGCGTGAGCGAGAAC 10
GAACTCGGCCGTGAAACGCCC 11
GACTGTTTACCAAAAACACAG 15
GAAGGCCTTAGGGTTGTAAAG 16
TCGTGGGCCTGCAGGTGGTGA 535
GAATATTGACAGGATCTGTCC 150
TCGAAGCAACGCGCAGAACCT 27
GGGGTAAAGGCTCACCAAGGC 31
GAAGGACGTGATACGCTGCGA 53
CTTTGCAGACCTGGCAGCGAC 33
AGGATGTGAGGATCCCAAAAC 24
CCGATAGCGAACCAGTACCGT 157
TCGGCCGTAAAACGCTCCAGC 14
AGGGGGTAGCGACTGTTTACC 1741
ACTCTAAGGGGACTGCCGGTG 195
AAATGCACGCGTAACTTCGGA 89
CTGCCGGTGATAAGCCGAGAG 63
ACTGATCTAGAAGCCCGGCAC 54
CAGCCCTTGACATCCCGGTCG 84
ACGGTAACCGGAGAAGAAGCC 1143
GCCCCTAAGGCGAGGCCGAAA 13
TTAGGCTGGACCGGAGACAGG 545
ATCCCGGGGCTGGAGCAGGTC 10
TGAGAGACTCCCTCGCCGAAC 11
CGGGGTTGTTTGGCACCTCGA 191
TGTCGGCTCATCGCATCCTGG 15
AGAACTGAAACATCTAAGTAC 19
TGTTTAGCAAAAACACAGGGC 11
GGTAAGTTCCGACCTGCACGA 12
AGAAGTCCTTGAGTAGGGCGG 746
ACCGAAGTGGGTGATAGTCCC 331

TGTAGAGGTGAAATTCGTAGA 116
GGGACTGCCGGTGATAAGCCG 3083
GGGCAGTTTGACTGGGGCGGT 14
CGCGTGAGTGATGAAGGCCCT 17
CTAACTGCGGCCCGTTATCCG 11
TCGAGCGCCCCGCAAGGGGAG 75
GCGGCGGCGCCGGCAGCGGCA 31
TCAAGCCATACACCGAAGCTG 276
ATCGGGATGACTTGTGGCTAG 893
TACCTGAAACCGGATGCCTAC 5192
CTGTTTAGCAAAAACACAGGG 13
AGACGACGTATAGGGCCTGAC 22
GCAGAGACGAAAGTCGGTCAT 103
ACGATTTCCGAATGGGGCAAC 19
AAGGCAATATGGAAGTAGGGC 25
GAAAGCGTGGGGAGCAAACAG 12
AGTGAAATAGTACCTGAAACC 25
GCACTCTAAGGGGACTGCCGG 277
GGTACGCCGGGGATAACAGGC 92
TACCCTGGTAGTCCACGCCGT 293
CTTGAGAGTTTGATCCTGGCC 12
ACGTCCGTCGTGGAGAGGGCA 51
GGAAGTCTTGAGTATGGTAGA 45
AACTCGGCCGTGAAACGCTCG 13
ATATTGGACAATGGGCGCAAG 572
GTGAACCAGTACCGTGAGGGA 30
ATAGGTGGTAGACTTTGAAGC 66
ATGAATGTTAGCCGTCGGGGT 6609
CCAACGCAGACTCAGTGAAAT 110
GTGAGACAGTTCGGTCCCTAT 142
ACTGTTTGTTGATTTGTGAGT 28
ACGGACGGGATAACCGCTGAA 2428
GCATGCCGCGGTGAATACGTT 209
CTCGGCCGTGAAACGCTCCAG 32888
GCTAAGTGGGAAAGGATGTGA 156
GTGAAGTCGGAACAAGGTAGC 20
GAAAGTCGGTCATAGTGATCC 119
GGTTTAGAACGTCGTGAGACA 14
AGCGAACCTGGGGAACTGAAT 31
GATTAGATACCCTGGTAGTCC 75
TAGGGTCTGACGCCTGCCCGG 1175
TGGTGACGGATCGCGTGTGTT 19
GATGCCTTGGCATGCACAGGC 33
GAGCGCCCCGCAAGGGGAGCG 72
AAGGCGGCTCACTGGACCATT 375
GGGAGAGTAGGTCGCTGCCAG 4335
TTTGGACAATGGGCGCAAGCC 59
CTGCAGGTGGTGACGGATCGC 107
AGTCCATATCGACGGGGTTGT 63
GAAGGCAATATGGAAGTAGGG 21
TTGCGTCGGTATCTGGGCTTG 47
TAGTCATCATAAATAAGGTAT 37
ACAGGATCTGTCCCTAGTACC 21
GGCGAGCGAACGCGGACCAGG 49

AGCGCACGTAGGCGGACTTTT 62
ATGAATGTTAGCCGTCGGGGC 38
GAACGCTGGCGGCAGGCTTAA 1800
GCTCACTGGTCTAAATAAGGG 20
TCAGTTCGATGCATTAGACCC 154
CGGGCTGGGTTACACACGTGC 10
AGCATTCAGTTGGGCACTCTA 13
CTGCATATAGACCGTACCCTA 241
GTGGAGCAGCCCGGTAGCTCG 172
GCACGTATGTGCGTGGTAGCG 57
CTGATGACCCCCAAGAGTCCA 265
TGACGTCAAGTCCTCATGGCC 48
CAGCGACTGGGGTGAAGTCGT 12
TTGAGTAGGGCGGGACACGTG 36
CCAGCGCCAATGGTACTTCGT 52
TCGGTCATAGTGATCCGGTGG 621
CTGTAGCTCAGCTGGGAGAGC 85
CTTGAGAGTTTGATCCTGGCT 2201
ACATCTAAGTACCCAGAGGAC 73
GGACAATGGGCGAAAGCCTGA 11
CCAATCAAACTTGGAGATAGC 15
GTTTGACTGGGGCGGTCGCCT 212
TAAGAGGAGAGGTGCAAGCCT 130
GGCTACACACGTGCTACAATG 122
CGCATCCTGGGGCTGGAGCAG 456
TTCAGTTGGGCACTCTAAGGT 26
CGAGCTGGGCTACACACGTGC 12
CTCGGCCGTGAAACGCTCCAA 13
AGGGCCATCGTTCAACGGATA 47
CTAGTAATCGCGGATCAGCAT 142
GGCCCGCACAAGCGGTGGAGC 151
AGCGAACCTGGGGAACTGAAC 99
GGGTGTGGAAGTGCGGCAACG 303
AGCGAGTCTGAACAGGGCGTT 49
GGGCTGGGCTACACACGTGCC 139
TGCTAACGTCCGTCGTGGAGA 26
TCAGCCTGACGATCGCTTGCA 10
TCGGTATCTGGGCTTGTAGCT 101
ACCCACCTTAGATGACTAGAA 51
GCGAGCGAACGCGGACCAGGC 1761
AGTAACGGAGGCGCGCGATGG 90
CTTTGCAAGCAGGGGGTCGTC 98
GAAACATCTAAGTACCCAGAG 50
ACTCTCCCGCGTCTTGAGACG 34
CCAAGGCGACGATCCATAGCT 263
ATGCCGCGGTGAATACGTTCC 238
CAACCCTCGCCCTTAGTTGCC 13
AGTACCTGAAACCGGATGCCG 23
TAGATATTCGGAGGAACACCG 110
AAGAGGAGAGGTGCAAGCCTT 126
ACCCCGGAACTGCCTTTGATA 147
GACGAAAGTCGGCCATAGTGA 10
GTAGCTATATACGGACGGGAT 50
GGCCGTGAAACGCTCCACCGC 11

CACGTAGAATAGCAGAAGTCC 603
GCGCGATGGTAGGCTCAGAAC 1322
ACGCTGCGATAAGCGTCGGGC 18
GAGGAAAGGACATCAAACGAG 21
CGAGCAAGCTTAAGCCGGTAG 116
CGCCACACTGGGACTGAGACA 19
TTTGACTGATCTAGAAGCCCG 22
CCCGGTGCTGGAAGGTTAAGA 21
ATCGAGAATTGGAAAGAGGCC 186
CATGGGTCGACCACGATCCAA 987
GCCTAAGTACTCGTGCATGAC 269
GGTTATGGCGGAGCGGCTGCA 182
GGGCTGGGCTACACACATGCT 11
TATTGACAGGATCTGTCCCTA 248
CATGAAGCTTACCGGTACTAA 88
CAAGTGATCTAGCCATGAGCA 92
CGAGAAGCTGGTCTTTCTGCT 305
TAGATATTCGGAGGAACACCA 3737
TAGGAATATTGACAGGATCTG 793
TCCTGGAGGTATCGGAAGTGA 21
GGGGACTCACCGTCTTACTGA 421
GGGCTGGGCTACACACGTGCT 49173
GCCGCAGGTTCAAATCCTGCC 13
AATACGTTCCCGGGCCTTGTA 45
GAGAATTGGAAAGAGGCCGGA 71
TTGGACAATGGGCGAAAGCCT 24
CCGGGCCTTGTACACACCGCC 268
GCGGCAGGCTTAACACATGCA 116
ATTGGGACTGAGACACGGCCC 37
CGTAAAGCGCACGTAGGCGGA 329
GGACGTGATACGCTGCGATAA 28
TATTCGGAAGAACACCAGTGG 38
AACATTCCGCCTGGGGAGTAC 268
CCAGCCACACTGGGACTGAGA 13
TCGTCGAGTGCAATGGCATAA 35
GGATAAAAGGTACTCCGGGGA 46
GCGGACCAGGCCAGTGGCTTG 10
GCCAATGGTACTTCGTCTCAA 32
AAGGTACGCCGGGGATAACAG 120
GTGGCTAGGGGTGAAAGGCCC 20
ACAATAACGGTCCTAAGGTAG 19
CTCCAACGCAGACTCAGTGAA 83
AGTGGGGAATTTTGGACAATG 47
CAAGTCGAGCGCCCCGCAAGG 92
TCAACCCCGGAACTGCCTTTG 27
CTGGGCGTAAAGCGCACGTAG 210
TTGAGAGAACTGCGTTGAAGG 4827
AGGGGGTCGTCGGTTCGATCC 771
CGGAGGCGCGCGACGGTAGGC 10
GAATTGACGGGGGCCCGCACA 18
TGACAGTGGGCAGCGAGCACG 369
TAGGTCGTCGGCCCATGTGGG 21
AAAGGAATTGACGGGGGCCCG 31
TAAGTACTCGTGCATGACCGA 85

TGGGCACTCTAAGGTGACTGC 25
GGCGACCACGGTAGGGTCAGC 2032
ACAACCAGGATGTTGGCTTAG 23
ACCGCCATCACCGATTGTATC 16
AGGCAAAGAACAGGCGCAGCC 10
GAGGAACACCAGTGGCGAAGG 1127
CGAAATTCCTTGTCGGGTAAC 60
AATACCCTTTGATCCGACGAT 850
GCACCTCGATGTCGGCTCATC 26
TGCAGCTTACCGGTACTAATA 24
CGGTAGCTCGTCAGGCTCATA 72
CGGAGGAACACCAGTGGCGAC 58
TGCTTTGCAAGCAGGGGGTCG 90
AGTGAAACAGTACCTGAAACC 27
CCGAAGCTGTGGATGCACGTA 68
CAGTGAAATTGAATTCCCCGT 35
ATATTCGGAAGAACACCAGTG 38
ATGTCGACTCATCGCATCCTG 178
AGTACTCGTGCATGACCGATA 24
CGGGGTAGAGCAGCCCGGTAG 10
TACGAGAGGACCGGGATGGAC 382
AATGATCGGCCCGCGTTGGAC 16
TCGATGGGAACCACGTTAATA 25
TGGCTCAGAACGAACGCTGGC 3307
CTGGAAGTCTTGAGTATGGTA 32
TGACTGGGGCGGTCGCCTCCT 128
CATATAGACCGTACCCTAAAC 276
CGAACTCGGCCGTGAAACGCA 22
TGACGACATGTGTAGGATAGA 26
GTGGGGTCGGAGGTTCAAGTC 3002
TGTCGTCAGCTCGTGTCGTGA 82
AGGCCTTAGGGTTGTAAAGCT 19
TCAAATCCTGCCCCCGCAACC 35
TGATTTGTGAGTAGTTGGGGG 13
CTCTGGTGGACCTGTTGTGGC 511
GGTCCCTATCTGCCGTGGGTG 396
AAGCCTGATCCAGCCATGCCG 173
CAGCCTGACGATCGCTTGCAG 10
AGCCCGGTAGCTCGTCAGGCT 458
ACACGGATTTGACCTTCGGGT 29
CAGCTCACTGGTCTAAATAAG 17
GATGTGAGGATCCCAAAACAA 21
ATCTTGCAGCGAAGCGGTTCC 99
ACTCAAATGAATTGACGGGGG 16
GTGCAATGGCATAAGCCTGCC 34
CAACGGATAAAAGGTACGCCG 41
GGAAGGGCCATCGTTCAACGG 20
GACTGAGACACGGCCCAGACT 176
CCGATAGTGAACCAGTACCGT 27
AGCAGTGGGGAATATTGGACA 10
GGTAACCGGAGAAGAAGCCCC 886
CGCAAGGGGAGCGGCAGACGG 44
TCGGGGAGGTGCGAATACCCT 30
AATGATCGGCCCGCGTTGGAT 522

AAACGATGAATGTTAGCCGTC 1889
CGACTGTTTACCAAAAACACA 15
ACAGTTCGGTCCCTATCTGCC 1321
GGTAGACTTTGAAGCAGGGGC 296
AACGGTCCTAAGGTAGCGAAC 12
GGATGTTGGCTTGGAAGCAGC 11
GAAGGCCCTAGGGTTGTAAAG 3462
GAATGCTGACATGAGTAACGA 62
CGATAGTGAACCAGTACCGTG 35
TGTTGTGGCGCCAGCCGCATA 1692
AGTAATCGCGGATCAGAATGC 11
CAGTGGGCAGCGAGCACGCGA 393
GTCCTAAGGTAGCGAAATTCC 76
TTTGCAGACCTGGCAGCGACC 61
GTTCGATCCCGTCCGGCTCCA 1046
AGGCGATGAAGGACGTGATAC 91
GCGTCTCCGTGTTTTACATGG 20
TCAAGTCCTCCCAGGCCCACC 185
CGGTCGGAAATCGTTCGTCGA 271
TTCGTAGATATTCGGAAGAAC 46
TGACGACATGTGTAGGATAGG 173
GGTTGTAAAGCTCTTTCACCG 204
ACTCTGCAACTCGAGTGCATG 142
TGGGAAAGGATGTGAGGATCC 98
GGTCGACCACGATCCAAGCCT 189
GTGACAGTGGGCAGCGAGCAC 483
ATTCAGTTGGGCACTCTAAGG 83
CCTGGGGAACTGAAACATCCA 12
GACACGGACCAGACTCCTACG 12
GCCTTTGATACTGGAAGTCTT 15
ATCGCATCCTGGGGCTGGAGA 13
TTCGTAGATATTCGGAGGAAC 1935
CGGTGGTCCCGCGTGGAAGGG 39
ACGGGCTGGGCTACACACGTA 19
CGCGGACCAGGCCAGTGGCTA 14
TCTAACTGCGGCCCGTTATCC 16
AAACACGGAGACGCAAACTTC 15
CGATTTCCGAATGGGGCAACC 19
GCTAGGGGTGAAAGGCCAATC 27
ACCCACCTGAAAACGAGTATT 10
TAGACTTTGAAGCAGGGGCGC 1056
AGCCGCGGTAATACGAAGGGG 69
CCGACGATTTCCGAATGGGGA 22
CACTGGACCATTACTGACGCC 14
ATATGGAAGTAGGGCAATAAG 43
AGGAAAGGACATCAAACGAGA 23
AAGCGTGGGGTCGGAGGTTCA 1234
AAGGCGACGATCCATAGCTGG 40
GAGTGCATGAAGTTGGAATCG 84
CTGTCTGAACATGGGTCGACC 2001
GCCCTAGGGTTGTAAAGCTCA 16
ACTTCGTCTCAAGACGCGGGA 26
GACACAGGTGCTGCATGGCTG 21
ACTGCAAGACTGACAAGTCGA 47

CACGAATGGCGTAACGACTTC 14
AATAACTCAGGGAAACTTGTG 78
GGGACACGTGAAATCCTGTCT 22
TTGCAATAGATCGGGATGACT 86
TCCATATCGACGGGGTTGTTT 85
AACAGGATTAGATACCCTGGT 12
GATGACTTGTGGCTAGGGGGG 184
CAGTTAGGCTGGACCGGAGAC 36
CTGAGCAGGGTTAGCTGGCCC 13
TGAGACACGGCCCAGACTCCT 266
AGCAGGTCCCAAGGGTATGGC 20
ACGTTGATAGGCCGGGTGTGG 51
GACGACATGTGTAGGATAGGT 515
AAGGTAGCGAAATTCCTTGTC 216
GTTCGCCATTTAAAGCGGTAC 34
TGATCTAGCCATGAGCAGGTT 74
CGCGGGAGAGTAGGTCGCTGA 10
ATACGCTGCGATAAGCGTCGG 39
GTACTAATAGCTCGATCGACG 13
CAGGGGGTCGTCGGTTCGATC 832
GCCCTAGGGTTGTAAAGCTCG 14
GTAGTTTGACTGGGGCGGTCT 53
CCGAATACCGGGGAGTACTAG 31
ACGGGCTGGGCTACACACGTG 40025
TGCAAGACTGACAAGTCGAGC 20
CAGGTCCCAAGGGTATGGCTG 2497
CGCGGACCAGGCCAGTGGCTG 10
GGAGGCGCGCTATGGTAGGCT 24
GGCCGTGAAACGTTCCAGCGC 14
GTAGGTCGCTGCCAGGTCTGC 1479
GGCCCAGACTCCTACGGGAGG 2072
GCTATATACGGACGGGATAAC 197
CGACGAGGGGAGTGAAACAGT 17
AGGACATCAAACGAGACTCCG 337
CTGGGGCGGTCGCCTCCTAAA 31
CACTGGACCATTACTGACGCT 5441
GGTGGATGCCTTGGCATGCAC 28
AGTGGGCAGCGAGCACGCGAG 298
TTGATACTGGAAGTCTTGAGT 34
AGGATGATCCGCCACACTGGG 13
CGGCCCATGTGGGCCGCCCCC 20
ATCGCGTGTGTTGTGAGGTCT 267
GTTGAAGGCAATATGGAAGTA 13
AACAATAACGGTCCTAAGGTA 19
GGTCAGCGACTTAGTGTATCG 188
GAAGAAGCGTGACCTCACTAT 53
ACAGGCTGATGACCCCCAAGA 5244
TCCCCGTGAAGATGCGGGGTT 26
ACTGAGACACGGACCAGACTC 20
GGAATTGACGGGGGCCCGCAC 13
ACTCGTGCATGACCGATAGCG 294
TGATCAGCCACACTGGGACTA 10
TAAGTTCCGACCTGCACGAAT 16
GTGAAACAGTACCTGAAACCG 29

AACGGATAAAAGGTACTCCGG 34
GTGGTGACGGATCGCGTGTGT 13
CATTACTGACGCTGGGGTGCG 14
GGGCCCGCACAAGCGGTGGAC 13
TGACGGTAACCGGAGAAGAAG 94
GGTAGGTGTAGGCGCAGCGAA 67
CGTGGGTGTAGGAATATTGAC 57
AGATATTCGGAAGAACACCAG 24
AGAGGAGAGGTGCAAGCCTTG 162
CATGAGTAACGATAAAGGGAG 25
AAGCGGTACGTGAGTTGGGTT 12
AACGGATAAAAGGTACCCCGG 69
TGACCACCATCTAAGGTCCCT 29
TGGGGAGTTTGACTGGGGCGG 248
TCGATCGACTTGATCACTCCC 412
AGGGCGGGACACGTGAAATCC 20
TTCGTGCCAGCAGCCGCGGTA 22
AGTTGGGCACTCTAAGGGGAA 27
AAACAACCAGGATGTTGGCTT 51
GCAGCCGCGGTAATACGAAGG 53
TTTGCTACGGAATAACTCAGG 166
GCCACATTGGGACTGAGACAC 19
CCTGGGGAGTACGGTCGCAAG 844
ATGCACAGGCGATGAAGGACG 299
AAGCCGGTAGGTGTAGGCGCA 148
GAGAGAACTGCGTTGAAGGAC 10
AACTCAAAGGAATTGACGGGG 193
CACACCAACTTCGATCCGAAA 14
CACGTAGGCGGACTTTTAAGT 64
CCGTACACGTAGAATAGCAGA 393
GAAGATCGAGAATTGGAAAGA 1041
AGCCTAAGTACTCGTGCATGA 141
ATAGCTGGTCTGAGAGGATGA 76
GTGAAGATGCGGGGTTCCTGC 49
AAATTCGTAGATATTCGGAAG 25
CAGCCACACTGGGACTGAGAC 316
CGGATTTGACCTTCGGGTTTG 24
AAGACGACGTATAGGGTCTGA 56
CGACCTGCACGAATGGCGTAA 107
TGTTTTGTTGGAGCAACGCTG 19
GTAGGATAGGTGGTAGACTTT 51
GGACTGGTAGAGAATACCAAG 133
GAACGCGGACCAGGCCAGTGG 2527
TCGGGGTGTTTACACTTCGGT 55
AGTATTCCCTATCAGAGCCGT 18
TGATCAGCCACACTGGGACTG 3211
CGCGCGATGGTAGGCTCAGAA 1314
ACACTGGCATTCGTGACGACA 222
GCCAGTCAGCCTGACGATCGC 46
GGGCGTTCAGTTCGATGCATG 11
GGAACCTGCGGCTGGATCACC 28
TGGCTGTCGTCAGCTCGTGTC 263
ATTGGACAATGGGCGCAAGCC 1578
TATTCGTGGGCCTGCAGGTGG 171

ATATACGGACGGGATAACCGC 185
AAGACGACGTATAGGGCCTGA 17
AGAACTGCGTTGAAGGAACTC 1806
CCATCGCTCAACGGATAAAAG 88
GATATTCGGAAGAACACCAGT 28
AAGGCCCTAGGGTTGTAAAGC 5745
TTGGGTTAAGTCCCGCAACGA 20
GCTAACGTCCGTCGTGGAGAG 17
CTATAGCTTTACACTGGCATT 13
GAAGTCGTAACAAGGTAGCCG 12
GGGTCAGCGACTGGGGTGAAG 350
TCGGCAAATTGCACGCGTAAC 10
AGCACCTGCTTTGCAAGCAGG 52
GGCGGGTAGTTTGACTGGGGC 18
TTCGGAAGAAGCGTGACCTCA 110
ACGAGCGCAACCCTCGCCCTT 41
TACCTTTTGTATAATGGGTCA 14
GGTTCCTGCGGTTAGACGGAA 258
GCCATACACCGAAGCTGTGGA 32
CCGACCTGGTGGTTATGGCGG 14
TGGGGCAACCCACCTTAGATG 31
ACTGGAGAGTTTGATCCTGGC 12
CAGTACCTGAAACCGGATGCC 6999
CGAATACCCTTTGATCCGACG 216
AGACAGTTCGGTCCCTATCTG 188
TGTGAGGATCCCAAAACAACC 27
GGGCTCAAGCCATACACCGAA 728
GAACGTCGTGAGACAGTTCGG 289
GTGAGACATCCTGGAGGTATC 145
CGTGTCGTGAGATGTTGGGTT 150
CTGGACCATTACTGACGCTAA 10
GGCGCTTGAGAGAACTGCGTT 41
GCTCAGAACGAACGCTGGCGG 2695
ACTGGGGCGGTCGCCTCCTAA 37
CGGGCCTTGTACACACCGCCC 23
GGAAGAGATTTTGGACGGTTT 10
ACTCGGCCGTGAAACGCTCCG 14
AGACCAGGGGGTAGCGACTGT 72
CAGCTCGTGTCGTGAGATGTC 10
GAGACGAAAGTCGGCCATAGT 10
GGCTGATGACCCCCAAGAGTC 4266
GTGAAATTCGTAGATATTCGG 285
GCGGGAGAGTAGGTCGCTGCC 10048
GAGATGTTGGGTTAAGTCCCG 43
GCTGAGTTTTGATGGATATTG 60
CTGGTGGACTGGTAGAGAATA 2462
AGCGAAAGCGAGTCTGAACAG 15
TTTTACGAAAGTCTGCCTGTT 17
GCTAGTTGGTGGGGTAAAGGC 41
AAGAAAGCGTAACAGCTCACT 33
ACATGGGTCGACCACGATCCA 1291
GCTGGTCTTTCTGCTGATACT 15
CGGATTGCACTCTGCAACTCG 342
GTGGGGAATTTTGGACAATGG 47

TCAGCTGGGAGAGCACCTGCT 545
CCAAACTCCGAATACCGAGGA 18
AGGAACTCGGCAAATTGCACG 21
AGACACGGACCAGACTCCTAC 10
ACATAGATCGCAGGCCAGTCA 333
ACGCTGAGGTGCGAAAGCGTG 71
ACTCGGCCGTGAAACGCTCCA 30375
GGGTTCCTGCGGTTAGACGGA 447
GCGGAAGAGATTTTGGACGGT 19
CAGCTCGTGTCGTGAGATGTT 234
GGGTAAAGGCTCACCAAGGCG 31
CTCAACGGATAAAAGGTACGC 83
CCCGTGAAGATGCGGGGTTCC 37
ACTTGTGGCTAGGGGTGAAAC 13
GGCCACACTGGGACTGAGACA 24
AGTCATCATAAATAAGGTATG 10
TGGACCATTACTGACGCTGAC 16
ACTCGGCCGTGAAACGTTCCA 13
GATTGATCTTGCAGCGAAGCG 84
TTGCTCACGGGCCGTACCGCA 16
GAGAACTGAAACATCTAAGTA 15
CACATTGGGACTGAGACACGG 41
AACACGTACTGGAGGACCGAA 330
GCAGGTGGTGACGGATCGCGT 70
GACCGATAGTGAACCAGTACC 12
CAACGCGCAGAACCTTACCAG 56
GCAAACCTGGGGAACTGAAAC 16
GGGCCATAGCTCAGCTGGGAG 1085
TACGTTCCCGGGCCTTGTACA 98
CCTCATGGCCCTTACGGGCTG 112
GTATGGTGGGTAGTTTGACTG 21
AGGAACACCAGTGGCGAAGGC 893
GACGGAAAGACCCCGTGCACC 364
CGGCCGTGAAACGCTCCACCG 11
ATGATCAGCCACACTGGGACT 5023
ACATCAAACGAGACTCCGCTA 116
GTCGCAAGATTAAAACTCAAA 144
AGCTGGGTTCAGAACGTCGTG 13
TCCCTATCAGAGCCGTGGAAG 54
CCTTGGCATGCACAGGCGATG 166
CCAAGGGTTTGGCTGTTCGCC 12
CAAGTCCTCATGGCCCTTACG 110
TCTGAGCAGGGGTAGCCGGCC 10
AAAGAAAGCGTAACAGCTCAC 10
GGATAAAAGGTACGCCGGGGA 1484
CCCTAAGGCGAGGCCGAAAGG 15
GTAGCCGTAGGGGAACCTGCG 29
CGGAGGAACACCAGTGGCGAA 2607
CTATCCTTCAGTTAGGCTGGA 12
CGAAATTCCTTGTCGGGTAAA 46
ATGTGTAGGATAGGTGGTAGG 12
TGGTAGAGGTGAGTGGAATTC 34
TCAAACTTGGAGATAGCTGGT 168
AGATCGAGAATTGGAAAGAGA 14

GGGCCGTGAAACGCTCCAGCG 13
GATCCTAACCAAACTCCGAAT 82
TGGCTCAGAACGAACGCTGGA 10
TCGACCGAAGTGGGTGATAGT 255
AAAGCTCTTTCACCGGTGAAG 678
TTCAGTTCGATGCATTAGACC 193
AGCAGCCGCGGTAATACGAAG 10
TCCGAATACCGGGGAGTACTA 26
CCGAGTGTAGAGGTGAAATTC 400
GCGTGACCTCACTATGGGCAA 16
ACTGGGCGTAAAGCGCACGTA 230
GACAGGATCTGTCCCTAGTAC 83
AGGCAGCAGTGGGGAATTTTG 23
ACCGATAGTGAACCAGTACCG 15
GAAGGAACTCGGCAAAATGCA 263
GACCGAATACCCCCGGGGGTA 1040
CCTGAAACCGGATGCCTACAG 11
CGAACTCGGCCGTGAAACGCC 48
ATGAAGTCGGAATCGCTAGTA 202
TCCTAAGGTAGCGAAATTCCT 81
CCATAGCTGGTCTGAGAGGAT 77
TACCCTGGTAGTCCACGCTGT 31
CAGTTCGGTCCCTATCTGCCG 993
AGACGAAAGTCGGTCATAGTG 137
GTTGTTTGGCACCTCGATGTC 175
AGATCGAGAATTGGAAAGAGG 586
GGCGCCAGCCTTTGTGGAGTC 14
ACCTGCTTTGCAAGCAGGGGG 88
GAGTAGGGCGGGACACGTGAA 27
CCGGATGCCTACAAACAGTTG 36
GCATGAAGTCGGAATCGCTAG 32
GTAGGCGGACTTTTAAGTCAG 19
CACCGCCCGTCACACCATGGG 36
ATGGCATAAGCCTGCCTGACT 177
GAGTGAGAGACTCCCTCGCCG 21
GGTGAAGTCGTAACAAGGTAG 50
TGCGGGGGCAGGATTTGAACC 18
CGGAGGAACACCAGTGGCGAG 85
TTCCGAATGGGGCAACCCACC 12
GGGGTGAAAGGCCAATCAAAC 13
GACAATGGGCGCAAGCCTGAT 254
CGAAATTCCTTGTCGGGTAAG 3305
GGTCAGCGACTGGGGTGAAGT 344
ACATCCCGGTCGCGGTTAGTG 57
GCCCAGACTCCTACGGGAGGC 1325
ATGTGTAGGATAGGTGGTAGA 1124
TCTAGCCATGAGCAGGTTGAA 23
AACGGTCCTAAGGTAGCGAAA 43
GACAGTGGGCAGCGAGCACGC 365
CCGTAGGGGAACCTGCGGCTG 29
CTTGAGTAGGGCGGGACACGT 32
GAATATTGGACAATGGGCGAA 21
CAAGATTAAAACTCAAAGGAA 81
GCTCGTGTCGTGAGATGTTGG 252

CTCTTGGGGGTCATCAGCCTG 20
TATTTGGTTGCGGGGGCAGGA 11
ACCGAAGCTGTGGATGCACGT 129
ATCGCATCCTGGGGCTGGAGC 283
TTGGTTGCGGGGGCAGGATTT 30
GTGACCTCACTATGGGCAACC 15
ATAGGGGTTAGAAGCGAACCT 83
CCTGAAACCGGATGCCTACAA 532
TGACGACATGTGTAGGATAGT 20
CGATGAAGGACGTGATACGCT 53
CCATTCCGAACTCGGCCGTGA 48
CGAACTCGGCCGTGAAACGCT 7282
GGACGTATCTCTGGTGGACCT 288
CACTGGACCATTACTGACGCA 11
CTGCGGTTAGACGGAAAGACC 55
GGGTTGTAAAGCTCTTTCACC 1582
GCATGGTTGTCGTCAGCTCGT 28
CCGACGATTTCCGAATGGGGC 168
CGGCCGTAAAACGCTCCAGCG 12
AATGGCATAAGCCTGCCTGAC 57
GAAGCCCCAGTAAACGGCGGC 10
CAAGCTTAAGCCGGTAGGTGT 15
ACGGGCTGGGCTACACACGTC 30
TTTGGTGGATGCCTTGGCATG 42
GAGAGTGATCAAGTGTCTTAA 216
TGATAGGCCGGGTGTGGAAGT 26
GCCCTAGGGTTGTAAAGCTCC 32
TGCATGACCGATAGCGAACCA 47
AAACACAGGGCTCTGCGAAGT 208
GTAGAATAGCAGAAGTCCTTG 1756
TTCCGAACTCGGCCGTGAAAC 1238
GCCGTGAAACGTTCCAGCGCC 12
GTACTAATAGCTCGATCGACC 26
GGCCCTTACGGGCTGGGCTAC 17
ATGTAATCGGATCAACTGAAG 10
CGCAGACGAGGCGCTGACACG 11
GTATTCCCTATCAGAGCCGTG 21
GGAGAGTAGGTCGCTGCCAGG 4230
AGAGTCCATATCGACGGGGTT 54
GGTGAGTGGAATTCCGAGTGT 53
TTTGTATAATGGGTCAGCGAC 128
GGGCTGCGCCTGTTCTTTGCC 56
CGTGAAGAGAAGATGTAATCG 22
GAAAGTCCAAGGGTTCCTGCT 13
ACACAGGGCTCTGCGAAGTCG 1109
GTGAAGTCGTAACAAGGTAGC 60
ACCAGGGGGTAGCGACTGTTT 1523
CTCGGCCGTGAAACGCTCGAG 12
CGTTGAAGGAACTCGGCAAAG 12
AACTATAACGGTCCTAAGGTA 15
AGCCATGAGCAGGTTGAAGGT 49
GAACGAACGCTGGCGGCAGGC 2894
GCCCTAGGGTTGTAAAGCTCT 6112
GTAGTTTGACTGGGGCGGTCG 254

TGGCGGACACGTTTCTTGGTA 119
GTTAATATTCGTGGGCCTGCA 101
CGCGGGAGAGTAGGTCGCTGC 5701
GTACTAATAGCTCGATCGACT 4899
CGTGAGATGTTGGGTTAAGTC 184
TCGGATTTACTGGGCGTAAAG 2558
CTCAAAGGAATTGACGGGGAC 22
TAAGGGGACTGCCGGTGATAA 182
CACTGGACCATTACTGACGCG 10
TTCCCGGGCCTTGTACACACC 229
TCCGCTAGTAGTGGCGAGCGA 56
CGGAACTGCCTTTGATACTGG 171
GGTAGCTCGTCAGGCTCATAA 72
CGCGGACCAGGCCAGTGGCTT 4371
CTTGAGAGAACTGCGTTGAAG 4295
TCAGTTGGGCACTCTAAGGTG 25
GGGCATTTGGTGGATGCCTTG 43
ACTCAGTGAAATTGAATTCCC 60
GAGGGCAACAACCCTGACCAC 146
GACGTGATACGCTGCGATAAG 21
GGGCTGGGCTACACACGTACT 15
TAACCGGAGAAGAAGCCCCGG 611
GTATGGTAGAGGTGAGTGGAA 23
AGCGCCCCGCAAGGGGAGCGG 76
CGTTGAAGGAACTCGGCAAAA 62
ACCGCCCGTCACACCATGGGA 48
CTAACACATGCAAGTCGAGCG 15
TAAGCCTGCCTGACTGCAAGA 231
GGCTGTCGTCAGCTCGTGTCG 75
GATAAAGGGAGTGAGAGACTC 133
CCTGCGGCTGGATCACCTCCT 102
TCGTCCGTGAAACGCTCCAGC 10
TGGAGCCCAAGGTTTGTCCTG 108
TGGGCAGCGAGCACGCGAGTG 67
TGTATCGAGCAAGCTTAAGCC 27
AAGTGCGGCAACGCATGCAGC 10
ACAGGCTGATGACCCCCAAGC 10
AAGGCCGCAGGTTCAAATCCT 15
TGGCGAGCGAACGCGGACCAG 43
TTCACCGGTGAAGATAATGAC 23
ACGGTCGCAAGATTAAAACTC 252
ATACAGGTGCTGCATGGCTGT 16
CAGCGACTTAGTGTATCGAGC 168
TGGGCTGCGCCTGTTCTTTGC 56
AGACGCGGGAGAGTAGGTCGC 728
GCAAGCAGGGGGTCGTCGGTT 778
CGGGCTAGGCTACACACGTGC 16
AAGTTCCGACCTGCACGAATG 14
AGGTACGCCGGGGATAACAGG 96
AGTTGGGCACTCTAAGGGGAC 1479
TATCGAGCAAGCTTAAGCCGG 16
GGCCGTACCGCAGCTGACGCT 50
TGGGTCAGCGACTTAGTGTAT 195
CGGGCTGGGCTACACACGTAC 18

CCCAAGAGTCCATATCGACGG 11
GGATGTTTGGTTAGGCGGAAG 49
GTAGATATTCGGAGGAACAAC 16
CCCTATCAGAGCCGTGGAAGA 31
TAGACGGAAAGACCCCGTGCA 331
GAGAGAACTGCGTTGAAGGAA 3127
CAGTACCTGAAACCGGATGCG 10
CAGTTGGGCACTCTAAGGGGA 1545
CATGGCTGTCGTCAGCTCGTG 1193
GCATAAGCCTGCCTGACTGCA 274
TCGAGTGCATGAAGTTGGAAT 251
TTGACATCCCGGTCGCGGTTA 162
ACCAGTGGCGAAGGCGGCTCG 13
GTCGGAATCGCTAGTAATCGC 215
TAACCAAACTCCGAATACCGG 39
CTGTGGATGCACGTATGTGCG 348
GGGTTAGCCGGCCCCTAAGGC 260
TATGGTAGAGGTGAGTGGAAT 24
GTGAGGATCCCAAAACAACCA 24
CGTTAATATTCGTGGGCCTGC 96
TCGCGGTTAGTGGAGACACTA 41
CGATCCCGTCCGGCTCCACCA 62
CTGAGATACGGCCCAGACTCC 10
CCAGCAGCCGCGGTAATACGA 23
AGAATGCTGACATGAGTAACG 111
ACACGGCCCAGACTCCTACGG 4888
TGAAACCGGATGCCTACAAAC 121
GGCAGGCGACCACGGTAGGGT 245
GTCGGTATCTGGGCTTGTAGC 83
CTGTTGCAATAGATCGGGATG 24
AGATACGGCCCAGACTCCTAC 12
GGACCAGGCCAGTGGCTTTTG 561
CAGTTCGATGCATTAGACCCG 105
GGGCCCGCACAAGCGGTGGAG 151
GCACTCTGCAACTCGAGTGCA 166
AAGTGGGAAAGGATGTGAGGA 102
ACGGAGGCGCGCGATGGTGGG 14
GTCTGAACAGGGCGTTCAGTT 10
GGGCGTTCAGTTCGATGCATT 378
CATAGGGGTTAGAAGCGAACC 115
CGCGCGATGGTAGGCTCAGAC 17
ATTCGTAGATATTCGGAAGAA 45
AGTGAGAGACTCCCTCGCCGA 29
CCGGTGCTGGAAGGTTAAGAG 20
GAGTGGAATTCCGAGTGTAGA 407
AGTCCCGCAACGAGCGCAACC 81
CCGCGGTAATACGAAGGGGGC 426
ACCAGTGGCGAAGGCGGCTCA 406
AAAGCGTAACAGCTCACTGGT 216
TGCCAGCAGCCGCGGTAATAC 15
GATCCGCCACACTGGGACTGA 20
ACTGCGTTGAAGGAACTCGGC 1498
TAATGACGGTAACCGGAGAAG 23
CTCCTGCATATAGACCGTACC 604

AGGGTAGCTATATACGGACGG 19
GGCTCAGAACGGTCGGAAATC 355
GGTAACACGTACTGGAGGACC 344
TTGCAAGCAGGGGGTCGTCGG 87
AGCCGGTAGGTGTAGGCGCAG 163
TGGTGGTTATGGCGGAGCGGC 2974
GGGCGCAAGCCTGATCCAGCC 436
CCGTACCGCAGCTGACGCTGC 17
TTGGCCGTGAAACGCTCCAGC 16
CAGTACCTGAAACCGGATGCA 20
TTGATGAGGGGCCGTAGCTCA 150
GGGCAACAACCCTGACCACCA 27
CCTGGGGAGTACGGTCGCAAC 13
AGGGTCTGACGCCTGCCCGGT 1293
TGTCCTGGGTGACAGCGTACC 56
AGCACCCCGACGAGGGGAGTG 32
ATGGGTCGACCACGATCCAAG 975
TTTATGGATGTCTAACTGCGG 17
CAGTTGGGCACTCTAAGGTGA 26
CGGTAGGTGTAGGCGCAGCGA 107
TGAAGTTGGAATCGCTAGTAA 94
GGGCTACACACGTGCTACAAT 219
TGGACCATTACTGACGCTGAG 18425
AGTCATCATAAATAAGGTATC 20
ACTTGTGGCTAGGGGTGAAAG 1009
GTGGATGCCTTGGCATGCACA 24
ACTGGGCCGTGAAACGCTCCA 13
ACTGGTTGGATGTTTGGTTAG 33
CCAATAAGGTTAGCGGGCTTT 64
AGAACGAACGCTGGCGGCAGG 2308
GATGTCGACTCATCGCATCCT 102
CAGCGAGCACGCGAGTGTGAG 21
TGGGACTGAGACACGGCCCAG 122
AGCGACTGGGGTGAAGTCGTA 12
CACCACGTTGATAGGCCGGGT 156
ATTGATCTTGCAGCGAAGCGG 162
CGTGAGTGATGAAGGCCCTAG 19
AAGTCCAAGGGTTCCTGCTTA 18
CGATGGTAGGCTCAGAACGGT 251
GAAATCGTTCGTCGAGTGCAA 15
TAGGGGTGAAAGGCCAATCAA 12
CTGTGAAGGGACAGTCGTGAG 41
CTTGATAAGCGTGGGGTCGGA 102
GAGAGACTCCCTCGCCGAAAG 24
TCGATGTCGACTCATCGCATC 43
ACCATTACTGACGCTGAGGAG 10
GCCTGACTGCAAGACTGACAA 67
CTAAACCGACACTGGTGGACT 124
GGATTAGATACCCTGGTAGTC 63
CAACGAGCGCAACCCTCGCCC 100
GACGACGTATAGGGTCTGACG 205
ATCCCGGTCGCGGTTAGTGGA 116
AGAACGAACGCTGGCGGCAGA 16
GTTTTGTTGGAGCAACGCTGG 26

TGTTTGTTGATTTGTGAGTAG 27
CTGTTTGTTGATTTGTGAGTA 27
GGTAGCGACTGTTTACCAAAA 84
CCCAAGGGTTTGGCTGTTCGC 16
CTCACCAAGGCGACGATCCAT 350
GTTGGAGCAACGCTGGATGGG 33
GGGCTACACACGTGCTACAAC 25
CGGAGACAGGTGCTGCATGGC 270
CGGGGTGTTTACACTTCGGTG 29
TGGACCATTACTGACGCTGAA 63
GTAGGCTCAGAACGGTCGGAA 149
CGGGCTGTGCTACACACGTGC 10
CTTTGAAGCAGGGGCGCCAGC 552
GACGCCTGCCCGGTGCTGGAA 228
ACGAGTATTCCCTATCAGAGC 11
TGGACTGGTAGAGAATACCAA 202
ATGGGCAACCATAGGGGGGTG 34
TGGTGCATGGCTGTCGTCAGC 15
CACAGGGCTCTGCGAAGTCGC 1095
AGTTTGACTGGGGTGGTCGCC 22
ACGACGTATAGGGCCTGACGC 40
GTTAGAAGCGAACCTGGGGAA 2650
GTTAAGAGGAGAGGTGCAAGC 28
CAAAGGAATTGACGGGGGCCC 69
CAAATGATCGGCCCGCGTTGG 2032
CATAGCTGGTCTGAGAGGATG 70
GTAGGGGAACCTGCGGCTGGA 59
GTTCGTCGAGTGCAATGGCAT 37
GACTGGTAGAGAATACCAAGG 65
GCGATGAAGGACGTGATACGC 90
ATTAGATACCCTGGTAGTCCA 133
GACTGATCTAGAAGCCCGGCA 51
CTAAGTACTCGTGCATGACCG 246
CCGTAAACGATGAATGTTAGC 240
GCGGCTGCACCCGATCCCATT 15
CGCGGACCAGGCCAGTGGCCT 16
GCCCGCACAAGCGGTGGAGCA 33
TTCCGCCTGGGGAGTACGGTC 2593
TTACGGGCTGGGCTACACACG 1454
CGGGATGACTTGTGGCTAGGA 11
AAAGGCAAAGAACAGGCGCAG 42
AGGCGCGCGATGGTAGGATCA 16
CCAAAAGCCGTCTCAGTTCGG 22
CTCAAAGGAATTGACGGGGGC 206
TCTCCAACGCAGACTCAGTGA 71
GGCTGTAGCTCAGCTGGGAGA 249
TGCGTTGAAGGAACTCGGCAC 16
GAAGAGATTTTGGACGGTTTA 10
TACTGACGCTGAGGTGCGAAA 3228
ACGCGGACCAGGCCAGTGGCA 39
CAACCCTGACCACCATCTAAG 33
TACACACCAACTTCGATCCGA 14
CGGAGGCGCGCGATGGTGGGC 18
GGAGGCGCGCGATGGTAGGCT 36784

ACGGATAAAAGGTACCCCGGG 78
TTTGAACCTGCGGCCTTCAGG 111
GCGTGGGGTCGGAGGTTCAAG 5676
ATTTATCGGCAAATGATCGGC 36
TCCTGGCTCAGAACGAACGCT 3299
AAGGACGTGATACGCTGCGAT 45
GACTCAGTGAAATTGAATTCC 59
TTGTATAATGGGTCAGCGACT 194
GACTGTTTATCAAAAACACAG 10
ACGCGGGAGAGTAGGTCGCTG 5628
TGCGGCTGGATCACCTCCTTT 151
CAAGCCATACACCGAAGCTGT 54
GCTCAACGGATAAAAGGTACT 113
GTCCTGGGTGACAGCGTACCT 24
GGAGGCGCGCGATGGTGGGCT 20
GAAGATGCGGGGTTCCTGCGG 47
ACCAGCCACACTGGGACTGAG 10
TCGCATCCTGGGGCTGGAGCA 473
AAAATGCACGCGTAACTTCGG 59
GCGGTTAGACGGAAAGACCCC 63
AAGGTACGGTAACACGTACTG 24
GAGGTGCGAAAGCGTGGGGAG 70
CGTGGGGTCGGAGGTTCAAGT 3281
CTGACGCTGAGGTGCGAAAGC 314
GGAAATCGTTCGTCGAGTGCA 36
CGCTGCGATAAGCGTCGGGGC 19
AAATCGTTCGTCGAGTGCAAT 10
GTCCCGCGTGGAAGGGCCATC 10
TAACTATAACGGTCCTAAGGT 10
GGCGGACACGTTTCTTGGTAA 108
GATGACCCCCAAGAGTCCATA 28
TGATACGCTGCGATAAGCGTC 32
TGAGACACGGACCAGACTCCT 10
ACGCGGACCAGGCCAGTGGCG 22
TGCAAGTCGAGCGCCCCGCAA 71
GAAGGGCCATCGCTCAACGGA 241
GATCCGACGATTTCCGAATGG 187
GCATGGCTGTCGTCAGCTCGT 1215
GGACTTTTAAGTCAGGGGTGA 31
GAACTGAAACATCTAAGTACC 124
GTCGGTCATAGTGATCCGGTG 216
GGCTAGGGGTGAAAGGCCAAT 24
GTCGTGAGATGTTGGGTTAAG 134
CGGGATGACTTGTGGCTAGGG 16070
CCCGCAAGGGGAGCGGCAGAC 61
ACGATCCAAGCCTAAGTACTC 39
ATGAGTAACGATAAAGGGAGT 29
TCGCAAGACGACGTATAGGGT 24
GCTGTTCGCCATTTAAAGCGG 35
TGATCCAGCCATGCCGCGTGA 19
GCTCAACGGATAAAAGGTACC 16
GTTAGTAGTGGCGAGCGAACG 25
AATACCCCCGGGGGTAGAGCA 17
TAGTACGAGAGGACCGGGATG 153

TCCAAAAGCCGTCTCAGTTCG 21
CGTGGGGTCGGAGGTTCAAGC 11
TTTAAGTCAGGGGTGAAATCC 22
GTATCGGAAGTGAGAATGCTG 23
GGGTAGCGACTGTTTACCAAA 243
GGAGGCGCGCGATGGTAGGCC 70
GCGCCCCGCAAGGGGAGCGGC 90
ATGTACCGGGGCTCAAGCCAT 522
TCCTGGCTCAGAACGAACGCC 24
GCGTGGGGTCGGAGGTTCAAA 16
TTTACGAAAGTCTGCCTGTTC 12
CATGGTTGTCGTCAGCTCGTG 31
AGCAGGTTGAAGGTACGGTAA 313
GCGAAGCGGTTCCAGGAAATA 13
CTAAGGAAGATCGAGAATTGG 45
GGCTCAGAACGAACGCTGGCG 2629
CGAAAGTCGGCCATAGTGATC 10
AATAGCTCGATCGACTTGATC 13
AAGGGCCATCGCTCAACGGAT 861
GTAGTCGATGGGAACCACGTT 59
TTAGAAGCGAACCTGGGGAAC 8677
CCCCGGAACTGCCTTTGATAC 213
TTTAAGAGCTGAGTTTTGATG 11
TATAGGGTGTGACGCCTGCCC 15
GAGAACTGCGTTGAAGGAACT 2395
GGCTCACTGGACCATTACTGA 7627
CGATCCAAGCCTAAGTACTCG 15
ATCTAGAAGCCCGGCACCGCA 30
CCTGGAGGTATCGGAAGTGAG 22
AGAACACCAGTGGCGAAGGCG 13
AACGATAAAGGGAGTGAGAGA 191
GCTCACCAAGGCGACGATCCA 359
CAGCGAAGCGGTTCCAGGAAA 56
GCGGGGTAGAGCAGCCCGGTA 10
TAGTGGCGAGCGAACGCGGAC 101
CAGAACGAACGCTGGCGGCAC 11
GCCGTGAAACGCTCCAGAGCC 10
GCAGTGGGGAATTTTGGACAA 54
AAGAGCTGAGTTTTGATGGAT 286
AACGAGGGCGGCGGCGCCGGC 24
CCAGTGGCGAAGGCGGCTCAC 384
ACCTGAAACCGGATGCCTACA 3765
CCTGGGGAACTGAAACATCTC 14
TAGTTGGTGGGGTAAAGGCTC 40
TGGACAATGGGCGAAAGCCTG 17
GAGACATCCTGGAGGTATCGG 116
TGTTGGAGCAACGCTGGATGG 35
ACTATCCTTCAGTTAGGCTGG 12
GGCTATGGGGACTCACCGTCC 13
CCCGGCTAACTTCGTGCCAGC 50
TCATCGCATCCTGGGGCTGGA 283
GCGGAGCGGCTGCACCCGATG 25
GACATCCTGGAGGTATCGGAA 105
AGCGAACCAGTACCGTGAGGG 192

GAGAACTGCGTTGAAGGAACC 15
GGCTCACTGGACCATTACTGG 13
ACGACTTCCCCGCTGTCTCCA 229
TCACCAAGGCGACGATCCATA 348
GACGGGATAACCGCTGAAGGC 2273
GAAGTAGGGCAATAAGGCAAT 12
CAGGGCGTTCAGTTCGATGCA 294
CACGGAGGCGCGCGATGGTAG 16
AACTTTGGCGGACACGTTTCT 24
GCCCTTGACATCCCGGTCGCG 161
TGAACATGGGTCGACCACGAT 1449
CGAGGGCGGCGGCGCCGGCAG 44
CATTGTCTGCGGATGGTTCGA 33
CATTAAACATTCCGCCTGGGC 10
GCGTCGGGGAGGTGCGAATAC 521
CCCAAGGGTATGGCTGTTCGC 2451
TCGGCTCATCGCATCCTGGGG 17
CACCTCGATGTCGGCTCATCG 10
GGCTATGGGGACTCACCGTCT 940
GTAGCGTTTGCGTCGGTATCT 20
GAGTGAAATAGTACCTGAAAC 15
AACCAGGATGTTGGCTTAGAA 55
GAACCTGCGGCTGGATCACCT 27
GCGGAGCGGCTGCACCCGATA 50
GCCTAACACATGCAAGTCGAG 12
GGGAATTTTGGACAATGGGCG 45
TTTGGTTGCGGGGGCAGGATT 36
GATCTAGCCATGAGCAGGTTG 65
CGGGAGAGTAGGTCGCTGCCA 6024
GGGCCGTAGCTCAGCTGGGAC 17
GGAATTTTGGACAATGGGCGC 43
GCAAGTCGAGCGCCCCGCAAG 108
ACCTGAAACCGGATGCCTACG 40
TCGACCGAATACCCCCGGGGG 4503
GTACCCCGGGGATAACAGGCT 54
ATTGTCTGCGGATGGTTCGAG 19
GGGCAGCGAGCACGCGAGTGT 43
CCTGGGGAACTGAAACATCTT 32
ATCTAAGCGGGAAACCCACCT 159
TCGGCAGACACACGGCGGGTG 334
TCATAGTGATCCGGTGGTCCC 665
ATATTCGTGGGCCTGCAGGTG 163
CTGGACCATTACTGACGCTGT 11
ACCATCTAAGGTCCCTAAGTT 12
CCGTGAAGATGCGGGGTTCCT 52
GCCCTTACGGGCTGGGCTACA 13
CCCCGACGAGGGGAGTGAAAC 12
TGATCCGCCACACTGGGACTG 29
GACATGAGTAACGATAAAGGG 19
GACACGTTTCTTGGTAAGAAC 51
ATCCGGTGGTCCCGCGTGGAA 37
TAAACCGACACTGGTGGACTG 50
ATTGGATTGATCTTGCAGCGA 12
TGGATTGATCTTGCAGCGAAG 19

GCTGGGCTACACACGTGATAC 19
TGGAAGTGCGGCAACGCATGC 28
TACACACCGCCCGTCACACCA 24
CATGCACAGGCGATGAAGGAC 439
GGCGCTGACACGGATTTGACC 20
GGTATCTGGGCTTGTAGCTCA 25
ACACCAGTGGCGAAGGCGGCC 12
ACTCCGGGGATAACAGGCTGA 18
GAGTATGGTAGAGGTGAGTGG 15
TGCACAGGCGATGAAGGACGT 182
TCCCGGGTCTTGTACACACCG 18
ATAAAAGGTACCCCGGGGATA 69
TACGGCCCAGACTCCTACGGG 18
TGTGGATGCACGTATGTGCGT 344
CCTGGCTCAGAACGAACGCTA 15
GCTCAGCTGGGAGAGCACCTG 744
TCCCCGCTGTCTCCAACGCAG 670
CACAGGCGATGAAGGACGTGA 121
GGGTAGCTATATACGGACGGG 22
GATGCACGTATGTGCGTGGTA 11
CTTCGGAAGAAGCGTGACCTC 106
TTGATAGGCCGGGTGTGGAAG 25
TGACAGGATCTGTCCCTAGTA 89
ATGGCGGAGCGGCTGCACCAG 12
TGTAGGATAGGTGGTAGACTT 61
AAGAGTCCATATCGACGGGGT 20
CCAAGGTTTGTCCTGGGTGAC 43
GCATATAGACCGTACCCTAAA 255
ATGAAGCTTACCGGTACTAAT 89
GGGCGTAAAGCGCACGTAGGC 243
GATCTAGAAGCCCGGCACCGC 31
TTTGACTGGGGCGGTCGCCTC 172
AGGCTCAGAACGGTCGGAAAT 281
TGGGCGCAAGCCTGATCCAGC 163
TCAACGGATAAAAGGTACCCC 33
TGGACCATTACTGACGCTGGG 17
TGAAGTCGGAATCGCTAGTAA 238
CTGGACCATTACTGACGCTGC 15
TGAAGGACGTGATACGCTGCG 35
CTGTTCGCCATTTAAAGCGGT 35
GGCATTGTCTGCGGATGGTTC 55
CCTATCAGAGCCGTGGAAGAC 30
GCTCTTTCACCGGTGAAGATA 23
AGTAGGGCAATAAGGCAATAT 10
CGTTCCGTAAGCCTGTGAAGG 18
GGGCTGGGCCACACACGTGCT 12
GGACACAGGTGCTGCATGGCT 12
ACTGGTAGAGAATACCAAGGC 74
CGGACCAGACTCCTACGGGAG 20
CGTGAAATCCTGTCTGAACAT 84
AGAGGATGATCAGCCACACTG 233
ACCATTACTGACGCTGAGGGG 107
TCACCGTCTTACTGATCCTAA 155
CCTGGCTCAGAACGAACGCTG 3446

CGGGCTGGGCTACACACGGGC 18
TGATCTAGAAGCCCGGCACCG 41
TGACTGCGTACCTTTTGTATA 12
GTTCAAGTCCTCCCAGGCCCA 130
ACACCAGTGGCGAAGGCGGCT 2721
AGACCGTACCCTAAACCGACA 974
GGAGCGGCTGCACCCGATCCC 1115
GTACTCGTGCATGACCGATAG 30
TACGGGCTGGGCTACACACGA 14
CACAGGTGCTGCATGGCTGTC 40
ACGAGGCGCTGACACGGATTT 57
AACGGCGGCCGTAACAATAAC 12
ATAGACCGTACCCTAAACCGA 1495
ACTGACGCTGAGGTGCGAAAG 1106
GAAGCCCCGGCTAACTTCGTG 98
CGAATGGCGTAACGACTTCCC 11
AGGTATCGGAAGTGAGAATGC 20
TATGGATGTCTAACTGCGGCC 105
CTTGGCCGTGAAACGCTCCAG 15
CGGGCTGGGCTACACACGTGT 12
GGATGACTTGTGGCTAGGGGT 13719
GGCCGTGAAACGCTCGAGCGC 10
AGCGTCGACCGAATACCCCCG 5374
ATGAGCCTGACGAGCTACCGG 53
AAAACTCGACCGAAGTGGGTG 10
CTGAGACACGGCCCAGACTCC 374
ACTGAAACATCTAAGTACCCA 52
AGGCGCAGCGAAAGCGAGTCT 27
GAGACTCCGCTAGTAGTGGCG 14
GTGAAGTCGAAACAAGGTAGC 27
GAAAGTCGGCCATAGTGATCC 10
GGTCCCTAAGTTATGGCTAAG 111
CTGAGCAGGGTTAGCCGGCAC 15
AACTTCGTGCCAGCAGCCGCG 21
CCGAAGGCGCTGTGCTAACCC 21
GAGAATGCTGACATGAGTAAC 132
CTCGGCCGTAAAACGCTCCAG 12
AAACCCACCTGAAAACGAGTA 10
AGAGCTGAGTTTTGATGGATA 283
TTGGATGTTTGGTTAGGCGGA 65
ACATGTGTAGGATAGGTGGTC 11
CGTGACGACATGTGTAGGATA 355
ATGAGCAGGTTGAAGGTACGG 477
TTGATCTTGCAGCGAAGCGGT 237
CGGCAAAATGCACGCGTAACT 30
CGATGTCGGCTCATCGCATCC 12
GCCGGGGATAACAGGCTGATG 16
ATCCGCCACACTGGGACTGAG 20
ACCGTATGTGCCCTTCGGGGG 56
GAGGAGGTCGCTGCCAGGTCT 14
ATGCCTACAAACAGTTGGAGC 45
TCGTGAGATGTTGGGTTAAGT 156
TCTGAACATGGGTCGACCACG 1419
AAGTCAGGGGTGAAATCCCGG 32

GGATGACTTGTGGCTAGGGGC 60
CGGGCTGGGCTACACACGTGC 52043
GTCGGCCCATGTGGGCCGCCC 21
CGGAGGCGCGCAATGGTAGGC 10
GCGTAACAGCTCACTGGTCTA 46
CATAACCACCAGGTCGGCGAA 60
CACCAGTGGCGAAGGCGGCTC 2580
TCAGAACGAACGCTGGCGGCC 17
TACCCAGAGGAAAGGACATCA 33
AGCAGCCCGGTAGCTCGTCAG 239
ATCATGTTGGTGTTGAGACGG 22
CGCGGGGTAGAGCAGCCCGGT 10
ACGCCGTAAACGATGAATGTT 182
AGTGATCTAGCCATGAGCAGG 99
CGGGCTGGGCTACACATGTGC 15
CAACGCATGCAGCTTACCGGT 33
ACTGACGCTGAGGTGCGAAAA 13
GTAACCGGAGAAGAAGCCCCG 841
GTAGAGCAGCCCGGTAGCTCG 10
GAGCCCAAGGTTTGTCCTGGG 38
GGAATATTGGACAATGGGCGA 14
CTCGATCGACTTGATCACTCC 410
TGAGACATCCTGGAGGTATCG 114
ATATAGACCGTACCCTAAACC 1550
CGCAGACTCAGTGAAATTGAA 78
CGAGCGAACGCGGACCAGGCG 12
GCGGTACGTGAGTTGGGTTCA 100
GATGTTGGCTTAGAAGCAGCA 28
CGGATGCCTACAAACAGTTGG 47
GTCCGTCGTGGAGAGGGCAAC 36
GCAAGGAGGCAGGCGACCACG 106
GGGGCTGGAGCAGGTCCCAAG 14
GTATAATGGGTCAGCGACTTA 82
AATTCGAAGCAACGCGCAGAA 31
AGAACGGTCGGAAATCGTTCG 63
AAAAGCACCCCGACGAGGGGA 31
CAGGTTGAAGGTACGGTAACA 190
AACCGCTGAAGGCATCTAAGC 10
CGAGACTCCGCTAGTAGTGGC 18
CTACGGGAGGCAGCAGTGGGG 44
GGTTAGAAGCGAACCTGGGGA 785
CTGTTTACCAAAAACACAGGG 19
TTACGGGCTGGGCTACACACT 14
GATCTTGCAGCGAAGCGGTTC 223
ACGGGCTGGGCTACACACGCG 13
GCTGTGCTAACCGCAAGGAGG 28
CCGTACCCTAAACCGACACTG 723
CCCAGACTCCTACGGGAGGCG 18
ACGCGGACCAGGCCAGTGGCC 36
TGCGTTGAAGGAACTCGGCAA 349
GCGGCTGGATCACCTCCTTTC 166
GTCGGGTAAGTTCCGACCTGC 24
CTAGAAGCCCGGCACCGCAGA 12
ACCACGATCCAAGCCTAAGTA 37

GGCAGCGAGCACGCGAGTGTG 35
CCTGACTGCAAGACTGACAAG 70
GAATACGTTCCCGGGCCTTGT 41
AGTGCATGAAGTTGGAATCGC 50
TGGACCTGTTGTGGCGCCAGC 3951
TGTTGCAATAGATCGGGATGA 24
TGTGGAAGTGCGGCAACGCAT 81
ACTCAAAGGAATTGACGGGGG 206
GCAGCAGTGGGGAATTTTGGA 43
GGAGGCGCGCGATGGTAGGCG 24
GCCGTAAACGATGAATGTTAG 210
ATTCGGAAGAACACCAGTGGC 42
GTTCAGTTCGATGCATTAGAC 339
CGCTGCGATAAGCGTCGGGGA 57
GGTCGCAAGATTAAAACTCAA 148
AGGTCCCTAAGTTATGGCTAA 112
AGTACTAGTCGGCAGACACAC 18
GCTCAACGGATAAAAGGTACG 90
GAGTGTAGAGGTGAAATTCGT 90
GTCGTGGAGAGGGCAACAACC 24
GGCTCAACCCCGGAACTGCCT 35
GCGTAACGACTTCCCCGCTGT 94
CCCAGACTCCTACGGGAGGCA 1289
TACCGGGGAGTACTAGTCGGC 704
ACGCGGACCAGGCCAGTGGCT 6418
GCGTTCAGTTCGATGCATTAG 331
GCGGGAAACCCACCTGAAAAC 16
AAGGGTATGGCTGTTCGCCAT 34
AGCAAACAGGATTAGATACCC 17
GAGACACTATCCTTCAGTTAG 16
TAACTTCGGAAGAAGCGTGAC 27
CTGCTTTGCAAGCAGGGGGTC 91
CCGAGAGGAAGGTGGGGATGA 102
GCGTTGAAGGAACTCGGCAAA 205
TTCTCTTTCTTCATTGTTGAT 18
TTACGGGCTGGGCTACACACC 45
GGGGGCCCGCACAAGCGGTGG 207
GAACTGAAACATCTAAGTACA 13
AGCGACTTAGTGTATCGAGCA 103
GGGTCCAGGACCGTGTATGGT 37
GACACGGCCCAGACTCCTACG 290
AGGTCCCTAAGTTATGGCTAG 11
AGGTGAAAAGCACCCCGACGA 28
GAAGTCCTTGAGTAGGGCGGG 744
CCTGCCGCCAGCGTTCGTTCT 10
GTAACGGAGGCGCGCGATGGT 674
GCAGGTCCCAAGGGTTTGGCT 11
GGTAGCGAAATTCCTTGTCGG 400
CGAAGCAACGCGCAGAACCTT 24
GTGTATCGAGCAAGCTTAAGC 32
TCCTCATGGCCCTTACGGGCT 107
AGTACGGTCGCAAGATTAAAA 95
CAGGGTTAGCCGGCCCCTAAG 1014
GAAGTCTTGAGTATGGTAGAG 33

GATGACTTGTGGCTAGGGGAG 15
CAATGAGATTGATCAAGTGTC 11
ACTCAAAGGAATTGACGGGGA 22
GCGTGGGGTCGGAGGTTCAAC 12
AATACCAAGGCGCTTGAGAGA 62
TTAGGTAGAGCGTCGACCGAA 231
ATCCCGGTCCTCTCGTACTAG 10
TGAAATTCGTAGATATTCGGA 281
GGGTAGAGCAGCCCGGTAGCT 10
GGAGGCGCGCGATGGTAGGCA 41
AGCTCAGCTGGGAGAGCGCCT 23
ATTTACTGGGCGTAAAGCGCA 2534
CAGGTCCCAAGGGTTTGGCTG 11
GTAGATATTCGGAAGAACACC 39
ACTCCGAATACCGGGGAGTAC 20
ATGGGGCAACCCACCTTAGAT 31
CATTAAACATTCCGCCTGGGG 596
CACTGGGACTGAGACACGGCC 940
GATGTTGGGTTAAGTCCCGCA 28
CACGTGAAATCCTGTCTGAAC 44
CGGAGATATTCGGAGGAACAC 19
TACTGGGCGTAAAGCGCACGT 1235
GAGTTTGGTTAGGATCAGTAA 14
TTCGGATTGCACTCTGCAACT 109
GATAAAAGGTACTCCGGGGAT 59
CGATAGCGAACCAGTACCGTG 137
GACAGTTCGGTCCCTATCTGC 214
TCAGCGACTTAGTGTATCGAG 182
CCGGGAAACGCTCCAGCGCCA 10
AAGCGTCGGGGAGGTGCGAAT 482
GTGACTGGGGTGAAGTCGTAA 11
TTGGGCACTCTAAGGGGACTG 811
AAGAGATTTTGGACGGTTTAG 13
CAACAACCCTGACCACCATCT 36
GTGGGGAATATTGGACAATGG 65
GGTGAAGTCGGAACAAGGTAG 19
GTTGATAGGCCGGGTGTGGAA 45
CATCTCAGTTCGGATTGCACT 104
CCTGGGGAACTGAAACATCTA 1696
CATTACTGACGCTGAGGTGGG 11
ACCTGAAACCGGATGCCTACC 26
GTGAAATTGAATTCCCCGTGA 28
CAGGCTGATGACCCCCAAGAA 10
GGGCCGTAGCTCAGCTGGGAG 10826
ATTGTATCTCGAGAAGCTGGT 24
CGGGAGAGTAGGTCGCTGCCT 29
ACTGATCCTAACCAAACTCCG 103
GCGGAGCGGCTGCACCCGATT 11
ATGACGTCAAGTCCTCATGGC 27
GTTGGAGCCCAAGGTTTGTCC 86
GCATCTAAGCGGGAAACCCAC 157
GGGAGTACTAGTCGGCAGACA 19
TCGTGAGACAGTTCGGTCTCT 12
TACATAGGGGTTAGAAGCGAA 11

CTGAGTTTTGATGGATATTGG 33
TTGAGAGGATGATCAGCCACA 17
CTCCAAAAGCCGTCTCAGTTC 20
CGGGGTGGAGCAGCCCGGTAG 202
CTTTTGTATAATGGGTCAGCG 33
ATGTCGGCTCATCGCATCCTG 17
CGCCGGGGATAACAGGCTGAT 19
CGGTCCTAAGGTAGCGAAATT 78
GAGAGTAGGTCGCTGCCAGGT 4876
TATGAGCCTGACGAGCTACCG 54
ACCAGTACCGTGAGGGAAAGG 24
CTCAACCCCGGAACTGCCTTT 18
GAGAACTGCGTTGAAGGAACA 20
GATCGGGATGACTTGTGGCTA 427
TGGGGCGGTCGCCTCCTAAAG 15
TGGGCTATGGGGACTCACCGT 1441
TGATCCTAACCAAACTCCGAA 91
AGTGGGAAAGGATGTGAGGAT 136
CAGCCGCGGTAATACGAAGGG 53
AGTGAGAATGCTGACATGAGT 126
GATCGGCCCGCGTTGGATTAG 137
CTTTGATACTGGAAGTCTTGA 20
CCAAAAACACAGGGCTCTGCG 63
GGTGAATACGTTCCCGGGCCT 158
GTTCAGAACGTCGTGAGACAG 101
GCGGAGCGGCTGCACCCGATC 2939
CGTTCCCGGGTCTTGTACACA 13
AGGAATATTGACAGGATCTGT 787
GACCAGGCCAGTGGCTTTTGT 397
TGGTACTTCGTCTCAAGACGC 136
CCCTTACGGGCTGGGCTACAC 194
CATTTGGTGGATGCCTTGGCA 41
CATGCAGCTTACCGGTACTAA 38
TAGGCGGAAGAGATTTTGGAC 37
AATCAAACTTGGAGATAGCTG 15
ATCTAAGTACCCAGAGGAAAG 162
GGCAGTTTGACTGGGGCGGTC 25
ACTGACAAGTCGAGCAGAGAC 51
GGGCCGTAGCTCAGCTGGGAA 72
CAGGCTGATGACCCCCAAGAG 4891
ACGAAAGTCGGTCATAGTGAT 153
CGGGAGAGTAGGTCGCTGCCC 57
GTACGGTCGCAAGATTAAAAC 245
ACGGAGGCGCGCAATGGTAGG 10
CAGAACGAACGCTGGCGGCAG 2407
TATTGGACAATGGGCGAAAGC 43
ATAGATCGGGATGACTTGTGG 496
ACTCGGCAAAATGCACGCGTA 68
GCGTACGGCGCGTGAGCGAGA 16
GAGCGAACGCGGACCAGGCCA 1579
CGAATGGGGCAACCCACCTTA 12
CTGGACCATTACTGACGCTGG 48
AAGGTACCCCGGGGATAACAG 72
GGAGACAGGTGCTGCATGGCT 240

AACACGCACTGGAGGACCGAA 17
TCGTGAAGAGAAGATGTAATC 22
AGAGCAGCCCGGTAGCTCGTC 10
GAGCAGCCCGGTAGCTCGTCA 219
ATTCCGAGTGTAGAGGTGAAA 332
CACGATGAATGTTAGCCGTCG 10
AGGCGACGATCCATAGCTGGT 53
CGGACGGGATAACCGCTGAAG 2412
AACCGCAAGGAGGCAGGCGAC 13
TCCTACGGGAGGCAGCAGTGG 64
CGAGTCTGAACAGGGCGTTCA 80
TACGGGCTGGGCTACACACGT 3149
GTGGTTATGGCGGAGCGGCTG 1032
CGTAGGCGGACTTTTAAGTCA 32
CCTAAGGTAGCGAAATTCCTT 82
GGAGGTATCGGAAGTGAGAAT 13
CGAGGCCGAAAGGCGTAGTCG 13
TCAAAGGAATTGACGGGGACC 22
GACGAAAGTCGGTCATAGTGA 124
ATTCCCTATCAGAGCCGTGGA 50
CAGTCGTGAGACATCCTGGAG 28
AGATACCCTGGTAGTCCACGC 358
CGGTCCAGACTCCTACGGGAG 15
GGGCACTCTAAGGTGACTGCC 18
GCAACCATAGGGGGGTGGCAC 33
GATAACCGCTGAAGGCATCTA 51
GTTGATTTGTGAGTAGTTGGG 10
TTTAAGCAGGAACCCTTGGAC 10
CCGCTGTCTCCAACGCAGACT 810
GTGGAAGTGCGGCAACGCATG 41
GGTGACGGATCGCGTGTGTTG 32
CAAAATGCACGCGTAACTTCG 38
TATGTGCCCTTCGGGGGAAAG 45
CATGAAGTCGGAATCGCTAGT 82
AGTCAGCCTGACGATCGCTTG 19
AAGCGGGAAACCCACCTGAAA 145
ACATGAGTAACGATAAAGGGA 32
ACCAAGTGATCTAGCCATGAG 87
GCTCAGAACGGTCGGAAATCG 556
GCCATAGCTCAGCTGGGAGAG 644
CGGCAGACACACGGCGGGTGC 305
CGGCAGGCTTAACACATGCAA 11
GAGGTGAAATTCGTAGATATT 77
AATGTTAGCCGTCGGGGTGTT 267
GAATGGCGTAACGACTTCCCC 10
CAATCAAACTTGGAGATAGCT 15
AGTCGAGCAGAGACGAAAGTC 44
TAGCCGTAGGGGAACCTGCGG 30
TAGATATTCGGAAGAACACCA 33
CTGGACCATTACTGACGCTGA 22993
GCTCAGGACGAACGCTGGCGG 18
TAAAACTCAAAGGAATTGACG 97
AACCATAGGGGGGTGGCACAG 16
GACCACGATCCAAGCCTAAGT 38

GTCGGCTCATCGCATCCTGGG 15
GCGCTGTGCTAACCGCAAGGA 32
AGGCCCTAGGGTTGTAAAGCT 6990
AGGAACTCGGCAAAATGCACG 595
TCCCTATCTGCCGTGGGTGTA 219
ATCTCAGTTCGGATTGCACTC 114
GTTGGATGTTTGGTTAGGCGG 53
TTTTAAGTCAGGGGTGAAATC 20
AGTTCGGTCCCTATCTGCCGT 958
AGGCATCTAAGCGGGAAACCC 52
ACACCAGTGGCGAAGGCGGCG 10
TGGATGTCTAACTGCGGCCCG 70
GCGAGTCTGAACAGGGCGTTC 125
GGCGTTCAGTTCGATGCATTA 332
CCTAAACCGACACTGGTGGAC 532
AGGTGTAGGCGCAGCGAAAGC 45
GCTGTCGTCAGCTCGTGTCGT 90
GGCTGCGCCTGTTCTTTGCCT 13
GGTCGCGGTTAGTGGAGACAC 64
TGAAGGCAATATGGAAGTAGG 19
ATTCGAAGCAACGCGCAGAAC 49
TCGTTTACGGCGTGGACTACC 12
AGACACTATCCTTCAGTTAGG 16
ACACTATCCTTCAGTTAGGCT 21
AACCCCGGAACTGCCTTTGAT 82
GCTATGTACGGACGGGATAAC 11
GACTTTGAAGCAGGGGCGCCA 687
TTCCGAGTGTAGAGGTGAAAT 357
CGGGCTGGGCTACACACGTGG 52
GGATGACTTGTGGCTAGGGGG 186
GCCGTCTCAGTTCGGATTGCA 22
CAACGGATAAAAGGTACCCCG 49
TACTGATCCTAACCAAACTCC 20
AGCTCACTGGTCTAAATAAGG 18
TGGAGGACCGAACCCATATCT 155
TAGACCGTACCCTAAACCGAC 1537
TTAAGGGCATTTGGTGGATGC 10
GAGGCGCGCGATGGTGGGCTC 21
AACGCATGCAGCTTACCGGTA 40
ACGCCTGCCCGGTGCTGGAAG 224
GCGACTGTTTACCAAAAACAC 21
GGAATCGCTAGTAATCGCGGA 1504
GGTAGCTATATACGGACGGGA 28
AAGGCCTTAGGGTTGTAAAGC 16
CTCACGGGCCGTACCGCAGCT 66
ATCGAACTGAACGCCCTGTTC 34
GTTGGCTTAGAAGCAGCCATC 63
GTACTAGTCGGCAGACACACG 15
GATGTTGGCTTAGAAGCAGCT 22
AGGAGGCAGGCGACCACGGTA 95
CGAGCGAACGCGGACCAGGCC 1731
CTTGATCACTCCCATTTACAA 29
CGGCCCATCAGGGCCGACGGC 41
ACATGTGTAGGATAGGTGGTA 1170

ACTTTGGCGGACACGTTTCTT 134
GGTCATAGTGATCCGGTGGTC 620
GTATCGAGCAAGCTTAAGCCG 18
AGATCGTCGGTTCTTTGAAAA 16
ACTGAAACATCTAAGTACCCT 10
TCAGAACGAACGCTGGCGGCA 2813
CCGGAGACAGGTGCTGCATGG 289
GGATGACTTGTGGCTAGGGGA 33
CATATCGACGGGGTTGTTTGG 80
CGGGCTGGGCTACACACGTGA 89
TACTCTCCCGCGTCTTGAGAC 32
AGGCCAGTGGCTTTTGTGAAT 18
TTTGCGTCGGTATCTGGGCTT 51
TCCGTCGTGGAGAGGGCAACA 25
GTCCCAAGGGTTTGGCTGTTC 10
AGGGGTGAAATCCCGGGGCTC 30
GTGTCGTGAGATGTTGGGTTA 127
ATAGCTTTACACTGGCATTCG 47
TTACTGACGCTGAGGGGCGAA 27
TTTACTGGGCGTAAAGCGCAC 2240
CGAATACCGGGGAGTACTAGT 31
AAATTCGTAGATATTCGGAGG 170
GATGTTGGCTTAGAAGCAGCC 275
ACCAAACTCCGAATACCGAGG 16
TGACTGATCTAGAAGCCCGGC 43
TACCCTAAACCGACACTGGTG 477
GGACACGTGAAATCCTGTCTG 22
TCTGAGAGGATGATCAGCCAC 130
GGAATATTGGACAATGGGCGC 546
GGTAATACGAAGGGGGCTAGC 130
GCCACACTGGGACTGAGACAC 605
CCGAAGGCGCTGTGCTAACCG 47
TTAGTAGTGGCGAGCGAACGC 25
GTTTGACTGGGGTGGTCGCCT 20
TTGCTACGGAATAACTCAGGG 221
GTGAGAGACTCCCTCGCCGAA 36
TAGATCGTCGGTTCTTTGAAA 16
TCCTTGAGTAGGGCGGGACAC 327
CTCATGGCCCTTACGGGCTGG 37
CAGCGCCAATGGTACTTCGTC 51
TGAGATACGGCCCAGACTCCT 12
CATGACCGATAGCGAACCAGT 139
AGCTCGATCGACTTGATCACT 43
TCGACGGGGTTGTTTGGCACC 1552
TATAGGGTCTGACGCCTGCCC 752
TACGCCGGGGATAACAGGCTG 76
TCTGAGCAGGGTTAGCCGGCA 22
GCTACGGAATAACTCAGGGAA 499
GACCATTACTGACGCTGAGGA 40
GCTCAAGCCATACACCGAAGC 374
TCGTAGATATTCGGAGGAACG 36
TTCGGGGGAAAGATTTATCGG 23
TCGACCACGATCCAAGCCTAA 34
CGTCGGGGTGTTTACACTTCG 93

TAAGGAAGATCGAGAATTGGA 49
ATAACGGTCCTAAGGTAGCGA 39
TGACTGGGGTGAAGTCGTAAC 13
CAAGCCTGATCCAGCCATGCC 214
TGGGTAGTTTGACTGGGGCGG 112
TGATAAGCGTGGGGTCGGAGG 34
AGGTGGTAGACTTTGAAGCAG 114
CGACCGAATACCCCCGGGGGA 10
CCGCCATCACCGATTGTATCT 16
GCATCCTGGGGCTGGAGCAGG 448
AGAGCACTGGATGGGCTATGG 26
GGATGTCTAACTGCGGCCCGT 62
GATCCAAGCCTAAGTACTCGT 15
GTAGAGAATACCAAGGCGCTT 53
TCCCGTACACGTAGAATAGCA 442
GTTTGGCACCTCGATGTCGGC 17
AAGCCGAGAGGAAGGTGGGGA 17
GGAGATATTCGGAGGAACACC 13
GGTTGTCGTCAGCTCGTGTCG 10
TTCCGACCTGCACGAATGGCG 17
AGCAGGGGGTCGTCGGTTCGA 386
CCCGGTAGCTCGTCAGGCTCA 36
GTAAACGATGAATGTTAGCCG 74
GTAAGCCTGTGAAGGGACAGT 15
GACCATTACTGACGCTGAGGG 107
CAGGAGGTTGGCTTAGAAGCA 28
ATCTCGAGAAGCTGGTCTTTC 180
CAACGCATGAAGCTTACCGGT 39
CTGCGATAAGCGTCGGGGAGG 33
GGCCGTGAAACGCTCCAGCGC 35088
TCGTAGATATTCGGAGGAACA 7088
ACGGTCCAGACTCCTACGGGA 15
GTCGCGGTTAGTGGAGACACT 41
TATGGCGGAGCGGCTGCACCA 26
AAGTACTCGTGCATGACCGAT 25
AAGGCTCACCAAGGCGACGAT 12
AGTCTTGAGTATGGTAGAGGT 32
ATGGCTGTCGTCAGCTCGTGT 265
GAGAGGATGATCAGCCACACT 159
AGGCCGGGTGTGGAAGTGCGG 561
GGCCGTGAAACGCTCCGGCGC 10
GTATGTGCGTGGTAGCGGAGC 52
ATTGACAGGATCTGTCCCTAG 291
CGACCGAATACCCCCGGGGGG 20
TCAACGGATAAAAGGTACTCC 79
GTCCTTGAGTAGGGCGGGACC 10
CCGCAGGTTCAAATCCTGCCC 12
ACTGGAAGTCTTGAGTATGGT 39
TGGGAACCACGTTAATATTCG 23
GCTGATGACCCCCAAGAGTCA 21
GAGCAGGGTTAGCCGGCCCCG 34
AAACGAGACTCCGCTAGTAGT 23
CCATCTAAGGTCCCTAAGTTA 12
AGAGGACCGGGATGGACGTAT 4118

ACCAAGGCGCTTGAGAGAACT 124
AGCAGGGTTAGCCGGCCCCTA 1910
GCTCAGTTGGTTAGAGCACAC 18
GCACTGGATGGGCTATGGGGA 1242
GAACTCGGCCGTGAAACGCTC 6828
ACAGGCGATGAAGGACGTGAT 139
GCGCGCGATGGTAGGCTCAGC 12
CCCGTACACGTAGAATAGCAG 393
TGGTTAGGCGGAAGAGATTTT 11
TACCCTTTGATCCGACGATTT 869
TTGTCCTGGGTGACAGCGTAC 61
CCACGATCCAAGCCTAAGTAC 45
GAGTACTAGTCGGCAGACACA 15
ATTAGAACATAGATCGCAGGC 22
CGCCGTAAACGATGAATGTTA 176
ACGTATAGGGCCTGACGCCTG 13
CCGGGTCTTGTACACACCGCC 30
GGATGTTGGCTTAGAAGCAGT 11
TGTGAAGGGACAGTCGTGAGA 35
GGGCTAGGCTACACACGTGCT 16
TGCGAATACCCTTTGATCCGA 20
ATGGCCCTTACGGGCTGGGCT 119
CACTGGTGGACTGGTAGAGAA 1865
ACCACGGTAGGGTCAGCGACT 2108
GGCCATAGCTCAGCTGGGAGA 820
GTGGGAAAGGATGTGAGGATC 183
CCGAAAGGCGTAGTCGATGGG 240
GGCCTGCAGGTGGTGACGGAT 72
TGCAATAGATCGGGATGACTT 47
AACCGACACTGGTGGACTGGT 79
TCGGAAGAAGCGTGACCTCAC 518
ACCTGTTGTGGCGCCAGCCGC 3829
CACGTTAATATTCGTGGGCCT 15
CGTTGTTCGGAATTACTGGGC 10
TTCGATGCATTAGACCCGAAA 34
AAGCGTAACAGCTCACTGGTC 399
CGCGGTGAATACGTTCCCGGG 206
ATGAGAGTGATCAAGTGTCTT 927
ATATTCGGAGGAACACCAGTC 12
GCATGAAGTTGGAATCGCTAG 74
GGATGTTGGCTTAGAAGCAGC 735
AGCGACTGTTTACCAAAAACA 15
GCCGAGAGGAAGGTGGGGATG 172
AACGCGGACCAGGCCAGTGGC 2615
TCAACGGATAAAAGGTACGCC 98
GAATACCCTTTGATCCGACGA 254
CTTTACACTGGCATTCGTGAC 446
ACGATGAATGTTAGCCGTCGG 7096
GATGCGGGGTTCCTGCGGTTA 70
CGAAAGCGAGTCTGAACAGGG 36
ACGTGATACGCTGCGATAAGC 39
CTCTACACTCGGAATTCCACT 10
CCGGGTCCAGGACCGTGTATG 36
GGATTGATCTTGCAGCGAAGC 39

TCCCGGTCGCGGTTAGTGGAG 224
ATTCGGAGGAACACCAGTGGC 2822
ACACACCAACTTCGATCCGAA 14
TCGGAAATCGTTCGTCGAGTG 92
ATAAGCGTGGGGTCGGAGGTT 158
GGCCGTGAAACGCTCCAGAGC 10
AATACCGGGGAGTACTAGTCG 67
TGTAAAGCTCTTTCACCGGTG 821
GTTGGTGGGGTAAAGGCTCAC 21
GTAGGCGCAGCGAAAGCGAGT 18
GTTAGCCGTCGGGGTGTTTAC 90
AGCCGTCGGGGTGTTTACACT 218
CGGGCTGGGCTACACACGCGC 15
AGAGAAACGTGGGCGGCATTG 11
TGCACTCTGCAACTCGAGTGC 178
ACAGACCAGGGGGTAGCGACT 133
CCGTGAAACGCTCCAGCGCAA 12
GCAGCGAAGCGGTTCCAGGAG 11
ATCTATTTAGGTAGAGCGTCG 25
GGGCTGGGCTACACACGTGTT 10
GCGAACCTGGGGAACTGAAAG 47
CTCGGCAAAATGCACGCGTAA 49
GGAGGCGCGCGATGGTAGACT 15
GGGCATGAAGTTGGAATCGCT 14
GCCGAAAGGCGTAGTCGATGG 192
ATGCACGTATGTGCGTGGTAG 27
GGTAGAGCAGCCCGGTAGCTC 10
CGCTGGCGGCAGGCTTAACAC 729
TGTTCGCCATTTAAAGCGGTA 34
AAGGACATCAAACGAGACTCC 300
TCGGAATCGCTAGTAATCGCG 90
CGCTGCCAGGTCTGCAAAGCA 10
GAACACCAGTGGCGAAGGCGG 784
CGAACCTGGGGAACTGAAACC 182
GGGGCCGTAGCTCAGCTGGGC 17
GAGACTCCCTCGCCGAAAGTC 22
CCAGTAAACGGCGGCCGTAAC 15
GTCTGACGCCTGCCCGGTGCT 1318
TATGGTGGGTAGTTTGACTGG 35
AGACCACCACGTTGATAGGCC 87
CACCTGCTTTGCAAGCAGGGG 86
ATTACTGACGCTGAGGTGCGG 23
CATCTAAGCGGGAAACCCACC 155
AAGGCGCGCGATGGTAGGCTC 12
GGCGCTGTGCTAACCGCAAGG 33
GTGGGGTAAAGGCTCACCAAG 27
TCGATGTCGGCTCATCGCATC 12
CGAACGCGGACCAGGCCAGTG 2514
CTGGGGTGAAGTCGTAACAAG 34
GGCGGCGGCGCCGGCAGCGGC 48
TGGACAATGGGCGCAAGCCTG 1392
TTCCCCGCTGTCTCCAACGCC 11
GTGGGTGATAGTCCCGTACAC 28
AAACATCTAAGTACCCAGAGG 47

GCAGCGAAGCGGTTCCAGGAA 115
AACGAGCATTTGCAGTCGAAT 11
TGCACGAATGGCGTAACGACT 16
GGAAGAAGCGTGACCTCACTA 29
GGTGCTAACGTCCGTCGTGGA 31
CTGACAAGTCGAGCAGAGACG 18
GCCGTGGGTGTAGGAATATTG 49
ATCCTGGAGGTATCGGAAGTG 20
GCGAACCTGGGGAACTGAAAA 263
AGGTTGAAGGTACGGTAACAC 137
GAACCAGTACCGTGAGGGAAA 78
GTATAGGGTCTGACGCCTGCC 905
ATTACTGACGCTGAGGTGCGA 8576
GTAGCTCGTCAGGCTCATAAC 83
CGGGGGCCCGCACAAGCGGTG 233
ACTGGTGGACTGGTAGAGAAT 2592
TTTTGTATAATGGGTCAGCGA 36
CCCCCGGGGGTAGAGCACTGG 365
CGAACCTGGGGAACTGAAACT 91
TTGGAGATAGCTGGTTCTCCG 20
GTGAGTGGAATTCCGAGTGTA 214
GTCTGACGCCTGCCCGGTGCC 12
CTTTGATCCGACGATTTCCGA 671
AATAAGGTTAGCGGGCTTTTT 32
GTGGACCTGTTGTGGCGCCAG 3783
GCAGGGGGTCGTCGGTTCGAT 821
GTTGGAATCGCTAGTAATCGC 1286
AGAAGCGAACCTGGGGAACTA 10
AGGTACGGTAACACGTACTGG 30
TGACGCTGCTGGCCCTGCGCA 23
ATAGATCGCAGGCCAGTCAGC 192
TAAAGCTCTTTCACCGGTGAA 942
GTAGCTCAGCTGGGAGAGCAA 23
CCGAAGTGGGTGATAGTCCCG 325
AGGCTGATGACCCCCAAGAGA 19
GGCCAGTCAGCCTGACGATCG 45
GGAAAGGACATCAAACGAGAC 22
TGTCGACTCATCGCATCCTGG 166
GCAAGATTAAAACTCAAAGGA 118
TGCCACCCCCCTATGGTTGCC 15
CTGGGACTGAGACACGGCCCA 225
CAAGTCGAGCAGAGACGAAAG 32
TAGCGAAATTCCTTGTCGGGT 923
TATTTAGGTAGAGCGTCGACC 21
GTCCATATCGACGGGGTTGTT 47
AAGTTGGAATCGCTAGTAATC 156
TGAAGTCGTAACAAGGTAGCC 52
GCTGACATGAGTAACGATAAA 14
TCGTGAGACAGTTCGGTCCCA 10
TGGTAGTCCACGCCGTAAACG 88
GAAAGTCTGCCTGTTCTGTAT 10
CCGGGATGGACGTATCTCTGG 862
CAATGGGCGCAAGCCTGATCC 119
ACTTAGTGTATCGAGCAAGCT 55

GGGTGTGACGCCTGCCCGGTG 11
GCGCAGAACCTTACCAGCCCG 11
GACTGTTTAGCAAAAACACAG 13
GATGCCTACAAACAGTTGGAG 48
GGAGGCGCGCGATGGTAGCCT 12
CCAAGAGTCCATATCGACGGG 14
GCGGACTTTTAAGTCAGGGGT 116
GATGAATGTTAGCCGTCGGGC 11
TCGTGACGACATGTGTAGGAT 475
AGGGGACTGCCGGTGATAAGC 2202
CGCATGCAGCTTACCGGTACT 166
TATCAGAGCCGTGGAAGACCA 11
TACGGACGGGATAACCGCTGA 583
CAATAGATCGGGATGACTTGT 220
GGAACTCGGCAAATTGCACGC 17
TTGTTGGAGCAACGCTGGATG 39
CGTCGGTTCGATCCCGTCCGG 205
GGCAGCAGTGGGGAATTTTGG 29
AGAAGCGAACCTGGGGAACTG 9556
TAACGACTTCCCCGCTGTCTC 86
ATGCCTTGGCATGCACAGGCG 48
CGATGAATGTTAGCCGTCGGG 7159
ATATTGACAGGATCTGTCCCT 204
TTGGTGGGGTAAAGGCTCACC 26
CAAAACAACCAGGATGTTGGC 86
GCTCTGCGAAGTCGCAAGACG 380
GCGTGAGTGATGAAGGCCCTA 19
AGGCGTAGTCGATGGGAACCA 68
ACGGAAAGACCCCGTGCACCT 215
TCACTGGACCATTACTGACGC 6873
CATTACTGACGCTGAGGGGCG 68
GGCGCGCGATGGTAGGCTCAG 2077
CTAGGGTTGTAAAGCTCTTTC 1785
TGGAATCGCTAGTAATCGCGG 1312
CGGAGTTTGGTTAGGATCAGT 17
GACTGGGGCGGTCGCCTCCTA 62
TGAGAGGATGATCAGCCACAC 153
CAAAAGCCATCTCAGTTCGGA 71
GAGAGGACCGGGATGGACGAA 12
CGGACACGTTTCTTGGTAAGA 65
GAAGTATAGGGTCTGACGCCT 12
TCTAGTCATCATAAATAAGGT 357
TCGGAGGAACACCAGTGGCGC 20
AAGGTACTCCGGGGATAACAG 90
ACGGATAAAAGGTACGCCGGG 1757
GCTGCGATAAGCGTCGGGGAG 73
TCTGAGCAGGGTTAGCCGGCC 11697
AGTCCTTGAGTAGGGCGGGAC 747
GATCAGCCACACTGGGACTGG 10
TCGACGGGGTTGTTTGGCACA 22
ACTTGAGAGTTTGATTCTGGC 11
AGTCCAAGGGTTCCTGCTTAA 10
CGATGGGAACCACGTTAATAT 20
TATGGCGGAGCGGCTGCACCT 22

TGGGCGTAAAGCGCACGTAGG 211
TCGTAGATATTCGGAGGAACT 17
GGCCGTGAAACGCTCCAGCGG 35
GGGGGTAGCGACTGTTTACCA 660
ACCCTGGTAGTCCACGCCGTA 286
GACCATTACTGACGCTGAGGC 35
GGAGGAACACCAGTGGCGAGG 31
TCTGAGCAGGGTTAGCTGGCC 14
GCTACGGAATAACTCAGGGAC 28
TCAAAGGAATTGACGGGGGCC 128
ACCTGGTGGTTATGGCGGAAC 27
TCGCGTAGTAGCGTTTGCGTC 10
GTTCCTGCGGTTAGACGGAAA 121
GGATGATCAGCCACACTGGGA 4504
GACGCTGAGGTGCGAAAGCGT 42
GGCGCAGCGAAAGCGAGTCTG 32
ACCAGGATGTTGGCTTAGAAG 738
AGGGGAACCTGCGGCTGGATC 89
GTAGGAATATTGACAGGATCT 712
ACGTTCCCGGGCCTTGTACAC 121
TTGTTTGGCACCTCGATGTCG 168
GTCGTCAGCTCGTGTCGTGAG 171
ATTTGCTACGGAATAACTCAG 24
CCGCACAAGCGGTGGAGCATG 11
TCGAGTGCAATGGCATAAGCC 56
AACCGGAGAAGAAGCCCCGGC 546
CCCCAGTAAACGGCGGCCGTA 25
GTTCCGTAAGCCTGTGAAGGG 17
GGCCGTGAAACGCTCCAGCGA 25
TCGTAGATATTCGGAGGAACC 148
GCACGAATGGCGTAACGACTT 16
TTGGTTAGGCGGAAGAGATTT 11
ACACGTTTCTTGGTAAGAACT 32
TCGGCCGTGAAACGCTCCACC 13
GTCTGAACATGGGTCGACCAC 291
AGTCCCGTACACGTAGAATAG 411
AAGCTTACCGGTACTAATAGC 91
AGAGTAGGTCGCTGCCAGGGC 10
GACCATTACTGACGCTGAGGT 16772
TGGTGGGTAGTTTGACTGGGG 71
TCGGTCCCTATCTGCCGTGGG 1517
GTTAATCTGAGCAGGGTTAGC 16
GATCAGCCACACTGGGACTGA 1757
AGCCGTCTCAGTTCGGATTGC 29
TACGCCGGGGATAACAGGCTC 22
TTTGGCGGACACGTTTCTTGG 133
TGGGGGTCATCAGCCTGTTAT 15
TCGACGGGGTTGTTTGGCACG 20
GAACGGTCGGAAATCGTTCGT 67
AAAACTCAAAGGAATTGACGG 136
GCTAACGCATTAAACATTCCG 130
AGATGTTGGGTTAAGTCCCGC 28
AATGCTGACATGAGTAACGAT 35
GCTGGGTTCAGAACGTCGTGA 20

TATGGCGGAGCGGCTGCACCC 9189
TCGTTCGTCGAGTGCAATGGC 59
TACCCCGGGGATAACAGGCTG 69
GAGCAGGGTTAGCCGGCCCCT 8588
GCTGATGACCCCCAAGAGTCC 3508
CCTTGTACACACCGCCCGTCA 11
CCGAATGGGGCAACCCACCTT 163
CAGTGGGGAATTTTGGACAAT 47
TCTAAGGAAGATCGAGAATTG 40
GCGAATACCCTTTGATCCGAC 211
TAAAAGGTACGCCGGGGATAA 197
GCGGGGTGGAGCAGCCCGGTA 315
GTCCTTGAGTAGGGCGGGACA 272
CGACCGAATACCCCCGGGGGT 2119
GGTCCAGACTCCTACGGGAGG 15
GAAGGCGGCTCACTGGACCAT 308
GAACTCGGCCGTGAAACGCTA 20
GGCTCAGGACGAACGCTGGCG 18
TTTACACACCAACTTCGATCC 14
AAGTCCCGCAACGAGCGCAAC 95
CTATGTACGGACGGGATAACC 11
AAGTGAGAATGCTGACATGAG 115
ACGCTTGATAAGCGTGGGGTC 17
ATGCTGACATGAGTAACGATA 22
AGGCCGAAAGGCGTAGTCGAT 27
CGCAGCGAAAGCGAGTCTGAA 24
TAGGGGAACCTGCGGCTGGAT 119
ACCAAACTCCGAATACCGGGA 11
GCGGACACGTTTCTTGGTAAG 80
TAGTTTGACTGGGGCGGTCGC 272
GGATGGACGTATCTCTGGTGG 736
GCGCGCGATGGTAGGCTCAGA 1884
TGGCTCAGAGCGAACGCTGGC 10
TGGCACAGACCAGGGGGTAGC 11
GACATCCCGGTCGCGGTTAGT 65
GTCTTGAGTATGGTAGAGGTG 22
ATCTAAGGTCCCTAAGTTATG 41
TGTACCGGGGCTCAAGCCATA 670
GGATGTTGGCTTAGAAGCAGG 19
CCGTGGGTGTAGGAATATTGA 56
ATATTCGGAGGAACACCAGTG 926
GGGGTAGAGCAGCCCGGTAGC 10
ACGGAGGCGCTCGATGGTAGG 10
ACTAATAGCTCGATCGACTTG 10
TGAGCAGGGTTAGCTGGCCCC 12
AGCATACCAAGGCGCTTGAGA 10
CCTCGATGTCGACTCATCGCA 27
GCCGTGAAACGCTCCACCGCC 11
GTACCTTTTGTATAATGGGTC 14
CGCCCCGCAAGGGGAGCGGCA 67
GAAGAACACCAGTGGCGAAGG 20
GCAGGTTCAAATCCTGCCCCC 12
GTAACGACTTCCCCGCTGTCT 87
CGCCAATGGTACTTCGTCTCA 39

TTGGCACCTCGATGTCGACTC 49
TCTGTTGCAATAGATCGGGAT 43
TGTGACGCCTGCCCGGTGCTG 10
ACGCAGACTCAGTGAAATTGA 123
CAGGGCCGACGGCCGGTCGGC 17
CCCGGGGGTAGAGCACTGGAT 233
GCGGAGCGTTCCGTAAGCCTG 22
AGTCGCAAGACGACGTATAGG 17
ACAGCTCACTGGTCTAAATAA 14
GATGGAGCAGCCCGGTAGCTC 15
CCCGGAACTGCCTTTGATACT 245
GGATGTTGGCTTAGAAGCAGA 139
TAGCCGGCCCCTAAGGCGAGG 24
GAGCAGGGTTAGCCGGCCACT 21
AAAACACAGGGCTCTGCGAAG 119
TGATCCTGGCTCAGAACGAAC 1523
GGGCACTCTAAGGGGACTGCC 704
GCTAACTTCGTGCCAGCAGCC 12
AGAGACTCCCTCGCCGAAAGT 21
AGGATAGGTGGTAGACTTTGA 48
TGAGTATGGTAGAGGTGAGTG 33
GATCGCAGGCCAGTCAGCCTG 34
TGTCGTGAGATGTTGGGTTAA 136
ACCAAACTCCGAATACCGGGG 26
CAAATGAATTGACGGGGGCCC 15
GACGATCCATAGCTGGTCTGA 108
CGAGCGCCCCGCAAGGGGAGC 72
GCGTGGGATCGGAGGTTCAAG 13
ATTGCTCACGGGCCGTACCGC 21
TGACATGAGTAACGATAAAGG 16
CTATAACGGTCCTAAGGTAGC 14
AGGTGCGAAAGCGTGGGGAGC 49
GTTATGGCGGAGCGGCTGCAC 1045
AGTACCCAGAGGAAAGGACAT 501
GGTAGAGGTGAGTGGAATTCC 36
GCATTTGGTGGATGCCTTGGC 47
GCTCCTGCATATAGACCGTAC 561
ACGGCCCAAACTCCTACGGGA 12
CCGAACTCGGCCGTGAAACCC 10
CGTCTCCGTGTTTTACATGGG 18
ACCGATTGTATCTCGAGAAGC 11
AACCACGTTAATATTCGTGGG 10
GTAAAGCGCACGTAGGCGGAC 335
GACGAGGCGCTGACACGGATT 49
GCGAACCTGGGGAACTGAAAT 32
TTGATCACTCCCATTTACAAT 17
CCTGACCACCATCTAAGGTCC 44
TTGATCCTGGCTCAGAACGAA 1334
CATCGTTTACGGCGTGGACTA 12
TGGGCCTGCAGGTGGTGACGG 144
ACGCATGCAGCTTACCGGTAC 219
CCCTAAACCGACACTGGTGGA 534
TGCACGCGTAACTTCGGAAGA 18
TGAAGGCCTTAGGGTTGTAAA 15

GTCGCAAGACGACGTATAGGG 21
GGGACTGAGACACGGCCCAGA 178
AGACTCCGCTAGTAGTGGCGA 14
CGTAGCTCAGCTGGGAGAGCA 6438
CCCACCTTAGATGACTAGAAA 54
GACAGGTGCTGCATGGCTGTC 19
ACAGTGGGCAGCGAGCACGCG 413
CTAGTCATCATAAATAAGGTA 81
GGTCTTGTACACACCGCCCGT 10
CAGGGTAGCTATATACGGACG 23
TACTCGTGCATGACCGATAGC 57
TGGAAGTCTTGAGTATGGTAG 29
GTCTGACGCCTGCCCGGTGCG 11
CTGGACCGGAGACAGGTGCTG 625
CCGGTGGTCCCGCGTGGAAGG 43
GGGGCCGTAGCTCAGCTGGGA 12177
TGGATGGGCTATGGGGACTCA 2143
AGGTCCCAAGGGTATGGCTGT 2493
CGAACCTGGGGAACTGAAACA 16442
CATGCCGCGGTGAATACGTTC 226
TAGGGCGGGACACGTGAAATC 16
ATATCGACGGGGTTGTTTGGC 122
CCATATCGACGGGGTTGTTTG 78
GGTTAGTGGAGACACTATCCT 33
CAGGCCAGTGGCTTTTGTGAA 21
ACCGGAGAAGAAGCCCCGGCT 496
AGCGTGGGGTCGGAGGTTCAA 1253
AACTTCGGAAGAAGCGTGACC 42
TTGTCTGCGGATGGTTCGAGA 13
TATGGGGACTCACCGTCTTAC 93
ACATTCCGCCTGGGGAGTACG 1956
AGACTCCCTCGCCGAAAGTCC 17
TCGTCGGCCCATGTGGGCCGC 21
AATCGGATCAACTGAAGAGTT 15
CCTAACACATGCAAGTCGAGC 19
AGTTGGGTTCAGAACGTCGTG 12
ATACGGACGGGATAACCGCTG 276
GCGAACCTGGGGAACTAAAAC 20
TTCCCCGCTGTCTCCAACGCA 458
CAGACGAGGCGCTGACACGGA 11
ATACGAAGGGGGCTAGCGTTG 29
GCGGCAACGCATGCAGCTTAC 31
GTACCCAGAGGAAAGGACATC 496
CGTAGCTCAGCTGGGAGAGCG 27
ATCCTAACCAAACTCCGAATA 62
GGTACTCCGGGGATAACAGGC 84
GGGGTTGTTTGGCACCTCGAT 193
GCGAACCTGGGGAACTGAAAC 34783
CAGGCGATGAAGGACGTGATA 100
ACGAGGGCGGCGGCGCCGGCA 41
CAACCCCGGAACTGCCTTTGA 48
GATGGGAACCACGTTAATATT 19
GCAGCGAAGCGGTTCCAGGAC 23
GATCTGTCCCTAGTACGAGAG 89

GTACCTGAAACCGGATGCATA 11
AGGCGGACTTTTAAGTCAGGG 24
CGAGTGTAGAGGTGAAATTCG 137
TGGGGACTCACCGTCTTACTG 121
GACCGATAGCGAACCAGTACC 139
AATGCACGCGTAACTTCGGAA 101
GAAGTGGGTGATAGTCCCGTA 90
TGAAACGCTCCAGCGCCAATG 169
AGGTGAAATTCGTAGATATTC 116
CCATTACTAACGCTGAGGTGC 10
GGTAGAGCACTGGATGGGCTA 17
CGTATCTCTGGTGGACCTGTT 75
CCCGCAACGAGCGCAACCCTC 116
GTCGCAAGACTAAAACTCAAA 15
GGGGCCGTAGCTCAGCTGGGG 14
GCGCCAATGGTACTTCGTCTC 53
CGAACCTGGGGAACTGAAACG 62
GTCGTCGGTTCGATCCCGTCC 311
GCCGTAGCTCAGCTGGGAGCG 12
TCCACGCCGTAAACGATGAAT 57
GATAGGCCGGGTGTGGAAGTG 60
AACGTCGTGAGACAGTTCGGT 320
TTGACGGGGGCCCGCACAAGC 190
GCCGCGTGAGTGATGAAGGCC 30
GTGATCCGGTGGTCCCGCGTG 10
CGGGAGGCAGCAGTGGGGAAT 15
GTTTGATCCTGGCTCAGAACG 587
TCACTGGTCTAAATAAGGGTC 38
GGAGGTTCAAGTCCTCCCAGG 41
TGTTTGGTTAGGCGGAAGAGA 33
ACGGTCGCAAGACTAAAACTC 10
GGACATCAAACGAGACTCCGC 344
CTTGGAGATAGCTGGTTCTCC 22
AGGCTGATGACCCCCAAGAGC 17
TAAGGGCATTTGGTGGATGCC 10
GCGGTGAATACGTTCCCGGGC 228
GTAGCTCAGCTGGGAGAGCAC 5940
AACCAAACTCCGAATACCGAG 14
AGTGGTTGACAGGTTGGTTTG 10
TCGTGACGACATGTGTAGGAA 18
GATGAATGTTAGCCGTCGGGG 7214
GGTAGGCTCAGAACGGTCGGA 35
TTGACAGGATCTGTCCCTAGT 306
TCGGAGGAACACCAGTGGCGG 31
AAGCAACGCGCAGAACCTTAC 28
GAAATTCGTAGATATTCGGAA 28
AAAGGACATCAAACGAGACTC 143
TAGCGAAATTCCTTGTCGGGG 11
TCGATCCCGTCCGGCTCCACC 1789
CCAAGTGATCTAGCCATGAGC 95
AGCGAACCTGGGGAACTGAGA 26
GCAGGTCCCAAGGGTATGGCT 2359
ATCGACGGGGTTGTTTGGCAC 282
CAACGCAGACTCAGTGAAATT 102

GGGTTGTTTGGCACCTCGATG 192
GCGCAGAACCTTACCAGCCCT 5954
TACGAAGGGGGCTAGCGTTGT 44
AGGCGACCACGGTAGGGTCAG 2019
TTGAAGGCAATATGGAAGTAG 19
GCTTTACACTGGCATTCGTGA 413
GGATGCCTTGGCATGCACAGG 27
ATGGTGGTGACAGTGGGCAGC 139
CCCTTGACATCCCGGTCGCGG 129
ATACCCTGGTAGTCCACGCTG 30
TAGCCATGAGCAGGTTGAAGG 39
AGGCTGATGACCCCCAAGAGT 4642
GTCGAGTGCAATGGCATAAGC 38
TGCATGAAGTTGGAATCGCTA 39
ACCCTGACCACCATCTAAGGT 30
GTACCTGAAACCGGATGCCTA 5119
GGGTTAAGTCCCGCAACGAGC 15
TCCCAAGGGTTTGGCTGTTCG 14
ACGGTCCTAAGGTAGCGAAAT 73
GGTGAATACGTTCCCGGGTCT 11
CCAGGATGTTGGCTTAGAAGC 1770
ATGGGGACTCACCGTCTTACT 89
GAATTTTGGACAATGGGCGCA 38
TGAGCAGGGTTAGCCGGCCAC 24
GGCGATGAAGGACGTGATACG 67
GCATGCACAGGCGATGAAGGA 432
CTGAGGTGCGAAAGCGTGGGG 87
TCCCGGGCCTTGTACACACCG 229
CATCTAAGTACCCAGAGGAAA 165
GGTGGGTAGTTTGACTGGGGC 74
CCAAGAGTCCATATCGACGGC 14
GGGTCAGCGACTTAGTGTATC 213
CGATAAGCGTCGGGGAGGTGC 45
TGAGATGTTGGGTTAAGTCCC 77
CTAAGCGGGAAACCCACCTGA 175
TCACTGGACCATTACTGACGA 26
GGATCTGTCCCTAGTACGAGA 100
GGAGAAGAAGCCCCGGCTAAC 94
GCCGTGAAACGCTCCAGCGGC 34
TGAGTAGGGCGGGACACGTGA 42
CTCAGTTCGGATTGCACTCTG 153
CGGATAAAAGGTACGCCGGGG 1721
GAAGGCCGCAGGTTCAAATCC 17
GCAGCAGTGGGGAATATTGGA 15
CGACGATCCATAGCTGGTCTG 397
TCGTGAGACAGTTCGGTCCCT 295
TCGGAGGAACACCAGTGGCGA 8594
AGTGATCAAGTGTCTTAAGGG 94
TAGGCGCAGCGAAAGCGAGTC 20
GAAATTCGTAGATATTCGGAG 174
CGGATAAAAGGTACGCTGGGG 13
GCGAACCTGGGGAACTGACAC 11
GTTGGGCACTCTAAGGGGACT 1347
GATAGTGAACCAGTACCGTGA 40

AGGTGAGTGGAATTCCGAGTG 62
CGTCGTGAGACAGTTCGGTCC 678
TGGGCTGGGCTACACACGTGC 13
GGTCGTCGGTTCGATCCCGTC 342
AAGCCGTCTCAGTTCGGATTG 26
ATCCTGGGGCTGGAGCAGGTC 512
TGGATGTTTGGTTAGGCGGAA 53
CAGCAGCCGCGGTAATACGAA 11
ACCGGGATGGACGTATCTCTC 40
ACGAAGGGGGCTAGCGTTGTT 528
TTGGGCACTCTAAGGTGACTG 25
ATCAAACTTGGAGATAGCTGG 56
GACCGAAGTGGGTGATAGTCC 255
AACGAACGCTGGCGGCAGGCA 11
GACCTGTTGTGGCGCCAGCCG 3858
TCCAGCCATGCCGCGTGAGTG 15
GGCGCAAGCCTGATCCAGCCA 306
TCTTGCAGCGAAGCGGTTCCA 132
CTTAGTGTATCGAGCAAGCTT 22
GGGTGGAGCAGCTCGGTAGCT 10
TTTGCGGACTTTTACGAAAGT 42
ACGGAAAGACCCCGTGAACCT 12
ACGCGAGTGTGAGCTAATCTC 10
GGCATTTGGTGGATGCCTTGG 43
GGTTAGACGGAAAGACCCCGT 36
CTCTAAGGGGACTGCCGGTGA 129
ATAATGGGTCAGCGACTTAGT 148
GTTGAAGGAACTCGGCAAAAT 95
ATTGCACTCTGCAACTCGAGT 349
TTGCGTCTCCGTGTTTTACAT 27
GGTTGTTTGGCACCTCGATGT 166
GTTCCCGGGCCTTGTACACAC 98
CCGGGGAGTACTAGTCGGCAG 797
AGCTGGTCTGAGAGGATGATC 56
AGTCGTGAGACATCCTGGAGG 27
TGGGGGTGCGACTGATTATAG 38
CTTCTCATGTTTGTGTTCTTC 23
AAGAACACCAGTGGCGAAGGC 14
ATAACTCAGGGAAACTTGTGC 65
GACCTGTTGTGGCGCCAGCCA 11
CCTTTGATACTGGAAGTCTTG 18
TGACTTGTGGCTAGGGGTGAA 1151
AAGTAGGGCAATAAGGCAATA 10
AGTTTGATCCTGGCTCAGAAC 503
CGTCGGTATCTGGGCTTGTAG 60
CCGGGGCTCAAGCCATACACC 3187
TGAGGGCTTTGGGCAAACATA 10
GTAACTATAACGGTCCTAAGG 10
TCATGGCCCTTACGGGCTGGG 116
CACTGGCATTCGTGACGACAT 211
TTATCGGCAAATGATCGGCCC 133
CGTCGTGAGACAGTTCGGTCT 17
TAGCTATATACGGACGGGATA 77
CGGTCATAGTGATCCGGTGGT 613

GCCCGCGTTGGATTAGCTAGT 93
GAGTGATCAAGTGTCTTAAGG 105
AGAGGTGAGTGGAATTCCGAG 112
CCCAAGGTTTGTCCTGGGTGA 43
ATGGACGTATCTCTGGTGGAC 733
GGCATGAAGTTGGAATCGCTA 14
CCGAACTCGGCCGTGAAACGA 13
GTCATCATAAATAAGGTATCT 10
CTTGTCGGGTAAGTTCCGACC 67
TGAAAAGCACCCCGACGAGGG 35
GATCCTGGCTCAGAACGAACC 13
ATAGTCCCGTACACGTAGAAT 206
GCCTGGGGAGTACGGTCGCAA 1190
CTATGGGGACTCACCGTCTTA 92
GCCAGCAGCCGCGGTAATACG 23
CAGTGGGGAATATTGGACAAT 20
AGAATTGGAAAGAGGCCGGAT 65
TGCGGGGTTCCTGCGGTTAGA 220
ACGTGAAATCCTGTCTGAACA 80
GGGAACTGAAACATCTAAGTA 181
GGTATCGGAAGTGAGAATGCT 17
CGAACCTGGGGAACTAAAACA 14
AGTAGCGTTTGCGTCGGTATC 14
CGCGATGGTAGGCTCAGAACC 23
CGCGGCCCATCAGGGCCGACG 33
CGGTCCCAAGGGTATGGCTGT 10
TCAGCTCGTGTCGTGAGATGT 295
GAGTACGGTCGCAAGATTAAA 111
CCCTGACCACCATCTAAGGTC 40
CGCGCAGAACCTTACCAGCCT 17
ATTCCGAACTCGGCCGTGAAA 999
CGACATGTGTAGGATAGGTGG 1086
CTGATCCAGCCATGCCGCGTG 41
CAGCGAAAGCGAGTCTGAACA 20
TAGGGGTTAGAAGCGAACCTG 326
AATCCTGTCTGAACATGGGTC 1104
AAAAGCCGTCTCAGTTCGGAT 25
CCTATCTGCCGTGGGTGTAGG 159
CAGTTGGAGCCCAAGGTTTGT 77
TGAAGGAACTCGGCAAAATGC 196
GTGCCACCCCCCTATGGTTGC 15
CACGTACTGGAGGACCGAACC 6978
TGGGGTCGGAGGTTCAAGTCC 656
TAGGTCGCTGCCAGGTCTGCA 13
ATTACTGACGCTGAGGGGCGA 35
GAGGGCGGCGGCGCCGGCAGC 40
TATACGGACGGGATAACCGCT 157
GGGGTCGGAGGTTCAAGTCCT 438
ATGAACTTTGGCGGACACGTT 12
ATGGGCTATGGGGACTCACCG 1236
CCGTCGGGGTGTTTACACTTC 126
AGGTAGAGCGTCGACCGAATA 340
CGGCAAATGATCGGCCCGCGT 2515
GGCCGTAGCTCAGCTGGGAAA 12

GAGGGCATGAAGTTGGAATCG 13
GTTCGGATTGCACTCTGCAAC 55
GCCTTAGGGTTGTAAAGCTCT 30
TACAGGTGCTGCATGGCTGTC 12
ACCAAAAACACAGGGCTCTGC 56
TGCGTCTCCGTGTTTTACATG 27
CTTAAGCCGGTAGGTGTAGGC 28
CGCGCAGAACCTTACCAGCCC 6852
CATTACTAACGCTGAGGTGCG 10
TGGAAGTAGGGCAATAAGGCA 28
TTGGAGCAACGCTGGATGGGT 25
TCGCGTGTGTTGTGAGGTCTT 75
AAGGGCATTTGGTGGATGCCT 18
GCTGTCTCCAACGCAGACTCA 40
CTGACCACCATCTAAGGTCCC 36
ACCCGAAGGCGCTGTGCTAAC 194
GCTTGATAAGCGTGGGGTCGG 41
CGACGATTTCCGAATGGGGAA 19
CACGTACTGGAGGACCGAACT 11
CAAGGGGAGCGGCAGACGGGT 20
AAGGGACAGTCGTGAGACATC 38
AAGGTAGCCGTAGGGGAACCT 14
GATGTCTAACTGCGGCCCGTT 57
TCGGCCGTGAAACGCTCCAGA 21
GGGGCCCGCACAAGCGGTGGA 208
GAATACCCCCGGGGGTAGAGC 51
AGTCTGCCTGTTCTGTATGAA 13
CGAGGGCATGAAGTTGGAATC 13
CTTAAGGGCATTTGGTGGATG 10
TCTGGTGGACCTGTTGTGGCG 629
CGCTTGAGAGAACTGCGTTGA 760
GCCATCGCTCAACGGATAAAA 80
GTTGCAATAGATCGGGATGAC 54
TGGTAGACTTTGAAGCAGGGG 262
TCCGGGTCCAGGACCGTGTAT 31
GAAAGGCGTAGTCGATGGGAA 207
AGATATTCGGAGGAACACCAG 1595
AACCTTACCAGCCCTTGACAT 119
GGGCCATCGTTCAACGGATAA 16
AGGTGGTGCATGGCTGTCGTC 11
ATAGTGATCCGGTGGTCCCGC 540
AATACGTTCCCGGGTCTTGTA 10
CTCATCGCATCCTGGGGCTGG 138
CAGGCGACCACGGTAGGGTCA 2026
ATGTCTAACTGCGGCCCGTTA 22
GTGCGGCTGGATCACCTCCTT 37
CGTAGATATTCGGAGGAACAA 11
GGGGTAGAGCACTGGATGGGC 77
TTGCACTCTGCAACTCGAGTG 191
CACGGCCCAGACTCCTACGGG 5126
GCATGAAGCTTACCGGTACTA 76
GTAGCTCAGTTGGTTAGAGCA 114
ATCCCAAAACAACCAGGATGT 53
TATAGACCGTACCCTAAACCG 1581

TTTACCCGAAGGCGCTGTGCT 320
GGATGTGAGGATCCCAAAACA 21
ACGTGCTGGGCTACACACGTG 11
CTCAGAACGGTCGGAAATCGT 514
GCCGGGTGTGGAAGTGCGGCA 438
CTTGGTGGGCCTGGGAGGACT 14
CCGTCTCAGTTCGGATTGCAC 10
GTGACGGATCGCGTGTGTTGT 40
TGGCATGCACAGGCGATGAAG 303
TGGGGAGTACGGTCGCAAGAT 43
CGCAAGGAGGCAGGCGACCAC 21
CGTATAGGGCCTGACGCCTGC 11
TCAACTGGAGAGTTTGATCCT 10
GGACCATTACTGACGCTGGGG 17
GTCCCGTACACGTAGAATAGC 431
TAACGGTCCTAAGGTAGCGAA 52
GCGGCCCATCAGGGCCGACGG 41
GCACGTAGGCGGACTTTTAAG 72
CCTTGACATCCCGGTCGCGGT 116
CAACTTGAGAGTTTGATCCTG 25
TGGTTGGATGTTTGGTTAGGC 33
AAAAGGTACCCCGGGGATAAC 75
TCTGTCCCTAGTACGAGAGGA 105
GATATTCGGAGGAACACCAGA 38
ATCGCTAGTAATCGCGGATCA 277
TGAGAGTTTGATCCTGGCTCA 769
TATCTGCCGTGGGTGTAGGAA 20
AATGAGAGTGATCAAGTGTCT 1593
AGCCCTTGACATCCCGGTCGC 86
CGACACTGGTGGACTGGTAGA 406
GCAATGGCATAAGCCTGCCTG 64
GTGGATGCACGTATGTGCGTG 344
TGCGGTTAGACGGAAAGACCC 50
ACTATAACGGTCCTAAGGTAG 12
AGACACGGCCCAGACTCCTAC 201
TTTGGCACCTCGATGTCGACT 93
GGGGAATATTGGACAATGGGC 391
GGGGAGTACTAGTCGGCAGAC 22
TACGGTAACACGTACTGGAGG 62
GGTAAAGGCTCACCAAGGCGA 33
ACGGGCTGGGCCACACACGTG 10
TATCGACGGGGTTGTTTGGCA 197
ACGGGAGGCAGCAGTGGGGAA 30
GCGGGGTGGAGCAGCTCGGTA 10
CGTAACGACTTCCCCGCTGTC 95
ACACGTAGAATAGCAGAAGTC 589
GACTGCAAGACTGACAAGTCG 47
GGCAACGCATGAAGCTTACCG 11
GGGTCGACCACGATCCAAGCC 941
CAGACTCCTACGGGAGGCAGC 591
CATTCCGCCTGGGGAGTACGG 2019
AGTGCAATGGCATAAGCCTGC 21
GGTCTGAGAGGATGATCAGCC 10
GATATTCGGAGGAACACCAGG 22

ACGTACTGGAGGACCGAACCG 12
GAATACCGGGGAGTACTAGTC 38
CGGCTGCACCCGATCCCATTC 14
ACGGAGGCGCGCGACGGTAGG 10
GGAAGATCGAGAATTGGAAAG 1097
GGAACACCAGTGGCGAAGGCG 563
TTACTGACGCTGAGGTGCGAA 7203
CGGTGAATACGTTCCCGGGCC 164
ATGTGCCCTTCGGGGGAAAGA 10
ACGCCGGGGATAACAGGCTGA 24
CACGATCCAAGCCTAAGTACT 41
TTCCGAATGGGGAAACCCACC 18
TTTGCAAGCAGGGGGTCGTCG 53
ACTTGAGAGTTTGATCCTGGC 2618
GTGAGTAACGCGTGGGAACGT 18
TTACACTGGCATTCGTGACGA 416
GAGGCGCGCTATGGTAGGCTC 24
GAGGCGCGCGATGGTAGGGTC 25
GAGAGGACCGGGATGGACGTA 1829
GGCGACGATCCATAGCTGGTC 68
GTTAGACGGAAAGACCCCGTG 76
ATGGGCTGGGCTACACACGTG 12
CGACTTGATCACTCCCATTTA 273
CAGAACCTTACCAGCCCTTGA 1031
CACGCCGTAAACGATGAATGT 135
GCCGTGAAACGCTCCAGCGCA 28
TAACACATGCAAGTCGAGCGG 13
TCACTGGACCATTACTGACCC 16
CTGCCTGACTGCAAGACTGAC 79
CTGGCATTCGTGACGACATGT 151
TTTAGATCGTCGGTTCTTTGA 17
GTCGGAAATCGTTCGTCGAGT 210
CCTAAGTACTCGTGCATGACC 345
CGGCTGGATCACCTCCTTTCT 167
TTGGTTTTACCCGAAGGCGCT 197
CACGGGCCGTACCGCAGCTGA 98
GATGGACGTATCTCTGGTGGA 723
ACGAGACTCCGCTAGTAGTGG 17
GTGATGAAGGCCCTAGGGTTG 64
CCACACTGGGACTGAGACACC 12
CTGCGTTGAAGGAACTCGGCC 23
ATCGGAAGTGAGAATGCTGAC 57
GAGCAGGTTGAAGGTACGGTA 531
GGGCTGGGCTACACACGTGAT 68
GTGTAGGATAGGTGGTAGACC 28
TATCGGAAGTGAGAATGCTGA 21
TGGTGGACTGGTAGAGAATAC 2436
GGGTGAAGTCGTAACAAGGTA 50
CGATCGACTTGATCACTCCCA 366
AAGGCGCTTGAGAGAACTGCG 47
GAACCTGGGGAACTGAAACAC 121
TTGGAAAGAGGCCGGATTTAT 10
ACCGCAAGGAGGCAGGCGACC 20
TGGAGATAGCTGGTTCTCCGC 20

ACCATTACTAACGCTGAGGTG 10
TTCGTCGAGTGCAATGGCATA 40
GATGTTTGGTTAGGCGGAAGA 46
TCCGAACTCGGCCGTGAAACC 46
TTTACACTGGCATTCGTGACG 409
CGGGATGGACGTATCTCTGGT 848
GTCCAGGACCGTGTATGGTGG 10
TGACTGCAAGACTGACAAGTC 72
GCAGGGTTAGCCGGCCCCTAA 1094
CGAAGGCGGCTCACTGGACCA 342
CCTGAAGGCCGCAGGTTCAAA 17
GAGCAGGGTTAGCTGGCCCCT 11
TGATGAGGGGCCGTAGCTCAG 139
ACACCGAAGCTGTGGATGCAC 276
CGTGCTGGGCTACACACGTGC 10
TATGGCTAAGTGGGAAAGGAT 131
GAATCGCTAGTAATCGCGGAT 1455
GTGTAGGATAGGTGGTAGACT 272
AACTCAAATGAATTGACGGGG 12
GTGACTGCGTACCTTTTGTAT 10
TGTTAGCCGTCGGGGTGTTTA 68
CAAGCAGGGGGTCGTCGGTTC 402
GTCAGGGGTGAAATCCCGGGG 36
TAACGTCCGTCGTGGAGAGGG 26
AGGCGCGCGATGGTAGGCTCC 58
ACTCGGCAAATTGCACGCGTA 10
TCTCAAGACGCGGGAGAGTAG 217
TGAATGTTAGCCGTCGGGGTC 10
TGGACGTATCTCTGGTGGACC 705
ATTTTGGACAATGGGCGCAAG 60
ACCCTTTGATCCGACGATTTC 1244
TAGCCGTCGGGGTGTTTACAC 199
TGTAATCGGATCAACTGAAGA 10
CTGATCCTAACCAAACTCCGA 103
GGTTCAGAACGTCGTGAGACA 120
GGTCCAGGACCGTGTATGGTG 35
GGAGTTGGTTTTACCCGAAGG 25
AACGCTGGCGGCAGGCTTAAC 1746
CAGCTTACCGGTACTAATAGC 47
GATAGGTGGTAGACTTTGAAG 42
CTGGGCCGTGAAACGCTCCAG 13
GGTTTTACCCGAAGGCGCTGT 130
TAGGTAGAGCGTCGACCGAAA 13
GAACCTGGGGAACTGAAACAT 7477
CAGTCAGCCTGACGATCGCTT 20
GCCTGTGAAGGGACAGTCGTG 21
CCAAACTCCGAATACCGGGGA 26
TACACTGGCATTCGTGACGAC 420
CGCTGTCTCCAACGCAGACTC 782
TTGCGGGGGCAGGATTTGAAC 39
GCAGGCGACCACGGTAGGGTC 2079
ACCGGGATGGACGTATCTCTA 13
GATGAGGGGCCGTAGCTCAGC 139
GGATGGAGCAGCCCGGTAGCT 15

ACATGCAAGTCGAGCGCCCCG 21
GCCCCAGTAAACGGCGGCCGT 27
AACGAACGCTGGCGGCAGGCC 15
GGAGAACTGAAACATCTAAGT 10
GGCAGACACACGGCGGGTGCT 201
CGAGTATTCCCTATCAGAGCC 13
AGCGCCAATGGTACTTCGTCT 43
AGCGTTTGCGTCGGTATCTGG 10
TGGTGGGGTAAAGGCTCACCA 26
TTGGAGCCCAAGGTTTGTCCT 117
GCTCGATCGACTTGATCACTC 75
CTGACTGCAAGACTGACAAGT 69
GTGAGGGAAAGGTGAAAAGAA 11
CTTCGTCTCAAGACGCGGGAG 33
AAAGGTACCCCGGGGATAACA 71
AGGACCGGGATGGACGTACCT 10
TACCCGAAGGCGCTGTGCTAA 182
CGGCCGTGAAACGCTCCAGAG 10
CTAGTAGTGGCGAGCGAACGC 58
GATCCTGGCTCAGAACGAACG 1365
GTAGCGAAATTCCTTGTCGGG 786
GGTGGTTATGGCGGAGCGGCT 2798
AAAGCACCCCGACGAGGGGAG 31
GTTGGGCACTCTAAGGTGACT 26
AATTGACGGGGGCCCGCACAA 22
ATAGCGAACCAGTACCGTGAG 100
ACGGATCGCGTGTGTTGTGAG 657
TGGGGTGAAGTCGTAACAAGG 34
GCCGTACCGCAGCTGACGCTG 24
CATCAGGGCCGACGGCCGGTC 39
CTGGGGAGTACGGTCGCAAGA 127
AACGAACGCTGGCGGCAGGCT 3235
ACCGGGATGGACGTATCTCTG 1235
AATCGCTAGTAATCGCGGATC 1398
GATAAGCGTCGGGGAGGTGCG 31
TAACTTCGTGCCAGCAGCCGC 20
CGCTGTGCTAACCGCAAGGAG 26
CCTGGGGCTGGAGCAGGTCCC 13
AGGGCATGAAGTTGGAATCGC 13
AAGGCATCTAAGCGGGAAACC 27
CCCGCGTGGAAGGGCCATCGC 10
GTGAGATGTTGGGTTAAGTCC 92
GCCTGGGGAGTACGGTCGCAC 19
CTTGGGGGTCATCAGCCTGTT 13
AGAGAATACCAAGGCGCTTGA 66
GGCCCTAGGGTTGTAAAGCTC 7146
TTTATCGGCAAATGATCGGCC 79
GATCCTGGCTCAGAACGAACA 19
GCACAGGCGATGAAGGACGTG 239
AGTTAGGCTGGACCGGAGACA 27
AATCTGAGCAGGGTTAGCCGG 215
CCGAACTCGGCCGTGAAACGC 8123
GCGACCACGGTAGGGTCAGCG 2125
CGACTGTTTATCAAAAACACA 12

TACCGTATGTGCCCTTCGGGG 13
TAGGTGGTAGACTTTGAAGCA 77
CGGATAAAAGGTACTCCGGGG 43
GGTAGAGCGTCGACCGAATAC 466
GCGCTTGAGAGAACTGCGTTG 761
GGCTAACTTCGTGCCAGCAGC 12
GAGGCCGAAAGGCGTAGTCGA 13
AAGCGTGACCTCACTATGGGC 224
TCGGAGGTTCAAGTCCTCCCA 42
GGGGTCGTCGGTTCGATCCCG 333
CACGTACTGGAGGACCGAACA 34
GTTGTAAAGCTCTTTCACCGG 392
ACGAGGGGAGTGAAACAGTAC 18
CTGACGAGCTACCGGGCTGCT 23
CGGCCCGCGTTGGATTAGCTA 256
ACTCCTACGGGAGGCAGCAGT 91
GGGCTCTGCGAAGTCGCAAGA 803
AAGGATGTGAGGATCCCAAAA 12
CATTCCGAACTCGGCCGTGAA 42
TTCTAGTCATCATAAATAAGG 422
CACTGGATGGGCTATGGGGAC 1247
ACACAGGTGCTGCATGGCTGT 38
TCCAAGCCTAAGTACTCGTGC 47
GGAGGCGCGCGATGGGAGGCT 10
TAGGTCGCTGCCAGGTCTGCC 32
GGCAAAGAACAGGCGCAGCCC 10
ACGAGCTGGGCTACACACGTG 10
TGGGGTCGGAGGTTCAAGTCA 23
CGTTGTTCGGATTTACTGGGC 344
AGTGAAATTGAATTCCCCGTG 34
GACGGGGTTGTTTGGCACCTC 496
GGGGCTAGCGTTGTTCGGATT 790
TTGGCATGCACAGGCGATGAA 297
AGTGGCGAGCGAACGCGGACC 114
TGGATGCCTTGGCATGCACAG 18
CCCGGGCCTTGTACACACCGC 257
AGCGAAGCGGTTCCAGGAAAT 15
TGAGAGGATGATCAGTCACAC 13
CACGTACTGGAGGACCGAACG 25
TCCTGCGGTTAGACGGAAAGA 32
CACCGTCTTACTGATCCTAAC 147
CTGTCTCCAACGCAGACTCAG 34
CCATACACCGAAGCTGTGGAT 31
GCTGGGCTACACACGTGCTAC 12948
AACCAGTACCGTGAGGGAAAG 78
CGCGCAGAACCTTACCAGCCA 10
TAAGCGGGAAACCCACCTGAA 163
TCGGCAAATGATCGGCCCGCG 2529
GTTTTACCCGAAGGCGCTGTG 184
CGCGATGGTAGGCTCAGAACG 776
GGAGCAGCCCGGTAGCTCGTC 252
GTACGTGAGTTGGGTTCAGAA 91
TCCTGTCTGAACATGGGTCGA 2104
ATAAGCGTCGGGGAGGTGCGA 316

TCGGCCGTGAAACGCTCCAGC 36830
ACCACCACGTTGATAGGCCGG 157
TGAGAGTGATCAAGTGTCTTA 558
TTTAGAACGTCGTGAGACAGT 10
GTGGGCAGCGAGCACGCGAGT 243
CTCAAATGAATTGACGGGGGC 16
ATACGGCCCAGACTCCTACGG 18
CAAACGAGACTCCGCTAGTAG 25
CAGGGGGTAGCGACTGTTTAC 1931
TTCGGGGTGGATCTGTGGATC 40
GGGGTCGGAGGTTCAAGTCCA 12
GCAACGCGCAGAACCTTACCA 36
CGGAGGCGCGCGATGGTAGAC 20
GTAACTTCGGAAGAAGCGTGA 11
AGCCGAGAGGAAGGTGGGGAT 39
CGTAGATATTCGGAGGAACAC 7033
CCGCGTGAGTGATGAAGGCCC 22
GATCGTCGGTTCTTTGAAAAC 16
CACCAAGGCGACGATCCATAG 344
AGCAAGCTTAAGCCGGTAGGT 108
CTTACGGGCTGGGCTACACAC 866
CTCCAGCGCCAATGGTACTTC 19
CGTCAGCTCGTGTCGTGAGAT 263
CTGAGAGGATGATCAGCCACA 131
ATGGGCGCAAGCCTGATCCAG 129
CTCAGTGAAATTGAATTCCCC 44
GTTGTGGCGCCAGCCGCATAG 108
ACCAATAAGGTTAGCGGGCTT 59
GTACGCCGGGGATAACAGGCT 100
GATATTCGGAGGAACACCAGC 23
ACGTACTGGAGGACCGAACCC 6255
ACAGGTGCTGCATGGCTGTCG 703
GGAGATAGCTGGTTCTCCGCG 25
CTGAGAGGATGATCAGTCACA 12
GGTGCTGCATGGCTGTCGTCA 858
GAGATCGAACTGTCACAATGA 10
CAGACTCCTACGGGAGGCAGG 15
GACCGGGATGGACGTATCTCT 4826
CCCTAGGGTTGTAAAGCTCTC 20
TCGTAGATATTCGGAAGAACA 44
GCAGACTCAGTGAAATTGAAT 94
CTGCCTTTGATACTGGAAGTC 28
CCGGCTAACTTCGTGCCAGCA 20
TAACACATGCAAGTCGAGCGC 17
CCACGGTAGGGTCAGCGACTG 2102
GCCGTGAAACGCTCCAGCGCT 11
TGGTTGTCGTCAGCTCGTGTC 22
TAGCGTTGTTCGGATTTACTG 19
GAGTTGGTTTTACCCGAAGGC 32
AAGAAGCCCCGGCTAACTTCG 27
CGTAGAATAGCAGAAGTCCTT 1449
AGCGAACCTGGGGAACTGACA 20
TCGGGTAAGTTCCGACCTGCA 21
TAATCGGATCAACTGAAGAGT 18

CAAGGTTTGTCCTGGGTGACA 33
AGGTACCCCGGGGATAACAGG 65
GATATTCGGAGGAACACCAGT 1532
CCAAGCCTAAGTACTCGTGCA 115
ACTGTTTAGCAAAAACACAGG 13
CCCTAGGGTTGTAAAGCTCTT 2989
ACTAGTCGGCAGACACACGGC 193
TTACCCGAAGGCGCTGTGCTA 225
AGGGTCAGCGACTGGGGTGAA 350
CGTGGGCCTGCAGGTGGTGAC 513
AGCTCTTTCACCGGTGAAGAT 31
AACAGTTGGAGCCCAAGGTTT 15
TCGCTGCCAGGTCTGCAAAGC 10
GTAACAATAACGGTCCTAAGG 13
ATACCCCCGGGGGTAGAGCAC 358
AGCGACTGTTTAGCAAAAACA 12
GATGAAGGACGTGATACGCTG 41
GTCAGCCTGACGATCGCTTGC 18
GAGAGGACCGGGATGGACGTC 12
TAGAGAATACCAAGGCGCTTG 61
GCAGTTTGACTGGGGCGGTCG 21
GAGGATCCCAAAACAACCAGG 54
ATTCGTAGATATTCGGAGGAA 1483
GATTAAAACTCAAAGGAATTG 67
AGACTTTGAAGCAGGGGCGCC 931
CCTACGGGAGGCAGCAGTGGG 55
GAAGAGAAGATGTAATCGGAT 18
GGTTCAAGTCCTCCCAGGCCC 35
CACACCGCCCGTCACACCATG 75
AGGCTGGACCGGAGACAGGTG 636
TCAGAGCCGTGGAAGACCACC 65
GCCGTGAAACGCTCCAGCGCC 33401
GGTGAAATTCGTAGATATTCG 192
GCATGACCGATAGCGAACCAG 136
AAGTGATCTAGCCATGAGCAG 103
CGACGTATAGGGTCTGACGCC 740
TCCTGGGGCTGGAGCAGGTCC 484
CTGCGTTGAAGGAACTCGGCA 1173
TCCAGACTCCTACGGGAGGCA 14
TTAGATCGTCGGTTCTTTGAA 16
AGCTCGTCAGGCTCATAACCT 36
GGCTTGTAGCTCAGTTGGTTA 117
TGAATGTTAGCCGTCGGGGTG 2977
TCGGGGTGGATCTGTGGATCG 13
CCCAAAACAACCAGGATGTTG 99
CGCTAGTAGTGGCGAGCGAAC 55
AGGCGCGCGATGGTAGGCTCG 92
TATTCGGAGGAACACCAGTGG 1242
TTTGAAGCAGGGGCGCCAGCC 547
GGCGGCTCACTGGACCATTAC 1136
CAAGCAGGGGGTCGTCGGTTG 12
GACTGCCGGTGATAAGCCGAG 1100
GGGGCTCAACCCCGGAACTGC 14
AGTGTAGAGGTGAAATTCGTA 84

GTGTAGGATAGGTGGTAGACA 72
GAGACAGTTCGGTCCCTATCT 153
TACACCGAAGCTGTGGATGCA 174
ATTGACGGGGGCCCGCACAAG 143
GAGGTTGGCTTAGAAGCAGCC 40
GCCGGCCCCTAAGGCGAGGCC 16
GCATTAAACATTCCGCCTGGG 626
TGAGTAACGCGTGGGAACGTA 16
GGTGGTAGACTTTGAAGCAGG 135
TTTCCGAATGGGGAAACCCAC 19
TGGTTATGGCGGAGCGGCTGC 937
CGGAAGAAGCGTGACCTCACT 450
AAGATCGAGAATTGGAAAGAG 902
CGTCGAGTGCAATGGCATAAG 35
CCGCAACGAGCGCAACCCTCG 114
AGGGGAGTGAAACAGTACCTG 18
CCACGTTAATATTCGTGGGCC 10
AGGCGGCTCACTGGACCATTA 629
AAGCCTAAGTACTCGTGCATG 133
GAACCTGGGGAACTGAAACAA 111
GACTGACAAGTCGAGCAGAGA 23
AGGATCTGTCCCTAGTACGAG 97
TAGGTAGAGCGTCGACCGAAT 284
TAACAATAACGGTCCTAAGGT 20
AACAACCAGGATGTTGGCTTA 16
CGAACCTGGGGAACTGAAAGA 14
CCCCGTGCACCTTTACTATAG 13
ATAGCAGAAGTCCTTGAGTAG 79
TAAGCCTGTGAAGGGACAGTC 13
GGGCTGGGTTACACACGTGCT 10
AGTCGATGGGAACCACGTTAA 21
GGGGTAGCGACTGTTTACCAA 368
ACTTGGCCGTGAAACGCTCCA 13
CAAGCAGGGGGTCGTCGGTTA 13
TGGCTAAGTGGGAAAGGATGT 118
GACTGCCGGTGATAAGCCGAA 18
CTGGTAGTCCACGCTGTAAAC 22
AGGGAGTGAGAGACTCCCTCG 41
AGGGGGCTAGCGTTGTTCGGA 1009
GCCTACAAACAGTTGGAGCCC 17
GTGTAGGATAGGTGGTAGACG 46
GAGACACGGCCCAGACTCCTA 182
CTGCGTTGAAGGAACTCGGCG 23
GGTAGTCCACGCCGTAAACGA 77
TAGCTGGTCTGAGAGGATGAT 80
CCACACTGGGACTGAGACACG 630
CATCCCGGTCGCGGTTAGTGG 55
AGTACCTGAAACCGGATGCAT 16
CAGGATCTGTCCCTAGTACGA 114
TGTCTGAACATGGGTCGACCA 322
AATAGCTCCTGCATATAGACC 37
GAAAGCGAGTCTGAACAGGGC 38
TGAATGTTAGCCGTCGGGGTA 31
CAAAGGAATTGACGGGGACCC 14

CTAGTTGGTGGGGTAAAGGCT 43
AGGCGCGCGATGGTAGGCTCA 10960
CCACCAGGTCGGCGAAGAACA 25
GGGAGTGAGAGACTCCCTCGC 41
TCGGAAGTGAGAATGCTGACA 127
ACTCGGCCGTAAAACGCTCCA 11
GCAACCCTCGCCCTTAGTTGC 10
GTTCGGTCCCTATCTGCCGTG 1036
TCCGAACTCGGCCGTGAAACG 7625
CGTCGACCGAATACCCCCGGG 5360
AGGTGCTGCATGGCTGTCGTC 780
ACCTGACTTTGGTTTTCGGAT 78
ACCCCCGGGGGTAGAGCACTG 447
CCGCCTGGGGAGTACGGTCGC 3609
TAGGCTGGACCGGAGACAGGT 700
ACTGAGACACGGCCCAGACTC 666
AGTTGGTGGGGTAAAGGCTCA 39
GTCCAGACTCCTACGGGAGGC 12
GAACCTGGGGAACTGAAACAG 14
TTTGGCACCTCGATGTCGGCT 13
GCATTCGTGACGACATGTGTA 33
TCGCTCAACGGATAAAAGGTA 168
TGTATGGTGGGTAGTTTGACT 14
CGACTCATCGCATCCTGGGGC 508
AACTTGGAGATAGCTGGTTCT 173
GCTCACTGGACCATTACTGAG 27
GTGCATGAAGTTGGAATCGCT 39
ATCCATAGCTGGTCTGAGAGG 21
CGGTACTAATAGCTCGATCGC 11
AACAGGCTGATGACCCCCAAG 392
ACGAAGGCGCGCGATGGTAGG 13
GTTGCGGGGGCAGGATTTGAA 35
GTAATCGCGGATCAGCATGTC 20
AAATGTACCGGGGCTCAAGCC 131
GGACCATTACTGACGCTGAGG 17878
GGGCCTGCAGGTGGTGACGGA 140
TAGTCGGCAGACACACGGCGG 188
GCTACACACGTGCTACAATGG 41
CTCGAGAAGCTGGTCTTTCTG 311
ACGAACGCTGGCGGCAGGCTC 16
CAAGCCTAAGTACTCGTGCAT 116
GCCGTCGGGGTGTTTACACTT 165
AGACTCCTACGGGAGGCAGCA 208
TTAGGGTTGTAAAGCTCTTTC 13
GAATATTGGACAATGGGCGCA 532
ATAGGGTGTGACGCCTGCCCG 11
CGGAATCGCTAGTAATCGCGG 81
AGGCAGGCGACCACGGTAGGG 150
CGAAGGCGCTGTGCTAACCGC 55
AAAATGTACCGGGGCTCAAGC 13
ACTCATCGCATCCTGGGGCTG 204
CCTGCGGTTAGACGGAAAGAC 55
GGTGGACTGGTAGAGAATACC 2304
GGATGGGCTATGGGGACTCAC 2504

CGGCCGTGAAACGCTCCGGCG 10
GATAAGCGTGGGGTCGGAGGT 54
CATCGCTCAACGGATAAAAGG 90
AGGTAGCGAAATTCCTTGTCG 372
GTCTCCAACGCAGACTCAGTG 22
CATAAGCCTGCCTGACTGCAA 187
CGACTTCCCCGCTGTCTCCAA 205
CCTTGTCGGGTAAGTTCCGAA 20
TCCCTAGTACGAGAGGACCGG 36
GAGGCGCGCGATGGTAGGATC 47
AACCTGGGGAACTGAAACATA 61
GCTCACTGGACCATTACTGAA 199
AAGTCTTGAGTATGGTAGAGG 29
CACCAGGTCGGCGAAGAACAC 25
CGGGCCGTACCGCAGCTGACG 116
AGAAGCGTGACCTCACTATGG 142
TGAAACATCTAAGTACCCAGA 54
CTTTCTAGTCATCATAAATAA 50
ATGACGGTAACCGGAGAAGAA 67
TAACGATAAAGGGAGTGAGAG 112
TTGGGACTGAGACACGGCCCA 27
CTGTGCTAACCGCAAGGAGGC 27
GGACAATGGGCGCAAGCCTGA 525
ATAAGCCTGCCTGACTGCAAG 219
CTAGTCGGCAGACACACGGCG 194
ACACTGGGACTGAGACACGGC 1073
AGGAAGATCGAGAATTGGAAA 34
TAGGGTCAGCGACTGGGGTGA 272
AGGTTTGTCCTGGGTGACAGC 32
CATTACTGACGCTGAGGTGCC 13
CACCAACTTCGATCCGAAAAC 42
AGACGAGGCGCTGACACGGAT 56
GGACCATTACTGACGCTGAGA 13
TAAGGTAGCGAAATTCCTTGT 151
TGGCTCAGGACGAACGCTGGC 23
ACGAACGCTGGCGGCAGGCTT 5598
GAGTAGTTGGGGGTGGTTTTC 11
CGCTAGTAATCGCGGATCAGC 86
TCCCATTCCGAACTCGGCCGT 131
AAGTCGAGCAGAGACGAAAGT 34
GCAGCACTCGACGGGCGTGAG 11
GTGAAAAGCACCCCGACGAGG 35
AAGCCATACACCGAAGCTGTG 29
GGGGACTGCCGGTGATAAGCC 3320
GCGTCGACCGAATACCCCCGT 11
TTTGACTGGGGCGGTCTCCTC 28
GAAGGCGCTGTGCTAACCGCA 19
AATATTCGTGGGCCTGCAGGT 74
GCTGGTTCTCCGCGAAATCTA 12
AAGGGGAGCGGCAGACGGGTG 12
TGGGCGGCATTGTCTGCGGAT 5660
GGACCATTACTGACGCTGCGG 12
CGGTCGCAAGACTAAAACTCA 10
ACTGCCTTTGATACTGGAAGT 38

AGCGAACCTGGAGAACTGAAA 13
CACGGATTTGACCTTCGGGTT 29
CGACGGGGTTGTTTGGCACCT 563
TCAGTTGGGCACTCTAAGGGG 1596
TGGAATTCCGAGTGTAGAGGT 526
GTAACACGTACTGGAGGACCG 146
TTGTGGCTAGGGGTGAAAGGC 821
ATGCACGCGTAACTTCGGAAG 181
GGTTCGATCCCGTCCGGCTCC 657
GGGTAGTTTGACTGGGGCGGT 127
CGAGAGGAAGGTGGGGATGAC 103
GCAAGCTTAAGCCGGTAGGTG 82
CCGTAGCTCAGCTGGGAGAGG 13
TAAGCCGGTAGGTGTAGGCGC 170
AATCGCGGATCAGCATGCCGC 28
GAGAGTTTGATCCTGGCTCAG 469
CTTGTGGCTAGGGGTGAAAGG 938
AACGATGAATGTTAGCCGTCG 2993
TAAAAGGTACCCCGGGGATAA 72
CGTGAAGATGCGGGGTTCCTG 56
CAAAAACACAGGGCTCTGCGA 64
GATGACTTGTGGCTAGGGGTC 11
CTACAATGGTGGTGACAGTGG 11
AGCGTCGGGGAGGTGCGAATA 520
GCCCCGCAAGGGGAGCGGCAG 67
GGAGGCGCGCGATGGTAGGGT 26
GTAGACTTTGAAGCAGGGGCG 787
GGCTCATCGCATCCTGGGGCT 13
GGCTGGAGCAGGTCCCAAGGG 16
CCTGGTGGTTATGGCGGAGCG 3147
GCAATAGATCGGGATGACTTG 241
CGGAAGAACACCAGTGGCGAA 42
ATACCAAGGCGCTTGAGAGAA 90
AGGGGTTAGAAGCGAACCTGG 693
CGCTTGATAAGCGTGGGGTCG 28
ACACACCGCCCGTCACACCAT 74
ACAGTTGGAGCCCAAGGTTTG 80
GTGGAATTCCGAGTGTAGAGG 540
GGAATATTGACAGGATCTGTC 185
TGGGCGGCATTGTCTGCGGAC 11
AAACCAATAAGGTTAGCGGGC 63
TGTTTGGCACCTCGATGTCGA 150
AAGTCTGCCTGTTCTGTATGA 11
AGCCCCGGCTAACTTCGTGCC 160
CTGACGCCTGCCCGGTGCTGG 786
GCAACCCACCTTAGATGACTA 23
CTGGCTCAGGACGAACGCTGG 20
GTACGAGAGGACCGGGATGGA 285
CGATGCATTAGACCCGAAACC 24
ACACGTACTGGAGGACCGAAC 7952
TGTATCTCGAGAAGCTGGTCT 19
ACCACGTTGATAGGCCGGGTG 131
TACACGTAGAATAGCAGAAGT 84
GAGAATACCAAGGCGCTTGAG 70

ACCACCAGGTCGGCGAAGAAC 41
TAGTAATCGCGGATCAGCACG 11
GCCTGATCCAGCCATGCCGCG 124
CCTTACCAGCCCTTGACATCC 111
AGTCGGAATCGCTAGTAATCG 245
CCGTAGCTCAGCTGGGAGAGA 34
CCAGGCCAGTGGCTTTTGTGA 56
CCGTAAGCCTGTGAAGGGACA 19
GAATAGCAGAAGTCCTTGAGT 253
GACGACGTATAGGGCCTGACG 33
GTCGAGCGCCCCGCAAGGGGA 61
GAGGCGCGCGATGGTAGGCTC 35083
GAATAACTCAGGGAAACTTGT 85
GGTTGGATGTTTGGTTAGGCG 27
GGGATGACTTGTGGCTAGGGC 18
ACGAATGGCGTAACGACTTCC 11
TACCCCCGGGGGTAGAGCACT 395
GAGGACCGGGATGGACGTATC 9471
GTAGATATTCGGAGGAACACT 26
ACTGAGATACGGCCCAGACTC 12
GTGCGGCAACGCATGCAGCTT 21
GTTCCGACCTGCACGAATGGC 17
AGTCGGCAGACACACGGCGGG 186
AGGTCCCAAGGGTTTGGCTGT 11
GTACACACCGCCCGTCACACC 23
TCCCAAGGGTATGGCTGTTCG 2366
GTGCTGCATGGCTGTCGTCAG 778
AAGACGCGGGAGAGTAGGTCG 393
TAAGGCGAGGCCGAAAGGCGT 10
TGCGTACGGCGCGTGAGCGAG 16
TCGTGTCGTGAGATGTTGGGT 192
TGTTGGGTTAAGTCCCGCAAC 23
GCTGGACCGGAGACAGGTGCT 1376
GTGGGTAGTTTGACTGGGGCG 106
GGTGGGGAGTTTGACTGGGGC 148
CTCACCGTCTTACTGATCCTA 170
TACGGGAGGCAGCAGTGGGGA 32
GGGATAACCGCTGAAGGCATC 217
CGAACGCTGGCGGCAGGCTTG 52
TGCTGCATGGCTGTCGTCAGC 806
CACTCTAAGGGGACTGCCGGT 240
GGTACGTGAGTTGGGTTCAGA 89
CCAGCCCTTGACATCCCGGTC 93
CGTCTTACTGATCCTAACCAA 19
GACGATTTCCGAATGGGGAAA 19
CCCTAGTACGAGAGGACCGGG 27
CCAAGGCGCTTGAGAGAACTC 58
TGAGGGGCCGTAGCTCAGCTG 138
AGCGAACCTGGGGAACTAAAA 13
CTGGTTGGATGTTTGGTTAGG 33
CATGCAAGTCGAGCGCCCCGC 21
CGGAAGTGAGAATGCTGACAT 105
GTGGACTGGTAGAGAATACCA 2023
GTAAACGGCGGCCGTAACAAT 10

GGCCGAAAGGCGTAGTCGATG 56
CTATCAGAGCCGTGGAAGACC 25
TCCCAAGGGTATGGCTGTTCA 13
ATGGGTCAGCGACTTAGTGTA 204
CTTGTAGCTCAGTTGGTTAGA 123
CTTGATGAGGGGCCGTAGCTC 152
TCCCTAAGTTATGGCTAAGTG 21
CCGTCTTACTGATCCTAACCA 26
CAATGAACTTTGGCGGACACG 16
GAGGACCGGGATGGACGTATT 11
AGGGCCATCGCTCAACGGATA 360
AGGGCGTTCAGTTCGATGCAT 316
GTAGATATTCGGAGGAACACC 6894
CCGGAGAAGAAGCCCCGGCTA 427
TACCAGCCCTTGACATCCCGG 57
GCAACGCATGCAGCTTACCGG 33
CTAAGGTAGCGAAATTCCTTG 116
AGGACCGGGATGGACGTATCA 12
ATTCGTGACGACATGTGTAGG 279
ACGGAGGCGCGCTATGGTAGG 24
GATTTACTGGGCGTAAAGCGC 2551
GAATTGGAAAGAGGCCGGATT 31
CCAGAGGAAAGGACATCAAAC 22
CCCTGGTAGTCCACGCTGTAA 26
CGTTCGTCGAGTGCAATGGCA 41
TAAACGATGAATGTTAGCCGT 185
GGCAACAACCCTGACCACCAT 24
GCTGGACCGGAGACAGGTGCC 12
ATGCGTACGGCGCGTGAGCGA 16
CCCTGGTAGTCCACGCCGTAA 240
TTCGTGGGCCTGCAGGTGGTG 444
CCGAATACCCCCGGGGGTAGA 223
AGTTTGATCCTGGCTCAGGAC 17
CGAACGCTGGCGGCAGGCTTA 2437
CATCCTGGGGCTGGAGCAGGA 20
GGTGCATGGCTGTCGTCAGCT 16
CGAAGCTGTGGATGCACGTAT 54
CGGTAATACGAAGGGGGCTAG 129
TGAAATCCTGTCTGAACATGG 20
GACGTATAGGGTCTGACGCCT 667
CATCGAACTGAACGCCCTGTT 26
GCGCAAGCCTGATCCAGCCAT 252
GGCACCTCGATGTCGACTCAT 10
GCTATGGGGACTCACCGTCTT 265
TAGCTCAGTTGGTTAGAGCAC 114
GACCATTACTGACGCTGCGGT 12
ATTTATGATGACTAGAAAGCT 13
TAAAGGCTCACCAAGGCGACG 18
CATCCTGGAGGTATCGGAAGT 43
AGGGCATTTGGTGGATGCCTT 29
GACGATTTCCGAATGGGGCAA 17
AGCCGTAGGGGAACCTGCGGC 30
CGGACCAGGCCAGTGGCTTTT 725
CAGCCCGGTAGCTCGTCAGGC 486

CGGTATCTGGGCTTGTAGCTC 66
ACCGGATGCCTACAAACAGTT 18
CGGTGCTGGAAGGTTAAGAGG 13
ATTTGTGAGTAGTTGGGGGTG 15
GCTGGCGGCAGGCTTAACACT 26
GCAATGAGAGTGATCAAGTGT 192
CTAAGGTCCCTAAGTTATGGC 526
TTGTAGCTCAGTTGGTTAGAG 125
GACTCCTACGGGAGGCAGCAG 125
GCGACGATCCATAGCTGGTCT 399
GCGACTGTTTAGCAAAAACAC 13
CTGCGAAGTCGCAAGACGACG 217
GGTACGGTAACACGTACTGGA 32
CACAGACCAGGGGGTAGCGAC 43
TAACTCAGGGAAACTTGTGCT 30
TTCGGATTTACTGGGCGTAAA 577
GCCTGCCCGGTGCTGGAAGGT 40
ACGGGTGAGTAACGCGTGGGA 19
GTCCCAAGGGTATGGCTGTTC 2487
AGAACGTCGTGAGACAGTTCG 268
CTCAGGACGAACGCTGGCGGC 23
GAGTTTGATCCTGGCTCAGGA 14
CCCATCAGGGCCGACGGCCGG 40
CGGCGGCGCCGGCAGCGGCAG 22
CGGGGGTAGAGCACTGGATGG 159
TGGGGAACTGAAACATCTAAC 18
TGGACCATTACTGACGCTGCG 12
GTCTGAGAGGATGATCAGCCA 57
AACACCAGTGGCGAAGGCGGC 921
GGCTCTGCGAAGTCGCAAGAC 753
GAGCACTGGATGGGCTATGGG 51
CTGGTGGTTATGGCGGAGCGG 3036
TGAGAATGCTGACATGAGTAA 144
ACCAGACTCCTACGGGAGGCA 14
CCTGCACGAATGGCGTAACGA 83
GAAACGCTCCAGCGCCAATGG 63
AACCAATAAGGTTAGCGGGCT 76
AGCGGCTGCACCCGATCCCAT 29
CTCTGCAACTCGAGTGCATGA 17
CGGGTAGTTTGACTGGGGCGG 14
CGGGGAGGTGCGAATACCCTT 11
CGACTGGGGTGAAGTCGTAAC 12
GCTGGCGGCAGGCTTAACACC 20
ATACACCGAAGCTGTGGATGC 168
ACGGCGGCCGTAACAATAACG 18
GGGCTGGGCTGCACACGTGCT 10
CACCTGAAAACGAGTATTCCC 10
TGAAGATGCGGGGTTCCTGCG 48
TAGAAGCAGCCATCATTTAAA 10
TACCGGGGCTCAAGCCATACA 1056
ACCATTACTGACGCTGAGGCG 30
TACGAAAGTCTGCCTGTTCTG 12
TTCCGTAAGCCTGTGAAGGGA 26
CGGTACTAATAGCTCGATCGA 5449

CTAAGGCGAGGCCGAAAGGCG 11
TGCGATAAGCGTCGGGGAGGT 37
GCTCACTGGACCATTACTGAT 23
CCCCGCTGTCTCCAACGCAGA 775
AACCTGGGGAACTGAAACATT 19
CCTTACGGGCTGGGCTACACA 477
ACCTTTTGTATAATGGGTCAG 24
GTCCCTAAGTTATGGCTAAGT 27
ACCCAGAGGAAAGGACATCAA 14
AGTTCCGACCTGCACGAATGG 17
TAAGGTCCCTAAGTTATGGCT 442
TCAGGATGTTGGCTTAGAAGC 14
CGCAAGATTAAAACTCAAAGG 177
AGGGGCCGTAGCTCAGCTGGG 156
GGATCCCAAAACAACCAGGAT 65
AGACTCAGTGAAATTGAATTC 69
TTACCAAAAACACAGGGCTCT 16
CTGAACATGGGTCGACCACGA 1433
GTGATCTAGCCATGAGCAGGT 102
AGCTATATACGGACGGGATAA 70
CCTGATCCAGCCATGCCGCGT 64
CATTACTGACGCTGAGGTGCG 12668
GGTGGACTGGTAGAGAATACA 22
GCTAGCGTTGTTCGGATTTAC 161
CGGTACGTGAGTTGGGTTCAG 88
CCTGGGGAACTGAAACATCAA 10
AGGAGGTTGGCTTAGAAGCAG 62
GGGCTGGGCTATACACGTGCT 19
CGTCTCAAGACGCGGGAGAGT 157
TGGAAGGGCCATCGCTCAACG 47
GGGTCTGACGCCTGCCCGGTG 1409
TCGTGCATGACCGATAGCGAA 309
CCTAACCAAACTCCGAATACC 88
GGCCATCGCTCAACGGATAAA 93
CCTTGTCGGGTAAGTTCCGAC 66
TTTCCGAATGGGGCAACCCAC 12
ATAACAGGCTGATGACCCCCA 304
TTCCCCGTGAAGATGCGGGGT 23
TGATGACCCCCAAGAGTCCAT 97
CTAACGCATTAAACATTCCGC 137
AAAGCCGTCTCAGTTCGGATT 24
GTCATAGTGATCCGGTGGTCC 600
ACCGATAGCGAACCAGTACCG 156
GAGCGTCGACCGAATACCCCC 4468
GCCTGCAGGTGGTGACGGATC 238
CCCCGGGGGTAGAGCACTGGA 365
GAGTCCATATCGACGGGGTTG 83
GCTCACTGGACCATTACTGAC 5024
AACCTGGGGAACTGAAACATC 5529
GTCGTCGGCCCATGTGGGCCG 21
ATAACCGCTGAAGGCATCTAA 15
TGACCGATAGCGAACCAGTAC 134
CACACGCTTGATAAGCGTGGG 11
ATAGCTCAGCTGGGAGAGCAC 12

CATTACTGACGCTGAGGTGCA 28
TTTGTCCTGGGTGACAGCGTA 48
CGGGTGTGGAAGTGCGGCAAC 503
GGTGGACTGGTAGAGAATACG 10
GAGACAGGTGCTGCATGGCTG 217
AAAAGCCATCTCAGTTCGGAT 108
ACTGCCGGTGATAAGCCGAGA 598
TTAAAACTCAAAGGAATTGAC 79
GATCCCATTCCGAACTCGGCC 30
AGATGCGGGGTTCCTGCGGTT 51
GAGCTGGGCTACACACGTGCT 12
TACTAATAGCTCGATCGACTT 1024
TTGCAGCGAAGCGGTTCCAGG 110
AACATGGGTCGACCACGATCC 1432
ACGAACGCTGGCGGCAGGCTG 16
GTCGATGGGAACCACGTTAAT 38
ACTGGGACTGAGACACGGCCC 656
GGGCTGGAGCAGGTCCCAAGG 17
GGACCATTACTGACGCTGAGC 12
GGCAACCCACCTTAGATGACT 24
TCAGCGACTGGGGTGAAGTCG 407
AGAGCGTCGACCGAATACCCC 3618
CGTACTGGAGGACCGAACCCA 3130
GGGGTGCGACTGATTATAGCC 16
AGGCGCTGACACGGATTTGAC 46
GCGTCGACCGAATACCCCCGG 5482
CGTATGTGCGTGGTAGCGGAG 56
ATGTTTGGTTAGGCGGAAGAG 31
GACCGGAGACAGGTGCTGCAT 221
CTGCGGCTGGATCACCTCCTT 82
ACGGGCTGGGCTACACACGGG 16
AAGACCACCACGTTGATAGGC 39
CAGCACTCGACGGGCGTGAGC 10
CCTGGTGGTTATGGCGGAGCC 28
CAAGTCCTCCCAGGCCCACCA 24
CGAAGGCGCGCGATGGTAGGC 13
GAGGCGCGCGATGGTAGGCTG 14
CGCAAGCCTGATCCAGCCATG 244
AACTTGAGAGTTTGATTCTGG 11
GAGACGAAAGTCGGTCATAGT 108
GCGGCTCACTGGACCATTACT 10694
CCTGCTTTGCAAGCAGGGGGT 91
AAAGGTACGCCGGGGATAACA 114
GCGAACCAGTACCGTGAGGGA 277
CTCTTTCTTCATTGTTGATTG 16
AACCCACCTGAAAACGAGTAT 10
GGAAGAACACCAGTGGCGAAG 33
AATATTGACAGGATCTGTCCC 152
CGAGCGCAACCCTCGCCCTTA 14
TTCCCGGGTCTTGTACACACC 10
GATGACTTGTGGCTAGGGGTA 15
ACACGTACTGGAGGACCGAAG 13
AACTTGAGAGTTTGATCCTGG 62
AGAGGATGATCAGTCACACTG 11

CGGAATAACTCAGGGAAACTT 153
AGGTTCAAGTCCTCCCAGGCC 36
GGGTAAGTTCCGACCTGCACG 17
CGCAGGTTCAAATCCTGCCCC 12
TCGGATTGCACTCTGCAACTC 408
GTAGAGCATACCAAGGCGCTT 10
AGTACGAGAGGACCGGGATGG 186
GGACTGAGACACGGCCCAGAC 144
TAGCTGGTTCTCCGCGAAATC 261
TAAACGGCGGCCGTAACAATA 12
GCGATAAGCGTCGGGGAGGTG 58
TATTGGACAATGGGCGCAAGC 650
TAGTGAACCAGTACCGTGAGG 33
AAGCGTGGGGTCGGAGGTTAA 17
ATCCTGGCTCAGAACGAACGC 1500
AGAATACCAAGGCGCTTGAGA 64
GGGGTGAAGTCGTAACAAGGT 38
CGTAAGCCTGTGAAGGGACAG 18
TGTAGCTCAGCTGGGAGAGCA 26
TGCGGCAACGCATGCAGCTTA 21
CGAATACCCCCGGGGGTAGAG 53
GTAGGGTCAGCGACTGGGGTG 196
GATGAAGGCCCTAGGGTTGTA 188
GATCGCGTGTGTTGTGAGGTC 592
CGGTAACCGGAGAAGAAGCCC 961
GGCATGCACAGGCGATGAAGG 328
ACACGTACTGGAGGACCGAAA 50
GATGACTTGTGGCTAGGGGTG 11800
GGCGGAGCGGCTGCACCCGAT 3564
ATGGATGTCTAACTGCGGCCC 166
ACCGACACTGGTGGACTGGTA 419
AGTCCACGCCGTAAACGATGA 24
TTACGAAAGTCTGCCTGTTCT 10
CTTGTGGCTAGGGGTGAAAGC 10
TGTTTACCAAAAACACAGGGC 19
GAGGCGCGCGATGGTAGGCTA 11
GAGTAACGATAAAGGGAGTGA 15
TCCGAATGGGGCAACCCACCT 938
CCGTAGCTCAGCTGGGAGAGC 7271
TGGACCGGAGACAGGTGCTGC 385
AAGGCGTAGTCGATGGGAACC 334
GCGGCTCACTGGACCATTACC 51
ACTCGAGTGCATGAAGTTGGA 165
CGGTAACACGTACTGGAGGAC 414
GCAGCGAGCACGCGAGTGTGA 29
AAACGCTCCAGCGCCAATGGT 49
AGGACCGGGATGGACGTATCT 9150
GTAGATATTCGGAGGAACACG 63
CTGAAACATCTAAGTACCCAG 54
GAGGACCGGGATGGACGTATA 16
GGGATGACTTGTGGCTAGGGA 32
CTGAGCAGGGGTAGCCGGCCC 10
TCCCAAGGGTATGGCTGTTCT 20
CACACTGGGACTGAGACACGG 716

AAGGCAAAGAACAGGCGCAGC 29
ACCGTACCCTAAACCGACACT 962
CCAAGGCGCTTGAGAGAACTG 83
TTGTCGGGTAAGTTCCGACCT 65
TAACGGAGGCGCGCGATGGTA 3412
TCTAGAAGCCCGGCACCGCAG 24
TGTCCCTAGTACGAGAGGACC 58
TGCAATGGCATAAGCCTGCCT 34
CATCCTGGGGCTGGAGCAGGT 413
GTGAAATCCTGTCTGAACATG 69
TACCGGTACTAATAGCTCGAT 506
AAGTCGGTCATAGTGATCCGG 132
GCTGGACCGGAGACAGGTGCG 13
AACATCTAAGTACCCAGAGGA 53
TAGAGCAGCCCGGTAGCTCGT 10
TGTAGCTCAGTTGGTTAGAGC 120
GTCAGCTCGTGTCGTGAGATG 273
TAGGCCGGGTGTGGAAGTGCG 483
CGAGAATTGGAAAGAGGCCGG 71
GCAGTGGGGAATATTGGACAA 17
ATAAAAGGTACGCCGGGGATA 216
CCATCAGGGCCGACGGCCGGT 39
CACCTCGATGTCGACTCATCG 30
GCACCTCGATGTCGACTCATC 53
CGCATGAAGCTTACCGGTACT 72
TGACTGCGAGACTGACAAGTC 10
CTAAGTACCCAGAGGAAAGGA 490
CCCCGTGAAGATGCGGGGTTC 27
ATCGTTTACGGCGTGGACTAC 12
TCCCAAGGGTATGGCTGTTCC 70
GCAAATGATCGGCCCGCGTTG 2131
CACCGAAGCTGTGGATGCACG 201
TTACTGATCCTAACCAAACTC 13
ATCGCAGGCCAGTCAGCCTGA 26
GCGACTTAGTGTATCGAGCAA 119
ACCGTACCCTAAACCGACACC 12
CAGTTTGACTGGGGCGGTCGC 25
GGGAGTTGGTTTTACCCGAAG 16
GTGGTGCATGGCTGTCGTCAG 15
AAGGGGACTGCCGGTGATAAG 560
ATAACCACCAGGTCGGCGAAG 58
GAAAGGACATCAAACGAGACT 19
GTAGATATTCGGAGGAACACA 41
ATGGTGGGTAGTTTGACTGGG 79
TTTGGGCTGCGCCTGTTCTTT 23
TGAAACAGTACCTGAAACCGG 42
GAGGACCGGGATGGACGTATG 22
GAAAACTCGACCGAAGTGGGT 16
GGGATGACTTGTGGCTAGGGG 15069
GTGGAAGGGCCATCGCTCAAC 45
CGGGTCTTGTACACACCGCCC 12
AGAGACGAAAGTCGGTCATAG 84
GAGGCGCTGACACGGATTTGA 51
TTCGGAAGAACACCAGTGGCG 43

GAAGTCGGAATCGCTAGTAAT 274
TAGGCCGGGTGTGGAAGTGCA 13
CAGGGCTCTGCGAAGTCGCAA 1149
TGGCTAGGGGTGAAAGGCCAA 47
TCGGTTCGATCCCGTCCGGCT 769
TCTGCGAAGTCGCAAGACGAC 309
AACGGAGGCGCGCGATGGTAG 12608
CGAACGCTGGCGGCAGGCTTC 22
CGCGGGATGGAGCAGCCCGGT 14
TTAGACGGAAAGACCCCGTGC 305
ACGGATAAAAGGTACTCCGGG 39
ACCCCGACGAGGGGAGTGAAA 12
CCCGACGAGGGGAGTGAAACA 14
GTGAAGAGAAGATGTAATCGG 22
GCTGGACCGGAGACAGGTGCA 10
ACGCGCAGAACCTTACCAGCC 6965
TAGAACATAGATCGCAGGCCA 28
TCTAAGGGGACTGCCGGTGAT 139
TATGGAAGTAGGGCAATAAGG 36
TGACGGATCGCGTGTGTTGTG 47
TTCGTCTCAAGACGCGGGAGA 38
GGTTAAGAGGAGAGGTGCAAG 10
CTCAACGGATAAAAGGTACTC 88
CAAGACGACGTATAGGGTCTG 48
GCTGTAGCTCAGCTGGGAGAG 109
AGTAACGATAAAGGGAGTGAG 22
CGGCAACGCATGCAGCTTACC 32
GAAACTCAAAGGAATTGACGG 11
TGGGGAACTGAAACATCTAAG 190
CCTGACTTTGGTTTTCGGATC 91
CGGGGTGGATCTGTGGATCGC 13
GGGTGAAAGGCCAATCAAACT 10
GGACCGGAGACAGGTGCTGCA 303
GGATCGCGTGTGTTGTGAGGT 655
AGAAAGCGTAACAGCTCACTG 193
ACTGGGGTGAAGTCGTAACAA 35
TAACGCATTAAACATTCCGCC 149
GCGGGACACGTGAAATCCTGT 19
ACGGACCAGACTCCTACGGGA 28
ATCAAACGAGACTCCGCTAGT 77
GGGATGGAGCAGCCCGGTAGC 15
GAACATGGGTCGACCACGATC 1444
GGTGGTGACAGTGGGCAGCGA 111
GCAAACAGGATTAGATACCCT 18
ATGTTGGGTTAAGTCCCGCAA 22
GGAGTGAAACAGTACCTGAAA 11
ACTGGCATTCGTGACGACATG 484
GTGGCGAGCGAACGCGGACCA 70
TCTGAACAGGGCGTTCAGTTC 16
CGGGATAACCGCTGAAGGCAC 12
TAAGTCAGGGGTGAAATCCCG 28
AGGTAGCCGTAGGGGAACCTG 15
GGCTCACCAAGGCGACGATCC 227
GTCGGAGGTTCAAGTCCTCCC 44

TGGGGAACTGAAACATCTAAA 19
GTTTGTCCTGGGTGACAGCGT 32
CTCGGCCGGGAAACGCTCCAG 17
CCAATGGTACTTCGTCTCAAG 28
GTGCCAGCAGCCGCGGTAATA 15
CGGACTTTTAAGTCAGGGGTG 107
ACTGTTTACCAAAAACACAGG 20
GCCGGTAGGTGTAGGCGCAGC 220
TGAGCCTGACGAGCTACCGGG 48
ACACGTGAAATCCTGTCTGAA 42
GAAAAGCACCCCGACGAGGGG 31
CGGAGGCGCGCTATGGTAGGC 24
GATGATCCGCCACACTGGGAC 16
GAAGGGGGCTAGCGTTGTTCG 601
GTTGGTTTTACCCGAAGGCGC 93
GCAGGGTAGCTATATACGGAC 31
AAGCTCTTTCACCGGTGAAGA 207
GGGCTGGGCTACACACGCGCT 13
TGTGCCACCCCCCTATGGTTG 15
TGTCTCCAACGCAGACTCAGT 29
TGCCGCGTGAGTGATGAAGGC 26
GCGGGAGAGTAGGTCGCTGAC 10
AACGAGCGCAACCCTCGCCCT 68
AAGCACCCCGACGAGGGGAGT 32
TCGGCCGTGAAACGCTCCGGC 10
CTGGTAGAGAATACCAAGGCG 34
GTCGTGAGACAGTTCGGTCTC 15
TCCAGGACCGTGTATGGTGGG 10
GCATTCAGTTGGGCACTCTAA 26
CGGGATAACCGCTGAAGGCAT 240
ATCAGGGCCGACGGCCGGTCG 26
GCCCAAGGTTTGTCCTGGGTG 38
TCCAGCGCCAATGGTACTTCG 40
GGAACTCGGCAAAATGCACGC 544
CATGGCCCTTACGGGCTGGGC 119
GAACATAGATCGCAGGCCAGT 25
CCTAGGGTTGTAAAGCTCTTT 2159
CAGCAGTGGGGAATTTTGGAC 52
TACTTCGTCTCAAGACGCGGG 38
TCCATAGCTGGTCTGAGAGGA 54
ATGAAGGCCCTAGGGTTGTAA 805
TTTTGTTGGAGCAACGCTGGA 34
GCTGGCGGCAGGCTTAACACA 304
TGCGAAGTCGCAAGACGACGT 199
ATCGAGCAAGCTTAAGCCGGT 14
GGGCTGGGCTACACACGGGCT 14
ACAGTACCTGAAACCGGATGC 7003
CTGTTGTGGCGCCAGCCGCAT 2222
TGTATAATGGGTCAGCGACTT 63
TACCTGACTTTGGTTTTCGGA 19
AGGACCGAACCCATATCTGTT 29
TGACAAGTCGAGCAGAGACGA 17
CGGGATGGAGCAGCCCGGTAG 15
GAGTATTCCCTATCAGAGCCG 16

TGCTCACGGGCCGTACCGCAG 17
TCTCAGTTCGGATTGCACTCT 187
TGGCATAAGCCTGCCTGACTG 278
CATAGTGATCCGGTGGTCCCG 581
AAAACAACCAGGATGTTGGCT 71
TCGTCAGCTCGTGTCGTGAGA 241
ACCAACTTCGATCCGAAAACC 17
GTGGGCCTGCAGGTGGTGACG 358
ACCGAATACCCCCGGGGGTAG 271
GCGTAAAGCGCACGTAGGCGG 342
TACTCCGGGGATAACAGGCTG 77
GGCTCAAGCCATACACCGAAG 429
GAAGTTGGAATCGCTAGTAAT 123
GGGCGGCGGCGCCGGCAGCGG 39
ACTCCGCTAGTAGTGGCGAGC 65
TGGCGGCAGGCTTAACACATG 183
TGGCGGAGCGGCTGCACCCGA 4251
GGTCTGACGCCTGCCCGGTGC 1389
ACGCGTAGAACCTTACCAGCC 12
AGTTTGACTGGGGCGGTCGCC 286
GATGACTTGTGGCTAGGGGCG 33
GGAGTACTAGTCGGCAGACAC 16
ACGACGTATAGGGTCTGACGC 701
TACGGTCGCAAGATTAAAACT 188
GTGATACGCTGCGATAAGCGT 29
TTACTGATGAAAATGGATTGA 10
TTATGGCGGAGCGGCTGCACC 9521
CCCCGGCTAACTTCGTGCCAG 69
CCGGTAGGTGTAGGCGCAGCC 12
GGCAACCATAGGGGGGTGGCA 33
TTGGGGGTCATCAGCCTGTTA 15
CACGCTTGATAAGCGTGGGGT 16
CGCTCAACGGATAAAAGGTAC 201
ACGTCAAGTCCTCATGGCCCT 118
AACGCGCAGAACCTTACCAGC 73
AGATAGCTGGTTCTCCGCGAA 42
TTCAGAACGTCGTGAGACAGT 122
TCAAACGAGACTCCGCTAGTA 28
CTGGAGGTATCGGAAGTGAGA 23
TCGGAGATATTCGGAGGAACA 21
TTATTTGGTTGCGGGGGCAGG 44
CCTAAGGCGAGGCCGAAAGGC 15
GGGCTGTAGCTCAGCTGGGAG 274
CCCGCTGTCTCCAACGCAGAC 819
GACCATTACTAACGCTGAGGT 10
TGAGTGATGAAGGCCCTAGGG 14
CGGCTAACTTCGTGCCAGCAG 14
TTATGGCGGAGCGGCTGCACT 14
CGCTGAGGTGCGAAAGCGTGG 74
TGACGGGGGCCCGCACAAGCG 156
TACACACGTGCTACAATGGTC 12
ACAGGGCTCTGCGAAGTCGCA 1833
CTGCAACTCGAGTGCATGAAG 13
GCGCGGCCCATCAGGGCCGAC 33

GAAGCAACGCGCAGAACCTTA 24
ACCAGGCCAGTGGCTTTTGTG 177
CGCGTAGTAGCGTTTGCGTCG 10
GGCTCAAGCCATACACCGAAA 23
CGGGACACGTGAAATCCTGTC 20
ATCCGACGATTTCCGAATGGG 131
TGACGCTGAGGTGCGAAAGCG 63
ATGGTAGAGGTGAGTGGAATT 34
TGGCGGAGCGGCTGCACCCGG 12
AAAAACACAGGGCTCTGCGAA 70
TTGGAATCGCTAGTAATCGCG 1315
AGAGTTTGATCCTGGCTCAGG 11
GCTTTGCAAGCAGGGGGTCGT 89
GAGTGAAACAGTACCTGAAAC 22
CAACCATAGGGGGGTGGCACA 29
AACCTGGGGAACTGAAACACC 14
CGGCATTGTCTGCGGATGGTG 51
CCGAAAGTCCAAGGGTTCCTG 10
ACTTGATCACTCCCATTTACC 13
GTGTAGGCGCAGCGAAAGCGA 14
GTTAGGCGGAAGAGATTTTGG 11
ACATCCTGGAGGTATCGGAAG 45
AATGGTACTTCGTCTCAAGAC 102
AAGATGCGGGGTTCCTGCGGT 38
CACGGTAGGGTCAGCGACTGG 107
TGAGGTGCGAAAGCGTGGGGA 80
GCGAGGCCGAAAGGCGTAGTC 11
AAAGCCATCTCAGTTCGGATT 113
TGGTTGACAGGTTGGTTTGAC 10
TGAATTGATTGAGGGTTTTGA 19
ACTTCGGAAGAAGCGTGACCT 90
AGTTTGACTGGGGCGGTCTCC 45
TCGGGATGACTTGTGGCTAGG 16004
AGATCGGGATGACTTGTGGCT 435
AGTGCGGCAACGCATGCAGCT 11
GGGGAACTGAAACATCTAAGT 210
GGATAGGTGGTAGACTTTGAA 35
ACGGGATAACCGCTGAAGGCT 30
TCAAGTCCTCATGGCCCTTAC 108
TAAGCGTGGGGTCGGAGGTTA 41
CCTTTGATCCGACGATTTCCG 1001
TTGACTGATCTAGAAGCCCGG 37
CCCATTCCGAACTCGGCCGTG 112
GGGTGGAGCAGCCCGGTAGCT 210
AATTTTGGACAATGGGCGCAA 52
TTATGGCTAAGTGGGAAAGGA 100
CCCGGTCGCGGTTAGTGGAGA 251
GTTAGCCGGCCCCTAAGGCGA 56
GTTTGACTGGGGCGGTCTCCT 40
TTTTACATGGGGGTGCGACTG 12
TCGTCGGTTCGATCCCGTCCG 248
GCGAAATTCCTTGTCGGGTAC 24
ATTCGTGCAGGTCGGAACTTA 11
AAATTCCTTGTCGGGTAAGTA 15

ATATTGGACAATGGGCGAAAG 41
GGGGGTCATCAGCCTGTTATC 15
AACGGATAAAAGGTACGCCGG 68
TACTTGATGAGGGGCCGTAGC 105
CTGGGGCTGGAGCAGGTCCCA 14
TCGGGATGACTTGTGGCTAGA 12
TTGTCGTCAGCTCGTGTCGTG 17
AGAGTTTGATCCTGGCTCAGA 444
TTAATCTGAGCAGGGTTAGCC 74
ACTTCGTGCCAGCAGCCGCGG 23
CGGCATTGTCTGCGGATGGTA 45
AACTGAAACATCTAAGTACCC 32
ACGTATGTGCGTGGTAGCGGA 67
TGGGGAATTTTGGACAATGGG 51
CTGGTTCTCCGCGAAATCTAT 11
ATTCGTGGGCCTGCAGGTGGT 448
CCGCTAGTAGTGGCGAGCGAA 52
CTGGTGGACCTGTTGTGGCGC 649
GGTAGAGAATACCAAGGCGCT 24
ACAAGTCGAGCAGAGACGAAA 28
CCCGCACAAGCGGTGGAGCAT 12
GGAGGTTGGCTTAGAAGCAGC 75
ATAAAGGGAGTGAGAGACTCC 23
AGAACATAGATCGCAGGCCAG 27
GGCGGCATTGTCTGCGGATGC 16
CTTGAGTATGGTAGAGGTGAG 31
GGTCCCAAGGGTATGGCTGTA 43
GCGTCGGTATCTGGGCTTGTA 54
ACGGGATAACCGCTGAAGGCC 54
TGCGTCGGTATCTGGGCTTGT 50
TAAGCGTGGGGTCGGAGGTTG 20
GGTGTAGGCGCAGCGAAAGCG 27
CAGTTCGGATTGCACTCTGCC 12
AGCTATGTACGGACGGGATAA 11
ATTGGAAAGAGGCCGGATTTA 10
CTCGACCGAAGTGGGTGATAG 251
TTGAGAGTTTGATCCTGGCTC 1944
TTGATAAGCGTGGGGTCGGAG 32
ATGGCGGAGCGGCTGCACCCT 10
GAAGCTTACCGGTACTAATAG 90
CCTTTTGTATAATGGGTCAGC 25
CGGGGTTCCTGCGGTTAGACG 622
CACTCTGCAACTCGAGTGCAT 161
ATGACTTGTGGCTAGGGGGGA 39
AGGGCAACAACCCTGACCACC 180
TTGGGTTCAGAACGTCGTGAG 145
TTTGATCCTGGCTCAGAACGA 2715
CGTCGGCCCATGTGGGCCGCC 21
GTAACGATAAAGGGAGTGAGA 49
ACGATTTCCGAATGGGGAAAC 19
CCGACGAGGGGAGTGAAACAG 14
TCTAAGTACCCAGAGGAAAGG 416
CGGGGGAAAGATTTATCGGCA 20
CCGTATGTGCCCTTCGGGGGA 61

TTCTCATGTTTGTGTTCTTCG 21
GCGGACCTGGGGAACTGAAAC 12
ACGAGAGGACCGGGATGGACA 38
GTCGTGAGACAGTTCGGTCCC 620
ACTCACCGTCTTACTGATCCT 586
CTAGCGTTGTTCGGATTTACT 124
TTAAGAGGAGAGGTGCAAGCC 107
GCTCACGGGCCGTACCGCAGC 14
GAGCACCTGCTTTGCAAGCAG 34
GCAGACCTGGCAGCGACCTAC 14
ATGCAAGTCGAGCGCCCCGCA 85
CTAGTACGAGAGGACCGGGAT 103
TACCTGAAACCGGATGCATAC 11
ATGTTGGCTTAGAAGCAGCCA 120
CGTTAGTAGTGGCGAGCGAAC 13
GGTTTGTCCTGGGTGACAGCG 32
AGTGGGGAATATTGGACAATG 29
GCTGCATGGCTGTCGTCAGCT 1461
TGAAGTCGGAACAAGGTAGCC 20
AGGGGTGAAAGGCCAATCAAA 12
CGATTTCCGAATGGGGAAACC 19
GACGTATCTCTGGTGGACCTG 61
GCGTAGTCGATGGGAACCACG 64
AGAGTAACGGAGGCGCGCGAT 20
GGATAAAAGGTACGCTGGGGA 13
ATCTGCCGTGGGTGTAGGAAT 16
CACTATCCTTCAGTTAGGCTG 21
CTCAACGGATAAAAGGTACCC 31
CAAATCCTGCCCCCGCAACCA 32
TGGGTCGACCACGATCCAAGC 973
ACTCGGCCGTGAAACGCTCGA 14
ATGGCTGTTCGCCATTTAAAG 11
TCTAAGCGGGAAACCCACCTG 310
CAGCAGTGGGGAATATTGGAC 15
ACGAGAGGACCGGGATGGACG 5119
GCGGCATTGTCTGCGGATGGA 18
TTTGCGTCTCCGTGTTTTACA 27
CTCGAGTGCATGAAGTTGGAA 56
GTACTTCGTCTCAAGACGCGG 74
TCCGTAAGCCTGTGAAGGGAC 35
GGCTGGGCTACACACGTGCTC 10
ATCGTTCGTCGAGTGCAATGG 33
AGCAGGTCCCAAGGGTTTGGC 11
GGTAGGGTCAGCGACTGGGGT 112
GTGATCAAGTGTCTTAAGGGC 99
GGTGTAGGAATATTGACAGGA 52
GCGAACCTGGAGAACTGAAAC 11
GTGGGGAGTTTGACTGGGGCG 230
TCGCAAGATTAAAACTCAAAG 185
CGGTAGGGTCAGCGACTGGGG 122
GGTGATAGTCCCGTACACGTA 29
TGGGGCTGGAGCAGGTCCCAA 14
TGGGTGTAGGAATATTGACAG 44
TGAACTTTGGCGGACACGTTT 19

TCCGGTGGTCCCGCGTGGAAG 45
ACCATTACTGACGCTGAGGTC 16
CGGGTGAGTAACGCGTGGGAA 18
CCCTATCTGCCGTGGGTGTAG 175
TCCGCCTGGGGAGTACGGTCG 3545
TTCCTGCGGTTAGACGGAAAG 17
CTCACTGGTCTAAATAAGGGT 23
AACCACCAGGTCGGCGAAGAA 54
TGACTGGGGCGGTCTCCTCCT 22
CTGGGGAACTGAAACATCTAA 580
TAGGGTTGTAAAGCTCTTTCA 1618
ACGACATGTGTAGGATAGGTC 14
GTAGTTTGACTGGGGTGGTCG 15
AGTAATCGCGGATCAGCATGC 158
CTGACTTTGGTTTTCGGATCG 81
CGCAACGAGCGCAACCCTCGC 114
AACATAGATCGCAGGCCAGTC 34
CGCGGGGTGGAGCAGCCCGGT 286
TCACGGGCCGTACCGCAGCTG 95
GACCTGGTGGTTATGGCGGAC 53
GATTTGTGAGTAGTTGGGGGT 15
CGTGAAACGCTCCAGCGCCAA 3392
GAAGACCACCACGTTGATAGG 22
GCAACGCATGAAGCTTACCGG 13
GTCGACTCATCGCATCCTGGG 256
CTCCGCTAGTAGTGGCGAGCG 66
CTCCTACGGGAGGCAGCAGTG 71
AGCTGTGGATGCACGTATGTG 31
TATTCCCTATCAGAGCCGTGG 18
GGCGTAAAGCGCACGTAGGCG 267
GAGTAACGGAGGCGCGCGATG 65
CGGAGGCGCTCGATGGTAGGC 10
TTCGGAGGAACACCAGTGGCG 3954
ATGACTTGTGGCTAGGGGTGA 2701
TGGCCGTGAAACGCTCCAGCG 16
TCAGAACGTCGTGAGACAGTT 315
AGCCTGATCCAGCCATGCCGC 132
CGCGGGGTGGAGCAGCTCGGT 10
GACCTGCACGAATGGCGTAAC 103
TGCAACTCGAGTGCATGAAGT 13
TTTCTAGTCATCATAAATAAG 426
GACTCACCGTCTTACTGATCC 470
TAGCTCAGCTGGGAGAGCACC 5328
ACGGAGGCGCGCGATGGGAGG 11
AAGTACCCAGAGGAAAGGACA 573
TGATCGGCCCGCGTTGGATTA 207
AGTAATCGCGGATCAGCATGT 20
AGTCTGAACAGGGCGTTCAGT 26
GCGTAGAACCTTACCAGCCCT 12
GTACGGTCGCAAGACTAAAAC 10
TGTGCCCTTCGGGGGAAAGAT 10
CTCTGCGAAGTCGCAAGACGA 304
ACGGATAAAAGGTACGCTGGG 15
AAGATTAAAACTCAAAGGAAT 81

GATGATCAGCCACACTGGGAC 6092
TGGAGGTATCGGAAGTGAGAA 16
TGGCTGTTCGCCATTTAAAGC 21
GGGCAACCATAGGGGGGTGGC 37
TGGGTTCAGAACGTCGTGAGA 172
AATTCGTAGATATTCGGAAGA 32
ACCTTAGATGACTAGAAAATC 169
TAATATTCGTGGGCCTGCAGG 41
TAGGATGTTGGCTTAGAAGCA 23
CGATGTCGACTCATCGCATCC 45
GGGTCGTCGGTTCGATCCCGT 334
GGGGAATTTTGGACAATGGGC 53
CGAGTGCATGAAGTTGGAATC 236
TGACTTTGGTTTTCGGATCGA 81
TCTATTTAGGTAGAGCGTCGA 25
GGTGGACCTGTTGTGGCGCCA 236
ATGACTTGTGGCTAGGGGTGG 107
CTCGTGCATGACCGATAGCGA 315
AGACGAAAGTCGGCCATAGTG 10
TTACTTGATGAGGGGCCGTAG 102
TTAGAAGCAGCCATCATTTAA 10
TGCATGAAGTCGGAATCGCTA 10
AAGTCGAGCGCCCCGCAAGGG 76
CTGAGCAGGGTTAGCCGGCCA 31
GAGAAGCTGGTCTTTCTGCTG 304
GGCCCCTAAGGCGAGGCCGAA 13
GAGTGCAATGGCATAAGCCTG 24
TCAGTTCGGATTGCACTCTGC 211
AACGCTCCAGCGCCAATGGTA 54
CGGGGTGGAGCAGCTCGGTAG 10
AAACATTCCGCCTGGGGAGTA 152
CCTTAGGGTTGTAAAGCTCTT 15
ATCGGATCAACTGAAGAGTTG 14
GATACGCTGCGATAAGCGTCG 32
TGGGCAACCATAGGGGGGTGG 30
TATCTCTGGTGGACCTGTTGT 58
CGAGCAGAGACGAAAGTCGGT 129
GTGGTAGACTTTGAAGCAGGG 255
CGGCCCCTAAGGCGAGGCCGA 16
CCGGTAGGTGTAGGCGCAGCG 149
GCGCAGCGAAAGCGAGTCTGA 23
ACCCTGGTAGTCCACGCTGTA 32
ATGGGAACCACGTTAATATTC 23
TCAGCCACACTGGGACTGAGA 297
GAGGCGCGCGATGGTAGGCCC 31
ACGATAAAGGGAGTGAGAGAC 795
GCAGCCCGGTAGCTCGTCAGG 464
TCGGGGGAAAGATTTATCGGC 34
CCTAGTACGAGAGGACCGGGA 28
GGGGAGTACGGTCGCAAGATT 69
GGCGGCAGGCTTAACACATGC 165
TGATACTGGAAGTCTTGAGTA 18
CCATTACTGACGCTGAGGCGC 25
TAGTAATCGCGGATCAGCATG 155

CGGTTCGATCCCGTCCGGCTC 684
GTGAGTGATGAAGGCCCTAGG 17
TTCGCCATTTAAAGCGGTACG 13
TTGACTGGGGCGGTCGCCTCC 141
GTACTCCGGGGATAACAGGCT 85
GTACGGTAACACGTACTGGAG 37
GTTAGGCTGGACCGGAGACAG 184
AAGTCCTCATGGCCCTTACGG 112
AAGTGGGTGATAGTCCCGTAC 71
GTCCCGCAACGAGCGCAACCC 74
GGACTGCCGGTGATAAGCCGA 2047
GCTTGAGAGAACTGCGTTGAA 1814
TCAGTGAAATTGAATTCCCCG 38
AAGAAGCGTGACCTCACTATG 89
CCGGCCCCTAAGGCGAGGCCG 16
TAATGGGTCAGCGACTTAGTG 73
AATGGTGGTGACAGTGGGCAG 94
AACTCAGGGAAACTTGTGCTA 22
TGATGAAGGCCCTAGGGTTGT 108
GCCGGGAAACGCTCCAGCGCC 18
AATATTGGACAATGGGCGAAA 30
CGCCTGCCCGGTGCTGGAAGG 168
TGAGCAGGTTGAAGGTACGGT 503
ACGGTAGGGTCAGCGACTGGG 129
AACCCTGACCACCATCTAAGG 29
TACAATGGTGGTGACAGTGGG 16
TTGATCCGACGATTTCCGAAT 308
CTGTTGTGGCGCCAGCCGCAA 10
ACAGTCGTGAGACATCCTGGA 37
TCGTCTCAAGACGCGGGAGAG 157
AGACCCCGTGCACCTTTACTA 10
GGGCCATCGCTCAACGGATAA 95
GGTGTGACGCCTGCCCGGTGC 11
GGGTGAAGTCGGAACAAGGTA 15
GGAGGCGCGCGATGGTAGGAT 47
GAGAAGAAGCCCCGGCTAACT 29
TCGAGAATTGGAAAGAGGCCG 107
CTTTGGCGGACACGTTTCTTG 175
TTCGGTCCCTATCTGCCGTGG 1406
TCGACTTGATCACTCCCATTT 387
GGGTCGGAGGTTCAAGTCCTC 283
TTTTGGACAATGGGCGCAAGC 64
GATCGAGAATTGGAAAGAGGC 453
TAATTCGAAGCAACGCGCAGA 28
TCAGGGGTGAAATCCCGGGGC 73
GACCATTACTGACGCTGGGGT 16
GACACTGGTGGACTGGTAGAG 376
CAGTGGCGAAGGCGGCTCACT 373
CTCCGAATACCGGGGAGTACT 20
ACGGTAACACGTACTGGAGGA 288
TAGTAGTGGCGAGCGAACGCG 34
TAGTGATCCGGTGGTCCCGCG 109
GGGGGCTAGCGTTGTTCGGAT 877
CTTGTACACACCGCCCGTCAC 23

CACGGCCCAAACTCCTACGGG 12
CCATTACTGACGCTGAGGAGC 10
TAACCACCAGGTCGGCGAAGA 52
TCGTGAGACATCCTGGAGGTA 117
CTAAGTGGGAAAGGATGTGAG 140
AACGGTCGGAAATCGTTCGTC 118
TTTGATCCGACGATTTCCGAA 523
ACTTGATCACTCCCATTTACA 34
AGCACTGGATGGGCTATGGGG 77
CGGCATTGTCTGCGGATGGTT 156
TTGGCACCTCGATGTCGGCTC 11
CTCAGAACGAACGCTGGCGGC 2696
CGGCCGGGAAACGCTCCAGCG 19
TTCAAATCCTGCCCCCGCAAC 29
ATGGAGCAGCCCGGTAGCTCG 15
ATGACCGATAGCGAACCAGTA 129
AAATTCCTTGTCGGGTAAGTT 315
CTTAACACATGCAAGTCGAGC 30
GCGAAATTCCTTGTCGGGTAG 17
TGAAGGCCGCAGGTTCAAATC 42
TTGGTGGGCCTGGGAGGACTT 12
GGCCTAACACATGCAAGTCGA 10
TAAGCGTGGGGTCGGAGGTTC 2466
GGAACCACGTTAATATTCGTG 23
ACGGGATAACCGCTGAAGGCG 16
GGTCCCAAGGGTATGGCTGTT 2447
TTATGAGCCTGACGAGCTACC 896
GGCGGCATTGTCTGCGGATGG 768
ACGTATAGGGTCTGACGCCTG 944
GGGAACCTGCGGCTGGATCAC 34
AATAGCAGAAGTCCTTGAGTA 91
GAGGTTCAAGTCCTCCCAGGC 41
GGCAACGCATGCAGCTTACCG 29
CCCGGTCGCGGTTAGTGGAGC 10
GCGAACCTGGGAAACTGAAAC 12
CAGAGACGAAAGTCGGTCATA 90
TAAGCGTCGGGGAGGTGCGAA 392
CATAGCTCAGCTGGGAGAGCA 12
ACCGGTACTAATAGCTCGATC 5375
TACGTGAGTTGGGTTCAGAAC 97
ACGTCGTGAGACAGTTTGGTC 26
GTAATCGCGGATCAGCATGCC 156
GCGAAGTCGCAAGACGACGTA 60
GTGCTGGGCTACACACGTGCT 10
GAGCAAGCTTAAGCCGGTAGG 114
GTTCCCGGGTCTTGTACACAC 19
CCCAGAGGAAAGGACATCAAA 18
TGGTGGTGACAGTGGGCAGCG 71
CGTACACGTAGAATAGCAGAA 175
ATCGTCGGTTCTTTGAAAACT 12
GGATGATCCGCCACACTGGGA 17
TTAGAAGCGAACCTGGGGACC 10
GCGAAATTCCTTGTCGGGTAA 4574
ACGTCGTGAGACAGTTCGGTC 799

CCTGCATATAGACCGTACCCT 777
CGGCATTGTCTGCGGATGGTC 42
GTAGGGCGGGACACGTGAAAT 15
GTGGTGACAGTGGGCAGCGAG 180
GGCGTAGTCGATGGGAACCAC 67
GACTTCCCCGCTGTCTCCAAC 191
TGCACGTATGTGCGTGGTAGC 35
AGCCCAGTGTAGAATGTCTGC 68
ACAACCCTGACCACCATCTAA 45
AGGGCTCTGCGAAGTCGCAAG 1119
ACACTGGTGGACTGGTAGAGA 2095
GGCGGCATTGTCTGCGGATGA 18
ATTAAACATTCCGCCTGGGGA 707
AGCCACATTGGGACTGAGACA 11
ATGGCGTAACGACTTCCCCGC 17
AATATTGGACAATGGGCGCAA 379
GAGTCTGAACAGGGCGTTCAG 41
CTTGGCATGCACAGGCGATGA 232
GACCGAACCCATATCTGTTGC 17
CAGTTCGGATTGCACTCTGCA 68
GATGACGTCAAGTCCTCATGG 10
CGCAACCCTCGCCCTTAGTTG 11
CCTTTGATCCGACGATTTCCC 11
TAAGCGTGGGGTCGGAGGTTT 11
CATTCGTGACGACATGTGTAG 33
TGGTAGAGAATACCAAGGCGC 33
ACGGGATAACCGCTGAAGGCA 888
GGGGAACTGAAACATCTAAGA 11
CAGGTGCTGCATGGCTGTCGT 753
ACGTGAGTTGGGTTCAGAACG 22
TTACCAGCCCTTGACATCCCG 65
CAGGGGTGAAATCCCGGGGCT 45
CGACCGAAGTGGGTGATAGTC 255
ATGGCGGAGCGGCTGCACCCG 6875
TCGCTAGTAATCGCGGATCAG 83
CAATGAGAGGGATCAAGTGTC 21
CGGATTTACTGGGCGTAAAGC 2580
ACGGGCTGGGCTATACACGTG 16
TAGAGGTGAGTGGAATTCCGA 98
GCCGCGGTAATACGAAGGGGG 289
CAAGGCGCTTGAGAGAACTGC 62
GATGGTAGGCTCAGAACGGTC 193
CATCGCATCCTGGGGCTGGAG 35
GACACTATCCTTCAGTTAGGC 16
GGAACTGAAACATCTAAGTAC 155
TGAAGGGACAGTCGTGAGACA 25
TTTTACCCGAAGGCGCTGTGC 387
GGAGGACCGAACCCATATCTG 31
GAGTTTGATCCTGGCTCAGAA 357
GTATGTGCCCTTCGGGGGAAA 45
CTTGCAGCGAAGCGGTTCCAG 112
GAGGCGCGCGATGGTAGCCTC 12
TAATCGCGGATCAGCATGTCG 19
ACCGGAGACAGGTGCTGCATG 371

CTGGAGAGTTTGATCCTGGCT 12
ACCATTACTGACGCTGAGGTG 16058
CCGTGAAACGCTCCAGCGGCA 11
CACGTATGTGCGTGGTAGCGG 55
TCTCTGGTGGACCTGTTGTGG 401
AATGGGTCAGCGACTTAGTGT 88
GAAGCGAACCTGGGGAACTGG 38
GCTGAGGTGCGAAAGCGTGGG 87
ACCATTACTGACGCTGGGGTG 16
GCCTGACGAGCTACCGGGCTG 17
TGGGCTACACACGTGCTACAA 321
TTGCAGACCTGGCAGCGACCT 21
GGGCTCAACCCCGGAACTGCC 36
GCGGCATTGTCTGCGGATGGT 509
AGCCATACACCGAAGCTGTGG 27
GGGGATAACAGGCTGATGACC 10
TGGTAGGCTCAGAACGGTCGG 29
TTGTATCTCGAGAAGCTGGTC 30
GCAACGAGCGCAACCCTCGCC 117
ACTTCCCCGCTGTCTCCAACG 273
GGGTGTAGGAATATTGACAGG 42
TCCCAAAACAACCAGGATGTT 87
AAACCAAGTGATCTAGCCATG 11
CATGAAGTTGGAATCGCTAGT 80
AGTGATCCGGTGGTCCCGCGT 68
TGAACCAGTACCGTGAGGGAA 29
TTGAAGGAACTCGGCAAAATG 163
TGAATACGTTCCCGGGCCTTG 181
CGTGATACGCTGCGATAAGCG 29
AGTTCGGATTGCACTCTGCAA 51
ATGGCGGAGCGGCTGCACCCA 12
GCAGCGAAAGCGAGTCTGAAC 30
GAGAGGATGATCCGCCACACT 11
AGAGCATACCAAGGCGCTTGA 10
GCGTAGTAGCGTTTGCGTCGG 10
CCAAAACAACCAGGATGTTGG 87
GCGCTGACACGGATTTGACCT 12
GAGGTATCGGAAGTGAGAATG 20
GGCTGGGCTACACACGTGCTA 13752
GAAGCGAACCTGGGGAACTGA 9180
GACTTGATCACTCCCATTTAC 235
GTGAAGGGACAGTCGTGAGAC 33
ATTTGGTGGATGCCTTGGCAT 37
GGTAGCCGTAGGGGAACCTGC 13
AGGGTTAGCCGGCCCCTAAGG 340
GGTGGAGCAGCCCGGTAGCTC 191
AGCAGAAGTCCTTGAGTAGGG 188
GCGGCATTGTCTGCGGATGGC 49
AGTTGGAGCCCAAGGTTTGTC 80
ACGAGAGGACCGGGATGGACT 15
ACCATTACTGACGCTGAGGTA 48
TAGCTCGTCAGGCTCATAACC 336
TTGGGCTGCGCCTGTTCTTTG 32
GACGTCAAGTCCTCATGGCCC 41

GGAGTTTGGTTAGGATCAGTA 17
TGTAGGAATATTGACAGGATC 119
CGGCTCACTGGACCATTACTG 8214
CTACACACGTGCTACAATGGT 26
TCGAGCAAGCTTAAGCCGGTA 101
TGCCTTTGATACTGGAAGTCT 10
CAAGGGTATGGCTGTTCGCCA 117
CGTATAGGGTCTGACGCCTGC 916
TATAGCCCAGTGTAGAATGTC 15
CTGGGGAACTGAAACATCTAC 13
GAAATTCCTTGTCGGGTAAGT 2169
CTGCATGGCTGTCGTCAGCTC 1435
ATAGGCCGGGTGTGGAAGTGC 297
TTTGTGAGTAGTTGGGGGTGG 20
CGTTCAGTTCGATGCATTAGA 322
AAAAGGTACTCCGGGGATAAC 72
ACGCTCCAGCGCCAATGGTAC 64
CGGGTAAGTTCCGACCTGCAC 17
GAAGCTGTGGATGCACGTATG 40
AGCTCAGTTGGTTAGAGCACA 83
GGAAGGGCCATCGCTCAACGG 84
TGCATGGCTGTCGTCAGCTCG 1085
AATATGGAAGTAGGGCAATAA 11
TCTAAGTTTCGGTTCATTGCC 27
ACGACATGTGTAGGATAGGTA 12
GCGGTTAGTGGAGACACTATC 41
CCCTTTGATCCGACGATTTCC 1200
TTCGTGCAGGTCGGAACTTAC 11
CGTGCCAGCAGCCGCGGTAAT 16
ACGGGGGCCCGCACAAGCGGT 295
CCTGACGAGCTACCGGGCTGC 20
ATGCGGGGTTCCTGCGGTTAG 129
ACACGCACTGGAGGACCGAAC 30
GACCTGGTGGTTATGGCGGAA 25
CTGAGCAGGGTTAGCCGGCCT 10
TAGCTCAGCTGGGAGAGCACA 12
TAGTCCACGCCGTAAACGATG 21
GAAACCGGATGCCTACAAACA 50
GTTTGATCCTGGCTCAGGACG 13
GACATGTGTAGGATAGGTGGT 984
CGGCCCAAACTCCTACGGGAG 14
CCATCTCAGTTCGGATTGCAC 111
ATAGCTCGATCGACTTGATCA 43
CTGGTCTGAGAGGATGATCAG 10
TCCGACGATTTCCGAATGGGG 182
CCGCAAGGAGGCAGGCGACCA 21
GTTTGGTTAGGCGGAAGAGAT 11
CGAGTGCAATGGCATAAGCCT 49
TCCTGCATATAGACCGTACCC 710
GGCTGGGCTACACACGTGATA 24
CCGACACTGGTGGACTGGTAG 413
AAGTACCCAGAGGAAAGGACC 13
ACGACATGTGTAGGATAGGTG 1113
AATAACGGTCCTAAGGTAGCG 10

GATTTCCGAATGGGGCAACCC 14
GAGGACCGGGATGGACGTACC 10
CCGGTAGCTCGTCAGGCTCAT 17
GCAGAACCTTACCAGCCCTTG 1422
TAACACGTACTGGAGGACCGA 213
CTGAGCAGGGTTAGCCGGCCC 10966
TAGCTCAGCTGGGAGAGCACG 11
GGAAGTAGGGCAATAAGGCAA 16
TCTTGGGGGTCATCAGCCTGT 20
CGACGTATAGGGCCTGACGCC 27
GTCGAGCAGAGACGAAAGTCG 70
GGGGGTGCGACTGATTATAGC 35
TGATAGTCCCGTACACGTAGA 38
AAGGAACTCGGCAAATTGCAC 21
CCTGTTGTGGCGCCAGCCGCA 3618
GAGCGGCTGCACCCGATCCCA 82
GGCCGGGTGTGGAAGTGCGGC 541
GTTGGGTTCAGAACGTCGTGA 45
ACGGGCCGTACCGCAGCTGAC 132
GTACACGTAGAATAGCAGAAG 78
GAAGGAACTCGGCAAATTGCA 20
GACCTGGTGGTTATGGCGGAG 3771
CTGATCTAGAAGCCCGGCACC 53
GACGAGGGGAGTGAAACAGTA 17
AGAGTAGGTCGCTGCCAGGTC 5710
AAATCCTGTCTGAACATGGGG 12
TCGGCCGTGAAACGCTCCAAC 11
ATACCGGGGAGTACTAGTCGG 481
GAAAGCGTAACAGCTCACTGG 206
TAGAGCGTCGACCGAATACCC 2024
CGCGGTTAGTGGAGACACTAT 44
TCTTGAGTATGGTAGAGGTGA 35
GAGGTGAGTGGAATTCCGAGT 111
TGAAGAGAAGATGTAATCGGA 23
ACCGCAGACGAGGCGCTGACA 12
GGTCGCAAGACTAAAACTCAA 12
CTTACCAGCCCTTGACATCCC 66
AGCCACACTGGGACTGAGACA 332
GACGGATCGCGTGTGTTGTGA 119
GAAGAAGCCCCGGCTAACTTC 23
CCGCCACACTGGGACTGAGAC 18
CGGAGGTTCAAGTCCTCCCAG 43
CGTTGATAGGCCGGGTGTGGA 48
CCCCTAAGGCGAGGCCGAAAG 15
CGGGCTGGGCTGCACACGTGC 11
CGGAGGCGCGCGATGGTAGGC 37976
TGAGGGAAAGGTGAAAAGAAC 13
TAGGATAGGTGGTAGACTTTG 45
AGCAACGCGCAGAACCTTACC 34
AGCGAACAGGATTAGATACCC 13
GGTGGTCCCGCGTGGAAGGGC 40
TTAAACATTCCGCCTGGGGAG 703
ATGGAAGTAGGGCAATAAGGC 35
GCCGTAAAACGCTCCAGCGCC 12

GGATAACCGCTGAAGGCATCT 93
AGGCGCGCGATGGTAGGGTCA 10
AGTCGGTCATAGTGATCCGGT 131
CTGGGCTACACACGTGCTACA 9786
GGATAAAAGGTACCCCGGGGA 75
TGATCCGACGATTTCCGAATG 254
AGTAGGTCGCTGCCAGGTCTG 876
TAGCTCCTGCATATAGACCGT 268
AATCGTTCGTCGAGTGCAATG 24
AAGGTTTGTCCTGGGTGACAG 32
CCACCACGTTGATAGGCCGGG 158
TGCCTTGGCATGCACAGGCGA 63
GGATGCCTACAAACAGTTGGA 45
CAGAACGTCGTGAGACAGTTC 263
CCATAGCTCAGCTGGGAGAGC 147
GATCGACTTGATCACTCCCAT 328
TAGTCGATGGGAACCACGTTA 21
CAAGGTAGCCGTAGGGGAACC 21
TGCCTACAAACAGTTGGAGCC 22
GTGATAGTCCCGTACACGTAG 28
GACCGTACCCTAAACCGACAC 839
CGAAAGGCGTAGTCGATGGGA 246
CGAGGGGAGTGAAACAGTACC 19
ACCTGGTGGTTATGGCGGACC 31
TGGGGAATATTGGACAATGGG 96
CGACCTGGTGGTTATGGCGGA 3949
GGATTTACTGGGCGTAAAGCG 2558
TGGGTTAAGTCCCGCAACGAG 18
GGCCGTAGCTCAGCTGGGAGC 19
AGTGGGTGATAGTCCCGTACA 26
ACGGGGTTGTTTGGCACCTCG 218
ATTTAGGTAGAGCGTCGACCG 86
CCCGGGTCTTGTACACACCGC 22
TGAGAGAACTGCGTTGAAGGA 5128
CTGGGCTACACACGTGCTACG 91
ATCTCTGGTGGACCTGTTGTG 117
TCCGACCTGCACGAATGGCGT 159
TGGTGGACCTGTTGTGGCGCC 344
CTCGGCCGTGAAACGTTCCAG 14
TGAAGCTTACCGGTACTAATA 90
TTTCACCGGTGAAGATAATGA 24
GTTGGTTAGAGCACACGCTTG 12
GGTGAAGTCGAAACAAGGTAG 18
AGCTTTACACTGGCATTCGTG 111
ATGACTTGTGGCTAGGGGCGA 13
TAGATCGGGATGACTTGTGGC 457
GGGCTGGGCTACACATGTGCT 13
AAATGATCGGCCCGCGTTGGA 1935
TGACCCCCAAGAGTCCATATC 12
CTCACTGGACCATTACTGACA 63
AGTTCGATGCATTAGACCCGA 82
AAACTCAAAGGAATTGACGGG 172
TAGCTCAGCTGGGAGAGCGCC 26
AACTCGGCAAAATGCACGCGT 414

GAGGATGATCAGCCACACTGG 1207
GTGTAGGAATATTGACAGGAT 111
AACACAGGGCTCTGCGAAGTC 242
CCACATTGGGACTGAGACACG 33
AGCCATCTCAGTTCGGATTGC 130
GCACCTGCTTTGCAAGCAGGG 86
GTTTAGAACGTCGTGAGACAG 11
TGAGACAGTTCGGTCCCTATC 179
CAGAACGGTCGGAAATCGTTC 93
GCATGCAGCTTACCGGTACTA 125
CACGGACCAGACTCCTACGGG 28
CGGATCGCGTGTGTTGTGAGG 657
CTGGCGGCAGGCTTAACACAT 190
CGTCAAGTCCTCATGGCCCTT 59
CGCACGTAGGCGGACTTTTAA 52
AGTAATCGCGGATCAGCACGC 11
GACTTTGGTTTTCGGATCGAA 41
GGACCAGACTCCTACGGGAGG 20
GAGAGGAAGGTGGGGATGACG 30
GCAAGGGGAGCGGCAGACGGG 25
CGCATTAAACATTCCGCCTGG 578
GGTGGGGTAAAGGCTCACCAA 26
ACCTGGGGAACTGAAACATCT 4074
AGCCGGCCCCTAAGGCGAGGC 20
TTCTGGTCTGGAATGCAGCGC 10
GCGATGGTAGGCTCAGAACGG 318
AAGGAAGATCGAGAATTGGAA 46
GTCGGCAGACACACGGCGGGT 240
CCGTGAAACGCTCCAGCGCCC 57
CTATATACGGACGGGATAACC 159
ACGAGCATTTGCAGTCGAATG 35
GTAAAGGCTCACCAAGGCGAC 36
TAGAAGCGAACCTGGGGAACT 9792
TGCAGACCTGGCAGCGACCTA 15
GCTGGTCTGAGAGGATGATCA 24
TTAGTGTATCGAGCAAGCTTA 17
ACGTATCTCTGGTGGACCTGT 101
AGTTGGGCACTCTAAGGTGAC 26
TCGGCCGGGAAACGCTCCAGC 19
CTACGGAATAACTCAGGGAAA 173
CGTTCCCGGGCCTTGTACACA 79
ATAGGGTCTGACGCCTGCCCG 970
GTGTGGAAGTGCGGCAACGCA 194
ATAAAAGGTACTCCGGGGATA 60
GCGCAACCCTCGCCCTTAGTT 13
TAGTTTGACTGGGGCGGTCTC 48
CGCTCCAGCGCCAATGGTACT 14
GGAGGCGCTCGATGGTAGGCT 10
GCTTACCGGTACTAATAGCTC 218
GTAGAGCACTGGATGGGCTAT 19
ACCGGGGCTCAAGCCATACAC 2690
CTCACTGGACCATTACTGACG 4285
GGGAATATTGGACAATGGGCG 529
GCCCGGTAGCTCGTCAGGCTC 316

GAAACAGTACCTGAAACCGGA 55
CACCTTAGATGACTAGAAAAT 69
CCGTGAAACGCTCCAGCGCCT 16
TTACTGGGCGTAAAGCGCACC 12
AATTCGTAGATATTCGGAGGA 245
TAGAAGCGAACCTGGGGAACC 29
TAACAGGCTGATGACCCCCAA 341
CGCAAGACGACGTATAGGGTC 29
GAGGACCGAACCCATATCTGT 29
AATGTACCGGGGCTCAAGCCA 253
GGGGGTAGAGCACTGGATGGG 110
ATGAAGGACGTGATACGCTGC 51
GAAGGGACAGTCGTGAGACAT 22
TTCAAGTCCTCCCAGGCCCAC 168
AGTCAGGGGTGAAATCCCGGG 36
ACCTGGGGAACTGAAACATCC 57
GAGCTGAGTTTTGATGGATAT 279
ACAATGGTGGTGACAGTGGGC 107
CAGAGGAAAGGACATCAAACG 21
GCGAACAGGATTAGATACCCT 11
GGTCGGAAATCGTTCGTCGAG 192
CTACACTCGGAATTCCACTCA 11
GCCAATCAAACTTGGAGATAG 15
AAAGGGAGTGAGAGACTCCCT 30
TCCGAATGGGGAAACCCACCT 32
TGTACACACCGCCCGTCACAC 18
TAAAGGGAGTGAGAGACTCCC 25
AAGCGAGTCTGAACAGGGCGT 29
GTATAGGGCCTGACGCCTGCC 11
ACTCGACCGAAGTGGGTGATA 251
AGCTCCTGCATATAGACCGTA 324
GTCTCAAGACGCGGGAGAGTA 155
GCGAACCTGGGGAACTGAACC 57
ACTGATGAAAATGGATTGACT 10
CCATTACTGACGCTGAGGTGA 11
TGTTGGCTTAGAAGCAGCCAT 86
TCGATGCATTAGACCCGAAAC 25
ATGGTAGGCTCAGAACGGTCG 74
TTACCTGACTTTGGTTTTCGG 18
ATCCCATTCCGAACTCGGCCG 85
AAACAGTACCTGAAACCGGAT 188
AAGCGCACGTAGGCGGACTTT 57
CGGCCCAGACTCCTACGGGAA 43
CAAACTTGGAGATAGCTGGTT 166
CGCGGTAATACGAAGGGGGCT 407
ACGCTGGCGGCAGGCTTAACG 25
TAACACGCACTGGAGGACCGA 14
AGGGTATGGCTGTTCGCCATT 24
ATCCTGTCTGAACATGGGTCG 1657
CTCAGCTGGGAGAGCACCTGC 563
ATTTCCGAATGGGGAAACCCA 19
TGAGCAGGGTTAGCCGGCACC 15
CATCAAACGAGACTCCGCTAG 86
TGGGTGATAGTCCCGTACACG 17

GCCGTAGCTCAGCTGGGAGAG 8047
TCGGCCGTGAAACGCTCGAGC 10
GAGGCAGGCGACCACGGTAGG 148
TGCGGACTTTTACGAAAGTCT 10
CCATTACTGACGCTGAGGTGG 20
TAAGAGCTGAGTTTTGATGGA 284
GAACCACGTTAATATTCGTGG 18
CGGCCCAGACTCCTACGGGAG 2735
CAACTGGAGAGTTTGATCCTG 10
ACTTGGAGATAGCTGGTTCTC 849
CAGCTGGGAGAGCACCTGCTT 493
CCACGCCGTAAACGATGAATG 134
GTTGTTCGGATTTACTGGGCG 291
ATACGTTCCCGGGTCTTGTAC 13
GACCAGGGGGTAGCGACTGTT 76
GGAAGACCACCACGTTGATAG 13
CGTAGCTCAGCTGGGAGAGAA 10
CCCCGCAAGGGGAGCGGCAGA 57
GACACGTGAAATCCTGTCTGA 26
ATAGCTCCTGCATATAGACCG 105
ATGCAGCTTACCGGTACTAAT 43
TAGTCCCGTACACGTAGAATA 360
TCCTAACCAAACTCCGAATAC 81
AACACATGCAAGTCGAGCGCC 14
CGAACCTGGGGAACTGAAAAA 70
GACCACGGTAGGGTCAGCGAC 2166
TTCGTGACGACATGTGTAGGA 310
GCCGTAGCTCAGCTGGGAGAA 38
AGCTTACCGGTACTAATAGCT 161
TGCATGGTTGTCGTCAGCTCG 13
CGGGTCCAGGACCGTGTATGG 37
AAGGGCCATCGTTCAACGGAT 99
CTAACCAAACTCCGAATACCG 53
CCGCGTTGGATTAGCTAGTTG 21
GAACTGCCTTTGATACTGGAA 118
CCTGCCCGGTGCTGGAAGGTT 17
ACGGATTTGACCTTCGGGTTT 30
ACGCTGGCGGCAGGCTTAACA 785
TAGGCGGACTTTTAAGTCAGG 18
ATGAAGGCCTTAGGGTTGTAA 15
GAAGGCGCGCGATGGTAGGCT 12
CGGTCGCGGTTAGTGGAGACA 94
ACTGGACCATTACTGACGCTA 12
AGGATTAGATACCCTGGTAGT 68
GCGCACGTAGGCGGACTTTTA 87
TTAAGCCGGTAGGTGTAGGCG 77
GATAAAAGGTACGCCGGGGAT 863
AAAGTCGGTCATAGTGATCCG 134
TTTAATTCGAAGCAACGCGCA 24
CGGTCCCTATCTGCCGTGGGG 24
ATACCCTTTGATCCGACGATA 16
TGATCTTGCAGCGAAGCGGTT 226
GAGTAGGTCGCTGCCAGGTCT 6251
ATCAGCCACACTGGGACTGAG 55

TCGAGCAGAGACGAAAGTCGG 131
CTTAGATGACTAGAAAATCTG 17
AACAGTACCTGAAACCGGATG 797
ATGAGGGGCCGTAGCTCAGCT 222
CATGGGGGTGCGACTGATTAT 45
TGAGCAGGGTTAGCCGGCCCC 10105
TAGCGTTTGCGTCGGTATCTG 10
CAAAAGCCGTCTCAGTTCGGA 24
ACAATGAACTTTGGCGGACAC 16
CAAGGAGGCAGGCGACCACGG 83
AGACGACGTATAGGGTCTGAC 135
AGCCTGCCTGACTGCAAGACT 210
GGTACTAATAGCTCGATCGAC 5436
ACACCAACTTCGATCCGAAAA 41
GTGGCGAAGGCGGCTCACTGG 231
GGGCTAGCGTTGTTCGGATTT 145
CAATGGCATAAGCCTGCCTGA 48
AGTGGAATTCCGAGTGTAGAG 561
CGGCCGTGAAACGCTCGAGCG 10
CAGGATTAGATACCCTGGTAG 39
TATAATGGGTCAGCGACTTAG 68
AAAGCGCACGTAGGCGGACTT 77
TAGATCGCAGGCCAGTCAGCC 90
ACACGGACCAGACTCCTACGG 19
CGTGAGACAGTTCGGTCCCTA 162
AAAGCGGTACGTGAGTTGGGT 10
TAGTTTGACTGGGGTGGTCGC 22
TATAACGGTCCTAAGGTAGCG 10
GATGGGCTATGGGGACTCACC 2688
ACATAGGGGTTAGAAGCGAAC 127
CTGCAAGACTGACAAGTCGAG 47
TTCCTTGTCGGGTAAGTTCCG 244
CTGCCGCCAGCGTTCGTTCTG 39
ACGTTAATATTCGTGGGCCTG 103
TCCTGGCTCAGGACGAACGCT 16
GCGTTGGATTAGCTAGTTGGT 10
GCTAGTAGTGGCGAGCGAACG 66
GGGGCAACCCACCTTAGATGA 26
GGAAAGACCCCGTGCACCTTT 51
ATGTGCGTGGTAGCGGAGCGT 23
TCCCGCGTGGAAGGGCCATCG 10
ACTGGACCATTACTGACGCTG 20454
CTAGCCATGAGCAGGTTGAAG 28
GGTCCTAAGGTAGCGAAATTC 73
CCGACCTGCACGAATGGCGTA 143
GCCATCTCAGTTCGGATTGCA 93
GAGTGATGAAGGCCCTAGGGT 26
GTTCGATGCATTAGACCCGAA 56
GTAGAGGTGAGTGGAATTCCG 103
TGTGGCTAGGGGTGAAAGGCC 587
TTGTGAGTAGTTGGGGGTGGT 12
TTCCCTATCAGAGCCGTGGAA 38
GCCCGGTGCTGGAAGGTTAAG 19
GTGCATGGCTGTCGTCAGCTC 16

GGTTAGCCGGCCCCTAAGGCG 100
AGGTACTCCGGGGATAACAGG 87
TTGCGGACTTTTACGAAAGTC 14
TTAATATTCGTGGGCCTGCAG 32
AGCCTGCCTGACTGCAAGACC 19
GCCTGCCTGACTGCAAGACTG 101
GTATCTCTGGTGGACCTGTTG 49
CGGGCTGGGCTACACACATGC 11
CAGACCAGGGGGTAGCGACTG 108
GGCTGGACCGGAGACAGGTGC 695
TCAGAACGGTCGGAAATCGTT 293
GGAGGCGCGCAATGGTAGGCT 10
CTGGCTCAGAACGAACGCTGA 18
GGGGTGGAGCAGCTCGGTAGC 10
AAATCCTGTCTGAACATGGGT 205
TTAGGCGGAAGAGATTTTGGA 44
CCAGACTCCTACGGGAGGCAG 1000
CATACACCGAAGCTGTGGATG 25
AAAAGGTACGCCGGGGATAAC 211
AAGACCCCGTGCACCTTTACT 55
TAATCGCGGATCAGCATGCCG 110
CAGGTGGTGACGGATCGCGTG 14
CAGAACGTCGTGAGACAGTTG 10
GCGAACGCGGACCAGGCCAGT 2648
AGCTCAGCTGGGAGAGCACCC 13
ATTCCCCGTGAAGATGCGGGG 13
AGACAGGTGCTGCATGGCTGT 69
AGACGGAAAGACCCCGTGCAC 337
GTACTGGAGGACCGAACCCAT 2666
AACGCAGACTCAGTGAAATTG 99
AATTCCGAGTGTAGAGGTGAA 445
GATCCCAAAACAACCAGGATG 68
GTTGTCGTCAGCTCGTGTCGT 13
GACGCGGGAGAGTAGGTCGCT 919
CGGAGGCGCGCGATGGTAGGA 58
TTTACCAAAAACACAGGGCTC 18
AAGGAACTCGGCAAAATGCAC 531
CTCAAGCCATACACCGAAGCT 307
AGCGTTGTTCGGATTTACTGG 26
GCTGGAGCAGGTCCCAAGGGT 16
TCCAACGCAGACTCAGTGAAA 97
CTGGGCTACACACGTGCTACC 264
CCGCGGTGAATACGTTCCCGG 205
CGCGTTGGATTAGCTAGTTGG 12
ATGGTACTTCGTCTCAAGACG 137
TAAGCCGAGAGGAAGGTGGGG 14
TGGCGAAGGCGGCTCACTGGA 354
CGACTTAGTGTATCGAGCAAG 114
GACGGAAAGACCCCGTGAACC 15
TCCGAGTGTAGAGGTGAAATT 467
TGGCCCTTACGGGCTGGGCTA 29
GTCGACCGAATACCCCCGGGG 5187
ACGATCCATAGCTGGTCTGAG 30
TTGGACAATGGGCGCAAGCCT 1709

ATCCTGGCTCAGGACGAACGC 11
TCATGTTGGTGTTGAGACGGA 22
CAGAACGTCGTGAGACAGTTA 12
GGGATGGACGTATCTCTGGTG 736
AGCTCAGCTGGGAGAGCACCT 2622
GGAACTGCCTTTGATACTGGA 161
GACATCAAACGAGACTCCGCT 335
TCCAAAAGCCATCTCAGTTCG 62
TTTGAGAGGATGATCAGCCAC 17
AAGGCGCTGTGCTAACCGCAA 18
CTGCCCGGTGCTGGAAGGTTA 14
AAAGCGAGTCTGAACAGGGCG 18
GAATTCCGAGTGTAGAGGTGA 268
CGACCTGGTGGTTATGGCGGC 10
CTGGCTCAGAACGAACGCTGG 3373
GGCTAGCGTTGTTCGGATTTA 123
ACAGGGCGTTCAGTTCGATGC 364
TCGGCCCATGTGGGCCGCCCC 21
CTGGGCTACACACGTGCTACT 42
GAACTCGGCAAATTGCACGCG 14
TATCCTTCAGTTAGGCTGGAC 12
TGGGCACTCTAAGGGGACTGC 977
TGGGCCGTGAAACGCTCCAGC 14
GGCCGTAGCTCAGCTGGGAGA 10311
GGTGACAGTGGGCAGCGAGCA 308
TTAAACATTCCGCCTGGGGAC 33
TTAGCCGTCGGGGTGTTTACA 183
GTGAAACGCTCCAGCGCCAAT 1072
ACCGTCTTACTGATCCTAACC 163
GGGTGAGTAACGCGTGGGAAC 18
TGACATCCCGGTCGCGGTTAG 156
ACCACGTTAATATTCGTGGGC 10
TATGTGCGTGGTAGCGGAGCG 27
GAACCTTACCAGCCCTTGACA 387
CGGAGGCGCGCGATGGTAGGG 27
GGTGCGAAAGCGTGGGGAGCA 10
AGCAGTGGGGAATTTTGGACA 49
TGGTTTTACCCGAAGGCGCTG 201
TTAGAACATAGATCGCAGGCC 29
CAAGGCGACGATCCATAGCTG 194
TAGATACCCTGGTAGTCCACG 277
CTCACTGGACCATTACTGACC 149
GCAGAAGTCCTTGAGTAGGGC 744
GAGGGGCCGTAGCTCAGCTGG 143
GGAAGTGAGAATGCTGACATG 103
AGAAGAAGCCCCGGCTAACTT 21
AAGGGAGTGAGAGACTCCCTC 41
AAGGTCCCTAAGTTATGGCTA 178
GCCCATCAGGGCCGACGGCCG 40
ATGGTTGTCGTCAGCTCGTGT 24
CGACTGTTTAGCAAAAACACA 13
ATGGCTAAGTGGGAAAGGATG 127
GTATCTCGAGAAGCTGGTCTT 11
CAATGAGAGTGATCAAGTGTC 12218

ACGGCCCAGACTCCTACGGGA 6099
CCTGTCTGAACATGGGTCGAC 2168
GTGAGAATGCTGACATGAGTA 335
AAGCTGTGGATGCACGTATGT 41
GTGAGTTGGGTTCAGAACGTC 18
TCGACTCATCGCATCCTGGGG 482
CAACCCACCTTAGATGACTAG 33
ACCTGGGGAACTGAAACATCG 17
CGAAAGTCGGTCATAGTGATC 142
TAGGCTCAGAACGGTCGGAAA 248
CGGCTCATCGCATCCTGGGGC 16
GGCACAGACCAGGGGGTAGCG 12
GGGAGTACGGTCGCAAGATTA 102
TAGAAGCGAACCTGGGGAACG 21
TAGAGCACTGGATGGGCTATG 23
TTACTGGGCGTAAAGCGCACG 1941
GTGGGTGTAGGAATATTGACA 65
AGTGTATCGAGCAAGCTTAAG 21
AGGACGTGATACGCTGCGATA 31
CGGAAAGACCCCGTGCACCTT 101
CCGTGAAACGCTCCAGCGCCA 11395
TTAGATACCCTGGTAGTCCAC 238
ACAAAGGCAAAGAACAGGCGC 46
GGCTAAGTGGGAAAGGATGTG 126
GCGAAGGCGGCTCACTGGACC 650
GCAGGTTGAAGGTACGGTAAC 932
GCCATGAGCAGGTTGAAGGTA 436
TAAACATTCCGCCTGGGGAGT 377
GGCTGCACCCGATCCCATTCC 14
ACGGCCCAGACTCCTACGGGG 15
GTTCGGATTTACTGGGCGTAA 44
CGTACCCTAAACCGACACTGG 639
ACTGGAGGACCGAACCCATAT 429
AGTTGGTTTTACCCGAAGGCG 34
CTCACTGGACCATTACTGACT 30
CAGAAGTCCTTGAGTAGGGCG 739
GGCATAAGCCTGCCTGACTGC 293
CTCAAGACGCGGGAGAGTAGG 239
ACGGAATAACTCAGGGAAACT 246
GGACTCACCGTCTTACTGATC 469
TTACTGGGCGTAAAGCGCACA 14
CCTTGAGTAGGGCGGGACACG 20
TGACGCCTGCCCGGTGCTGGA 526
CCGTGAAACGCTCCAGCGCCG 23
TTAAGAGCTGAGTTTTGATGG 265
CGTGAGTTGGGTTCAGAACGT 21
ATCCAGCCATGCCGCGTGAGT 12
ACCTGGGGAACTGAAACATCA 130
AGAATAGCAGAAGTCCTTGAG 472
AACAACCCTGACCACCATCTA 15
TGAAACTCAAAGGAATTGACG 11
AAGCCCCGGCTAACTTCGTGC 147
TAGCTTTACACTGGCATTCGT 88
AGAGATTTTGGACGGTTTAGA 15

CCCGCGTTGGATTAGCTAGTT 47
ATTTGGTTGCGGGGGCAGGAT 56
AGGATGTTGGCTTAGAAGCAG 1130
GTGCATGACCGATAGCGAACC 288
ATGATCCGCCACACTGGGACT 25
GATACCCTGGTAGTCCACGCT 23
CGGAGCGGCTGCACCCGATCC 1950
CGGCCCAGACTCCTACGGGAC 49
ACGAAAGTCGGCCATAGTGAT 10
GCGGTAATACGAAGGGGGCTA 205
AGGAATTGACGGGGGCCCGCA 16
CTGAAGGCCGCAGGTTCAAAT 17
TTAAGTCCCGCAACGAGCGCA 32
GACAAGTCGAGCAGAGACGAA 16
CCATTACTGACGCTGAGGTGC 15363
AGGCGCTGTGCTAACCGCAAG 26
CGGAGAAGAAGCCCCGGCTAA 96
TTAACACATGCAAGTCGAGCG 29
GGCCCATCAGGGCCGACGGCC 42
GCTAGTAATCGCGGATCAGCA 146
CTCCAAAAGCCATCTCAGTTC 55
GCAAGCCTGATCCAGCCATGC 221
GGAGTGAGAGACTCCCTCGCC 41
GGTGAGTAACGCGTGGGAACG 18
TAGAGCATACCAAGGCGCTTG 10
GCGACTGGGGTGAAGTCGTAA 12
ACCGGGGAGTACTAGTCGGCA 808
TAGCTCGATCGACTTGATCAC 41
TAGGTGTAGGCGCAGCGAAAG 38
GACCAGACTCCTACGGGAGGC 15
AACGCATGAAGCTTACCGGTA 61
CGGTTAGTGGAGACACTATCC 40
AAGCCATCTCAGTTCGGATTG 98
CCTGGCTCAGGACGAACGCTG 13
GGTGCTGGAAGGTTAAGAGGA 15
CGTGCATGACCGATAGCGAAC 316
GGGCTGGGCTACACACGTGGT 28
GTTGGGTTAAGTCCCGCAACG 24
ACAGGATTAGATACCCTGGTA 34
CTGAAACCGGATGCCTACAAA 147
AGGATGTTGGCTTAGAAGCAA 36
AACGCATTAAACATTCCGCCT 459
GATACCCTGGTAGTCCACGCC 363
GCCGCGGTGAATACGTTCCCG 197
AAAGTCCAAGGGTTCCTGCTT 15
CTTCGTGCCAGCAGCCGCGGT 23
AAGTCGGAATCGCTAGTAATC 290
AGAAGCCCCGGCTAACTTCGT 62
TAAGTGGGAAAGGATGTGAGG 101
GTTTGAGAGGATGATCAGCCA 12
CGTGAGACATCCTGGAGGTAT 111
GCCGTAGCTCAGCTGGGAGAC 15
TGGTTGCGGGGGCAGGATTTG 33
GAGAGGATGATCAGTCACACT 14

GGAAAACTCGACCGAAGTGGG 17
GAAGCGTGACCTCACTATGGG 192
ATCTGTTGCAATAGATCGGGA 27
TCAGGGCCGACGGCCGGTCGG 23
AACCTGCGGCTGGATCACCTC 31
ACGCTGGCGGCAGGCTTAACC 27
ACGTAGAATAGCAGAAGTCCT 1633
ATGACCCCCAAGAGTCCATAT 12
GGGTTCAGAACGTCGTGAGAC 141
CAATGGTACTTCGTCTCAAGA 21
TGCAGGTGGTGACGGATCGCG 79
TCGGATCAACTGAAGAGTTGA 14
TGAAGGCCCTAGGGTTGTAAA 1509
TCTAAGGTCCCTAAGTTATGG 381
GCAAGACGACGTATAGGGTCT 37
TGAAGGAACTCGGCAAATTGC 10
GGGTTAGAAGCGAACCTGGGG 812
CGGTCCCTATCTGCCGTGGGT 1339
AACTGGAGAGTTTGATCCTGG 12
ATCTCCAAAAGCCATCTCAGT 10
GGCGGACTTTTAAGTCAGGGG 68
TCGAGAAGCTGGTCTTTCTGC 306
CCGGTACTAATAGCTCGATCG 5477
GCCGACCTGGTGGTTATGGCG 11
ACTGGACCATTACTGACGCTC 10
ACCTGCACGAATGGCGTAACG 100
GGTACTTCGTCTCAAGACGCG 109
CGGTCGCGGTTAGTGGAGACC 11
AGAAGCGAACCTGGGGAACGG 14
TCCTTGTCGGGTAAGTTCCGA 225
ACTTGATGAGGGGCCGTAGCT 170
AAGGGGGCTAGCGTTGTTCGG 676
CAACGGATAAAAGGTACTCCG 39
GAGTAGGTCGCTGCCAGGTCG 18
TAAAGCGCACGTAGGCGGACC 12
TTAAGTCAGGGGTGAAATCCC 23
ATACCCTTTGATCCGACGATC 14
CCATGAGCAGGTTGAAGGTAC 423
CATGTGTAGGATAGGTGGTAG 971
AGCAGAGACGAAAGTCGGTCA 35
GAGATAGCTGGTTCTCCGCGA 23
GAGGGGAGTGAAACAGTACCT 18
GAAATCCTGTCTGAACATGGG 90
GATAGTCCCGTACACGTAGAA 63
CGGCCGTGAAACGTTCCAGCG 14
CCTGGTAGTCCACGCTGTAAA 25
TGAGTAGTTGGGGGTGGTTTT 11
CCATTACTGACGCTGAGGGGC 88
GACTGGGGTGAAGTCGTAACA 20
GACGGTAACCGGAGAAGAAGC 170
GGTTAGCCGGCCCCTAAGGCC 11
GGGCTTGTAGCTCAGTTGGTT 117
GTGAATACGTTCCCGGGCCTT 167
CGTACGGCGCGTGAGCGAGAA 13

AGGGTTGTAAAGCTCTTTCAC 1626
TTGAAGCAGGGGCGCCAGCCT 262
TACCAAGGCGCTTGAGAGAAC 98
CCGGGGATAACAGGCTGATGA 10
AACGACTTCCCCGCTGTCTCC 74
ATCGCTCAACGGATAAAAGGT 135
GCGTACCTTTTGTATAATGGG 11
TATAATGGGTCAGCGACTTAT 14
AACTGCGTTGAAGGAACTCGG 1123
GGGGGTCGTCGGTTCGATCCC 512
GCGGGGTTCCTGCGGTTAGAC 665
TAAAGCGCACGTAGGCGGACT 247
GTAGAGGTGAAATTCGTAGAT 182
ATACCCTTTGATCCGACGATT 1859
GGGCAACCCACCTTAGATGAC 26
CGGTCCCTATCTGCCGTGGGC 13
CACTGGTCTAAATAAGGGTCT 12
ATTGGACAATGGGCGAAAGCC 36
CCTGCCTGACTGCAAGACTGA 84
TCTCATGTTTGTGTTCTTCGC 21
ACTGGATGGGCTATGGGGACT 3002
GGCGAAGGCGGCTCACTGGAC 430
GATAAAAGGTACGCCGGGGAA 10
TCTGAGAGGATGATCAGTCAC 12
ACTGGACCATTACTGACGCTT 11
ATCGTGAAGAGAAGATGTAAT 25
CGGGCTGGGCCACACACGTGC 13
TTTGATACTGGAAGTCTTGAG 33
CGAAAGCGTGGGGAGCAAACA 12
TTCCCTATCAGAGCCGTGGAC 11
CTAGGTCGTCGGCCCATGTGG 21
CTAAGGGGACTGCCGGTGATA 163
GGCTGGACCGGAGACAGGTGA 11
AGGATCCCAAAACAACCAGGA 70
GGGCCGTACCGCAGCTGACGC 93
AATAGATCGGGATGACTTGTG 173
GCACACGCTTGATAAGCGTGG 11
GAACTGCGTTGAAGGAACTCA 10
CGGGGAGTACTAGTCGGCAGA 335
GGAATTCCGAGTGTAGAGGTG 404
CGAGAGGACCGGGATGGACGG 19
GTCCTCATGGCCCTTACGGGC 118
GCAGCTTACCGGTACTAATAG 45
GTCGTGAGACATCCTGGAGGT 27
GTCGGGGTGTTTACACTTCGG 66
AGGCGGAAGAGATTTTGGACG 17
TTCGAAGCAACGCGCAGAACC 70
GAAACCAAGTGATCTAGCCAT 11
ATAAAGTGGAACGAGTTGGAA 10
GGGCGGCATTGTCTGCGGATC 16
GGCCGGGAAACGCTCCAGCGC 19
GTGGTTGACAGGTTGGTTTGA 10
CGGTTAGACGGAAAGACCCCG 49
AGTGATGAAGGCCCTAGGGTT 34

TCCCGCAACGAGCGCAACCCT 95
CTTACCGGTACTAATAGCTCG 265
GGGGAACCTGCGGCTGGATCA 43
GGACCGGGATGGACGTATCTC 9421
GGTACCCCGGGGATAACAGGC 59
CAGGATGTTGGCTTAGAAGCA 2044
GTCGGGGAGGTGCGAATACCC 313
TAGCAGAAGTCCTTGAGTAGG 86
AAGCGAACCTGGGGAACTGAC 74
GGTCGTCGGCCCATGTGGGCC 17
TTAATTCGAAGCAACGCGCAG 25
CCCACCTGAAAACGAGTATTC 10
ACCTTACCAGCCCTTGACATC 133
GGAGGCAGGCGACCACGGTAG 139
GCCGTAGGGGAACCTGCGGCT 29
CGTAGGGGAACCTGCGGCTGG 29
TTGGTGGATGCCTTGGCATGC 47
GTACCGGGGCTCAAGCCATAC 808
ACGGAGGCGCGCGATGGTAGC 18
CATTGGGACTGAGACACGGCC 42
ACTATAGCTTTACACTGGCAT 15
TGGTGGATGCCTTGGCATGCA 37
GCGGGAGAGGAGGTCGCTGCC 13
GTAAAGCTCTTTCACCGGTGG 12
AGAGAACTGCGTTGAAGGAAG 10
AAGGAATTGACGGGGGCCCGC 24
GGATTGCACTCTGCAACTCGA 334
GGTGAAAAGCACCCCGACGAG 35
AGGGCGGCGGCGCCGGCAGCG 41
CCATTACTGACGCTGGGGTGC 16
TCTCTTTCTTCATTGTTGATT 22
GGTAGTTTGACTGGGGCGGTC 156
GTAATCGCGGATCAGAATGCC 11
CAGGATGTTGGCTTAGAAGCG 28
CCGCAAGGGGAGCGGCAGACG 55
GAACTGCGTTGAAGGAACTCG 1101
CTGTCCCTAGTACGAGAGGAC 99
CCGGAACTGCCTTTGATACTG 159
CGAGAGGACCGGGATGGACGA 32
GCGTTCCGTAAGCCTGTGAAG 17
ACTCGGCCGGGAAACGCTCCA 18
GATAGCTGGTTCTCCGCGAAA 93
GGTAGTTTGACTGGGGTGGTC 13
GGGGCTGTAGCTCAGCTGGGA 412
GACGTATAGGGCCTGACGCCT 12
GACTTGTGGCTAGGGGTGAAA 921
CAAGACGCGGGAGAGTAGGTC 334
GTAATCGGATCAACTGAAGAG 18
GTAGGTGTAGGCGCAGCGAAA 62
CGCCTGGGGAGTACGGTCGCC 12
GTAAAGCTCTTTCACCGGTGA 994
GTCAGCGACTGGGGTGAAGTC 411
AGCCCAAGGTTTGTCCTGGGT 14
AGAGAACTGCGTTGAAGGAAA 30

AAGCAGGGGGTCGTCGGTTCC 24
CGGCCGTGAAACGCTCCAGCG 35314
AAGCGAACCTGGGGAACTGAT 17
TAAGTCCCGCAACGAGCGCAA 79
CCAGTGTAGAATGTCTGCGCA 67
ATGTGAGGATCCCAAAACAAC 24
GGCTGTTCGCCATTTAAAGCG 26
AGAGTGATCAAGTGTCTTAAG 213
GCGGGTAGTTTGACTGGGGCG 12
GGCCCGCGTTGGATTAGCTAG 104
GGGGAGTTTGACTGGGGCGGT 192
TTGTACACACCGCCCGTCACA 19
CCACCTTAGATGACTAGAAAA 69
ATCGGCCCGCGTTGGATTAGC 270
TACGCTGCGATAAGCGTCGGG 56
TGGCGTAACGACTTCCCCGCT 13
AATCGCGGATCAGCATGTCGC 20
ATTTCCGAATGGGGCAACCCA 10
CGACCACGGTAGGGTCAGCGA 2121
TGCCGCGGTGAATACGTTCCC 212
CGTACCTTTTGTATAATGGGT 14
ATAGCTGGTTCTCCGCGAAAT 254
TGCATATAGACCGTACCCTAA 182
GTGACGACATGTGTAGGATAG 154
CAACCAGGATGTTGGCTTAGA 21
AGTGGCGAAGGCGGCTCACTG 301
ATGATCGGCCCGCGTTGGATT 365
AGCTGAGTTTTGATGGATATT 262
TCAGTTAGGCTGGACCGGAGA 41
ACAATGGGCGCAAGCCTGATC 194
TGGTGACAGTGGGCAGCGAGC 202
GGCCTTAGGGTTGTAAAGCTC 29
GGCGGAAGAGATTTTGGACGG 15
GGGTAGAGCACTGGATGGGCT 39
CGGAGGCGCGCGATGGTAGCC 12
AACTGCCTTTGATACTGGAAG 52
TAGCGAACCAGTACCGTGAGG 99
GGAATAACTCAGGGAAACTTG 88
ATGGGGGTGCGACTGATTATA 40
AAGTCCTTGAGTAGGGCGGGA 746
GGAGGCGCGCGACGGTAGGCT 10
GATAGCGAACCAGTACCGTGA 118
CGACGTTCTGAACCCAACTCA 10
TTGACTGGGGCGGTCTCCTCC 26
AGGGGGTAGCGACTGTTTACA 27
ATGCCGCGTGAGTGATGAAGG 24
TGCCCGGTGCTGGAAGGTTAA 16
GGACCGAACCCATATCTGTTG 20
CGTAGATATTCGGAGGAACCC 22
TCCTGGCTCAGAGCGAACGCT 10
GTCGACCACGATCCAAGCCTA 18
TCGGAAGAACACCAGTGGCGA 43
ACCTGGTGGTTATGGCGGAGC 4741
TACTGGAGGACCGAACCCATA 2092

TTACCGGTACTAATAGCTCGA 141
GAGCATACCAAGGCGCTTGAG 10
GATGAAGGCCTTAGGGTTGTA 12
GGACACGTTTCTTGGTAAGAA 57
AAACTTGGAGATAGCTGGTTC 158
AGCGTAACAGCTCACTGGTCT 301
GGAGGAACACCAGTGGCGAAG 1536
TGAGAGACTCCCTCGCCGAAA 24
GAGGCGCGCGATGGTAGACTC 15
CGACCACGATCCAAGCCTAAG 34
TGCAGCGAAGCGGTTCCAGGA 85
GATACGGCCCAGACTCCTACG 15
ATCTGAGCAGGGTTAGCCGGC 496
GAAGGGCCATCGTTCAACGGA 55
CTGGGTTCAGAACGTCGTGAG 29
ATGAAGTTGGAATCGCTAGTA 100
TACCTGAAACCGGATGCCTAA 12
GGGGCTCAAGCCATACACCGA 1292
GGTTGCGGGGGCAGGATTTGA 28
GGGGTTAGAAGCGAACCTGGG 805
CGAGGCGCTGACACGGATTTG 84
ACTTTGAAGCAGGGGCGCCAG 596
AAACCGACACTGGTGGACTGG 22
CCTGCAGGTGGTGACGGATCG 117
CCGGGTGTGGAAGTGCGGCAA 503
CTGGTAGTCCACGCCGTAAAC 196
TTGAGTATGGTAGAGGTGAGT 29
TCAAGACGCGGGAGAGTAGGT 309
TGCAAGCAGGGGGTCGTCGGT 90
ATAGTGAACCAGTACCGTGAG 33
AGGCGCTTGAGAGAACTGCGT 60
GTAGTGGCGAGCGAACGCGGA 81
CAAAGGCAAAGAACAGGCGCA 41
CTGACGCTGCTGGCCCTGCGC 25
CCGCAGACGAGGCGCTGACAC 14
ATTCCTTGTCGGGTAAGTTCC 294
CCTGGTAGTCCACGCCGTAAA 201
TCGTGCCAGCAGCCGCGGTAA 20
CTTCCCCGCTGTCTCCAACGC 424
GTAGAGCGTCGACCGAATACC 1563
GCTTGTAGCTCAGTTGGTTAG 123
CCGGGGGTAGAGCACTGGATG 190
ACACACGTGCTACAATGGTCG 10
TTGGATTGATCTTGCAGCGAA 22
CTGTCGTCAGCTCGTGTCGTG 77
CTGATGACCCCCAAGAGTCCT 13
CGAAGCGGTTCCAGGAAATAG 10
AGCGGGAAACCCACCTGAAAA 13
CTCGATGTCGACTCATCGCAT 27
GATTGCACTCTGCAACTCGAG 336
GTCCACGCCGTAAACGATGAA 32
AGAAGCTGGTCTTTCTGCTGA 13
TCGCAGGCCAGTCAGCCTGAC 10
GGACCTGTTGTGGCGCCAGCC 3933

ACGCATGAAGCTTACCGGTAC 62
ACAGGATCTGTCCCTAGTACG 214
TATAGCTTTACACTGGCATTC 13
ATGAATGTTAGCCGTCGGGGG 86
GCGGGGGCAGGATTTGAACCT 14
CTTGACATCCCGGTCGCGGTT 221
GACTCATCGCATCCTGGGGCT 440
ATTCCGCCTGGGGAGTACGGT 2328
ATACCCTGGTAGTCCACGCCG 277
AGCTCGTGTCGTGAGATGTTG 218
GAATACCAAGGCGCTTGAGAG 58
GAGATACGGCCCAGACTCCTA 12
GCGGGATGGAGCAGCCCGGTA 15
AGCGAACCTGGGGAACTGAAG 33
GTAGTCCACGCCGTAAACGAT 55
CCTGTGAAGGGACAGTCGTGA 45
GTAGCGACTGTTTACCAAAAA 12
GCCCCGGCTAACTTCGTGCCA 80
ACGGACGGGATAACCGCTGAC 27
ATCAGAGCCGTGGAAGACCAC 16
TGCCTGACTGCAAGACTGACA 78
ATTAAAACTCAAAGGAATTGA 56
GCCGTGAAACGCTCCAGCGAC 17
CATCTAAGGTCCCTAAGTTAT 12
CAGACTCAGTGAAATTGAATT 80
AGATTAAAACTCAAAGGAATT 72
GACCACCACGTTGATAGGCCG 127
TCTCGAGAAGCTGGTCTTTCT 241
CTGACATGAGTAACGATAAAG 16
CGGATAAAAGGTACCCCGGGG 79
TATCGGCAAATGATCGGCCCG 218
AACCAAACTCCGAATACCGGG 38
GTAGCTCAGCTGGGAGAGCGC 19
ACAGGATCTGTCCCTAGTACA 16
CTGCACGAATGGCGTAACGAC 41
ATACATAGGGGTTAGAAGCGA 11
CAATGGTGGTGACAGTGGGCA 96
TTGGCGGACACGTTTCTTGGT 121
GCAACAACCCTGACCACCATC 48
TGCTACGGAATAACTCAGGGA 273
AACTCGGCCGTGAAACGCTCC 6123
GCAAAATGCACGCGTAACTTC 30
CTTGAGAGTTTGATCCTGGCG 20
ACATCTAAGTACCCAGAGGAA 180
CGGGCTGGGCTATACACGTGC 19
CCTTAGATGACTAGAAAATCT 19
ACGTACTGGAGGACCGAACAC 11
ACGCATTAAACATTCCGCCTG 1950
GGTTAGGCGGAAGAGATTTTG 11
CTGATGACCCCCAAGAGTCCC 39
AGCGAAATTCCTTGTCGGGTA 1171
GATTTCCGAATGGGGAAACCC 19
ACACCGCCCGTCACACCATGG 47
TTCTAAGGAAGATCGAGAATT 40

TAGAATAGCAGAAGTCCTTGA 1147
AGAGGGCAACAACCCTGACCA 17
ATCGGCAAATGATCGGCCCGC 1555
CGTGACCTCACTATGGGCAAC 15
CGGTCGCAAGATTAAAACTCA 171
GATGTCGGCTCATCGCATCCT 13
GCACAGACCAGGGGGTAGCGA 40
TGGCATTCGTGACGACATGTG 11
TCTCCAAAAGCCATCTCAGTT 54
AGCGAACGCGGACCAGGCCAG 1666
CGGGGCTCAAGCCATACACCG 1554
AGCGAACCTGGGGAACTGAAA 17565
GGCACTCTAAGGGGACTGCCG 252
AGTCGAGCGCCCCGCAAGGGG 65
CTCGGCCGTGAAACGCTCCAC 17
GGAGCCCAAGGTTTGTCCTGG 56
CCAAGGGTATGGCTGTTCGCC 2294
TTCAGTTGGGCACTCTAAGGG 133
CAAACTCCGAATACCGGGGAG 15
AGGATGATCAGCCACACTGGG 1863
TAACCGCTGAAGGCATCTAAG 15
CTTCGGGGGAAAGATTTATCG 23
GGGGTTCCTGCGGTTAGACGG 468
ACAAGGTAGCCGTAGGGGAAC 28
TAAGTACCCAGAGGAAAGGAC 757
GTACCCTAAACCGACACTGGT 479
ACCAAGGCGACGATCCATAGC 340
GGGCTGGGCTACACACGTGCA 29
CGTAAACGATGAATGTTAGCC 235
GCGAACCTGGGGAACTGAAGC 23
AATTCCTTGTCGGGTAAGTTC 171
AGAGCACCTGCTTTGCAAGCA 10
TGTTCGGATTTACTGGGCGTA 150
TAGATATTCGGAGGAACACCT 35
AGTACCTGAAACCGGATGCCT 6527
ACCTCGATGTCGACTCATCGC 28
ACGGTCGGAAATCGTTCGTCG 298
GGGACTCACCGTCTTACTGAT 458
TTACACACCAACTTCGATCCG 14
CAAGAGTCCATATCGACGGGG 14
GTTCAAATCCTGCCCCCGCAA 20
CTAACTTCGTGCCAGCAGCCG 16
GTCAAGTCCTCATGGCCCTTA 59
ACCCTAAACCGACACTGGTGG 546
GGAGTACGGTCGCAAGATTAA 126
TGTCGGGTAAGTTCCGACCTG 60
TTCGATCCCGTCCGGCTCCAC 1686
AAGAGAAACGTGGGCGGCATT 12
GAATGTTAGCCGTCGGGGTGT 445
GCGAAAGCGAGTCTGAACAGG 24
TAGAGGTGAAATTCGTAGATA 97
CTTTCACCGGTGAAGATAATG 12
TACCAAAAACACAGGGCTCTG 16
AGAGGTGAAATTCGTAGATAT 72

ATCGAGAATTGGAAAGAGGCA 14
GTCAGCGACTTAGTGTATCGA 172
AGTCCTCATGGCCCTTACGGG 116
ATGTTAGCCGTCGGGGTGTTT 66
CGGAAATCGTTCGTCGAGTGC 90
CGTAGATATTCGGAAGAACAC 43
TAGATATTCGGAGGAACACCC 35
TTTAGGTAGAGCGTCGACCGA 136
AAACGGCGGCCGTAACAATAA 11
GGACCATTACTAACGCTGAGG 10
TACTAGTCGGCAGACACACGG 15
TGAATGTTAGCCGTCGGGGGG 25
CTGGATGGGCTATGGGGACTC 2959
GGGGTGGAGCAGCCCGGTAGC 208
GTCCCTATCTGCCGTGGGTGT 256
GACTTAGTGTATCGAGCAAGC 113
GGGCTGGGCTACACACGTGCG 155
CCACCTGAAAACGAGTATTCC 10
TACGGAATAACTCAGGGAAAC 159
GGAGACACTATCCTTCAGTTA 13
ACCTGCGGCTGGATCACCTCC 126
TGAAGCAGGGGCGCCAGCCTT 92
TAGCGACTGTTTACCAAAAAC 17
TCGGCCGTGAAACGTTCCAGC 14
CGAAGTGGGTGATAGTCCCGT 297
CGGAGGCGCGCGATGGGAGGC 11
GAGCAGAGACGAAAGTCGGTC 126
CGATCCATAGCTGGTCTGAGA 14
CTATTTAGGTAGAGCGTCGAC 25
GCGGACCAGGCCAGTGGCTTT 884
AGATCGCAGGCCAGTCAGCCT 69
TCGGCAAAATGCACGCGTAAC 59
CCACGTTGATAGGCCGGGTGT 18
GTGGCTAGGGGTGAAAGGCCA 273
CTGGGCTACACACGTGATACA 12
AATGCGTACGGCGCGTGAGCG 11
ACGCTGCGATAAGCGTCGGGG 90
CGAAGGGGGCTAGCGTTGTTC 561
AGCTGGTTCTCCGCGAAATCT 235
TTTTATTTGGTTGCGGGGGCA 36
TGAAATTGAATTCCCCGTGAA 10
AAGGAGGCAGGCGACCACGGT 89
GAACTCGGCAAAATGCACGCG 452
TAGTAATCGCGGATCAGAATG 11
TCGTGCAGGTCGGAACTTACC 11
TTAGCCGGCCCCTAAGGCGAG 41
GGGGCCATAGCTCAGCTGGGA 1434
CTCGGCAAATTGCACGCGTAA 10
CATTACTGACGCTGAGGCGCG 18
AACCCACCTTAGATGACTAGA 40
GCATTGTCTGCGGATGGTTCG 38
TTTGTTGGAGCAACGCTGGAT 47
CGAGAGGACCGGGATGGACGT 4691
TATATACGGACGGGATAACCG 85

AGTGAACCAGTACCGTGAGGG 36
AGAACCTTACCAGCCCTTGAC 916
GGGCTATGGGGACTCACCGTC 1406
CGCGTAGAACCTTACCAGCCC 12
CGGGGAGTACTAGTCGGCAGC 18
TTATGGATGTCTAACTGCGGC 52
CCAGGGGGTAGCGACTGTTTA 1922
ACGTAGGCGGACTTTTAAGTC 50
GGTGTGGAAGTGCGGCAACGC 255
AAGCCTGCCTGACTGCAAGAC 256
AGGTCGTCGGCCCATGTGGGC 19
TCGGCCCGCGTTGGATTAGCT 465
ACATTGGGACTGAGACACGGC 43
AGTAGTGGCGAGCGAACGCGG 68
CGTCGGGGAGGTGCGAATACC 515
GGGCGGCATTGTCTGCGGATA 17
GATAAAAGGTACCCCGGGGAT 71
TCTGCAACTCGAGTGCATGAA 15
TACTGATGAAAATGGATTGAC 10
CCAGTCAGCCTGACGATCGCT 37
ACGGGCTAGGCTACACACGTG 15
AATGGGCGCAAGCCTGATCCA 103
CTCGTGTCGTGAGATGTTGGG 255
GCTGTGGATGCACGTATGTGC 358
CGGCCGTGAAACGCTCCAGCC 14
ACCAGCCCTTGACATCCCGGT 91
GTCGGTTCGATCCCGTCCGGC 533
AAGCGAACCTGGGGAACTGAA 7005
AAAGACCCCGTGCACCTTTAC 44
AAGCAGGGGGTCGTCGGTTCG 359
TTTCTAAGGAAGATCGAGAAT 12
CCGGTCGCGGTTAGTGGAGAC 221
CGTAGTCGATGGGAACCACGT 60
ACGGAGGCGCGCGATGGTAGA 43
TGGGGTAAAGGCTCACCAAGG 26
AAAGGCGTAGTCGATGGGAAC 270
CGTATGTGCCCTTCGGGGGAA 49
GCAGACGAGGCGCTGACACGG 15
GTGTAGAGGTGAAATTCGTAG 108
CAGGATGTTGGCTTAGAAGCT 27
GTCCCTAGTACGAGAGGACCG 42
GGGCGGCATTGTCTGCGGATG 3407
TCAAATGAATTGACGGGGGCC 17
TGAGTGGAATTCCGAGTGTAG 283
ACTCTTGGGGGTCATCAGCCT 20
TTGTAAAGCTCTTTCACCGGT 786
GAAGTGAGAATGCTGACATGA 111
TTTCTCTTTCTTCATTGTTGA 20
CGCAGAACCTTACCAGCCCTT 3866
GCCTTGGCATGCACAGGCGAT 159
CTTAGGGTTGTAAAGCTCTTT 14
CACGCACTGGAGGACCGAACC 30
GTGCGAATACCCTTTGATCCG 20
ATCTGTCCCTAGTACGAGAGG 80

AGACATCCTGGAGGTATCGGA 117
TCCGCCACACTGGGACTGAGA 20
AAAGGTACTCCGGGGATAACA 80
CATAGATCGCAGGCCAGTCAG 224
AGAGAACTGCGTTGAAGGAAC 4416
CAATAACGGTCCTAAGGTAGC 21
GGACGGGATAACCGCTGAAGG 2330
GGGAACCACGTTAATATTCGT 23
CGCCTGGGGAGTACGGTCGCA 2767
ACGGAGGCGCGCGATGGTAGG 37882
TAATCTGAGCAGGGTTAGCCG 119
ATCGAAGTTGGTGTGTAAATA 16
GACTTGTGGCTAGGGGTGAAC 25
GGGCGCCAGCCTTTGTGGAGT 10
GTATAGGGTGTGACGCCTGCC 12
GGCATCTAAGCGGGAAACCCA 50
ATCGACTTGATCACTCCCATT 609
GAACTTTGGCGGACACGTTTC 19
TGTGTAGGATAGGTGGTAGAC 996
CTATCTGCCGTGGGTGTAGGA 114
AAGCGAACCTGGGGAACTGAG 45
CGCCATCACCGATTGTATCTC 16
TTGTTCGGATTTACTGGGCGT 191
GACTCCGCTAGTAGTGGCGAG 12
GAAAGACCCCGTGCACCTTTA 43
TCTGACGCCTGCCCGGTGCTG 1211
CCAAAAGCCATCTCAGTTCGG 70
ACTTTGGTTTTCGGATCGAAG 21
AACTCGGCAAATTGCACGCGT 14
GACGGGGGCCCGCACAAGCGG 184
AGTTGGAATCGCTAGTAATCG 366
TGAGGATCCCAAAACAACCAG 29
GGGACTGCCGGTGATAAGCCA 53
TGGAGCAGCCCGGTAGCTCGT 186
CGACGATTTCCGAATGGGGCA 31
ATACGTTCCCGGGCCTTGTAC 181
GGTCGGAGGTTCAAGTCCTCC 30
CTGGAGGACCGAACCCATATC 406
CGATAAAGGGAGTGAGAGACT 659
TAAAAGGTACTCCGGGGATAA 64
CCCGAAGGCGCTGTGCTAACC 225
TTGATTTGTGAGTAGTTGGGG 11
GGCAAATGATCGGCCCGCGTT 2423
GGAGGAACACCAGTGGCGAAC 28
CGAACCAGTACCGTGAGGGAA 144
GCGTTGTTCGGATTTACTGGG 348
AATGACGGTAACCGGAGAAGA 34
AGAGGATGATCCGCCACACTG 16
GGCCGTAAAACGCTCCAGCGC 12
GTTTGGCACCTCGATGTCGAC 145
ACACGCTTGATAAGCGTGGGG 16
TGCTGACATGAGTAACGATAA 12
CATGAGCAGGTTGAAGGTACG 393
AGGCTCACCAAGGCGACGATC 76

AGATATTCGGAGGAACACCCG 11
GTACGGCGCGTGAGCGAGAAC 10
GAACTCGGCCGTGAAACGCCC 11
GACTGTTTACCAAAAACACAG 15
GAAGGCCTTAGGGTTGTAAAG 16
TCGTGGGCCTGCAGGTGGTGA 535
GAATATTGACAGGATCTGTCC 150
TCGAAGCAACGCGCAGAACCT 27
GGGGTAAAGGCTCACCAAGGC 31
GAAGGACGTGATACGCTGCGA 53
CTTTGCAGACCTGGCAGCGAC 33
AGGATGTGAGGATCCCAAAAC 24
CCGATAGCGAACCAGTACCGT 157
TCGGCCGTAAAACGCTCCAGC 14
AGGGGGTAGCGACTGTTTACC 1741
ACTCTAAGGGGACTGCCGGTG 195
AAATGCACGCGTAACTTCGGA 89
CTGCCGGTGATAAGCCGAGAG 63
ACTGATCTAGAAGCCCGGCAC 54
CAGCCCTTGACATCCCGGTCG 84
ACGGTAACCGGAGAAGAAGCC 1143
GCCCCTAAGGCGAGGCCGAAA 13
TTAGGCTGGACCGGAGACAGG 545
ATCCCGGGGCTGGAGCAGGTC 10
TGAGAGACTCCCTCGCCGAAC 11
CGGGGTTGTTTGGCACCTCGA 191
TGTCGGCTCATCGCATCCTGG 15
AGAACTGAAACATCTAAGTAC 19
TGTTTAGCAAAAACACAGGGC 11
GGTAAGTTCCGACCTGCACGA 12
AGAAGTCCTTGAGTAGGGCGG 746
ACCGAAGTGGGTGATAGTCCC 331
TGTAGAGGTGAAATTCGTAGA 116
GGGACTGCCGGTGATAAGCCG 3083
GGGCAGTTTGACTGGGGCGGT 14
CGCGTGAGTGATGAAGGCCCT 17
CTAACTGCGGCCCGTTATCCG 11
TCGAGCGCCCCGCAAGGGGAG 75
GCGGCGGCGCCGGCAGCGGCA 31
TCAAGCCATACACCGAAGCTG 276
ATCGGGATGACTTGTGGCTAG 893
TACCTGAAACCGGATGCCTAC 5192
CTGTTTAGCAAAAACACAGGG 13
AGACGACGTATAGGGCCTGAC 22
GCAGAGACGAAAGTCGGTCAT 103
ACGATTTCCGAATGGGGCAAC 19
AAGGCAATATGGAAGTAGGGC 25
GAAAGCGTGGGGAGCAAACAG 12
AGTGAAATAGTACCTGAAACC 25
GCACTCTAAGGGGACTGCCGG 277
GGTACGCCGGGGATAACAGGC 92
TACCCTGGTAGTCCACGCCGT 293
CTTGAGAGTTTGATCCTGGCC 12
GGAAGTCTTGAGTATGGTAGA 45

AACTCGGCCGTGAAACGCTCG 13
ATATTGGACAATGGGCGCAAG 572
GTGAACCAGTACCGTGAGGGA 30
ATAGGTGGTAGACTTTGAAGC 66
ATGAATGTTAGCCGTCGGGGT 6609
CCAACGCAGACTCAGTGAAAT 110
GTGAGACAGTTCGGTCCCTAT 142
ACTGTTTGTTGATTTGTGAGT 28
ACGGACGGGATAACCGCTGAA 2428
GCATGCCGCGGTGAATACGTT 209
CTCGGCCGTGAAACGCTCCAG 32888
GCTAAGTGGGAAAGGATGTGA 156
GTGAAGTCGGAACAAGGTAGC 20
GAAAGTCGGTCATAGTGATCC 119
GGTTTAGAACGTCGTGAGACA 14
AGCGAACCTGGGGAACTGAAT 31
GATTAGATACCCTGGTAGTCC 75
TAGGGTCTGACGCCTGCCCGG 1175
TGGTGACGGATCGCGTGTGTT 19
GATGCCTTGGCATGCACAGGC 33
GAGCGCCCCGCAAGGGGAGCG 72
AAGGCGGCTCACTGGACCATT 375
GGGAGAGTAGGTCGCTGCCAG 4335
TTTGGACAATGGGCGCAAGCC 59
CTGCAGGTGGTGACGGATCGC 107
AGTCCATATCGACGGGGTTGT 63
GAAGGCAATATGGAAGTAGGG 21
TTGCGTCGGTATCTGGGCTTG 47
TAGTCATCATAAATAAGGTAT 37
ACAGGATCTGTCCCTAGTACC 21
GGCGAGCGAACGCGGACCAGG 49
AGCGCACGTAGGCGGACTTTT 62
ATGAATGTTAGCCGTCGGGGC 38
GAACGCTGGCGGCAGGCTTAA 1800
GCTCACTGGTCTAAATAAGGG 20
TCAGTTCGATGCATTAGACCC 154
CGGGCTGGGTTACACACGTGC 10
AGCATTCAGTTGGGCACTCTA 13
CTGCATATAGACCGTACCCTA 241
GTGGAGCAGCCCGGTAGCTCG 172
GCACGTATGTGCGTGGTAGCG 57
CTGATGACCCCCAAGAGTCCA 265
TGACGTCAAGTCCTCATGGCC 48
CAGCGACTGGGGTGAAGTCGT 12
TTGAGTAGGGCGGGACACGTG 36
CCAGCGCCAATGGTACTTCGT 52
TCGGTCATAGTGATCCGGTGG 621
CTGTAGCTCAGCTGGGAGAGC 85
CTTGAGAGTTTGATCCTGGCT 2201
ACATCTAAGTACCCAGAGGAC 73
GGACAATGGGCGAAAGCCTGA 11
CCAATCAAACTTGGAGATAGC 15
GTTTGACTGGGGCGGTCGCCT 212
TAAGAGGAGAGGTGCAAGCCT 130

GGCTACACACGTGCTACAATG 122
CGCATCCTGGGGCTGGAGCAG 456
TTCAGTTGGGCACTCTAAGGT 26
CGAGCTGGGCTACACACGTGC 12
CTCGGCCGTGAAACGCTCCAA 13
AGGGCCATCGTTCAACGGATA 47
CTAGTAATCGCGGATCAGCAT 142
GGCCCGCACAAGCGGTGGAGC 151
AGCGAACCTGGGGAACTGAAC 99
GGGTGTGGAAGTGCGGCAACG 303
AGCGAGTCTGAACAGGGCGTT 49
GGGCTGGGCTACACACGTGCC 139
TCAGCCTGACGATCGCTTGCA 10
TCGGTATCTGGGCTTGTAGCT 101
ACCCACCTTAGATGACTAGAA 51
GCGAGCGAACGCGGACCAGGC 1761
AGTAACGGAGGCGCGCGATGG 90
CTTTGCAAGCAGGGGGTCGTC 98
GAAACATCTAAGTACCCAGAG 50
ACTCTCCCGCGTCTTGAGACG 34
CCAAGGCGACGATCCATAGCT 263
ATGCCGCGGTGAATACGTTCC 238
CAACCCTCGCCCTTAGTTGCC 13
AGTACCTGAAACCGGATGCCG 23
TAGATATTCGGAGGAACACCG 110
AAGAGGAGAGGTGCAAGCCTT 126
ACCCCGGAACTGCCTTTGATA 147
GACGAAAGTCGGCCATAGTGA 10
GTAGCTATATACGGACGGGAT 50
GGCCGTGAAACGCTCCACCGC 11
CACGTAGAATAGCAGAAGTCC 603
GCGCGATGGTAGGCTCAGAAC 1322
ACGCTGCGATAAGCGTCGGGC 18
GAGGAAAGGACATCAAACGAG 21
CGAGCAAGCTTAAGCCGGTAG 116
CGCCACACTGGGACTGAGACA 19
TTTGACTGATCTAGAAGCCCG 22
CCCGGTGCTGGAAGGTTAAGA 21
ATCGAGAATTGGAAAGAGGCC 186
CATGGGTCGACCACGATCCAA 987
GCCTAAGTACTCGTGCATGAC 269
GGTTATGGCGGAGCGGCTGCA 182
GGGCTGGGCTACACACATGCT 11
TATTGACAGGATCTGTCCCTA 248
CATGAAGCTTACCGGTACTAA 88
CAAGTGATCTAGCCATGAGCA 92
CGAGAAGCTGGTCTTTCTGCT 305
TAGATATTCGGAGGAACACCA 3737
TAGGAATATTGACAGGATCTG 793
TCCTGGAGGTATCGGAAGTGA 21
GGGGACTCACCGTCTTACTGA 421
GGGCTGGGCTACACACGTGCT 49173
GCCGCAGGTTCAAATCCTGCC 13
AATACGTTCCCGGGCCTTGTA 45

GAGAATTGGAAAGAGGCCGGA 71
TTGGACAATGGGCGAAAGCCT 24
CCGGGCCTTGTACACACCGCC 268
GCGGCAGGCTTAACACATGCA 116
ATTGGGACTGAGACACGGCCC 37
CGTAAAGCGCACGTAGGCGGA 329
GGACGTGATACGCTGCGATAA 28
TATTCGGAAGAACACCAGTGG 38
AACATTCCGCCTGGGGAGTAC 268
CCAGCCACACTGGGACTGAGA 13
TCGTCGAGTGCAATGGCATAA 35
GGATAAAAGGTACTCCGGGGA 46
GCGGACCAGGCCAGTGGCTTG 10
GCCAATGGTACTTCGTCTCAA 32
AAGGTACGCCGGGGATAACAG 120
GTGGCTAGGGGTGAAAGGCCC 20
ACAATAACGGTCCTAAGGTAG 19
CTCCAACGCAGACTCAGTGAA 83
AGTGGGGAATTTTGGACAATG 47
CAAGTCGAGCGCCCCGCAAGG 92
TCAACCCCGGAACTGCCTTTG 27
CTGGGCGTAAAGCGCACGTAG 210
TTGAGAGAACTGCGTTGAAGG 4827
AGGGGGTCGTCGGTTCGATCC 771
CGGAGGCGCGCGACGGTAGGC 10
GAATTGACGGGGGCCCGCACA 18
TGACAGTGGGCAGCGAGCACG 369
TAGGTCGTCGGCCCATGTGGG 21
AAAGGAATTGACGGGGGCCCG 31
TAAGTACTCGTGCATGACCGA 85
TGGGCACTCTAAGGTGACTGC 25
GGCGACCACGGTAGGGTCAGC 2032
ACAACCAGGATGTTGGCTTAG 23
ACCGCCATCACCGATTGTATC 16
AGGCAAAGAACAGGCGCAGCC 10
GAGGAACACCAGTGGCGAAGG 1127
CGAAATTCCTTGTCGGGTAAC 60
AATACCCTTTGATCCGACGAT 850
GCACCTCGATGTCGGCTCATC 26
TGCAGCTTACCGGTACTAATA 24
CGGTAGCTCGTCAGGCTCATA 72
CGGAGGAACACCAGTGGCGAC 58
TGCTTTGCAAGCAGGGGGTCG 90
AGTGAAACAGTACCTGAAACC 27
CCGAAGCTGTGGATGCACGTA 68
CAGTGAAATTGAATTCCCCGT 35
ATATTCGGAAGAACACCAGTG 38
ATGTCGACTCATCGCATCCTG 178
AGTACTCGTGCATGACCGATA 24
CGGGGTAGAGCAGCCCGGTAG 10
TACGAGAGGACCGGGATGGAC 382
AATGATCGGCCCGCGTTGGAC 16
TCGATGGGAACCACGTTAATA 25
TGGCTCAGAACGAACGCTGGC 3307

CTGGAAGTCTTGAGTATGGTA 32
TGACTGGGGCGGTCGCCTCCT 128
CATATAGACCGTACCCTAAAC 276
CGAACTCGGCCGTGAAACGCA 22
TGACGACATGTGTAGGATAGA 26
GTGGGGTCGGAGGTTCAAGTC 3002
TGTCGTCAGCTCGTGTCGTGA 82
AGGCCTTAGGGTTGTAAAGCT 19
TCAAATCCTGCCCCCGCAACC 35
TGATTTGTGAGTAGTTGGGGG 13
CTCTGGTGGACCTGTTGTGGC 511
GGTCCCTATCTGCCGTGGGTG 396
AAGCCTGATCCAGCCATGCCG 173
CAGCCTGACGATCGCTTGCAG 10
AGCCCGGTAGCTCGTCAGGCT 458
ACACGGATTTGACCTTCGGGT 29
CAGCTCACTGGTCTAAATAAG 17
GATGTGAGGATCCCAAAACAA 21
ATCTTGCAGCGAAGCGGTTCC 99
ACTCAAATGAATTGACGGGGG 16
GTGCAATGGCATAAGCCTGCC 34
CAACGGATAAAAGGTACGCCG 41
GGAAGGGCCATCGTTCAACGG 20
GACTGAGACACGGCCCAGACT 176
CCGATAGTGAACCAGTACCGT 27
AGCAGTGGGGAATATTGGACA 10
GGTAACCGGAGAAGAAGCCCC 886
CGCAAGGGGAGCGGCAGACGG 44
TCGGGGAGGTGCGAATACCCT 30
AATGATCGGCCCGCGTTGGAT 522
AAACGATGAATGTTAGCCGTC 1889
CGACTGTTTACCAAAAACACA 15
ACAGTTCGGTCCCTATCTGCC 1321
GGTAGACTTTGAAGCAGGGGC 296
AACGGTCCTAAGGTAGCGAAC 12
GGATGTTGGCTTGGAAGCAGC 11
GAAGGCCCTAGGGTTGTAAAG 3462
GAATGCTGACATGAGTAACGA 62
CGATAGTGAACCAGTACCGTG 35
TGTTGTGGCGCCAGCCGCATA 1692
AGTAATCGCGGATCAGAATGC 11
CAGTGGGCAGCGAGCACGCGA 393
GTCCTAAGGTAGCGAAATTCC 76
TTTGCAGACCTGGCAGCGACC 61
GTTCGATCCCGTCCGGCTCCA 1046
AGGCGATGAAGGACGTGATAC 91
GCGTCTCCGTGTTTTACATGG 20
TCAAGTCCTCCCAGGCCCACC 185
CGGTCGGAAATCGTTCGTCGA 271
TTCGTAGATATTCGGAAGAAC 46
TGACGACATGTGTAGGATAGG 173
GGTTGTAAAGCTCTTTCACCG 204
ACTCTGCAACTCGAGTGCATG 142
TGGGAAAGGATGTGAGGATCC 98

GGTCGACCACGATCCAAGCCT 189
GTGACAGTGGGCAGCGAGCAC 483
ATTCAGTTGGGCACTCTAAGG 83
CCTGGGGAACTGAAACATCCA 12
GACACGGACCAGACTCCTACG 12
GCCTTTGATACTGGAAGTCTT 15
ATCGCATCCTGGGGCTGGAGA 13
TTCGTAGATATTCGGAGGAAC 1935
CGGTGGTCCCGCGTGGAAGGG 39
ACGGGCTGGGCTACACACGTA 19
CGCGGACCAGGCCAGTGGCTA 14
TCTAACTGCGGCCCGTTATCC 16
AAACACGGAGACGCAAACTTC 15
CGATTTCCGAATGGGGCAACC 19
GCTAGGGGTGAAAGGCCAATC 27
ACCCACCTGAAAACGAGTATT 10
TAGACTTTGAAGCAGGGGCGC 1056
AGCCGCGGTAATACGAAGGGG 69
CCGACGATTTCCGAATGGGGA 22
CACTGGACCATTACTGACGCC 14
ATATGGAAGTAGGGCAATAAG 43
AGGAAAGGACATCAAACGAGA 23
AAGCGTGGGGTCGGAGGTTCA 1234
AAGGCGACGATCCATAGCTGG 40
GAGTGCATGAAGTTGGAATCG 84
CTGTCTGAACATGGGTCGACC 2001
GCCCTAGGGTTGTAAAGCTCA 16
ACTTCGTCTCAAGACGCGGGA 26
GACACAGGTGCTGCATGGCTG 21
ACTGCAAGACTGACAAGTCGA 47
CACGAATGGCGTAACGACTTC 14
AATAACTCAGGGAAACTTGTG 78
GGGACACGTGAAATCCTGTCT 22
TTGCAATAGATCGGGATGACT 86
TCCATATCGACGGGGTTGTTT 85
AACAGGATTAGATACCCTGGT 12
GATGACTTGTGGCTAGGGGGG 184
CAGTTAGGCTGGACCGGAGAC 36
CTGAGCAGGGTTAGCTGGCCC 13
TGAGACACGGCCCAGACTCCT 266
AGCAGGTCCCAAGGGTATGGC 20
ACGTTGATAGGCCGGGTGTGG 51
GACGACATGTGTAGGATAGGT 515
AAGGTAGCGAAATTCCTTGTC 216
GTTCGCCATTTAAAGCGGTAC 34
TGATCTAGCCATGAGCAGGTT 74
CGCGGGAGAGTAGGTCGCTGA 10
ATACGCTGCGATAAGCGTCGG 39
GTACTAATAGCTCGATCGACG 13
CAGGGGGTCGTCGGTTCGATC 832
GCCCTAGGGTTGTAAAGCTCG 14
GTAGTTTGACTGGGGCGGTCT 53
CCGAATACCGGGGAGTACTAG 31
ACGGGCTGGGCTACACACGTG 40025

TGCAAGACTGACAAGTCGAGC 20
CAGGTCCCAAGGGTATGGCTG 2497
CGCGGACCAGGCCAGTGGCTG 10
GGAGGCGCGCTATGGTAGGCT 24
GGCTGATGGTTTTTGTTTACC 19
GGCCGTGAAACGTTCCAGCGC 14
GTAGGTCGCTGCCAGGTCTGC 1479
GGCCCAGACTCCTACGGGAGG 2072
GCTATATACGGACGGGATAAC 197
CGACGAGGGGAGTGAAACAGT 17
AGGACATCAAACGAGACTCCG 337
CTGGGGCGGTCGCCTCCTAAA 31
CACTGGACCATTACTGACGCT 5441
GGTGGATGCCTTGGCATGCAC 28
AGTGGGCAGCGAGCACGCGAG 298
TTGATACTGGAAGTCTTGAGT 34
AGGATGATCCGCCACACTGGG 13
CGGCCCATGTGGGCCGCCCCC 20
ATCGCGTGTGTTGTGAGGTCT 267
GTTGAAGGCAATATGGAAGTA 13
AACAATAACGGTCCTAAGGTA 19
GGTCAGCGACTTAGTGTATCG 188
GAAGAAGCGTGACCTCACTAT 53
ACAGGCTGATGACCCCCAAGA 5244
TCCCCGTGAAGATGCGGGGTT 26
ACTGAGACACGGACCAGACTC 20
GGAATTGACGGGGGCCCGCAC 13
ACTCGTGCATGACCGATAGCG 294
TGATCAGCCACACTGGGACTA 10
TAAGTTCCGACCTGCACGAAT 16
GTGAAACAGTACCTGAAACCG 29
AACGGATAAAAGGTACTCCGG 34
GTGGTGACGGATCGCGTGTGT 13
CATTACTGACGCTGGGGTGCG 14
GGGCCCGCACAAGCGGTGGAC 13
TGACGGTAACCGGAGAAGAAG 94
GGTAGGTGTAGGCGCAGCGAA 67
CGTGGGTGTAGGAATATTGAC 57
AGATATTCGGAAGAACACCAG 24
AGAGGAGAGGTGCAAGCCTTG 162
CATGAGTAACGATAAAGGGAG 25
AAGCGGTACGTGAGTTGGGTT 12
AACGGATAAAAGGTACCCCGG 69
TGACCACCATCTAAGGTCCCT 29
TGGGGAGTTTGACTGGGGCGG 248
TCGATCGACTTGATCACTCCC 412
AGGGCGGGACACGTGAAATCC 20
TTCGTGCCAGCAGCCGCGGTA 22
AGTTGGGCACTCTAAGGGGAA 27
AAACAACCAGGATGTTGGCTT 51
GCAGCCGCGGTAATACGAAGG 53
TTTGCTACGGAATAACTCAGG 166
GCCACATTGGGACTGAGACAC 19
CCTGGGGAGTACGGTCGCAAG 844

ATGCACAGGCGATGAAGGACG 299
AAGCCGGTAGGTGTAGGCGCA 148
GAGAGAACTGCGTTGAAGGAC 10
AACTCAAAGGAATTGACGGGG 193
CACACCAACTTCGATCCGAAA 14
CACGTAGGCGGACTTTTAAGT 64
CCGTACACGTAGAATAGCAGA 393
GAAGATCGAGAATTGGAAAGA 1041
AGCCTAAGTACTCGTGCATGA 141
ATAGCTGGTCTGAGAGGATGA 76
GTGAAGATGCGGGGTTCCTGC 49
AAATTCGTAGATATTCGGAAG 25
CAGCCACACTGGGACTGAGAC 316
CGGATTTGACCTTCGGGTTTG 24
AAGACGACGTATAGGGTCTGA 56
CGACCTGCACGAATGGCGTAA 107
TGTTTTGTTGGAGCAACGCTG 19
GTAGGATAGGTGGTAGACTTT 51
GGACTGGTAGAGAATACCAAG 133
GAACGCGGACCAGGCCAGTGG 2527
TCGGGGTGTTTACACTTCGGT 55
AGTATTCCCTATCAGAGCCGT 18
TGATCAGCCACACTGGGACTG 3211
CGCGCGATGGTAGGCTCAGAA 1314
ACACTGGCATTCGTGACGACA 222
GCCAGTCAGCCTGACGATCGC 46
GGGCGTTCAGTTCGATGCATG 11
GGAACCTGCGGCTGGATCACC 28
TGGCTGTCGTCAGCTCGTGTC 263
ATTGGACAATGGGCGCAAGCC 1578
TATTCGTGGGCCTGCAGGTGG 171
ATATACGGACGGGATAACCGC 185
AAGACGACGTATAGGGCCTGA 17
AGAACTGCGTTGAAGGAACTC 1806
CCATCGCTCAACGGATAAAAG 88
GATATTCGGAAGAACACCAGT 28
AAGGCCCTAGGGTTGTAAAGC 5745
TTGGGTTAAGTCCCGCAACGA 20
CTATAGCTTTACACTGGCATT 13
GAAGTCGTAACAAGGTAGCCG 12
GGGTCAGCGACTGGGGTGAAG 350
TCGGCAAATTGCACGCGTAAC 10
AGCACCTGCTTTGCAAGCAGG 52
GGCGGGTAGTTTGACTGGGGC 18
TTCGGAAGAAGCGTGACCTCA 110
ACGAGCGCAACCCTCGCCCTT 41
TACCTTTTGTATAATGGGTCA 14
GGTTCCTGCGGTTAGACGGAA 258
GCCATACACCGAAGCTGTGGA 32
CCGACCTGGTGGTTATGGCGG 14
TGGGGCAACCCACCTTAGATG 31
ACTGGAGAGTTTGATCCTGGC 12
CAGTACCTGAAACCGGATGCC 6999
CGAATACCCTTTGATCCGACG 216

AGACAGTTCGGTCCCTATCTG 188
TGTGAGGATCCCAAAACAACC 27
GGGCTCAAGCCATACACCGAA 728
GAACGTCGTGAGACAGTTCGG 289
GTGAGACATCCTGGAGGTATC 145
CGTGTCGTGAGATGTTGGGTT 150
CTGGACCATTACTGACGCTAA 10
GGCGCTTGAGAGAACTGCGTT 41
GCTCAGAACGAACGCTGGCGG 2695
ACTGGGGCGGTCGCCTCCTAA 37
CGGGCCTTGTACACACCGCCC 23
GGAAGAGATTTTGGACGGTTT 10
ACTCGGCCGTGAAACGCTCCG 14
AGACCAGGGGGTAGCGACTGT 72
CAGCTCGTGTCGTGAGATGTC 10
GAGACGAAAGTCGGCCATAGT 10
GGCTGATGACCCCCAAGAGTC 4266
GTGAAATTCGTAGATATTCGG 285
GCGGGAGAGTAGGTCGCTGCC 10048
GAGATGTTGGGTTAAGTCCCG 43
GCTGAGTTTTGATGGATATTG 60
CTGGTGGACTGGTAGAGAATA 2462
AGCGAAAGCGAGTCTGAACAG 15
TTTTACGAAAGTCTGCCTGTT 17
GCTAGTTGGTGGGGTAAAGGC 41
AAGAAAGCGTAACAGCTCACT 33
ACATGGGTCGACCACGATCCA 1291
GCTGGTCTTTCTGCTGATACT 15
CGGATTGCACTCTGCAACTCG 342
GTGGGGAATTTTGGACAATGG 47
TCAGCTGGGAGAGCACCTGCT 545
CCAAACTCCGAATACCGAGGA 18
AGGAACTCGGCAAATTGCACG 21
AGACACGGACCAGACTCCTAC 10
ACATAGATCGCAGGCCAGTCA 333
ACGCTGAGGTGCGAAAGCGTG 71
ACTCGGCCGTGAAACGCTCCA 30375
GGGTTCCTGCGGTTAGACGGA 447
GCGGAAGAGATTTTGGACGGT 19
CAGCTCGTGTCGTGAGATGTT 234
GGGTAAAGGCTCACCAAGGCG 31
CTCAACGGATAAAAGGTACGC 83
CCCGTGAAGATGCGGGGTTCC 37
ACTTGTGGCTAGGGGTGAAAC 13
GGCCACACTGGGACTGAGACA 24
AGTCATCATAAATAAGGTATG 10
TGGACCATTACTGACGCTGAC 16
ACTCGGCCGTGAAACGTTCCA 13
GATTGATCTTGCAGCGAAGCG 84
TTGCTCACGGGCCGTACCGCA 16
GAGAACTGAAACATCTAAGTA 15
CACATTGGGACTGAGACACGG 41
AACACGTACTGGAGGACCGAA 330
GCAGGTGGTGACGGATCGCGT 70

GACCGATAGTGAACCAGTACC 12
CAACGCGCAGAACCTTACCAG 56
GCAAACCTGGGGAACTGAAAC 16
GGGCCATAGCTCAGCTGGGAG 1085
TACGTTCCCGGGCCTTGTACA 98
CCTCATGGCCCTTACGGGCTG 112
GTATGGTGGGTAGTTTGACTG 21
AGGAACACCAGTGGCGAAGGC 893
GACGGAAAGACCCCGTGCACC 364
CGGCCGTGAAACGCTCCACCG 11
ATGATCAGCCACACTGGGACT 5023
ACATCAAACGAGACTCCGCTA 116
GTCGCAAGATTAAAACTCAAA 144
AGCTGGGTTCAGAACGTCGTG 13
TCCCTATCAGAGCCGTGGAAG 54
CCTTGGCATGCACAGGCGATG 166
CCAAGGGTTTGGCTGTTCGCC 12
CAAGTCCTCATGGCCCTTACG 110
TCTGAGCAGGGGTAGCCGGCC 10
AAAGAAAGCGTAACAGCTCAC 10
GGATAAAAGGTACGCCGGGGA 1484
CCCTAAGGCGAGGCCGAAAGG 15
GTAGCCGTAGGGGAACCTGCG 29
CGGAGGAACACCAGTGGCGAA 2607
CTATCCTTCAGTTAGGCTGGA 12
CGAAATTCCTTGTCGGGTAAA 46
ATGTGTAGGATAGGTGGTAGG 12
TGGTAGAGGTGAGTGGAATTC 34
TCAAACTTGGAGATAGCTGGT 168
AGATCGAGAATTGGAAAGAGA 14
GGGCCGTGAAACGCTCCAGCG 13
GATCCTAACCAAACTCCGAAT 82
TGGCTCAGAACGAACGCTGGA 10
TCGACCGAAGTGGGTGATAGT 255
AAAGCTCTTTCACCGGTGAAG 678
TTCAGTTCGATGCATTAGACC 193
AGCAGCCGCGGTAATACGAAG 10
TCCGAATACCGGGGAGTACTA 26
CCGAGTGTAGAGGTGAAATTC 400
GCGTGACCTCACTATGGGCAA 16
ACTGGGCGTAAAGCGCACGTA 230
GACAGGATCTGTCCCTAGTAC 83
AGGCAGCAGTGGGGAATTTTG 23
ACCGATAGTGAACCAGTACCG 15
GAAGGAACTCGGCAAAATGCA 263
GACCGAATACCCCCGGGGGTA 1040
CCTGAAACCGGATGCCTACAG 11
CGAACTCGGCCGTGAAACGCC 48
ATGAAGTCGGAATCGCTAGTA 202
TCCTAAGGTAGCGAAATTCCT 81
CCATAGCTGGTCTGAGAGGAT 77
TACCCTGGTAGTCCACGCTGT 31
CAGTTCGGTCCCTATCTGCCG 993
AGACGAAAGTCGGTCATAGTG 137

GTTGTTTGGCACCTCGATGTC 175
AGATCGAGAATTGGAAAGAGG 586
GGCGCCAGCCTTTGTGGAGTC 14
ACCTGCTTTGCAAGCAGGGGG 88
GAGTAGGGCGGGACACGTGAA 27
CCGGATGCCTACAAACAGTTG 36
GCATGAAGTCGGAATCGCTAG 32
GTAGGCGGACTTTTAAGTCAG 19
CACCGCCCGTCACACCATGGG 36
ATGGCATAAGCCTGCCTGACT 177
GAGTGAGAGACTCCCTCGCCG 21
GGTGAAGTCGTAACAAGGTAG 50
TGCGGGGGCAGGATTTGAACC 18
CGGAGGAACACCAGTGGCGAG 85
TTCCGAATGGGGCAACCCACC 12
GGGGTGAAAGGCCAATCAAAC 13
GACAATGGGCGCAAGCCTGAT 254
CGAAATTCCTTGTCGGGTAAG 3305
GGTCAGCGACTGGGGTGAAGT 344
ACATCCCGGTCGCGGTTAGTG 57
GCCCAGACTCCTACGGGAGGC 1325
ATGTGTAGGATAGGTGGTAGA 1124
TCTAGCCATGAGCAGGTTGAA 23
AACGGTCCTAAGGTAGCGAAA 43
GACAGTGGGCAGCGAGCACGC 365
CCGTAGGGGAACCTGCGGCTG 29
CTTGAGTAGGGCGGGACACGT 32
GAATATTGGACAATGGGCGAA 21
CAAGATTAAAACTCAAAGGAA 81
GCTCGTGTCGTGAGATGTTGG 252
CTCTTGGGGGTCATCAGCCTG 20
TATTTGGTTGCGGGGGCAGGA 43
ACCGAAGCTGTGGATGCACGT 129
ATCGCATCCTGGGGCTGGAGC 283
TTGGTTGCGGGGGCAGGATTT 32
GTGACCTCACTATGGGCAACC 15
ATAGGGGTTAGAAGCGAACCT 83
CCTGAAACCGGATGCCTACAA 532
TGACGACATGTGTAGGATAGT 20
CGATGAAGGACGTGATACGCT 53
CCATTCCGAACTCGGCCGTGA 48
CGAACTCGGCCGTGAAACGCT 7282
GGACGTATCTCTGGTGGACCT 288
CACTGGACCATTACTGACGCA 11
CTGCGGTTAGACGGAAAGACC 55
GGGTTGTAAAGCTCTTTCACC 1582
GCATGGTTGTCGTCAGCTCGT 28
CCGACGATTTCCGAATGGGGC 168
CAATTGGCTGATGGTTTTTGT 20
CGGCCGTAAAACGCTCCAGCG 12
AATGGCATAAGCCTGCCTGAC 57
GAAGCCCCAGTAAACGGCGGC 10
CAAGCTTAAGCCGGTAGGTGT 15
ACGGGCTGGGCTACACACGTC 30

TTTGGTGGATGCCTTGGCATG 42
GAGAGTGATCAAGTGTCTTAA 216
TGATAGGCCGGGTGTGGAAGT 26
GCCCTAGGGTTGTAAAGCTCC 32
TGCATGACCGATAGCGAACCA 47
AAACACAGGGCTCTGCGAAGT 208
GTAGAATAGCAGAAGTCCTTG 1756
TTCCGAACTCGGCCGTGAAAC 1238
GCCGTGAAACGTTCCAGCGCC 12
GTACTAATAGCTCGATCGACC 26
GGCCCTTACGGGCTGGGCTAC 17
ATGTAATCGGATCAACTGAAG 10
CGCAGACGAGGCGCTGACACG 11
GTATTCCCTATCAGAGCCGTG 21
GGAGAGTAGGTCGCTGCCAGG 4230
AGAGTCCATATCGACGGGGTT 54
GGTGAGTGGAATTCCGAGTGT 53
TTTGTATAATGGGTCAGCGAC 128
GGGCTGCGCCTGTTCTTTGCC 56
CGTGAAGAGAAGATGTAATCG 22
GAAAGTCCAAGGGTTCCTGCT 13
ACACAGGGCTCTGCGAAGTCG 1109
GTGAAGTCGTAACAAGGTAGC 60
ACCAGGGGGTAGCGACTGTTT 1523
CTCGGCCGTGAAACGCTCGAG 12
CGTTGAAGGAACTCGGCAAAG 12
AACTATAACGGTCCTAAGGTA 15
AGCCATGAGCAGGTTGAAGGT 49
GAACGAACGCTGGCGGCAGGC 2894
GCCCTAGGGTTGTAAAGCTCT 6112
GTAGTTTGACTGGGGCGGTCG 254
TGGCGGACACGTTTCTTGGTA 119
GTTAATATTCGTGGGCCTGCA 101
CGCGGGAGAGTAGGTCGCTGC 5701
GTACTAATAGCTCGATCGACT 4899
CGTGAGATGTTGGGTTAAGTC 184
TCGGATTTACTGGGCGTAAAG 2558
CTCAAAGGAATTGACGGGGAC 22
AGAAGCTCTCCACTAAATCAG 12
TAAGGGGACTGCCGGTGATAA 182
CACTGGACCATTACTGACGCG 10
TTCCCGGGCCTTGTACACACC 229
TCCGCTAGTAGTGGCGAGCGA 56
CGGAACTGCCTTTGATACTGG 171
GGTAGCTCGTCAGGCTCATAA 72
CGCGGACCAGGCCAGTGGCTT 4371
CTTGAGAGAACTGCGTTGAAG 4295
TCAGTTGGGCACTCTAAGGTG 25
GGGCATTTGGTGGATGCCTTG 43
ACTCAGTGAAATTGAATTCCC 60
GAGGGCAACAACCCTGACCAC 144
GACGTGATACGCTGCGATAAG 21
GGGCTGGGCTACACACGTACT 15
TAACCGGAGAAGAAGCCCCGG 611

GTATGGTAGAGGTGAGTGGAA 23
AGCGCCCCGCAAGGGGAGCGG 76
CGTTGAAGGAACTCGGCAAAA 62
ACCGCCCGTCACACCATGGGA 48
CTAACACATGCAAGTCGAGCG 15
TAAGCCTGCCTGACTGCAAGA 231
GGCTGTCGTCAGCTCGTGTCG 75
GATAAAGGGAGTGAGAGACTC 133
CCTGCGGCTGGATCACCTCCT 102
TCGTCCGTGAAACGCTCCAGC 10
TGGAGCCCAAGGTTTGTCCTG 108
TGGGCAGCGAGCACGCGAGTG 67
TGTATCGAGCAAGCTTAAGCC 27
AAGTGCGGCAACGCATGCAGC 10
ACAGGCTGATGACCCCCAAGC 10
AAGGCCGCAGGTTCAAATCCT 15
TGGCGAGCGAACGCGGACCAG 43
TTCACCGGTGAAGATAATGAC 23
ACGGTCGCAAGATTAAAACTC 252
ATACAGGTGCTGCATGGCTGT 16
CAGCGACTTAGTGTATCGAGC 168
TGGGCTGCGCCTGTTCTTTGC 56
AGACGCGGGAGAGTAGGTCGC 728
GCAAGCAGGGGGTCGTCGGTT 778
CGGGCTAGGCTACACACGTGC 16
AAGTTCCGACCTGCACGAATG 14
AGGTACGCCGGGGATAACAGG 96
AGTTGGGCACTCTAAGGGGAC 1479
TATCGAGCAAGCTTAAGCCGG 16
GGCCGTACCGCAGCTGACGCT 50
TGGGTCAGCGACTTAGTGTAT 195
CGGGCTGGGCTACACACGTAC 18
CCCAAGAGTCCATATCGACGG 11
GGATGTTTGGTTAGGCGGAAG 49
GTAGATATTCGGAGGAACAAC 16
CCCTATCAGAGCCGTGGAAGA 31
TAGACGGAAAGACCCCGTGCA 331
GAGAGAACTGCGTTGAAGGAA 3127
CAGTACCTGAAACCGGATGCG 10
CAGTTGGGCACTCTAAGGGGA 1545
CATGGCTGTCGTCAGCTCGTG 1193
GCATAAGCCTGCCTGACTGCA 274
TCGAGTGCATGAAGTTGGAAT 251
TTGACATCCCGGTCGCGGTTA 162
ACCAGTGGCGAAGGCGGCTCG 13
GTCGGAATCGCTAGTAATCGC 215
TAACCAAACTCCGAATACCGG 39
CTGTGGATGCACGTATGTGCG 348
GGGTTAGCCGGCCCCTAAGGC 260
TATGGTAGAGGTGAGTGGAAT 24
GTGAGGATCCCAAAACAACCA 24
CGTTAATATTCGTGGGCCTGC 96
TCGCGGTTAGTGGAGACACTA 41
CGATCCCGTCCGGCTCCACCA 62

CTGAGATACGGCCCAGACTCC 10
CCAGCAGCCGCGGTAATACGA 23
AGAATGCTGACATGAGTAACG 111
ACACGGCCCAGACTCCTACGG 4888
TGAAACCGGATGCCTACAAAC 121
GGCAGGCGACCACGGTAGGGT 245
GTCGGTATCTGGGCTTGTAGC 83
CTGTTGCAATAGATCGGGATG 24
AGATACGGCCCAGACTCCTAC 12
GGACCAGGCCAGTGGCTTTTG 561
CAGTTCGATGCATTAGACCCG 105
GGGCCCGCACAAGCGGTGGAG 151
GCACTCTGCAACTCGAGTGCA 166
AAGTGGGAAAGGATGTGAGGA 102
ACGGAGGCGCGCGATGGTGGG 14
GTCTGAACAGGGCGTTCAGTT 10
GGGCGTTCAGTTCGATGCATT 378
CATAGGGGTTAGAAGCGAACC 115
CGCGCGATGGTAGGCTCAGAC 17
ATTCGTAGATATTCGGAAGAA 45
AGTGAGAGACTCCCTCGCCGA 29
CCGGTGCTGGAAGGTTAAGAG 20
GAGTGGAATTCCGAGTGTAGA 407
AGTCCCGCAACGAGCGCAACC 81
CCGCGGTAATACGAAGGGGGC 426
ACCAGTGGCGAAGGCGGCTCA 406
AAAGCGTAACAGCTCACTGGT 216
TGCCAGCAGCCGCGGTAATAC 15
GATCCGCCACACTGGGACTGA 20
ACTGCGTTGAAGGAACTCGGC 1498
TAATGACGGTAACCGGAGAAG 23
CTCCTGCATATAGACCGTACC 604
AGGGTAGCTATATACGGACGG 19
GGCTCAGAACGGTCGGAAATC 355
GGTAACACGTACTGGAGGACC 344
TTGCAAGCAGGGGGTCGTCGG 87
AGCCGGTAGGTGTAGGCGCAG 163
TGGTGGTTATGGCGGAGCGGC 2974
GGGCGCAAGCCTGATCCAGCC 436
CCGTACCGCAGCTGACGCTGC 17
TTGGCCGTGAAACGCTCCAGC 16
CAGTACCTGAAACCGGATGCA 20
TTGATGAGGGGCCGTAGCTCA 150
GGGCAACAACCCTGACCACCA 26
CCTGGGGAGTACGGTCGCAAC 13
AGGGTCTGACGCCTGCCCGGT 1293
TGTCCTGGGTGACAGCGTACC 56
AGCACCCCGACGAGGGGAGTG 32
ATGGGTCGACCACGATCCAAG 975
TTTATGGATGTCTAACTGCGG 17
CAGTTGGGCACTCTAAGGTGA 26
CGGTAGGTGTAGGCGCAGCGA 107
TGAAGTTGGAATCGCTAGTAA 94
GGGCTACACACGTGCTACAAT 219

TGGACCATTACTGACGCTGAG 18425
AGTCATCATAAATAAGGTATC 20
ACTTGTGGCTAGGGGTGAAAG 1009
GTGGATGCCTTGGCATGCACA 24
ACTGGGCCGTGAAACGCTCCA 13
ACTGGTTGGATGTTTGGTTAG 33
AGAACGAACGCTGGCGGCAGG 2308
GATGTCGACTCATCGCATCCT 102
CAGCGAGCACGCGAGTGTGAG 21
TGGGACTGAGACACGGCCCAG 122
AGCGACTGGGGTGAAGTCGTA 12
CACCACGTTGATAGGCCGGGT 156
ATTGATCTTGCAGCGAAGCGG 162
CGTGAGTGATGAAGGCCCTAG 19
AAGTCCAAGGGTTCCTGCTTA 18
CGATGGTAGGCTCAGAACGGT 251
GAAATCGTTCGTCGAGTGCAA 15
TAGGGGTGAAAGGCCAATCAA 12
CTGTGAAGGGACAGTCGTGAG 41
CTTGATAAGCGTGGGGTCGGA 102
GAGAGACTCCCTCGCCGAAAG 24
TCGATGTCGACTCATCGCATC 43
ACCATTACTGACGCTGAGGAG 10
GCCTGACTGCAAGACTGACAA 67
CTAAACCGACACTGGTGGACT 124
GGATTAGATACCCTGGTAGTC 63
CAACGAGCGCAACCCTCGCCC 100
GACGACGTATAGGGTCTGACG 205
ATCCCGGTCGCGGTTAGTGGA 116
AGAACGAACGCTGGCGGCAGA 16
GTTTTGTTGGAGCAACGCTGG 26
TGTTTGTTGATTTGTGAGTAG 27
CTGTTTGTTGATTTGTGAGTA 27
GGTAGCGACTGTTTACCAAAA 84
CCCAAGGGTTTGGCTGTTCGC 16
CTCACCAAGGCGACGATCCAT 350
GTTGGAGCAACGCTGGATGGG 33
GGGCTACACACGTGCTACAAC 25
CGGAGACAGGTGCTGCATGGC 270
CGGGGTGTTTACACTTCGGTG 29
TGGACCATTACTGACGCTGAA 63
GTAGGCTCAGAACGGTCGGAA 149
CGGGCTGTGCTACACACGTGC 10
CTTTGAAGCAGGGGCGCCAGC 552
GACGCCTGCCCGGTGCTGGAA 228
ACGAGTATTCCCTATCAGAGC 11
TGGACTGGTAGAGAATACCAA 202
ATGGGCAACCATAGGGGGGTG 34
TGGTGCATGGCTGTCGTCAGC 15
CACAGGGCTCTGCGAAGTCGC 1095
AGTTTGACTGGGGTGGTCGCC 22
ACGACGTATAGGGCCTGACGC 40
GTTAGAAGCGAACCTGGGGAA 2650
GTTAAGAGGAGAGGTGCAAGC 28

CAAAGGAATTGACGGGGGCCC 69
CAAATGATCGGCCCGCGTTGG 2032
CATAGCTGGTCTGAGAGGATG 70
GTAGGGGAACCTGCGGCTGGA 59
GTTCGTCGAGTGCAATGGCAT 37
GACTGGTAGAGAATACCAAGG 65
GCGATGAAGGACGTGATACGC 90
ATTAGATACCCTGGTAGTCCA 133
GACTGATCTAGAAGCCCGGCA 51
CTAAGTACTCGTGCATGACCG 246
CCGTAAACGATGAATGTTAGC 240
GCGGCTGCACCCGATCCCATT 15
CGCGGACCAGGCCAGTGGCCT 16
GCCCGCACAAGCGGTGGAGCA 33
TTCCGCCTGGGGAGTACGGTC 2593
TTACGGGCTGGGCTACACACG 1454
CGGGATGACTTGTGGCTAGGA 11
AAAGGCAAAGAACAGGCGCAG 42
AGGCGCGCGATGGTAGGATCA 16
CCAAAAGCCGTCTCAGTTCGG 22
CTCAAAGGAATTGACGGGGGC 206
TCTCCAACGCAGACTCAGTGA 71
GGCTGTAGCTCAGCTGGGAGA 249
TGCGTTGAAGGAACTCGGCAC 16
GAAGAGATTTTGGACGGTTTA 10
TACTGACGCTGAGGTGCGAAA 3228
ACGCGGACCAGGCCAGTGGCA 39
CAACCCTGACCACCATCTAAG 33
TACACACCAACTTCGATCCGA 14
CGGAGGCGCGCGATGGTGGGC 18
GGAGGCGCGCGATGGTAGGCT 36784
ACGGATAAAAGGTACCCCGGG 78
TTTGAACCTGCGGCCTTCAGG 111
GCGTGGGGTCGGAGGTTCAAG 5676
ATTTATCGGCAAATGATCGGC 36
TCCTGGCTCAGAACGAACGCT 3299
AAGGACGTGATACGCTGCGAT 45
GACTCAGTGAAATTGAATTCC 59
TTGTATAATGGGTCAGCGACT 194
GACTGTTTATCAAAAACACAG 10
ACGCGGGAGAGTAGGTCGCTG 5628
TGCGGCTGGATCACCTCCTTT 151
CAAGCCATACACCGAAGCTGT 54
GCTCAACGGATAAAAGGTACT 113
GTCCTGGGTGACAGCGTACCT 24
GGAGGCGCGCGATGGTGGGCT 20
GAAGATGCGGGGTTCCTGCGG 47
ACCAGCCACACTGGGACTGAG 10
TCGCATCCTGGGGCTGGAGCA 473
AAAATGCACGCGTAACTTCGG 59
GCGGTTAGACGGAAAGACCCC 63
AAGGTACGGTAACACGTACTG 24
GAGGTGCGAAAGCGTGGGGAG 70
CGTGGGGTCGGAGGTTCAAGT 3281

CTGACGCTGAGGTGCGAAAGC 314
GGAAATCGTTCGTCGAGTGCA 36
CGCTGCGATAAGCGTCGGGGC 19
AAATCGTTCGTCGAGTGCAAT 10
GTCCCGCGTGGAAGGGCCATC 10
TAACTATAACGGTCCTAAGGT 10
GGCGGACACGTTTCTTGGTAA 108
GATGACCCCCAAGAGTCCATA 28
TGATACGCTGCGATAAGCGTC 32
TGAGACACGGACCAGACTCCT 10
ACGCGGACCAGGCCAGTGGCG 22
AATTGGCTGATGGTTTTTGTT 17
TGCAAGTCGAGCGCCCCGCAA 71
GAAGGGCCATCGCTCAACGGA 241
GATCCGACGATTTCCGAATGG 187
GCATGGCTGTCGTCAGCTCGT 1215
GGACTTTTAAGTCAGGGGTGA 31
GAACTGAAACATCTAAGTACC 124
GTCGGTCATAGTGATCCGGTG 216
GGCTAGGGGTGAAAGGCCAAT 24
GTCGTGAGATGTTGGGTTAAG 134
CGGGATGACTTGTGGCTAGGG 16070
CCCGCAAGGGGAGCGGCAGAC 61
ACGATCCAAGCCTAAGTACTC 39
ATGAGTAACGATAAAGGGAGT 29
TCGCAAGACGACGTATAGGGT 24
GCTGTTCGCCATTTAAAGCGG 35
TGATCCAGCCATGCCGCGTGA 19
GCTCAACGGATAAAAGGTACC 16
GTTAGTAGTGGCGAGCGAACG 25
AATACCCCCGGGGGTAGAGCA 17
TAGTACGAGAGGACCGGGATG 153
TCCAAAAGCCGTCTCAGTTCG 21
CGTGGGGTCGGAGGTTCAAGC 11
TTTAAGTCAGGGGTGAAATCC 22
GTATCGGAAGTGAGAATGCTG 23
GGGTAGCGACTGTTTACCAAA 243
GGAGGCGCGCGATGGTAGGCC 70
GCGCCCCGCAAGGGGAGCGGC 90
ATGTACCGGGGCTCAAGCCAT 522
TCCTGGCTCAGAACGAACGCC 24
GCGTGGGGTCGGAGGTTCAAA 16
TTTACGAAAGTCTGCCTGTTC 12
CATGGTTGTCGTCAGCTCGTG 31
AGCAGGTTGAAGGTACGGTAA 313
GCGAAGCGGTTCCAGGAAATA 13
CTAAGGAAGATCGAGAATTGG 45
GGCTCAGAACGAACGCTGGCG 2629
CGAAAGTCGGCCATAGTGATC 10
AATAGCTCGATCGACTTGATC 13
AAGGGCCATCGCTCAACGGAT 861
GTAGTCGATGGGAACCACGTT 59
TTAGAAGCGAACCTGGGGAAC 8677
CCCCGGAACTGCCTTTGATAC 213

TTTAAGAGCTGAGTTTTGATG 11
TATAGGGTGTGACGCCTGCCC 15
GAGAACTGCGTTGAAGGAACT 2395
GGCTCACTGGACCATTACTGA 7627
CGATCCAAGCCTAAGTACTCG 15
ATCTAGAAGCCCGGCACCGCA 30
CCTGGAGGTATCGGAAGTGAG 22
AGAACACCAGTGGCGAAGGCG 13
AACGATAAAGGGAGTGAGAGA 191
GCTCACCAAGGCGACGATCCA 359
CAGCGAAGCGGTTCCAGGAAA 56
GCGGGGTAGAGCAGCCCGGTA 10
TAGTGGCGAGCGAACGCGGAC 101
CAGAACGAACGCTGGCGGCAC 11
GCCGTGAAACGCTCCAGAGCC 10
GCAGTGGGGAATTTTGGACAA 54
AAGAGCTGAGTTTTGATGGAT 286
AACGAGGGCGGCGGCGCCGGC 24
CCAGTGGCGAAGGCGGCTCAC 384
ACCTGAAACCGGATGCCTACA 3765
CCTGGGGAACTGAAACATCTC 14
TAGTTGGTGGGGTAAAGGCTC 40
TGGACAATGGGCGAAAGCCTG 17
GAGACATCCTGGAGGTATCGG 116
TGTTGGAGCAACGCTGGATGG 35
ACTATCCTTCAGTTAGGCTGG 12
GGCTATGGGGACTCACCGTCC 13
CCCGGCTAACTTCGTGCCAGC 50
TCATCGCATCCTGGGGCTGGA 283
GCGGAGCGGCTGCACCCGATG 25
GACATCCTGGAGGTATCGGAA 105
AGCGAACCAGTACCGTGAGGG 192
GAGAACTGCGTTGAAGGAACC 15
GGCTCACTGGACCATTACTGG 13
ACGACTTCCCCGCTGTCTCCA 229
TCACCAAGGCGACGATCCATA 348
GACGGGATAACCGCTGAAGGC 2273
GAAGTAGGGCAATAAGGCAAT 12
CAGGGCGTTCAGTTCGATGCA 294
CACGGAGGCGCGCGATGGTAG 16
AACTTTGGCGGACACGTTTCT 24
GCCCTTGACATCCCGGTCGCG 161
TGAACATGGGTCGACCACGAT 1449
CGAGGGCGGCGGCGCCGGCAG 44
CATTGTCTGCGGATGGTTCGA 33
CATTAAACATTCCGCCTGGGC 10
GCGTCGGGGAGGTGCGAATAC 521
CCCAAGGGTATGGCTGTTCGC 2451
TCGGCTCATCGCATCCTGGGG 17
CACCTCGATGTCGGCTCATCG 10
GGCTATGGGGACTCACCGTCT 940
GTAGCGTTTGCGTCGGTATCT 20
GAGTGAAATAGTACCTGAAAC 15
AACCAGGATGTTGGCTTAGAA 55

GAACCTGCGGCTGGATCACCT 27
GCGGAGCGGCTGCACCCGATA 50
GCCTAACACATGCAAGTCGAG 12
GGGAATTTTGGACAATGGGCG 45
TTTGGTTGCGGGGGCAGGATT 43
GATCTAGCCATGAGCAGGTTG 65
CGGGAGAGTAGGTCGCTGCCA 6024
GGGCCGTAGCTCAGCTGGGAC 17
GGAATTTTGGACAATGGGCGC 43
GCAAGTCGAGCGCCCCGCAAG 108
ACCTGAAACCGGATGCCTACG 40
TCGACCGAATACCCCCGGGGG 4503
GTACCCCGGGGATAACAGGCT 54
ATTGTCTGCGGATGGTTCGAG 19
GGGCAGCGAGCACGCGAGTGT 43
CCTGGGGAACTGAAACATCTT 32
ATCTAAGCGGGAAACCCACCT 159
TCGGCAGACACACGGCGGGTG 334
TCATAGTGATCCGGTGGTCCC 665
ATATTCGTGGGCCTGCAGGTG 163
CTGGACCATTACTGACGCTGT 11
ACCATCTAAGGTCCCTAAGTT 12
CCGTGAAGATGCGGGGTTCCT 52
GCCCTTACGGGCTGGGCTACA 13
CCCCGACGAGGGGAGTGAAAC 12
TGATCCGCCACACTGGGACTG 29
GACATGAGTAACGATAAAGGG 19
GACACGTTTCTTGGTAAGAAC 51
ATCCGGTGGTCCCGCGTGGAA 37
TAAACCGACACTGGTGGACTG 50
ATTGGATTGATCTTGCAGCGA 12
TGGATTGATCTTGCAGCGAAG 19
GCTGGGCTACACACGTGATAC 19
TGGAAGTGCGGCAACGCATGC 28
TACACACCGCCCGTCACACCA 24
CATGCACAGGCGATGAAGGAC 439
GGCGCTGACACGGATTTGACC 20
GGTATCTGGGCTTGTAGCTCA 25
ACACCAGTGGCGAAGGCGGCC 12
ACTCCGGGGATAACAGGCTGA 18
GAGTATGGTAGAGGTGAGTGG 15
TGCACAGGCGATGAAGGACGT 182
TCCCGGGTCTTGTACACACCG 18
ATAAAAGGTACCCCGGGGATA 69
TACGGCCCAGACTCCTACGGG 18
TGTGGATGCACGTATGTGCGT 344
CCTGGCTCAGAACGAACGCTA 15
GCTCAGCTGGGAGAGCACCTG 744
TCCCCGCTGTCTCCAACGCAG 670
CACAGGCGATGAAGGACGTGA 121
GGGTAGCTATATACGGACGGG 22
GATGCACGTATGTGCGTGGTA 11
CTTCGGAAGAAGCGTGACCTC 106
TTGATAGGCCGGGTGTGGAAG 25

TGACAGGATCTGTCCCTAGTA 89
ATGGCGGAGCGGCTGCACCAG 12
TGTAGGATAGGTGGTAGACTT 61
AAGAGTCCATATCGACGGGGT 20
CCAAGGTTTGTCCTGGGTGAC 43
GCATATAGACCGTACCCTAAA 255
ATGAAGCTTACCGGTACTAAT 89
GGGCGTAAAGCGCACGTAGGC 243
GATCTAGAAGCCCGGCACCGC 31
TTTGACTGGGGCGGTCGCCTC 172
AGGCTCAGAACGGTCGGAAAT 281
TGGGCGCAAGCCTGATCCAGC 163
TCAACGGATAAAAGGTACCCC 33
TGGACCATTACTGACGCTGGG 17
TGAAGTCGGAATCGCTAGTAA 238
CTGGACCATTACTGACGCTGC 15
TGAAGGACGTGATACGCTGCG 35
CTGTTCGCCATTTAAAGCGGT 35
GGCATTGTCTGCGGATGGTTC 55
CCTATCAGAGCCGTGGAAGAC 30
GCTCTTTCACCGGTGAAGATA 23
AGTAGGGCAATAAGGCAATAT 10
CGTTCCGTAAGCCTGTGAAGG 18
GGGCTGGGCCACACACGTGCT 12
GGACACAGGTGCTGCATGGCT 12
ACTGGTAGAGAATACCAAGGC 74
CGGACCAGACTCCTACGGGAG 20
CGTGAAATCCTGTCTGAACAT 84
AGAGGATGATCAGCCACACTG 233
ACCATTACTGACGCTGAGGGG 107
TAGCCCAGTGTAGAATGTCTG 37
TCACCGTCTTACTGATCCTAA 155
CCTGGCTCAGAACGAACGCTG 3446
CGGGCTGGGCTACACACGGGC 18
TGATCTAGAAGCCCGGCACCG 41
TGACTGCGTACCTTTTGTATA 12
GTTCAAGTCCTCCCAGGCCCA 130
ACACCAGTGGCGAAGGCGGCT 2721
AGACCGTACCCTAAACCGACA 974
GGAGCGGCTGCACCCGATCCC 1115
GTACTCGTGCATGACCGATAG 30
TACGGGCTGGGCTACACACGA 14
CACAGGTGCTGCATGGCTGTC 40
ACGAGGCGCTGACACGGATTT 57
AACGGCGGCCGTAACAATAAC 12
ATAGACCGTACCCTAAACCGA 1495
ACTGACGCTGAGGTGCGAAAG 1106
GAAGCCCCGGCTAACTTCGTG 98
CGAATGGCGTAACGACTTCCC 11
AGGTATCGGAAGTGAGAATGC 20
TATGGATGTCTAACTGCGGCC 105
CTTGGCCGTGAAACGCTCCAG 15
CGGGCTGGGCTACACACGTGT 12
GGATGACTTGTGGCTAGGGGT 13719

GGCCGTGAAACGCTCGAGCGC 10
AGCGTCGACCGAATACCCCCG 5374
ATGAGCCTGACGAGCTACCGG 53
AAAACTCGACCGAAGTGGGTG 10
CTGAGACACGGCCCAGACTCC 374
ACTGAAACATCTAAGTACCCA 52
AGGCGCAGCGAAAGCGAGTCT 27
GAGACTCCGCTAGTAGTGGCG 14
GTGAAGTCGAAACAAGGTAGC 27
GAAAGTCGGCCATAGTGATCC 10
GGTCCCTAAGTTATGGCTAAG 111
CTGAGCAGGGTTAGCCGGCAC 15
AACTTCGTGCCAGCAGCCGCG 21
ATAGCCCAGTGTAGAATGTCT 15
CCGAAGGCGCTGTGCTAACCC 21
GAGAATGCTGACATGAGTAAC 132
CTCGGCCGTAAAACGCTCCAG 12
AAACCCACCTGAAAACGAGTA 10
AGAGCTGAGTTTTGATGGATA 283
TTGGATGTTTGGTTAGGCGGA 65
ACATGTGTAGGATAGGTGGTC 11
CGTGACGACATGTGTAGGATA 355
ATGAGCAGGTTGAAGGTACGG 477
TTGATCTTGCAGCGAAGCGGT 237
CGGCAAAATGCACGCGTAACT 30
CGATGTCGGCTCATCGCATCC 12
GCCGGGGATAACAGGCTGATG 16
ATCCGCCACACTGGGACTGAG 20
ACCGTATGTGCCCTTCGGGGG 56
GAGGAGGTCGCTGCCAGGTCT 14
ATGCCTACAAACAGTTGGAGC 45
TCGTGAGATGTTGGGTTAAGT 156
TCTGAACATGGGTCGACCACG 1419
AAGTCAGGGGTGAAATCCCGG 32
GGATGACTTGTGGCTAGGGGC 60
CGGGCTGGGCTACACACGTGC 52043
GTCGGCCCATGTGGGCCGCCC 21
CGGAGGCGCGCAATGGTAGGC 10
GCGTAACAGCTCACTGGTCTA 46
CATAACCACCAGGTCGGCGAA 60
CACCAGTGGCGAAGGCGGCTC 2580
TCAGAACGAACGCTGGCGGCC 17
TACCCAGAGGAAAGGACATCA 33
AGCAGCCCGGTAGCTCGTCAG 239
ATCATGTTGGTGTTGAGACGG 22
CGCGGGGTAGAGCAGCCCGGT 10
ACGCCGTAAACGATGAATGTT 182
AGTGATCTAGCCATGAGCAGG 99
CGGGCTGGGCTACACATGTGC 15
CAACGCATGCAGCTTACCGGT 33
ACTGACGCTGAGGTGCGAAAA 13
GTAACCGGAGAAGAAGCCCCG 841
GTAGAGCAGCCCGGTAGCTCG 10
GAGCCCAAGGTTTGTCCTGGG 38

GGAATATTGGACAATGGGCGA 14
CTCGATCGACTTGATCACTCC 410
TGAGACATCCTGGAGGTATCG 114
ATATAGACCGTACCCTAAACC 1550
CGCAGACTCAGTGAAATTGAA 78
CGAGCGAACGCGGACCAGGCG 12
GCGGTACGTGAGTTGGGTTCA 100
GATGTTGGCTTAGAAGCAGCA 28
CGGATGCCTACAAACAGTTGG 47
GCAAGGAGGCAGGCGACCACG 106
GGGGCTGGAGCAGGTCCCAAG 14
GTATAATGGGTCAGCGACTTA 82
AATTCGAAGCAACGCGCAGAA 31
AGAACGGTCGGAAATCGTTCG 63
AAAAGCACCCCGACGAGGGGA 31
CAGGTTGAAGGTACGGTAACA 190
AACCGCTGAAGGCATCTAAGC 10
CGAGACTCCGCTAGTAGTGGC 18
TTTATTTGGTTGCGGGGGCAG 35
CTACGGGAGGCAGCAGTGGGG 44
GGTTAGAAGCGAACCTGGGGA 785
CTGTTTACCAAAAACACAGGG 19
TTACGGGCTGGGCTACACACT 14
GATCTTGCAGCGAAGCGGTTC 223
ACGGGCTGGGCTACACACGCG 13
GCTGTGCTAACCGCAAGGAGG 28
CCGTACCCTAAACCGACACTG 723
CCCAGACTCCTACGGGAGGCG 18
ACGCGGACCAGGCCAGTGGCC 36
TGCGTTGAAGGAACTCGGCAA 349
GCGGCTGGATCACCTCCTTTC 166
GTCGGGTAAGTTCCGACCTGC 24
CTAGAAGCCCGGCACCGCAGA 12
ACCACGATCCAAGCCTAAGTA 37
GGCAGCGAGCACGCGAGTGTG 35
CCTGACTGCAAGACTGACAAG 70
GAATACGTTCCCGGGCCTTGT 41
AGTGCATGAAGTTGGAATCGC 50
TGGACCTGTTGTGGCGCCAGC 3951
TGTTGCAATAGATCGGGATGA 24
TGTGGAAGTGCGGCAACGCAT 81
ACTCAAAGGAATTGACGGGGG 206
GCAGCAGTGGGGAATTTTGGA 43
GGAGGCGCGCGATGGTAGGCG 24
GCCGTAAACGATGAATGTTAG 210
ATTCGGAAGAACACCAGTGGC 42
GTTCAGTTCGATGCATTAGAC 339
CGCTGCGATAAGCGTCGGGGA 57
GGTCGCAAGATTAAAACTCAA 148
AGGTCCCTAAGTTATGGCTAA 112
AGTACTAGTCGGCAGACACAC 18
GCTCAACGGATAAAAGGTACG 90
GAGTGTAGAGGTGAAATTCGT 90
GGCTCAACCCCGGAACTGCCT 35

GCGTAACGACTTCCCCGCTGT 94
CCCAGACTCCTACGGGAGGCA 1289
TACCGGGGAGTACTAGTCGGC 704
ACGCGGACCAGGCCAGTGGCT 6418
GCGTTCAGTTCGATGCATTAG 331
GCGGGAAACCCACCTGAAAAC 16
AAGGGTATGGCTGTTCGCCAT 34
AGCAAACAGGATTAGATACCC 17
GAGACACTATCCTTCAGTTAG 16
TAACTTCGGAAGAAGCGTGAC 27
CTGCTTTGCAAGCAGGGGGTC 91
CCGAGAGGAAGGTGGGGATGA 102
GCGTTGAAGGAACTCGGCAAA 205
TTCTCTTTCTTCATTGTTGAT 18
CCCAGTGTAGAATGTCTGCGC 65
TTACGGGCTGGGCTACACACC 45
GGGGGCCCGCACAAGCGGTGG 207
GAACTGAAACATCTAAGTACA 13
AGCGACTTAGTGTATCGAGCA 103
GGGTCCAGGACCGTGTATGGT 37
GACACGGCCCAGACTCCTACG 290
AGGTCCCTAAGTTATGGCTAG 11
AGGTGAAAAGCACCCCGACGA 28
GAAGTCCTTGAGTAGGGCGGG 744
CCTGCCGCCAGCGTTCGTTCT 10
GTAACGGAGGCGCGCGATGGT 674
GCAGGTCCCAAGGGTTTGGCT 11
GGTAGCGAAATTCCTTGTCGG 400
CGAAGCAACGCGCAGAACCTT 24
GTGTATCGAGCAAGCTTAAGC 32
TCCTCATGGCCCTTACGGGCT 107
AGTACGGTCGCAAGATTAAAA 95
CAGGGTTAGCCGGCCCCTAAG 1014
GAAGTCTTGAGTATGGTAGAG 33
GATGACTTGTGGCTAGGGGAG 15
CAATGAGATTGATCAAGTGTC 11
ACTCAAAGGAATTGACGGGGA 22
GCGTGGGGTCGGAGGTTCAAC 12
AATACCAAGGCGCTTGAGAGA 62
TTAGGTAGAGCGTCGACCGAA 231
ATCCCGGTCCTCTCGTACTAG 10
TGAAATTCGTAGATATTCGGA 281
GGGTAGAGCAGCCCGGTAGCT 10
GGAGGCGCGCGATGGTAGGCA 41
AGCTCAGCTGGGAGAGCGCCT 23
ATTTACTGGGCGTAAAGCGCA 2534
CAGGTCCCAAGGGTTTGGCTG 11
GTAGATATTCGGAAGAACACC 39
ACTCCGAATACCGGGGAGTAC 20
ATGGGGCAACCCACCTTAGAT 31
CATTAAACATTCCGCCTGGGG 596
CACTGGGACTGAGACACGGCC 940
GATGTTGGGTTAAGTCCCGCA 28
CACGTGAAATCCTGTCTGAAC 44

CGGAGATATTCGGAGGAACAC 19
TACTGGGCGTAAAGCGCACGT 1235
GAGTTTGGTTAGGATCAGTAA 14
TTCGGATTGCACTCTGCAACT 109
GATAAAAGGTACTCCGGGGAT 59
CGATAGCGAACCAGTACCGTG 137
GACAGTTCGGTCCCTATCTGC 214
TCAGCGACTTAGTGTATCGAG 182
CCGGGAAACGCTCCAGCGCCA 10
AAGCGTCGGGGAGGTGCGAAT 482
GTGACTGGGGTGAAGTCGTAA 11
TTGGGCACTCTAAGGGGACTG 811
AAGAGATTTTGGACGGTTTAG 13
CAACAACCCTGACCACCATCT 36
GTGGGGAATATTGGACAATGG 65
GGTGAAGTCGGAACAAGGTAG 19
GTTGATAGGCCGGGTGTGGAA 45
CATCTCAGTTCGGATTGCACT 104
CCTGGGGAACTGAAACATCTA 1696
CATTACTGACGCTGAGGTGGG 11
ACCTGAAACCGGATGCCTACC 26
GTGAAATTGAATTCCCCGTGA 28
CAGGCTGATGACCCCCAAGAA 10
GGGCCGTAGCTCAGCTGGGAG 10826
ATTGTATCTCGAGAAGCTGGT 24
CGGGAGAGTAGGTCGCTGCCT 29
ACTGATCCTAACCAAACTCCG 103
GCGGAGCGGCTGCACCCGATT 11
ATGACGTCAAGTCCTCATGGC 27
GTTGGAGCCCAAGGTTTGTCC 86
GCATCTAAGCGGGAAACCCAC 157
GGGAGTACTAGTCGGCAGACA 19
TCGTGAGACAGTTCGGTCTCT 12
TACATAGGGGTTAGAAGCGAA 11
CTGAGTTTTGATGGATATTGG 33
TTGAGAGGATGATCAGCCACA 17
CTCCAAAAGCCGTCTCAGTTC 20
CGGGGTGGAGCAGCCCGGTAG 202
CTTTTGTATAATGGGTCAGCG 33
ATGTCGGCTCATCGCATCCTG 17
CGCCGGGGATAACAGGCTGAT 19
CGGTCCTAAGGTAGCGAAATT 78
GAGAGTAGGTCGCTGCCAGGT 4876
TATGAGCCTGACGAGCTACCG 54
ACCAGTACCGTGAGGGAAAGG 24
CTCAACCCCGGAACTGCCTTT 18
GAGAACTGCGTTGAAGGAACA 20
GATCGGGATGACTTGTGGCTA 427
TGGGGCGGTCGCCTCCTAAAG 15
TGGGCTATGGGGACTCACCGT 1441
TGATCCTAACCAAACTCCGAA 91
AGTGGGAAAGGATGTGAGGAT 136
CAGCCGCGGTAATACGAAGGG 53
AGTGAGAATGCTGACATGAGT 126

GATCGGCCCGCGTTGGATTAG 137
CTTTGATACTGGAAGTCTTGA 20
CCAAAAACACAGGGCTCTGCG 63
GGTGAATACGTTCCCGGGCCT 158
GTTCAGAACGTCGTGAGACAG 101
GCGGAGCGGCTGCACCCGATC 2939
CGTTCCCGGGTCTTGTACACA 13
AGGAATATTGACAGGATCTGT 787
GACCAGGCCAGTGGCTTTTGT 397
TGGTACTTCGTCTCAAGACGC 136
CCCTTACGGGCTGGGCTACAC 194
CATTTGGTGGATGCCTTGGCA 41
CATGCAGCTTACCGGTACTAA 38
TAGGCGGAAGAGATTTTGGAC 37
AATCAAACTTGGAGATAGCTG 15
ATCTAAGTACCCAGAGGAAAG 162
GGCAGTTTGACTGGGGCGGTC 25
ACTGACAAGTCGAGCAGAGAC 51
GGGCCGTAGCTCAGCTGGGAA 72
CAGGCTGATGACCCCCAAGAG 4891
ACGAAAGTCGGTCATAGTGAT 153
CGGGAGAGTAGGTCGCTGCCC 57
GTACGGTCGCAAGATTAAAAC 245
ACGGAGGCGCGCAATGGTAGG 10
CAGAACGAACGCTGGCGGCAG 2407
TATTGGACAATGGGCGAAAGC 43
ATAGATCGGGATGACTTGTGG 496
ACTCGGCAAAATGCACGCGTA 68
GCGTACGGCGCGTGAGCGAGA 16
GAGCGAACGCGGACCAGGCCA 1579
CGAATGGGGCAACCCACCTTA 12
CTGGACCATTACTGACGCTGG 48
AAGGTACCCCGGGGATAACAG 72
GGAGACAGGTGCTGCATGGCT 240
AACACGCACTGGAGGACCGAA 17
TCGTGAAGAGAAGATGTAATC 22
AGAGCAGCCCGGTAGCTCGTC 10
GAGCAGCCCGGTAGCTCGTCA 219
ATTCCGAGTGTAGAGGTGAAA 332
CACGATGAATGTTAGCCGTCG 10
AGGCGACGATCCATAGCTGGT 53
CGGACGGGATAACCGCTGAAG 2412
AACCGCAAGGAGGCAGGCGAC 13
TCCTACGGGAGGCAGCAGTGG 64
CGAGTCTGAACAGGGCGTTCA 80
TACGGGCTGGGCTACACACGT 3149
GTGGTTATGGCGGAGCGGCTG 1032
CGTAGGCGGACTTTTAAGTCA 32
CCTAAGGTAGCGAAATTCCTT 82
GGAGGTATCGGAAGTGAGAAT 13
CGAGGCCGAAAGGCGTAGTCG 13
TCAAAGGAATTGACGGGGACC 22
GACGAAAGTCGGTCATAGTGA 124
ATTCCCTATCAGAGCCGTGGA 50

CAGTCGTGAGACATCCTGGAG 28
AGATACCCTGGTAGTCCACGC 358
CGGTCCAGACTCCTACGGGAG 15
GGGCACTCTAAGGTGACTGCC 18
GCAACCATAGGGGGGTGGCAC 33
GATAACCGCTGAAGGCATCTA 51
GTTGATTTGTGAGTAGTTGGG 10
TTTAAGCAGGAACCCTTGGAC 10
CCGCTGTCTCCAACGCAGACT 810
GTGGAAGTGCGGCAACGCATG 41
GGTGACGGATCGCGTGTGTTG 32
CAAAATGCACGCGTAACTTCG 38
TATGTGCCCTTCGGGGGAAAG 45
CATGAAGTCGGAATCGCTAGT 82
AGTCAGCCTGACGATCGCTTG 19
AAGCGGGAAACCCACCTGAAA 145
ACATGAGTAACGATAAAGGGA 32
ACCAAGTGATCTAGCCATGAG 87
GCTCAGAACGGTCGGAAATCG 556
GCCATAGCTCAGCTGGGAGAG 644
CGGCAGACACACGGCGGGTGC 305
CGGCAGGCTTAACACATGCAA 11
GAGGTGAAATTCGTAGATATT 77
AATGTTAGCCGTCGGGGTGTT 267
GAATGGCGTAACGACTTCCCC 10
CAATCAAACTTGGAGATAGCT 15
AGTCGAGCAGAGACGAAAGTC 44
TAGCCGTAGGGGAACCTGCGG 30
TAGATATTCGGAAGAACACCA 33
CTGGACCATTACTGACGCTGA 22993
GCTCAGGACGAACGCTGGCGG 18
TAAAACTCAAAGGAATTGACG 97
AACCATAGGGGGGTGGCACAG 16
GACCACGATCCAAGCCTAAGT 38
GTCGGCTCATCGCATCCTGGG 15
GCGCTGTGCTAACCGCAAGGA 32
AGGCCCTAGGGTTGTAAAGCT 6990
AGGAACTCGGCAAAATGCACG 595
TCCCTATCTGCCGTGGGTGTA 219
ATCTCAGTTCGGATTGCACTC 114
GTTGGATGTTTGGTTAGGCGG 53
TTTTAAGTCAGGGGTGAAATC 20
AGTTCGGTCCCTATCTGCCGT 958
AGGCATCTAAGCGGGAAACCC 52
ACACCAGTGGCGAAGGCGGCG 10
TGGATGTCTAACTGCGGCCCG 70
GCGAGTCTGAACAGGGCGTTC 125
GGCGTTCAGTTCGATGCATTA 332
CCTAAACCGACACTGGTGGAC 532
AGGTGTAGGCGCAGCGAAAGC 45
GCTGTCGTCAGCTCGTGTCGT 90
GGCTGCGCCTGTTCTTTGCCT 13
GGTCGCGGTTAGTGGAGACAC 64
TGAAGGCAATATGGAAGTAGG 19

ATTCGAAGCAACGCGCAGAAC 49
TCGTTTACGGCGTGGACTACC 12
AGACACTATCCTTCAGTTAGG 16
ACACTATCCTTCAGTTAGGCT 21
AACCCCGGAACTGCCTTTGAT 82
GCTATGTACGGACGGGATAAC 11
GACTTTGAAGCAGGGGCGCCA 687
TTCCGAGTGTAGAGGTGAAAT 357
CGGGCTGGGCTACACACGTGG 52
GGATGACTTGTGGCTAGGGGG 186
GCCGTCTCAGTTCGGATTGCA 22
CAACGGATAAAAGGTACCCCG 49
TACTGATCCTAACCAAACTCC 20
AGCTCACTGGTCTAAATAAGG 18
TGGAGGACCGAACCCATATCT 155
TAGACCGTACCCTAAACCGAC 1537
TTAAGGGCATTTGGTGGATGC 10
GAGGCGCGCGATGGTGGGCTC 21
AACGCATGCAGCTTACCGGTA 40
ACGCCTGCCCGGTGCTGGAAG 224
GCGACTGTTTACCAAAAACAC 21
GGAATCGCTAGTAATCGCGGA 1504
GGTAGCTATATACGGACGGGA 28
AAGGCCTTAGGGTTGTAAAGC 16
CTCACGGGCCGTACCGCAGCT 66
ATCGAACTGAACGCCCTGTTC 34
GTTGGCTTAGAAGCAGCCATC 63
GTACTAGTCGGCAGACACACG 15
GATGTTGGCTTAGAAGCAGCT 22
AGGAGGCAGGCGACCACGGTA 95
CGAGCGAACGCGGACCAGGCC 1731
CTTGATCACTCCCATTTACAA 29
CGGCCCATCAGGGCCGACGGC 41
ACATGTGTAGGATAGGTGGTA 1170
ACTTTGGCGGACACGTTTCTT 134
GGTCATAGTGATCCGGTGGTC 620
GTATCGAGCAAGCTTAAGCCG 18
AGATCGTCGGTTCTTTGAAAA 16
ACTGAAACATCTAAGTACCCT 10
TCAGAACGAACGCTGGCGGCA 2813
CCGGAGACAGGTGCTGCATGG 289
GGATGACTTGTGGCTAGGGGA 33
CATATCGACGGGGTTGTTTGG 80
CGGGCTGGGCTACACACGTGA 89
TACTCTCCCGCGTCTTGAGAC 32
AGGCCAGTGGCTTTTGTGAAT 18
TTTGCGTCGGTATCTGGGCTT 51
GTCCCAAGGGTTTGGCTGTTC 10
AGGGGTGAAATCCCGGGGCTC 30
GTGTCGTGAGATGTTGGGTTA 127
ATAGCTTTACACTGGCATTCG 47
TTACTGACGCTGAGGGGCGAA 27
TTTACTGGGCGTAAAGCGCAC 2240
CGAATACCGGGGAGTACTAGT 31

AAATTCGTAGATATTCGGAGG 170
GATGTTGGCTTAGAAGCAGCC 275
ACCAAACTCCGAATACCGAGG 16
TGACTGATCTAGAAGCCCGGC 43
TACCCTAAACCGACACTGGTG 477
GGACACGTGAAATCCTGTCTG 22
TCTGAGAGGATGATCAGCCAC 130
GGAATATTGGACAATGGGCGC 546
GGTAATACGAAGGGGGCTAGC 130
GCCACACTGGGACTGAGACAC 605
CCGAAGGCGCTGTGCTAACCG 47
TTAGTAGTGGCGAGCGAACGC 25
GTTTGACTGGGGTGGTCGCCT 20
TTGCTACGGAATAACTCAGGG 221
GTGAGAGACTCCCTCGCCGAA 36
TAGATCGTCGGTTCTTTGAAA 16
TCCTTGAGTAGGGCGGGACAC 327
CTCATGGCCCTTACGGGCTGG 37
CAGCGCCAATGGTACTTCGTC 51
TGAGATACGGCCCAGACTCCT 12
CATGACCGATAGCGAACCAGT 139
AGCTCGATCGACTTGATCACT 43
TCGACGGGGTTGTTTGGCACC 1552
TATAGGGTCTGACGCCTGCCC 752
TACGCCGGGGATAACAGGCTG 76
TCTGAGCAGGGTTAGCCGGCA 22
GCTACGGAATAACTCAGGGAA 499
GACCATTACTGACGCTGAGGA 40
GCTCAAGCCATACACCGAAGC 374
TCGTAGATATTCGGAGGAACG 36
TTCGGGGGAAAGATTTATCGG 23
TCGACCACGATCCAAGCCTAA 34
CGTCGGGGTGTTTACACTTCG 93
TAAGGAAGATCGAGAATTGGA 49
ATAACGGTCCTAAGGTAGCGA 39
TGACTGGGGTGAAGTCGTAAC 13
CAAGCCTGATCCAGCCATGCC 214
TGGGTAGTTTGACTGGGGCGG 112
TGATAAGCGTGGGGTCGGAGG 34
AGGTGGTAGACTTTGAAGCAG 114
CGACCGAATACCCCCGGGGGA 10
CCGCCATCACCGATTGTATCT 16
GCATCCTGGGGCTGGAGCAGG 448
AGAGCACTGGATGGGCTATGG 26
GGATGTCTAACTGCGGCCCGT 62
GATCCAAGCCTAAGTACTCGT 15
GTAGAGAATACCAAGGCGCTT 53
TCCCGTACACGTAGAATAGCA 442
GTTTGGCACCTCGATGTCGGC 17
AAGCCGAGAGGAAGGTGGGGA 17
GGAGATATTCGGAGGAACACC 13
GGTTGTCGTCAGCTCGTGTCG 10
TTCCGACCTGCACGAATGGCG 17
AGCAGGGGGTCGTCGGTTCGA 386

CCCGGTAGCTCGTCAGGCTCA 36
GTAAACGATGAATGTTAGCCG 74
GTAAGCCTGTGAAGGGACAGT 15
GACCATTACTGACGCTGAGGG 107
CAGGAGGTTGGCTTAGAAGCA 28
ATCTCGAGAAGCTGGTCTTTC 180
CAACGCATGAAGCTTACCGGT 39
CTGCGATAAGCGTCGGGGAGG 33
GGCCGTGAAACGCTCCAGCGC 35088
TCGTAGATATTCGGAGGAACA 7088
ACGGTCCAGACTCCTACGGGA 15
GTCGCGGTTAGTGGAGACACT 41
TATGGCGGAGCGGCTGCACCA 26
AAGTACTCGTGCATGACCGAT 25
AAGGCTCACCAAGGCGACGAT 12
AGTCTTGAGTATGGTAGAGGT 32
ATGGCTGTCGTCAGCTCGTGT 265
GAGAGGATGATCAGCCACACT 159
AGGCCGGGTGTGGAAGTGCGG 561
GGCCGTGAAACGCTCCGGCGC 10
GTATGTGCGTGGTAGCGGAGC 52
ATTGACAGGATCTGTCCCTAG 291
CGACCGAATACCCCCGGGGGG 20
TCAACGGATAAAAGGTACTCC 79
GTCCTTGAGTAGGGCGGGACC 10
CCGCAGGTTCAAATCCTGCCC 12
ACTGGAAGTCTTGAGTATGGT 39
TGGGAACCACGTTAATATTCG 23
GCTGATGACCCCCAAGAGTCA 21
GAGCAGGGTTAGCCGGCCCCG 34
AAACGAGACTCCGCTAGTAGT 23
CCATCTAAGGTCCCTAAGTTA 12
AGAGGACCGGGATGGACGTAT 4118
ACCAAGGCGCTTGAGAGAACT 124
AGCAGGGTTAGCCGGCCCCTA 1910
GCTCAGTTGGTTAGAGCACAC 18
GCACTGGATGGGCTATGGGGA 1242
GAACTCGGCCGTGAAACGCTC 6828
ACAGGCGATGAAGGACGTGAT 139
GCGCGCGATGGTAGGCTCAGC 12
CCCGTACACGTAGAATAGCAG 393
TGGTTAGGCGGAAGAGATTTT 11
TACCCTTTGATCCGACGATTT 869
TTGTCCTGGGTGACAGCGTAC 61
CCACGATCCAAGCCTAAGTAC 45
GAGTACTAGTCGGCAGACACA 15
ATTAGAACATAGATCGCAGGC 22
CGCCGTAAACGATGAATGTTA 176
ACGTATAGGGCCTGACGCCTG 13
CCGGGTCTTGTACACACCGCC 30
GGATGTTGGCTTAGAAGCAGT 11
TGTGAAGGGACAGTCGTGAGA 35
GGGCTAGGCTACACACGTGCT 16
TGCGAATACCCTTTGATCCGA 20

ATGGCCCTTACGGGCTGGGCT 119
CACTGGTGGACTGGTAGAGAA 1865
ACCACGGTAGGGTCAGCGACT 2108
GGCCATAGCTCAGCTGGGAGA 820
GTGGGAAAGGATGTGAGGATC 183
CCGAAAGGCGTAGTCGATGGG 240
GGCCTGCAGGTGGTGACGGAT 72
TGCAATAGATCGGGATGACTT 47
AACCGACACTGGTGGACTGGT 79
TCGGAAGAAGCGTGACCTCAC 518
ACCTGTTGTGGCGCCAGCCGC 3829
CACGTTAATATTCGTGGGCCT 15
CGTTGTTCGGAATTACTGGGC 10
TTCGATGCATTAGACCCGAAA 34
AAGCGTAACAGCTCACTGGTC 399
CGCGGTGAATACGTTCCCGGG 206
ATGAGAGTGATCAAGTGTCTT 927
ATATTCGGAGGAACACCAGTC 12
GCATGAAGTTGGAATCGCTAG 74
GGATGTTGGCTTAGAAGCAGC 735
AGCGACTGTTTACCAAAAACA 15
GCCGAGAGGAAGGTGGGGATG 172
AACGCGGACCAGGCCAGTGGC 2615
TCAACGGATAAAAGGTACGCC 98
GAATACCCTTTGATCCGACGA 254
CTTTACACTGGCATTCGTGAC 446
ACGATGAATGTTAGCCGTCGG 7096
GATGCGGGGTTCCTGCGGTTA 70
CGAAAGCGAGTCTGAACAGGG 36
ACGTGATACGCTGCGATAAGC 39
CTCTACACTCGGAATTCCACT 10
CCGGGTCCAGGACCGTGTATG 36
GGATTGATCTTGCAGCGAAGC 39
TCCCGGTCGCGGTTAGTGGAG 224
ATTCGGAGGAACACCAGTGGC 2822
ACACACCAACTTCGATCCGAA 14
TCGGAAATCGTTCGTCGAGTG 92
ATAAGCGTGGGGTCGGAGGTT 158
GGCCGTGAAACGCTCCAGAGC 10
AATACCGGGGAGTACTAGTCG 67
TGTAAAGCTCTTTCACCGGTG 821
GTTGGTGGGGTAAAGGCTCAC 21
GTAGGCGCAGCGAAAGCGAGT 18
GTTAGCCGTCGGGGTGTTTAC 90
AGCCGTCGGGGTGTTTACACT 218
ATTGGCTGATGGTTTTTGTTT 20
CGGGCTGGGCTACACACGCGC 15
AGAGAAACGTGGGCGGCATTG 11
TGCACTCTGCAACTCGAGTGC 178
ACAGACCAGGGGGTAGCGACT 133
CCGTGAAACGCTCCAGCGCAA 12
GCAGCGAAGCGGTTCCAGGAG 11
ATCTATTTAGGTAGAGCGTCG 25
GGGCTGGGCTACACACGTGTT 10

GCGAACCTGGGGAACTGAAAG 47
CTCGGCAAAATGCACGCGTAA 49
GGAGGCGCGCGATGGTAGACT 15
GGGCATGAAGTTGGAATCGCT 14
GCCGAAAGGCGTAGTCGATGG 192
ATGCACGTATGTGCGTGGTAG 27
GGTAGAGCAGCCCGGTAGCTC 10
CGCTGGCGGCAGGCTTAACAC 729
TGTTCGCCATTTAAAGCGGTA 34
AAGGACATCAAACGAGACTCC 300
TCGGAATCGCTAGTAATCGCG 90
CGCTGCCAGGTCTGCAAAGCA 10
GAACACCAGTGGCGAAGGCGG 784
CGAACCTGGGGAACTGAAACC 182
GGGGCCGTAGCTCAGCTGGGC 17
GAGACTCCCTCGCCGAAAGTC 22
CCAGTAAACGGCGGCCGTAAC 15
GTCTGACGCCTGCCCGGTGCT 1318
TATGGTGGGTAGTTTGACTGG 35
AGACCACCACGTTGATAGGCC 87
CACCTGCTTTGCAAGCAGGGG 86
ATTACTGACGCTGAGGTGCGG 23
CATCTAAGCGGGAAACCCACC 155
AAGGCGCGCGATGGTAGGCTC 12
GGCGCTGTGCTAACCGCAAGG 33
GTGGGGTAAAGGCTCACCAAG 27
TCGATGTCGGCTCATCGCATC 12
CGAACGCGGACCAGGCCAGTG 2514
CTGGGGTGAAGTCGTAACAAG 34
GGCGGCGGCGCCGGCAGCGGC 48
TGGACAATGGGCGCAAGCCTG 1392
TTCCCCGCTGTCTCCAACGCC 11
GTGGGTGATAGTCCCGTACAC 28
AAACATCTAAGTACCCAGAGG 47
GCAGCGAAGCGGTTCCAGGAA 115
AACGAGCATTTGCAGTCGAAT 11
TGCACGAATGGCGTAACGACT 16
GGAAGAAGCGTGACCTCACTA 29
CTGACAAGTCGAGCAGAGACG 18
GCCGTGGGTGTAGGAATATTG 49
ATCCTGGAGGTATCGGAAGTG 20
GCGAACCTGGGGAACTGAAAA 263
AGGTTGAAGGTACGGTAACAC 137
GAACCAGTACCGTGAGGGAAA 78
GTATAGGGTCTGACGCCTGCC 905
ATTACTGACGCTGAGGTGCGA 8576
GTAGCTCGTCAGGCTCATAAC 83
CGGGGGCCCGCACAAGCGGTG 233
ACTGGTGGACTGGTAGAGAAT 2592
TTTTGTATAATGGGTCAGCGA 36
CCCCCGGGGGTAGAGCACTGG 365
CGAACCTGGGGAACTGAAACT 91
TTGGAGATAGCTGGTTCTCCG 20
GTGAGTGGAATTCCGAGTGTA 214

GTCTGACGCCTGCCCGGTGCC 12
CTTTGATCCGACGATTTCCGA 671
GTGGACCTGTTGTGGCGCCAG 3783
GCAGGGGGTCGTCGGTTCGAT 821
GTTGGAATCGCTAGTAATCGC 1286
AGAAGCGAACCTGGGGAACTA 10
AGGTACGGTAACACGTACTGG 30
TGACGCTGCTGGCCCTGCGCA 23
ATAGATCGCAGGCCAGTCAGC 192
TAAAGCTCTTTCACCGGTGAA 942
GTAGCTCAGCTGGGAGAGCAA 23
CCGAAGTGGGTGATAGTCCCG 325
AGGCTGATGACCCCCAAGAGA 19
GGCCAGTCAGCCTGACGATCG 45
GGAAAGGACATCAAACGAGAC 22
TGTCGACTCATCGCATCCTGG 166
GCAAGATTAAAACTCAAAGGA 118
TGCCACCCCCCTATGGTTGCC 15
CTGGGACTGAGACACGGCCCA 225
CAAGTCGAGCAGAGACGAAAG 32
TAGCGAAATTCCTTGTCGGGT 923
TATTTAGGTAGAGCGTCGACC 21
GTCCATATCGACGGGGTTGTT 47
AAGTTGGAATCGCTAGTAATC 156
TGAAGTCGTAACAAGGTAGCC 52
GCTGACATGAGTAACGATAAA 14
TCGTGAGACAGTTCGGTCCCA 10
TGGTAGTCCACGCCGTAAACG 88
GAAAGTCTGCCTGTTCTGTAT 10
CCGGGATGGACGTATCTCTGG 862
CAATGGGCGCAAGCCTGATCC 119
ACTTAGTGTATCGAGCAAGCT 55
GGGTGTGACGCCTGCCCGGTG 11
GCGCAGAACCTTACCAGCCCG 11
GACTGTTTAGCAAAAACACAG 13
GATGCCTACAAACAGTTGGAG 48
GGAGGCGCGCGATGGTAGCCT 12
CCAAGAGTCCATATCGACGGG 14
GCGGACTTTTAAGTCAGGGGT 116
GATGAATGTTAGCCGTCGGGC 11
TCGTGACGACATGTGTAGGAT 475
AGGGGACTGCCGGTGATAAGC 2202
CGCATGCAGCTTACCGGTACT 166
TATCAGAGCCGTGGAAGACCA 11
TACGGACGGGATAACCGCTGA 583
CAATAGATCGGGATGACTTGT 220
GGAACTCGGCAAATTGCACGC 17
TTGTTGGAGCAACGCTGGATG 39
CGTCGGTTCGATCCCGTCCGG 205
GGCAGCAGTGGGGAATTTTGG 29
AGAAGCGAACCTGGGGAACTG 9556
TAACGACTTCCCCGCTGTCTC 86
ATGCCTTGGCATGCACAGGCG 48
CGATGAATGTTAGCCGTCGGG 7159

ATATTGACAGGATCTGTCCCT 204
TTGGTGGGGTAAAGGCTCACC 26
CAAAACAACCAGGATGTTGGC 86
GCTCTGCGAAGTCGCAAGACG 380
GCGTGAGTGATGAAGGCCCTA 19
AGGCGTAGTCGATGGGAACCA 68
ACGGAAAGACCCCGTGCACCT 215
TCACTGGACCATTACTGACGC 6873
CATTACTGACGCTGAGGGGCG 68
GGCGCGCGATGGTAGGCTCAG 2077
CTAGGGTTGTAAAGCTCTTTC 1785
TGGAATCGCTAGTAATCGCGG 1312
CGGAGTTTGGTTAGGATCAGT 17
GACTGGGGCGGTCGCCTCCTA 62
TGAGAGGATGATCAGCCACAC 153
CAAAAGCCATCTCAGTTCGGA 71
GAGAGGACCGGGATGGACGAA 12
CGGACACGTTTCTTGGTAAGA 65
GAAGTATAGGGTCTGACGCCT 12
TCTAGTCATCATAAATAAGGT 357
TCGGAGGAACACCAGTGGCGC 20
AAGGTACTCCGGGGATAACAG 90
ACGGATAAAAGGTACGCCGGG 1757
GCTGCGATAAGCGTCGGGGAG 73
TCTGAGCAGGGTTAGCCGGCC 11697
AGTCCTTGAGTAGGGCGGGAC 747
GATCAGCCACACTGGGACTGG 10
TCGACGGGGTTGTTTGGCACA 22
ACTTGAGAGTTTGATTCTGGC 11
AGTCCAAGGGTTCCTGCTTAA 10
CGATGGGAACCACGTTAATAT 20
TATGGCGGAGCGGCTGCACCT 22
TGGGCGTAAAGCGCACGTAGG 211
TCGTAGATATTCGGAGGAACT 17
GGCCGTGAAACGCTCCAGCGG 35
GGGGGTAGCGACTGTTTACCA 660
ACCCTGGTAGTCCACGCCGTA 286
GACCATTACTGACGCTGAGGC 35
GGAGGAACACCAGTGGCGAGG 31
TCTGAGCAGGGTTAGCTGGCC 14
GCTACGGAATAACTCAGGGAC 28
TCAAAGGAATTGACGGGGGCC 128
ACCTGGTGGTTATGGCGGAAC 27
TCGCGTAGTAGCGTTTGCGTC 10
GTTCCTGCGGTTAGACGGAAA 121
GGATGATCAGCCACACTGGGA 4504
GACGCTGAGGTGCGAAAGCGT 42
GGCGCAGCGAAAGCGAGTCTG 32
ACCAGGATGTTGGCTTAGAAG 738
AGGGGAACCTGCGGCTGGATC 89
GTAGGAATATTGACAGGATCT 712
ACGTTCCCGGGCCTTGTACAC 121
TTGTTTGGCACCTCGATGTCG 168
GTCGTCAGCTCGTGTCGTGAG 171

ATTTGCTACGGAATAACTCAG 24
CCGCACAAGCGGTGGAGCATG 11
TCGAGTGCAATGGCATAAGCC 56
AACCGGAGAAGAAGCCCCGGC 546
CCCCAGTAAACGGCGGCCGTA 25
GTTCCGTAAGCCTGTGAAGGG 17
GGCCGTGAAACGCTCCAGCGA 25
TCGTAGATATTCGGAGGAACC 148
GCACGAATGGCGTAACGACTT 16
TTGGTTAGGCGGAAGAGATTT 11
ACACGTTTCTTGGTAAGAACT 32
TCGGCCGTGAAACGCTCCACC 13
GTCTGAACATGGGTCGACCAC 291
AGTCCCGTACACGTAGAATAG 411
AAGCTTACCGGTACTAATAGC 91
AGAGTAGGTCGCTGCCAGGGC 10
GACCATTACTGACGCTGAGGT 16772
TGGTGGGTAGTTTGACTGGGG 71
TCGGTCCCTATCTGCCGTGGG 1517
GTTAATCTGAGCAGGGTTAGC 16
GATCAGCCACACTGGGACTGA 1757
AGCCGTCTCAGTTCGGATTGC 29
TACGCCGGGGATAACAGGCTC 22
TTTGGCGGACACGTTTCTTGG 133
TGGGGGTCATCAGCCTGTTAT 15
TCGACGGGGTTGTTTGGCACG 20
GAACGGTCGGAAATCGTTCGT 67
AAAACTCAAAGGAATTGACGG 136
GCTAACGCATTAAACATTCCG 130
AGATGTTGGGTTAAGTCCCGC 28
AATGCTGACATGAGTAACGAT 35
GCTGGGTTCAGAACGTCGTGA 20
TATGGCGGAGCGGCTGCACCC 9189
TCGTTCGTCGAGTGCAATGGC 59
TACCCCGGGGATAACAGGCTG 69
GAGCAGGGTTAGCCGGCCCCT 8588
GCTGATGACCCCCAAGAGTCC 3508
CCTTGTACACACCGCCCGTCA 11
CCGAATGGGGCAACCCACCTT 163
CAGTGGGGAATTTTGGACAAT 47
TCTAAGGAAGATCGAGAATTG 40
GCGAATACCCTTTGATCCGAC 211
TAAAAGGTACGCCGGGGATAA 197
GCGGGGTGGAGCAGCCCGGTA 315
GTCCTTGAGTAGGGCGGGACA 272
CGACCGAATACCCCCGGGGGT 2119
GGTCCAGACTCCTACGGGAGG 15
GAAGGCGGCTCACTGGACCAT 308
GAACTCGGCCGTGAAACGCTA 20
GGCTCAGGACGAACGCTGGCG 18
TTTACACACCAACTTCGATCC 14
AAGTCCCGCAACGAGCGCAAC 95
CTATGTACGGACGGGATAACC 11
AAGTGAGAATGCTGACATGAG 115

ACGCTTGATAAGCGTGGGGTC 17
ATGCTGACATGAGTAACGATA 22
AGGCCGAAAGGCGTAGTCGAT 27
CGCAGCGAAAGCGAGTCTGAA 24
TAGGGGAACCTGCGGCTGGAT 119
ACCAAACTCCGAATACCGGGA 11
GCGGACACGTTTCTTGGTAAG 80
TAGTTTGACTGGGGCGGTCGC 272
GGATGGACGTATCTCTGGTGG 736
GCGCGCGATGGTAGGCTCAGA 1884
TGGCTCAGAGCGAACGCTGGC 10
TGGCACAGACCAGGGGGTAGC 11
GACATCCCGGTCGCGGTTAGT 65
GTCTTGAGTATGGTAGAGGTG 22
ATCTAAGGTCCCTAAGTTATG 41
TGTACCGGGGCTCAAGCCATA 670
GGATGTTGGCTTAGAAGCAGG 19
CCGTGGGTGTAGGAATATTGA 56
ATATTCGGAGGAACACCAGTG 926
GGGGTAGAGCAGCCCGGTAGC 10
ACGGAGGCGCTCGATGGTAGG 10
ACTAATAGCTCGATCGACTTG 10
TGAGCAGGGTTAGCTGGCCCC 12
AGCATACCAAGGCGCTTGAGA 10
CCTCGATGTCGACTCATCGCA 27
GCCGTGAAACGCTCCACCGCC 11
GTACCTTTTGTATAATGGGTC 14
CGCCCCGCAAGGGGAGCGGCA 67
GAAGAACACCAGTGGCGAAGG 20
GCAGGTTCAAATCCTGCCCCC 12
GTAACGACTTCCCCGCTGTCT 87
CGCCAATGGTACTTCGTCTCA 39
TTGGCACCTCGATGTCGACTC 49
TCTGTTGCAATAGATCGGGAT 43
TGTGACGCCTGCCCGGTGCTG 10
ACGCAGACTCAGTGAAATTGA 123
CAGGGCCGACGGCCGGTCGGC 17
CCCGGGGGTAGAGCACTGGAT 233
GCGGAGCGTTCCGTAAGCCTG 22
AGTCGCAAGACGACGTATAGG 17
ACAGCTCACTGGTCTAAATAA 14
GATGGAGCAGCCCGGTAGCTC 15
CCCGGAACTGCCTTTGATACT 245
GGATGTTGGCTTAGAAGCAGA 139
TAGCCGGCCCCTAAGGCGAGG 24
GAGCAGGGTTAGCCGGCCACT 21
AAAACACAGGGCTCTGCGAAG 119
TGATCCTGGCTCAGAACGAAC 1523
GGGCACTCTAAGGGGACTGCC 704
GCTAACTTCGTGCCAGCAGCC 12
AGAGACTCCCTCGCCGAAAGT 21
AGGATAGGTGGTAGACTTTGA 48
TGAGTATGGTAGAGGTGAGTG 33
GATCGCAGGCCAGTCAGCCTG 34

TGTCGTGAGATGTTGGGTTAA 136
ACCAAACTCCGAATACCGGGG 26
CAAATGAATTGACGGGGGCCC 15
GACGATCCATAGCTGGTCTGA 108
CGAGCGCCCCGCAAGGGGAGC 72
GCGTGGGATCGGAGGTTCAAG 13
ATTGCTCACGGGCCGTACCGC 21
TGACATGAGTAACGATAAAGG 16
CTATAACGGTCCTAAGGTAGC 14
AGGTGCGAAAGCGTGGGGAGC 49
GTTATGGCGGAGCGGCTGCAC 1045
AGTACCCAGAGGAAAGGACAT 501
GGTAGAGGTGAGTGGAATTCC 36
GCATTTGGTGGATGCCTTGGC 47
GCTCCTGCATATAGACCGTAC 561
ACGGCCCAAACTCCTACGGGA 12
CCGAACTCGGCCGTGAAACCC 10
CGTCTCCGTGTTTTACATGGG 18
ACCGATTGTATCTCGAGAAGC 11
AACCACGTTAATATTCGTGGG 10
GTAAAGCGCACGTAGGCGGAC 335
GACGAGGCGCTGACACGGATT 49
GCGAACCTGGGGAACTGAAAT 32
TTGATCACTCCCATTTACAAT 17
CCTGACCACCATCTAAGGTCC 44
TTGATCCTGGCTCAGAACGAA 1334
CATCGTTTACGGCGTGGACTA 12
TGGGCCTGCAGGTGGTGACGG 144
ACGCATGCAGCTTACCGGTAC 219
CCCTAAACCGACACTGGTGGA 534
TGCACGCGTAACTTCGGAAGA 18
TGAAGGCCTTAGGGTTGTAAA 15
GTCGCAAGACGACGTATAGGG 21
GGGACTGAGACACGGCCCAGA 178
AGACTCCGCTAGTAGTGGCGA 14
CGTAGCTCAGCTGGGAGAGCA 6438
CCCACCTTAGATGACTAGAAA 54
GACAGGTGCTGCATGGCTGTC 19
ACAGTGGGCAGCGAGCACGCG 413
CTAGTCATCATAAATAAGGTA 81
GGTCTTGTACACACCGCCCGT 10
CAGGGTAGCTATATACGGACG 23
TACTCGTGCATGACCGATAGC 57
TGGAAGTCTTGAGTATGGTAG 29
GTCTGACGCCTGCCCGGTGCG 11
CTGGACCGGAGACAGGTGCTG 625
CCGGTGGTCCCGCGTGGAAGG 43
GGGGCCGTAGCTCAGCTGGGA 12177
TGGATGGGCTATGGGGACTCA 2143
AGGTCCCAAGGGTATGGCTGT 2493
CGAACCTGGGGAACTGAAACA 16442
CATGCCGCGGTGAATACGTTC 226
TAGGGCGGGACACGTGAAATC 16
ATATCGACGGGGTTGTTTGGC 122

CCATATCGACGGGGTTGTTTG 78
GGTTAGTGGAGACACTATCCT 33
CAGGCCAGTGGCTTTTGTGAA 21
ACCGGAGAAGAAGCCCCGGCT 496
AGCGTGGGGTCGGAGGTTCAA 1253
AACTTCGGAAGAAGCGTGACC 42
TTGTCTGCGGATGGTTCGAGA 13
TATGGGGACTCACCGTCTTAC 93
ACATTCCGCCTGGGGAGTACG 1956
AGACTCCCTCGCCGAAAGTCC 17
TCGTCGGCCCATGTGGGCCGC 21
AATCGGATCAACTGAAGAGTT 15
CCTAACACATGCAAGTCGAGC 19
AGTTGGGTTCAGAACGTCGTG 12
ATACGGACGGGATAACCGCTG 276
GCGAACCTGGGGAACTAAAAC 20
TTCCCCGCTGTCTCCAACGCA 458
CAGACGAGGCGCTGACACGGA 11
ATACGAAGGGGGCTAGCGTTG 29
GCGGCAACGCATGCAGCTTAC 31
GTACCCAGAGGAAAGGACATC 496
CGTAGCTCAGCTGGGAGAGCG 27
ATCCTAACCAAACTCCGAATA 62
GGTACTCCGGGGATAACAGGC 84
GGGGTTGTTTGGCACCTCGAT 193
GCGAACCTGGGGAACTGAAAC 34783
CAGGCGATGAAGGACGTGATA 100
ACGAGGGCGGCGGCGCCGGCA 41
CAACCCCGGAACTGCCTTTGA 48
GATGGGAACCACGTTAATATT 19
GCAGCGAAGCGGTTCCAGGAC 23
GATCTGTCCCTAGTACGAGAG 89
GTACCTGAAACCGGATGCATA 11
AGGCGGACTTTTAAGTCAGGG 24
CGAGTGTAGAGGTGAAATTCG 137
TGGGGACTCACCGTCTTACTG 121
GACCGATAGCGAACCAGTACC 139
AATGCACGCGTAACTTCGGAA 101
GAAGTGGGTGATAGTCCCGTA 90
TGAAACGCTCCAGCGCCAATG 169
AGGTGAAATTCGTAGATATTC 116
CCATTACTAACGCTGAGGTGC 10
GGTAGAGCACTGGATGGGCTA 17
CGTATCTCTGGTGGACCTGTT 75
CCCGCAACGAGCGCAACCCTC 116
GTCGCAAGACTAAAACTCAAA 15
GGGGCCGTAGCTCAGCTGGGG 14
GCGCCAATGGTACTTCGTCTC 53
CGAACCTGGGGAACTGAAACG 62
GTCGTCGGTTCGATCCCGTCC 311
GCCGTAGCTCAGCTGGGAGCG 12
TCCACGCCGTAAACGATGAAT 57
GATAGGCCGGGTGTGGAAGTG 60
AACGTCGTGAGACAGTTCGGT 320

TTGACGGGGGCCCGCACAAGC 190
GCCGCGTGAGTGATGAAGGCC 30
GTGATCCGGTGGTCCCGCGTG 10
CGGGAGGCAGCAGTGGGGAAT 15
GTTTGATCCTGGCTCAGAACG 587
TCACTGGTCTAAATAAGGGTC 38
GGAGGTTCAAGTCCTCCCAGG 41
TGTTTGGTTAGGCGGAAGAGA 33
ACGGTCGCAAGACTAAAACTC 10
GGACATCAAACGAGACTCCGC 344
CTTGGAGATAGCTGGTTCTCC 22
AGGCTGATGACCCCCAAGAGC 17
TAAGGGCATTTGGTGGATGCC 10
GCGGTGAATACGTTCCCGGGC 228
GTAGCTCAGCTGGGAGAGCAC 5940
AACCAAACTCCGAATACCGAG 14
AGTGGTTGACAGGTTGGTTTG 10
TCGTGACGACATGTGTAGGAA 18
GATGAATGTTAGCCGTCGGGG 7214
GGTAGGCTCAGAACGGTCGGA 35
TTGACAGGATCTGTCCCTAGT 306
TCGGAGGAACACCAGTGGCGG 31
AAGCAACGCGCAGAACCTTAC 28
GAAATTCGTAGATATTCGGAA 28
AAAGGACATCAAACGAGACTC 143
TAGCGAAATTCCTTGTCGGGG 11
TCGATCCCGTCCGGCTCCACC 1789
CCAAGTGATCTAGCCATGAGC 95
AGCGAACCTGGGGAACTGAGA 26
GCAGGTCCCAAGGGTATGGCT 2359
ATCGACGGGGTTGTTTGGCAC 282
CAACGCAGACTCAGTGAAATT 102
GGGTTGTTTGGCACCTCGATG 192
GCGCAGAACCTTACCAGCCCT 5954
TACGAAGGGGGCTAGCGTTGT 44
AGGCGACCACGGTAGGGTCAG 2019
TTGAAGGCAATATGGAAGTAG 19
GCTTTACACTGGCATTCGTGA 413
GGATGCCTTGGCATGCACAGG 27
ATGGTGGTGACAGTGGGCAGC 139
CCCTTGACATCCCGGTCGCGG 129
ATACCCTGGTAGTCCACGCTG 30
TAGCCATGAGCAGGTTGAAGG 39
AGGCTGATGACCCCCAAGAGT 4642
GTCGAGTGCAATGGCATAAGC 38
TGCATGAAGTTGGAATCGCTA 39
ACCCTGACCACCATCTAAGGT 30
GTACCTGAAACCGGATGCCTA 5119
GGGTTAAGTCCCGCAACGAGC 15
TCCCAAGGGTTTGGCTGTTCG 14
ACGGTCCTAAGGTAGCGAAAT 73
GGTGAATACGTTCCCGGGTCT 11
CCAGGATGTTGGCTTAGAAGC 1770
ATGGGGACTCACCGTCTTACT 89

GAATTTTGGACAATGGGCGCA 38
TGAGCAGGGTTAGCCGGCCAC 24
GGCGATGAAGGACGTGATACG 67
GCATGCACAGGCGATGAAGGA 432
CTGAGGTGCGAAAGCGTGGGG 87
TCCCGGGCCTTGTACACACCG 229
CATCTAAGTACCCAGAGGAAA 165
GGTGGGTAGTTTGACTGGGGC 74
CCAAGAGTCCATATCGACGGC 14
GGGTCAGCGACTTAGTGTATC 213
CGATAAGCGTCGGGGAGGTGC 45
TGAGATGTTGGGTTAAGTCCC 77
CTAAGCGGGAAACCCACCTGA 175
TCACTGGACCATTACTGACGA 26
GGATCTGTCCCTAGTACGAGA 100
GGAGAAGAAGCCCCGGCTAAC 94
GCCGTGAAACGCTCCAGCGGC 34
TGAGTAGGGCGGGACACGTGA 42
CTCAGTTCGGATTGCACTCTG 153
CGGATAAAAGGTACGCCGGGG 1721
GAAGGCCGCAGGTTCAAATCC 17
GCAGCAGTGGGGAATATTGGA 15
CGACGATCCATAGCTGGTCTG 397
TCGTGAGACAGTTCGGTCCCT 295
TCGGAGGAACACCAGTGGCGA 8594
AGTGATCAAGTGTCTTAAGGG 94
TAGGCGCAGCGAAAGCGAGTC 20
GAAATTCGTAGATATTCGGAG 174
CGGATAAAAGGTACGCTGGGG 13
GCGAACCTGGGGAACTGACAC 11
GTTGGGCACTCTAAGGGGACT 1347
GATAGTGAACCAGTACCGTGA 40
AGGTGAGTGGAATTCCGAGTG 62
CGTCGTGAGACAGTTCGGTCC 678
TGGGCTGGGCTACACACGTGC 13
GGTCGTCGGTTCGATCCCGTC 342
AAGCCGTCTCAGTTCGGATTG 26
ATCCTGGGGCTGGAGCAGGTC 512
TGGATGTTTGGTTAGGCGGAA 53
CAGCAGCCGCGGTAATACGAA 11
ACCGGGATGGACGTATCTCTC 40
ACGAAGGGGGCTAGCGTTGTT 528
TTGGGCACTCTAAGGTGACTG 25
ATCAAACTTGGAGATAGCTGG 56
GACCGAAGTGGGTGATAGTCC 255
AACGAACGCTGGCGGCAGGCA 11
GACCTGTTGTGGCGCCAGCCG 3858
TCCAGCCATGCCGCGTGAGTG 15
GGCGCAAGCCTGATCCAGCCA 306
TCTTGCAGCGAAGCGGTTCCA 132
CTTAGTGTATCGAGCAAGCTT 22
GGGTGGAGCAGCTCGGTAGCT 10
TTTGCGGACTTTTACGAAAGT 42
CAGAAGCTCTCCACTAAATCA 12

ACGGAAAGACCCCGTGAACCT 12
ACGCGAGTGTGAGCTAATCTC 10
GGCATTTGGTGGATGCCTTGG 43
GGTTAGACGGAAAGACCCCGT 36
CTCTAAGGGGACTGCCGGTGA 129
ATAATGGGTCAGCGACTTAGT 148
GTTGAAGGAACTCGGCAAAAT 95
ATTGCACTCTGCAACTCGAGT 349
TTGCGTCTCCGTGTTTTACAT 27
GGTTGTTTGGCACCTCGATGT 166
GTTCCCGGGCCTTGTACACAC 98
CCGGGGAGTACTAGTCGGCAG 797
AGCTGGTCTGAGAGGATGATC 56
AGTCGTGAGACATCCTGGAGG 27
TGGGGGTGCGACTGATTATAG 38
CTTCTCATGTTTGTGTTCTTC 23
AAGAACACCAGTGGCGAAGGC 14
ATAACTCAGGGAAACTTGTGC 65
GACCTGTTGTGGCGCCAGCCA 11
CCTTTGATACTGGAAGTCTTG 18
TGACTTGTGGCTAGGGGTGAA 1151
AAGTAGGGCAATAAGGCAATA 10
AGTTTGATCCTGGCTCAGAAC 503
CGTCGGTATCTGGGCTTGTAG 60
CCGGGGCTCAAGCCATACACC 3187
TGAGGGCTTTGGGCAAACATA 10
GTAACTATAACGGTCCTAAGG 10
TCATGGCCCTTACGGGCTGGG 116
CACTGGCATTCGTGACGACAT 211
TTATCGGCAAATGATCGGCCC 133
CGTCGTGAGACAGTTCGGTCT 17
TAGCTATATACGGACGGGATA 77
CGGTCATAGTGATCCGGTGGT 613
GCCCGCGTTGGATTAGCTAGT 93
GAGTGATCAAGTGTCTTAAGG 105
AGAGGTGAGTGGAATTCCGAG 112
CCCAAGGTTTGTCCTGGGTGA 43
ATGGACGTATCTCTGGTGGAC 733
GGCATGAAGTTGGAATCGCTA 14
CCGAACTCGGCCGTGAAACGA 13
GTCATCATAAATAAGGTATCT 10
CTTGTCGGGTAAGTTCCGACC 67
TGAAAAGCACCCCGACGAGGG 35
GATCCTGGCTCAGAACGAACC 13
ATAGTCCCGTACACGTAGAAT 206
GCCTGGGGAGTACGGTCGCAA 1190
CTATGGGGACTCACCGTCTTA 92
GCCAGCAGCCGCGGTAATACG 23
CAGTGGGGAATATTGGACAAT 20
AGAATTGGAAAGAGGCCGGAT 65
TGCGGGGTTCCTGCGGTTAGA 220
ACGTGAAATCCTGTCTGAACA 80
GGGAACTGAAACATCTAAGTA 181
GGTATCGGAAGTGAGAATGCT 17

CGAACCTGGGGAACTAAAACA 14
AGTAGCGTTTGCGTCGGTATC 14
CGCGATGGTAGGCTCAGAACC 23
CGCGGCCCATCAGGGCCGACG 33
CGGTCCCAAGGGTATGGCTGT 10
TCAGCTCGTGTCGTGAGATGT 295
GAGTACGGTCGCAAGATTAAA 111
CCCTGACCACCATCTAAGGTC 40
CGCGCAGAACCTTACCAGCCT 17
ATTCCGAACTCGGCCGTGAAA 999
CGACATGTGTAGGATAGGTGG 1086
CTGATCCAGCCATGCCGCGTG 41
CAGCGAAAGCGAGTCTGAACA 20
TAGGGGTTAGAAGCGAACCTG 326
AATCCTGTCTGAACATGGGTC 1104
AAAAGCCGTCTCAGTTCGGAT 25
CCTATCTGCCGTGGGTGTAGG 159
CAGTTGGAGCCCAAGGTTTGT 77
TGAAGGAACTCGGCAAAATGC 196
GTGCCACCCCCCTATGGTTGC 15
CACGTACTGGAGGACCGAACC 6978
TGGGGTCGGAGGTTCAAGTCC 656
TAGGTCGCTGCCAGGTCTGCA 13
ATTACTGACGCTGAGGGGCGA 35
GAGGGCGGCGGCGCCGGCAGC 40
TATACGGACGGGATAACCGCT 157
GGGGTCGGAGGTTCAAGTCCT 438
ATGAACTTTGGCGGACACGTT 12
ATGGGCTATGGGGACTCACCG 1236
CCGTCGGGGTGTTTACACTTC 126
AGGTAGAGCGTCGACCGAATA 340
CGGCAAATGATCGGCCCGCGT 2515
GGCCGTAGCTCAGCTGGGAAA 12
GAGGGCATGAAGTTGGAATCG 13
GTTCGGATTGCACTCTGCAAC 55
GCCTTAGGGTTGTAAAGCTCT 30
TACAGGTGCTGCATGGCTGTC 12
ACCAAAAACACAGGGCTCTGC 56
TGCGTCTCCGTGTTTTACATG 27
CTTAAGCCGGTAGGTGTAGGC 28
CGCGCAGAACCTTACCAGCCC 6852
CATTACTAACGCTGAGGTGCG 10
TGGAAGTAGGGCAATAAGGCA 28
TTGGAGCAACGCTGGATGGGT 25
TCGCGTGTGTTGTGAGGTCTT 75
AAGGGCATTTGGTGGATGCCT 18
GCTGTCTCCAACGCAGACTCA 40
CTGACCACCATCTAAGGTCCC 36
ACCCGAAGGCGCTGTGCTAAC 194
GCTTGATAAGCGTGGGGTCGG 41
CGACGATTTCCGAATGGGGAA 19
CACGTACTGGAGGACCGAACT 11
CAAGGGGAGCGGCAGACGGGT 20
AAGGGACAGTCGTGAGACATC 38

AAGGTAGCCGTAGGGGAACCT 14
GATGTCTAACTGCGGCCCGTT 57
TCGGCCGTGAAACGCTCCAGA 21
GGGGCCCGCACAAGCGGTGGA 208
GAATACCCCCGGGGGTAGAGC 51
AGTCTGCCTGTTCTGTATGAA 13
CGAGGGCATGAAGTTGGAATC 13
CTTAAGGGCATTTGGTGGATG 10
TCTGGTGGACCTGTTGTGGCG 629
CGCTTGAGAGAACTGCGTTGA 760
GCCATCGCTCAACGGATAAAA 80
GTTGCAATAGATCGGGATGAC 54
TGGTAGACTTTGAAGCAGGGG 262
TCCGGGTCCAGGACCGTGTAT 31
GAAAGGCGTAGTCGATGGGAA 207
AGATATTCGGAGGAACACCAG 1595
AACCTTACCAGCCCTTGACAT 119
GGGCCATCGTTCAACGGATAA 16
AGGTGGTGCATGGCTGTCGTC 11
ATAGTGATCCGGTGGTCCCGC 540
AATACGTTCCCGGGTCTTGTA 10
CTCATCGCATCCTGGGGCTGG 138
CAGGCGACCACGGTAGGGTCA 2026
ATGTCTAACTGCGGCCCGTTA 22
GTGCGGCTGGATCACCTCCTT 37
CGTAGATATTCGGAGGAACAA 11
GGGGTAGAGCACTGGATGGGC 77
TTGCACTCTGCAACTCGAGTG 191
CACGGCCCAGACTCCTACGGG 5126
GCATGAAGCTTACCGGTACTA 76
GTAGCTCAGTTGGTTAGAGCA 114
ATCCCAAAACAACCAGGATGT 53
TATAGACCGTACCCTAAACCG 1581
TTTACCCGAAGGCGCTGTGCT 320
GGATGTGAGGATCCCAAAACA 21
ACGTGCTGGGCTACACACGTG 11
CTCAGAACGGTCGGAAATCGT 514
GCCGGGTGTGGAAGTGCGGCA 438
CTTGGTGGGCCTGGGAGGACT 14
TTCTACACTGGGCTATAAATC 11
CCGTCTCAGTTCGGATTGCAC 10
GTGACGGATCGCGTGTGTTGT 40
TGGCATGCACAGGCGATGAAG 303
TGGGGAGTACGGTCGCAAGAT 43
CGCAAGGAGGCAGGCGACCAC 21
CGTATAGGGCCTGACGCCTGC 11
TCAACTGGAGAGTTTGATCCT 10
GGACCATTACTGACGCTGGGG 17
GTCCCGTACACGTAGAATAGC 431
TAACGGTCCTAAGGTAGCGAA 52
GCGGCCCATCAGGGCCGACGG 41
GCACGTAGGCGGACTTTTAAG 72
CCTTGACATCCCGGTCGCGGT 116
CAACTTGAGAGTTTGATCCTG 25

TGGTTGGATGTTTGGTTAGGC 33
AAAAGGTACCCCGGGGATAAC 75
TCTGTCCCTAGTACGAGAGGA 105
GATATTCGGAGGAACACCAGA 38
ATCGCTAGTAATCGCGGATCA 277
TGAGAGTTTGATCCTGGCTCA 769
TATCTGCCGTGGGTGTAGGAA 20
AATGAGAGTGATCAAGTGTCT 1593
AGCCCTTGACATCCCGGTCGC 86
CGACACTGGTGGACTGGTAGA 406
GCAATGGCATAAGCCTGCCTG 64
GTGGATGCACGTATGTGCGTG 344
TGCGGTTAGACGGAAAGACCC 50
ACTATAACGGTCCTAAGGTAG 12
AGACACGGCCCAGACTCCTAC 201
TTTGGCACCTCGATGTCGACT 93
GGGGAATATTGGACAATGGGC 391
GGGGAGTACTAGTCGGCAGAC 22
TACGGTAACACGTACTGGAGG 62
GGTAAAGGCTCACCAAGGCGA 33
ACGGGCTGGGCCACACACGTG 10
TATCGACGGGGTTGTTTGGCA 197
ACGGGAGGCAGCAGTGGGGAA 30
GCGGGGTGGAGCAGCTCGGTA 10
CGTAACGACTTCCCCGCTGTC 95
ACACGTAGAATAGCAGAAGTC 589
GACTGCAAGACTGACAAGTCG 47
GGCAACGCATGAAGCTTACCG 11
GGGTCGACCACGATCCAAGCC 941
CAGACTCCTACGGGAGGCAGC 591
CATTCCGCCTGGGGAGTACGG 2019
AGTGCAATGGCATAAGCCTGC 21
GGTCTGAGAGGATGATCAGCC 10
GATATTCGGAGGAACACCAGG 22
ACGTACTGGAGGACCGAACCG 12
GAATACCGGGGAGTACTAGTC 38
CGGCTGCACCCGATCCCATTC 14
ACGGAGGCGCGCGACGGTAGG 10
GGAAGATCGAGAATTGGAAAG 1097
GGAACACCAGTGGCGAAGGCG 563
TTACTGACGCTGAGGTGCGAA 7203
CGGTGAATACGTTCCCGGGCC 164
ATGTGCCCTTCGGGGGAAAGA 10
ACGCCGGGGATAACAGGCTGA 24
CACGATCCAAGCCTAAGTACT 41
TTCCGAATGGGGAAACCCACC 18
TTTGCAAGCAGGGGGTCGTCG 53
ACTTGAGAGTTTGATCCTGGC 2618
GTGAGTAACGCGTGGGAACGT 18
TTACACTGGCATTCGTGACGA 416
GAGGCGCGCTATGGTAGGCTC 24
GAGGCGCGCGATGGTAGGGTC 25
GAGAGGACCGGGATGGACGTA 1829
GGCGACGATCCATAGCTGGTC 68

GTTAGACGGAAAGACCCCGTG 76
ATGGGCTGGGCTACACACGTG 12
CGACTTGATCACTCCCATTTA 273
CAGAACCTTACCAGCCCTTGA 1031
CACGCCGTAAACGATGAATGT 135
GCCGTGAAACGCTCCAGCGCA 28
TAACACATGCAAGTCGAGCGG 13
TCACTGGACCATTACTGACCC 16
CTGCCTGACTGCAAGACTGAC 79
CTGGCATTCGTGACGACATGT 151
TTTAGATCGTCGGTTCTTTGA 17
GTCGGAAATCGTTCGTCGAGT 210
CCTAAGTACTCGTGCATGACC 345
CGGCTGGATCACCTCCTTTCT 167
TTGGTTTTACCCGAAGGCGCT 197
CACGGGCCGTACCGCAGCTGA 98
GATGGACGTATCTCTGGTGGA 723
ACGAGACTCCGCTAGTAGTGG 17
GTGATGAAGGCCCTAGGGTTG 64
CCACACTGGGACTGAGACACC 12
CTGCGTTGAAGGAACTCGGCC 23
ATCGGAAGTGAGAATGCTGAC 57
GAGCAGGTTGAAGGTACGGTA 531
GGGCTGGGCTACACACGTGAT 68
GTGTAGGATAGGTGGTAGACC 28
TATCGGAAGTGAGAATGCTGA 21
TGGTGGACTGGTAGAGAATAC 2436
GGGTGAAGTCGTAACAAGGTA 50
CGATCGACTTGATCACTCCCA 366
AAGGCGCTTGAGAGAACTGCG 47
GAACCTGGGGAACTGAAACAC 121
TTGGAAAGAGGCCGGATTTAT 10
ACCGCAAGGAGGCAGGCGACC 20
TGGAGATAGCTGGTTCTCCGC 20
ACCATTACTAACGCTGAGGTG 10
TTCGTCGAGTGCAATGGCATA 40
GATGTTTGGTTAGGCGGAAGA 46
TCCGAACTCGGCCGTGAAACC 46
TTTACACTGGCATTCGTGACG 409
CGGGATGGACGTATCTCTGGT 848
GTCCAGGACCGTGTATGGTGG 10
TGACTGCAAGACTGACAAGTC 72
GCAGGGTTAGCCGGCCCCTAA 1094
CGAAGGCGGCTCACTGGACCA 342
CCTGAAGGCCGCAGGTTCAAA 17
GAGCAGGGTTAGCTGGCCCCT 11
TGATGAGGGGCCGTAGCTCAG 139
ACACCGAAGCTGTGGATGCAC 276
CGTGCTGGGCTACACACGTGC 10
TATGGCTAAGTGGGAAAGGAT 131
GAATCGCTAGTAATCGCGGAT 1455
GTGTAGGATAGGTGGTAGACT 272
AACTCAAATGAATTGACGGGG 12
GTGACTGCGTACCTTTTGTAT 10

TGTTAGCCGTCGGGGTGTTTA 68
CAAGCAGGGGGTCGTCGGTTC 402
GTCAGGGGTGAAATCCCGGGG 36
AGGCGCGCGATGGTAGGCTCC 58
ACTCGGCAAATTGCACGCGTA 10
TCTCAAGACGCGGGAGAGTAG 217
TGAATGTTAGCCGTCGGGGTC 10
TGGACGTATCTCTGGTGGACC 705
ATTTTGGACAATGGGCGCAAG 60
ACCCTTTGATCCGACGATTTC 1244
TAGCCGTCGGGGTGTTTACAC 199
TGTAATCGGATCAACTGAAGA 10
CTGATCCTAACCAAACTCCGA 103
GGTTCAGAACGTCGTGAGACA 120
GGTCCAGGACCGTGTATGGTG 35
GGAGTTGGTTTTACCCGAAGG 25
AACGCTGGCGGCAGGCTTAAC 1746
CAGCTTACCGGTACTAATAGC 47
GATAGGTGGTAGACTTTGAAG 42
CTGGGCCGTGAAACGCTCCAG 13
GGTTTTACCCGAAGGCGCTGT 130
TAGGTAGAGCGTCGACCGAAA 13
GAACCTGGGGAACTGAAACAT 7477
CAGTCAGCCTGACGATCGCTT 20
GCCTGTGAAGGGACAGTCGTG 21
CCAAACTCCGAATACCGGGGA 26
TACACTGGCATTCGTGACGAC 420
CGCTGTCTCCAACGCAGACTC 782
TTGCGGGGGCAGGATTTGAAC 39
GCAGGCGACCACGGTAGGGTC 2079
ACCGGGATGGACGTATCTCTA 13
GATGAGGGGCCGTAGCTCAGC 139
GGATGGAGCAGCCCGGTAGCT 15
ACATGCAAGTCGAGCGCCCCG 21
GCCCCAGTAAACGGCGGCCGT 27
AACGAACGCTGGCGGCAGGCC 15
GGAGAACTGAAACATCTAAGT 10
GGCAGACACACGGCGGGTGCT 201
CGAGTATTCCCTATCAGAGCC 13
AGCGCCAATGGTACTTCGTCT 43
AGCGTTTGCGTCGGTATCTGG 10
TGGTGGGGTAAAGGCTCACCA 26
TTGGAGCCCAAGGTTTGTCCT 117
GCTCGATCGACTTGATCACTC 75
CTGACTGCAAGACTGACAAGT 69
GTGAGGGAAAGGTGAAAAGAA 11
CTTCGTCTCAAGACGCGGGAG 33
AAAGGTACCCCGGGGATAACA 71
AGGACCGGGATGGACGTACCT 10
TACCCGAAGGCGCTGTGCTAA 182
CGGCCGTGAAACGCTCCAGAG 10
CTAGTAGTGGCGAGCGAACGC 58
GATCCTGGCTCAGAACGAACG 1365
GTAGCGAAATTCCTTGTCGGG 786

GGTGGTTATGGCGGAGCGGCT 2798
AAAGCACCCCGACGAGGGGAG 31
GTTGGGCACTCTAAGGTGACT 26
AATTGACGGGGGCCCGCACAA 22
ATAGCGAACCAGTACCGTGAG 100
ACGGATCGCGTGTGTTGTGAG 657
TGGGGTGAAGTCGTAACAAGG 34
GCCGTACCGCAGCTGACGCTG 24
CATCAGGGCCGACGGCCGGTC 39
CTGGGGAGTACGGTCGCAAGA 127
AACGAACGCTGGCGGCAGGCT 3235
ACCGGGATGGACGTATCTCTG 1235
AATCGCTAGTAATCGCGGATC 1398
GATAAGCGTCGGGGAGGTGCG 31
TAACTTCGTGCCAGCAGCCGC 20
CGCTGTGCTAACCGCAAGGAG 26
CCTGGGGCTGGAGCAGGTCCC 13
AGGGCATGAAGTTGGAATCGC 13
AAGGCATCTAAGCGGGAAACC 27
CCCGCGTGGAAGGGCCATCGC 10
GTGAGATGTTGGGTTAAGTCC 92
GCCTGGGGAGTACGGTCGCAC 19
CTTGGGGGTCATCAGCCTGTT 13
AGAGAATACCAAGGCGCTTGA 66
GGCCCTAGGGTTGTAAAGCTC 7146
TTTATCGGCAAATGATCGGCC 79
GATCCTGGCTCAGAACGAACA 19
GCACAGGCGATGAAGGACGTG 239
AGTTAGGCTGGACCGGAGACA 27
AATCTGAGCAGGGTTAGCCGG 215
CCGAACTCGGCCGTGAAACGC 8123
GCCCAGTGTAGAATGTCTGCG 65
GCGACCACGGTAGGGTCAGCG 2125
CGACTGTTTATCAAAAACACA 12
TACCGTATGTGCCCTTCGGGG 13
TAGGTGGTAGACTTTGAAGCA 77
CGGATAAAAGGTACTCCGGGG 43
GGTAGAGCGTCGACCGAATAC 466
GCGCTTGAGAGAACTGCGTTG 761
GGCTAACTTCGTGCCAGCAGC 12
GAGGCCGAAAGGCGTAGTCGA 13
AAGCGTGACCTCACTATGGGC 224
TCGGAGGTTCAAGTCCTCCCA 42
GGGGTCGTCGGTTCGATCCCG 333
CACGTACTGGAGGACCGAACA 34
GTTGTAAAGCTCTTTCACCGG 392
ACGAGGGGAGTGAAACAGTAC 18
CTGACGAGCTACCGGGCTGCT 23
CGGCCCGCGTTGGATTAGCTA 256
ACTCCTACGGGAGGCAGCAGT 91
GGGCTCTGCGAAGTCGCAAGA 803
AAGGATGTGAGGATCCCAAAA 12
CATTCCGAACTCGGCCGTGAA 42
TTCTAGTCATCATAAATAAGG 422

CACTGGATGGGCTATGGGGAC 1247
ACACAGGTGCTGCATGGCTGT 38
TCCAAGCCTAAGTACTCGTGC 47
GGAGGCGCGCGATGGGAGGCT 10
TAGGTCGCTGCCAGGTCTGCC 32
GGCAAAGAACAGGCGCAGCCC 10
ACGAGCTGGGCTACACACGTG 10
TGGGGTCGGAGGTTCAAGTCA 23
CGTTGTTCGGATTTACTGGGC 344
AGTGAAATTGAATTCCCCGTG 34
GACGGGGTTGTTTGGCACCTC 496
GGGGCTAGCGTTGTTCGGATT 790
TTGGCATGCACAGGCGATGAA 297
AGTGGCGAGCGAACGCGGACC 114
TGGATGCCTTGGCATGCACAG 18
CCCGGGCCTTGTACACACCGC 257
AGCGAAGCGGTTCCAGGAAAT 15
TGAGAGGATGATCAGTCACAC 13
CACGTACTGGAGGACCGAACG 25
TCCTGCGGTTAGACGGAAAGA 32
CACCGTCTTACTGATCCTAAC 147
CTGTCTCCAACGCAGACTCAG 34
CCATACACCGAAGCTGTGGAT 31
GCTGGGCTACACACGTGCTAC 12948
AACCAGTACCGTGAGGGAAAG 78
CGCGCAGAACCTTACCAGCCA 10
TAAGCGGGAAACCCACCTGAA 163
TCGGCAAATGATCGGCCCGCG 2529
GTTTTACCCGAAGGCGCTGTG 184
CGCGATGGTAGGCTCAGAACG 776
GGAGCAGCCCGGTAGCTCGTC 252
GTACGTGAGTTGGGTTCAGAA 91
TCCTGTCTGAACATGGGTCGA 2104
ATAAGCGTCGGGGAGGTGCGA 316
TCGGCCGTGAAACGCTCCAGC 36830
ACCACCACGTTGATAGGCCGG 157
TGAGAGTGATCAAGTGTCTTA 558
TTTAGAACGTCGTGAGACAGT 10
GTGGGCAGCGAGCACGCGAGT 243
CTCAAATGAATTGACGGGGGC 16
ATACGGCCCAGACTCCTACGG 18
CAAACGAGACTCCGCTAGTAG 25
CAGGGGGTAGCGACTGTTTAC 1931
TTCGGGGTGGATCTGTGGATC 40
GGGGTCGGAGGTTCAAGTCCA 12
GCAACGCGCAGAACCTTACCA 36
CGGAGGCGCGCGATGGTAGAC 20
GTAACTTCGGAAGAAGCGTGA 11
AGCCGAGAGGAAGGTGGGGAT 39
CGTAGATATTCGGAGGAACAC 7033
CCGCGTGAGTGATGAAGGCCC 22
GATCGTCGGTTCTTTGAAAAC 16
CACCAAGGCGACGATCCATAG 344
AGCAAGCTTAAGCCGGTAGGT 108

CTTACGGGCTGGGCTACACAC 866
CTCCAGCGCCAATGGTACTTC 19
CGTCAGCTCGTGTCGTGAGAT 263
CTGAGAGGATGATCAGCCACA 131
ATGGGCGCAAGCCTGATCCAG 129
CTCAGTGAAATTGAATTCCCC 44
GTTGTGGCGCCAGCCGCATAG 108
GTACGCCGGGGATAACAGGCT 100
GATATTCGGAGGAACACCAGC 23
ACGTACTGGAGGACCGAACCC 6255
ACAGGTGCTGCATGGCTGTCG 703
GGAGATAGCTGGTTCTCCGCG 25
CTGAGAGGATGATCAGTCACA 12
GGTGCTGCATGGCTGTCGTCA 858
GAGATCGAACTGTCACAATGA 10
CAGACTCCTACGGGAGGCAGG 15
GACCGGGATGGACGTATCTCT 4826
CCCTAGGGTTGTAAAGCTCTC 20
TCGTAGATATTCGGAAGAACA 44
GCAGACTCAGTGAAATTGAAT 94
CTGCCTTTGATACTGGAAGTC 28
CCGGCTAACTTCGTGCCAGCA 20
TAACACATGCAAGTCGAGCGC 17
CCACGGTAGGGTCAGCGACTG 2102
GCCGTGAAACGCTCCAGCGCT 11
TGGTTGTCGTCAGCTCGTGTC 22
TAGCGTTGTTCGGATTTACTG 19
GAGTTGGTTTTACCCGAAGGC 32
AAGAAGCCCCGGCTAACTTCG 27
CGTAGAATAGCAGAAGTCCTT 1449
AGCGAACCTGGGGAACTGACA 20
TCGGGTAAGTTCCGACCTGCA 21
TAATCGGATCAACTGAAGAGT 18
CAATGAACCGAAACTTAGAGA 11
CAAGGTTTGTCCTGGGTGACA 33
AGGTACCCCGGGGATAACAGG 65
GATATTCGGAGGAACACCAGT 1532
CCAAGCCTAAGTACTCGTGCA 115
ACTGTTTAGCAAAAACACAGG 13
CCCTAGGGTTGTAAAGCTCTT 2989
ACTAGTCGGCAGACACACGGC 193
TTACCCGAAGGCGCTGTGCTA 225
AGGGTCAGCGACTGGGGTGAA 350
CGTGGGCCTGCAGGTGGTGAC 513
AGCTCTTTCACCGGTGAAGAT 31
AACAGTTGGAGCCCAAGGTTT 15
TCGCTGCCAGGTCTGCAAAGC 10
GTAACAATAACGGTCCTAAGG 13
ATACCCCCGGGGGTAGAGCAC 358
AGCGACTGTTTAGCAAAAACA 12
GATGAAGGACGTGATACGCTG 41
GTCAGCCTGACGATCGCTTGC 18
GAGAGGACCGGGATGGACGTC 12
TAGAGAATACCAAGGCGCTTG 61

GCAGTTTGACTGGGGCGGTCG 21
GAGGATCCCAAAACAACCAGG 54
ATTCGTAGATATTCGGAGGAA 1483
GATTAAAACTCAAAGGAATTG 67
AGACTTTGAAGCAGGGGCGCC 931
CCTACGGGAGGCAGCAGTGGG 55
GAAGAGAAGATGTAATCGGAT 18
GGTTCAAGTCCTCCCAGGCCC 35
CACACCGCCCGTCACACCATG 75
AGGCTGGACCGGAGACAGGTG 636
TCAGAGCCGTGGAAGACCACC 65
GCCGTGAAACGCTCCAGCGCC 33401
GGTGAAATTCGTAGATATTCG 192
GCATGACCGATAGCGAACCAG 136
AAGTGATCTAGCCATGAGCAG 103
CGACGTATAGGGTCTGACGCC 740
TCCTGGGGCTGGAGCAGGTCC 484
CTGCGTTGAAGGAACTCGGCA 1173
TCCAGACTCCTACGGGAGGCA 14
TTAGATCGTCGGTTCTTTGAA 16
AGCTCGTCAGGCTCATAACCT 36
GGCTTGTAGCTCAGTTGGTTA 117
TGAATGTTAGCCGTCGGGGTG 2977
TCGGGGTGGATCTGTGGATCG 13
CCCAAAACAACCAGGATGTTG 99
CGCTAGTAGTGGCGAGCGAAC 55
AGGCGCGCGATGGTAGGCTCG 92
TATTCGGAGGAACACCAGTGG 1242
TTTGAAGCAGGGGCGCCAGCC 547
GGCGGCTCACTGGACCATTAC 1136
CAAGCAGGGGGTCGTCGGTTG 12
GACTGCCGGTGATAAGCCGAG 1100
GGGGCTCAACCCCGGAACTGC 14
AGTGTAGAGGTGAAATTCGTA 84
GTGTAGGATAGGTGGTAGACA 72
GAGACAGTTCGGTCCCTATCT 153
TACACCGAAGCTGTGGATGCA 174
ATTGACGGGGGCCCGCACAAG 143
GAGGTTGGCTTAGAAGCAGCC 40
GCCGGCCCCTAAGGCGAGGCC 16
GCATTAAACATTCCGCCTGGG 626
TGAGTAACGCGTGGGAACGTA 16
GGTGGTAGACTTTGAAGCAGG 135
TTTCCGAATGGGGAAACCCAC 19
TGGTTATGGCGGAGCGGCTGC 937
CGGAAGAAGCGTGACCTCACT 450
AAGATCGAGAATTGGAAAGAG 902
CGTCGAGTGCAATGGCATAAG 35
CCGCAACGAGCGCAACCCTCG 114
AGGGGAGTGAAACAGTACCTG 18
CCACGTTAATATTCGTGGGCC 10
AGGCGGCTCACTGGACCATTA 629
AAGCCTAAGTACTCGTGCATG 133
GAACCTGGGGAACTGAAACAA 111

GACTGACAAGTCGAGCAGAGA 23
AGGATCTGTCCCTAGTACGAG 97
TAGGTAGAGCGTCGACCGAAT 284
TAACAATAACGGTCCTAAGGT 20
AACAACCAGGATGTTGGCTTA 16
CGAACCTGGGGAACTGAAAGA 14
CCCCGTGCACCTTTACTATAG 13
ATAGCAGAAGTCCTTGAGTAG 79
TAAGCCTGTGAAGGGACAGTC 13
GGGCTGGGTTACACACGTGCT 10
AGTCGATGGGAACCACGTTAA 21
GGGGTAGCGACTGTTTACCAA 368
ACTTGGCCGTGAAACGCTCCA 13
CAAGCAGGGGGTCGTCGGTTA 13
TGGCTAAGTGGGAAAGGATGT 118
GACTGCCGGTGATAAGCCGAA 18
CTGGTAGTCCACGCTGTAAAC 22
AGGGAGTGAGAGACTCCCTCG 41
AGGGGGCTAGCGTTGTTCGGA 1009
GCCTACAAACAGTTGGAGCCC 17
GTGTAGGATAGGTGGTAGACG 46
GAGACACGGCCCAGACTCCTA 182
CTGCGTTGAAGGAACTCGGCG 23
GGTAGTCCACGCCGTAAACGA 77
TAGCTGGTCTGAGAGGATGAT 80
CCACACTGGGACTGAGACACG 630
CATCCCGGTCGCGGTTAGTGG 55
AGTACCTGAAACCGGATGCAT 16
CAGGATCTGTCCCTAGTACGA 114
TGTCTGAACATGGGTCGACCA 322
AATAGCTCCTGCATATAGACC 37
GAAAGCGAGTCTGAACAGGGC 38
TGAATGTTAGCCGTCGGGGTA 31
CAAAGGAATTGACGGGGACCC 14
CTAGTTGGTGGGGTAAAGGCT 43
AGGCGCGCGATGGTAGGCTCA 10960
CCACCAGGTCGGCGAAGAACA 25
GGGAGTGAGAGACTCCCTCGC 41
TCGGAAGTGAGAATGCTGACA 127
ACTCGGCCGTAAAACGCTCCA 11
GCAACCCTCGCCCTTAGTTGC 10
GTTCGGTCCCTATCTGCCGTG 1036
TCCGAACTCGGCCGTGAAACG 7625
CGTCGACCGAATACCCCCGGG 5360
AGGTGCTGCATGGCTGTCGTC 780
ACCTGACTTTGGTTTTCGGAT 78
ACCCCCGGGGGTAGAGCACTG 447
CCGCCTGGGGAGTACGGTCGC 3609
TAGGCTGGACCGGAGACAGGT 700
ACTGAGACACGGCCCAGACTC 666
AGTTGGTGGGGTAAAGGCTCA 39
GTCCAGACTCCTACGGGAGGC 12
GAACCTGGGGAACTGAAACAG 14
TTTGGCACCTCGATGTCGGCT 13

GCATTCGTGACGACATGTGTA 33
TCGCTCAACGGATAAAAGGTA 168
TGTATGGTGGGTAGTTTGACT 14
CGACTCATCGCATCCTGGGGC 508
AACTTGGAGATAGCTGGTTCT 173
GCTCACTGGACCATTACTGAG 27
GTGCATGAAGTTGGAATCGCT 39
ATCCATAGCTGGTCTGAGAGG 21
CGGTACTAATAGCTCGATCGC 11
AACAGGCTGATGACCCCCAAG 392
ACGAAGGCGCGCGATGGTAGG 13
GTTGCGGGGGCAGGATTTGAA 35
GTAATCGCGGATCAGCATGTC 20
AAATGTACCGGGGCTCAAGCC 131
GGACCATTACTGACGCTGAGG 17878
GGGCCTGCAGGTGGTGACGGA 140
TAGTCGGCAGACACACGGCGG 188
GCTACACACGTGCTACAATGG 41
CTCGAGAAGCTGGTCTTTCTG 311
ACGAACGCTGGCGGCAGGCTC 16
CAAGCCTAAGTACTCGTGCAT 116
GCCGTCGGGGTGTTTACACTT 165
AGACTCCTACGGGAGGCAGCA 208
TTAGGGTTGTAAAGCTCTTTC 13
GAATATTGGACAATGGGCGCA 532
ATAGGGTGTGACGCCTGCCCG 11
CGGAATCGCTAGTAATCGCGG 81
AGGCAGGCGACCACGGTAGGG 150
CGAAGGCGCTGTGCTAACCGC 55
AAAATGTACCGGGGCTCAAGC 13
ACTCATCGCATCCTGGGGCTG 204
CCTGCGGTTAGACGGAAAGAC 55
GGTGGACTGGTAGAGAATACC 2304
GGATGGGCTATGGGGACTCAC 2504
CGGCCGTGAAACGCTCCGGCG 10
GATAAGCGTGGGGTCGGAGGT 54
CATCGCTCAACGGATAAAAGG 90
AGGTAGCGAAATTCCTTGTCG 372
GTCTCCAACGCAGACTCAGTG 22
CATAAGCCTGCCTGACTGCAA 187
CGACTTCCCCGCTGTCTCCAA 205
CCTTGTCGGGTAAGTTCCGAA 20
TCCCTAGTACGAGAGGACCGG 36
GAGGCGCGCGATGGTAGGATC 47
AACCTGGGGAACTGAAACATA 61
GCTCACTGGACCATTACTGAA 199
AAGTCTTGAGTATGGTAGAGG 29
CACCAGGTCGGCGAAGAACAC 25
CGGGCCGTACCGCAGCTGACG 116
AGAAGCGTGACCTCACTATGG 142
TGAAACATCTAAGTACCCAGA 54
CTTTCTAGTCATCATAAATAA 50
ATGACGGTAACCGGAGAAGAA 67
TAACGATAAAGGGAGTGAGAG 112

TTGGGACTGAGACACGGCCCA 27
CTGTGCTAACCGCAAGGAGGC 27
GGACAATGGGCGCAAGCCTGA 525
ATAAGCCTGCCTGACTGCAAG 219
CTAGTCGGCAGACACACGGCG 194
ACACTGGGACTGAGACACGGC 1073
AGGAAGATCGAGAATTGGAAA 34
TAGGGTCAGCGACTGGGGTGA 272
AGGTTTGTCCTGGGTGACAGC 32
CATTACTGACGCTGAGGTGCC 13
CACCAACTTCGATCCGAAAAC 42
AGACGAGGCGCTGACACGGAT 56
GGACCATTACTGACGCTGAGA 13
TAAGGTAGCGAAATTCCTTGT 151
TGGCTCAGGACGAACGCTGGC 23
ACGAACGCTGGCGGCAGGCTT 5598
GAGTAGTTGGGGGTGGTTTTC 11
CGCTAGTAATCGCGGATCAGC 86
TCCCATTCCGAACTCGGCCGT 131
AAGTCGAGCAGAGACGAAAGT 34
GTGAAAAGCACCCCGACGAGG 35
AAGCCATACACCGAAGCTGTG 29
GGGGACTGCCGGTGATAAGCC 3320
GCGTCGACCGAATACCCCCGT 11
TTTGACTGGGGCGGTCTCCTC 28
GAAGGCGCTGTGCTAACCGCA 19
AATATTCGTGGGCCTGCAGGT 74
GCTGGTTCTCCGCGAAATCTA 12
AAGGGGAGCGGCAGACGGGTG 12
TGGGCGGCATTGTCTGCGGAT 5660
GGACCATTACTGACGCTGCGG 12
CGGTCGCAAGACTAAAACTCA 10
ACTGCCTTTGATACTGGAAGT 38
AGCGAACCTGGAGAACTGAAA 13
CACGGATTTGACCTTCGGGTT 29
CGACGGGGTTGTTTGGCACCT 563
TCAGTTGGGCACTCTAAGGGG 1596
TGGAATTCCGAGTGTAGAGGT 526
GTAACACGTACTGGAGGACCG 146
TTGTGGCTAGGGGTGAAAGGC 821
ATGCACGCGTAACTTCGGAAG 181
GGTTCGATCCCGTCCGGCTCC 657
GGGTAGTTTGACTGGGGCGGT 127
CGAGAGGAAGGTGGGGATGAC 103
GCAAGCTTAAGCCGGTAGGTG 82
CCGTAGCTCAGCTGGGAGAGG 13
TAAGCCGGTAGGTGTAGGCGC 170
AATCGCGGATCAGCATGCCGC 28
GAGAGTTTGATCCTGGCTCAG 469
CTTGTGGCTAGGGGTGAAAGG 938
AACGATGAATGTTAGCCGTCG 2993
TAAAAGGTACCCCGGGGATAA 72
CGTGAAGATGCGGGGTTCCTG 56
CAAAAACACAGGGCTCTGCGA 64

GATGACTTGTGGCTAGGGGTC 11
CTACAATGGTGGTGACAGTGG 11
AGCGTCGGGGAGGTGCGAATA 520
GCCCCGCAAGGGGAGCGGCAG 67
GGAGGCGCGCGATGGTAGGGT 26
GTAGACTTTGAAGCAGGGGCG 787
GGCTCATCGCATCCTGGGGCT 13
GGCTGGAGCAGGTCCCAAGGG 16
CCTGGTGGTTATGGCGGAGCG 3147
GCAATAGATCGGGATGACTTG 241
CGGAAGAACACCAGTGGCGAA 42
ATACCAAGGCGCTTGAGAGAA 90
AGGGGTTAGAAGCGAACCTGG 693
CGCTTGATAAGCGTGGGGTCG 28
ACACACCGCCCGTCACACCAT 74
ACAGTTGGAGCCCAAGGTTTG 80
GTGGAATTCCGAGTGTAGAGG 540
GGAATATTGACAGGATCTGTC 185
TGGGCGGCATTGTCTGCGGAC 11
TGTTTGGCACCTCGATGTCGA 150
AAGTCTGCCTGTTCTGTATGA 11
AGCCCCGGCTAACTTCGTGCC 160
CTGACGCCTGCCCGGTGCTGG 786
GCAACCCACCTTAGATGACTA 23
CTGGCTCAGGACGAACGCTGG 20
GTACGAGAGGACCGGGATGGA 285
CGATGCATTAGACCCGAAACC 24
ACACGTACTGGAGGACCGAAC 7952
TGTATCTCGAGAAGCTGGTCT 19
ACCACGTTGATAGGCCGGGTG 131
TACACGTAGAATAGCAGAAGT 84
GAGAATACCAAGGCGCTTGAG 70
ACCACCAGGTCGGCGAAGAAC 41
TAGTAATCGCGGATCAGCACG 11
GCCTGATCCAGCCATGCCGCG 124
CCTTACCAGCCCTTGACATCC 111
AGTCGGAATCGCTAGTAATCG 245
CCGTAGCTCAGCTGGGAGAGA 34
CCAGGCCAGTGGCTTTTGTGA 56
CCGTAAGCCTGTGAAGGGACA 19
TTGGCTGATGGTTTTTGTTTA 20
GAATAGCAGAAGTCCTTGAGT 253
GACGACGTATAGGGCCTGACG 33
GTCGAGCGCCCCGCAAGGGGA 61
GAGGCGCGCGATGGTAGGCTC 35083
GAATAACTCAGGGAAACTTGT 85
GGTTGGATGTTTGGTTAGGCG 27
GGGATGACTTGTGGCTAGGGC 18
ACGAATGGCGTAACGACTTCC 11
TACCCCCGGGGGTAGAGCACT 395
GAGGACCGGGATGGACGTATC 9471
GTAGATATTCGGAGGAACACT 26
ACTGAGATACGGCCCAGACTC 12
GTGCGGCAACGCATGCAGCTT 21

GTTCCGACCTGCACGAATGGC 17
AGTCGGCAGACACACGGCGGG 186
AGGTCCCAAGGGTTTGGCTGT 11
GTACACACCGCCCGTCACACC 23
TCCCAAGGGTATGGCTGTTCG 2366
GTGCTGCATGGCTGTCGTCAG 778
AAGACGCGGGAGAGTAGGTCG 393
TAAGGCGAGGCCGAAAGGCGT 10
TGCGTACGGCGCGTGAGCGAG 16
TCGTGTCGTGAGATGTTGGGT 192
TGTTGGGTTAAGTCCCGCAAC 23
GCTGGACCGGAGACAGGTGCT 1376
GTGGGTAGTTTGACTGGGGCG 106
GGTGGGGAGTTTGACTGGGGC 148
CTCACCGTCTTACTGATCCTA 170
TACGGGAGGCAGCAGTGGGGA 32
GGGATAACCGCTGAAGGCATC 217
CGAACGCTGGCGGCAGGCTTG 52
TGCTGCATGGCTGTCGTCAGC 806
CACTCTAAGGGGACTGCCGGT 240
GGTACGTGAGTTGGGTTCAGA 89
CCAGCCCTTGACATCCCGGTC 93
CGTCTTACTGATCCTAACCAA 19
GACGATTTCCGAATGGGGAAA 19
CCCTAGTACGAGAGGACCGGG 27
CCAAGGCGCTTGAGAGAACTC 58
TGAGGGGCCGTAGCTCAGCTG 138
AGCGAACCTGGGGAACTAAAA 13
CTGGTTGGATGTTTGGTTAGG 33
CATGCAAGTCGAGCGCCCCGC 21
CGGAAGTGAGAATGCTGACAT 105
GTGGACTGGTAGAGAATACCA 2023
GTAAACGGCGGCCGTAACAAT 10
GGCCGAAAGGCGTAGTCGATG 56
CTATCAGAGCCGTGGAAGACC 25
TCCCAAGGGTATGGCTGTTCA 13
ATGGGTCAGCGACTTAGTGTA 204
CTTGTAGCTCAGTTGGTTAGA 123
CTTGATGAGGGGCCGTAGCTC 152
TCCCTAAGTTATGGCTAAGTG 21
CCGTCTTACTGATCCTAACCA 26
CAATGAACTTTGGCGGACACG 16
GAGGACCGGGATGGACGTATT 11
AGGGCCATCGCTCAACGGATA 360
AGGGCGTTCAGTTCGATGCAT 316
GTAGATATTCGGAGGAACACC 6894
CCGGAGAAGAAGCCCCGGCTA 427
TACCAGCCCTTGACATCCCGG 57
GCAACGCATGCAGCTTACCGG 33
CTAAGGTAGCGAAATTCCTTG 116
AGGACCGGGATGGACGTATCA 12
ATTCGTGACGACATGTGTAGG 279
ACGGAGGCGCGCTATGGTAGG 24
GATTTACTGGGCGTAAAGCGC 2551

GAATTGGAAAGAGGCCGGATT 31
CCAGAGGAAAGGACATCAAAC 22
CCCTGGTAGTCCACGCTGTAA 26
CGTTCGTCGAGTGCAATGGCA 41
TAAACGATGAATGTTAGCCGT 185
GGCAACAACCCTGACCACCAT 23
GCTGGACCGGAGACAGGTGCC 12
ATGCGTACGGCGCGTGAGCGA 16
CCCTGGTAGTCCACGCCGTAA 240
TTCGTGGGCCTGCAGGTGGTG 444
CCGAATACCCCCGGGGGTAGA 223
AGTTTGATCCTGGCTCAGGAC 17
CGAACGCTGGCGGCAGGCTTA 2437
CATCCTGGGGCTGGAGCAGGA 20
GGTGCATGGCTGTCGTCAGCT 16
CGAAGCTGTGGATGCACGTAT 54
CGGTAATACGAAGGGGGCTAG 129
TGAAATCCTGTCTGAACATGG 20
GACGTATAGGGTCTGACGCCT 667
CATCGAACTGAACGCCCTGTT 26
GCGCAAGCCTGATCCAGCCAT 252
GGCACCTCGATGTCGACTCAT 10
GCTATGGGGACTCACCGTCTT 265
TAGCTCAGTTGGTTAGAGCAC 114
GACCATTACTGACGCTGCGGT 12
ATTTATGATGACTAGAAAGCT 13
TAAAGGCTCACCAAGGCGACG 18
CATCCTGGAGGTATCGGAAGT 43
AGGGCATTTGGTGGATGCCTT 29
GACGATTTCCGAATGGGGCAA 17
AGCCGTAGGGGAACCTGCGGC 30
CGGACCAGGCCAGTGGCTTTT 725
CAGCCCGGTAGCTCGTCAGGC 486
CGGTATCTGGGCTTGTAGCTC 66
ACCGGATGCCTACAAACAGTT 18
CGGTGCTGGAAGGTTAAGAGG 13
ATTTGTGAGTAGTTGGGGGTG 15
GCTGGCGGCAGGCTTAACACT 26
GCAATGAGAGTGATCAAGTGT 192
CTAAGGTCCCTAAGTTATGGC 526
TTGTAGCTCAGTTGGTTAGAG 125
GACTCCTACGGGAGGCAGCAG 125
GCGACGATCCATAGCTGGTCT 399
GCGACTGTTTAGCAAAAACAC 13
CTGCGAAGTCGCAAGACGACG 217
GGTACGGTAACACGTACTGGA 32
CACAGACCAGGGGGTAGCGAC 43
TAACTCAGGGAAACTTGTGCT 30
TTCGGATTTACTGGGCGTAAA 577
GCCTGCCCGGTGCTGGAAGGT 40
ACGGGTGAGTAACGCGTGGGA 19
GTCCCAAGGGTATGGCTGTTC 2487
AGAACGTCGTGAGACAGTTCG 268
CTCAGGACGAACGCTGGCGGC 23

GAGTTTGATCCTGGCTCAGGA 14
CCCATCAGGGCCGACGGCCGG 40
CGGCGGCGCCGGCAGCGGCAG 22
CGGGGGTAGAGCACTGGATGG 159
TGGGGAACTGAAACATCTAAC 18
TGGACCATTACTGACGCTGCG 12
GTCTGAGAGGATGATCAGCCA 57
AACACCAGTGGCGAAGGCGGC 921
GGCTCTGCGAAGTCGCAAGAC 753
GAGCACTGGATGGGCTATGGG 51
CTGGTGGTTATGGCGGAGCGG 3036
TGAGAATGCTGACATGAGTAA 144
ACCAGACTCCTACGGGAGGCA 14
CCTGCACGAATGGCGTAACGA 83
GAAACGCTCCAGCGCCAATGG 63
AGCGGCTGCACCCGATCCCAT 29
CTCTGCAACTCGAGTGCATGA 17
CGGGTAGTTTGACTGGGGCGG 14
CGGGGAGGTGCGAATACCCTT 11
CGACTGGGGTGAAGTCGTAAC 12
GCTGGCGGCAGGCTTAACACC 20
ATACACCGAAGCTGTGGATGC 168
ACGGCGGCCGTAACAATAACG 18
GGGCTGGGCTGCACACGTGCT 10
CACCTGAAAACGAGTATTCCC 10
TGAAGATGCGGGGTTCCTGCG 48
TAGAAGCAGCCATCATTTAAA 10
TACCGGGGCTCAAGCCATACA 1056
ACCATTACTGACGCTGAGGCG 30
TACGAAAGTCTGCCTGTTCTG 12
TTCCGTAAGCCTGTGAAGGGA 26
CGGTACTAATAGCTCGATCGA 5449
CTAAGGCGAGGCCGAAAGGCG 11
TGCGATAAGCGTCGGGGAGGT 37
GCTCACTGGACCATTACTGAT 23
CCCCGCTGTCTCCAACGCAGA 775
AACCTGGGGAACTGAAACATT 19
CCTTACGGGCTGGGCTACACA 477
ACCTTTTGTATAATGGGTCAG 24
GTCCCTAAGTTATGGCTAAGT 27
ACCCAGAGGAAAGGACATCAA 14
AGTTCCGACCTGCACGAATGG 17
TAAGGTCCCTAAGTTATGGCT 442
TCAGGATGTTGGCTTAGAAGC 14
CGCAAGATTAAAACTCAAAGG 177
AGGGGCCGTAGCTCAGCTGGG 156
GGATCCCAAAACAACCAGGAT 65
AGACTCAGTGAAATTGAATTC 69
TTACCAAAAACACAGGGCTCT 16
CTGAACATGGGTCGACCACGA 1433
GTGATCTAGCCATGAGCAGGT 102
AGCTATATACGGACGGGATAA 70
CCTGATCCAGCCATGCCGCGT 64
CATTACTGACGCTGAGGTGCG 12668

GGTGGACTGGTAGAGAATACA 22
GCTAGCGTTGTTCGGATTTAC 161
CGGTACGTGAGTTGGGTTCAG 88
CCTGGGGAACTGAAACATCAA 10
AGGAGGTTGGCTTAGAAGCAG 62
GGGCTGGGCTATACACGTGCT 19
CGTCTCAAGACGCGGGAGAGT 157
TGGAAGGGCCATCGCTCAACG 47
AGTGTAGAATGTCTGCGCACG 25
GGGTCTGACGCCTGCCCGGTG 1409
TCGTGCATGACCGATAGCGAA 309
CCTAACCAAACTCCGAATACC 88
GGCCATCGCTCAACGGATAAA 93
CCTTGTCGGGTAAGTTCCGAC 66
TTTCCGAATGGGGCAACCCAC 12
ATAACAGGCTGATGACCCCCA 304
TTCCCCGTGAAGATGCGGGGT 23
TGATGACCCCCAAGAGTCCAT 97
CTAACGCATTAAACATTCCGC 137
AAAGCCGTCTCAGTTCGGATT 24
GTCATAGTGATCCGGTGGTCC 600
ACCGATAGCGAACCAGTACCG 156
GAGCGTCGACCGAATACCCCC 4468
GCCTGCAGGTGGTGACGGATC 238
CCCCGGGGGTAGAGCACTGGA 365
GAGTCCATATCGACGGGGTTG 83
GCTCACTGGACCATTACTGAC 5024
AACCTGGGGAACTGAAACATC 5529
GTCGTCGGCCCATGTGGGCCG 21
ATAACCGCTGAAGGCATCTAA 15
TGACCGATAGCGAACCAGTAC 134
CACACGCTTGATAAGCGTGGG 11
ATAGCTCAGCTGGGAGAGCAC 12
CATTACTGACGCTGAGGTGCA 28
TTTGTCCTGGGTGACAGCGTA 48
CGGGTGTGGAAGTGCGGCAAC 503
GGTGGACTGGTAGAGAATACG 10
GAGACAGGTGCTGCATGGCTG 217
AAAAGCCATCTCAGTTCGGAT 108
ACTGCCGGTGATAAGCCGAGA 598
TTAAAACTCAAAGGAATTGAC 79
GATCCCATTCCGAACTCGGCC 30
AGATGCGGGGTTCCTGCGGTT 51
GAGCTGGGCTACACACGTGCT 12
TACTAATAGCTCGATCGACTT 1024
TTGCAGCGAAGCGGTTCCAGG 110
AACATGGGTCGACCACGATCC 1432
ACGAACGCTGGCGGCAGGCTG 16
GTCGATGGGAACCACGTTAAT 38
ACTGGGACTGAGACACGGCCC 656
GGGCTGGAGCAGGTCCCAAGG 17
GGACCATTACTGACGCTGAGC 12
GGCAACCCACCTTAGATGACT 24
TCAGCGACTGGGGTGAAGTCG 407

AGAGCGTCGACCGAATACCCC 3618
CGTACTGGAGGACCGAACCCA 3130
GGGGTGCGACTGATTATAGCC 16
AGGCGCTGACACGGATTTGAC 46
GCGTCGACCGAATACCCCCGG 5482
CGTATGTGCGTGGTAGCGGAG 56
ATGTTTGGTTAGGCGGAAGAG 31
GACCGGAGACAGGTGCTGCAT 221
CTGCGGCTGGATCACCTCCTT 82
ACGGGCTGGGCTACACACGGG 16
AAGACCACCACGTTGATAGGC 39
CCTGGTGGTTATGGCGGAGCC 28
CAAGTCCTCCCAGGCCCACCA 24
CGAAGGCGCGCGATGGTAGGC 13
GAGGCGCGCGATGGTAGGCTG 14
CGCAAGCCTGATCCAGCCATG 244
AACTTGAGAGTTTGATTCTGG 11
GAGACGAAAGTCGGTCATAGT 108
GCGGCTCACTGGACCATTACT 10694
CCTGCTTTGCAAGCAGGGGGT 91
AAAGGTACGCCGGGGATAACA 114
GCGAACCAGTACCGTGAGGGA 277
CTCTTTCTTCATTGTTGATTG 16
AACCCACCTGAAAACGAGTAT 10
GGAAGAACACCAGTGGCGAAG 33
AATATTGACAGGATCTGTCCC 152
CGAGCGCAACCCTCGCCCTTA 14
TTCCCGGGTCTTGTACACACC 10
GATGACTTGTGGCTAGGGGTA 15
ACACGTACTGGAGGACCGAAG 13
AACTTGAGAGTTTGATCCTGG 62
AGAGGATGATCAGTCACACTG 11
CGGAATAACTCAGGGAAACTT 153
AGGTTCAAGTCCTCCCAGGCC 36
GGGTAAGTTCCGACCTGCACG 17
CGCAGGTTCAAATCCTGCCCC 12
TCGGATTGCACTCTGCAACTC 408
GTAGAGCATACCAAGGCGCTT 10
AGTACGAGAGGACCGGGATGG 186
GGACTGAGACACGGCCCAGAC 144
TAGCTGGTTCTCCGCGAAATC 261
TAAACGGCGGCCGTAACAATA 12
GCGATAAGCGTCGGGGAGGTG 58
TATTGGACAATGGGCGCAAGC 650
TAGTGAACCAGTACCGTGAGG 33
AAGCGTGGGGTCGGAGGTTAA 17
ATCCTGGCTCAGAACGAACGC 1500
AGAATACCAAGGCGCTTGAGA 64
GGGGTGAAGTCGTAACAAGGT 38
CGTAAGCCTGTGAAGGGACAG 18
TGTAGCTCAGCTGGGAGAGCA 26
TGCGGCAACGCATGCAGCTTA 21
TGGCTGATGGTTTTTGTTTAC 20
CGAATACCCCCGGGGGTAGAG 53

GTAGGGTCAGCGACTGGGGTG 196
GATGAAGGCCCTAGGGTTGTA 188
GATCGCGTGTGTTGTGAGGTC 592
CGGTAACCGGAGAAGAAGCCC 961
GGCATGCACAGGCGATGAAGG 328
ACACGTACTGGAGGACCGAAA 50
GATGACTTGTGGCTAGGGGTG 11800
GGCGGAGCGGCTGCACCCGAT 3564
ATGGATGTCTAACTGCGGCCC 166
ACCGACACTGGTGGACTGGTA 419
AGTCCACGCCGTAAACGATGA 24
TTACGAAAGTCTGCCTGTTCT 10
CTTGTGGCTAGGGGTGAAAGC 10
TGTTTACCAAAAACACAGGGC 19
GAGGCGCGCGATGGTAGGCTA 11
GAGTAACGATAAAGGGAGTGA 15
TCCGAATGGGGCAACCCACCT 938
CCGTAGCTCAGCTGGGAGAGC 7271
TGGACCGGAGACAGGTGCTGC 385
AAGGCGTAGTCGATGGGAACC 334
GCGGCTCACTGGACCATTACC 51
ACTCGAGTGCATGAAGTTGGA 165
CGGTAACACGTACTGGAGGAC 414
GCAGCGAGCACGCGAGTGTGA 29
AAACGCTCCAGCGCCAATGGT 49
AGGACCGGGATGGACGTATCT 9150
GTAGATATTCGGAGGAACACG 63
CTGAAACATCTAAGTACCCAG 54
GAGGACCGGGATGGACGTATA 16
GGGATGACTTGTGGCTAGGGA 32
CTGAGCAGGGGTAGCCGGCCC 10
TCCCAAGGGTATGGCTGTTCT 20
CACACTGGGACTGAGACACGG 716
AAGGCAAAGAACAGGCGCAGC 29
ACCGTACCCTAAACCGACACT 962
CCAAGGCGCTTGAGAGAACTG 83
TTGTCGGGTAAGTTCCGACCT 65
TAACGGAGGCGCGCGATGGTA 3412
TCTAGAAGCCCGGCACCGCAG 24
TGTCCCTAGTACGAGAGGACC 58
TGCAATGGCATAAGCCTGCCT 34
CATCCTGGGGCTGGAGCAGGT 413
GTGAAATCCTGTCTGAACATG 69
TACCGGTACTAATAGCTCGAT 506
AAGTCGGTCATAGTGATCCGG 132
GCTGGACCGGAGACAGGTGCG 13
AACATCTAAGTACCCAGAGGA 53
TAGAGCAGCCCGGTAGCTCGT 10
TGTAGCTCAGTTGGTTAGAGC 120
GTCAGCTCGTGTCGTGAGATG 273
TAGGCCGGGTGTGGAAGTGCG 483
CGAGAATTGGAAAGAGGCCGG 71
ATTCTACACTGGGCTATAAAT 10
GCAGTGGGGAATATTGGACAA 17

ATAAAAGGTACGCCGGGGATA 216
CCATCAGGGCCGACGGCCGGT 39
CACCTCGATGTCGACTCATCG 30
GCACCTCGATGTCGACTCATC 53
CGCATGAAGCTTACCGGTACT 72
TGACTGCGAGACTGACAAGTC 10
CTAAGTACCCAGAGGAAAGGA 490
CCCCGTGAAGATGCGGGGTTC 27
ATCGTTTACGGCGTGGACTAC 12
TCCCAAGGGTATGGCTGTTCC 70
GCAAATGATCGGCCCGCGTTG 2131
CACCGAAGCTGTGGATGCACG 201
TTACTGATCCTAACCAAACTC 13
ATCGCAGGCCAGTCAGCCTGA 26
GCGACTTAGTGTATCGAGCAA 119
ACCGTACCCTAAACCGACACC 12
CAGTTTGACTGGGGCGGTCGC 25
GGGAGTTGGTTTTACCCGAAG 16
GTGGTGCATGGCTGTCGTCAG 15
AAGGGGACTGCCGGTGATAAG 560
ATAACCACCAGGTCGGCGAAG 58
GAAAGGACATCAAACGAGACT 19
GTAGATATTCGGAGGAACACA 41
ATGGTGGGTAGTTTGACTGGG 79
TTTGGGCTGCGCCTGTTCTTT 23
TGAAACAGTACCTGAAACCGG 42
GAGGACCGGGATGGACGTATG 22
GAAAACTCGACCGAAGTGGGT 16
GGGATGACTTGTGGCTAGGGG 15069
GTGGAAGGGCCATCGCTCAAC 45
CGGGTCTTGTACACACCGCCC 12
AGAGACGAAAGTCGGTCATAG 84
GAGGCGCTGACACGGATTTGA 51
TTCGGAAGAACACCAGTGGCG 43
GAAGTCGGAATCGCTAGTAAT 274
TAGGCCGGGTGTGGAAGTGCA 13
CAGGGCTCTGCGAAGTCGCAA 1149
TGGCTAGGGGTGAAAGGCCAA 47
TCGGTTCGATCCCGTCCGGCT 769
TCTGCGAAGTCGCAAGACGAC 309
AACGGAGGCGCGCGATGGTAG 12608
CGAACGCTGGCGGCAGGCTTC 22
CGCGGGATGGAGCAGCCCGGT 14
TTAGACGGAAAGACCCCGTGC 305
ACGGATAAAAGGTACTCCGGG 39
ACCCCGACGAGGGGAGTGAAA 12
CCCGACGAGGGGAGTGAAACA 14
GTGAAGAGAAGATGTAATCGG 22
GCTGGACCGGAGACAGGTGCA 10
ACGCGCAGAACCTTACCAGCC 6965
TAGAACATAGATCGCAGGCCA 28
TCTAAGGGGACTGCCGGTGAT 139
CAGTGTAGAATGTCTGCGCAC 36
TATGGAAGTAGGGCAATAAGG 36

TGACGGATCGCGTGTGTTGTG 47
TTCGTCTCAAGACGCGGGAGA 38
GGTTAAGAGGAGAGGTGCAAG 10
CTCAACGGATAAAAGGTACTC 88
CAAGACGACGTATAGGGTCTG 48
GCTGTAGCTCAGCTGGGAGAG 109
AGTAACGATAAAGGGAGTGAG 22
CGGCAACGCATGCAGCTTACC 32
GAAACTCAAAGGAATTGACGG 11
TGGGGAACTGAAACATCTAAG 190
CCTGACTTTGGTTTTCGGATC 91
CGGGGTGGATCTGTGGATCGC 13
GGGTGAAAGGCCAATCAAACT 10
GGACCGGAGACAGGTGCTGCA 303
GGATCGCGTGTGTTGTGAGGT 655
AGAAAGCGTAACAGCTCACTG 193
ACTGGGGTGAAGTCGTAACAA 35
TAACGCATTAAACATTCCGCC 149
GCGGGACACGTGAAATCCTGT 19
ACGGACCAGACTCCTACGGGA 28
ATCAAACGAGACTCCGCTAGT 77
GGGATGGAGCAGCCCGGTAGC 15
GAACATGGGTCGACCACGATC 1444
GGTGGTGACAGTGGGCAGCGA 111
GCAAACAGGATTAGATACCCT 18
ATGTTGGGTTAAGTCCCGCAA 22
GGAGTGAAACAGTACCTGAAA 11
ACTGGCATTCGTGACGACATG 484
GTGGCGAGCGAACGCGGACCA 70
TCTGAACAGGGCGTTCAGTTC 16
CGGGATAACCGCTGAAGGCAC 12
TAAGTCAGGGGTGAAATCCCG 28
AGGTAGCCGTAGGGGAACCTG 15
GGCTCACCAAGGCGACGATCC 227
GTCGGAGGTTCAAGTCCTCCC 44
TGGGGAACTGAAACATCTAAA 19
GTTTGTCCTGGGTGACAGCGT 32
CTCGGCCGGGAAACGCTCCAG 17
CCAATGGTACTTCGTCTCAAG 28
GTGCCAGCAGCCGCGGTAATA 15
CGGACTTTTAAGTCAGGGGTG 107
ACTGTTTACCAAAAACACAGG 20
GCCGGTAGGTGTAGGCGCAGC 220
TGAGCCTGACGAGCTACCGGG 48
ACACGTGAAATCCTGTCTGAA 42
GAAAAGCACCCCGACGAGGGG 31
CGGAGGCGCGCTATGGTAGGC 24
GATGATCCGCCACACTGGGAC 16
GAAGGGGGCTAGCGTTGTTCG 601
GTTGGTTTTACCCGAAGGCGC 93
GCAGGGTAGCTATATACGGAC 31
AAGCTCTTTCACCGGTGAAGA 207
GGGCTGGGCTACACACGCGCT 13
TGTGCCACCCCCCTATGGTTG 15

TGTCTCCAACGCAGACTCAGT 29
TGCCGCGTGAGTGATGAAGGC 26
GCGGGAGAGTAGGTCGCTGAC 10
AACGAGCGCAACCCTCGCCCT 68
AAGCACCCCGACGAGGGGAGT 32
TCGGCCGTGAAACGCTCCGGC 10
CTGGTAGAGAATACCAAGGCG 34
GTCGTGAGACAGTTCGGTCTC 15
TCCAGGACCGTGTATGGTGGG 10
GCATTCAGTTGGGCACTCTAA 26
CGGGATAACCGCTGAAGGCAT 240
ATCAGGGCCGACGGCCGGTCG 26
GCCCAAGGTTTGTCCTGGGTG 38
TCCAGCGCCAATGGTACTTCG 40
GGAACTCGGCAAAATGCACGC 544
CATGGCCCTTACGGGCTGGGC 119
GAACATAGATCGCAGGCCAGT 25
CCTAGGGTTGTAAAGCTCTTT 2159
CAGCAGTGGGGAATTTTGGAC 52
TACTTCGTCTCAAGACGCGGG 38
TCCATAGCTGGTCTGAGAGGA 54
ATGAAGGCCCTAGGGTTGTAA 805
TTTTGTTGGAGCAACGCTGGA 34
GCTGGCGGCAGGCTTAACACA 304
